# Identification of a Different Agonist-Binding Site and Activation Mechanism of the Human P2Y_1_ Receptor

**DOI:** 10.1038/s41598-017-14268-1

**Published:** 2017-10-23

**Authors:** Yang Li, Can Yin, Pi Liu, Dongmei Li, Jianping Lin

**Affiliations:** 1 State Key Laboratory of Medicinal Chemical Biology, College of Pharmacy and Tianjin Key Laboratory of Molecular Drug Research, Nankai University, Haihe Education Park, 38 Tongyan Road, Tianjin, 300353 China; 2 Pharmaceutical Intelligence Platform, Tianjin Joint Academy of Biomedicine and Technology, Tianjin, 300457 China; 3 Biodesign Center, Tianjin Institute of Industrial Biotechnology, Chinese Academy of Sciences, Tianjin, 300308 China

## Abstract

The human P2Y_1_ receptor (P2Y_1_R) is a purinergic G-protein-coupled receptor (GPCR) that functions as a receptor for adenosine 5′-diphosphate (ADP). An antagonist of P2Y_1_R might potentially have antithrombotic effects, whereas agonists might serve as antidiabetic agents. On the basis of the antagonist-bound MRS2500-P2Y_1_R crystal structure, we constructed computational models of apo-P2Y_1_R and the agonist-receptor complex 2MeSADP-P2Y_1_R. We then performed conventional molecular dynamics (cMD) and accelerated molecular dynamics (aMD) simulations to study the conformational dynamics after binding with agonist/antagonist as well as the P2Y_1_R activation mechanism. We identified a new agonist-binding site of P2Y_1_R that is consistent with previous mutagenesis data. This new site is deeper than those of the agonist ADP in the recently simulated ADP-P2Y_1_R structure and the antagonist MRS2500 in the MRS2500-P2Y_1_R crystal structure. During P2Y_1_R activation, the cytoplasmic end of helix VI shifts outward 9.1 Å, the Ser146^3.47^-Tyr237^5.58^ hydrogen bond breaks, a Tyr237^5.58^-Val262^6.37^ hydrogen bond forms, and the conformation of the χ1 rotamer of Phe269^6.44^ changes from parallel to perpendicular to helix VI. The apo-P2Y_1_R system and the MRS2500-P2Y_1_R system remain inactive. The newly identified agonist binding site and activation mechanism revealed in this study may aid in the design of P2Y_1_R antagonists/agonists as antithrombotic/antidiabetic agents, respectively.

## Introduction

The members of the G-protein-coupled receptor (GPCR) superfamily, the largest family of cell-surface receptors^1^, translate chemical information from extracellular signals into interpretable stimuli, thus resulting in intracellular biological responses. The activation of GPCR causes conformational changes in the transmembrane helices, thereby triggering downstream signalling through partners, such as G proteins or β-arrestins, on the intracellular side of the membrane. GPCRs are thought exist in an equilibrium between inactive and active conformations. Agonists stabilize the active conformation, and antagonists stabilize the inactive conformation of these receptors^2^. In addition, GPCRs provide therapeutic targets for a diverse set of human diseases^3,4^ and are the targets of more than 40% of modern drugs^5^.

The P2Y_1_ (P2Y_1_R) and P2Y_12_ (P2Y_12_R) receptors are human purinergic GPCRs and are two of the eight members of the human P2YR family^6^. Both P2Y_1_R and P2Y_12_R can be activated in platelets by the endogenous agonist adenosine 5′-diphosphate (ADP). The activation of each of these receptors facilitates platelet aggregation and plays a vital role in thrombosis formation^7,8^. Accordingly, P2Y_12_R is one of the most important clinical targets for antithrombotic drugs^9^. P2Y_1_R is expressed in a number of different tissues, such as the heart, blood vessels, brain, skeletal muscle and smooth muscles^10^. Recent preclinical data have suggested that antagonists of P2Y_1_R and P2Y_12_R provide equivalent antithrombotic efficacy, whereas a P2Y_1_R antagonist shows potential for decreasing the risk of bleeding^7^. Hence, antagonists of P2Y_1_R might potentially serve as attractive antithrombotic compounds. However, adenosine 5′-triphosphate (ATP) stimulates pancreatic insulin release via a glucose-dependent mechanism involving P2Y_1_R, thus indicating that agonists of P2Y_1_R might have potential as antidiabetic agents^11^.

[^3^H]2-methylthio-adenosine 5′-diphosphate (2MeSADP), a close analogue of the endogenous agonist ADP, is a potent P2Y_1_R agonist (EC_50_ = 1.27 nM)^12^ and has been used in many experimental studies to identify P2Y_1_R antagonists^13,14^. (1′R, 2′S, 4′S, 5′S)-4-(2-Iodo-6-methylaminopurin-9-yl)-1-[(phosphato)methyl]-2(phosphato)bicycle[3.1.0]-hexane (MRS2500) is a P2Y_1_R nucleotide antagonist (IC_50_ = 8.4 nM) and shows strong antithrombotic activity^14,15^. A crystal structure of the agonist 2MeSADP binding with P2Y_12_R (PDBID: 4PXZ), a representative of another P2YR subfamily, has previously been determined^16^. In the 2MeSADP-P2Y_12_R structure, 2MeSADP binds P2Y_12_R within the seven transmembrane helical bundle through electrostatic interactions with phosphate groups. The binding of 2MeSADP involves an inward shift of the extracellular part of helix VI and helix VII towards the center of the seven transmembrane helical bundle. Recently, the crystal structure of antagonist MRS2500-bound P2Y_1_R has been reported by Zhang *et al*. (PDBID: 4XNW)^17^. In this structure, MRS2500 binds to the extracellular vestibule of P2Y_1_R, a pocket composed of residues mainly from the N-terminal, ECL2 and extracellular side of helices VI and VII. However, the crystal structure of agonist-bound P2Y_1_R has not been resolved. Therefore, it is essential to study the conformational dynamics of P2Y_1_R after binding with agonists/antagonists and the activation mechanism of this receptor by using alternative tools.

The conformational dynamics induced by agonist/antagonist and activation mechanism of GPCRs have been extensively studied recent years by many computational chemists using conventional molecular dynamics (cMD) simulations^18–24^. cMD allows studies on timescales of tens to hundreds of nanoseconds, or several microseconds at most; however, many biological processes (*e.g*., the activation process of GPCR) occur over longer timescales of up to milliseconds or more^25^. To overcome the challenge of long timescales and to explore the portions of the energy landscape that are separated by high barriers from the initial minimum, the McCammon group has developed accelerated molecular dynamics (aMD) by introducing a bias potential into cMD^26–28^. In aMD, the system’s potential is modified with a bias boost potential, and the height of local barriers is decreased; thus, the calculation evolves much faster than that in cMD^26–28^. aMD has been successfully used to study many GPCR systems^29–34^, and aMD simulations at hundreds of nanoseconds have been shown to capture events that occur on millisecond scale^29,35^.

While this manuscript was in preparation, Yuan *et al*. published long-timescale cMD simulations on P2Y_1_R using Schrödinger Desmond software^36^. In their simulations, the agonist ADP was first placed 15 Å from the orthosteric site of P2Y_1_R. After 6 × 2 μs cMD simulations, ADP was found to bind to the extracellular vestibule of P2Y_1_R, a similar site to that of the antagonist MRS2500. The activation of P2Y_1_R was characterized by the breaking of an extracellular ionic lock (Asp204^ECL2^-Arg310^7.39^) and the formation of a water channel through the seven transmembrane helical bundle.

Here, we performed cMD and aMD simulations for 2MeSADP-P2Y_1_R, apo-P2Y_1_R and MRS2500-P2Y_1_R embedded in a lipid bilayer/water environment to investigate the conformational dynamics after binding with agonists/antagonists and the P2Y_1_R activation mechanism. Our calculations identified a different agonist-binding site from that of the ADP-P2Y_1_R system in the cMD simulations of Yuan *et al*.^36^. The newly identified agonist-binding site in our simulations is consistent with previous mutagenesis data^6,12,13,17,37,38^. In our aMD simulations, the activation of P2Y_1_R is characterized by (i) the outward shift of helix VI cytoplasmic end of approximately 9.1 Å; (ii) the breaking of the Ser149^3.50^-Tyr237^5.58^ hydrogen bond; (iii) the formation of a Tyr237^5.58^-Val262^6.37^ hydrogen bond; and (iv) a χ1 rotamer change of Phe269^6.44^ from parallel to perpendicular to helix VI. In contrast, the apo-P2Y_1_R system and the MRS2500-P2Y_1_R system remain in the inactive state.

## Results and Discussion

### The binding mode of 2MeSADP in P2Y_1_R

Like other GPCRs, P2Y_1_R can exist in multiple distinct states (*e.g*., active state or inactive state), and apo-P2Y_1_R typically exhibits basal activity. GPCRs exhibit an equilibrium between inactive and active conformations. Agonists stabilize the active conformation, whereas antagonists stabilize the inactive conformation^2^. Therefore, we studied the conformational dynamics of P2Y_1_R in the apo form and in the presence of the agonist 2MeSADP and the antagonist MRS2500 to capture the characteristics of the different P2Y_1_R states.

The currently available structure of P2Y_1_R is the antagonist MRS2500-bound form (PDBID: 4XNW)^17^. In this structure, MRS2500 binds to the extracellular vestibule of P2Y_1_R, a pocket composed of residues mainly from the N-terminal, ECL2 and extracellular side of helices VI and VII.

Initially, we docked 2MeSADP to the MRS2500 binding site (depicted in Supplementary Figure S1). Figure S1 shows that 2MeSADP binds with P2Y_1_R in the same orientation as that of MRS2500. The aromatic adenine ring of 2MeSADP interacts with the hydroxyphenyl group of Tyr303^7.32^ through π-π stacking. The amino group and the N^1^ in adenine of 2MeSADP interact with the amide group of Asn283^6.58^ through hydrogen bonds. The negatively charged pyrophosphates interact with the positively charged amidine groups of Arg128^3.29^ and Arg310^7.39^ through electrostatic interactions and with the phenolic hydroxyl groups of Tyr110^2.63^ and Tyr306^7.35^ through hydrogen bonds. The residues in the 2MeSADP binding site (Supplementary Figure S1) are consistent with previous mutagenesis data^6,12,13,17,37,38^ (Table 1). Recently, Yuan *et al*.^36^ have published long-timescale cMD simulations of the agonist ADP bound with P2Y_1_R. Their simulations showed that ADP binds with P2Y_1_R in a similar site to that of the antagonist MRS2500 in the MRS2500-P2Y_1_R crystal structure^17^. Our identified site for the agonist 2MeSADP (Figure S1) is the same as the simulated ADP binding site^36^ and that of the antagonist MRS2500, as determined by crystallography^17^.

**Table 1:** The effects of the mutated residue on the K_d_ or EC50 value loss compared with those of wild-type P2Y_1_R.

Mutated residue	Position	K_d_ or EC50 value loss	Reference
Y110F	2.63	NS	17
R128A	3.29	>100000	38
H132A	3.33	13	38
Y136A	3.37	10	38
T205A	ECL2	NS	17
T221A	5.42	13	38
T222A	5.43	9	38
K280A	6.55	810	6
N283A	6.58	NS	17
R287A	6.62	11654	38
Y303F	7.32	1.24	17
Y306F	7.35	>300	17
R310A	7.39	329	38

NS: negligible specific binding for [^3^H]2MeSADP in the mutant P2Y_1_R.

**Figure 1:**
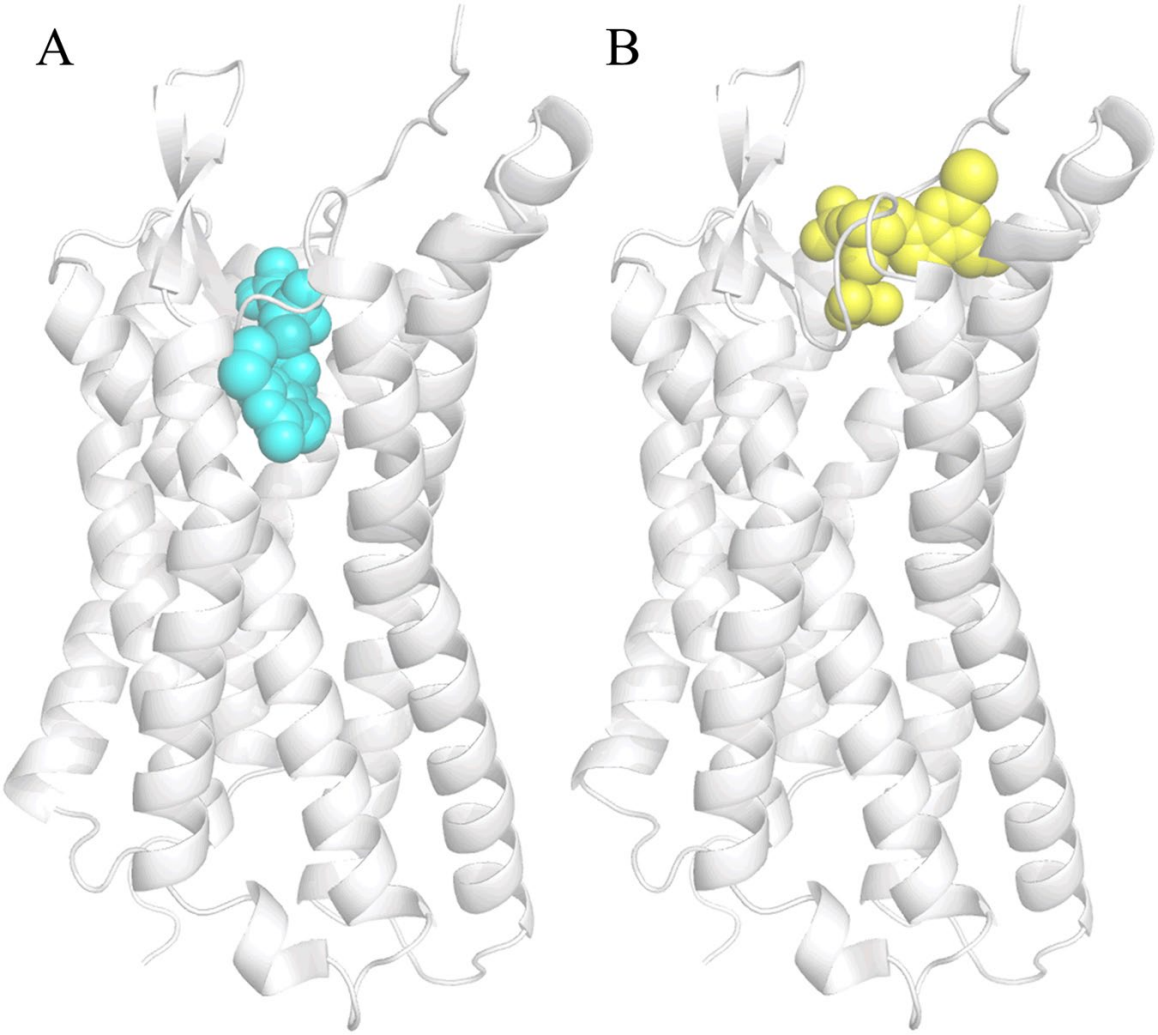
Comparison of the agonist/antagonist-binding sites between (**A**) 2MeSADP-P2Y_1_R and (**B**) MRS2500-P2Y_1_R. The P2Y_1_R structure is shown in cartoon and is colored in silver, 2MeSADP and MRS2500 are shown in sphere and colored in cyan and yellow, respectively.

However, the mutagenesis of residues His132^3.33^, Tyr136^3.37^, Thr222^5.43^ and Lys280^6.55^ decreased the P2Y_1_R binding affinity of 2MeSADP^6,12,13,17,37,38^ (Table 1). These residues are located much deeper than the antagonist MRS2500 binding site in the available P2Y_1_R crystal structure. However, these residues do overlapp with the agonist 2MeSADP binding site in the 2MeSADP-P2Y_12_R crystal structure (PDB ID: 4PXZ)^16^. These observations suggest the possibility of a new potential agonist-binding site distinct from the MRS2500 site in P2Y_1_R. However, the deep cavity in the MRS2500-P2Y_1_R crystal structure is too small to accommodate 2MeSADP. The ECL2 extends deep into the ligand-binding pocket and blocks 2MeSADP from accessing the deep cavity^17^. Therefore, we cut a portion of ECL2, mutated two residues in the deep cavity and induced fit docked 2MeSADP to the deep cavity of P2Y_1_R. Then, the 2MeSADP-P2Y_1_R structure was reconstructed by adding the missing residues in ECL2 and mutating back in the cavity. We then identified a new agonist-binding site in the 2MeSADP-P2Y_1_R structure. Figure 1 shows a comparison of the 2MeSADP and the MRS2500 binding sites between 2MeSADP-P2Y_1_R and MRS2500-P2Y_1_R. Recent long-timescale cMD simulations by Yuan *et al*.^36^ have shown that ADP binds to P2Y_1_R in a similar site to that of the antagonist MRS2500 in the MRS2500-P2Y_1_R crystal structure^17^. However, it can be seen in Figure 1 that the newly identified binding site of the agonist 2MeSADP is deeper than that of ADP in the simulated ADP-P2Y_1_R structure^36^ and the antagonist MRS2500 in the MRS2500-P2Y_1_R crystal structure^17^.

**Figure 2:**
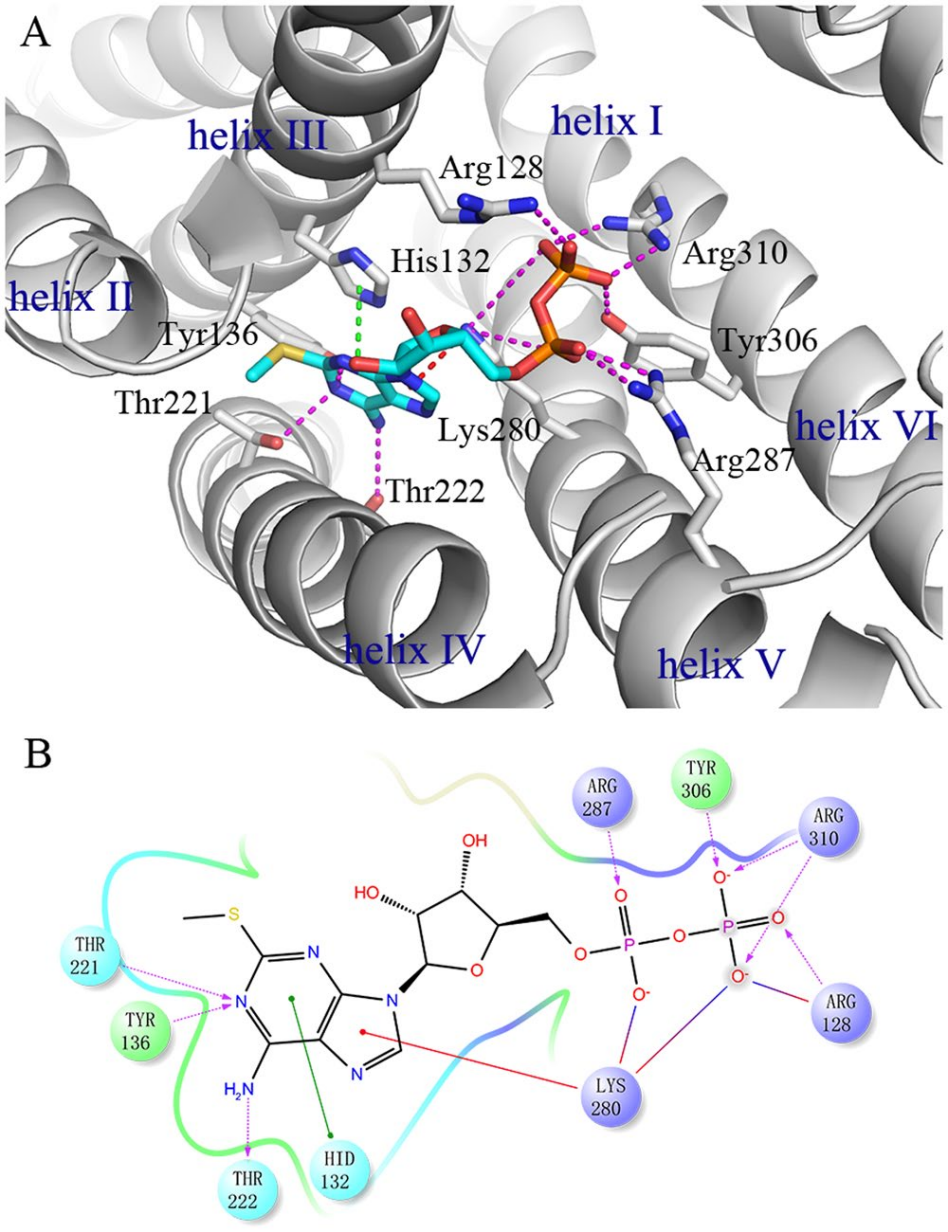
(**A**) Binding mode of 2MeSADP in P2Y_1_R. Hydrogen bonds between 2MeSADP and P2Y_1_R are represented by dashed lines. (**B**) Schematic representation of interactions between 2MeSADP and P2Y_1_R.

The specific binding mode of 2MeSADP in P2Y_1_R is depicted in Figure 2 and the interactions between 2MeSADP and P2Y_1_R over time are shown in Supplementary Figure S2. The aromatic adenine ring of 2MeSADP interacts with the imidazole group of His132^3.33^ and with the ε-amino group of Lys280^6.55^ through π-π and π-cation stacking. In addition, the amino group in the adenine of 2MeSADP forms a hydrogen bond with the hydroxyl group of Thr222^5.43^. The N^1^ in the adenine of 2MeSADP forms hydrogen bonds with the phenolic hydroxyl group of Tyr136^3.37^ and the hydroxyl group of Thr221^5.42^. The negatively charged pyrophosphates interact strongly with several positively charged or polar residues, including Arg128^3.29^, Arg287^6.62^, Arg310^7.39^, Lys280^6.55^ and Tyr306^7.35^. The residues shown in the 2MeSADP binding site in Figure 2 have been demonstrated to be important for the binding of this ligand to P2Y_1_R and show 9 or more fold changes in K_d_ or EC50 values in previous mutation studies^6,12,13,17,37,38^ (Table 1). This result indicates that the newly identified 2MeSADP binding site in our simulation is consistent with previous mutagenesis data.

**Figure 3:**
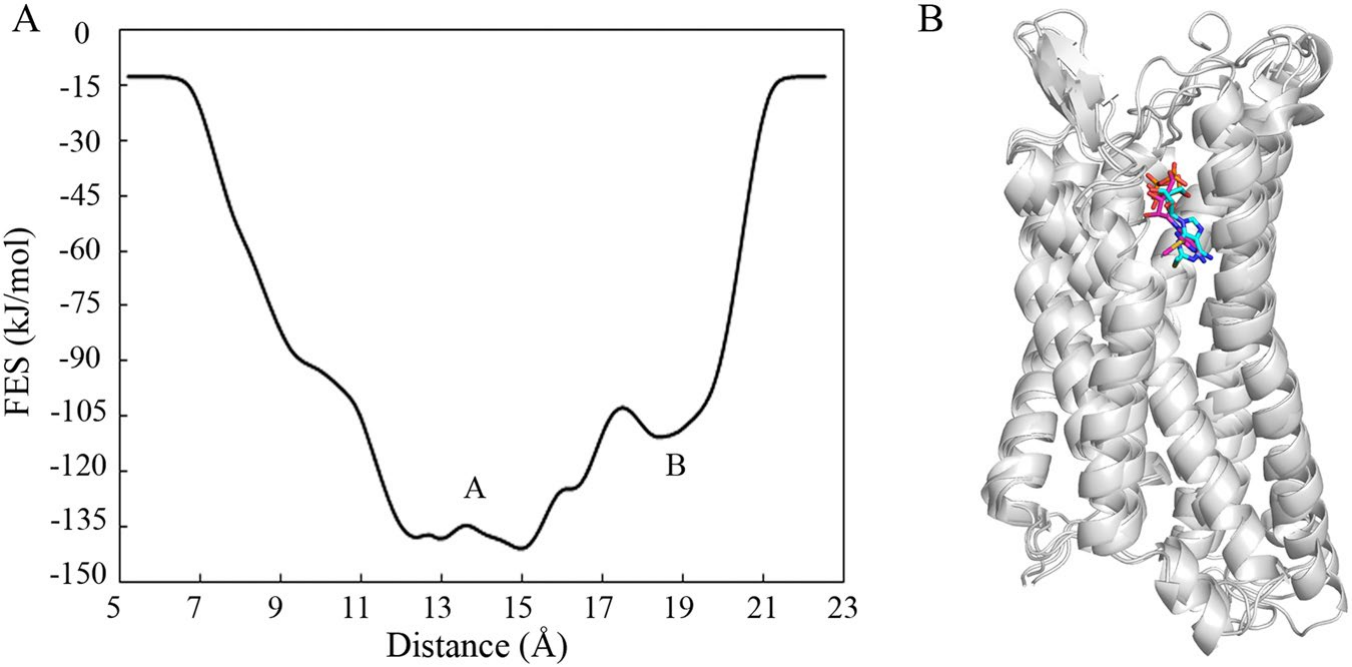
(**A**) Free energy surface associated with 2MeSADP-P2Y_1_R interactions, as a function of the distance between the COMs of 2MeSADP and the seven transmembrane helical bundles of P2Y_1_R in the *Z*-direction. (**B**) Comparison of the newly identified agonist-binding site and minimum A in Figure 3A. The P2Y_1_R structure is shown in cartoon and is colored in silver, 2MeSADP in the newly identified agonist-binding site and minimum A are shown in stick and colored in cyan and magenta, respectively.

In addition, we performed metadynamics simulation for the 2MeSADP-P2Y_1_R system and compared the results with those from our aMD simulations. The free energy surface associated with 2MeSADP-P2Y_1_R interactions along the distance between the COMs of 2MeSADP and the seven transmembrane helical bundles of P2Y_1_R in the direction perpendicular to membrane (*i.e*. the *Z*-direction) is depicted in Figure 3A. Figure 3A shows two minima (A and B). The distance between the COMs of 2MeSADP and P2Y_1_R in the *Z*-direction is around 12 to 15 Å and 18 to 19 Å in minima A and B, respectively. In the newly identified 2MeSADP binding site (Figure 2) and the initial site (Figure S1), this distance is 13.1 Å and 19.6 Å, respectively. Moreover, the binding pose in minimum A revealed using the metadynamics simulation aligns well with the newly identified 2MeSADP binding mode in the aMD simulation (Figure 3B). These confirm the reasonability of the newly identified 2MeSADP binding site and the reliability of the aMD method.

### Conformational states revealed by PMF analyses

The starting X-ray structure of P2Y_1_R was for the inactive state. For each system (*i.e*., 2MeSADP-P2Y_1_R, apo-P2Y_1_R and MRS2500-P2Y_1_R), we performed a 100-ns cMD simulation and a subsequent 300-ns aMD simulation. In the 100-ns cMD simulations, P2Y_1_R did not deviate substantially from the starting inactive structure. In the aMD simulations, P2Y_1_R shows increased conformational dynamics, especially in the 2MeSADP-P2Y_1_R system. Supplementary Figure S3 shows the RMSFs of the C_α_ atoms in P2Y_1_R, as calculated from the 300-ns aMD trajectories of the 2MeSADP-P2Y_1_R system, the apo-P2Y_1_R system and the MRS2500-P2Y_1_R systems. The RMSFs indicated that the ICLs and ECLs show higher conformational fluctuations than the helices. The most significant conformational dynamics in the 2MeSADP-P2Y_1_R system compared with the apo-P2Y_1_R system and MRS2500-P2Y_1_R systems was the fluctuation in the cytoplasmic end of helix VI (residues Leu254^6.29^ to Pro275^6.50^, labelled with a pink box in Supplementary Figure S3). This result was consistent with the conventional concept that movements of VI are absolutely essential for GPCR activation^2,39–41^.

**Figure 4:**
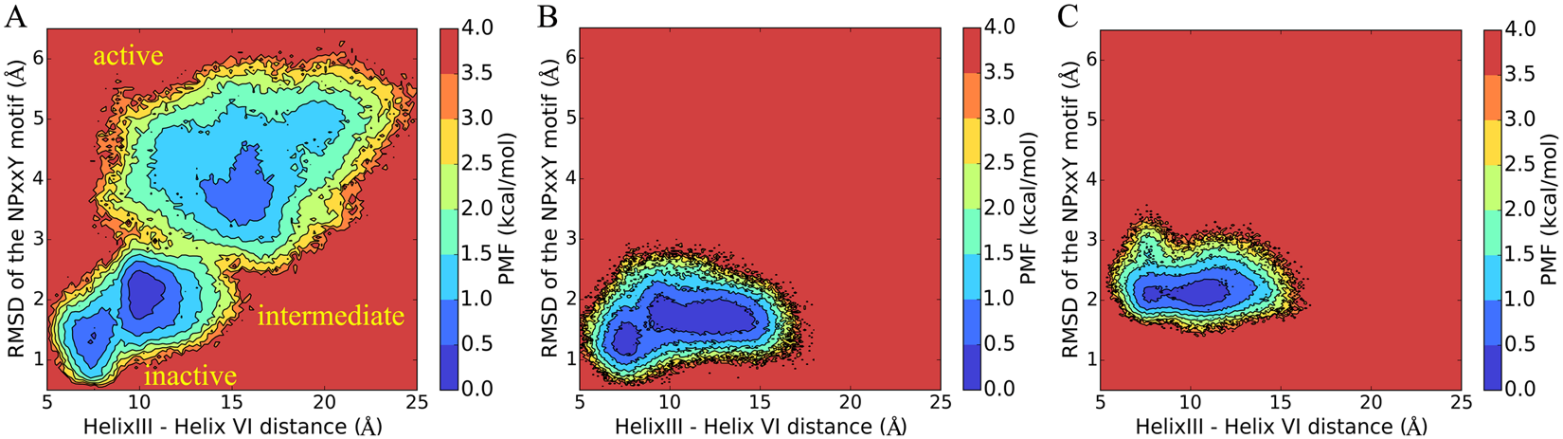
Potential of mean force (PMF) calculated for the helix III-helix VI distance and the RMSD of the NPxxY motif relative to the inactive starting structure for (**A**) the 2MeSADP-P2Y_1_R system, (**B**) the apo-P2Y_1_R system and (**C**) the MRS2500-P2Y_1_R system.

The movement of helix VI was monitored on the basis of the helix III-helix VI distance on the cytoplasmic side (C_α_-C_α_ distance of Val153^3.54^ and Leu254^6.29^). A significantly larger conformational space was sampled in the P2Y_1_R aMD simulations than in the cMD simulations. To gain insight into the effects of binding with 2MeSADP/MRS2500 on the overall conformational dynamics of P2Y_1_R, we performed PMF analyses on the basis of the 300-ns aMD trajectories, generating 2D energy landscape maps for the 2MeSADP-P2Y_1_R, apo-P2Y_1_R and MRS2500-P2Y_1_R systems. Figure 4 shows the PMF maps for these systems.

The PMF map reveals three different conformational states (*i.e*., the inactive, intermediate and active states) of P2Y_1_R in the 2MeSADP-P2Y_1_R system (Figure 4A). In contrast, in the apo-P2Y_1_R and the MRS2500-P2Y_1_R systems (Figure 4B and 4C), only two P2Y_1_R states (*i.e*., the inactive and intermediate states) are identified by using the 300-ns aMD trajectories.

To get solid and statistical conclusion, we performed additional 3 × 300 ns aMD simulations for each of the 2MeSADP-P2Y_1_R, apo-P2Y_1_R and MRS2500-P2Y_1_R systems. The PMF maps calculated from the 3 × 300 ns trajectories for each stystem show similar results that the 2MeSADP-P2Y_1_R system (Supplementary Figure S4) equilibrates in the inactive, intermediate and active states, whereas the apo-P2Y_1_R system (Supplementary Figure S5) and the MRS2500-P2Y_1_R system (Supplementary Figure S6) only stay in the inactive and intermediate states.

### Activation/inactivation mechanisms of P2Y_1_R after binding with 2MeSADP/MRS2500

**Figure 5:**
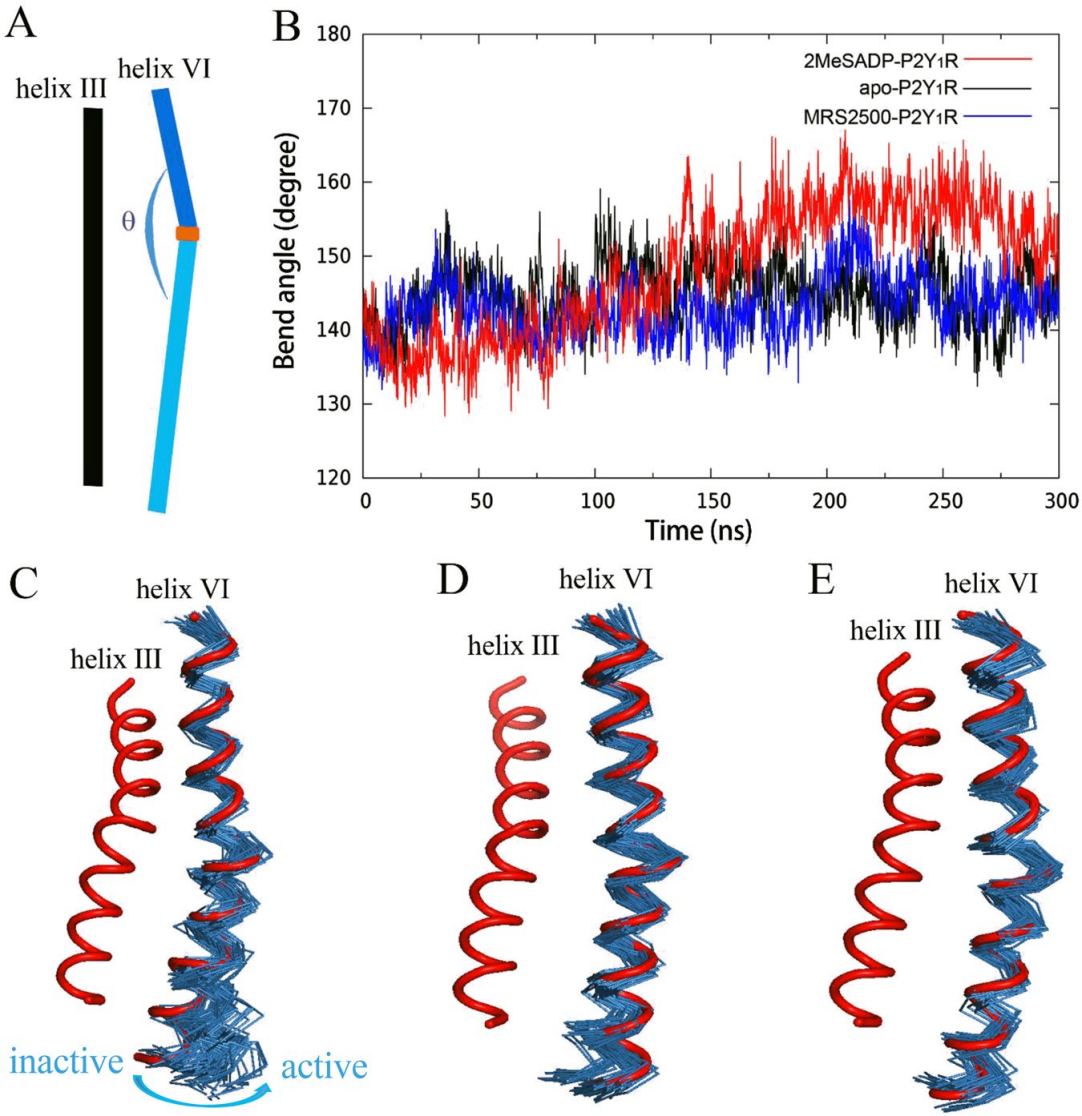
(**A**) Schematic representation of the bend angle (*θ*) of helix VI of P2Y_1_R. (**B**) Plots of *θ* in the 300-ns aMD trajectories of the 2MeSADP-P2Y_1_R, apo-P2Y_1_R and MRS2500-P2Y_1_R systems. Snapshots of helix VI extracted from the aMD trajectories of (**C**) the 2MeSADP-P2Y_1_R system, (**D**) the apo-P2Y_1_R system and (**E**) the MRS2500-P2Y_1_R system. The initial structures of helices VI and III are colored in red. The helix VI structures are superimposed on the initial structure with the C_α_ atoms of residues Leu254^6.29^ to Leu288^6.63^.

As shown in Figure 4, P2Y_1_R undergoes significant conformational changes after activation. To explore the conformational dynamics induced by the agonist 2MeSADP and the antagonist MRS2500 on P2Y_1_R, we monitored the dynamic movements of helix VI in the 2MeSADP-P2Y_1_R, apo-P2Y_1_R and MRS2500-P2Y_1_R systems. Figure 5 depicts the time dependence of the bend angle (*θ*) of helix VI and the representative snapshots extracted from the aMD trajectories of the three systems.

The bend angle of helix VI stretches after 2MeSADP binding (Figure 5B and 5C). The stretch of the helix VI bend angle causes helix VI to shift away from helix III. The bend angles of the inactive, intermediate and active state are approximately 137°, 142° and 156°, respectively. The corresponding helix III-helix VI distance of the inactive, intermediate and active state are approximately 7.6 Å, 10.6 Å and 16.7 Å (Supplementary Figure S7A). In comparison, no significant stretching of the bend angle of helix VI was observed in the apo-P2Y_1_R system during the aMD simulation (Figure 5B and 5D). Moreover, in the MRS2500-P2Y_1_R system, binding of the antagonist MRS2500 blocks the bend angle of helix VI from stretching and locks P2Y_1_R in its inactive state. These observations were consistent with a model in which 2MeSADP is a P2Y_1_R agonist and MRS2500 is a P2Y_1_R antagonist.

**Figure 6:**
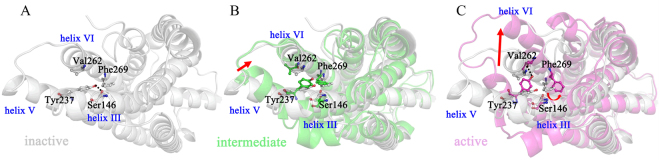
Representative structures of the cytoplasmic side of the (**A**) inactive (in silver), (**B**) intermediate (in green) and (**C**) active (in magenta) states of P2Y_1_R. P2Y_1_R is displayed in cartoon, and residues Ser146^3.47^, Tyr237^5.58^, Val262^6.37^ and Phe269^6.44^ are displayed in ball-and-stick.

The initial structure of the 2MeSADP-P2Y_1_R system was the inactive state. During the aMD simulation, P2Y_1_R passed through the intermediate state and reached an active state. The three different conformational states identified in Figure 4A correspond to the inactive, intermediate and active states of P2Y_1_R. To identify the microscopic structural characters discriminating the three states during the P2Y_1_R activation, the snapshots in the aMD trajectory of the 2MeSADP-P2Y_1_R system were grouped into three clusters: the inactive state (snapshots in 0–40 ns), the intermediate state (snapshots in 55–130 ns) and the active state (snapshots in 180–300 ns). Figure 6 shows the representative structures for each state. The following values were measured for the 2MeSADP-P2Y_1_R, apo-P2Y_1_R and MRS2500-P2Y_1_R systems in the 300-ns aMD simulations: the helix III-helix VI distance; the O–O distance between the hydroxyl of Ser146^3.47^ and the hydroxyl of Tyr237^5.58^; the O–O distance between the hydroxyl of Tyr237^5.58^ and the backbone oxygen of Val262^6.37^; and the χ1 rotamer (measured by the N-C_α_-C_β_-C_γ_ torsion angle) of Phe269^6.44^ (Supplementary Figure S7 to S9).

The initial condition of the 2MeSADP-P2Y_1_R system is the inactive state. In this state (Figure 6A), the helix III-helix VI distance is 7.6 Å (Supplementary Figure S7A), which is very close to the distance of 7.5 Å found in the crystal structure. The hydroxyl oxygen of Ser146^3.47^ and the hydroxyl oxygen of Tyr237^5.58^ form a hydrogen bond with an O–O distance of 3.0 Å (Supplementary Figure S7B). The χ1 rotamer of Phe269^6.44^ is approximately −82° (*i.e*., the *gauche-* state) and almost parallel to helix VI (Supplementary Figure S7D). P2Y_1_R then transitions to the intermediate state. In the intermediate state (Figure 6B), the helix III-helix VI distance increases to 10.6 Å (Supplementary Figure S7A). In addition, the cytoplasmic end of helix V exhibits high mobility, thus leading to disruption of the hydrogen bond between the two hydroxyl oxygen atoms of Ser146^3.47^ and Tyr237^5.58^ (Supplementary Figure S7B). The χ1 rotamer of Phe269^6.44^ remains in the *gauche-* state (Supplementary Figure S7D). After the intermediate stage, the helix III-helix VI distance sharply increases and approaches 16.7 Å (Supplementary Figure S7A). The cytoplasmic end of helix VI shifts outward by 9.1 Å relative to the inactive structure. This change drives P2Y_1_R to an active state similar to the active X-ray structures of β_2_AR and rhodopsin^42,43^. In addition, the largely opened G-protein-binding crevice allows for a G-protein to bind to the cytoplasmic surface of P2Y_1_R. In the active state, Tyr237^5.58^ reorients its side chain, thus allowing a hydrogen bond to form with the backbone oxygen of Val262^6.37^ (Supplementary Figure S7C). The χ1 rotamer of Phe269^6.44^ changes to ±180° (*i.e*., the *trans* state) and is almost perpendicular to helix VI (Supplementary Figure S7D).

In the apo-P2Y_1_R/MRS2500-P2Y_1_R system, the helix III-helix VI distance is 7.5/7.8 Å in the beginning of the aMD simulation (0 to 50 ns in Supplementary Figure S8A and 0 to 10 ns in Supplementary Figure S9A), which is very close to the distance in the inactive crystal structure. This distance increases to 11.3/10.3 Å and is maintained for the remaining trajectory. In the aMD simulation, the hydrogen bond between the hydroxyl oxygen of Ser146^3.47^ and the hydroxyl oxygen of Tyr237^5.58^ is closed, with an O–O distance of 3.4 Å (Supplementary Figures S8B and S9B). The hydroxyl oxygen of Tyr237^5.58^ and the backbone oxygen of Val262^6.37^ cannot form a hydrogen bond(Supplementary Figures S8C and S9C). The χ1 rotamer of Phe269^6.44^ is always in the *gauche-* state (Supplementary Figures S8D and S9D). These observations revealed that P2Y_1_R remained in the inactive state during the aMD simulations of the apo-P2Y_1_R and the MRS2500-P2Y_1_R systems.

Thus, the activation/inactivation mechanisms of P2Y_1_R after binding with 2MeSADP/MRS2500 can be summarized as follows. Binding of agonist 2MeSADP to P2Y_1_R leads to a stretching of the bend angle of helix VI by 19°. Consequently, the cytoplasmic end of helix VI shifts outward by 9.1 Å from helix III, thus activating P2Y_1_R for G-protein binding. Tyr237^5.58^ reorients, thereby breaking the hydrogen bond with Ser146^3.47^ and forms a new hydrogen bond with Val262^6.37^. The χ1 rotamer of Phe269^6.44^ changes from parallel to perpendicular to helix VI. In comparison, the binding of the antagonist MRS2500 blocks the bend angle of helix VI and locks P2Y_1_R in its inactive state.

## Conclusion

In the present study, we identified a new agonist-binding site and explored the activation mechanism of P2Y_1_R. The identified 2MeSADP binding site is much deeper than that in the crystal MRS2500-P2Y_1_R structure and the previous simulated ADP-P2Y_1_R structure but partially overlaps with the corresponding 2MeSADP binding site in the 2MeSADP-P2Y_12_R crystal structure. 2MeSADP interacts with His132^3.33^, Lys280^6.55^, Tyr136^3.37^, Thr221^5.42^, Thr222^5.43^, Arg128^3.29^, Arg287^6.62^, Arg310^7.39^, Tyr306^7.35^ through π-π stacking, π-cation interaction, hydrogen bonds and salt bridges. This binding mode is consistent with previous mutagenesis data. Binding of the agonist 2MeSADP to P2Y_1_R leads to stretching of the bend angle of helix VI and a significant outward shifting of the helix VI cytoplasmic end. The activation of P2Y_1_R is also characterized by the breaking of the Ser146^3.47^-Tyr237^5.58^ hydrogen bond, the formation of the Tyr237^5.58^-Val262^6.37^ hydrogen bond and a χ1 rotamer change of Phe269^6.44^ (from parallel to perpendicular to helix VI). In contrast, binding of the antagonist MRS2500 locks P2Y_1_R in its inactive state. The newly identified agonist-binding site and the activation mechanism P2Y_1_R revealed in this work should provide assistance in the design of potent P2Y_1_R antagonists and agonists, which might by used as antithrombotic and antidiabetic drugs.

## Methods

### System preparation

Three simulation systems were set up, including 2MeSADP-P2Y_1_R, apo-P2Y_1_R and MRS2500-P2Y_1_R. The MRS2500-P2Y_1_R structure was extracted from Protein Data Bank crystal structures (PDB ID: 4XNW)^17^. T4 lysozyme and unnecessary small molecules were removed from this crystal structure. The missing residues were constructed by homology modelling using the Modeller module of CHIMERA^44^. The protonation state for titratable residues at neutral pH were determined using H++ ^45^. The apo-P2Y_1_R structure was prepared by removing MRS2500 from the MRS2500-P2Y_1_R complex. The 2MeSADP-P2Y_1_R structure was constructed by consulting the 2MeSADP-P2Y_12_R crystal structure^16^. In addition to the MRS2500-binding site, there was a deeper cavity in the MRS2500-P2Y_1_R structure that partially overlapped with 2MeSADP in the 2MeSADP-P2Y_12_R structure. However, this cavity was too small to accommodate 2MeSADP. The ECL2 domain extended deep into the ligand-binding pocket and blocked the access of 2MeSADP to the deep cavity. Thus, we removed Asp204, Thr205, Thr206 and Ser207 of ECL2 to open the access to this deep cavity. To enlarge the deep cavity to accommodate 2MeSADP, we also mutated Thr203^ECL2^ and Ile186^4.51^ to alanines. Then, 2MeSAD was docked into this deep cavity by using the Schrödinger Induced Fit Docking protocol^46^. Then, the Asp204, Thr205, Thr206 and Ser207 of ECL2 residues were added back by homology modelling using the Modeller module of CHIMERA,^44^ and residues Ala203^ECL2^ and Ala186^4.51^ were mutated back to threonine and isoleucine.

The CHARMM-GUI Membrane builder^47^ was used to construct the membrane-lipid systems. The transmembrane helical bundle of P2Y_1_R was oriented along the *Z*-axis of the POPC bilayer and the overlapping lipid molecules were removed. Then, the P2Y_1_R-bilayer complexes were neutralized at 0.15 M KCl and were solvated in TIP3P^48^ water boxes. The final simulation systems of 2MeSADP-P2Y_1_R, apo-P2Y_1_R and MRS2500-P2Y_1_R consisted 67498, 64172 and 67132 atoms.

### Molecular dynamics simulations

The cMD and aMD simulations in the present study were performed using the PMEMD module of AMBER 14^49^. The AMBER FF99SB force field^50^ was used for P2Y_1_R, the general AMBER force field (GAFF)^51^ was used for 2MeSADP and MRS2500, and the amber lipid force field LIPID14^52^ was used for POPC. A series of minimizations were carried out for each system (*i.e*., 2MeSADP-P2Y_1_R, apo-P2Y_1_R and MRS2500-P2Y_1_R). First, the waters were minimized for 10000 steps, and the P2Y_1_R, ligand and POPCs were constrained with 500 kcal·mol^−1^·Å^−2^. Second, the waters and the POPCs were minimized for 20000 steps, and P2Y_1_R and the ligand were constrained with 500 kcal·mol^−1^·Å^−2^. Third, the whole system was released and minimized for 10000 steps. Then, each system was heated from 0 K to 310 K in 700 ps with a Langevin^53^ thermostat, and the P2Y_1_R, ligand and POPCs were constrained with 10 kcal**·**mol^−1^ **·**Å^−2^. Then, each system was equilibrated for 200 ps with 10 kcal**·**mol^−1^ **·**Å^−2^ on P2Y_1_R, the ligand and the POPCs and for 5 ns with no constrains. After equilibration, the 100-ns cMD simulation was carried out in a constant pressure (NPT) ensemble for each system. Long-range electrostatic interactions was treated by using the particle mesh Ewald (PME)^54^ algorithm. All of the covalent bonds involving hydrogen atoms were constrained by using the SHAKE^55^ algorithm. To capture more obvious conformational changes involved in P2Y_1_R activation, three independent 300-ns aMD simulations were performed on the 2MeSADP-P2Y_1_R, apo-P2Y_1_R and MRS2500-P2Y_1_R systems by restarting from the last snapshot of the 100-ns cMD simulations.

### Metadynamics simulation

The well-tempered metadynamics^56,57^ was performed using the AMBER 14 program^49^ and PLUMED 2.4a plugin^58^ after the 100 ns cMD simulation of the 2MeSADP-P2Y_1_R system. We used two collective variables, CV_1_ and CV_2_, to investigate the binding of 2MeSADP to P2Y_1_R. CV_1_ was the distance between the centers of mass (COM) of 2MeSADP and the seven transmembrane helical bundles of P2Y_1_R in the direction perpendicular to membrane (Z-direction). CV_2_ was the distance between the cytoplasmic ends of helix III and helix VI (represented by the C_α_-C_α_ distance between Val153^3.54^ and Leu254^6.29^). The metadynamics was activated in CV_1_ and CV_2_ by depositing a Gaussian bias term every picosecond with height of 1 kJ**·**mol^−1^ and width of 0.05 nm. The bias factor was 15, and the temperature was 310 K. To keep the 2MeSADP in contact with P2Y_1_R, we had enforced the XY component of the distance between COM of 2MeSADP and COM of the seven transmembrane helical bundles of P2Y_1_R below 3 nm.

### Potential of mean force

We used potential of mean force (PMF)^59^ analyses and generated 2D energy landscapes to characterize the conformational changes of P2Y_1_R for each of the three simulated systems. The reaction coordinates of PMF map were the helix III-helix VI distance on the cytoplasmic side and the RMSD of the NPxxY motif. The energy landscape was calculated as^59^:

1 $${\rm{\Delta }}G(x,y)=-{k}_{B}T\,\mathrm{ln}\,g(x,y)$$

in which *k*_B_ is the Boltzmann constant, *T* is the temperature, and *g*(*x*, *y*) is the normalized joint probability distribution.

## Electronic supplementary material

Supplementary Information

pdb file of the inactive state

pdb file of the intermediate state

pdb file of the active state
