# Supplementary material for: Identification of a Different Agonist-Binding Site and Activation Mechanism of the Human P2Y1 Receptor
Source: Sci Rep. 2017 Oct 23;7:13764. doi: 10.1038/s41598-017-14268-1 (PMC5653743; doi:10.1038/s41598-017-14268-1)
Supplement: Supplementary file 1 — Supplementary Information [file 41598_2017_14268_MOESM1_ESM.pdf]

Supplementary Information

Identification of a Different Agonist-Binding Site and Activation Mechanism of the Human $P2Y_1$ Receptor

Yang Li, Can Yin, Pi Liu, Dongmei Li and Jianping Lin**

Contents:

- **Figure S1.** (A) The binding mode of 2MeSADP in the MRS2500 binding site of $P2Y_1R$. 2MeSADP and MRS2500 are colored in cyan and yellow respectively. (B) Schematic representation of interactions between 2MeSADP and $P2Y_1R$ in the MRS2500 binding site.
- **Figure S2.** Interactions between 2MeSADP and $P2Y_1R$ along the simulation. (A) The π-π stacking between the adenine ring of 2MeSADP and the imidazole group of His132$^{3.33}$; (B) the π-cation stacking between the adenine ring of 2MeSADP and the ε-amino group of Lys280$^{6.55}$; (C) the hydrogen bond between the amino group in adenine of 2MeSADP and the hydroxyl group of Thr222$^{5.43}$; (D) the hydrogen bond between the N^1 in adenine of 2MeSADP and the phenolic hydroxyl group of Tyr136$^{3.37}$; (E) the hydrogen bond between the N^1 in adenine of 2MeSADP and and the hydroxyl group of Thr221$^{5.42}$; the electrostatic interactions between the negatively charged pyrophosphates of 2MeSADP and the positively charged amidine group of (F) Arg128$^{3.29}$, (G) Arg287$^{6.62}$ (H) Arg310$^{7.39}$; (I) the electrostatic interactions between the negatively charged pyrophosphates of 2MeSADP and the positively charged ε-amino group of Lys280$^{6.55}$ and (J) the hydrogen bond between the O^{1B} in the pyrophosphate of 2MeSADP and the phenolic hydroxyl group of Tyr306$^{7.35}$.
- **Figure S3.** Plots of RMSF of C_α atoms in $P2Y_1R$ calculated from the 300 ns aMD trajectories of the 2MeSADP-$P2Y_1R$ system, the apo-$P2Y_1R$ system and the MRS2500-$P2Y_1R$ system.
- **Figure S4.** Potential of mean force (PMF) calculated for the helix III-helix VI distance and the RMSD of the NPxxY motif relative to the inactive starting structure for the 2MeSADP-$P2Y_1R$ system.

- **Figure S5.** Potential of mean force (PMF) calculated for the helix III-helix VI distance and the RMSD of the NPxxY motif relative to the inactive starting structure for the apo-$P2Y_1R$ system.
- **Figure S6.** Potential of mean force (PMF) calculated for the helix III-helix VI distance and the RMSD of the NPxxY motif relative to the inactive starting structure for the MRS2500-$P2Y_1R$ system.
- **Figure S7.** Plots of (A) the helix III-helix VI distance, (B) the O--O distance between the hydroxyl of $Ser146^{3.47}$ and the hydroxyl of $Tyr237^{5.58}$, (C) the O--O distance between the hydroxyl of $Tyr237^{5.58}$ and the backbone oxygen of $Val262^{6.37}$, (D) the χ1 rotamer of $Phe269^{6.44}$ in the 300 ns aMD simulations of the 2MeSADP-$P2Y_1R$ system.
- **Figure S8.** Plots of (A) the helix III-helix VI distance, (B) the O--O distance between the hydroxyl of $Ser146^{3.47}$ and the hydroxyl of $Tyr237^{5.58}$, (C) the O--O distance between the hydroxyl of $Tyr237^{5.58}$ and the backbone oxygen of $Val262^{6.37}$, (D) the χ1 rotamer of $Phe269^{6.44}$ in the 300 ns aMD simulations of the apo-$P2Y_1R$ system.
- **Figure S9.** Plots of (A) the helix III-helix VI distance, (B) the O--O distance between the hydroxyl of $Ser146^{3.47}$ and the hydroxyl of $Tyr237^{5.58}$, (C) the O--O distance between the hydroxyl of $Tyr237^{5.58}$ and the backbone oxygen of $Val262^{6.37}$, (D) the χ1 rotamer of $Phe269^{6.44}$ in the 300 ns aMD simulations of the MRS2500-$P2Y_1R$ system.
- Coordinates of the inactive, intermediate and active states of the 2MeSADP-$P2Y_{12}R$ system (pdb files).

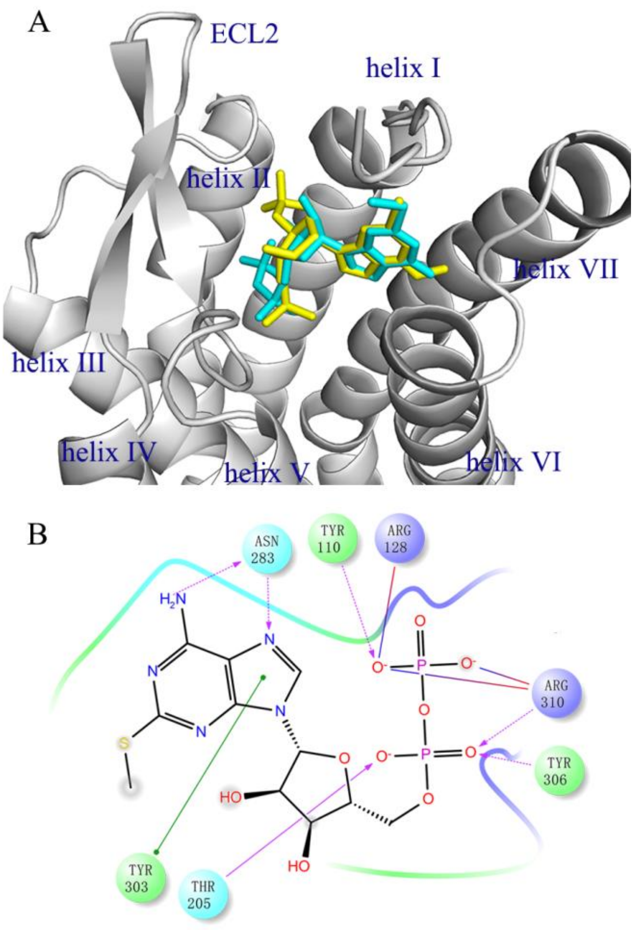

Figure S1. (A) The binding mode of 2MeSADP in the MRS2500 binding site of $P2Y_1R$. 2MeSADP and MRS2500 are colored in cyan and yellow respectively. (B) Schematic representation of interactions between 2MeSADP and $P2Y_1R$ in the MRS2500 binding site.

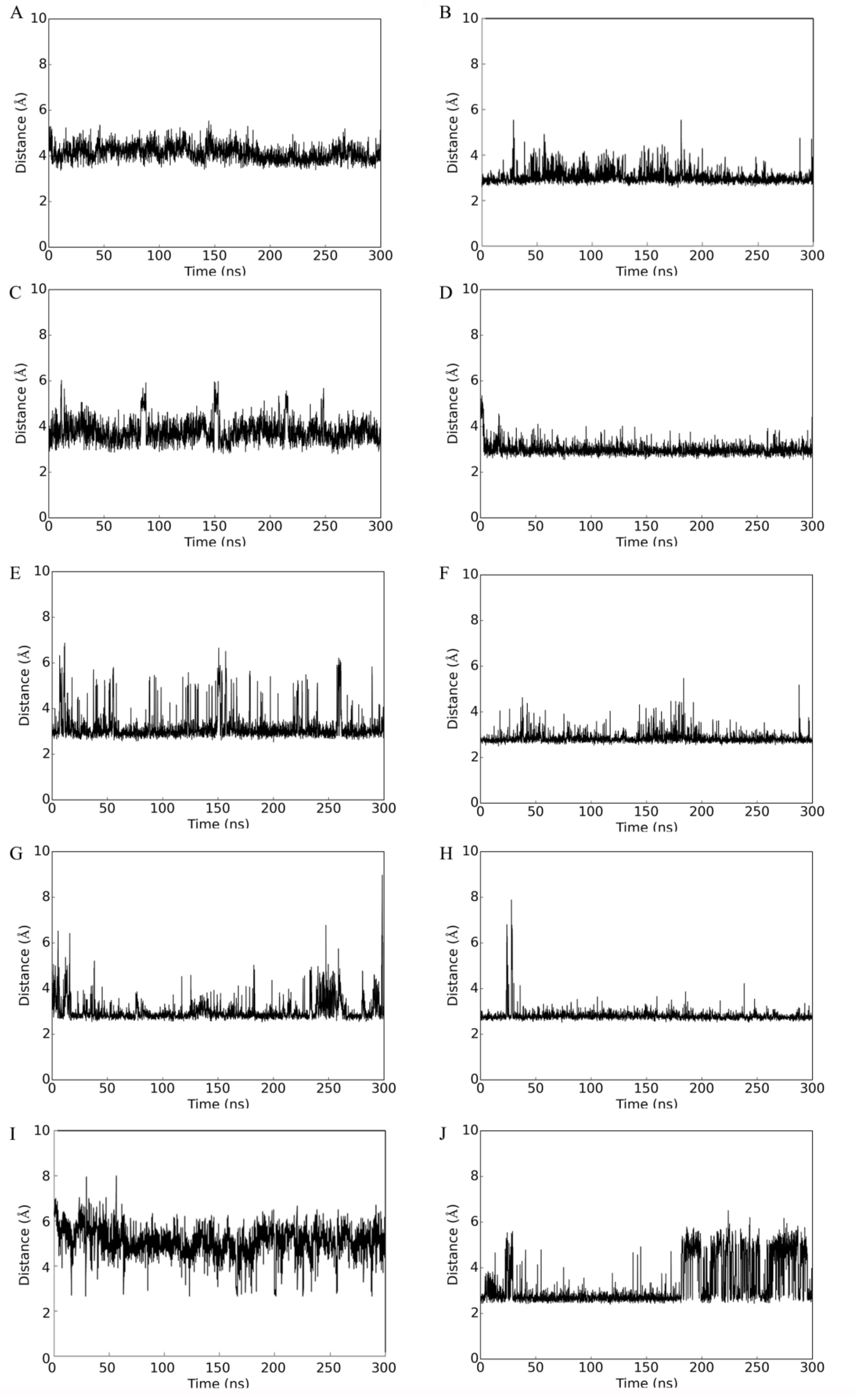
A
B
C
D
E
F
G
H
I
J
Distance (Å)
Time (ns)

Figure S2. Interactions between 2MeSADP and P2Y1R along the simulation time. (A) The π-π stacking between the adenine ring of 2MeSADP and the imidazole group of His132$^{3.33}$; (B) the π-cation stacking between the adenine ring of 2MeSADP and the ε-amino group of Lys280$^{6.55}$; (C) the hydrogen bond between the amino group in adenine of 2MeSADP and the hydroxyl group of Thr222$^{5.43}$; (D) the hydrogen bond between the N^1 in adenine of 2MeSADP and the phenolic hydroxyl group of Tyr136$^{3.37}$; (E) the hydrogen bond between the N^1 in adenine of 2MeSADP and and the hydroxyl group of Thr221$^{5.42}$; the electrostatic interactions between the negatively charged pyrophosphates of 2MeSADP and the positively charged amidine group of (F) Arg128$^{3.29}$, (G) Arg287$^{6.62}$ (H) Arg310$^{7.39}$; (I) the electrostatic interactions between the negatively charged pyrophosphates of 2MeSADP and the positively charged ε-amino group of Lys280$^{6.55}$ and (J) the hydrogen bond between the O^{1B} in the pyrophosphate of 2MeSADP and the phenolic hydroxyl group of Tyr306$^{7.35}$.

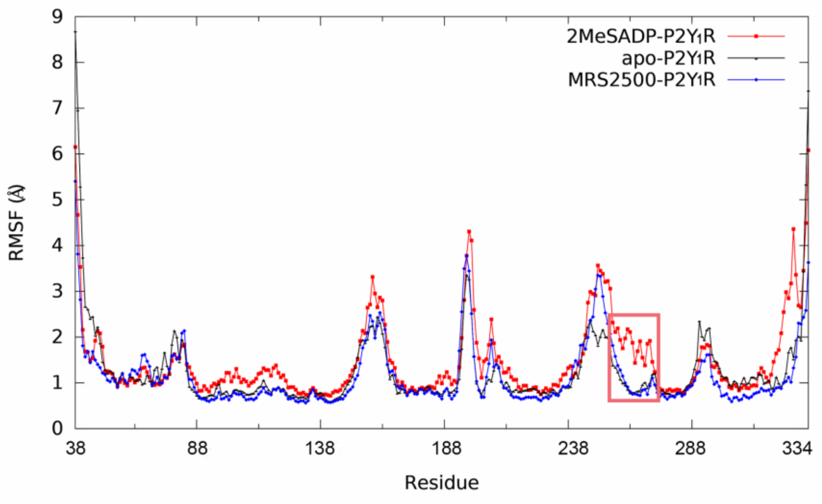

Figure S3. Plots of RMSF of C_α atoms in $P2Y_1R$ calculated from the 300 ns aMD trajectories of the 2MeSADP-$P2Y_1R$ system, the apo-$P2Y_1R$ system and the MRS2500-$P2Y_1R$ system.

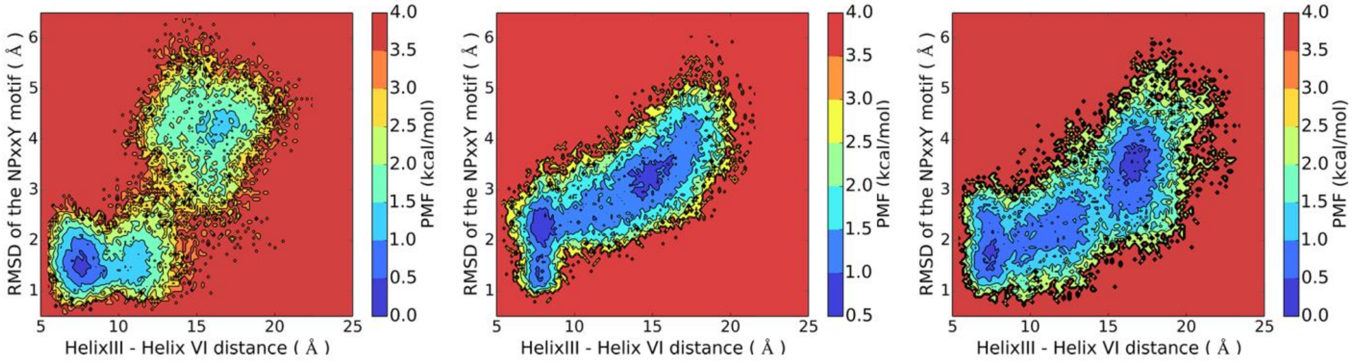

Figure S4. Potential of mean force (PMF) calculated for the helix III-helix VI distance and the RMSD of the NPxxY motif relative to the inactive starting structure for the 2MeSADP-P2Y_1R system.

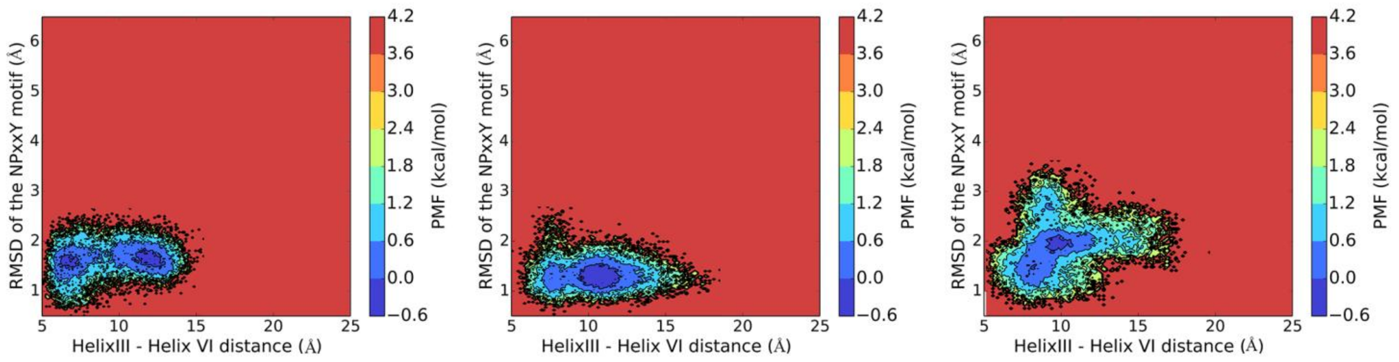

Figure S5. Potential of mean force (PMF) calculated for the helix III-helix VI distance and the RMSD of the NPxxY motif relative to the inactive starting structure for the apo-P2Y_1R system.

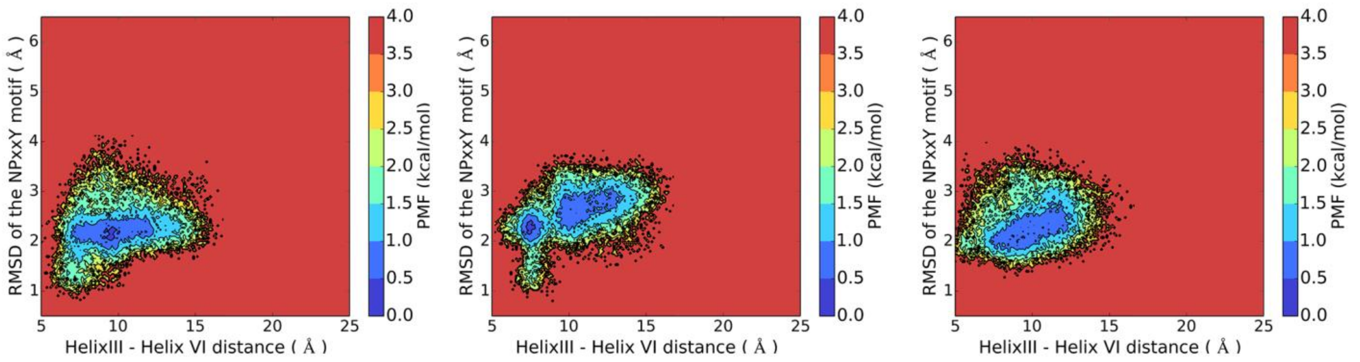

Figure S6. Potential of mean force (PMF) calculated for the helix III-helix VI distance and the RMSD of the NPxxY motif relative to the inactive starting structure for the MRS2500-P2Y_1R system.

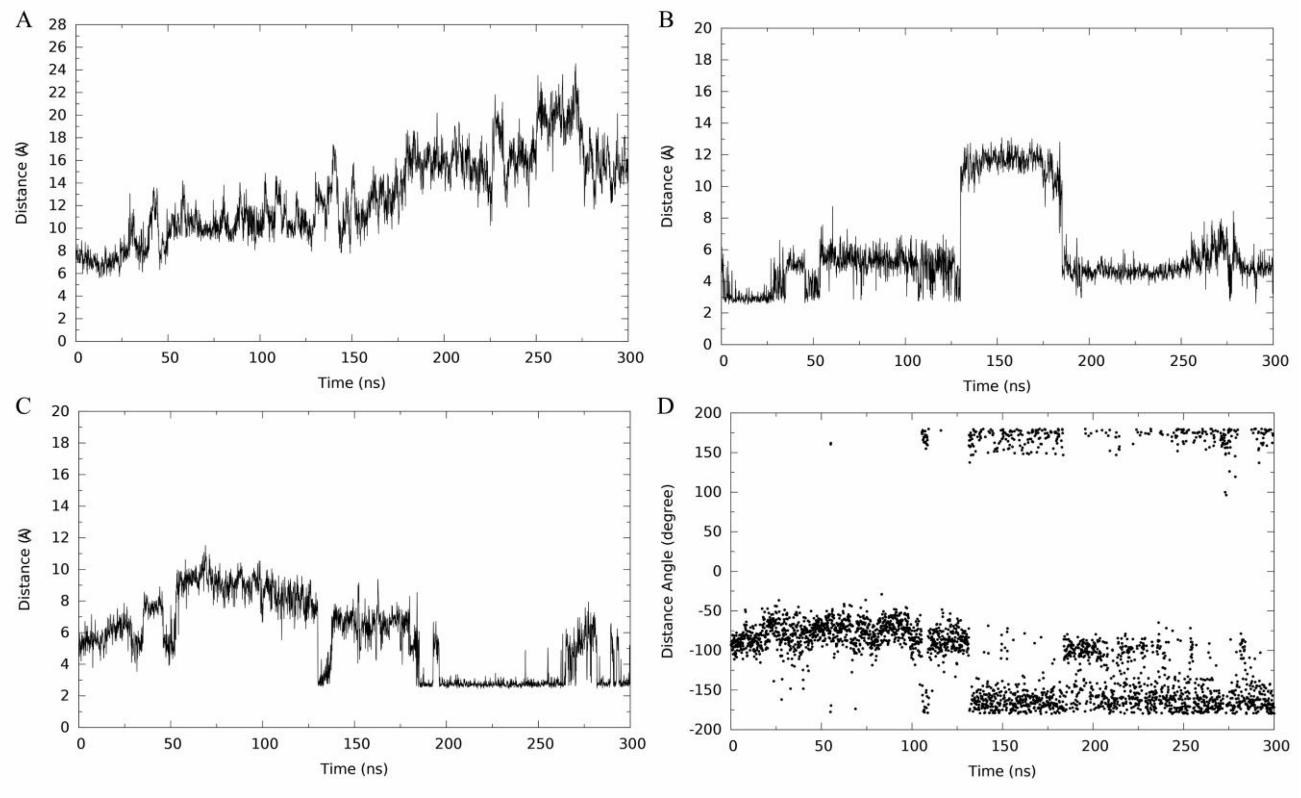

Figure S7. Plots of (A) the helix III-helix VI distance, (B) the O--O distance between the hydroxyl of Ser146$^{3.47}$ and the hydroxyl of Tyr237$^{5.58}$, (C) the O--O distance between the hydroxyl of Tyr237$^{5.58}$ and the backbone oxygen of Val262$^{6.37}$, (D) the $\chi 1$ rotamer of Phe269$^{6.44}$ in the 300 ns aMD simulations of the 2MeSADP-P2Y$_1$R system.

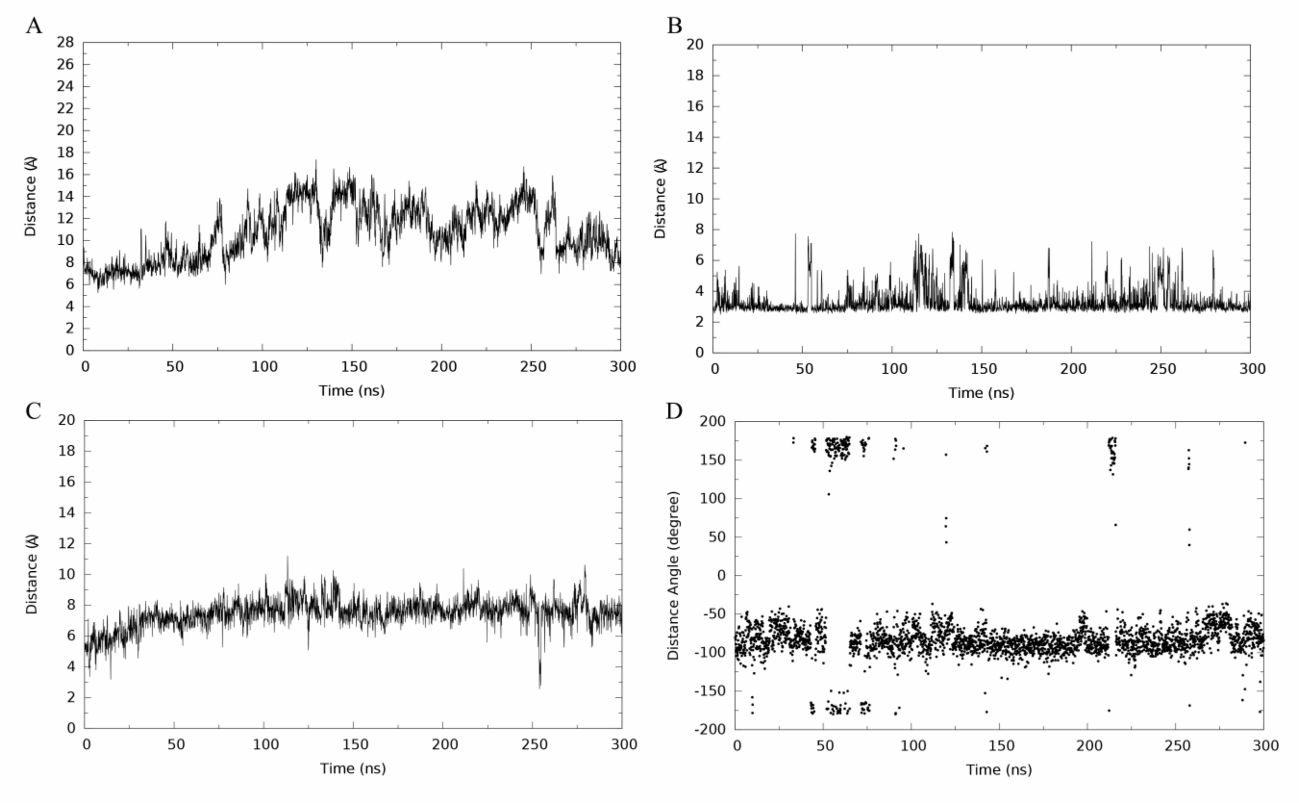

Figure S8. Plots of (A) the helix III-helix VI distance, (B) the O--O distance between the hydroxyl of Ser146$^{3.47}$ and the hydroxyl of Tyr237$^{5.58}$, (C) the O--O distance between the hydroxyl of Tyr237$^{5.58}$ and the backbone oxygen of Val262$^{6.37}$, (D) the $\chi 1$ rotamer of Phe269$^{6.44}$ in the 300 ns aMD simulations of the apo-P2Y$_1$R system.

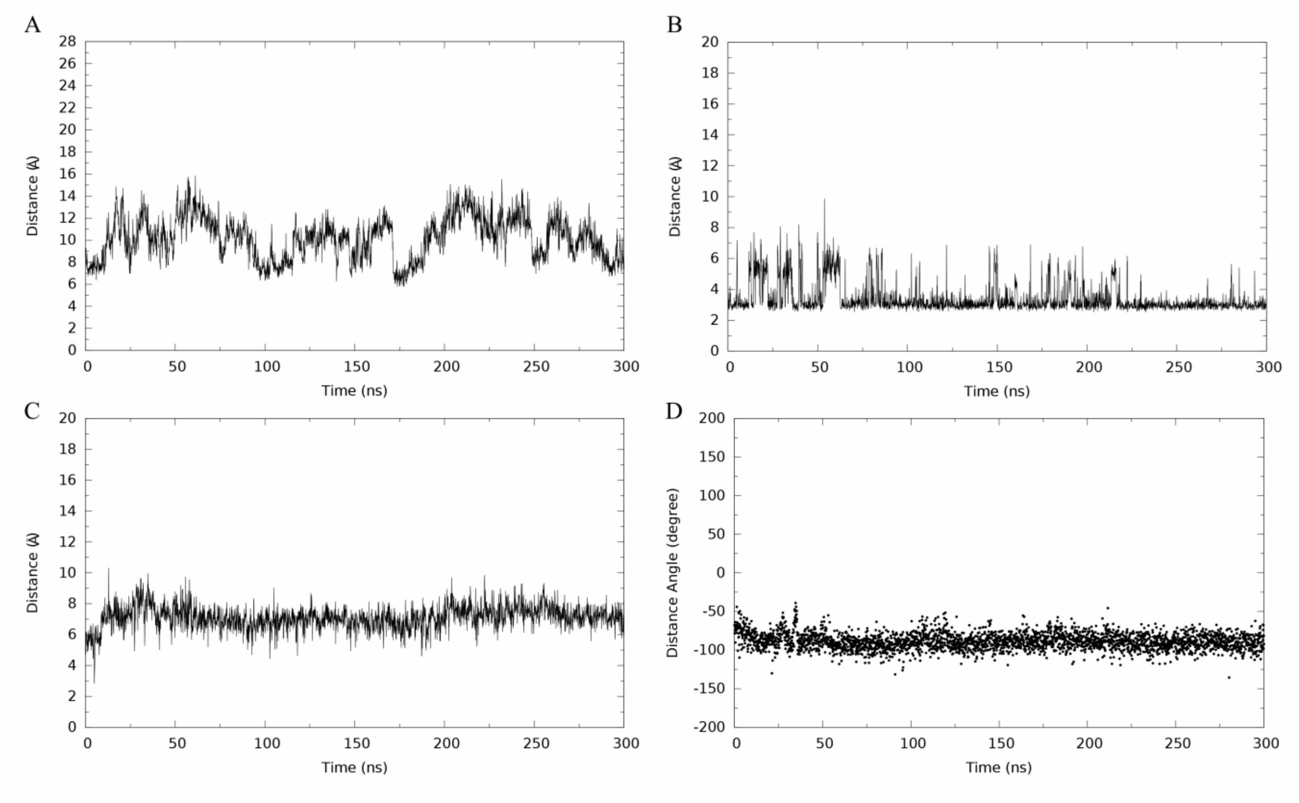

Figure S9. Plots of (A) the helix III-helix VI distance, (B) the O--O distance between the hydroxyl of Ser146$^{3.47}$ and the hydroxyl of Tyr237$^{5.58}$, (C) the O--O distance between the hydroxyl of Tyr237$^{5.58}$ and the backbone oxygen of Val262$^{6.37}$, (D) the $\chi1$ rotamer of Phe269$^{6.44}$ in the 300 ns aMD simulations of the MRS2500-P2Y$_1$R system.
