# Supplementary material for: Identification of a Different Agonist-Binding Site and Activation Mechanism of the Human P2Y1 Receptor
Source: Sci Rep. 2017 Oct 23;7:13764. doi: 10.1038/s41598-017-14268-1 (PMC5653743; doi:10.1038/s41598-017-14268-1)
Supplement: Supplementary file 2 — pdb file of the inactive state [file 41598_2017_14268_MOESM2_ESM.doc]

# Supplementary Information

Identification of a Different Agonist-Binding Site and Activation Mechanism of the Human P2Y_1_ Receptor

Yang Li, Can Yin, Pi Liu, Dongmei Li* and Jianping Lin*

- Coordinates of the inactive state of the 2MeSADP-P2Y_12_R system (pdb file).

INACTIVE STATE

ATOM 1 N SER 1 50.583 60.659 20.708 1.00 0.00 N

ATOM 2 H1 SER 1 50.666 60.919 21.680 1.00 0.00 H

ATOM 3 H2 SER 1 50.868 59.690 20.674 1.00 0.00 H

ATOM 4 H3 SER 1 49.591 60.724 20.526 1.00 0.00 H

ATOM 5 CA SER 1 51.351 61.499 19.771 1.00 0.00 C

ATOM 6 HA SER 1 52.360 61.662 20.150 1.00 0.00 H

ATOM 7 CB SER 1 51.383 60.838 18.428 1.00 0.00 C

ATOM 8 HB2 SER 1 50.464 61.127 17.918 1.00 0.00 H

ATOM 9 HB3 SER 1 52.208 61.290 17.877 1.00 0.00 H

ATOM 10 OG SER 1 51.499 59.451 18.548 1.00 0.00 O

ATOM 11 HG SER 1 51.488 59.035 17.683 1.00 0.00 H

ATOM 12 C SER 1 50.642 62.846 19.723 1.00 0.00 C

ATOM 13 O SER 1 49.426 63.006 19.808 1.00 0.00 O

ATOM 14 N SER 2 51.435 63.908 19.503 1.00 0.00 N

ATOM 15 H SER 2 52.409 63.759 19.726 1.00 0.00 H

ATOM 16 CA SER 2 50.979 65.254 19.128 1.00 0.00 C

ATOM 17 HA SER 2 49.977 65.053 18.747 1.00 0.00 H

ATOM 18 CB SER 2 50.855 65.980 20.502 1.00 0.00 C

ATOM 19 HB2 SER 2 50.102 66.767 20.463 1.00 0.00 H

ATOM 20 HB3 SER 2 50.545 65.288 21.285 1.00 0.00 H

ATOM 21 OG SER 2 52.097 66.481 20.998 1.00 0.00 O

ATOM 22 HG SER 2 51.838 66.879 21.833 1.00 0.00 H

ATOM 23 C SER 2 51.936 65.879 18.109 1.00 0.00 C

ATOM 24 O SER 2 53.076 65.480 17.853 1.00 0.00 O

ATOM 25 N PHE 3 51.407 66.930 17.456 1.00 0.00 N

ATOM 26 H PHE 3 50.467 67.181 17.727 1.00 0.00 H

ATOM 27 CA PHE 3 52.321 67.813 16.652 1.00 0.00 C

ATOM 28 HA PHE 3 52.690 67.245 15.797 1.00 0.00 H

ATOM 29 CB PHE 3 51.492 69.020 16.068 1.00 0.00 C

ATOM 30 HB2 PHE 3 50.686 68.714 15.401 1.00 0.00 H

ATOM 31 HB3 PHE 3 50.919 69.573 16.813 1.00 0.00 H

ATOM 32 CG PHE 3 52.331 70.028 15.331 1.00 0.00 C

ATOM 33 CD1 PHE 3 52.257 71.425 15.550 1.00 0.00 C

ATOM 34 HD1 PHE 3 51.565 71.753 16.312 1.00 0.00 H

ATOM 35 CE1 PHE 3 53.051 72.275 14.788 1.00 0.00 C

ATOM 36 HE1 PHE 3 53.135 73.341 14.942 1.00 0.00 H

ATOM 37 CZ PHE 3 53.861 71.793 13.708 1.00 0.00 C

ATOM 38 HZ PHE 3 54.501 72.468 13.161 1.00 0.00 H

ATOM 39 CE2 PHE 3 53.839 70.426 13.381 1.00 0.00 C

ATOM 40 HE2 PHE 3 54.410 70.026 12.555 1.00 0.00 H

ATOM 41 CD2 PHE 3 52.993 69.607 14.186 1.00 0.00 C

ATOM 42 HD2 PHE 3 52.990 68.548 13.975 1.00 0.00 H

ATOM 43 C PHE 3 53.525 68.429 17.441 1.00 0.00 C

ATOM 44 O PHE 3 53.410 69.245 18.372 1.00 0.00 O

ATOM 45 N LYS 4 54.826 68.240 16.930 1.00 0.00 N

ATOM 46 H LYS 4 54.913 67.486 16.263 1.00 0.00 H

ATOM 47 CA LYS 4 56.001 68.555 17.706 1.00 0.00 C

ATOM 48 HA LYS 4 55.779 68.365 18.756 1.00 0.00 H

ATOM 49 CB LYS 4 57.163 67.589 17.490 1.00 0.00 C

ATOM 50 HB2 LYS 4 57.352 67.523 16.419 1.00 0.00 H

ATOM 51 HB3 LYS 4 58.062 67.860 18.044 1.00 0.00 H

ATOM 52 CG LYS 4 56.809 66.205 18.063 1.00 0.00 C

ATOM 53 HG2 LYS 4 57.105 66.341 19.103 1.00 0.00 H

ATOM 54 HG3 LYS 4 55.756 65.922 18.058 1.00 0.00 H

ATOM 55 CD LYS 4 57.608 65.090 17.412 1.00 0.00 C

ATOM 56 HD2 LYS 4 57.066 64.514 16.662 1.00 0.00 H

ATOM 57 HD3 LYS 4 58.499 65.525 16.960 1.00 0.00 H

ATOM 58 CE LYS 4 58.063 64.123 18.495 1.00 0.00 C

ATOM 59 HE2 LYS 4 58.526 64.509 19.402 1.00 0.00 H

ATOM 60 HE3 LYS 4 57.133 63.674 18.846 1.00 0.00 H

ATOM 61 NZ LYS 4 58.967 63.137 17.873 1.00 0.00 N

ATOM 62 HZ1 LYS 4 58.919 63.055 16.868 1.00 0.00 H

ATOM 63 HZ2 LYS 4 59.925 63.338 18.122 1.00 0.00 H

ATOM 64 HZ3 LYS 4 58.638 62.275 18.285 1.00 0.00 H

ATOM 65 C LYS 4 56.471 70.013 17.691 1.00 0.00 C

ATOM 66 O LYS 4 57.614 70.300 17.327 1.00 0.00 O

ATOM 67 N CYX 5 55.533 70.945 17.898 1.00 0.00 N

ATOM 68 H CYX 5 54.606 70.613 18.124 1.00 0.00 H

ATOM 69 CA CYX 5 55.834 72.432 17.877 1.00 0.00 C

ATOM 70 HA CYX 5 56.163 72.674 16.866 1.00 0.00 H

ATOM 71 CB CYX 5 54.459 73.004 18.233 1.00 0.00 C

ATOM 72 HB2 CYX 5 53.618 72.368 17.957 1.00 0.00 H

ATOM 73 HB3 CYX 5 54.337 73.214 19.296 1.00 0.00 H

ATOM 74 SG CYX 5 54.181 74.648 17.455 1.00 0.00 S

ATOM 75 C CYX 5 56.854 72.995 18.900 1.00 0.00 C

ATOM 76 O CYX 5 56.933 72.492 20.029 1.00 0.00 O

ATOM 77 N ALA 6 57.733 73.920 18.560 1.00 0.00 N

ATOM 78 H ALA 6 57.474 74.258 17.644 1.00 0.00 H

ATOM 79 CA ALA 6 58.897 74.450 19.267 1.00 0.00 C

ATOM 80 HA ALA 6 59.643 73.704 19.542 1.00 0.00 H

ATOM 81 CB ALA 6 59.550 75.466 18.349 1.00 0.00 C

ATOM 82 HB1 ALA 6 59.060 76.439 18.317 1.00 0.00 H

ATOM 83 HB2 ALA 6 60.578 75.534 18.706 1.00 0.00 H

ATOM 84 HB3 ALA 6 59.608 75.077 17.333 1.00 0.00 H

ATOM 85 C ALA 6 58.476 75.319 20.509 1.00 0.00 C

ATOM 86 O ALA 6 57.365 75.801 20.502 1.00 0.00 O

ATOM 87 N LEU 7 59.301 75.400 21.561 1.00 0.00 N

ATOM 88 H LEU 7 60.094 74.775 21.572 1.00 0.00 H

ATOM 89 CA LEU 7 58.904 75.989 22.845 1.00 0.00 C

ATOM 90 HA LEU 7 58.025 76.614 22.692 1.00 0.00 H

ATOM 91 CB LEU 7 58.518 74.853 23.797 1.00 0.00 C

ATOM 92 HB2 LEU 7 59.390 74.205 23.714 1.00 0.00 H

ATOM 93 HB3 LEU 7 58.364 75.160 24.832 1.00 0.00 H

ATOM 94 CG LEU 7 57.141 74.145 23.441 1.00 0.00 C

ATOM 95 HG LEU 7 57.281 73.683 22.463 1.00 0.00 H

ATOM 96 CD1 LEU 7 56.914 72.928 24.277 1.00 0.00 C

ATOM 97 HD11 LEU 7 57.554 72.115 23.934 1.00 0.00 H

ATOM 98 HD12 LEU 7 57.303 73.195 25.259 1.00 0.00 H

ATOM 99 HD13 LEU 7 55.866 72.645 24.373 1.00 0.00 H

ATOM 100 CD2 LEU 7 55.888 74.995 23.500 1.00 0.00 C

ATOM 101 HD21 LEU 7 54.972 74.567 23.092 1.00 0.00 H

ATOM 102 HD22 LEU 7 55.656 75.285 24.525 1.00 0.00 H

ATOM 103 HD23 LEU 7 56.086 76.019 23.185 1.00 0.00 H

ATOM 104 C LEU 7 60.030 76.936 23.418 1.00 0.00 C

ATOM 105 O LEU 7 61.170 76.446 23.684 1.00 0.00 O

ATOM 106 N THR 8 59.612 78.162 23.564 1.00 0.00 N

ATOM 107 H THR 8 58.631 78.385 23.477 1.00 0.00 H

ATOM 108 CA THR 8 60.441 79.323 23.266 1.00 0.00 C

ATOM 109 HA THR 8 61.197 79.071 22.523 1.00 0.00 H

ATOM 110 CB THR 8 59.558 80.510 22.831 1.00 0.00 C

ATOM 111 HB THR 8 58.989 80.873 23.687 1.00 0.00 H

ATOM 112 CG2 THR 8 60.215 81.760 22.158 1.00 0.00 C

ATOM 113 HG21 THR 8 60.820 81.574 21.270 1.00 0.00 H

ATOM 114 HG22 THR 8 59.376 82.438 22.000 1.00 0.00 H

ATOM 115 HG23 THR 8 60.831 82.233 22.923 1.00 0.00 H

ATOM 116 OG1 THR 8 58.698 80.048 21.866 1.00 0.00 O

ATOM 117 HG1 THR 8 57.816 80.141 22.234 1.00 0.00 H

ATOM 118 C THR 8 61.162 79.827 24.486 1.00 0.00 C

ATOM 119 O THR 8 60.618 80.026 25.555 1.00 0.00 O

ATOM 120 N LYS 9 62.465 80.103 24.381 1.00 0.00 N

ATOM 121 H LYS 9 63.007 79.810 23.581 1.00 0.00 H

ATOM 122 CA LYS 9 63.259 80.744 25.374 1.00 0.00 C

ATOM 123 HA LYS 9 63.139 80.270 26.348 1.00 0.00 H

ATOM 124 CB LYS 9 64.777 80.812 24.910 1.00 0.00 C

ATOM 125 HB2 LYS 9 64.906 81.247 23.919 1.00 0.00 H

ATOM 126 HB3 LYS 9 65.161 81.538 25.626 1.00 0.00 H

ATOM 127 CG LYS 9 65.498 79.441 24.872 1.00 0.00 C

ATOM 128 HG2 LYS 9 65.297 79.015 25.854 1.00 0.00 H

ATOM 129 HG3 LYS 9 64.997 78.792 24.153 1.00 0.00 H

ATOM 130 CD LYS 9 66.987 79.516 24.464 1.00 0.00 C

ATOM 131 HD2 LYS 9 67.008 80.150 23.578 1.00 0.00 H

ATOM 132 HD3 LYS 9 67.552 80.132 25.164 1.00 0.00 H

ATOM 133 CE LYS 9 67.656 78.134 24.329 1.00 0.00 C

ATOM 134 HE2 LYS 9 68.704 78.307 24.088 1.00 0.00 H

ATOM 135 HE3 LYS 9 67.670 77.575 25.264 1.00 0.00 H

ATOM 136 NZ LYS 9 67.105 77.269 23.285 1.00 0.00 N

ATOM 137 HZ1 LYS 9 67.511 76.345 23.240 1.00 0.00 H

ATOM 138 HZ2 LYS 9 66.104 77.142 23.339 1.00 0.00 H

ATOM 139 HZ3 LYS 9 67.328 77.658 22.380 1.00 0.00 H

ATOM 140 C LYS 9 62.781 82.238 25.540 1.00 0.00 C

ATOM 141 O LYS 9 62.958 83.081 24.683 1.00 0.00 O

ATOM 142 N THR 10 62.062 82.543 26.568 1.00 0.00 N

ATOM 143 H THR 10 61.902 81.878 27.311 1.00 0.00 H

ATOM 144 CA THR 10 61.783 83.955 26.906 1.00 0.00 C

ATOM 145 HA THR 10 61.484 84.455 25.985 1.00 0.00 H

ATOM 146 CB THR 10 60.749 84.062 28.008 1.00 0.00 C

ATOM 147 HB THR 10 60.850 85.048 28.461 1.00 0.00 H

ATOM 148 CG2 THR 10 59.330 84.100 27.408 1.00 0.00 C

ATOM 149 HG21 THR 10 58.611 84.189 28.222 1.00 0.00 H

ATOM 150 HG22 THR 10 59.260 85.024 26.834 1.00 0.00 H

ATOM 151 HG23 THR 10 59.232 83.240 26.746 1.00 0.00 H

ATOM 152 OG1 THR 10 60.725 83.138 29.057 1.00 0.00 O

ATOM 153 HG1 THR 10 60.659 82.258 28.677 1.00 0.00 H

ATOM 154 C THR 10 62.976 84.750 27.364 1.00 0.00 C

ATOM 155 O THR 10 64.038 84.264 27.622 1.00 0.00 O

ATOM 156 N GLY 11 62.912 86.081 27.147 1.00 0.00 N

ATOM 157 H GLY 11 62.168 86.440 26.566 1.00 0.00 H

ATOM 158 CA GLY 11 63.667 87.095 27.914 1.00 0.00 C

ATOM 159 HA2 GLY 11 64.653 87.068 27.449 1.00 0.00 H

ATOM 160 HA3 GLY 11 63.103 88.023 27.816 1.00 0.00 H

ATOM 161 C GLY 11 63.731 86.802 29.423 1.00 0.00 C

ATOM 162 O GLY 11 64.797 86.749 30.003 1.00 0.00 O

ATOM 163 N PHE 12 62.552 86.497 29.973 1.00 0.00 N

ATOM 164 H PHE 12 61.704 86.817 29.528 1.00 0.00 H

ATOM 165 CA PHE 12 62.470 85.854 31.253 1.00 0.00 C

ATOM 166 HA PHE 12 62.789 86.536 32.041 1.00 0.00 H

ATOM 167 CB PHE 12 60.997 85.624 31.640 1.00 0.00 C

ATOM 168 HB2 PHE 12 60.491 86.569 31.842 1.00 0.00 H

ATOM 169 HB3 PHE 12 60.483 85.271 30.746 1.00 0.00 H

ATOM 170 CG PHE 12 60.771 84.821 32.907 1.00 0.00 C

ATOM 171 CD1 PHE 12 61.230 85.414 34.118 1.00 0.00 C

ATOM 172 HD1 PHE 12 61.371 86.484 34.108 1.00 0.00 H

ATOM 173 CE1 PHE 12 61.169 84.688 35.323 1.00 0.00 C

ATOM 174 HE1 PHE 12 61.456 85.147 36.258 1.00 0.00 H

ATOM 175 CZ PHE 12 60.892 83.316 35.271 1.00 0.00 C

ATOM 176 HZ PHE 12 60.768 82.781 36.201 1.00 0.00 H

ATOM 177 CE2 PHE 12 60.508 82.694 34.046 1.00 0.00 C

ATOM 178 HE2 PHE 12 60.358 81.626 34.109 1.00 0.00 H

ATOM 179 CD2 PHE 12 60.489 83.455 32.809 1.00 0.00 C

ATOM 180 HD2 PHE 12 60.246 82.969 31.875 1.00 0.00 H

ATOM 181 C PHE 12 63.392 84.620 31.479 1.00 0.00 C

ATOM 182 O PHE 12 64.318 84.709 32.261 1.00 0.00 O

ATOM 183 N GLN 13 63.221 83.582 30.653 1.00 0.00 N

ATOM 184 H GLN 13 62.326 83.658 30.190 1.00 0.00 H

ATOM 185 CA GLN 13 64.055 82.392 30.610 1.00 0.00 C

ATOM 186 HA GLN 13 63.830 82.010 31.606 1.00 0.00 H

ATOM 187 CB GLN 13 63.598 81.447 29.599 1.00 0.00 C

ATOM 188 HB2 GLN 13 62.646 81.017 29.908 1.00 0.00 H

ATOM 189 HB3 GLN 13 63.437 82.011 28.680 1.00 0.00 H

ATOM 190 CG GLN 13 64.615 80.312 29.276 1.00 0.00 C

ATOM 191 HG2 GLN 13 65.269 80.760 28.528 1.00 0.00 H

ATOM 192 HG3 GLN 13 65.206 80.168 30.181 1.00 0.00 H

ATOM 193 CD GLN 13 63.804 79.024 28.762 1.00 0.00 C

ATOM 194 OE1 GLN 13 62.576 78.949 28.771 1.00 0.00 O

ATOM 195 NE2 GLN 13 64.505 78.013 28.273 1.00 0.00 N

ATOM 196 HE21 GLN 13 64.067 77.175 27.918 1.00 0.00 H

ATOM 197 HE22 GLN 13 65.492 78.113 28.082 1.00 0.00 H

ATOM 198 C GLN 13 65.529 82.761 30.493 1.00 0.00 C

ATOM 199 O GLN 13 66.297 82.193 31.279 1.00 0.00 O

ATOM 200 N PHE 14 65.857 83.753 29.595 1.00 0.00 N

ATOM 201 H PHE 14 65.210 83.995 28.858 1.00 0.00 H

ATOM 202 CA PHE 14 67.265 84.085 29.316 1.00 0.00 C

ATOM 203 HA PHE 14 67.816 83.167 29.115 1.00 0.00 H

ATOM 204 CB PHE 14 67.414 84.986 28.038 1.00 0.00 C

ATOM 205 HB2 PHE 14 66.616 85.727 28.079 1.00 0.00 H

ATOM 206 HB3 PHE 14 68.335 85.565 28.111 1.00 0.00 H

ATOM 207 CG PHE 14 67.539 84.255 26.652 1.00 0.00 C

ATOM 208 CD1 PHE 14 66.606 84.456 25.652 1.00 0.00 C

ATOM 209 HD1 PHE 14 65.790 85.122 25.893 1.00 0.00 H

ATOM 210 CE1 PHE 14 66.849 84.000 24.343 1.00 0.00 C

ATOM 211 HE1 PHE 14 66.176 84.348 23.574 1.00 0.00 H

ATOM 212 CZ PHE 14 67.839 83.103 24.098 1.00 0.00 C

ATOM 213 HZ PHE 14 67.770 82.602 23.144 1.00 0.00 H

ATOM 214 CE2 PHE 14 68.673 82.696 25.183 1.00 0.00 C

ATOM 215 HE2 PHE 14 69.575 82.172 24.902 1.00 0.00 H

ATOM 216 CD2 PHE 14 68.518 83.311 26.430 1.00 0.00 C

ATOM 217 HD2 PHE 14 69.340 83.312 27.131 1.00 0.00 H

ATOM 218 C PHE 14 67.936 84.802 30.561 1.00 0.00 C

ATOM 219 O PHE 14 69.072 84.370 30.889 1.00 0.00 O

ATOM 220 N TYR 15 67.310 85.827 31.148 1.00 0.00 N

ATOM 221 H TYR 15 66.507 86.180 30.647 1.00 0.00 H

ATOM 222 CA TYR 15 67.979 86.827 32.049 1.00 0.00 C

ATOM 223 HA TYR 15 69.030 86.549 32.135 1.00 0.00 H

ATOM 224 CB TYR 15 67.794 88.273 31.582 1.00 0.00 C

ATOM 225 HB2 TYR 15 66.770 88.535 31.847 1.00 0.00 H

ATOM 226 HB3 TYR 15 68.376 89.038 32.097 1.00 0.00 H

ATOM 227 CG TYR 15 67.996 88.534 30.061 1.00 0.00 C

ATOM 228 CD1 TYR 15 66.964 89.187 29.337 1.00 0.00 C

ATOM 229 HD1 TYR 15 66.082 89.554 29.842 1.00 0.00 H

ATOM 230 CE1 TYR 15 67.095 89.414 27.915 1.00 0.00 C

ATOM 231 HE1 TYR 15 66.256 89.728 27.312 1.00 0.00 H

ATOM 232 CZ TYR 15 68.351 88.984 27.346 1.00 0.00 C

ATOM 233 OH TYR 15 68.559 89.058 26.011 1.00 0.00 O

ATOM 234 HH TYR 15 69.498 89.156 25.835 1.00 0.00 H

ATOM 235 CE2 TYR 15 69.402 88.438 28.121 1.00 0.00 C

ATOM 236 HE2 TYR 15 70.352 88.216 27.657 1.00 0.00 H

ATOM 237 CD2 TYR 15 69.235 88.240 29.510 1.00 0.00 C

ATOM 238 HD2 TYR 15 70.065 87.853 30.082 1.00 0.00 H

ATOM 239 C TYR 15 67.373 86.880 33.487 1.00 0.00 C

ATOM 240 O TYR 15 68.171 87.090 34.379 1.00 0.00 O

ATOM 241 N TYR 16 66.126 86.401 33.691 1.00 0.00 N

ATOM 242 H TYR 16 65.511 86.356 32.891 1.00 0.00 H

ATOM 243 CA TYR 16 65.398 86.576 35.021 1.00 0.00 C

ATOM 244 HA TYR 16 66.040 87.156 35.683 1.00 0.00 H

ATOM 245 CB TYR 16 63.958 87.279 34.865 1.00 0.00 C

ATOM 246 HB2 TYR 16 63.538 86.839 33.961 1.00 0.00 H

ATOM 247 HB3 TYR 16 63.326 87.093 35.733 1.00 0.00 H

ATOM 248 CG TYR 16 64.071 88.753 34.535 1.00 0.00 C

ATOM 249 CD1 TYR 16 64.383 89.660 35.578 1.00 0.00 C

ATOM 250 HD1 TYR 16 64.528 89.358 36.604 1.00 0.00 H

ATOM 251 CE1 TYR 16 64.538 91.053 35.250 1.00 0.00 C

ATOM 252 HE1 TYR 16 64.916 91.719 36.011 1.00 0.00 H

ATOM 253 CZ TYR 16 64.362 91.467 33.918 1.00 0.00 C

ATOM 254 OH TYR 16 64.423 92.816 33.606 1.00 0.00 O

ATOM 255 HH TYR 16 64.022 93.032 32.761 1.00 0.00 H

ATOM 256 CE2 TYR 16 64.329 90.505 32.887 1.00 0.00 C

ATOM 257 HE2 TYR 16 64.235 90.863 31.872 1.00 0.00 H

ATOM 258 CD2 TYR 16 64.103 89.147 33.188 1.00 0.00 C

ATOM 259 HD2 TYR 16 63.809 88.470 32.400 1.00 0.00 H

ATOM 260 C TYR 16 65.293 85.184 35.705 1.00 0.00 C

ATOM 261 O TYR 16 65.173 85.114 36.941 1.00 0.00 O

ATOM 262 N LEU 17 65.350 84.021 34.992 1.00 0.00 N

ATOM 263 H LEU 17 65.158 84.001 34.001 1.00 0.00 H

ATOM 264 CA LEU 17 65.515 82.707 35.657 1.00 0.00 C

ATOM 265 HA LEU 17 64.753 82.714 36.437 1.00 0.00 H

ATOM 266 CB LEU 17 65.018 81.588 34.692 1.00 0.00 C

ATOM 267 HB2 LEU 17 64.189 81.998 34.114 1.00 0.00 H

ATOM 268 HB3 LEU 17 65.821 81.350 33.995 1.00 0.00 H

ATOM 269 CG LEU 17 64.549 80.297 35.360 1.00 0.00 C

ATOM 270 HG LEU 17 65.399 79.750 35.768 1.00 0.00 H

ATOM 271 CD1 LEU 17 63.432 80.439 36.460 1.00 0.00 C

ATOM 272 HD11 LEU 17 63.034 79.469 36.757 1.00 0.00 H

ATOM 273 HD12 LEU 17 63.857 80.980 37.305 1.00 0.00 H

ATOM 274 HD13 LEU 17 62.584 80.968 36.024 1.00 0.00 H

ATOM 275 CD2 LEU 17 63.859 79.548 34.189 1.00 0.00 C

ATOM 276 HD21 LEU 17 63.512 78.549 34.449 1.00 0.00 H

ATOM 277 HD22 LEU 17 62.879 80.008 34.062 1.00 0.00 H

ATOM 278 HD23 LEU 17 64.356 79.543 33.219 1.00 0.00 H

ATOM 279 C LEU 17 66.931 82.346 36.221 1.00 0.00 C

ATOM 280 O LEU 17 67.005 81.979 37.382 1.00 0.00 O

ATOM 281 N PRO 18 68.017 82.890 35.604 1.00 0.00 N

ATOM 282 CD PRO 18 68.234 83.567 34.358 1.00 0.00 C

ATOM 283 HD2 PRO 18 67.791 84.544 34.547 1.00 0.00 H

ATOM 284 HD3 PRO 18 67.854 83.001 33.507 1.00 0.00 H

ATOM 285 CG PRO 18 69.822 83.697 34.101 1.00 0.00 C

ATOM 286 HG2 PRO 18 70.055 84.725 33.824 1.00 0.00 H

ATOM 287 HG3 PRO 18 70.122 83.091 33.247 1.00 0.00 H

ATOM 288 CB PRO 18 70.454 83.286 35.395 1.00 0.00 C

ATOM 289 HB2 PRO 18 71.175 84.005 35.784 1.00 0.00 H

ATOM 290 HB3 PRO 18 70.990 82.345 35.273 1.00 0.00 H

ATOM 291 CA PRO 18 69.265 82.961 36.334 1.00 0.00 C

ATOM 292 HA PRO 18 69.483 81.937 36.639 1.00 0.00 H

ATOM 293 C PRO 18 69.263 83.833 37.573 1.00 0.00 C

ATOM 294 O PRO 18 69.567 83.419 38.707 1.00 0.00 O

ATOM 295 N ALA 19 68.488 84.996 37.539 1.00 0.00 N

ATOM 296 H ALA 19 67.868 85.146 36.755 1.00 0.00 H

ATOM 297 CA ALA 19 68.281 85.908 38.672 1.00 0.00 C

ATOM 298 HA ALA 19 69.302 86.163 38.958 1.00 0.00 H

ATOM 299 CB ALA 19 67.752 87.282 38.187 1.00 0.00 C

ATOM 300 HB1 ALA 19 66.891 87.175 37.527 1.00 0.00 H

ATOM 301 HB2 ALA 19 67.587 87.856 39.098 1.00 0.00 H

ATOM 302 HB3 ALA 19 68.554 87.815 37.677 1.00 0.00 H

ATOM 303 C ALA 19 67.517 85.372 39.842 1.00 0.00 C

ATOM 304 O ALA 19 67.966 85.475 40.917 1.00 0.00 O

ATOM 305 N VAL 20 66.425 84.615 39.573 1.00 0.00 N

ATOM 306 H VAL 20 66.069 84.567 38.629 1.00 0.00 H

ATOM 307 CA VAL 20 65.901 83.733 40.607 1.00 0.00 C

ATOM 308 HA VAL 20 65.672 84.361 41.468 1.00 0.00 H

ATOM 309 CB VAL 20 64.610 82.980 40.103 1.00 0.00 C

ATOM 310 HB VAL 20 64.799 82.315 39.261 1.00 0.00 H

ATOM 311 CG1 VAL 20 64.066 82.061 41.215 1.00 0.00 C

ATOM 312 HG11 VAL 20 64.722 81.191 41.192 1.00 0.00 H

ATOM 313 HG12 VAL 20 64.215 82.625 42.135 1.00 0.00 H

ATOM 314 HG13 VAL 20 63.033 81.896 40.907 1.00 0.00 H

ATOM 315 CG2 VAL 20 63.540 84.015 39.773 1.00 0.00 C

ATOM 316 HG21 VAL 20 63.168 84.676 40.556 1.00 0.00 H

ATOM 317 HG22 VAL 20 63.829 84.681 38.961 1.00 0.00 H

ATOM 318 HG23 VAL 20 62.729 83.529 39.230 1.00 0.00 H

ATOM 319 C VAL 20 66.936 82.775 41.191 1.00 0.00 C

ATOM 320 O VAL 20 67.006 82.698 42.479 1.00 0.00 O

ATOM 321 N TYR 21 67.713 82.022 40.422 1.00 0.00 N

ATOM 322 H TYR 21 67.517 81.956 39.434 1.00 0.00 H

ATOM 323 CA TYR 21 68.542 80.913 40.862 1.00 0.00 C

ATOM 324 HA TYR 21 67.966 80.434 41.653 1.00 0.00 H

ATOM 325 CB TYR 21 68.995 80.006 39.665 1.00 0.00 C

ATOM 326 HB2 TYR 21 69.157 80.582 38.754 1.00 0.00 H

ATOM 327 HB3 TYR 21 69.916 79.486 39.926 1.00 0.00 H

ATOM 328 CG TYR 21 67.913 78.895 39.389 1.00 0.00 C

ATOM 329 CD1 TYR 21 67.127 78.842 38.213 1.00 0.00 C

ATOM 330 HD1 TYR 21 67.301 79.559 37.424 1.00 0.00 H

ATOM 331 CE1 TYR 21 65.859 78.076 38.227 1.00 0.00 C

ATOM 332 HE1 TYR 21 65.271 77.955 37.329 1.00 0.00 H

ATOM 333 CZ TYR 21 65.577 77.211 39.343 1.00 0.00 C

ATOM 334 OH TYR 21 64.596 76.231 39.216 1.00 0.00 O

ATOM 335 HH TYR 21 64.492 75.820 40.078 1.00 0.00 H

ATOM 336 CE2 TYR 21 66.498 77.118 40.416 1.00 0.00 C

ATOM 337 HE2 TYR 21 66.501 76.471 41.281 1.00 0.00 H

ATOM 338 CD2 TYR 21 67.569 77.992 40.436 1.00 0.00 C

ATOM 339 HD2 TYR 21 68.101 78.158 41.361 1.00 0.00 H

ATOM 340 C TYR 21 69.782 81.367 41.686 1.00 0.00 C

ATOM 341 O TYR 21 70.039 80.855 42.760 1.00 0.00 O

ATOM 342 N ILE 22 70.480 82.429 41.229 1.00 0.00 N

ATOM 343 H ILE 22 70.338 82.709 40.269 1.00 0.00 H

ATOM 344 CA ILE 22 71.497 83.115 42.080 1.00 0.00 C

ATOM 345 HA ILE 22 72.247 82.361 42.318 1.00 0.00 H

ATOM 346 CB ILE 22 72.212 84.180 41.272 1.00 0.00 C

ATOM 347 HB ILE 22 73.066 84.500 41.870 1.00 0.00 H

ATOM 348 CG2 ILE 22 72.856 83.506 40.054 1.00 0.00 C

ATOM 349 HG21 ILE 22 73.264 84.350 39.497 1.00 0.00 H

ATOM 350 HG22 ILE 22 73.746 82.984 40.405 1.00 0.00 H

ATOM 351 HG23 ILE 22 72.157 82.882 39.497 1.00 0.00 H

ATOM 352 CG1 ILE 22 71.368 85.283 40.807 1.00 0.00 C

ATOM 353 HG12 ILE 22 70.445 84.827 40.448 1.00 0.00 H

ATOM 354 HG13 ILE 22 71.201 85.867 41.712 1.00 0.00 H

ATOM 355 CD1 ILE 22 71.826 86.314 39.748 1.00 0.00 C

ATOM 356 HD11 ILE 22 71.783 85.898 38.741 1.00 0.00 H

ATOM 357 HD12 ILE 22 71.185 87.191 39.834 1.00 0.00 H

ATOM 358 HD13 ILE 22 72.834 86.636 40.010 1.00 0.00 H

ATOM 359 C ILE 22 70.991 83.721 43.378 1.00 0.00 C

ATOM 360 O ILE 22 71.802 83.826 44.285 1.00 0.00 O

ATOM 361 N LEU 23 69.737 84.179 43.458 1.00 0.00 N

ATOM 362 H LEU 23 69.199 84.233 42.605 1.00 0.00 H

ATOM 363 CA LEU 23 69.144 84.687 44.661 1.00 0.00 C

ATOM 364 HA LEU 23 69.760 85.423 45.178 1.00 0.00 H

ATOM 365 CB LEU 23 67.930 85.629 44.257 1.00 0.00 C

ATOM 366 HB2 LEU 23 67.428 85.150 43.416 1.00 0.00 H

ATOM 367 HB3 LEU 23 67.245 85.666 45.104 1.00 0.00 H

ATOM 368 CG LEU 23 68.249 87.111 43.962 1.00 0.00 C

ATOM 369 HG LEU 23 69.148 87.194 43.351 1.00 0.00 H

ATOM 370 CD1 LEU 23 66.984 87.809 43.366 1.00 0.00 C

ATOM 371 HD11 LEU 23 66.843 87.571 42.311 1.00 0.00 H

ATOM 372 HD12 LEU 23 66.073 87.582 43.919 1.00 0.00 H

ATOM 373 HD13 LEU 23 67.159 88.883 43.433 1.00 0.00 H

ATOM 374 CD2 LEU 23 68.532 87.824 45.249 1.00 0.00 C

ATOM 375 HD21 LEU 23 67.577 88.107 45.691 1.00 0.00 H

ATOM 376 HD22 LEU 23 69.144 87.247 45.942 1.00 0.00 H

ATOM 377 HD23 LEU 23 69.131 88.711 45.040 1.00 0.00 H

ATOM 378 C LEU 23 68.699 83.652 45.672 1.00 0.00 C

ATOM 379 O LEU 23 68.885 83.977 46.885 1.00 0.00 O

ATOM 380 N VAL 24 68.015 82.631 45.206 1.00 0.00 N

ATOM 381 H VAL 24 67.699 82.680 44.248 1.00 0.00 H

ATOM 382 CA VAL 24 67.644 81.445 45.971 1.00 0.00 C

ATOM 383 HA VAL 24 67.007 81.681 46.823 1.00 0.00 H

ATOM 384 CB VAL 24 66.791 80.345 45.195 1.00 0.00 C

ATOM 385 HB VAL 24 67.300 79.934 44.323 1.00 0.00 H

ATOM 386 CG1 VAL 24 66.349 79.123 45.966 1.00 0.00 C

ATOM 387 HG11 VAL 24 65.815 78.411 45.337 1.00 0.00 H

ATOM 388 HG12 VAL 24 67.200 78.533 46.305 1.00 0.00 H

ATOM 389 HG13 VAL 24 65.786 79.467 46.834 1.00 0.00 H

ATOM 390 CG2 VAL 24 65.474 81.053 44.635 1.00 0.00 C

ATOM 391 HG21 VAL 24 65.036 80.415 43.867 1.00 0.00 H

ATOM 392 HG22 VAL 24 64.761 81.229 45.441 1.00 0.00 H

ATOM 393 HG23 VAL 24 65.709 82.051 44.268 1.00 0.00 H

ATOM 394 C VAL 24 68.884 80.719 46.560 1.00 0.00 C

ATOM 395 O VAL 24 68.973 80.304 47.679 1.00 0.00 O

ATOM 396 N PHE 25 70.045 80.674 45.786 1.00 0.00 N

ATOM 397 H PHE 25 69.986 80.764 44.782 1.00 0.00 H

ATOM 398 CA PHE 25 71.332 80.141 46.362 1.00 0.00 C

ATOM 399 HA PHE 25 71.044 79.245 46.913 1.00 0.00 H

ATOM 400 CB PHE 25 72.295 79.892 45.117 1.00 0.00 C

ATOM 401 HB2 PHE 25 71.864 79.266 44.336 1.00 0.00 H

ATOM 402 HB3 PHE 25 72.464 80.868 44.663 1.00 0.00 H

ATOM 403 CG PHE 25 73.644 79.441 45.509 1.00 0.00 C

ATOM 404 CD1 PHE 25 73.723 78.219 46.131 1.00 0.00 C

ATOM 405 HD1 PHE 25 72.824 77.621 46.123 1.00 0.00 H

ATOM 406 CE1 PHE 25 74.947 77.624 46.660 1.00 0.00 C

ATOM 407 HE1 PHE 25 74.965 76.680 47.184 1.00 0.00 H

ATOM 408 CZ PHE 25 76.088 78.355 46.523 1.00 0.00 C

ATOM 409 HZ PHE 25 76.943 77.937 47.033 1.00 0.00 H

ATOM 410 CE2 PHE 25 76.031 79.574 45.815 1.00 0.00 C

ATOM 411 HE2 PHE 25 76.907 80.206 45.844 1.00 0.00 H

ATOM 412 CD2 PHE 25 74.828 80.197 45.439 1.00 0.00 C

ATOM 413 HD2 PHE 25 74.717 81.195 45.041 1.00 0.00 H

ATOM 414 C PHE 25 71.909 81.192 47.309 1.00 0.00 C

ATOM 415 O PHE 25 72.308 80.795 48.431 1.00 0.00 O

ATOM 416 N ILE 26 72.034 82.508 46.935 1.00 0.00 N

ATOM 417 H ILE 26 71.694 82.825 46.039 1.00 0.00 H

ATOM 418 CA ILE 26 72.827 83.449 47.782 1.00 0.00 C

ATOM 419 HA ILE 26 73.686 82.879 48.134 1.00 0.00 H

ATOM 420 CB ILE 26 73.230 84.727 47.069 1.00 0.00 C

ATOM 421 HB ILE 26 73.486 84.474 46.041 1.00 0.00 H

ATOM 422 CG2 ILE 26 72.096 85.683 47.024 1.00 0.00 C

ATOM 423 HG21 ILE 26 71.192 85.209 46.642 1.00 0.00 H

ATOM 424 HG22 ILE 26 71.909 85.955 48.063 1.00 0.00 H

ATOM 425 HG23 ILE 26 72.498 86.554 46.507 1.00 0.00 H

ATOM 426 CG1 ILE 26 74.534 85.250 47.855 1.00 0.00 C

ATOM 427 HG12 ILE 26 74.300 85.504 48.889 1.00 0.00 H

ATOM 428 HG13 ILE 26 75.355 84.556 47.678 1.00 0.00 H

ATOM 429 CD1 ILE 26 75.189 86.641 47.443 1.00 0.00 C

ATOM 430 HD11 ILE 26 74.517 87.498 47.470 1.00 0.00 H

ATOM 431 HD12 ILE 26 75.949 86.908 48.177 1.00 0.00 H

ATOM 432 HD13 ILE 26 75.469 86.698 46.390 1.00 0.00 H

ATOM 433 C ILE 26 72.138 83.674 49.141 1.00 0.00 C

ATOM 434 O ILE 26 72.916 83.838 50.052 1.00 0.00 O

ATOM 435 N ILE 27 70.799 83.677 49.324 1.00 0.00 N

ATOM 436 H ILE 27 70.226 83.913 48.527 1.00 0.00 H

ATOM 437 CA ILE 27 70.029 83.466 50.560 1.00 0.00 C

ATOM 438 HA ILE 27 70.587 84.043 51.297 1.00 0.00 H

ATOM 439 CB ILE 27 68.582 84.063 50.376 1.00 0.00 C

ATOM 440 HB ILE 27 68.132 83.582 49.508 1.00 0.00 H

ATOM 441 CG2 ILE 27 67.760 83.628 51.603 1.00 0.00 C

ATOM 442 HG21 ILE 27 67.012 84.414 51.707 1.00 0.00 H

ATOM 443 HG22 ILE 27 67.359 82.622 51.478 1.00 0.00 H

ATOM 444 HG23 ILE 27 68.428 83.805 52.446 1.00 0.00 H

ATOM 445 CG1 ILE 27 68.610 85.589 50.193 1.00 0.00 C

ATOM 446 HG12 ILE 27 68.686 86.065 51.171 1.00 0.00 H

ATOM 447 HG13 ILE 27 69.519 85.882 49.668 1.00 0.00 H

ATOM 448 CD1 ILE 27 67.485 86.042 49.433 1.00 0.00 C

ATOM 449 HD11 ILE 27 67.376 87.126 49.455 1.00 0.00 H

ATOM 450 HD12 ILE 27 67.511 85.707 48.396 1.00 0.00 H

ATOM 451 HD13 ILE 27 66.606 85.533 49.827 1.00 0.00 H

ATOM 452 C ILE 27 70.096 81.940 51.023 1.00 0.00 C

ATOM 453 O ILE 27 70.535 81.612 52.130 1.00 0.00 O

ATOM 454 N GLY 28 69.567 81.075 50.163 1.00 0.00 N

ATOM 455 H GLY 28 69.494 81.319 49.186 1.00 0.00 H

ATOM 456 CA GLY 28 69.156 79.763 50.588 1.00 0.00 C

ATOM 457 HA2 GLY 28 68.297 79.928 51.238 1.00 0.00 H

ATOM 458 HA3 GLY 28 68.740 79.204 49.750 1.00 0.00 H

ATOM 459 C GLY 28 70.253 78.860 51.267 1.00 0.00 C

ATOM 460 O GLY 28 69.940 78.171 52.207 1.00 0.00 O

ATOM 461 N PHE 29 71.439 78.895 50.682 1.00 0.00 N

ATOM 462 H PHE 29 71.617 79.524 49.911 1.00 0.00 H

ATOM 463 CA PHE 29 72.598 78.072 51.078 1.00 0.00 C

ATOM 464 HA PHE 29 72.293 77.025 51.094 1.00 0.00 H

ATOM 465 CB PHE 29 73.612 77.969 49.923 1.00 0.00 C

ATOM 466 HB2 PHE 29 73.068 77.525 49.090 1.00 0.00 H

ATOM 467 HB3 PHE 29 73.976 78.923 49.540 1.00 0.00 H

ATOM 468 CG PHE 29 74.780 77.078 50.103 1.00 0.00 C

ATOM 469 CD1 PHE 29 74.639 75.691 50.134 1.00 0.00 C

ATOM 470 HD1 PHE 29 73.704 75.305 49.755 1.00 0.00 H

ATOM 471 CE1 PHE 29 75.752 74.862 50.379 1.00 0.00 C

ATOM 472 HE1 PHE 29 75.468 73.822 50.434 1.00 0.00 H

ATOM 473 CZ PHE 29 76.972 75.329 50.734 1.00 0.00 C

ATOM 474 HZ PHE 29 77.790 74.641 50.889 1.00 0.00 H

ATOM 475 CE2 PHE 29 77.089 76.753 50.777 1.00 0.00 C

ATOM 476 HE2 PHE 29 78.079 77.185 50.811 1.00 0.00 H

ATOM 477 CD2 PHE 29 76.017 77.591 50.391 1.00 0.00 C

ATOM 478 HD2 PHE 29 76.147 78.663 50.352 1.00 0.00 H

ATOM 479 C PHE 29 73.152 78.505 52.400 1.00 0.00 C

ATOM 480 O PHE 29 73.110 77.724 53.356 1.00 0.00 O

ATOM 481 N LEU 30 73.615 79.778 52.429 1.00 0.00 N

ATOM 482 H LEU 30 73.699 80.288 51.561 1.00 0.00 H

ATOM 483 CA LEU 30 73.912 80.508 53.604 1.00 0.00 C

ATOM 484 HA LEU 30 74.820 80.033 53.977 1.00 0.00 H

ATOM 485 CB LEU 30 74.200 81.991 53.291 1.00 0.00 C

ATOM 486 HB2 LEU 30 73.289 82.343 52.807 1.00 0.00 H

ATOM 487 HB3 LEU 30 74.295 82.577 54.205 1.00 0.00 H

ATOM 488 CG LEU 30 75.322 82.284 52.346 1.00 0.00 C

ATOM 489 HG LEU 30 74.856 82.081 51.382 1.00 0.00 H

ATOM 490 CD1 LEU 30 75.788 83.721 52.452 1.00 0.00 C

ATOM 491 HD11 LEU 30 75.724 84.160 53.447 1.00 0.00 H

ATOM 492 HD12 LEU 30 76.785 83.715 52.010 1.00 0.00 H

ATOM 493 HD13 LEU 30 75.080 84.272 51.832 1.00 0.00 H

ATOM 494 CD2 LEU 30 76.532 81.349 52.691 1.00 0.00 C

ATOM 495 HD21 LEU 30 76.815 81.410 53.742 1.00 0.00 H

ATOM 496 HD22 LEU 30 76.283 80.296 52.558 1.00 0.00 H

ATOM 497 HD23 LEU 30 77.374 81.591 52.043 1.00 0.00 H

ATOM 498 C LEU 30 72.845 80.309 54.733 1.00 0.00 C

ATOM 499 O LEU 30 73.281 80.216 55.836 1.00 0.00 O

ATOM 500 N GLY 31 71.567 80.335 54.459 1.00 0.00 N

ATOM 501 H GLY 31 71.217 80.702 53.585 1.00 0.00 H

ATOM 502 CA GLY 31 70.496 80.309 55.435 1.00 0.00 C

ATOM 503 HA2 GLY 31 70.814 80.906 56.289 1.00 0.00 H

ATOM 504 HA3 GLY 31 69.541 80.642 55.027 1.00 0.00 H

ATOM 505 C GLY 31 70.230 78.955 56.005 1.00 0.00 C

ATOM 506 O GLY 31 70.372 78.798 57.215 1.00 0.00 O

ATOM 507 N ASN 32 70.271 77.867 55.221 1.00 0.00 N

ATOM 508 H ASN 32 70.391 78.099 54.246 1.00 0.00 H

ATOM 509 CA ASN 32 70.470 76.485 55.614 1.00 0.00 C

ATOM 510 HA ASN 32 69.698 76.195 56.328 1.00 0.00 H

ATOM 511 CB ASN 32 70.231 75.520 54.439 1.00 0.00 C

ATOM 512 HB2 ASN 32 70.705 75.823 53.505 1.00 0.00 H

ATOM 513 HB3 ASN 32 70.563 74.534 54.766 1.00 0.00 H

ATOM 514 CG ASN 32 68.743 75.386 54.198 1.00 0.00 C

ATOM 515 OD1 ASN 32 68.016 74.812 54.982 1.00 0.00 O

ATOM 516 ND2 ASN 32 68.296 75.788 53.020 1.00 0.00 N

ATOM 517 HD21 ASN 32 68.809 76.518 52.548 1.00 0.00 H

ATOM 518 HD22 ASN 32 67.421 75.390 52.711 1.00 0.00 H

ATOM 519 C ASN 32 71.836 76.331 56.254 1.00 0.00 C

ATOM 520 O ASN 32 71.946 75.470 57.134 1.00 0.00 O

ATOM 521 N SER 33 72.935 77.011 55.919 1.00 0.00 N

ATOM 522 H SER 33 72.801 77.720 55.212 1.00 0.00 H

ATOM 523 CA SER 33 74.306 76.895 56.417 1.00 0.00 C

ATOM 524 HA SER 33 74.569 75.852 56.595 1.00 0.00 H

ATOM 525 CB SER 33 75.384 77.648 55.627 1.00 0.00 C

ATOM 526 HB2 SER 33 75.152 78.706 55.510 1.00 0.00 H

ATOM 527 HB3 SER 33 76.312 77.560 56.192 1.00 0.00 H

ATOM 528 OG SER 33 75.420 76.946 54.404 1.00 0.00 O

ATOM 529 HG SER 33 74.550 77.127 54.041 1.00 0.00 H

ATOM 530 C SER 33 74.420 77.473 57.870 1.00 0.00 C

ATOM 531 O SER 33 75.104 76.890 58.705 1.00 0.00 O

ATOM 532 N VAL 34 73.645 78.519 58.252 1.00 0.00 N

ATOM 533 H VAL 34 73.213 79.128 57.572 1.00 0.00 H

ATOM 534 CA VAL 34 73.316 78.759 59.682 1.00 0.00 C

ATOM 535 HA VAL 34 74.252 78.849 60.234 1.00 0.00 H

ATOM 536 CB VAL 34 72.589 80.125 59.908 1.00 0.00 C

ATOM 537 HB VAL 34 71.546 80.058 59.602 1.00 0.00 H

ATOM 538 CG1 VAL 34 72.615 80.578 61.374 1.00 0.00 C

ATOM 539 HG11 VAL 34 73.587 80.323 61.797 1.00 0.00 H

ATOM 540 HG12 VAL 34 72.619 81.626 61.672 1.00 0.00 H

ATOM 541 HG13 VAL 34 71.795 80.171 61.964 1.00 0.00 H

ATOM 542 CG2 VAL 34 73.152 81.323 59.133 1.00 0.00 C

ATOM 543 HG21 VAL 34 74.046 81.754 59.584 1.00 0.00 H

ATOM 544 HG22 VAL 34 73.181 81.180 58.053 1.00 0.00 H

ATOM 545 HG23 VAL 34 72.434 82.114 59.353 1.00 0.00 H

ATOM 546 C VAL 34 72.556 77.650 60.339 1.00 0.00 C

ATOM 547 O VAL 34 72.836 77.261 61.474 1.00 0.00 O

ATOM 548 N ALA 35 71.391 77.215 59.865 1.00 0.00 N

ATOM 549 H ALA 35 71.083 77.618 58.991 1.00 0.00 H

ATOM 550 CA ALA 35 70.577 76.195 60.446 1.00 0.00 C

ATOM 551 HA ALA 35 70.311 76.491 61.461 1.00 0.00 H

ATOM 552 CB ALA 35 69.232 76.190 59.690 1.00 0.00 C

ATOM 553 HB1 ALA 35 68.586 75.535 60.273 1.00 0.00 H

ATOM 554 HB2 ALA 35 68.832 77.202 59.757 1.00 0.00 H

ATOM 555 HB3 ALA 35 69.246 75.782 58.680 1.00 0.00 H

ATOM 556 C ALA 35 71.182 74.813 60.636 1.00 0.00 C

ATOM 557 O ALA 35 70.957 74.264 61.659 1.00 0.00 O

ATOM 558 N ILE 36 71.974 74.245 59.701 1.00 0.00 N

ATOM 559 H ILE 36 72.031 74.710 58.807 1.00 0.00 H

ATOM 560 CA ILE 36 72.689 73.019 59.970 1.00 0.00 C

ATOM 561 HA ILE 36 72.015 72.416 60.578 1.00 0.00 H

ATOM 562 CB ILE 36 73.185 72.337 58.605 1.00 0.00 C

ATOM 563 HB ILE 36 72.338 72.365 57.920 1.00 0.00 H

ATOM 564 CG2 ILE 36 74.362 73.120 58.114 1.00 0.00 C

ATOM 565 HG21 ILE 36 74.585 72.933 57.064 1.00 0.00 H

ATOM 566 HG22 ILE 36 74.335 74.201 58.249 1.00 0.00 H

ATOM 567 HG23 ILE 36 75.170 72.733 58.736 1.00 0.00 H

ATOM 568 CG1 ILE 36 73.529 70.828 58.706 1.00 0.00 C

ATOM 569 HG12 ILE 36 73.984 70.429 57.798 1.00 0.00 H

ATOM 570 HG13 ILE 36 74.218 70.546 59.503 1.00 0.00 H

ATOM 571 CD1 ILE 36 72.344 69.936 58.958 1.00 0.00 C

ATOM 572 HD11 ILE 36 72.806 68.950 58.994 1.00 0.00 H

ATOM 573 HD12 ILE 36 71.683 70.072 59.814 1.00 0.00 H

ATOM 574 HD13 ILE 36 71.671 69.939 58.101 1.00 0.00 H

ATOM 575 C ILE 36 73.926 73.224 60.885 1.00 0.00 C

ATOM 576 O ILE 36 74.099 72.280 61.697 1.00 0.00 O

ATOM 577 N TRP 37 74.504 74.440 61.009 1.00 0.00 N

ATOM 578 H TRP 37 74.149 75.194 60.439 1.00 0.00 H

ATOM 579 CA TRP 37 75.414 74.787 62.124 1.00 0.00 C

ATOM 580 HA TRP 37 76.185 74.016 62.137 1.00 0.00 H

ATOM 581 CB TRP 37 76.135 76.174 61.926 1.00 0.00 C

ATOM 582 HB2 TRP 37 76.509 76.326 60.914 1.00 0.00 H

ATOM 583 HB3 TRP 37 75.446 76.979 62.182 1.00 0.00 H

ATOM 584 CG TRP 37 77.274 76.415 62.842 1.00 0.00 C

ATOM 585 CD1 TRP 37 77.307 76.578 64.146 1.00 0.00 C

ATOM 586 HD1 TRP 37 76.428 76.728 64.755 1.00 0.00 H

ATOM 587 NE1 TRP 37 78.658 76.718 64.571 1.00 0.00 N

ATOM 588 HE1 TRP 37 78.898 76.762 65.551 1.00 0.00 H

ATOM 589 CE2 TRP 37 79.564 76.328 63.598 1.00 0.00 C

ATOM 590 CZ2 TRP 37 80.927 76.193 63.654 1.00 0.00 C

ATOM 591 HZ2 TRP 37 81.438 76.050 64.594 1.00 0.00 H

ATOM 592 CH2 TRP 37 81.577 75.825 62.470 1.00 0.00 C

ATOM 593 HH2 TRP 37 82.647 75.681 62.456 1.00 0.00 H

ATOM 594 CZ3 TRP 37 80.892 75.998 61.253 1.00 0.00 C

ATOM 595 HZ3 TRP 37 81.308 75.800 60.276 1.00 0.00 H

ATOM 596 CE3 TRP 37 79.534 76.290 61.262 1.00 0.00 C

ATOM 597 HE3 TRP 37 78.978 76.290 60.336 1.00 0.00 H

ATOM 598 CD2 TRP 37 78.758 76.336 62.452 1.00 0.00 C

ATOM 599 C TRP 37 74.704 74.652 63.494 1.00 0.00 C

ATOM 600 O TRP 37 75.248 74.038 64.350 1.00 0.00 O

ATOM 601 N MET 38 73.407 74.999 63.486 1.00 0.00 N

ATOM 602 H MET 38 73.035 75.660 62.819 1.00 0.00 H

ATOM 603 CA MET 38 72.552 74.788 64.664 1.00 0.00 C

ATOM 604 HA MET 38 73.044 75.335 65.468 1.00 0.00 H

ATOM 605 CB MET 38 71.206 75.632 64.591 1.00 0.00 C

ATOM 606 HB2 MET 38 71.207 76.341 63.763 1.00 0.00 H

ATOM 607 HB3 MET 38 70.398 74.946 64.339 1.00 0.00 H

ATOM 608 CG MET 38 70.839 76.359 65.858 1.00 0.00 C

ATOM 609 HG2 MET 38 71.532 77.160 66.116 1.00 0.00 H

ATOM 610 HG3 MET 38 69.845 76.702 65.571 1.00 0.00 H

ATOM 611 SD MET 38 70.803 75.313 67.247 1.00 0.00 S

ATOM 612 CE MET 38 69.414 76.183 68.118 1.00 0.00 C

ATOM 613 HE1 MET 38 68.625 76.479 67.427 1.00 0.00 H

ATOM 614 HE2 MET 38 69.053 75.426 68.814 1.00 0.00 H

ATOM 615 HE3 MET 38 69.744 77.112 68.584 1.00 0.00 H

ATOM 616 C MET 38 72.306 73.308 65.068 1.00 0.00 C

ATOM 617 O MET 38 72.705 72.859 66.137 1.00 0.00 O

ATOM 618 N PHE 39 71.791 72.522 64.109 1.00 0.00 N

ATOM 619 H PHE 39 71.548 72.996 63.251 1.00 0.00 H

ATOM 620 CA PHE 39 71.336 71.141 64.299 1.00 0.00 C

ATOM 621 HA PHE 39 70.720 71.100 65.197 1.00 0.00 H

ATOM 622 CB PHE 39 70.558 70.593 63.092 1.00 0.00 C

ATOM 623 HB2 PHE 39 71.289 70.495 62.289 1.00 0.00 H

ATOM 624 HB3 PHE 39 70.276 69.556 63.277 1.00 0.00 H

ATOM 625 CG PHE 39 69.292 71.293 62.699 1.00 0.00 C

ATOM 626 CD1 PHE 39 69.104 71.619 61.365 1.00 0.00 C

ATOM 627 HD1 PHE 39 69.723 71.009 60.723 1.00 0.00 H

ATOM 628 CE1 PHE 39 67.949 72.313 60.944 1.00 0.00 C

ATOM 629 HE1 PHE 39 67.740 72.468 59.896 1.00 0.00 H

ATOM 630 CZ PHE 39 66.870 72.544 61.851 1.00 0.00 C

ATOM 631 HZ PHE 39 65.870 72.853 61.588 1.00 0.00 H

ATOM 632 CE2 PHE 39 67.052 72.147 63.229 1.00 0.00 C

ATOM 633 HE2 PHE 39 66.338 72.319 64.020 1.00 0.00 H

ATOM 634 CD2 PHE 39 68.278 71.594 63.653 1.00 0.00 C

ATOM 635 HD2 PHE 39 68.469 71.451 64.706 1.00 0.00 H

ATOM 636 C PHE 39 72.563 70.301 64.831 1.00 0.00 C

ATOM 637 O PHE 39 72.422 69.497 65.755 1.00 0.00 O

ATOM 638 N VAL 40 73.748 70.450 64.236 1.00 0.00 N

ATOM 639 H VAL 40 74.005 70.968 63.408 1.00 0.00 H

ATOM 640 CA VAL 40 74.811 69.511 64.525 1.00 0.00 C

ATOM 641 HA VAL 40 74.314 68.578 64.792 1.00 0.00 H

ATOM 642 CB VAL 40 75.516 69.109 63.219 1.00 0.00 C

ATOM 643 HB VAL 40 74.745 69.206 62.455 1.00 0.00 H

ATOM 644 CG1 VAL 40 76.582 70.129 62.848 1.00 0.00 C

ATOM 645 HG11 VAL 40 76.688 70.082 61.765 1.00 0.00 H

ATOM 646 HG12 VAL 40 76.392 71.171 63.106 1.00 0.00 H

ATOM 647 HG13 VAL 40 77.503 69.859 63.364 1.00 0.00 H

ATOM 648 CG2 VAL 40 75.834 67.626 63.139 1.00 0.00 C

ATOM 649 HG21 VAL 40 74.913 67.092 63.371 1.00 0.00 H

ATOM 650 HG22 VAL 40 76.120 67.280 62.145 1.00 0.00 H

ATOM 651 HG23 VAL 40 76.600 67.248 63.817 1.00 0.00 H

ATOM 652 C VAL 40 75.780 69.942 65.647 1.00 0.00 C

ATOM 653 O VAL 40 76.561 69.099 66.109 1.00 0.00 O

ATOM 654 N PHE 41 75.949 71.270 65.896 1.00 0.00 N

ATOM 655 H PHE 41 75.506 71.997 65.353 1.00 0.00 H

ATOM 656 CA PHE 41 76.997 71.818 66.840 1.00 0.00 C

ATOM 657 HA PHE 41 77.560 70.935 67.143 1.00 0.00 H

ATOM 658 CB PHE 41 77.987 72.746 66.064 1.00 0.00 C

ATOM 659 HB2 PHE 41 77.862 72.308 65.073 1.00 0.00 H

ATOM 660 HB3 PHE 41 77.642 73.779 66.030 1.00 0.00 H

ATOM 661 CG PHE 41 79.392 72.681 66.420 1.00 0.00 C

ATOM 662 CD1 PHE 41 80.369 73.240 65.515 1.00 0.00 C

ATOM 663 HD1 PHE 41 80.071 73.600 64.541 1.00 0.00 H

ATOM 664 CE1 PHE 41 81.757 73.421 65.869 1.00 0.00 C

ATOM 665 HE1 PHE 41 82.521 73.866 65.249 1.00 0.00 H

ATOM 666 CZ PHE 41 82.165 73.014 67.148 1.00 0.00 C

ATOM 667 HZ PHE 41 83.131 73.362 67.485 1.00 0.00 H

ATOM 668 CE2 PHE 41 81.202 72.507 68.035 1.00 0.00 C

ATOM 669 HE2 PHE 41 81.399 72.287 69.074 1.00 0.00 H

ATOM 670 CD2 PHE 41 79.827 72.383 67.669 1.00 0.00 C

ATOM 671 HD2 PHE 41 79.148 72.171 68.482 1.00 0.00 H

ATOM 672 C PHE 41 76.474 72.522 68.165 1.00 0.00 C

ATOM 673 O PHE 41 76.781 72.104 69.280 1.00 0.00 O

ATOM 674 N HID 42 75.543 73.525 67.962 1.00 0.00 N

ATOM 675 H HID 42 75.232 73.406 67.009 1.00 0.00 H

ATOM 676 CA HID 42 74.658 74.153 68.985 1.00 0.00 C

ATOM 677 HA HID 42 75.352 74.423 69.781 1.00 0.00 H

ATOM 678 CB HID 42 74.039 75.404 68.464 1.00 0.00 C

ATOM 679 HB2 HID 42 73.585 75.254 67.485 1.00 0.00 H

ATOM 680 HB3 HID 42 73.373 75.812 69.225 1.00 0.00 H

ATOM 681 CG HID 42 75.034 76.546 68.244 1.00 0.00 C

ATOM 682 ND1 HID 42 75.738 77.315 69.124 1.00 0.00 N

ATOM 683 HD1 HID 42 75.902 77.117 70.100 1.00 0.00 H

ATOM 684 CE1 HID 42 76.463 78.240 68.514 1.00 0.00 C

ATOM 685 HE1 HID 42 77.340 78.720 68.921 1.00 0.00 H

ATOM 686 NE2 HID 42 76.174 78.185 67.196 1.00 0.00 N

ATOM 687 CD2 HID 42 75.344 77.124 67.029 1.00 0.00 C

ATOM 688 HD2 HID 42 74.802 76.853 66.136 1.00 0.00 H

ATOM 689 C HID 42 73.600 73.227 69.709 1.00 0.00 C

ATOM 690 O HID 42 73.545 73.312 70.952 1.00 0.00 O

ATOM 691 N MET 43 72.754 72.531 68.998 1.00 0.00 N

ATOM 692 H MET 43 73.016 72.382 68.034 1.00 0.00 H

ATOM 693 CA MET 43 71.555 71.897 69.560 1.00 0.00 C

ATOM 694 HA MET 43 70.976 72.622 70.133 1.00 0.00 H

ATOM 695 CB MET 43 70.594 71.424 68.410 1.00 0.00 C

ATOM 696 HB2 MET 43 70.221 72.290 67.862 1.00 0.00 H

ATOM 697 HB3 MET 43 71.136 70.800 67.700 1.00 0.00 H

ATOM 698 CG MET 43 69.334 70.747 68.831 1.00 0.00 C

ATOM 699 HG2 MET 43 69.494 69.933 69.538 1.00 0.00 H

ATOM 700 HG3 MET 43 68.656 71.496 69.241 1.00 0.00 H

ATOM 701 SD MET 43 68.314 69.963 67.525 1.00 0.00 S

ATOM 702 CE MET 43 67.300 71.399 67.016 1.00 0.00 C

ATOM 703 HE1 MET 43 67.957 72.221 66.731 1.00 0.00 H

ATOM 704 HE2 MET 43 66.707 71.176 66.129 1.00 0.00 H

ATOM 705 HE3 MET 43 66.673 71.766 67.829 1.00 0.00 H

ATOM 706 C MET 43 71.910 70.727 70.486 1.00 0.00 C

ATOM 707 O MET 43 72.632 69.865 70.093 1.00 0.00 O

ATOM 708 N LYS 44 71.496 70.807 71.764 1.00 0.00 N

ATOM 709 H LYS 44 71.065 71.666 72.073 1.00 0.00 H

ATOM 710 CA LYS 44 72.049 70.034 72.903 1.00 0.00 C

ATOM 711 HA LYS 44 72.399 69.076 72.520 1.00 0.00 H

ATOM 712 CB LYS 44 73.226 70.722 73.590 1.00 0.00 C

ATOM 713 HB2 LYS 44 73.959 70.951 72.817 1.00 0.00 H

ATOM 714 HB3 LYS 44 72.905 71.690 73.974 1.00 0.00 H

ATOM 715 CG LYS 44 73.874 69.870 74.679 1.00 0.00 C

ATOM 716 HG2 LYS 44 73.427 70.061 75.654 1.00 0.00 H

ATOM 717 HG3 LYS 44 73.821 68.849 74.300 1.00 0.00 H

ATOM 718 CD LYS 44 75.366 70.308 74.776 1.00 0.00 C

ATOM 719 HD2 LYS 44 75.964 69.796 74.023 1.00 0.00 H

ATOM 720 HD3 LYS 44 75.479 71.371 74.561 1.00 0.00 H

ATOM 721 CE LYS 44 75.882 70.009 76.185 1.00 0.00 C

ATOM 722 HE2 LYS 44 75.129 70.409 76.863 1.00 0.00 H

ATOM 723 HE3 LYS 44 75.683 68.966 76.433 1.00 0.00 H

ATOM 724 NZ LYS 44 77.302 70.413 76.410 1.00 0.00 N

ATOM 725 HZ1 LYS 44 77.937 69.679 76.131 1.00 0.00 H

ATOM 726 HZ2 LYS 44 77.648 71.131 75.790 1.00 0.00 H

ATOM 727 HZ3 LYS 44 77.463 70.745 77.350 1.00 0.00 H

ATOM 728 C LYS 44 70.835 69.783 73.901 1.00 0.00 C

ATOM 729 O LYS 44 70.398 70.785 74.530 1.00 0.00 O

ATOM 730 N PRO 45 70.439 68.534 74.188 1.00 0.00 N

ATOM 731 CD PRO 45 69.606 68.370 75.344 1.00 0.00 C

ATOM 732 HD2 PRO 45 68.874 69.178 75.354 1.00 0.00 H

ATOM 733 HD3 PRO 45 70.204 68.375 76.255 1.00 0.00 H

ATOM 734 CG PRO 45 68.983 67.014 75.145 1.00 0.00 C

ATOM 735 HG2 PRO 45 68.034 66.948 74.613 1.00 0.00 H

ATOM 736 HG3 PRO 45 68.981 66.462 76.086 1.00 0.00 H

ATOM 737 CB PRO 45 70.100 66.220 74.362 1.00 0.00 C

ATOM 738 HB2 PRO 45 69.809 65.318 73.824 1.00 0.00 H

ATOM 739 HB3 PRO 45 70.886 65.906 75.049 1.00 0.00 H

ATOM 740 CA PRO 45 70.672 67.307 73.437 1.00 0.00 C

ATOM 741 HA PRO 45 71.721 67.242 73.149 1.00 0.00 H

ATOM 742 C PRO 45 69.796 67.283 72.150 1.00 0.00 C

ATOM 743 O PRO 45 69.253 68.364 71.794 1.00 0.00 O

ATOM 744 N TRP 46 69.863 66.238 71.361 1.00 0.00 N

ATOM 745 H TRP 46 70.507 65.476 71.518 1.00 0.00 H

ATOM 746 CA TRP 46 68.944 65.892 70.303 1.00 0.00 C

ATOM 747 HA TRP 46 68.510 66.811 69.908 1.00 0.00 H

ATOM 748 CB TRP 46 69.716 64.923 69.373 1.00 0.00 C

ATOM 749 HB2 TRP 46 70.467 64.400 69.966 1.00 0.00 H

ATOM 750 HB3 TRP 46 68.986 64.231 68.953 1.00 0.00 H

ATOM 751 CG TRP 46 70.525 65.585 68.273 1.00 0.00 C

ATOM 752 CD1 TRP 46 70.863 66.871 68.232 1.00 0.00 C

ATOM 753 HD1 TRP 46 70.622 67.589 69.002 1.00 0.00 H

ATOM 754 NE1 TRP 46 71.386 67.073 66.983 1.00 0.00 N

ATOM 755 HE1 TRP 46 71.647 67.994 66.660 1.00 0.00 H

ATOM 756 CE2 TRP 46 71.562 65.950 66.246 1.00 0.00 C

ATOM 757 CZ2 TRP 46 71.864 65.773 64.913 1.00 0.00 C

ATOM 758 HZ2 TRP 46 72.023 66.658 64.315 1.00 0.00 H

ATOM 759 CH2 TRP 46 72.000 64.485 64.333 1.00 0.00 C

ATOM 760 HH2 TRP 46 72.234 64.349 63.287 1.00 0.00 H

ATOM 761 CZ3 TRP 46 71.589 63.414 65.125 1.00 0.00 C

ATOM 762 HZ3 TRP 46 71.566 62.440 64.658 1.00 0.00 H

ATOM 763 CE3 TRP 46 71.114 63.638 66.436 1.00 0.00 C

ATOM 764 HE3 TRP 46 70.907 62.732 66.986 1.00 0.00 H

ATOM 765 CD2 TRP 46 71.031 64.928 67.069 1.00 0.00 C

ATOM 766 C TRP 46 67.742 65.070 70.840 1.00 0.00 C

ATOM 767 O TRP 46 67.913 64.413 71.821 1.00 0.00 O

ATOM 768 N SER 47 66.555 65.171 70.203 1.00 0.00 N

ATOM 769 H SER 47 66.301 65.914 69.568 1.00 0.00 H

ATOM 770 CA SER 47 65.529 64.132 70.341 1.00 0.00 C

ATOM 771 HA SER 47 65.886 63.336 70.994 1.00 0.00 H

ATOM 772 CB SER 47 64.269 64.840 70.898 1.00 0.00 C

ATOM 773 HB2 SER 47 63.612 64.109 71.370 1.00 0.00 H

ATOM 774 HB3 SER 47 64.412 65.668 71.593 1.00 0.00 H

ATOM 775 OG SER 47 63.481 65.425 69.900 1.00 0.00 O

ATOM 776 HG SER 47 63.181 66.247 70.296 1.00 0.00 H

ATOM 777 C SER 47 65.301 63.457 68.948 1.00 0.00 C

ATOM 778 O SER 47 66.047 63.685 68.010 1.00 0.00 O

ATOM 779 N GLY 48 64.382 62.528 68.996 1.00 0.00 N

ATOM 780 H GLY 48 63.808 62.480 69.826 1.00 0.00 H

ATOM 781 CA GLY 48 63.858 62.011 67.732 1.00 0.00 C

ATOM 782 HA2 GLY 48 64.566 61.326 67.265 1.00 0.00 H

ATOM 783 HA3 GLY 48 63.023 61.346 67.953 1.00 0.00 H

ATOM 784 C GLY 48 63.200 63.030 66.756 1.00 0.00 C

ATOM 785 O GLY 48 63.250 62.934 65.536 1.00 0.00 O

ATOM 786 N ILE 49 62.538 64.058 67.292 1.00 0.00 N

ATOM 787 H ILE 49 62.329 64.120 68.278 1.00 0.00 H

ATOM 788 CA ILE 49 62.134 65.240 66.506 1.00 0.00 C

ATOM 789 HA ILE 49 61.512 64.950 65.659 1.00 0.00 H

ATOM 790 CB ILE 49 61.336 66.350 67.255 1.00 0.00 C

ATOM 791 HB ILE 49 61.823 66.369 68.230 1.00 0.00 H

ATOM 792 CG2 ILE 49 61.172 67.683 66.565 1.00 0.00 C

ATOM 793 HG21 ILE 49 60.459 67.624 65.742 1.00 0.00 H

ATOM 794 HG22 ILE 49 60.771 68.484 67.187 1.00 0.00 H

ATOM 795 HG23 ILE 49 62.145 68.042 66.231 1.00 0.00 H

ATOM 796 CG1 ILE 49 60.015 65.767 67.664 1.00 0.00 C

ATOM 797 HG12 ILE 49 60.299 64.889 68.245 1.00 0.00 H

ATOM 798 HG13 ILE 49 59.491 66.475 68.306 1.00 0.00 H

ATOM 799 CD1 ILE 49 58.987 65.349 66.561 1.00 0.00 C

ATOM 800 HD11 ILE 49 59.346 64.405 66.148 1.00 0.00 H

ATOM 801 HD12 ILE 49 57.959 65.228 66.903 1.00 0.00 H

ATOM 802 HD13 ILE 49 58.801 66.097 65.790 1.00 0.00 H

ATOM 803 C ILE 49 63.314 65.761 65.690 1.00 0.00 C

ATOM 804 O ILE 49 63.285 65.963 64.473 1.00 0.00 O

ATOM 805 N SER 50 64.343 66.024 66.446 1.00 0.00 N

ATOM 806 H SER 50 64.312 65.875 67.444 1.00 0.00 H

ATOM 807 CA SER 50 65.598 66.657 65.979 1.00 0.00 C

ATOM 808 HA SER 50 65.309 67.608 65.530 1.00 0.00 H

ATOM 809 CB SER 50 66.636 66.843 67.081 1.00 0.00 C

ATOM 810 HB2 SER 50 67.244 65.988 67.376 1.00 0.00 H

ATOM 811 HB3 SER 50 67.328 67.580 66.674 1.00 0.00 H

ATOM 812 OG SER 50 65.879 67.311 68.203 1.00 0.00 O

ATOM 813 HG SER 50 66.382 68.094 68.439 1.00 0.00 H

ATOM 814 C SER 50 66.175 65.787 64.833 1.00 0.00 C

ATOM 815 O SER 50 66.515 66.325 63.781 1.00 0.00 O

ATOM 816 N VAL 51 66.052 64.431 64.864 1.00 0.00 N

ATOM 817 H VAL 51 65.736 64.062 65.749 1.00 0.00 H

ATOM 818 CA VAL 51 66.539 63.584 63.757 1.00 0.00 C

ATOM 819 HA VAL 51 67.480 64.054 63.468 1.00 0.00 H

ATOM 820 CB VAL 51 66.860 62.131 64.243 1.00 0.00 C

ATOM 821 HB VAL 51 66.125 61.797 64.974 1.00 0.00 H

ATOM 822 CG1 VAL 51 66.803 61.032 63.203 1.00 0.00 C

ATOM 823 HG11 VAL 51 66.925 60.059 63.679 1.00 0.00 H

ATOM 824 HG12 VAL 51 65.878 61.082 62.629 1.00 0.00 H

ATOM 825 HG13 VAL 51 67.675 61.075 62.550 1.00 0.00 H

ATOM 826 CG2 VAL 51 68.224 62.128 65.027 1.00 0.00 C

ATOM 827 HG21 VAL 51 68.042 62.794 65.871 1.00 0.00 H

ATOM 828 HG22 VAL 51 68.548 61.148 65.375 1.00 0.00 H

ATOM 829 HG23 VAL 51 69.001 62.389 64.308 1.00 0.00 H

ATOM 830 C VAL 51 65.628 63.631 62.558 1.00 0.00 C

ATOM 831 O VAL 51 66.166 63.677 61.461 1.00 0.00 O

ATOM 832 N TYR 52 64.264 63.755 62.696 1.00 0.00 N

ATOM 833 H TYR 52 63.958 63.701 63.657 1.00 0.00 H

ATOM 834 CA TYR 52 63.393 64.093 61.643 1.00 0.00 C

ATOM 835 HA TYR 52 63.535 63.416 60.800 1.00 0.00 H

ATOM 836 CB TYR 52 61.923 63.987 62.067 1.00 0.00 C

ATOM 837 HB2 TYR 52 61.631 64.716 62.822 1.00 0.00 H

ATOM 838 HB3 TYR 52 61.315 64.301 61.218 1.00 0.00 H

ATOM 839 CG TYR 52 61.342 62.614 62.477 1.00 0.00 C

ATOM 840 CD1 TYR 52 61.933 61.468 62.053 1.00 0.00 C

ATOM 841 HD1 TYR 52 62.784 61.509 61.389 1.00 0.00 H

ATOM 842 CE1 TYR 52 61.522 60.226 62.496 1.00 0.00 C

ATOM 843 HE1 TYR 52 61.869 59.303 62.056 1.00 0.00 H

ATOM 844 CZ TYR 52 60.502 60.182 63.422 1.00 0.00 C

ATOM 845 OH TYR 52 60.190 58.971 63.906 1.00 0.00 O

ATOM 846 HH TYR 52 59.419 59.064 64.470 1.00 0.00 H

ATOM 847 CE2 TYR 52 59.859 61.348 63.818 1.00 0.00 C

ATOM 848 HE2 TYR 52 59.104 61.249 64.583 1.00 0.00 H

ATOM 849 CD2 TYR 52 60.252 62.625 63.368 1.00 0.00 C

ATOM 850 HD2 TYR 52 59.824 63.521 63.792 1.00 0.00 H

ATOM 851 C TYR 52 63.732 65.479 60.978 1.00 0.00 C

ATOM 852 O TYR 52 63.870 65.546 59.760 1.00 0.00 O

ATOM 853 N MET 53 63.920 66.577 61.756 1.00 0.00 N

ATOM 854 H MET 53 63.757 66.478 62.747 1.00 0.00 H

ATOM 855 CA MET 53 64.301 67.933 61.350 1.00 0.00 C

ATOM 856 HA MET 53 63.589 68.427 60.689 1.00 0.00 H

ATOM 857 CB MET 53 64.377 68.780 62.603 1.00 0.00 C

ATOM 858 HB2 MET 53 64.390 68.171 63.506 1.00 0.00 H

ATOM 859 HB3 MET 53 65.257 69.423 62.626 1.00 0.00 H

ATOM 860 CG MET 53 63.144 69.748 62.802 1.00 0.00 C

ATOM 861 HG2 MET 53 62.226 69.162 62.781 1.00 0.00 H

ATOM 862 HG3 MET 53 63.188 70.060 63.845 1.00 0.00 H

ATOM 863 SD MET 53 63.041 71.137 61.584 1.00 0.00 S

ATOM 864 CE MET 53 61.714 72.166 62.203 1.00 0.00 C

ATOM 865 HE1 MET 53 60.810 71.561 62.128 1.00 0.00 H

ATOM 866 HE2 MET 53 61.901 72.346 63.262 1.00 0.00 H

ATOM 867 HE3 MET 53 61.707 73.097 61.637 1.00 0.00 H

ATOM 868 C MET 53 65.608 67.985 60.605 1.00 0.00 C

ATOM 869 O MET 53 65.798 68.907 59.791 1.00 0.00 O

ATOM 870 N PHE 54 66.493 67.084 60.953 1.00 0.00 N

ATOM 871 H PHE 54 66.530 66.768 61.912 1.00 0.00 H

ATOM 872 CA PHE 54 67.778 66.911 60.257 1.00 0.00 C

ATOM 873 HA PHE 54 68.112 67.926 60.042 1.00 0.00 H

ATOM 874 CB PHE 54 68.804 66.220 61.055 1.00 0.00 C

ATOM 875 HB2 PHE 54 68.685 66.272 62.137 1.00 0.00 H

ATOM 876 HB3 PHE 54 68.642 65.153 60.900 1.00 0.00 H

ATOM 877 CG PHE 54 70.246 66.591 60.822 1.00 0.00 C

ATOM 878 CD1 PHE 54 70.876 67.284 61.791 1.00 0.00 C

ATOM 879 HD1 PHE 54 70.469 67.620 62.733 1.00 0.00 H

ATOM 880 CE1 PHE 54 72.296 67.556 61.659 1.00 0.00 C

ATOM 881 HE1 PHE 54 72.805 68.123 62.424 1.00 0.00 H

ATOM 882 CZ PHE 54 73.048 66.950 60.680 1.00 0.00 C

ATOM 883 HZ PHE 54 74.096 67.203 60.613 1.00 0.00 H

ATOM 884 CE2 PHE 54 72.402 66.175 59.762 1.00 0.00 C

ATOM 885 HE2 PHE 54 73.007 65.643 59.043 1.00 0.00 H

ATOM 886 CD2 PHE 54 71.014 65.911 59.819 1.00 0.00 C

ATOM 887 HD2 PHE 54 70.442 65.254 59.180 1.00 0.00 H

ATOM 888 C PHE 54 67.558 66.383 58.813 1.00 0.00 C

ATOM 889 O PHE 54 68.200 66.990 57.892 1.00 0.00 O

ATOM 890 N ASN 55 66.818 65.268 58.643 1.00 0.00 N

ATOM 891 H ASN 55 66.592 64.788 59.502 1.00 0.00 H

ATOM 892 CA ASN 55 66.495 64.662 57.291 1.00 0.00 C

ATOM 893 HA ASN 55 67.399 64.554 56.692 1.00 0.00 H

ATOM 894 CB ASN 55 65.988 63.236 57.499 1.00 0.00 C

ATOM 895 HB2 ASN 55 65.134 63.299 58.173 1.00 0.00 H

ATOM 896 HB3 ASN 55 65.507 62.839 56.605 1.00 0.00 H

ATOM 897 CG ASN 55 66.997 62.202 58.045 1.00 0.00 C

ATOM 898 OD1 ASN 55 67.787 61.606 57.319 1.00 0.00 O

ATOM 899 ND2 ASN 55 67.089 62.108 59.337 1.00 0.00 N

ATOM 900 HD21 ASN 55 67.806 61.446 59.596 1.00 0.00 H

ATOM 901 HD22 ASN 55 66.621 62.774 59.935 1.00 0.00 H

ATOM 902 C ASN 55 65.685 65.564 56.399 1.00 0.00 C

ATOM 903 O ASN 55 65.942 65.643 55.209 1.00 0.00 O

ATOM 904 N LEU 56 64.741 66.366 56.938 1.00 0.00 N

ATOM 905 H LEU 56 64.807 66.368 57.945 1.00 0.00 H

ATOM 906 CA LEU 56 64.094 67.534 56.388 1.00 0.00 C

ATOM 907 HA LEU 56 63.700 67.249 55.412 1.00 0.00 H

ATOM 908 CB LEU 56 63.101 68.124 57.386 1.00 0.00 C

ATOM 909 HB2 LEU 56 62.714 67.380 58.082 1.00 0.00 H

ATOM 910 HB3 LEU 56 63.614 68.899 57.956 1.00 0.00 H

ATOM 911 CG LEU 56 61.987 68.956 56.694 1.00 0.00 C

ATOM 912 HG LEU 56 61.719 68.511 55.735 1.00 0.00 H

ATOM 913 CD1 LEU 56 60.842 68.999 57.668 1.00 0.00 C

ATOM 914 HD11 LEU 56 60.340 68.033 57.610 1.00 0.00 H

ATOM 915 HD12 LEU 56 61.093 69.231 58.703 1.00 0.00 H

ATOM 916 HD13 LEU 56 60.111 69.730 57.324 1.00 0.00 H

ATOM 917 CD2 LEU 56 62.409 70.384 56.326 1.00 0.00 C

ATOM 918 HD21 LEU 56 63.210 70.479 55.593 1.00 0.00 H

ATOM 919 HD22 LEU 56 61.544 70.938 55.964 1.00 0.00 H

ATOM 920 HD23 LEU 56 62.663 70.931 57.234 1.00 0.00 H

ATOM 921 C LEU 56 65.153 68.587 55.958 1.00 0.00 C

ATOM 922 O LEU 56 65.170 68.982 54.813 1.00 0.00 O

ATOM 923 N ALA 57 66.071 69.009 56.869 1.00 0.00 N

ATOM 924 H ALA 57 65.963 68.812 57.854 1.00 0.00 H

ATOM 925 CA ALA 57 67.082 70.003 56.529 1.00 0.00 C

ATOM 926 HA ALA 57 66.523 70.842 56.114 1.00 0.00 H

ATOM 927 CB ALA 57 67.863 70.411 57.730 1.00 0.00 C

ATOM 928 HB1 ALA 57 68.419 69.509 57.986 1.00 0.00 H

ATOM 929 HB2 ALA 57 68.572 71.218 57.544 1.00 0.00 H

ATOM 930 HB3 ALA 57 67.217 70.705 58.558 1.00 0.00 H

ATOM 931 C ALA 57 68.041 69.545 55.386 1.00 0.00 C

ATOM 932 O ALA 57 68.521 70.349 54.585 1.00 0.00 O

ATOM 933 N LEU 58 68.411 68.288 55.422 1.00 0.00 N

ATOM 934 H LEU 58 68.127 67.738 56.220 1.00 0.00 H

ATOM 935 CA LEU 58 69.115 67.599 54.299 1.00 0.00 C

ATOM 936 HA LEU 58 70.056 68.119 54.120 1.00 0.00 H

ATOM 937 CB LEU 58 69.470 66.076 54.714 1.00 0.00 C

ATOM 938 HB2 LEU 58 70.002 66.184 55.659 1.00 0.00 H

ATOM 939 HB3 LEU 58 68.623 65.423 54.920 1.00 0.00 H

ATOM 940 CG LEU 58 70.437 65.295 53.762 1.00 0.00 C

ATOM 941 HG LEU 58 71.286 65.913 53.474 1.00 0.00 H

ATOM 942 CD1 LEU 58 71.036 64.082 54.518 1.00 0.00 C

ATOM 943 HD11 LEU 58 70.289 63.379 54.887 1.00 0.00 H

ATOM 944 HD12 LEU 58 71.685 63.601 53.785 1.00 0.00 H

ATOM 945 HD13 LEU 58 71.536 64.459 55.411 1.00 0.00 H

ATOM 946 CD2 LEU 58 69.701 64.781 52.522 1.00 0.00 C

ATOM 947 HD21 LEU 58 70.422 64.229 51.919 1.00 0.00 H

ATOM 948 HD22 LEU 58 68.759 64.267 52.714 1.00 0.00 H

ATOM 949 HD23 LEU 58 69.430 65.629 51.894 1.00 0.00 H

ATOM 950 C LEU 58 68.325 67.675 52.988 1.00 0.00 C

ATOM 951 O LEU 58 68.943 67.891 51.938 1.00 0.00 O

ATOM 952 N ALA 59 66.961 67.397 52.878 1.00 0.00 N

ATOM 953 H ALA 59 66.450 67.702 53.694 1.00 0.00 H

ATOM 954 CA ALA 59 66.302 67.603 51.595 1.00 0.00 C

ATOM 955 HA ALA 59 66.928 67.131 50.838 1.00 0.00 H

ATOM 956 CB ALA 59 65.012 66.725 51.719 1.00 0.00 C

ATOM 957 HB1 ALA 59 65.015 66.007 52.539 1.00 0.00 H

ATOM 958 HB2 ALA 59 64.097 67.297 51.876 1.00 0.00 H

ATOM 959 HB3 ALA 59 64.780 66.240 50.771 1.00 0.00 H

ATOM 960 C ALA 59 66.228 69.058 51.136 1.00 0.00 C

ATOM 961 O ALA 59 66.187 69.358 49.972 1.00 0.00 O

ATOM 962 N ASP 60 66.159 70.021 52.074 1.00 0.00 N

ATOM 963 H ASP 60 65.706 69.844 52.960 1.00 0.00 H

ATOM 964 CA ASP 60 66.202 71.476 51.715 1.00 0.00 C

ATOM 965 HA ASP 60 65.569 71.723 50.863 1.00 0.00 H

ATOM 966 CB ASP 60 65.907 72.250 53.056 1.00 0.00 C

ATOM 967 HB2 ASP 60 65.140 71.768 53.662 1.00 0.00 H

ATOM 968 HB3 ASP 60 66.746 72.402 53.734 1.00 0.00 H

ATOM 969 CG ASP 60 65.249 73.629 52.782 1.00 0.00 C

ATOM 970 OD1 ASP 60 65.959 74.495 52.268 1.00 0.00 O

ATOM 971 OD2 ASP 60 63.996 73.735 52.924 1.00 0.00 O

ATOM 972 C ASP 60 67.606 71.904 51.223 1.00 0.00 C

ATOM 973 O ASP 60 67.694 72.480 50.149 1.00 0.00 O

ATOM 974 N PHE 61 68.696 71.516 52.002 1.00 0.00 N

ATOM 975 H PHE 61 68.471 71.120 52.904 1.00 0.00 H

ATOM 976 CA PHE 61 70.148 71.930 51.745 1.00 0.00 C

ATOM 977 HA PHE 61 70.027 72.995 51.544 1.00 0.00 H

ATOM 978 CB PHE 61 70.924 71.897 53.085 1.00 0.00 C

ATOM 979 HB2 PHE 61 70.314 72.329 53.878 1.00 0.00 H

ATOM 980 HB3 PHE 61 71.089 70.836 53.272 1.00 0.00 H

ATOM 981 CG PHE 61 72.266 72.563 53.177 1.00 0.00 C

ATOM 982 CD1 PHE 61 72.549 73.804 52.586 1.00 0.00 C

ATOM 983 HD1 PHE 61 71.868 74.217 51.856 1.00 0.00 H

ATOM 984 CE1 PHE 61 73.824 74.413 52.784 1.00 0.00 C

ATOM 985 HE1 PHE 61 74.021 75.391 52.370 1.00 0.00 H

ATOM 986 CZ PHE 61 74.725 73.839 53.631 1.00 0.00 C

ATOM 987 HZ PHE 61 75.631 74.425 53.679 1.00 0.00 H

ATOM 988 CE2 PHE 61 74.500 72.530 54.161 1.00 0.00 C

ATOM 989 HE2 PHE 61 75.230 72.007 54.761 1.00 0.00 H

ATOM 990 CD2 PHE 61 73.221 72.002 54.051 1.00 0.00 C

ATOM 991 HD2 PHE 61 72.951 71.136 54.636 1.00 0.00 H

ATOM 992 C PHE 61 70.925 71.227 50.608 1.00 0.00 C

ATOM 993 O PHE 61 71.774 71.898 49.995 1.00 0.00 O

ATOM 994 N LEU 62 70.646 69.984 50.279 1.00 0.00 N

ATOM 995 H LEU 62 70.176 69.397 50.954 1.00 0.00 H

ATOM 996 CA LEU 62 71.278 69.292 49.118 1.00 0.00 C

ATOM 997 HA LEU 62 72.342 69.521 49.064 1.00 0.00 H

ATOM 998 CB LEU 62 71.244 67.789 49.414 1.00 0.00 C

ATOM 999 HB2 LEU 62 71.553 67.430 50.396 1.00 0.00 H

ATOM 1000 HB3 LEU 62 70.187 67.539 49.315 1.00 0.00 H

ATOM 1001 CG LEU 62 71.835 66.823 48.378 1.00 0.00 C

ATOM 1002 HG LEU 62 71.309 67.134 47.476 1.00 0.00 H

ATOM 1003 CD1 LEU 62 73.296 67.014 48.030 1.00 0.00 C

ATOM 1004 HD11 LEU 62 73.325 67.900 47.397 1.00 0.00 H

ATOM 1005 HD12 LEU 62 73.894 67.223 48.917 1.00 0.00 H

ATOM 1006 HD13 LEU 62 73.584 66.198 47.367 1.00 0.00 H

ATOM 1007 CD2 LEU 62 71.525 65.369 48.645 1.00 0.00 C

ATOM 1008 HD21 LEU 62 71.819 65.119 49.664 1.00 0.00 H

ATOM 1009 HD22 LEU 62 70.442 65.271 48.574 1.00 0.00 H

ATOM 1010 HD23 LEU 62 71.990 64.711 47.910 1.00 0.00 H

ATOM 1011 C LEU 62 70.665 69.684 47.763 1.00 0.00 C

ATOM 1012 O LEU 62 71.359 69.634 46.762 1.00 0.00 O

ATOM 1013 N TYR 63 69.434 70.301 47.670 1.00 0.00 N

ATOM 1014 H TYR 63 68.821 70.362 48.470 1.00 0.00 H

ATOM 1015 CA TYR 63 69.022 71.090 46.488 1.00 0.00 C

ATOM 1016 HA TYR 63 69.244 70.491 45.605 1.00 0.00 H

ATOM 1017 CB TYR 63 67.510 71.327 46.511 1.00 0.00 C

ATOM 1018 HB2 TYR 63 66.913 70.424 46.631 1.00 0.00 H

ATOM 1019 HB3 TYR 63 67.267 71.875 47.421 1.00 0.00 H

ATOM 1020 CG TYR 63 66.956 72.114 45.366 1.00 0.00 C

ATOM 1021 CD1 TYR 63 67.299 71.858 44.030 1.00 0.00 C

ATOM 1022 HD1 TYR 63 67.855 70.956 43.819 1.00 0.00 H

ATOM 1023 CE1 TYR 63 66.752 72.585 42.915 1.00 0.00 C

ATOM 1024 HE1 TYR 63 66.801 72.093 41.955 1.00 0.00 H

ATOM 1025 CZ TYR 63 65.692 73.463 43.154 1.00 0.00 C

ATOM 1026 OH TYR 63 65.271 74.258 42.078 1.00 0.00 O

ATOM 1027 HH TYR 63 64.609 74.831 42.471 1.00 0.00 H

ATOM 1028 CE2 TYR 63 65.328 73.785 44.472 1.00 0.00 C

ATOM 1029 HE2 TYR 63 64.684 74.637 44.629 1.00 0.00 H

ATOM 1030 CD2 TYR 63 65.905 73.048 45.526 1.00 0.00 C

ATOM 1031 HD2 TYR 63 65.648 73.301 46.544 1.00 0.00 H

ATOM 1032 C TYR 63 69.785 72.473 46.257 1.00 0.00 C

ATOM 1033 O TYR 63 70.452 72.641 45.248 1.00 0.00 O

ATOM 1034 N VAL 64 69.781 73.391 47.172 1.00 0.00 N

ATOM 1035 H VAL 64 68.981 73.399 47.790 1.00 0.00 H

ATOM 1036 CA VAL 64 70.626 74.658 47.159 1.00 0.00 C

ATOM 1037 HA VAL 64 70.552 75.230 46.234 1.00 0.00 H

ATOM 1038 CB VAL 64 70.293 75.611 48.309 1.00 0.00 C

ATOM 1039 HB VAL 64 71.025 76.410 48.425 1.00 0.00 H

ATOM 1040 CG1 VAL 64 69.058 76.345 47.873 1.00 0.00 C

ATOM 1041 HG11 VAL 64 68.970 77.322 48.350 1.00 0.00 H

ATOM 1042 HG12 VAL 64 69.049 76.670 46.832 1.00 0.00 H

ATOM 1043 HG13 VAL 64 68.214 75.664 47.973 1.00 0.00 H

ATOM 1044 CG2 VAL 64 70.148 75.065 49.681 1.00 0.00 C

ATOM 1045 HG21 VAL 64 70.280 75.923 50.340 1.00 0.00 H

ATOM 1046 HG22 VAL 64 69.117 74.743 49.825 1.00 0.00 H

ATOM 1047 HG23 VAL 64 70.803 74.240 49.960 1.00 0.00 H

ATOM 1048 C VAL 64 72.124 74.292 47.185 1.00 0.00 C

ATOM 1049 O VAL 64 72.891 75.184 46.950 1.00 0.00 O

ATOM 1050 N LEU 65 72.701 73.198 47.712 1.00 0.00 N

ATOM 1051 H LEU 65 72.091 72.559 48.201 1.00 0.00 H

ATOM 1052 CA LEU 65 74.051 72.712 47.268 1.00 0.00 C

ATOM 1053 HA LEU 65 74.779 73.513 47.399 1.00 0.00 H

ATOM 1054 CB LEU 65 74.635 71.548 48.136 1.00 0.00 C

ATOM 1055 HB2 LEU 65 74.413 71.823 49.167 1.00 0.00 H

ATOM 1056 HB3 LEU 65 74.017 70.678 47.916 1.00 0.00 H

ATOM 1057 CG LEU 65 76.109 71.199 47.926 1.00 0.00 C

ATOM 1058 HG LEU 65 76.321 70.916 46.895 1.00 0.00 H

ATOM 1059 CD1 LEU 65 77.148 72.278 48.373 1.00 0.00 C

ATOM 1060 HD11 LEU 65 76.783 73.271 48.111 1.00 0.00 H

ATOM 1061 HD12 LEU 65 77.367 72.235 49.440 1.00 0.00 H

ATOM 1062 HD13 LEU 65 78.051 72.155 47.775 1.00 0.00 H

ATOM 1063 CD2 LEU 65 76.573 69.968 48.611 1.00 0.00 C

ATOM 1064 HD21 LEU 65 75.916 69.203 48.196 1.00 0.00 H

ATOM 1065 HD22 LEU 65 77.569 69.655 48.296 1.00 0.00 H

ATOM 1066 HD23 LEU 65 76.459 70.069 49.690 1.00 0.00 H

ATOM 1067 C LEU 65 74.307 72.443 45.801 1.00 0.00 C

ATOM 1068 O LEU 65 75.416 72.570 45.236 1.00 0.00 O

ATOM 1069 N THR 66 73.241 71.896 45.169 1.00 0.00 N

ATOM 1070 H THR 66 72.341 71.783 45.613 1.00 0.00 H

ATOM 1071 CA THR 66 73.314 71.518 43.708 1.00 0.00 C

ATOM 1072 HA THR 66 74.351 71.579 43.378 1.00 0.00 H

ATOM 1073 CB THR 66 72.780 70.063 43.475 1.00 0.00 C

ATOM 1074 HB THR 66 72.503 69.982 42.424 1.00 0.00 H

ATOM 1075 CG2 THR 66 73.716 68.961 43.934 1.00 0.00 C

ATOM 1076 HG21 THR 66 74.564 68.893 43.252 1.00 0.00 H

ATOM 1077 HG22 THR 66 74.079 69.113 44.950 1.00 0.00 H

ATOM 1078 HG23 THR 66 73.284 67.961 43.960 1.00 0.00 H

ATOM 1079 OG1 THR 66 71.575 69.869 44.145 1.00 0.00 O

ATOM 1080 HG1 THR 66 71.751 69.674 45.068 1.00 0.00 H

ATOM 1081 C THR 66 72.867 72.585 42.631 1.00 0.00 C

ATOM 1082 O THR 66 73.442 72.706 41.577 1.00 0.00 O

ATOM 1083 N LEU 67 72.155 73.585 43.194 1.00 0.00 N

ATOM 1084 H LEU 67 71.849 73.443 44.146 1.00 0.00 H

ATOM 1085 CA LEU 67 71.768 74.910 42.547 1.00 0.00 C

ATOM 1086 HA LEU 67 71.044 74.666 41.770 1.00 0.00 H

ATOM 1087 CB LEU 67 71.017 75.905 43.549 1.00 0.00 C

ATOM 1088 HB2 LEU 67 71.287 75.535 44.538 1.00 0.00 H

ATOM 1089 HB3 LEU 67 71.573 76.837 43.456 1.00 0.00 H

ATOM 1090 CG LEU 67 69.504 76.153 43.348 1.00 0.00 C

ATOM 1091 HG LEU 67 69.273 76.218 42.285 1.00 0.00 H

ATOM 1092 CD1 LEU 67 68.594 75.041 43.945 1.00 0.00 C

ATOM 1093 HD11 LEU 67 68.453 75.202 45.014 1.00 0.00 H

ATOM 1094 HD12 LEU 67 67.605 74.979 43.493 1.00 0.00 H

ATOM 1095 HD13 LEU 67 69.013 74.054 43.750 1.00 0.00 H

ATOM 1096 CD2 LEU 67 69.115 77.486 43.992 1.00 0.00 C

ATOM 1097 HD21 LEU 67 69.665 77.730 44.901 1.00 0.00 H

ATOM 1098 HD22 LEU 67 69.165 78.300 43.269 1.00 0.00 H

ATOM 1099 HD23 LEU 67 68.052 77.504 44.232 1.00 0.00 H

ATOM 1100 C LEU 67 72.911 75.646 41.899 1.00 0.00 C

ATOM 1101 O LEU 67 72.609 76.210 40.840 1.00 0.00 O

ATOM 1102 N PRO 68 74.185 75.711 42.408 1.00 0.00 N

ATOM 1103 CD PRO 68 74.624 75.652 43.848 1.00 0.00 C

ATOM 1104 HD2 PRO 68 74.195 74.787 44.354 1.00 0.00 H

ATOM 1105 HD3 PRO 68 74.323 76.511 44.446 1.00 0.00 H

ATOM 1106 CG PRO 68 76.156 75.708 43.815 1.00 0.00 C

ATOM 1107 HG2 PRO 68 76.714 75.108 44.535 1.00 0.00 H

ATOM 1108 HG3 PRO 68 76.449 76.751 43.937 1.00 0.00 H

ATOM 1109 CB PRO 68 76.413 75.205 42.315 1.00 0.00 C

ATOM 1110 HB2 PRO 68 76.266 74.130 42.212 1.00 0.00 H

ATOM 1111 HB3 PRO 68 77.389 75.530 41.957 1.00 0.00 H

ATOM 1112 CA PRO 68 75.330 75.935 41.564 1.00 0.00 C

ATOM 1113 HA PRO 68 75.521 77.008 41.532 1.00 0.00 H

ATOM 1114 C PRO 68 75.194 75.421 40.084 1.00 0.00 C

ATOM 1115 O PRO 68 75.276 76.188 39.149 1.00 0.00 O

ATOM 1116 N ALA 69 74.981 74.087 39.805 1.00 0.00 N

ATOM 1117 H ALA 69 74.835 73.478 40.597 1.00 0.00 H

ATOM 1118 CA ALA 69 74.773 73.571 38.423 1.00 0.00 C

ATOM 1119 HA ALA 69 75.603 73.902 37.799 1.00 0.00 H

ATOM 1120 CB ALA 69 74.671 72.031 38.457 1.00 0.00 C

ATOM 1121 HB1 ALA 69 73.890 71.650 37.800 1.00 0.00 H

ATOM 1122 HB2 ALA 69 75.561 71.743 37.896 1.00 0.00 H

ATOM 1123 HB3 ALA 69 74.613 71.654 39.478 1.00 0.00 H

ATOM 1124 C ALA 69 73.534 74.167 37.640 1.00 0.00 C

ATOM 1125 O ALA 69 73.642 74.360 36.432 1.00 0.00 O

ATOM 1126 N LEU 70 72.486 74.582 38.280 1.00 0.00 N

ATOM 1127 H LEU 70 72.737 74.819 39.229 1.00 0.00 H

ATOM 1128 CA LEU 70 71.301 75.193 37.740 1.00 0.00 C

ATOM 1129 HA LEU 70 71.036 74.635 36.842 1.00 0.00 H

ATOM 1130 CB LEU 70 70.150 75.130 38.786 1.00 0.00 C

ATOM 1131 HB2 LEU 70 70.598 75.015 39.773 1.00 0.00 H

ATOM 1132 HB3 LEU 70 69.687 76.115 38.734 1.00 0.00 H

ATOM 1133 CG LEU 70 69.032 74.143 38.344 1.00 0.00 C

ATOM 1134 HG LEU 70 69.591 73.229 38.139 1.00 0.00 H

ATOM 1135 CD1 LEU 70 68.255 73.814 39.579 1.00 0.00 C

ATOM 1136 HD11 LEU 70 68.955 73.525 40.363 1.00 0.00 H

ATOM 1137 HD12 LEU 70 67.735 74.716 39.902 1.00 0.00 H

ATOM 1138 HD13 LEU 70 67.550 73.022 39.325 1.00 0.00 H

ATOM 1139 CD2 LEU 70 68.108 74.511 37.156 1.00 0.00 C

ATOM 1140 HD21 LEU 70 67.397 73.686 37.119 1.00 0.00 H

ATOM 1141 HD22 LEU 70 67.666 75.486 37.363 1.00 0.00 H

ATOM 1142 HD23 LEU 70 68.645 74.591 36.211 1.00 0.00 H

ATOM 1143 C LEU 70 71.518 76.699 37.343 1.00 0.00 C

ATOM 1144 O LEU 70 71.004 77.200 36.327 1.00 0.00 O

ATOM 1145 N ILE 71 72.080 77.429 38.279 1.00 0.00 N

ATOM 1146 H ILE 71 72.385 76.939 39.108 1.00 0.00 H

ATOM 1147 CA ILE 71 72.854 78.717 37.962 1.00 0.00 C

ATOM 1148 HA ILE 71 72.068 79.427 37.705 1.00 0.00 H

ATOM 1149 CB ILE 71 73.721 79.255 39.177 1.00 0.00 C

ATOM 1150 HB ILE 71 74.335 78.387 39.419 1.00 0.00 H

ATOM 1151 CG2 ILE 71 74.624 80.394 38.755 1.00 0.00 C

ATOM 1152 HG21 ILE 71 75.301 80.120 37.945 1.00 0.00 H

ATOM 1153 HG22 ILE 71 73.916 81.172 38.468 1.00 0.00 H

ATOM 1154 HG23 ILE 71 75.120 80.803 39.635 1.00 0.00 H

ATOM 1155 CG1 ILE 71 72.792 79.653 40.400 1.00 0.00 C

ATOM 1156 HG12 ILE 71 72.248 80.519 40.023 1.00 0.00 H

ATOM 1157 HG13 ILE 71 72.116 78.823 40.607 1.00 0.00 H

ATOM 1158 CD1 ILE 71 73.529 79.867 41.771 1.00 0.00 C

ATOM 1159 HD11 ILE 71 73.929 78.900 42.079 1.00 0.00 H

ATOM 1160 HD12 ILE 71 74.355 80.575 41.714 1.00 0.00 H

ATOM 1161 HD13 ILE 71 72.674 80.144 42.387 1.00 0.00 H

ATOM 1162 C ILE 71 73.497 78.532 36.572 1.00 0.00 C

ATOM 1163 O ILE 71 73.036 79.236 35.624 1.00 0.00 O

ATOM 1164 N PHE 72 74.468 77.706 36.453 1.00 0.00 N

ATOM 1165 H PHE 72 74.726 77.376 37.372 1.00 0.00 H

ATOM 1166 CA PHE 72 75.334 77.518 35.288 1.00 0.00 C

ATOM 1167 HA PHE 72 75.843 78.438 34.998 1.00 0.00 H

ATOM 1168 CB PHE 72 76.338 76.519 35.763 1.00 0.00 C

ATOM 1169 HB2 PHE 72 76.122 76.043 36.719 1.00 0.00 H

ATOM 1170 HB3 PHE 72 76.581 75.748 35.032 1.00 0.00 H

ATOM 1171 CG PHE 72 77.676 77.229 35.839 1.00 0.00 C

ATOM 1172 CD1 PHE 72 78.445 77.486 34.663 1.00 0.00 C

ATOM 1173 HD1 PHE 72 77.930 77.453 33.715 1.00 0.00 H

ATOM 1174 CE1 PHE 72 79.739 77.931 34.770 1.00 0.00 C

ATOM 1175 HE1 PHE 72 80.346 78.004 33.880 1.00 0.00 H

ATOM 1176 CZ PHE 72 80.361 78.076 36.023 1.00 0.00 C

ATOM 1177 HZ PHE 72 81.364 78.468 36.097 1.00 0.00 H

ATOM 1178 CE2 PHE 72 79.655 77.852 37.214 1.00 0.00 C

ATOM 1179 HE2 PHE 72 80.093 77.929 38.198 1.00 0.00 H

ATOM 1180 CD2 PHE 72 78.347 77.393 37.119 1.00 0.00 C

ATOM 1181 HD2 PHE 72 77.797 77.195 38.028 1.00 0.00 H

ATOM 1182 C PHE 72 74.593 77.031 34.030 1.00 0.00 C

ATOM 1183 O PHE 72 74.866 77.342 32.936 1.00 0.00 O

ATOM 1184 N TYR 73 73.443 76.395 34.250 1.00 0.00 N

ATOM 1185 H TYR 73 73.136 75.973 35.115 1.00 0.00 H

ATOM 1186 CA TYR 73 72.637 75.892 33.119 1.00 0.00 C

ATOM 1187 HA TYR 73 73.249 75.501 32.307 1.00 0.00 H

ATOM 1188 CB TYR 73 71.643 74.779 33.629 1.00 0.00 C

ATOM 1189 HB2 TYR 73 72.145 73.858 33.926 1.00 0.00 H

ATOM 1190 HB3 TYR 73 71.066 75.093 34.499 1.00 0.00 H

ATOM 1191 CG TYR 73 70.615 74.300 32.626 1.00 0.00 C

ATOM 1192 CD1 TYR 73 70.990 73.487 31.530 1.00 0.00 C

ATOM 1193 HD1 TYR 73 72.034 73.356 31.287 1.00 0.00 H

ATOM 1194 CE1 TYR 73 69.944 72.947 30.762 1.00 0.00 C

ATOM 1195 HE1 TYR 73 70.200 72.215 30.010 1.00 0.00 H

ATOM 1196 CZ TYR 73 68.562 73.203 31.023 1.00 0.00 C

ATOM 1197 OH TYR 73 67.578 72.748 30.131 1.00 0.00 O

ATOM 1198 HH TYR 73 68.001 72.094 29.569 1.00 0.00 H

ATOM 1199 CE2 TYR 73 68.188 74.083 32.027 1.00 0.00 C

ATOM 1200 HE2 TYR 73 67.154 74.389 32.069 1.00 0.00 H

ATOM 1201 CD2 TYR 73 69.224 74.647 32.845 1.00 0.00 C

ATOM 1202 HD2 TYR 73 68.965 75.361 33.612 1.00 0.00 H

ATOM 1203 C TYR 73 71.757 77.024 32.532 1.00 0.00 C

ATOM 1204 O TYR 73 71.856 77.315 31.317 1.00 0.00 O

ATOM 1205 N TYR 74 70.999 77.837 33.345 1.00 0.00 N

ATOM 1206 H TYR 74 70.822 77.426 34.250 1.00 0.00 H

ATOM 1207 CA TYR 74 70.316 79.003 32.868 1.00 0.00 C

ATOM 1208 HA TYR 74 69.828 78.755 31.926 1.00 0.00 H

ATOM 1209 CB TYR 74 69.159 79.480 33.828 1.00 0.00 C

ATOM 1210 HB2 TYR 74 69.572 79.592 34.831 1.00 0.00 H

ATOM 1211 HB3 TYR 74 68.863 80.489 33.542 1.00 0.00 H

ATOM 1212 CG TYR 74 67.970 78.566 33.829 1.00 0.00 C

ATOM 1213 CD1 TYR 74 67.783 77.580 34.756 1.00 0.00 C

ATOM 1214 HD1 TYR 74 68.305 77.654 35.699 1.00 0.00 H

ATOM 1215 CE1 TYR 74 66.776 76.610 34.621 1.00 0.00 C

ATOM 1216 HE1 TYR 74 66.776 75.732 35.250 1.00 0.00 H

ATOM 1217 CZ TYR 74 65.863 76.712 33.572 1.00 0.00 C

ATOM 1218 OH TYR 74 64.969 75.715 33.352 1.00 0.00 O

ATOM 1219 HH TYR 74 65.467 74.930 33.591 1.00 0.00 H

ATOM 1220 CE2 TYR 74 66.077 77.742 32.605 1.00 0.00 C

ATOM 1221 HE2 TYR 74 65.375 77.835 31.790 1.00 0.00 H

ATOM 1222 CD2 TYR 74 67.159 78.621 32.702 1.00 0.00 C

ATOM 1223 HD2 TYR 74 67.239 79.303 31.868 1.00 0.00 H

ATOM 1224 C TYR 74 71.238 80.108 32.451 1.00 0.00 C

ATOM 1225 O TYR 74 70.866 80.794 31.503 1.00 0.00 O

ATOM 1226 N PHE 75 72.467 80.225 33.019 1.00 0.00 N

ATOM 1227 H PHE 75 72.708 79.592 33.768 1.00 0.00 H

ATOM 1228 CA PHE 75 73.531 81.129 32.374 1.00 0.00 C

ATOM 1229 HA PHE 75 73.142 82.140 32.252 1.00 0.00 H

ATOM 1230 CB PHE 75 74.806 81.244 33.262 1.00 0.00 C

ATOM 1231 HB2 PHE 75 74.622 80.476 34.014 1.00 0.00 H

ATOM 1232 HB3 PHE 75 75.665 80.932 32.669 1.00 0.00 H

ATOM 1233 CG PHE 75 75.137 82.638 33.848 1.00 0.00 C

ATOM 1234 CD1 PHE 75 76.070 83.490 33.274 1.00 0.00 C

ATOM 1235 HD1 PHE 75 76.667 83.138 32.446 1.00 0.00 H

ATOM 1236 CE1 PHE 75 76.105 84.901 33.665 1.00 0.00 C

ATOM 1237 HE1 PHE 75 76.800 85.669 33.358 1.00 0.00 H

ATOM 1238 CZ PHE 75 75.276 85.305 34.765 1.00 0.00 C

ATOM 1239 HZ PHE 75 75.284 86.324 35.123 1.00 0.00 H

ATOM 1240 CE2 PHE 75 74.461 84.366 35.476 1.00 0.00 C

ATOM 1241 HE2 PHE 75 73.916 84.675 36.356 1.00 0.00 H

ATOM 1242 CD2 PHE 75 74.337 83.060 34.913 1.00 0.00 C

ATOM 1243 HD2 PHE 75 73.687 82.316 35.348 1.00 0.00 H

ATOM 1244 C PHE 75 73.891 80.794 30.883 1.00 0.00 C

ATOM 1245 O PHE 75 73.912 81.633 30.015 1.00 0.00 O

ATOM 1246 N ASN 76 74.236 79.529 30.574 1.00 0.00 N

ATOM 1247 H ASN 76 74.294 78.802 31.273 1.00 0.00 H

ATOM 1248 CA ASN 76 74.721 79.147 29.207 1.00 0.00 C

ATOM 1249 HA ASN 76 75.424 79.921 28.898 1.00 0.00 H

ATOM 1250 CB ASN 76 75.455 77.846 29.495 1.00 0.00 C

ATOM 1251 HB2 ASN 76 76.094 77.976 30.369 1.00 0.00 H

ATOM 1252 HB3 ASN 76 74.716 77.062 29.657 1.00 0.00 H

ATOM 1253 CG ASN 76 76.383 77.378 28.362 1.00 0.00 C

ATOM 1254 OD1 ASN 76 76.543 77.970 27.271 1.00 0.00 O

ATOM 1255 ND2 ASN 76 77.059 76.264 28.699 1.00 0.00 N

ATOM 1256 HD21 ASN 76 77.552 75.811 27.942 1.00 0.00 H

ATOM 1257 HD22 ASN 76 76.812 75.779 29.550 1.00 0.00 H

ATOM 1258 C ASN 76 73.612 78.852 28.210 1.00 0.00 C

ATOM 1259 O ASN 76 73.692 78.022 27.300 1.00 0.00 O

ATOM 1260 N LYS 77 72.509 79.630 28.346 1.00 0.00 N

ATOM 1261 H LYS 77 72.556 80.253 29.140 1.00 0.00 H

ATOM 1262 CA LYS 77 71.314 79.534 27.563 1.00 0.00 C

ATOM 1263 HA LYS 77 70.608 80.030 28.229 1.00 0.00 H

ATOM 1264 CB LYS 77 71.447 80.276 26.178 1.00 0.00 C

ATOM 1265 HB2 LYS 77 72.007 79.561 25.575 1.00 0.00 H

ATOM 1266 HB3 LYS 77 70.411 80.484 25.910 1.00 0.00 H

ATOM 1267 CG LYS 77 72.343 81.493 26.133 1.00 0.00 C

ATOM 1268 HG2 LYS 77 72.016 82.251 26.845 1.00 0.00 H

ATOM 1269 HG3 LYS 77 73.319 81.306 26.581 1.00 0.00 H

ATOM 1270 CD LYS 77 72.615 82.137 24.801 1.00 0.00 C

ATOM 1271 HD2 LYS 77 72.583 81.330 24.070 1.00 0.00 H

ATOM 1272 HD3 LYS 77 71.657 82.577 24.522 1.00 0.00 H

ATOM 1273 CE LYS 77 73.712 83.130 24.539 1.00 0.00 C

ATOM 1274 HE2 LYS 77 73.793 83.200 23.455 1.00 0.00 H

ATOM 1275 HE3 LYS 77 73.351 84.085 24.923 1.00 0.00 H

ATOM 1276 NZ LYS 77 74.965 82.730 25.250 1.00 0.00 N

ATOM 1277 HZ1 LYS 77 74.966 82.922 26.242 1.00 0.00 H

ATOM 1278 HZ2 LYS 77 75.157 81.742 25.170 1.00 0.00 H

ATOM 1279 HZ3 LYS 77 75.741 83.178 24.784 1.00 0.00 H

ATOM 1280 C LYS 77 70.974 78.108 27.384 1.00 0.00 C

ATOM 1281 O LYS 77 70.629 77.721 26.268 1.00 0.00 O

ATOM 1282 N THR 78 70.855 77.366 28.544 1.00 0.00 N

ATOM 1283 H THR 78 70.958 77.933 29.373 1.00 0.00 H

ATOM 1284 CA THR 78 70.216 76.055 28.730 1.00 0.00 C

ATOM 1285 HA THR 78 70.532 75.625 29.681 1.00 0.00 H

ATOM 1286 CB THR 78 68.666 75.982 28.871 1.00 0.00 C

ATOM 1287 HB THR 78 68.253 74.975 28.909 1.00 0.00 H

ATOM 1288 CG2 THR 78 68.283 76.818 30.161 1.00 0.00 C

ATOM 1289 HG21 THR 78 68.862 76.287 30.917 1.00 0.00 H

ATOM 1290 HG22 THR 78 68.679 77.831 30.098 1.00 0.00 H

ATOM 1291 HG23 THR 78 67.200 76.940 30.169 1.00 0.00 H

ATOM 1292 OG1 THR 78 68.100 76.718 27.839 1.00 0.00 O

ATOM 1293 HG1 THR 78 67.377 76.204 27.472 1.00 0.00 H

ATOM 1294 C THR 78 70.672 75.009 27.648 1.00 0.00 C

ATOM 1295 O THR 78 69.975 74.044 27.357 1.00 0.00 O

ATOM 1296 N ASP 79 71.979 75.120 27.275 1.00 0.00 N

ATOM 1297 H ASP 79 72.564 75.768 27.782 1.00 0.00 H

ATOM 1298 CA ASP 79 72.776 73.941 26.843 1.00 0.00 C

ATOM 1299 HA ASP 79 72.151 73.389 26.142 1.00 0.00 H

ATOM 1300 CB ASP 79 73.970 74.486 26.098 1.00 0.00 C

ATOM 1301 HB2 ASP 79 73.537 75.198 25.395 1.00 0.00 H

ATOM 1302 HB3 ASP 79 74.530 75.085 26.816 1.00 0.00 H

ATOM 1303 CG ASP 79 74.875 73.491 25.392 1.00 0.00 C

ATOM 1304 OD1 ASP 79 75.933 73.822 24.806 1.00 0.00 O

ATOM 1305 OD2 ASP 79 74.542 72.266 25.348 1.00 0.00 O

ATOM 1306 C ASP 79 73.202 73.130 28.151 1.00 0.00 C

ATOM 1307 O ASP 79 73.725 73.774 29.119 1.00 0.00 O

ATOM 1308 N TRP 80 73.035 71.740 28.210 1.00 0.00 N

ATOM 1309 H TRP 80 72.852 71.353 27.295 1.00 0.00 H

ATOM 1310 CA TRP 80 73.616 70.932 29.289 1.00 0.00 C

ATOM 1311 HA TRP 80 73.677 71.549 30.185 1.00 0.00 H

ATOM 1312 CB TRP 80 72.696 69.773 29.682 1.00 0.00 C

ATOM 1313 HB2 TRP 80 71.671 70.134 29.771 1.00 0.00 H

ATOM 1314 HB3 TRP 80 72.657 69.008 28.907 1.00 0.00 H

ATOM 1315 CG TRP 80 73.111 68.945 30.918 1.00 0.00 C

ATOM 1316 CD1 TRP 80 73.310 67.589 31.011 1.00 0.00 C

ATOM 1317 HD1 TRP 80 73.215 66.874 30.208 1.00 0.00 H

ATOM 1318 NE1 TRP 80 73.568 67.288 32.305 1.00 0.00 N

ATOM 1319 HE1 TRP 80 73.866 66.399 32.680 1.00 0.00 H

ATOM 1320 CE2 TRP 80 73.444 68.399 33.098 1.00 0.00 C

ATOM 1321 CZ2 TRP 80 73.386 68.425 34.534 1.00 0.00 C

ATOM 1322 HZ2 TRP 80 73.894 67.645 35.083 1.00 0.00 H

ATOM 1323 CH2 TRP 80 73.137 69.636 35.119 1.00 0.00 C

ATOM 1324 HH2 TRP 80 73.448 69.727 36.149 1.00 0.00 H

ATOM 1325 CZ3 TRP 80 72.562 70.721 34.422 1.00 0.00 C

ATOM 1326 HZ3 TRP 80 72.061 71.522 34.945 1.00 0.00 H

ATOM 1327 CE3 TRP 80 72.530 70.570 33.008 1.00 0.00 C

ATOM 1328 HE3 TRP 80 72.288 71.394 32.352 1.00 0.00 H

ATOM 1329 CD2 TRP 80 73.006 69.437 32.304 1.00 0.00 C

ATOM 1330 C TRP 80 75.022 70.406 29.081 1.00 0.00 C

ATOM 1331 O TRP 80 75.233 69.581 28.177 1.00 0.00 O

ATOM 1332 N ILE 81 76.022 70.916 29.777 1.00 0.00 N

ATOM 1333 H ILE 81 75.779 71.489 30.573 1.00 0.00 H

ATOM 1334 CA ILE 81 77.483 70.591 29.647 1.00 0.00 C

ATOM 1335 HA ILE 81 77.581 70.226 28.625 1.00 0.00 H

ATOM 1336 CB ILE 81 78.380 71.763 30.009 1.00 0.00 C

ATOM 1337 HB ILE 81 79.420 71.495 29.820 1.00 0.00 H

ATOM 1338 CG2 ILE 81 78.044 73.004 29.142 1.00 0.00 C

ATOM 1339 HG21 ILE 81 77.854 72.604 28.146 1.00 0.00 H

ATOM 1340 HG22 ILE 81 77.118 73.521 29.393 1.00 0.00 H

ATOM 1341 HG23 ILE 81 78.829 73.729 28.923 1.00 0.00 H

ATOM 1342 CG1 ILE 81 78.360 72.071 31.554 1.00 0.00 C

ATOM 1343 HG12 ILE 81 77.338 72.163 31.920 1.00 0.00 H

ATOM 1344 HG13 ILE 81 78.667 71.217 32.157 1.00 0.00 H

ATOM 1345 CD1 ILE 81 79.159 73.317 31.960 1.00 0.00 C

ATOM 1346 HD11 ILE 81 79.965 73.425 31.234 1.00 0.00 H

ATOM 1347 HD12 ILE 81 78.506 74.189 31.924 1.00 0.00 H

ATOM 1348 HD13 ILE 81 79.631 73.081 32.914 1.00 0.00 H

ATOM 1349 C ILE 81 77.872 69.329 30.396 1.00 0.00 C

ATOM 1350 O ILE 81 78.919 68.842 30.056 1.00 0.00 O

ATOM 1351 N PHE 82 77.026 68.743 31.381 1.00 0.00 N

ATOM 1352 H PHE 82 76.105 69.152 31.446 1.00 0.00 H

ATOM 1353 CA PHE 82 77.465 67.920 32.552 1.00 0.00 C

ATOM 1354 HA PHE 82 78.546 68.027 32.641 1.00 0.00 H

ATOM 1355 CB PHE 82 77.009 68.420 33.989 1.00 0.00 C

ATOM 1356 HB2 PHE 82 75.936 68.229 34.019 1.00 0.00 H

ATOM 1357 HB3 PHE 82 77.457 67.969 34.875 1.00 0.00 H

ATOM 1358 CG PHE 82 77.194 69.933 34.244 1.00 0.00 C

ATOM 1359 CD1 PHE 82 76.018 70.765 34.331 1.00 0.00 C

ATOM 1360 HD1 PHE 82 75.065 70.375 34.004 1.00 0.00 H

ATOM 1361 CE1 PHE 82 76.220 72.094 34.741 1.00 0.00 C

ATOM 1362 HE1 PHE 82 75.398 72.795 34.742 1.00 0.00 H

ATOM 1363 CZ PHE 82 77.486 72.541 35.045 1.00 0.00 C

ATOM 1364 HZ PHE 82 77.593 73.576 35.336 1.00 0.00 H

ATOM 1365 CE2 PHE 82 78.566 71.731 35.170 1.00 0.00 C

ATOM 1366 HE2 PHE 82 79.479 72.171 35.543 1.00 0.00 H

ATOM 1367 CD2 PHE 82 78.390 70.383 34.742 1.00 0.00 C

ATOM 1368 HD2 PHE 82 79.224 69.759 34.455 1.00 0.00 H

ATOM 1369 C PHE 82 77.232 66.418 32.370 1.00 0.00 C

ATOM 1370 O PHE 82 77.633 65.642 33.232 1.00 0.00 O

ATOM 1371 N GLY 83 76.521 65.934 31.352 1.00 0.00 N

ATOM 1372 H GLY 83 76.136 66.588 30.685 1.00 0.00 H

ATOM 1373 CA GLY 83 76.214 64.496 31.122 1.00 0.00 C

ATOM 1374 HA2 GLY 83 76.098 64.403 30.042 1.00 0.00 H

ATOM 1375 HA3 GLY 83 77.080 63.852 31.273 1.00 0.00 H

ATOM 1376 C GLY 83 74.914 63.908 31.696 1.00 0.00 C

ATOM 1377 O GLY 83 73.969 64.587 32.168 1.00 0.00 O

ATOM 1378 N ASP 84 74.663 62.695 31.338 1.00 0.00 N

ATOM 1379 H ASP 84 75.338 62.092 30.891 1.00 0.00 H

ATOM 1380 CA ASP 84 73.472 61.864 31.539 1.00 0.00 C

ATOM 1381 HA ASP 84 72.591 62.417 31.213 1.00 0.00 H

ATOM 1382 CB ASP 84 73.528 60.545 30.697 1.00 0.00 C

ATOM 1383 HB2 ASP 84 73.723 60.783 29.651 1.00 0.00 H

ATOM 1384 HB3 ASP 84 74.363 59.973 31.101 1.00 0.00 H

ATOM 1385 CG ASP 84 72.148 59.782 30.771 1.00 0.00 C

ATOM 1386 OD1 ASP 84 71.902 59.088 31.804 1.00 0.00 O

ATOM 1387 OD2 ASP 84 71.210 60.111 29.940 1.00 0.00 O

ATOM 1388 C ASP 84 73.244 61.602 33.034 1.00 0.00 C

ATOM 1389 O ASP 84 72.202 61.862 33.640 1.00 0.00 O

ATOM 1390 N ALA 85 74.339 61.173 33.636 1.00 0.00 N

ATOM 1391 H ALA 85 75.204 61.076 33.123 1.00 0.00 H

ATOM 1392 CA ALA 85 74.161 60.832 35.012 1.00 0.00 C

ATOM 1393 HA ALA 85 73.195 60.341 35.132 1.00 0.00 H

ATOM 1394 CB ALA 85 75.289 59.816 35.445 1.00 0.00 C

ATOM 1395 HB1 ALA 85 75.437 59.058 34.676 1.00 0.00 H

ATOM 1396 HB2 ALA 85 76.185 60.395 35.668 1.00 0.00 H

ATOM 1397 HB3 ALA 85 75.021 59.215 36.314 1.00 0.00 H

ATOM 1398 C ALA 85 74.111 61.949 36.032 1.00 0.00 C

ATOM 1399 O ALA 85 73.348 61.905 36.991 1.00 0.00 O

ATOM 1400 N MET 86 74.663 63.117 35.721 1.00 0.00 N

ATOM 1401 H MET 86 75.265 63.186 34.913 1.00 0.00 H

ATOM 1402 CA MET 86 74.607 64.359 36.579 1.00 0.00 C

ATOM 1403 HA MET 86 74.661 64.049 37.623 1.00 0.00 H

ATOM 1404 CB MET 86 75.803 65.283 36.275 1.00 0.00 C

ATOM 1405 HB2 MET 86 76.699 64.662 36.277 1.00 0.00 H

ATOM 1406 HB3 MET 86 75.671 65.781 35.314 1.00 0.00 H

ATOM 1407 CG MET 86 75.941 66.545 37.236 1.00 0.00 C

ATOM 1408 HG2 MET 86 76.773 67.191 36.958 1.00 0.00 H

ATOM 1409 HG3 MET 86 75.080 67.213 37.252 1.00 0.00 H

ATOM 1410 SD MET 86 76.114 66.186 39.047 1.00 0.00 S

ATOM 1411 CE MET 86 74.855 67.068 39.887 1.00 0.00 C

ATOM 1412 HE1 MET 86 74.827 68.119 39.597 1.00 0.00 H

ATOM 1413 HE2 MET 86 73.838 66.790 39.607 1.00 0.00 H

ATOM 1414 HE3 MET 86 74.895 66.898 40.963 1.00 0.00 H

ATOM 1415 C MET 86 73.220 65.104 36.422 1.00 0.00 C

ATOM 1416 O MET 86 72.669 65.408 37.447 1.00 0.00 O

ATOM 1417 N CYX 87 72.649 65.124 35.260 1.00 0.00 N

ATOM 1418 H CYX 87 73.179 64.767 34.477 1.00 0.00 H

ATOM 1419 CA CYX 87 71.280 65.470 35.159 1.00 0.00 C

ATOM 1420 HA CYX 87 71.134 66.483 35.533 1.00 0.00 H

ATOM 1421 CB CYX 87 70.939 65.392 33.662 1.00 0.00 C

ATOM 1422 HB2 CYX 87 71.554 66.108 33.118 1.00 0.00 H

ATOM 1423 HB3 CYX 87 71.105 64.372 33.314 1.00 0.00 H

ATOM 1424 SG CYX 87 69.176 65.730 33.332 1.00 0.00 S

ATOM 1425 C CYX 87 70.311 64.543 35.907 1.00 0.00 C

ATOM 1426 O CYX 87 69.389 65.036 36.599 1.00 0.00 O

ATOM 1427 N LYS 88 70.575 63.237 35.943 1.00 0.00 N

ATOM 1428 H LYS 88 71.312 62.896 35.342 1.00 0.00 H

ATOM 1429 CA LYS 88 69.750 62.274 36.801 1.00 0.00 C

ATOM 1430 HA LYS 88 68.727 62.651 36.775 1.00 0.00 H

ATOM 1431 CB LYS 88 69.838 60.914 36.154 1.00 0.00 C

ATOM 1432 HB2 LYS 88 70.844 60.673 35.810 1.00 0.00 H

ATOM 1433 HB3 LYS 88 69.424 60.171 36.836 1.00 0.00 H

ATOM 1434 CG LYS 88 68.932 60.777 34.906 1.00 0.00 C

ATOM 1435 HG2 LYS 88 67.898 60.745 35.250 1.00 0.00 H

ATOM 1436 HG3 LYS 88 69.177 61.660 34.317 1.00 0.00 H

ATOM 1437 CD LYS 88 69.316 59.517 34.196 1.00 0.00 C

ATOM 1438 HD2 LYS 88 70.372 59.425 33.945 1.00 0.00 H

ATOM 1439 HD3 LYS 88 69.070 58.665 34.830 1.00 0.00 H

ATOM 1440 CE LYS 88 68.451 59.545 32.914 1.00 0.00 C

ATOM 1441 HE2 LYS 88 67.523 59.102 33.278 1.00 0.00 H

ATOM 1442 HE3 LYS 88 68.281 60.553 32.535 1.00 0.00 H

ATOM 1443 NZ LYS 88 69.072 58.772 31.921 1.00 0.00 N

ATOM 1444 HZ1 LYS 88 70.050 58.926 31.725 1.00 0.00 H

ATOM 1445 HZ2 LYS 88 68.948 57.824 32.245 1.00 0.00 H

ATOM 1446 HZ3 LYS 88 68.560 58.718 31.052 1.00 0.00 H

ATOM 1447 C LYS 88 69.976 62.240 38.279 1.00 0.00 C

ATOM 1448 O LYS 88 68.993 62.206 39.017 1.00 0.00 O

ATOM 1449 N LEU 89 71.187 62.555 38.802 1.00 0.00 N

ATOM 1450 H LEU 89 71.894 62.737 38.104 1.00 0.00 H

ATOM 1451 CA LEU 89 71.342 63.143 40.112 1.00 0.00 C

ATOM 1452 HA LEU 89 70.989 62.437 40.864 1.00 0.00 H

ATOM 1453 CB LEU 89 72.895 63.288 40.310 1.00 0.00 C

ATOM 1454 HB2 LEU 89 73.478 62.379 40.160 1.00 0.00 H

ATOM 1455 HB3 LEU 89 73.287 64.103 39.702 1.00 0.00 H

ATOM 1456 CG LEU 89 73.165 63.802 41.793 1.00 0.00 C

ATOM 1457 HG LEU 89 72.741 64.787 41.986 1.00 0.00 H

ATOM 1458 CD1 LEU 89 72.681 62.750 42.764 1.00 0.00 C

ATOM 1459 HD11 LEU 89 72.957 63.207 43.714 1.00 0.00 H

ATOM 1460 HD12 LEU 89 71.592 62.702 42.781 1.00 0.00 H

ATOM 1461 HD13 LEU 89 73.165 61.813 42.489 1.00 0.00 H

ATOM 1462 CD2 LEU 89 74.694 64.050 42.008 1.00 0.00 C

ATOM 1463 HD21 LEU 89 74.958 64.631 42.892 1.00 0.00 H

ATOM 1464 HD22 LEU 89 75.153 63.062 42.048 1.00 0.00 H

ATOM 1465 HD23 LEU 89 75.075 64.724 41.241 1.00 0.00 H

ATOM 1466 C LEU 89 70.474 64.428 40.296 1.00 0.00 C

ATOM 1467 O LEU 89 69.717 64.543 41.257 1.00 0.00 O

ATOM 1468 N GLN 90 70.665 65.495 39.497 1.00 0.00 N

ATOM 1469 H GLN 90 71.185 65.303 38.652 1.00 0.00 H

ATOM 1470 CA GLN 90 70.141 66.764 39.798 1.00 0.00 C

ATOM 1471 HA GLN 90 70.519 67.172 40.736 1.00 0.00 H

ATOM 1472 CB GLN 90 70.637 67.758 38.692 1.00 0.00 C

ATOM 1473 HB2 GLN 90 71.654 67.507 38.390 1.00 0.00 H

ATOM 1474 HB3 GLN 90 69.995 67.597 37.826 1.00 0.00 H

ATOM 1475 CG GLN 90 70.468 69.318 39.071 1.00 0.00 C

ATOM 1476 HG2 GLN 90 69.986 69.868 38.263 1.00 0.00 H

ATOM 1477 HG3 GLN 90 69.801 69.431 39.926 1.00 0.00 H

ATOM 1478 CD GLN 90 71.741 70.021 39.367 1.00 0.00 C

ATOM 1479 OE1 GLN 90 72.829 69.566 39.091 1.00 0.00 O

ATOM 1480 NE2 GLN 90 71.648 71.212 39.905 1.00 0.00 N

ATOM 1481 HE21 GLN 90 72.533 71.587 40.215 1.00 0.00 H

ATOM 1482 HE22 GLN 90 70.756 71.574 40.211 1.00 0.00 H

ATOM 1483 C GLN 90 68.635 66.861 39.962 1.00 0.00 C

ATOM 1484 O GLN 90 68.152 67.472 40.909 1.00 0.00 O

ATOM 1485 N ARG 91 67.928 66.032 39.135 1.00 0.00 N

ATOM 1486 H ARG 91 68.400 65.770 38.282 1.00 0.00 H

ATOM 1487 CA ARG 91 66.505 65.831 39.169 1.00 0.00 C

ATOM 1488 HA ARG 91 66.005 66.799 39.185 1.00 0.00 H

ATOM 1489 CB ARG 91 66.105 65.251 37.875 1.00 0.00 C

ATOM 1490 HB2 ARG 91 66.870 64.533 37.578 1.00 0.00 H

ATOM 1491 HB3 ARG 91 65.132 64.761 37.901 1.00 0.00 H

ATOM 1492 CG ARG 91 66.164 66.146 36.651 1.00 0.00 C

ATOM 1493 HG2 ARG 91 65.570 67.056 36.741 1.00 0.00 H

ATOM 1494 HG3 ARG 91 67.166 66.519 36.437 1.00 0.00 H

ATOM 1495 CD ARG 91 65.616 65.357 35.462 1.00 0.00 C

ATOM 1496 HD2 ARG 91 65.812 64.304 35.667 1.00 0.00 H

ATOM 1497 HD3 ARG 91 64.533 65.240 35.499 1.00 0.00 H

ATOM 1498 NE ARG 91 66.111 65.858 34.154 1.00 0.00 N

ATOM 1499 HE ARG 91 66.945 65.441 33.764 1.00 0.00 H

ATOM 1500 CZ ARG 91 65.319 66.571 33.326 1.00 0.00 C

ATOM 1501 NH1 ARG 91 64.579 67.541 33.756 1.00 0.00 N

ATOM 1502 HH11 ARG 91 64.486 68.399 33.232 1.00 0.00 H

ATOM 1503 HH12 ARG 91 64.490 67.719 34.746 1.00 0.00 H

ATOM 1504 NH2 ARG 91 65.563 66.743 32.054 1.00 0.00 N

ATOM 1505 HH21 ARG 91 66.098 66.076 31.516 1.00 0.00 H

ATOM 1506 HH22 ARG 91 64.939 67.330 31.521 1.00 0.00 H

ATOM 1507 C ARG 91 65.993 64.928 40.318 1.00 0.00 C

ATOM 1508 O ARG 91 65.038 65.408 40.975 1.00 0.00 O

ATOM 1509 N PHE 92 66.670 63.820 40.715 1.00 0.00 N

ATOM 1510 H PHE 92 67.507 63.560 40.213 1.00 0.00 H

ATOM 1511 CA PHE 92 66.340 63.110 41.988 1.00 0.00 C

ATOM 1512 HA PHE 92 65.312 62.754 41.919 1.00 0.00 H

ATOM 1513 CB PHE 92 67.228 61.885 42.165 1.00 0.00 C

ATOM 1514 HB2 PHE 92 66.883 61.112 41.478 1.00 0.00 H

ATOM 1515 HB3 PHE 92 68.253 62.102 41.862 1.00 0.00 H

ATOM 1516 CG PHE 92 67.320 61.250 43.528 1.00 0.00 C

ATOM 1517 CD1 PHE 92 66.115 60.780 44.165 1.00 0.00 C

ATOM 1518 HD1 PHE 92 65.330 60.639 43.438 1.00 0.00 H

ATOM 1519 CE1 PHE 92 66.103 60.209 45.474 1.00 0.00 C

ATOM 1520 HE1 PHE 92 65.303 59.572 45.819 1.00 0.00 H

ATOM 1521 CZ PHE 92 67.170 60.496 46.297 1.00 0.00 C

ATOM 1522 HZ PHE 92 67.390 60.143 47.294 1.00 0.00 H

ATOM 1523 CE2 PHE 92 68.342 61.054 45.746 1.00 0.00 C

ATOM 1524 HE2 PHE 92 69.173 61.205 46.419 1.00 0.00 H

ATOM 1525 CD2 PHE 92 68.342 61.523 44.427 1.00 0.00 C

ATOM 1526 HD2 PHE 92 69.263 61.885 43.995 1.00 0.00 H

ATOM 1527 C PHE 92 66.390 63.974 43.215 1.00 0.00 C

ATOM 1528 O PHE 92 65.412 64.132 43.927 1.00 0.00 O

ATOM 1529 N ILE 93 67.365 64.906 43.186 1.00 0.00 N

ATOM 1530 H ILE 93 68.139 64.905 42.537 1.00 0.00 H

ATOM 1531 CA ILE 93 67.554 65.736 44.378 1.00 0.00 C

ATOM 1532 HA ILE 93 67.262 65.093 45.209 1.00 0.00 H

ATOM 1533 CB ILE 93 69.025 66.161 44.497 1.00 0.00 C

ATOM 1534 HB ILE 93 69.318 66.421 43.480 1.00 0.00 H

ATOM 1535 CG2 ILE 93 69.153 67.573 45.214 1.00 0.00 C

ATOM 1536 HG21 ILE 93 68.465 68.270 44.736 1.00 0.00 H

ATOM 1537 HG22 ILE 93 68.932 67.437 46.272 1.00 0.00 H

ATOM 1538 HG23 ILE 93 70.142 68.016 45.099 1.00 0.00 H

ATOM 1539 CG1 ILE 93 69.932 65.112 45.202 1.00 0.00 C

ATOM 1540 HG12 ILE 93 69.808 65.233 46.278 1.00 0.00 H

ATOM 1541 HG13 ILE 93 69.701 64.074 44.961 1.00 0.00 H

ATOM 1542 CD1 ILE 93 71.388 65.282 44.875 1.00 0.00 C

ATOM 1543 HD11 ILE 93 71.656 66.298 45.166 1.00 0.00 H

ATOM 1544 HD12 ILE 93 72.041 64.491 45.243 1.00 0.00 H

ATOM 1545 HD13 ILE 93 71.456 65.198 43.791 1.00 0.00 H

ATOM 1546 C ILE 93 66.491 66.905 44.329 1.00 0.00 C

ATOM 1547 O ILE 93 65.766 67.000 45.284 1.00 0.00 O

ATOM 1548 N PHE 94 66.170 67.413 43.145 1.00 0.00 N

ATOM 1549 H PHE 94 66.737 67.139 42.356 1.00 0.00 H

ATOM 1550 CA PHE 94 64.966 68.291 42.948 1.00 0.00 C

ATOM 1551 HA PHE 94 65.026 69.135 43.635 1.00 0.00 H

ATOM 1552 CB PHE 94 65.044 68.761 41.538 1.00 0.00 C

ATOM 1553 HB2 PHE 94 65.800 69.546 41.500 1.00 0.00 H

ATOM 1554 HB3 PHE 94 65.178 67.926 40.850 1.00 0.00 H

ATOM 1555 CG PHE 94 63.792 69.507 40.970 1.00 0.00 C

ATOM 1556 CD1 PHE 94 63.581 70.894 41.283 1.00 0.00 C

ATOM 1557 HD1 PHE 94 64.235 71.374 41.996 1.00 0.00 H

ATOM 1558 CE1 PHE 94 62.451 71.655 40.898 1.00 0.00 C

ATOM 1559 HE1 PHE 94 62.374 72.698 41.168 1.00 0.00 H

ATOM 1560 CZ PHE 94 61.515 71.050 40.015 1.00 0.00 C

ATOM 1561 HZ PHE 94 60.623 71.518 39.624 1.00 0.00 H

ATOM 1562 CE2 PHE 94 61.697 69.647 39.802 1.00 0.00 C

ATOM 1563 HE2 PHE 94 60.978 69.067 39.242 1.00 0.00 H

ATOM 1564 CD2 PHE 94 62.890 68.987 40.075 1.00 0.00 C

ATOM 1565 HD2 PHE 94 62.988 67.958 39.762 1.00 0.00 H

ATOM 1566 C PHE 94 63.606 67.724 43.248 1.00 0.00 C

ATOM 1567 O PHE 94 62.697 68.394 43.782 1.00 0.00 O

ATOM 1568 N HID 95 63.356 66.393 43.075 1.00 0.00 N

ATOM 1569 H HID 95 64.219 65.906 42.881 1.00 0.00 H

ATOM 1570 CA HID 95 62.065 65.737 43.334 1.00 0.00 C

ATOM 1571 HA HID 95 61.300 66.468 43.074 1.00 0.00 H

ATOM 1572 CB HID 95 61.831 64.559 42.427 1.00 0.00 C

ATOM 1573 HB2 HID 95 62.668 63.872 42.305 1.00 0.00 H

ATOM 1574 HB3 HID 95 60.982 64.011 42.836 1.00 0.00 H

ATOM 1575 CG HID 95 61.382 64.986 41.001 1.00 0.00 C

ATOM 1576 ND1 HID 95 62.319 65.468 40.059 1.00 0.00 N

ATOM 1577 HD1 HID 95 63.302 65.595 40.252 1.00 0.00 H

ATOM 1578 CE1 HID 95 61.594 65.982 39.042 1.00 0.00 C

ATOM 1579 HE1 HID 95 62.083 66.486 38.222 1.00 0.00 H

ATOM 1580 NE2 HID 95 60.261 65.859 39.255 1.00 0.00 N

ATOM 1581 CD2 HID 95 60.126 65.278 40.508 1.00 0.00 C

ATOM 1582 HD2 HID 95 59.158 65.272 40.986 1.00 0.00 H

ATOM 1583 C HID 95 61.988 65.282 44.765 1.00 0.00 C

ATOM 1584 O HID 95 60.971 65.386 45.435 1.00 0.00 O

ATOM 1585 N VAL 96 63.080 64.986 45.466 1.00 0.00 N

ATOM 1586 H VAL 96 63.895 64.894 44.877 1.00 0.00 H

ATOM 1587 CA VAL 96 63.212 64.741 46.920 1.00 0.00 C

ATOM 1588 HA VAL 96 62.274 64.283 47.235 1.00 0.00 H

ATOM 1589 CB VAL 96 64.330 63.695 47.192 1.00 0.00 C

ATOM 1590 HB VAL 96 64.669 63.282 46.242 1.00 0.00 H

ATOM 1591 CG1 VAL 96 65.545 64.139 47.993 1.00 0.00 C

ATOM 1592 HG11 VAL 96 65.206 64.348 49.008 1.00 0.00 H

ATOM 1593 HG12 VAL 96 66.252 63.312 47.932 1.00 0.00 H

ATOM 1594 HG13 VAL 96 65.960 65.023 47.508 1.00 0.00 H

ATOM 1595 CG2 VAL 96 63.725 62.526 47.998 1.00 0.00 C

ATOM 1596 HG21 VAL 96 63.198 61.937 47.247 1.00 0.00 H

ATOM 1597 HG22 VAL 96 64.358 61.773 48.467 1.00 0.00 H

ATOM 1598 HG23 VAL 96 63.091 62.953 48.775 1.00 0.00 H

ATOM 1599 C VAL 96 63.260 65.965 47.795 1.00 0.00 C

ATOM 1600 O VAL 96 62.742 65.866 48.957 1.00 0.00 O

ATOM 1601 N ASN 97 63.655 67.163 47.286 1.00 0.00 N

ATOM 1602 H ASN 97 63.884 67.097 46.305 1.00 0.00 H

ATOM 1603 CA ASN 97 63.312 68.454 47.939 1.00 0.00 C

ATOM 1604 HA ASN 97 63.711 68.485 48.953 1.00 0.00 H

ATOM 1605 CB ASN 97 63.904 69.619 47.031 1.00 0.00 C

ATOM 1606 HB2 ASN 97 64.948 69.482 46.746 1.00 0.00 H

ATOM 1607 HB3 ASN 97 63.406 69.599 46.061 1.00 0.00 H

ATOM 1608 CG ASN 97 63.760 70.998 47.628 1.00 0.00 C

ATOM 1609 OD1 ASN 97 63.219 71.873 46.936 1.00 0.00 O

ATOM 1610 ND2 ASN 97 64.351 71.285 48.755 1.00 0.00 N

ATOM 1611 HD21 ASN 97 64.238 72.235 49.078 1.00 0.00 H

ATOM 1612 HD22 ASN 97 65.132 70.786 49.157 1.00 0.00 H

ATOM 1613 C ASN 97 61.801 68.643 48.156 1.00 0.00 C

ATOM 1614 O ASN 97 61.514 69.369 49.132 1.00 0.00 O

ATOM 1615 N LEU 98 60.850 67.934 47.472 1.00 0.00 N

ATOM 1616 H LEU 98 61.140 67.189 46.855 1.00 0.00 H

ATOM 1617 CA LEU 98 59.454 68.044 47.786 1.00 0.00 C

ATOM 1618 HA LEU 98 59.320 68.945 48.386 1.00 0.00 H

ATOM 1619 CB LEU 98 58.609 68.432 46.604 1.00 0.00 C

ATOM 1620 HB2 LEU 98 59.007 69.323 46.119 1.00 0.00 H

ATOM 1621 HB3 LEU 98 58.649 67.675 45.821 1.00 0.00 H

ATOM 1622 CG LEU 98 57.140 68.676 46.904 1.00 0.00 C

ATOM 1623 HG LEU 98 56.725 67.895 47.541 1.00 0.00 H

ATOM 1624 CD1 LEU 98 56.992 69.882 47.841 1.00 0.00 C

ATOM 1625 HD11 LEU 98 57.726 70.679 47.725 1.00 0.00 H

ATOM 1626 HD12 LEU 98 55.979 70.256 47.696 1.00 0.00 H

ATOM 1627 HD13 LEU 98 57.108 69.505 48.857 1.00 0.00 H

ATOM 1628 CD2 LEU 98 56.227 68.832 45.666 1.00 0.00 C

ATOM 1629 HD21 LEU 98 56.622 69.636 45.045 1.00 0.00 H

ATOM 1630 HD22 LEU 98 56.160 67.890 45.122 1.00 0.00 H

ATOM 1631 HD23 LEU 98 55.263 69.278 45.909 1.00 0.00 H

ATOM 1632 C LEU 98 59.046 66.747 48.455 1.00 0.00 C

ATOM 1633 O LEU 98 58.719 66.622 49.603 1.00 0.00 O

ATOM 1634 N TYR 99 59.272 65.642 47.752 1.00 0.00 N

ATOM 1635 H TYR 99 59.696 65.799 46.849 1.00 0.00 H

ATOM 1636 CA TYR 99 58.718 64.278 48.128 1.00 0.00 C

ATOM 1637 HA TYR 99 57.635 64.394 48.174 1.00 0.00 H

ATOM 1638 CB TYR 99 58.843 63.251 47.027 1.00 0.00 C

ATOM 1639 HB2 TYR 99 59.910 63.129 46.842 1.00 0.00 H

ATOM 1640 HB3 TYR 99 58.422 62.305 47.367 1.00 0.00 H

ATOM 1641 CG TYR 99 58.248 63.637 45.723 1.00 0.00 C

ATOM 1642 CD1 TYR 99 57.190 64.639 45.542 1.00 0.00 C

ATOM 1643 HD1 TYR 99 56.671 65.039 46.400 1.00 0.00 H

ATOM 1644 CE1 TYR 99 56.889 65.054 44.246 1.00 0.00 C

ATOM 1645 HE1 TYR 99 56.207 65.885 44.141 1.00 0.00 H

ATOM 1646 CZ TYR 99 57.244 64.294 43.079 1.00 0.00 C

ATOM 1647 OH TYR 99 56.599 64.624 41.964 1.00 0.00 O

ATOM 1648 HH TYR 99 56.895 64.110 41.209 1.00 0.00 H

ATOM 1649 CE2 TYR 99 58.208 63.305 43.233 1.00 0.00 C

ATOM 1650 HE2 TYR 99 58.493 62.740 42.358 1.00 0.00 H

ATOM 1651 CD2 TYR 99 58.756 63.015 44.516 1.00 0.00 C

ATOM 1652 HD2 TYR 99 59.190 62.041 44.690 1.00 0.00 H

ATOM 1653 C TYR 99 59.305 63.701 49.457 1.00 0.00 C

ATOM 1654 O TYR 99 58.700 62.844 50.052 1.00 0.00 O

ATOM 1655 N GLY 100 60.496 64.202 49.795 1.00 0.00 N

ATOM 1656 H GLY 100 60.981 64.909 49.260 1.00 0.00 H

ATOM 1657 CA GLY 100 61.161 63.737 50.944 1.00 0.00 C

ATOM 1658 HA2 GLY 100 60.972 62.677 51.114 1.00 0.00 H

ATOM 1659 HA3 GLY 100 62.180 63.994 50.655 1.00 0.00 H

ATOM 1660 C GLY 100 60.811 64.666 52.105 1.00 0.00 C

ATOM 1661 O GLY 100 60.514 64.129 53.257 1.00 0.00 O

ATOM 1662 N SER 101 60.841 65.996 51.895 1.00 0.00 N

ATOM 1663 H SER 101 60.999 66.267 50.934 1.00 0.00 H

ATOM 1664 CA SER 101 60.643 67.120 52.802 1.00 0.00 C

ATOM 1665 HA SER 101 61.223 67.090 53.724 1.00 0.00 H

ATOM 1666 CB SER 101 60.941 68.523 52.267 1.00 0.00 C

ATOM 1667 HB2 SER 101 60.849 69.123 53.172 1.00 0.00 H

ATOM 1668 HB3 SER 101 61.943 68.676 51.865 1.00 0.00 H

ATOM 1669 OG SER 101 60.093 68.970 51.249 1.00 0.00 O

ATOM 1670 HG SER 101 60.551 68.983 50.406 1.00 0.00 H

ATOM 1671 C SER 101 59.289 66.985 53.368 1.00 0.00 C

ATOM 1672 O SER 101 59.212 66.851 54.580 1.00 0.00 O

ATOM 1673 N ILE 102 58.269 66.850 52.522 1.00 0.00 N

ATOM 1674 H ILE 102 58.486 66.951 51.541 1.00 0.00 H

ATOM 1675 CA ILE 102 56.854 66.826 53.079 1.00 0.00 C

ATOM 1676 HA ILE 102 56.707 67.591 53.841 1.00 0.00 H

ATOM 1677 CB ILE 102 55.924 67.075 51.869 1.00 0.00 C

ATOM 1678 HB ILE 102 56.348 67.897 51.293 1.00 0.00 H

ATOM 1679 CG2 ILE 102 55.730 65.701 51.037 1.00 0.00 C

ATOM 1680 HG21 ILE 102 55.183 64.987 51.654 1.00 0.00 H

ATOM 1681 HG22 ILE 102 55.220 65.931 50.101 1.00 0.00 H

ATOM 1682 HG23 ILE 102 56.733 65.360 50.779 1.00 0.00 H

ATOM 1683 CG1 ILE 102 54.608 67.652 52.420 1.00 0.00 C

ATOM 1684 HG12 ILE 102 54.160 66.995 53.166 1.00 0.00 H

ATOM 1685 HG13 ILE 102 54.944 68.603 52.832 1.00 0.00 H

ATOM 1686 CD1 ILE 102 53.688 67.864 51.212 1.00 0.00 C

ATOM 1687 HD11 ILE 102 54.249 68.343 50.410 1.00 0.00 H

ATOM 1688 HD12 ILE 102 53.410 66.933 50.718 1.00 0.00 H

ATOM 1689 HD13 ILE 102 52.744 68.378 51.395 1.00 0.00 H

ATOM 1690 C ILE 102 56.579 65.512 53.882 1.00 0.00 C

ATOM 1691 O ILE 102 55.678 65.510 54.742 1.00 0.00 O

ATOM 1692 N LEU 103 57.406 64.429 53.687 1.00 0.00 N

ATOM 1693 H LEU 103 58.162 64.321 53.026 1.00 0.00 H

ATOM 1694 CA LEU 103 57.264 63.277 54.617 1.00 0.00 C

ATOM 1695 HA LEU 103 56.183 63.144 54.635 1.00 0.00 H

ATOM 1696 CB LEU 103 57.810 62.029 53.948 1.00 0.00 C

ATOM 1697 HB2 LEU 103 58.772 62.375 53.570 1.00 0.00 H

ATOM 1698 HB3 LEU 103 57.899 61.288 54.742 1.00 0.00 H

ATOM 1699 CG LEU 103 57.014 61.485 52.793 1.00 0.00 C

ATOM 1700 HG LEU 103 57.003 62.290 52.058 1.00 0.00 H

ATOM 1701 CD1 LEU 103 57.760 60.297 52.219 1.00 0.00 C

ATOM 1702 HD11 LEU 103 58.834 60.391 52.056 1.00 0.00 H

ATOM 1703 HD12 LEU 103 57.701 59.450 52.903 1.00 0.00 H

ATOM 1704 HD13 LEU 103 57.181 59.981 51.351 1.00 0.00 H

ATOM 1705 CD2 LEU 103 55.618 61.019 53.269 1.00 0.00 C

ATOM 1706 HD21 LEU 103 55.062 61.859 53.687 1.00 0.00 H

ATOM 1707 HD22 LEU 103 55.005 60.626 52.459 1.00 0.00 H

ATOM 1708 HD23 LEU 103 55.659 60.267 54.057 1.00 0.00 H

ATOM 1709 C LEU 103 57.818 63.519 56.006 1.00 0.00 C

ATOM 1710 O LEU 103 57.153 63.150 57.014 1.00 0.00 O

ATOM 1711 N PHE 104 59.010 64.024 56.177 1.00 0.00 N

ATOM 1712 H PHE 104 59.471 64.385 55.354 1.00 0.00 H

ATOM 1713 CA PHE 104 59.577 64.525 57.484 1.00 0.00 C

ATOM 1714 HA PHE 104 59.518 63.763 58.262 1.00 0.00 H

ATOM 1715 CB PHE 104 61.066 64.897 57.230 1.00 0.00 C

ATOM 1716 HB2 PHE 104 60.979 65.648 56.444 1.00 0.00 H

ATOM 1717 HB3 PHE 104 61.519 65.436 58.063 1.00 0.00 H

ATOM 1718 CG PHE 104 62.063 63.825 56.806 1.00 0.00 C

ATOM 1719 CD1 PHE 104 62.249 62.668 57.586 1.00 0.00 C

ATOM 1720 HD1 PHE 104 61.815 62.691 58.575 1.00 0.00 H

ATOM 1721 CE1 PHE 104 62.934 61.546 57.158 1.00 0.00 C

ATOM 1722 HE1 PHE 104 63.140 60.706 57.806 1.00 0.00 H

ATOM 1723 CZ PHE 104 63.353 61.544 55.781 1.00 0.00 C

ATOM 1724 HZ PHE 104 63.828 60.649 55.406 1.00 0.00 H

ATOM 1725 CE2 PHE 104 63.309 62.722 55.024 1.00 0.00 C

ATOM 1726 HE2 PHE 104 63.493 62.734 53.960 1.00 0.00 H

ATOM 1727 CD2 PHE 104 62.686 63.850 55.536 1.00 0.00 C

ATOM 1728 HD2 PHE 104 62.550 64.704 54.890 1.00 0.00 H

ATOM 1729 C PHE 104 58.806 65.788 58.044 1.00 0.00 C

ATOM 1730 O PHE 104 58.475 65.711 59.226 1.00 0.00 O

ATOM 1731 N LEU 105 58.171 66.652 57.222 1.00 0.00 N

ATOM 1732 H LEU 105 58.633 66.969 56.381 1.00 0.00 H

ATOM 1733 CA LEU 105 57.086 67.591 57.635 1.00 0.00 C

ATOM 1734 HA LEU 105 57.420 68.217 58.463 1.00 0.00 H

ATOM 1735 CB LEU 105 56.760 68.653 56.449 1.00 0.00 C

ATOM 1736 HB2 LEU 105 57.611 68.660 55.769 1.00 0.00 H

ATOM 1737 HB3 LEU 105 55.814 68.356 55.995 1.00 0.00 H

ATOM 1738 CG LEU 105 56.717 70.074 57.084 1.00 0.00 C

ATOM 1739 HG LEU 105 57.735 70.316 57.390 1.00 0.00 H

ATOM 1740 CD1 LEU 105 56.371 70.965 55.857 1.00 0.00 C

ATOM 1741 HD11 LEU 105 56.288 72.012 56.149 1.00 0.00 H

ATOM 1742 HD12 LEU 105 57.154 70.828 55.111 1.00 0.00 H

ATOM 1743 HD13 LEU 105 55.421 70.731 55.379 1.00 0.00 H

ATOM 1744 CD2 LEU 105 55.780 70.397 58.185 1.00 0.00 C

ATOM 1745 HD21 LEU 105 56.077 69.945 59.132 1.00 0.00 H

ATOM 1746 HD22 LEU 105 55.558 71.449 58.365 1.00 0.00 H

ATOM 1747 HD23 LEU 105 54.806 70.166 57.755 1.00 0.00 H

ATOM 1748 C LEU 105 55.794 66.929 58.208 1.00 0.00 C

ATOM 1749 O LEU 105 55.308 67.395 59.239 1.00 0.00 O

ATOM 1750 N THR 106 55.357 65.824 57.713 1.00 0.00 N

ATOM 1751 H THR 106 55.945 65.379 57.024 1.00 0.00 H

ATOM 1752 CA THR 106 54.313 64.920 58.247 1.00 0.00 C

ATOM 1753 HA THR 106 53.449 65.533 58.504 1.00 0.00 H

ATOM 1754 CB THR 106 53.942 63.791 57.270 1.00 0.00 C

ATOM 1755 HB THR 106 54.686 63.009 57.121 1.00 0.00 H

ATOM 1756 CG2 THR 106 52.793 62.988 57.935 1.00 0.00 C

ATOM 1757 HG21 THR 106 53.358 62.361 58.625 1.00 0.00 H

ATOM 1758 HG22 THR 106 52.081 63.550 58.539 1.00 0.00 H

ATOM 1759 HG23 THR 106 52.400 62.382 57.118 1.00 0.00 H

ATOM 1760 OG1 THR 106 53.394 64.251 56.077 1.00 0.00 O

ATOM 1761 HG1 THR 106 53.768 65.118 55.905 1.00 0.00 H

ATOM 1762 C THR 106 54.658 64.366 59.617 1.00 0.00 C

ATOM 1763 O THR 106 53.986 64.754 60.545 1.00 0.00 O

ATOM 1764 N CYX 107 55.825 63.786 59.815 1.00 0.00 N

ATOM 1765 H CYX 107 56.366 63.501 59.011 1.00 0.00 H

ATOM 1766 CA CYX 107 56.350 63.382 61.191 1.00 0.00 C

ATOM 1767 HA CYX 107 55.712 62.523 61.397 1.00 0.00 H

ATOM 1768 CB CYX 107 57.795 62.731 61.041 1.00 0.00 C

ATOM 1769 HB2 CYX 107 58.470 63.459 60.590 1.00 0.00 H

ATOM 1770 HB3 CYX 107 58.154 62.459 62.034 1.00 0.00 H

ATOM 1771 SG CYX 107 57.679 61.209 59.922 1.00 0.00 S

ATOM 1772 C CYX 107 56.258 64.557 62.238 1.00 0.00 C

ATOM 1773 O CYX 107 55.766 64.339 63.386 1.00 0.00 O

ATOM 1774 N ILE 108 56.797 65.731 61.916 1.00 0.00 N

ATOM 1775 H ILE 108 57.351 65.802 61.074 1.00 0.00 H

ATOM 1776 CA ILE 108 56.769 66.824 62.915 1.00 0.00 C

ATOM 1777 HA ILE 108 57.139 66.370 63.834 1.00 0.00 H

ATOM 1778 CB ILE 108 57.771 67.992 62.508 1.00 0.00 C

ATOM 1779 HB ILE 108 57.409 68.426 61.576 1.00 0.00 H

ATOM 1780 CG2 ILE 108 57.779 69.064 63.659 1.00 0.00 C

ATOM 1781 HG21 ILE 108 58.793 69.276 63.999 1.00 0.00 H

ATOM 1782 HG22 ILE 108 57.533 69.992 63.142 1.00 0.00 H

ATOM 1783 HG23 ILE 108 57.099 68.852 64.484 1.00 0.00 H

ATOM 1784 CG1 ILE 108 59.153 67.472 62.218 1.00 0.00 C

ATOM 1785 HG12 ILE 108 59.678 67.388 63.170 1.00 0.00 H

ATOM 1786 HG13 ILE 108 59.149 66.489 61.745 1.00 0.00 H

ATOM 1787 CD1 ILE 108 59.914 68.479 61.444 1.00 0.00 C

ATOM 1788 HD11 ILE 108 59.473 68.804 60.502 1.00 0.00 H

ATOM 1789 HD12 ILE 108 60.145 69.398 61.981 1.00 0.00 H

ATOM 1790 HD13 ILE 108 60.871 68.074 61.113 1.00 0.00 H

ATOM 1791 C ILE 108 55.309 67.332 63.153 1.00 0.00 C

ATOM 1792 O ILE 108 54.928 67.533 64.256 1.00 0.00 O

ATOM 1793 N SER 109 54.555 67.357 62.024 1.00 0.00 N

ATOM 1794 H SER 109 55.076 67.187 61.176 1.00 0.00 H

ATOM 1795 CA SER 109 53.140 67.765 61.998 1.00 0.00 C

ATOM 1796 HA SER 109 53.059 68.684 62.578 1.00 0.00 H

ATOM 1797 CB SER 109 52.634 67.985 60.605 1.00 0.00 C

ATOM 1798 HB2 SER 109 52.702 67.089 59.989 1.00 0.00 H

ATOM 1799 HB3 SER 109 51.569 68.213 60.663 1.00 0.00 H

ATOM 1800 OG SER 109 53.250 69.037 60.004 1.00 0.00 O

ATOM 1801 HG SER 109 54.133 68.712 59.814 1.00 0.00 H

ATOM 1802 C SER 109 52.242 66.797 62.876 1.00 0.00 C

ATOM 1803 O SER 109 51.462 67.229 63.690 1.00 0.00 O

ATOM 1804 N ALA 110 52.388 65.501 62.680 1.00 0.00 N

ATOM 1805 H ALA 110 53.204 65.190 62.173 1.00 0.00 H

ATOM 1806 CA ALA 110 51.631 64.500 63.429 1.00 0.00 C

ATOM 1807 HA ALA 110 50.561 64.569 63.231 1.00 0.00 H

ATOM 1808 CB ALA 110 51.996 63.128 62.871 1.00 0.00 C

ATOM 1809 HB1 ALA 110 51.639 63.007 61.848 1.00 0.00 H

ATOM 1810 HB2 ALA 110 53.044 62.881 63.041 1.00 0.00 H

ATOM 1811 HB3 ALA 110 51.472 62.332 63.400 1.00 0.00 H

ATOM 1812 C ALA 110 51.880 64.633 64.936 1.00 0.00 C

ATOM 1813 O ALA 110 51.001 64.513 65.763 1.00 0.00 O

ATOM 1814 N HID 111 53.140 64.898 65.266 1.00 0.00 N

ATOM 1815 H HID 111 53.825 64.974 64.527 1.00 0.00 H

ATOM 1816 CA HID 111 53.645 65.003 66.571 1.00 0.00 C

ATOM 1817 HA HID 111 53.122 64.301 67.220 1.00 0.00 H

ATOM 1818 CB HID 111 55.044 64.451 66.593 1.00 0.00 C

ATOM 1819 HB2 HID 111 55.105 63.715 65.791 1.00 0.00 H

ATOM 1820 HB3 HID 111 55.719 65.278 66.368 1.00 0.00 H

ATOM 1821 CG HID 111 55.651 63.916 67.845 1.00 0.00 C

ATOM 1822 ND1 HID 111 54.920 63.707 68.995 1.00 0.00 N

ATOM 1823 HD1 HID 111 53.968 64.025 69.105 1.00 0.00 H

ATOM 1824 CE1 HID 111 55.363 62.675 69.680 1.00 0.00 C

ATOM 1825 HE1 HID 111 54.669 62.035 70.204 1.00 0.00 H

ATOM 1826 NE2 HID 111 56.486 62.203 69.086 1.00 0.00 N

ATOM 1827 CD2 HID 111 56.593 62.899 67.890 1.00 0.00 C

ATOM 1828 HD2 HID 111 57.291 62.626 67.113 1.00 0.00 H

ATOM 1829 C HID 111 53.368 66.346 67.242 1.00 0.00 C

ATOM 1830 O HID 111 53.286 66.444 68.458 1.00 0.00 O

ATOM 1831 N ARG 112 53.089 67.407 66.522 1.00 0.00 N

ATOM 1832 H ARG 112 53.374 67.361 65.554 1.00 0.00 H

ATOM 1833 CA ARG 112 52.379 68.674 67.031 1.00 0.00 C

ATOM 1834 HA ARG 112 52.946 69.004 67.902 1.00 0.00 H

ATOM 1835 CB ARG 112 52.217 69.777 65.940 1.00 0.00 C

ATOM 1836 HB2 ARG 112 52.333 69.346 64.946 1.00 0.00 H

ATOM 1837 HB3 ARG 112 51.185 70.126 65.890 1.00 0.00 H

ATOM 1838 CG ARG 112 53.188 70.855 66.088 1.00 0.00 C

ATOM 1839 HG2 ARG 112 52.828 71.697 65.497 1.00 0.00 H

ATOM 1840 HG3 ARG 112 53.187 71.067 67.157 1.00 0.00 H

ATOM 1841 CD ARG 112 54.616 70.671 65.635 1.00 0.00 C

ATOM 1842 HD2 ARG 112 54.591 70.088 64.714 1.00 0.00 H

ATOM 1843 HD3 ARG 112 55.085 71.647 65.507 1.00 0.00 H

ATOM 1844 NE ARG 112 55.408 69.892 66.595 1.00 0.00 N

ATOM 1845 HE ARG 112 54.929 69.097 66.992 1.00 0.00 H

ATOM 1846 CZ ARG 112 56.661 70.048 66.874 1.00 0.00 C

ATOM 1847 NH1 ARG 112 57.175 69.324 67.778 1.00 0.00 N

ATOM 1848 HH11 ARG 112 56.581 68.717 68.324 1.00 0.00 H

ATOM 1849 HH12 ARG 112 58.145 69.471 68.019 1.00 0.00 H

ATOM 1850 NH2 ARG 112 57.425 70.892 66.286 1.00 0.00 N

ATOM 1851 HH21 ARG 112 58.361 70.981 66.653 1.00 0.00 H

ATOM 1852 HH22 ARG 112 57.119 71.483 65.526 1.00 0.00 H

ATOM 1853 C ARG 112 50.966 68.256 67.488 1.00 0.00 C

ATOM 1854 O ARG 112 50.625 68.603 68.612 1.00 0.00 O

ATOM 1855 N TYR 113 50.268 67.442 66.691 1.00 0.00 N

ATOM 1856 H TYR 113 50.677 67.338 65.774 1.00 0.00 H

ATOM 1857 CA TYR 113 48.834 67.105 66.985 1.00 0.00 C

ATOM 1858 HA TYR 113 48.289 68.029 67.179 1.00 0.00 H

ATOM 1859 CB TYR 113 48.138 66.432 65.791 1.00 0.00 C

ATOM 1860 HB2 TYR 113 47.837 67.098 64.982 1.00 0.00 H

ATOM 1861 HB3 TYR 113 48.997 65.984 65.291 1.00 0.00 H

ATOM 1862 CG TYR 113 47.126 65.380 66.077 1.00 0.00 C

ATOM 1863 CD1 TYR 113 45.801 65.625 66.185 1.00 0.00 C

ATOM 1864 HD1 TYR 113 45.504 66.663 66.135 1.00 0.00 H

ATOM 1865 CE1 TYR 113 44.934 64.665 66.493 1.00 0.00 C

ATOM 1866 HE1 TYR 113 43.892 64.840 66.714 1.00 0.00 H

ATOM 1867 CZ TYR 113 45.251 63.317 66.656 1.00 0.00 C

ATOM 1868 OH TYR 113 44.370 62.263 66.892 1.00 0.00 O

ATOM 1869 HH TYR 113 43.478 62.485 67.169 1.00 0.00 H

ATOM 1870 CE2 TYR 113 46.592 63.003 66.550 1.00 0.00 C

ATOM 1871 HE2 TYR 113 46.901 61.982 66.717 1.00 0.00 H

ATOM 1872 CD2 TYR 113 47.519 64.044 66.476 1.00 0.00 C

ATOM 1873 HD2 TYR 113 48.563 63.774 66.535 1.00 0.00 H

ATOM 1874 C TYR 113 48.747 66.252 68.344 1.00 0.00 C

ATOM 1875 O TYR 113 47.833 66.556 69.148 1.00 0.00 O

ATOM 1876 N SER 114 49.645 65.338 68.670 1.00 0.00 N

ATOM 1877 H SER 114 50.047 64.914 67.847 1.00 0.00 H

ATOM 1878 CA SER 114 49.772 64.653 69.946 1.00 0.00 C

ATOM 1879 HA SER 114 48.743 64.444 70.239 1.00 0.00 H

ATOM 1880 CB SER 114 50.429 63.293 69.795 1.00 0.00 C

ATOM 1881 HB2 SER 114 50.566 62.851 70.782 1.00 0.00 H

ATOM 1882 HB3 SER 114 49.752 62.611 69.280 1.00 0.00 H

ATOM 1883 OG SER 114 51.646 63.322 69.068 1.00 0.00 O

ATOM 1884 HG SER 114 51.845 62.435 68.761 1.00 0.00 H

ATOM 1885 C SER 114 50.359 65.468 71.085 1.00 0.00 C

ATOM 1886 O SER 114 49.809 65.542 72.191 1.00 0.00 O

ATOM 1887 N GLY 115 51.341 66.366 70.792 1.00 0.00 N

ATOM 1888 H GLY 115 51.760 66.420 69.875 1.00 0.00 H

ATOM 1889 CA GLY 115 51.857 67.417 71.720 1.00 0.00 C

ATOM 1890 HA2 GLY 115 52.225 67.015 72.665 1.00 0.00 H

ATOM 1891 HA3 GLY 115 52.684 67.927 71.225 1.00 0.00 H

ATOM 1892 C GLY 115 50.811 68.464 72.069 1.00 0.00 C

ATOM 1893 O GLY 115 51.003 69.391 72.898 1.00 0.00 O

ATOM 1894 N VAL 116 49.669 68.383 71.406 1.00 0.00 N

ATOM 1895 H VAL 116 49.700 67.949 70.495 1.00 0.00 H

ATOM 1896 CA VAL 116 48.485 69.198 71.829 1.00 0.00 C

ATOM 1897 HA VAL 116 48.734 69.936 72.592 1.00 0.00 H

ATOM 1898 CB VAL 116 47.927 69.978 70.574 1.00 0.00 C

ATOM 1899 HB VAL 116 48.018 69.360 69.681 1.00 0.00 H

ATOM 1900 CG1 VAL 116 46.520 70.552 70.615 1.00 0.00 C

ATOM 1901 HG11 VAL 116 45.804 69.741 70.481 1.00 0.00 H

ATOM 1902 HG12 VAL 116 46.373 71.071 71.562 1.00 0.00 H

ATOM 1903 HG13 VAL 116 46.199 71.295 69.884 1.00 0.00 H

ATOM 1904 CG2 VAL 116 48.816 71.096 70.205 1.00 0.00 C

ATOM 1905 HG21 VAL 116 48.954 71.640 71.139 1.00 0.00 H

ATOM 1906 HG22 VAL 116 49.749 70.607 69.923 1.00 0.00 H

ATOM 1907 HG23 VAL 116 48.471 71.693 69.361 1.00 0.00 H

ATOM 1908 C VAL 116 47.243 68.417 72.352 1.00 0.00 C

ATOM 1909 O VAL 116 46.695 68.758 73.434 1.00 0.00 O

ATOM 1910 N VAL 117 46.879 67.294 71.728 1.00 0.00 N

ATOM 1911 H VAL 117 47.291 67.002 70.854 1.00 0.00 H

ATOM 1912 CA VAL 117 45.722 66.554 72.155 1.00 0.00 C

ATOM 1913 HA VAL 117 45.027 67.283 72.572 1.00 0.00 H

ATOM 1914 CB VAL 117 45.108 65.722 70.973 1.00 0.00 C

ATOM 1915 HB VAL 117 45.903 65.085 70.583 1.00 0.00 H

ATOM 1916 CG1 VAL 117 43.928 64.954 71.503 1.00 0.00 C

ATOM 1917 HG11 VAL 117 44.210 64.155 72.189 1.00 0.00 H

ATOM 1918 HG12 VAL 117 43.161 65.431 72.114 1.00 0.00 H

ATOM 1919 HG13 VAL 117 43.451 64.376 70.712 1.00 0.00 H

ATOM 1920 CG2 VAL 117 44.657 66.539 69.788 1.00 0.00 C

ATOM 1921 HG21 VAL 117 45.463 67.215 69.502 1.00 0.00 H

ATOM 1922 HG22 VAL 117 44.693 65.761 69.026 1.00 0.00 H

ATOM 1923 HG23 VAL 117 43.686 67.031 69.843 1.00 0.00 H

ATOM 1924 C VAL 117 45.949 65.661 73.428 1.00 0.00 C

ATOM 1925 O VAL 117 45.073 65.554 74.247 1.00 0.00 O

ATOM 1926 N TYR 118 47.172 65.214 73.640 1.00 0.00 N

ATOM 1927 H TYR 118 47.923 65.318 72.973 1.00 0.00 H

ATOM 1928 CA TYR 118 47.558 64.399 74.779 1.00 0.00 C

ATOM 1929 HA TYR 118 46.852 64.396 75.610 1.00 0.00 H

ATOM 1930 CB TYR 118 47.565 62.943 74.266 1.00 0.00 C

ATOM 1931 HB2 TYR 118 48.188 62.990 73.373 1.00 0.00 H

ATOM 1932 HB3 TYR 118 48.035 62.333 75.038 1.00 0.00 H

ATOM 1933 CG TYR 118 46.208 62.374 73.982 1.00 0.00 C

ATOM 1934 CD1 TYR 118 45.876 61.815 72.768 1.00 0.00 C

ATOM 1935 HD1 TYR 118 46.553 61.862 71.927 1.00 0.00 H

ATOM 1936 CE1 TYR 118 44.604 61.322 72.461 1.00 0.00 C

ATOM 1937 HE1 TYR 118 44.282 60.928 71.509 1.00 0.00 H

ATOM 1938 CZ TYR 118 43.647 61.302 73.516 1.00 0.00 C

ATOM 1939 OH TYR 118 42.454 60.783 73.162 1.00 0.00 O

ATOM 1940 HH TYR 118 42.406 60.505 72.245 1.00 0.00 H

ATOM 1941 CE2 TYR 118 43.917 61.741 74.816 1.00 0.00 C

ATOM 1942 HE2 TYR 118 43.057 61.810 75.465 1.00 0.00 H

ATOM 1943 CD2 TYR 118 45.199 62.354 75.007 1.00 0.00 C

ATOM 1944 HD2 TYR 118 45.325 62.671 76.032 1.00 0.00 H

ATOM 1945 C TYR 118 48.852 64.903 75.366 1.00 0.00 C

ATOM 1946 O TYR 118 49.778 64.161 75.480 1.00 0.00 O

ATOM 1947 N PRO 119 48.944 66.235 75.565 1.00 0.00 N

ATOM 1948 CD PRO 119 47.924 67.129 76.136 1.00 0.00 C

ATOM 1949 HD2 PRO 119 47.470 66.863 77.091 1.00 0.00 H

ATOM 1950 HD3 PRO 119 47.151 67.293 75.386 1.00 0.00 H

ATOM 1951 CG PRO 119 48.522 68.501 76.411 1.00 0.00 C

ATOM 1952 HG2 PRO 119 48.468 68.804 77.457 1.00 0.00 H

ATOM 1953 HG3 PRO 119 47.923 69.199 75.826 1.00 0.00 H

ATOM 1954 CB PRO 119 49.983 68.412 75.941 1.00 0.00 C

ATOM 1955 HB2 PRO 119 50.570 68.896 76.722 1.00 0.00 H

ATOM 1956 HB3 PRO 119 50.069 69.006 75.031 1.00 0.00 H

ATOM 1957 CA PRO 119 50.235 66.911 75.587 1.00 0.00 C

ATOM 1958 HA PRO 119 50.665 66.841 74.587 1.00 0.00 H

ATOM 1959 C PRO 119 51.298 66.300 76.538 1.00 0.00 C

ATOM 1960 O PRO 119 52.384 65.949 76.003 1.00 0.00 O

ATOM 1961 N LEU 120 51.165 66.258 77.874 1.00 0.00 N

ATOM 1962 H LEU 120 50.258 66.312 78.314 1.00 0.00 H

ATOM 1963 CA LEU 120 52.199 65.752 78.770 1.00 0.00 C

ATOM 1964 HA LEU 120 53.028 66.423 78.544 1.00 0.00 H

ATOM 1965 CB LEU 120 51.785 65.901 80.232 1.00 0.00 C

ATOM 1966 HB2 LEU 120 51.710 66.985 80.319 1.00 0.00 H

ATOM 1967 HB3 LEU 120 50.756 65.545 80.289 1.00 0.00 H

ATOM 1968 CG LEU 120 52.713 65.409 81.376 1.00 0.00 C

ATOM 1969 HG LEU 120 53.110 64.417 81.156 1.00 0.00 H

ATOM 1970 CD1 LEU 120 53.916 66.339 81.547 1.00 0.00 C

ATOM 1971 HD11 LEU 120 54.527 65.968 82.370 1.00 0.00 H

ATOM 1972 HD12 LEU 120 54.501 66.408 80.631 1.00 0.00 H

ATOM 1973 HD13 LEU 120 53.502 67.279 81.913 1.00 0.00 H

ATOM 1974 CD2 LEU 120 51.998 65.472 82.718 1.00 0.00 C

ATOM 1975 HD21 LEU 120 51.491 64.514 82.827 1.00 0.00 H

ATOM 1976 HD22 LEU 120 52.738 65.607 83.507 1.00 0.00 H

ATOM 1977 HD23 LEU 120 51.358 66.354 82.709 1.00 0.00 H

ATOM 1978 C LEU 120 52.741 64.357 78.417 1.00 0.00 C

ATOM 1979 O LEU 120 53.907 63.978 78.643 1.00 0.00 O

ATOM 1980 N LYS 121 51.876 63.539 77.853 1.00 0.00 N

ATOM 1981 H LYS 121 50.901 63.801 77.830 1.00 0.00 H

ATOM 1982 CA LYS 121 52.180 62.204 77.483 1.00 0.00 C

ATOM 1983 HA LYS 121 52.956 61.856 78.164 1.00 0.00 H

ATOM 1984 CB LYS 121 50.947 61.263 77.727 1.00 0.00 C

ATOM 1985 HB2 LYS 121 51.440 60.303 77.576 1.00 0.00 H

ATOM 1986 HB3 LYS 121 50.617 61.346 78.763 1.00 0.00 H

ATOM 1987 CG LYS 121 49.707 61.464 76.907 1.00 0.00 C

ATOM 1988 HG2 LYS 121 49.190 62.288 77.399 1.00 0.00 H

ATOM 1989 HG3 LYS 121 49.961 61.495 75.847 1.00 0.00 H

ATOM 1990 CD LYS 121 48.921 60.092 77.162 1.00 0.00 C

ATOM 1991 HD2 LYS 121 48.256 59.864 76.328 1.00 0.00 H

ATOM 1992 HD3 LYS 121 49.600 59.248 77.278 1.00 0.00 H

ATOM 1993 CE LYS 121 48.071 60.199 78.417 1.00 0.00 C

ATOM 1994 HE2 LYS 121 48.486 60.998 79.031 1.00 0.00 H

ATOM 1995 HE3 LYS 121 47.057 60.581 78.308 1.00 0.00 H

ATOM 1996 NZ LYS 121 48.088 58.969 79.181 1.00 0.00 N

ATOM 1997 HZ1 LYS 121 49.001 58.538 79.219 1.00 0.00 H

ATOM 1998 HZ2 LYS 121 47.773 59.033 80.138 1.00 0.00 H

ATOM 1999 HZ3 LYS 121 47.462 58.250 78.846 1.00 0.00 H

ATOM 2000 C LYS 121 52.824 62.067 76.086 1.00 0.00 C

ATOM 2001 O LYS 121 53.212 60.945 75.687 1.00 0.00 O

ATOM 2002 N SER 122 52.912 63.186 75.386 1.00 0.00 N

ATOM 2003 H SER 122 52.545 64.054 75.750 1.00 0.00 H

ATOM 2004 CA SER 122 53.409 63.213 74.018 1.00 0.00 C

ATOM 2005 HA SER 122 53.715 62.199 73.760 1.00 0.00 H

ATOM 2006 CB SER 122 52.282 63.619 73.104 1.00 0.00 C

ATOM 2007 HB2 SER 122 52.049 64.674 73.247 1.00 0.00 H

ATOM 2008 HB3 SER 122 52.491 63.511 72.040 1.00 0.00 H

ATOM 2009 OG SER 122 51.081 62.823 73.276 1.00 0.00 O

ATOM 2010 HG SER 122 50.700 63.090 74.115 1.00 0.00 H

ATOM 2011 C SER 122 54.593 64.149 73.725 1.00 0.00 C

ATOM 2012 O SER 122 55.167 63.869 72.729 1.00 0.00 O

ATOM 2013 N LEU 123 54.762 65.246 74.501 1.00 0.00 N

ATOM 2014 H LEU 123 54.194 65.513 75.292 1.00 0.00 H

ATOM 2015 CA LEU 123 55.803 66.240 74.182 1.00 0.00 C

ATOM 2016 HA LEU 123 55.662 66.752 73.230 1.00 0.00 H

ATOM 2017 CB LEU 123 55.724 67.235 75.330 1.00 0.00 C

ATOM 2018 HB2 LEU 123 55.550 66.666 76.243 1.00 0.00 H

ATOM 2019 HB3 LEU 123 56.660 67.758 75.526 1.00 0.00 H

ATOM 2020 CG LEU 123 54.526 68.172 75.134 1.00 0.00 C

ATOM 2021 HG LEU 123 53.790 67.608 74.561 1.00 0.00 H

ATOM 2022 CD1 LEU 123 53.973 68.679 76.425 1.00 0.00 C

ATOM 2023 HD11 LEU 123 53.690 67.759 76.937 1.00 0.00 H

ATOM 2024 HD12 LEU 123 54.717 69.222 77.008 1.00 0.00 H

ATOM 2025 HD13 LEU 123 53.126 69.322 76.188 1.00 0.00 H

ATOM 2026 CD2 LEU 123 54.807 69.337 74.186 1.00 0.00 C

ATOM 2027 HD21 LEU 123 55.335 69.038 73.281 1.00 0.00 H

ATOM 2028 HD22 LEU 123 53.845 69.810 73.989 1.00 0.00 H

ATOM 2029 HD23 LEU 123 55.571 69.950 74.664 1.00 0.00 H

ATOM 2030 C LEU 123 57.231 65.606 74.048 1.00 0.00 C

ATOM 2031 O LEU 123 57.974 65.941 73.135 1.00 0.00 O

ATOM 2032 N GLY 124 57.443 64.677 74.965 1.00 0.00 N

ATOM 2033 H GLY 124 56.708 64.392 75.597 1.00 0.00 H

ATOM 2034 CA GLY 124 58.760 63.959 74.978 1.00 0.00 C

ATOM 2035 HA2 GLY 124 59.479 64.374 74.272 1.00 0.00 H

ATOM 2036 HA3 GLY 124 59.133 64.246 75.961 1.00 0.00 H

ATOM 2037 C GLY 124 58.621 62.470 74.841 1.00 0.00 C

ATOM 2038 O GLY 124 58.912 61.693 75.731 1.00 0.00 O

ATOM 2039 N ARG 125 58.108 62.031 73.685 1.00 0.00 N

ATOM 2040 H ARG 125 57.947 62.718 72.963 1.00 0.00 H

ATOM 2041 CA ARG 125 57.536 60.668 73.481 1.00 0.00 C

ATOM 2042 HA ARG 125 57.294 60.344 74.493 1.00 0.00 H

ATOM 2043 CB ARG 125 56.219 60.840 72.689 1.00 0.00 C

ATOM 2044 HB2 ARG 125 55.550 61.483 73.261 1.00 0.00 H

ATOM 2045 HB3 ARG 125 56.488 61.352 71.764 1.00 0.00 H

ATOM 2046 CG ARG 125 55.544 59.436 72.393 1.00 0.00 C

ATOM 2047 HG2 ARG 125 54.719 59.475 71.682 1.00 0.00 H

ATOM 2048 HG3 ARG 125 56.295 58.702 72.100 1.00 0.00 H

ATOM 2049 CD ARG 125 54.919 58.865 73.717 1.00 0.00 C

ATOM 2050 HD2 ARG 125 55.723 58.388 74.277 1.00 0.00 H

ATOM 2051 HD3 ARG 125 54.505 59.725 74.245 1.00 0.00 H

ATOM 2052 NE ARG 125 53.936 57.877 73.428 1.00 0.00 N

ATOM 2053 HE ARG 125 54.382 57.315 72.717 1.00 0.00 H

ATOM 2054 CZ ARG 125 52.626 58.011 73.438 1.00 0.00 C

ATOM 2055 NH1 ARG 125 51.820 57.215 72.774 1.00 0.00 N

ATOM 2056 HH11 ARG 125 52.087 56.854 71.869 1.00 0.00 H

ATOM 2057 HH12 ARG 125 51.031 56.839 73.280 1.00 0.00 H

ATOM 2058 NH2 ARG 125 52.007 58.660 74.402 1.00 0.00 N

ATOM 2059 HH21 ARG 125 51.037 58.871 74.214 1.00 0.00 H

ATOM 2060 HH22 ARG 125 52.519 59.262 75.030 1.00 0.00 H

ATOM 2061 C ARG 125 58.559 59.613 72.917 1.00 0.00 C

ATOM 2062 O ARG 125 58.869 58.683 73.623 1.00 0.00 O

ATOM 2063 N LEU 126 58.878 59.662 71.631 1.00 0.00 N

ATOM 2064 H LEU 126 58.705 60.515 71.118 1.00 0.00 H

ATOM 2065 CA LEU 126 59.668 58.697 70.834 1.00 0.00 C

ATOM 2066 HA LEU 126 59.589 57.727 71.324 1.00 0.00 H

ATOM 2067 CB LEU 126 59.004 58.587 69.391 1.00 0.00 C

ATOM 2068 HB2 LEU 126 57.951 58.353 69.548 1.00 0.00 H

ATOM 2069 HB3 LEU 126 59.045 59.608 69.012 1.00 0.00 H

ATOM 2070 CG LEU 126 59.709 57.610 68.462 1.00 0.00 C

ATOM 2071 HG LEU 126 60.253 56.949 69.137 1.00 0.00 H

ATOM 2072 CD1 LEU 126 58.727 56.686 67.776 1.00 0.00 C

ATOM 2073 HD11 LEU 126 58.117 56.120 68.480 1.00 0.00 H

ATOM 2074 HD12 LEU 126 58.052 57.280 67.160 1.00 0.00 H

ATOM 2075 HD13 LEU 126 59.254 55.981 67.133 1.00 0.00 H

ATOM 2076 CD2 LEU 126 60.548 58.249 67.385 1.00 0.00 C

ATOM 2077 HD21 LEU 126 61.232 57.507 66.975 1.00 0.00 H

ATOM 2078 HD22 LEU 126 59.943 58.544 66.528 1.00 0.00 H

ATOM 2079 HD23 LEU 126 61.052 59.184 67.632 1.00 0.00 H

ATOM 2080 C LEU 126 61.245 58.916 70.948 1.00 0.00 C

ATOM 2081 O LEU 126 61.686 60.030 71.060 1.00 0.00 O

ATOM 2082 N LYS 127 62.070 57.825 71.075 1.00 0.00 N

ATOM 2083 H LYS 127 61.637 56.928 71.239 1.00 0.00 H

ATOM 2084 CA LYS 127 63.487 58.022 71.366 1.00 0.00 C

ATOM 2085 HA LYS 127 63.761 58.916 71.925 1.00 0.00 H

ATOM 2086 CB LYS 127 64.101 56.835 72.198 1.00 0.00 C

ATOM 2087 HB2 LYS 127 64.503 56.062 71.542 1.00 0.00 H

ATOM 2088 HB3 LYS 127 65.084 57.172 72.528 1.00 0.00 H

ATOM 2089 CG LYS 127 63.244 56.257 73.383 1.00 0.00 C

ATOM 2090 HG2 LYS 127 62.300 55.854 73.017 1.00 0.00 H

ATOM 2091 HG3 LYS 127 63.766 55.399 73.807 1.00 0.00 H

ATOM 2092 CD LYS 127 62.958 57.247 74.485 1.00 0.00 C

ATOM 2093 HD2 LYS 127 63.607 57.012 75.328 1.00 0.00 H

ATOM 2094 HD3 LYS 127 63.317 58.233 74.190 1.00 0.00 H

ATOM 2095 CE LYS 127 61.461 57.410 74.823 1.00 0.00 C

ATOM 2096 HE2 LYS 127 60.850 57.051 73.996 1.00 0.00 H

ATOM 2097 HE3 LYS 127 61.139 56.728 75.610 1.00 0.00 H

ATOM 2098 NZ LYS 127 61.207 58.776 75.304 1.00 0.00 N

ATOM 2099 HZ1 LYS 127 60.212 58.823 75.467 1.00 0.00 H

ATOM 2100 HZ2 LYS 127 61.652 58.884 76.204 1.00 0.00 H

ATOM 2101 HZ3 LYS 127 61.493 59.486 74.645 1.00 0.00 H

ATOM 2102 C LYS 127 64.240 58.242 69.998 1.00 0.00 C

ATOM 2103 O LYS 127 63.785 57.942 68.936 1.00 0.00 O

ATOM 2104 N LYS 128 65.478 58.747 70.073 1.00 0.00 N

ATOM 2105 H LYS 128 65.832 59.042 70.972 1.00 0.00 H

ATOM 2106 CA LYS 128 66.198 59.170 68.830 1.00 0.00 C

ATOM 2107 HA LYS 128 65.454 59.577 68.145 1.00 0.00 H

ATOM 2108 CB LYS 128 67.288 60.230 69.074 1.00 0.00 C

ATOM 2109 HB2 LYS 128 67.729 60.619 68.157 1.00 0.00 H

ATOM 2110 HB3 LYS 128 66.860 61.095 69.580 1.00 0.00 H

ATOM 2111 CG LYS 128 68.276 59.848 70.142 1.00 0.00 C

ATOM 2112 HG2 LYS 128 67.877 59.854 71.156 1.00 0.00 H

ATOM 2113 HG3 LYS 128 68.567 58.799 70.091 1.00 0.00 H

ATOM 2114 CD LYS 128 69.469 60.817 70.113 1.00 0.00 C

ATOM 2115 HD2 LYS 128 69.915 60.710 69.124 1.00 0.00 H

ATOM 2116 HD3 LYS 128 69.129 61.847 70.222 1.00 0.00 H

ATOM 2117 CE LYS 128 70.535 60.467 71.107 1.00 0.00 C

ATOM 2118 HE2 LYS 128 70.507 59.437 71.463 1.00 0.00 H

ATOM 2119 HE3 LYS 128 71.505 60.461 70.609 1.00 0.00 H

ATOM 2120 NZ LYS 128 70.606 61.380 72.263 1.00 0.00 N

ATOM 2121 HZ1 LYS 128 71.286 61.002 72.906 1.00 0.00 H

ATOM 2122 HZ2 LYS 128 70.844 62.338 72.048 1.00 0.00 H

ATOM 2123 HZ3 LYS 128 69.709 61.542 72.699 1.00 0.00 H

ATOM 2124 C LYS 128 66.734 58.029 67.929 1.00 0.00 C

ATOM 2125 O LYS 128 66.740 58.139 66.690 1.00 0.00 O

ATOM 2126 N LYS 129 67.065 56.847 68.469 1.00 0.00 N

ATOM 2127 H LYS 129 67.137 56.859 69.477 1.00 0.00 H

ATOM 2128 CA LYS 129 67.305 55.590 67.719 1.00 0.00 C

ATOM 2129 HA LYS 129 68.177 55.706 67.075 1.00 0.00 H

ATOM 2130 CB LYS 129 67.665 54.538 68.817 1.00 0.00 C

ATOM 2131 HB2 LYS 129 68.638 54.834 69.210 1.00 0.00 H

ATOM 2132 HB3 LYS 129 66.878 54.612 69.567 1.00 0.00 H

ATOM 2133 CG LYS 129 67.675 53.071 68.342 1.00 0.00 C

ATOM 2134 HG2 LYS 129 66.673 52.681 68.161 1.00 0.00 H

ATOM 2135 HG3 LYS 129 68.224 53.007 67.402 1.00 0.00 H

ATOM 2136 CD LYS 129 68.503 52.171 69.267 1.00 0.00 C

ATOM 2137 HD2 LYS 129 68.438 51.126 68.963 1.00 0.00 H

ATOM 2138 HD3 LYS 129 69.545 52.428 69.076 1.00 0.00 H

ATOM 2139 CE LYS 129 68.193 52.196 70.755 1.00 0.00 C

ATOM 2140 HE2 LYS 129 68.034 53.220 71.094 1.00 0.00 H

ATOM 2141 HE3 LYS 129 67.222 51.749 70.969 1.00 0.00 H

ATOM 2142 NZ LYS 129 69.231 51.638 71.654 1.00 0.00 N

ATOM 2143 HZ1 LYS 129 68.961 51.855 72.603 1.00 0.00 H

ATOM 2144 HZ2 LYS 129 69.281 50.656 71.423 1.00 0.00 H

ATOM 2145 HZ3 LYS 129 70.078 52.181 71.568 1.00 0.00 H

ATOM 2146 C LYS 129 66.129 55.082 66.909 1.00 0.00 C

ATOM 2147 O LYS 129 66.370 54.887 65.750 1.00 0.00 O

ATOM 2148 N ASN 130 64.922 55.098 67.441 1.00 0.00 N

ATOM 2149 H ASN 130 64.814 55.249 68.434 1.00 0.00 H

ATOM 2150 CA ASN 130 63.711 54.780 66.671 1.00 0.00 C

ATOM 2151 HA ASN 130 63.807 53.736 66.376 1.00 0.00 H

ATOM 2152 CB ASN 130 62.432 54.908 67.564 1.00 0.00 C

ATOM 2153 HB2 ASN 130 62.269 55.936 67.887 1.00 0.00 H

ATOM 2154 HB3 ASN 130 61.501 54.620 67.077 1.00 0.00 H

ATOM 2155 CG ASN 130 62.581 54.097 68.886 1.00 0.00 C

ATOM 2156 OD1 ASN 130 63.501 54.116 69.615 1.00 0.00 O

ATOM 2157 ND2 ASN 130 61.493 53.427 69.321 1.00 0.00 N

ATOM 2158 HD21 ASN 130 61.879 52.711 69.919 1.00 0.00 H

ATOM 2159 HD22 ASN 130 60.835 53.110 68.623 1.00 0.00 H

ATOM 2160 C ASN 130 63.472 55.688 65.435 1.00 0.00 C

ATOM 2161 O ASN 130 63.349 55.312 64.271 1.00 0.00 O

ATOM 2162 N ALA 131 63.504 57.011 65.710 1.00 0.00 N

ATOM 2163 H ALA 131 63.366 57.356 66.649 1.00 0.00 H

ATOM 2164 CA ALA 131 63.618 58.061 64.698 1.00 0.00 C

ATOM 2165 HA ALA 131 62.655 58.070 64.187 1.00 0.00 H

ATOM 2166 CB ALA 131 63.743 59.448 65.314 1.00 0.00 C

ATOM 2167 HB1 ALA 131 63.476 60.242 64.617 1.00 0.00 H

ATOM 2168 HB2 ALA 131 63.058 59.554 66.156 1.00 0.00 H

ATOM 2169 HB3 ALA 131 64.766 59.679 65.611 1.00 0.00 H

ATOM 2170 C ALA 131 64.630 57.783 63.645 1.00 0.00 C

ATOM 2171 O ALA 131 64.396 58.097 62.467 1.00 0.00 O

ATOM 2172 N ILE 132 65.862 57.337 63.993 1.00 0.00 N

ATOM 2173 H ILE 132 66.236 57.534 64.910 1.00 0.00 H

ATOM 2174 CA ILE 132 66.851 56.909 63.011 1.00 0.00 C

ATOM 2175 HA ILE 132 66.926 57.718 62.284 1.00 0.00 H

ATOM 2176 CB ILE 132 68.340 56.724 63.502 1.00 0.00 C

ATOM 2177 HB ILE 132 68.391 55.898 64.212 1.00 0.00 H

ATOM 2178 CG2 ILE 132 69.295 56.422 62.296 1.00 0.00 C

ATOM 2179 HG21 ILE 132 69.312 57.212 61.545 1.00 0.00 H

ATOM 2180 HG22 ILE 132 70.312 56.174 62.597 1.00 0.00 H

ATOM 2181 HG23 ILE 132 68.863 55.508 61.887 1.00 0.00 H

ATOM 2182 CG1 ILE 132 68.874 58.048 64.120 1.00 0.00 C

ATOM 2183 HG12 ILE 132 69.363 58.640 63.346 1.00 0.00 H

ATOM 2184 HG13 ILE 132 68.024 58.567 64.563 1.00 0.00 H

ATOM 2185 CD1 ILE 132 69.841 57.922 65.226 1.00 0.00 C

ATOM 2186 HD11 ILE 132 69.383 57.436 66.087 1.00 0.00 H

ATOM 2187 HD12 ILE 132 70.759 57.441 64.888 1.00 0.00 H

ATOM 2188 HD13 ILE 132 70.143 58.916 65.554 1.00 0.00 H

ATOM 2189 C ILE 132 66.354 55.690 62.227 1.00 0.00 C

ATOM 2190 O ILE 132 66.260 55.674 60.994 1.00 0.00 O

ATOM 2191 N CYX 133 65.991 54.568 62.834 1.00 0.00 N

ATOM 2192 H CYX 133 66.174 54.495 63.825 1.00 0.00 H

ATOM 2193 CA CYX 133 65.492 53.367 62.161 1.00 0.00 C

ATOM 2194 HA CYX 133 66.298 52.954 61.555 1.00 0.00 H

ATOM 2195 CB CYX 133 65.114 52.279 63.176 1.00 0.00 C

ATOM 2196 HB2 CYX 133 64.155 52.507 63.639 1.00 0.00 H

ATOM 2197 HB3 CYX 133 64.924 51.302 62.733 1.00 0.00 H

ATOM 2198 SG CYX 133 66.358 51.979 64.559 1.00 0.00 S

ATOM 2199 C CYX 133 64.316 53.667 61.243 1.00 0.00 C

ATOM 2200 O CYX 133 64.312 52.949 60.218 1.00 0.00 O

ATOM 2201 N ILE 134 63.344 54.517 61.613 1.00 0.00 N

ATOM 2202 H ILE 134 63.530 54.993 62.484 1.00 0.00 H

ATOM 2203 CA ILE 134 62.265 55.151 60.878 1.00 0.00 C

ATOM 2204 HA ILE 134 61.727 54.303 60.454 1.00 0.00 H

ATOM 2205 CB ILE 134 61.273 55.912 61.850 1.00 0.00 C

ATOM 2206 HB ILE 134 61.833 56.596 62.487 1.00 0.00 H

ATOM 2207 CG2 ILE 134 60.247 56.735 60.962 1.00 0.00 C

ATOM 2208 HG21 ILE 134 59.347 57.074 61.475 1.00 0.00 H

ATOM 2209 HG22 ILE 134 60.641 57.681 60.590 1.00 0.00 H

ATOM 2210 HG23 ILE 134 59.888 55.985 60.257 1.00 0.00 H

ATOM 2211 CG1 ILE 134 60.450 54.959 62.727 1.00 0.00 C

ATOM 2212 HG12 ILE 134 59.794 54.350 62.105 1.00 0.00 H

ATOM 2213 HG13 ILE 134 61.249 54.330 63.119 1.00 0.00 H

ATOM 2214 CD1 ILE 134 59.688 55.597 63.969 1.00 0.00 C

ATOM 2215 HD11 ILE 134 59.063 54.919 64.550 1.00 0.00 H

ATOM 2216 HD12 ILE 134 60.449 55.983 64.647 1.00 0.00 H

ATOM 2217 HD13 ILE 134 59.003 56.343 63.565 1.00 0.00 H

ATOM 2218 C ILE 134 62.870 55.915 59.657 1.00 0.00 C

ATOM 2219 O ILE 134 62.601 55.562 58.551 1.00 0.00 O

ATOM 2220 N SER 135 63.729 56.916 59.859 1.00 0.00 N

ATOM 2221 H SER 135 64.001 57.017 60.827 1.00 0.00 H

ATOM 2222 CA SER 135 64.260 57.835 58.832 1.00 0.00 C

ATOM 2223 HA SER 135 63.527 58.508 58.388 1.00 0.00 H

ATOM 2224 CB SER 135 65.249 58.760 59.460 1.00 0.00 C

ATOM 2225 HB2 SER 135 66.026 58.272 60.048 1.00 0.00 H

ATOM 2226 HB3 SER 135 65.721 59.459 58.770 1.00 0.00 H

ATOM 2227 OG SER 135 64.561 59.728 60.290 1.00 0.00 O

ATOM 2228 HG SER 135 64.400 59.389 61.174 1.00 0.00 H

ATOM 2229 C SER 135 64.980 57.056 57.741 1.00 0.00 C

ATOM 2230 O SER 135 64.769 57.274 56.583 1.00 0.00 O

ATOM 2231 N VAL 136 65.807 56.053 58.092 1.00 0.00 N

ATOM 2232 H VAL 136 65.852 55.825 59.075 1.00 0.00 H

ATOM 2233 CA VAL 136 66.400 55.082 57.188 1.00 0.00 C

ATOM 2234 HA VAL 136 67.116 55.618 56.564 1.00 0.00 H

ATOM 2235 CB VAL 136 67.109 53.974 58.048 1.00 0.00 C

ATOM 2236 HB VAL 136 66.409 53.652 58.819 1.00 0.00 H

ATOM 2237 CG1 VAL 136 67.606 52.905 57.153 1.00 0.00 C

ATOM 2238 HG11 VAL 136 68.297 53.406 56.475 1.00 0.00 H

ATOM 2239 HG12 VAL 136 68.105 52.146 57.756 1.00 0.00 H

ATOM 2240 HG13 VAL 136 66.850 52.342 56.607 1.00 0.00 H

ATOM 2241 CG2 VAL 136 68.418 54.616 58.669 1.00 0.00 C

ATOM 2242 HG21 VAL 136 69.261 54.242 58.088 1.00 0.00 H

ATOM 2243 HG22 VAL 136 68.530 55.700 58.648 1.00 0.00 H

ATOM 2244 HG23 VAL 136 68.414 54.289 59.708 1.00 0.00 H

ATOM 2245 C VAL 136 65.402 54.367 56.301 1.00 0.00 C

ATOM 2246 O VAL 136 65.637 54.217 55.106 1.00 0.00 O

ATOM 2247 N LEU 137 64.411 53.737 56.897 1.00 0.00 N

ATOM 2248 H LEU 137 64.134 53.922 57.850 1.00 0.00 H

ATOM 2249 CA LEU 137 63.317 53.040 56.249 1.00 0.00 C

ATOM 2250 HA LEU 137 63.780 52.189 55.750 1.00 0.00 H

ATOM 2251 CB LEU 137 62.382 52.470 57.383 1.00 0.00 C

ATOM 2252 HB2 LEU 137 62.985 52.024 58.175 1.00 0.00 H

ATOM 2253 HB3 LEU 137 61.819 53.339 57.722 1.00 0.00 H

ATOM 2254 CG LEU 137 61.400 51.324 56.881 1.00 0.00 C

ATOM 2255 HG LEU 137 60.789 51.808 56.120 1.00 0.00 H

ATOM 2256 CD1 LEU 137 62.103 50.098 56.294 1.00 0.00 C

ATOM 2257 HD11 LEU 137 62.664 49.431 56.948 1.00 0.00 H

ATOM 2258 HD12 LEU 137 61.359 49.410 55.892 1.00 0.00 H

ATOM 2259 HD13 LEU 137 62.637 50.479 55.423 1.00 0.00 H

ATOM 2260 CD2 LEU 137 60.663 50.709 58.056 1.00 0.00 C

ATOM 2261 HD21 LEU 137 61.468 50.574 58.779 1.00 0.00 H

ATOM 2262 HD22 LEU 137 59.890 51.379 58.431 1.00 0.00 H

ATOM 2263 HD23 LEU 137 60.180 49.761 57.822 1.00 0.00 H

ATOM 2264 C LEU 137 62.502 53.899 55.228 1.00 0.00 C

ATOM 2265 O LEU 137 61.878 53.301 54.368 1.00 0.00 O

ATOM 2266 N VAL 138 62.317 55.249 55.443 1.00 0.00 N

ATOM 2267 H VAL 138 62.810 55.658 56.224 1.00 0.00 H

ATOM 2268 CA VAL 138 61.492 56.076 54.593 1.00 0.00 C

ATOM 2269 HA VAL 138 60.615 55.488 54.324 1.00 0.00 H

ATOM 2270 CB VAL 138 60.903 57.297 55.378 1.00 0.00 C

ATOM 2271 HB VAL 138 60.135 56.939 56.064 1.00 0.00 H

ATOM 2272 CG1 VAL 138 61.941 58.240 56.132 1.00 0.00 C

ATOM 2273 HG11 VAL 138 61.349 59.037 56.582 1.00 0.00 H

ATOM 2274 HG12 VAL 138 62.267 57.500 56.863 1.00 0.00 H

ATOM 2275 HG13 VAL 138 62.749 58.445 55.429 1.00 0.00 H

ATOM 2276 CG2 VAL 138 60.200 58.246 54.387 1.00 0.00 C

ATOM 2277 HG21 VAL 138 59.731 59.045 54.961 1.00 0.00 H

ATOM 2278 HG22 VAL 138 60.872 58.745 53.690 1.00 0.00 H

ATOM 2279 HG23 VAL 138 59.387 57.820 53.800 1.00 0.00 H

ATOM 2280 C VAL 138 62.294 56.439 53.309 1.00 0.00 C

ATOM 2281 O VAL 138 61.844 56.142 52.162 1.00 0.00 O

ATOM 2282 N TRP 139 63.525 56.942 53.417 1.00 0.00 N

ATOM 2283 H TRP 139 63.889 57.175 54.330 1.00 0.00 H

ATOM 2284 CA TRP 139 64.422 57.196 52.366 1.00 0.00 C

ATOM 2285 HA TRP 139 63.888 57.889 51.716 1.00 0.00 H

ATOM 2286 CB TRP 139 65.785 57.622 52.846 1.00 0.00 C

ATOM 2287 HB2 TRP 139 66.169 56.864 53.529 1.00 0.00 H

ATOM 2288 HB3 TRP 139 66.420 57.793 51.977 1.00 0.00 H

ATOM 2289 CG TRP 139 65.981 58.934 53.524 1.00 0.00 C

ATOM 2290 CD1 TRP 139 66.489 59.174 54.744 1.00 0.00 C

ATOM 2291 HD1 TRP 139 66.749 58.440 55.492 1.00 0.00 H

ATOM 2292 NE1 TRP 139 66.848 60.509 54.868 1.00 0.00 N

ATOM 2293 HE1 TRP 139 67.320 60.835 55.699 1.00 0.00 H

ATOM 2294 CE2 TRP 139 66.314 61.233 53.812 1.00 0.00 C

ATOM 2295 CZ2 TRP 139 66.453 62.601 53.485 1.00 0.00 C

ATOM 2296 HZ2 TRP 139 66.961 63.315 54.118 1.00 0.00 H

ATOM 2297 CH2 TRP 139 65.841 63.016 52.242 1.00 0.00 C

ATOM 2298 HH2 TRP 139 65.969 64.046 51.945 1.00 0.00 H

ATOM 2299 CZ3 TRP 139 65.208 62.066 51.395 1.00 0.00 C

ATOM 2300 HZ3 TRP 139 64.559 62.505 50.651 1.00 0.00 H

ATOM 2301 CE3 TRP 139 65.204 60.706 51.720 1.00 0.00 C

ATOM 2302 HE3 TRP 139 64.659 60.084 51.026 1.00 0.00 H

ATOM 2303 CD2 TRP 139 65.826 60.219 52.870 1.00 0.00 C

ATOM 2304 C TRP 139 64.737 55.917 51.506 1.00 0.00 C

ATOM 2305 O TRP 139 64.532 56.011 50.343 1.00 0.00 O

ATOM 2306 N LEU 140 64.985 54.774 52.121 1.00 0.00 N

ATOM 2307 H LEU 140 65.080 54.798 53.126 1.00 0.00 H

ATOM 2308 CA LEU 140 65.005 53.451 51.412 1.00 0.00 C

ATOM 2309 HA LEU 140 65.916 53.329 50.827 1.00 0.00 H

ATOM 2310 CB LEU 140 65.071 52.358 52.435 1.00 0.00 C

ATOM 2311 HB2 LEU 140 65.773 52.645 53.218 1.00 0.00 H

ATOM 2312 HB3 LEU 140 64.128 52.408 52.981 1.00 0.00 H

ATOM 2313 CG LEU 140 65.417 50.906 52.064 1.00 0.00 C

ATOM 2314 HG LEU 140 65.676 50.350 52.965 1.00 0.00 H

ATOM 2315 CD1 LEU 140 64.219 50.174 51.501 1.00 0.00 C

ATOM 2316 HD11 LEU 140 63.907 50.641 50.567 1.00 0.00 H

ATOM 2317 HD12 LEU 140 64.583 49.154 51.378 1.00 0.00 H

ATOM 2318 HD13 LEU 140 63.508 50.320 52.315 1.00 0.00 H

ATOM 2319 CD2 LEU 140 66.556 50.860 50.953 1.00 0.00 C

ATOM 2320 HD21 LEU 140 66.146 51.036 49.959 1.00 0.00 H

ATOM 2321 HD22 LEU 140 67.385 51.488 51.281 1.00 0.00 H

ATOM 2322 HD23 LEU 140 66.889 49.823 50.974 1.00 0.00 H

ATOM 2323 C LEU 140 63.793 53.237 50.439 1.00 0.00 C

ATOM 2324 O LEU 140 63.944 53.169 49.225 1.00 0.00 O

ATOM 2325 N ILE 141 62.547 53.571 50.840 1.00 0.00 N

ATOM 2326 H ILE 141 62.498 53.696 51.841 1.00 0.00 H

ATOM 2327 CA ILE 141 61.328 53.310 50.100 1.00 0.00 C

ATOM 2328 HA ILE 141 61.570 52.512 49.398 1.00 0.00 H

ATOM 2329 CB ILE 141 60.080 52.808 50.930 1.00 0.00 C

ATOM 2330 HB ILE 141 59.354 52.667 50.129 1.00 0.00 H

ATOM 2331 CG2 ILE 141 60.407 51.438 51.564 1.00 0.00 C

ATOM 2332 HG21 ILE 141 59.591 50.751 51.790 1.00 0.00 H

ATOM 2333 HG22 ILE 141 60.916 50.890 50.771 1.00 0.00 H

ATOM 2334 HG23 ILE 141 61.106 51.715 52.353 1.00 0.00 H

ATOM 2335 CG1 ILE 141 59.509 53.757 51.920 1.00 0.00 C

ATOM 2336 HG12 ILE 141 59.964 53.578 52.894 1.00 0.00 H

ATOM 2337 HG13 ILE 141 59.840 54.734 51.568 1.00 0.00 H

ATOM 2338 CD1 ILE 141 57.975 53.684 52.171 1.00 0.00 C

ATOM 2339 HD11 ILE 141 57.721 52.719 52.609 1.00 0.00 H

ATOM 2340 HD12 ILE 141 57.819 54.421 52.959 1.00 0.00 H

ATOM 2341 HD13 ILE 141 57.376 53.806 51.268 1.00 0.00 H

ATOM 2342 C ILE 141 61.117 54.523 49.092 1.00 0.00 C

ATOM 2343 O ILE 141 60.479 54.307 48.091 1.00 0.00 O

ATOM 2344 N VAL 142 61.632 55.731 49.315 1.00 0.00 N

ATOM 2345 H VAL 142 62.139 55.847 50.180 1.00 0.00 H

ATOM 2346 CA VAL 142 61.657 56.815 48.316 1.00 0.00 C

ATOM 2347 HA VAL 142 60.769 56.890 47.689 1.00 0.00 H

ATOM 2348 CB VAL 142 61.795 58.213 49.022 1.00 0.00 C

ATOM 2349 HB VAL 142 62.480 58.211 49.870 1.00 0.00 H

ATOM 2350 CG1 VAL 142 62.394 59.306 48.163 1.00 0.00 C

ATOM 2351 HG11 VAL 142 63.337 58.905 47.789 1.00 0.00 H

ATOM 2352 HG12 VAL 142 61.755 59.564 47.319 1.00 0.00 H

ATOM 2353 HG13 VAL 142 62.549 60.195 48.774 1.00 0.00 H

ATOM 2354 CG2 VAL 142 60.452 58.537 49.654 1.00 0.00 C

ATOM 2355 HG21 VAL 142 59.740 58.370 48.846 1.00 0.00 H

ATOM 2356 HG22 VAL 142 60.286 57.781 50.422 1.00 0.00 H

ATOM 2357 HG23 VAL 142 60.496 59.582 49.961 1.00 0.00 H

ATOM 2358 C VAL 142 62.748 56.551 47.180 1.00 0.00 C

ATOM 2359 O VAL 142 62.473 56.708 45.988 1.00 0.00 O

ATOM 2360 N VAL 143 63.910 56.054 47.498 1.00 0.00 N

ATOM 2361 H VAL 143 63.934 55.845 48.486 1.00 0.00 H

ATOM 2362 CA VAL 143 64.926 55.475 46.679 1.00 0.00 C

ATOM 2363 HA VAL 143 65.175 56.228 45.932 1.00 0.00 H

ATOM 2364 CB VAL 143 66.236 55.257 47.489 1.00 0.00 C

ATOM 2365 HB VAL 143 66.061 54.614 48.352 1.00 0.00 H

ATOM 2366 CG1 VAL 143 67.181 54.501 46.594 1.00 0.00 C

ATOM 2367 HG11 VAL 143 66.899 53.473 46.363 1.00 0.00 H

ATOM 2368 HG12 VAL 143 67.187 55.056 45.656 1.00 0.00 H

ATOM 2369 HG13 VAL 143 68.183 54.530 47.022 1.00 0.00 H

ATOM 2370 CG2 VAL 143 66.810 56.611 47.921 1.00 0.00 C

ATOM 2371 HG21 VAL 143 66.307 56.903 48.843 1.00 0.00 H

ATOM 2372 HG22 VAL 143 67.867 56.630 48.185 1.00 0.00 H

ATOM 2373 HG23 VAL 143 66.791 57.378 47.147 1.00 0.00 H

ATOM 2374 C VAL 143 64.387 54.215 45.846 1.00 0.00 C

ATOM 2375 O VAL 143 64.418 54.294 44.611 1.00 0.00 O

ATOM 2376 N VAL 144 63.737 53.237 46.488 1.00 0.00 N

ATOM 2377 H VAL 144 63.649 53.330 47.490 1.00 0.00 H

ATOM 2378 CA VAL 144 63.011 52.208 45.700 1.00 0.00 C

ATOM 2379 HA VAL 144 63.723 51.676 45.068 1.00 0.00 H

ATOM 2380 CB VAL 144 62.389 51.076 46.620 1.00 0.00 C

ATOM 2381 HB VAL 144 61.836 51.544 47.433 1.00 0.00 H

ATOM 2382 CG1 VAL 144 61.348 50.175 45.963 1.00 0.00 C

ATOM 2383 HG11 VAL 144 61.015 49.364 46.611 1.00 0.00 H

ATOM 2384 HG12 VAL 144 60.527 50.843 45.702 1.00 0.00 H

ATOM 2385 HG13 VAL 144 61.760 49.789 45.031 1.00 0.00 H

ATOM 2386 CG2 VAL 144 63.619 50.326 47.089 1.00 0.00 C

ATOM 2387 HG21 VAL 144 64.175 51.063 47.668 1.00 0.00 H

ATOM 2388 HG22 VAL 144 63.192 49.590 47.771 1.00 0.00 H

ATOM 2389 HG23 VAL 144 64.039 49.794 46.236 1.00 0.00 H

ATOM 2390 C VAL 144 61.946 52.753 44.779 1.00 0.00 C

ATOM 2391 O VAL 144 61.632 52.187 43.733 1.00 0.00 O

ATOM 2392 N ALA 145 61.218 53.758 45.266 1.00 0.00 N

ATOM 2393 H ALA 145 61.424 54.060 46.208 1.00 0.00 H

ATOM 2394 CA ALA 145 60.105 54.397 44.596 1.00 0.00 C

ATOM 2395 HA ALA 145 59.462 53.633 44.159 1.00 0.00 H

ATOM 2396 CB ALA 145 59.227 55.250 45.402 1.00 0.00 C

ATOM 2397 HB1 ALA 145 59.825 56.072 45.796 1.00 0.00 H

ATOM 2398 HB2 ALA 145 58.362 55.494 44.786 1.00 0.00 H

ATOM 2399 HB3 ALA 145 58.933 54.692 46.291 1.00 0.00 H

ATOM 2400 C ALA 145 60.522 55.126 43.306 1.00 0.00 C

ATOM 2401 O ALA 145 59.901 54.796 42.295 1.00 0.00 O

ATOM 2402 N ILE 146 61.627 55.893 43.329 1.00 0.00 N

ATOM 2403 H ILE 146 62.166 56.083 44.162 1.00 0.00 H

ATOM 2404 CA ILE 146 62.053 56.597 42.059 1.00 0.00 C

ATOM 2405 HA ILE 146 61.145 56.756 41.476 1.00 0.00 H

ATOM 2406 CB ILE 146 62.582 57.982 42.485 1.00 0.00 C

ATOM 2407 HB ILE 146 63.385 57.864 43.213 1.00 0.00 H

ATOM 2408 CG2 ILE 146 63.135 58.797 41.341 1.00 0.00 C

ATOM 2409 HG21 ILE 146 64.110 58.384 41.084 1.00 0.00 H

ATOM 2410 HG22 ILE 146 62.537 58.853 40.431 1.00 0.00 H

ATOM 2411 HG23 ILE 146 63.325 59.838 41.600 1.00 0.00 H

ATOM 2412 CG1 ILE 146 61.376 58.799 43.130 1.00 0.00 C

ATOM 2413 HG12 ILE 146 60.577 59.045 42.431 1.00 0.00 H

ATOM 2414 HG13 ILE 146 60.779 58.244 43.854 1.00 0.00 H

ATOM 2415 CD1 ILE 146 61.833 60.055 43.884 1.00 0.00 C

ATOM 2416 HD11 ILE 146 62.367 59.843 44.810 1.00 0.00 H

ATOM 2417 HD12 ILE 146 62.532 60.529 43.195 1.00 0.00 H

ATOM 2418 HD13 ILE 146 60.966 60.701 44.022 1.00 0.00 H

ATOM 2419 C ILE 146 62.873 55.720 41.215 1.00 0.00 C

ATOM 2420 O ILE 146 62.734 55.826 39.989 1.00 0.00 O

ATOM 2421 N SER 147 63.756 54.875 41.726 1.00 0.00 N

ATOM 2422 H SER 147 63.899 54.803 42.723 1.00 0.00 H

ATOM 2423 CA SER 147 64.645 53.992 40.929 1.00 0.00 C

ATOM 2424 HA SER 147 65.391 54.701 40.570 1.00 0.00 H

ATOM 2425 CB SER 147 65.362 52.990 41.801 1.00 0.00 C

ATOM 2426 HB2 SER 147 64.663 52.315 42.295 1.00 0.00 H

ATOM 2427 HB3 SER 147 66.035 52.389 41.191 1.00 0.00 H

ATOM 2428 OG SER 147 66.146 53.615 42.783 1.00 0.00 O

ATOM 2429 HG SER 147 65.593 53.964 43.486 1.00 0.00 H

ATOM 2430 C SER 147 64.048 53.401 39.641 1.00 0.00 C

ATOM 2431 O SER 147 64.737 53.437 38.590 1.00 0.00 O

ATOM 2432 N PRO 148 62.842 52.805 39.647 1.00 0.00 N

ATOM 2433 CD PRO 148 62.202 52.301 40.858 1.00 0.00 C

ATOM 2434 HD2 PRO 148 61.839 53.130 41.464 1.00 0.00 H

ATOM 2435 HD3 PRO 148 62.853 51.599 41.380 1.00 0.00 H

ATOM 2436 CG PRO 148 60.921 51.577 40.391 1.00 0.00 C

ATOM 2437 HG2 PRO 148 60.193 52.376 40.254 1.00 0.00 H

ATOM 2438 HG3 PRO 148 60.642 50.851 41.155 1.00 0.00 H

ATOM 2439 CB PRO 148 61.322 50.996 39.039 1.00 0.00 C

ATOM 2440 HB2 PRO 148 60.431 50.868 38.423 1.00 0.00 H

ATOM 2441 HB3 PRO 148 61.710 49.983 39.146 1.00 0.00 H

ATOM 2442 CA PRO 148 62.288 52.002 38.540 1.00 0.00 C

ATOM 2443 HA PRO 148 63.178 51.520 38.136 1.00 0.00 H

ATOM 2444 C PRO 148 61.877 52.801 37.277 1.00 0.00 C

ATOM 2445 O PRO 148 61.714 52.186 36.223 1.00 0.00 O

ATOM 2446 N ILE 149 61.918 54.109 37.326 1.00 0.00 N

ATOM 2447 H ILE 149 62.068 54.552 38.221 1.00 0.00 H

ATOM 2448 CA ILE 149 61.844 54.931 36.132 1.00 0.00 C

ATOM 2449 HA ILE 149 61.810 54.272 35.264 1.00 0.00 H

ATOM 2450 CB ILE 149 60.533 55.836 36.245 1.00 0.00 C

ATOM 2451 HB ILE 149 59.635 55.270 36.494 1.00 0.00 H

ATOM 2452 CG2 ILE 149 60.515 56.801 37.452 1.00 0.00 C

ATOM 2453 HG21 ILE 149 60.495 56.281 38.410 1.00 0.00 H

ATOM 2454 HG22 ILE 149 61.467 57.331 37.411 1.00 0.00 H

ATOM 2455 HG23 ILE 149 59.674 57.467 37.260 1.00 0.00 H

ATOM 2456 CG1 ILE 149 60.234 56.581 34.893 1.00 0.00 C

ATOM 2457 HG12 ILE 149 59.276 57.096 34.958 1.00 0.00 H

ATOM 2458 HG13 ILE 149 61.034 57.275 34.632 1.00 0.00 H

ATOM 2459 CD1 ILE 149 60.082 55.585 33.741 1.00 0.00 C

ATOM 2460 HD11 ILE 149 59.612 56.087 32.895 1.00 0.00 H

ATOM 2461 HD12 ILE 149 61.079 55.261 33.443 1.00 0.00 H

ATOM 2462 HD13 ILE 149 59.441 54.741 33.996 1.00 0.00 H

ATOM 2463 C ILE 149 63.113 55.797 35.911 1.00 0.00 C

ATOM 2464 O ILE 149 63.395 56.182 34.786 1.00 0.00 O

ATOM 2465 N LEU 150 63.943 56.141 36.937 1.00 0.00 N

ATOM 2466 H LEU 150 63.767 55.821 37.878 1.00 0.00 H

ATOM 2467 CA LEU 150 65.038 57.095 36.760 1.00 0.00 C

ATOM 2468 HA LEU 150 64.520 57.939 36.305 1.00 0.00 H

ATOM 2469 CB LEU 150 65.609 57.377 38.063 1.00 0.00 C

ATOM 2470 HB2 LEU 150 64.928 57.325 38.912 1.00 0.00 H

ATOM 2471 HB3 LEU 150 66.282 56.569 38.351 1.00 0.00 H

ATOM 2472 CG LEU 150 66.375 58.740 38.221 1.00 0.00 C

ATOM 2473 HG LEU 150 67.071 58.715 37.383 1.00 0.00 H

ATOM 2474 CD1 LEU 150 65.400 59.847 38.335 1.00 0.00 C

ATOM 2475 HD11 LEU 150 64.510 59.653 37.736 1.00 0.00 H

ATOM 2476 HD12 LEU 150 65.060 60.045 39.351 1.00 0.00 H

ATOM 2477 HD13 LEU 150 65.978 60.747 38.123 1.00 0.00 H

ATOM 2478 CD2 LEU 150 67.126 58.677 39.509 1.00 0.00 C

ATOM 2479 HD21 LEU 150 67.920 57.930 39.481 1.00 0.00 H

ATOM 2480 HD22 LEU 150 67.694 59.607 39.523 1.00 0.00 H

ATOM 2481 HD23 LEU 150 66.409 58.515 40.314 1.00 0.00 H

ATOM 2482 C LEU 150 66.148 56.645 35.776 1.00 0.00 C

ATOM 2483 O LEU 150 66.656 57.486 35.066 1.00 0.00 O

ATOM 2484 N PHE 151 66.366 55.338 35.662 1.00 0.00 N

ATOM 2485 H PHE 151 66.018 54.707 36.370 1.00 0.00 H

ATOM 2486 CA PHE 151 67.357 54.822 34.638 1.00 0.00 C

ATOM 2487 HA PHE 151 68.272 55.297 34.991 1.00 0.00 H

ATOM 2488 CB PHE 151 67.454 53.301 34.488 1.00 0.00 C

ATOM 2489 HB2 PHE 151 68.205 52.901 33.806 1.00 0.00 H

ATOM 2490 HB3 PHE 151 67.859 52.912 35.422 1.00 0.00 H

ATOM 2491 CG PHE 151 66.264 52.525 34.092 1.00 0.00 C

ATOM 2492 CD1 PHE 151 65.956 52.357 32.655 1.00 0.00 C

ATOM 2493 HD1 PHE 151 66.564 52.719 31.840 1.00 0.00 H

ATOM 2494 CE1 PHE 151 64.893 51.435 32.450 1.00 0.00 C

ATOM 2495 HE1 PHE 151 64.589 51.241 31.432 1.00 0.00 H

ATOM 2496 CZ PHE 151 64.181 50.722 33.467 1.00 0.00 C

ATOM 2497 HZ PHE 151 63.473 49.969 33.155 1.00 0.00 H

ATOM 2498 CE2 PHE 151 64.375 51.175 34.781 1.00 0.00 C

ATOM 2499 HE2 PHE 151 63.921 50.760 35.668 1.00 0.00 H

ATOM 2500 CD2 PHE 151 65.460 51.980 35.081 1.00 0.00 C

ATOM 2501 HD2 PHE 151 65.743 52.232 36.092 1.00 0.00 H

ATOM 2502 C PHE 151 67.055 55.386 33.235 1.00 0.00 C

ATOM 2503 O PHE 151 67.953 55.689 32.418 1.00 0.00 O

ATOM 2504 N TYR 152 65.748 55.385 32.811 1.00 0.00 N

ATOM 2505 H TYR 152 65.016 55.366 33.507 1.00 0.00 H

ATOM 2506 CA TYR 152 65.354 55.870 31.530 1.00 0.00 C

ATOM 2507 HA TYR 152 65.941 55.428 30.726 1.00 0.00 H

ATOM 2508 CB TYR 152 63.962 55.366 31.184 1.00 0.00 C

ATOM 2509 HB2 TYR 152 63.705 54.448 31.713 1.00 0.00 H

ATOM 2510 HB3 TYR 152 63.198 55.999 31.634 1.00 0.00 H

ATOM 2511 CG TYR 152 63.880 55.330 29.717 1.00 0.00 C

ATOM 2512 CD1 TYR 152 64.339 54.179 29.059 1.00 0.00 C

ATOM 2513 HD1 TYR 152 64.882 53.436 29.624 1.00 0.00 H

ATOM 2514 CE1 TYR 152 64.169 54.084 27.669 1.00 0.00 C

ATOM 2515 HE1 TYR 152 64.334 53.173 27.113 1.00 0.00 H

ATOM 2516 CZ TYR 152 63.588 55.186 26.934 1.00 0.00 C

ATOM 2517 OH TYR 152 63.404 55.152 25.569 1.00 0.00 O

ATOM 2518 HH TYR 152 63.055 55.956 25.178 1.00 0.00 H

ATOM 2519 CE2 TYR 152 63.221 56.347 27.573 1.00 0.00 C

ATOM 2520 HE2 TYR 152 62.603 57.078 27.072 1.00 0.00 H

ATOM 2521 CD2 TYR 152 63.278 56.394 29.012 1.00 0.00 C

ATOM 2522 HD2 TYR 152 62.888 57.244 29.552 1.00 0.00 H

ATOM 2523 C TYR 152 65.501 57.386 31.504 1.00 0.00 C

ATOM 2524 O TYR 152 66.335 57.976 30.813 1.00 0.00 O

ATOM 2525 N SER 153 64.699 58.047 32.297 1.00 0.00 N

ATOM 2526 H SER 153 64.071 57.520 32.888 1.00 0.00 H

ATOM 2527 CA SER 153 64.192 59.399 31.961 1.00 0.00 C

ATOM 2528 HA SER 153 63.993 59.354 30.890 1.00 0.00 H

ATOM 2529 CB SER 153 62.870 59.681 32.658 1.00 0.00 C

ATOM 2530 HB2 SER 153 62.865 59.398 33.710 1.00 0.00 H

ATOM 2531 HB3 SER 153 62.542 60.715 32.553 1.00 0.00 H

ATOM 2532 OG SER 153 61.865 58.900 32.064 1.00 0.00 O

ATOM 2533 HG SER 153 61.766 59.314 31.203 1.00 0.00 H

ATOM 2534 C SER 153 65.158 60.617 32.176 1.00 0.00 C

ATOM 2535 O SER 153 65.741 60.827 33.213 1.00 0.00 O

ATOM 2536 N GLY 154 64.986 61.654 31.388 1.00 0.00 N

ATOM 2537 H GLY 154 64.335 61.400 30.659 1.00 0.00 H

ATOM 2538 CA GLY 154 65.321 63.070 31.556 1.00 0.00 C

ATOM 2539 HA2 GLY 154 64.458 63.727 31.454 1.00 0.00 H

ATOM 2540 HA3 GLY 154 65.531 63.221 32.615 1.00 0.00 H

ATOM 2541 C GLY 154 66.424 63.527 30.594 1.00 0.00 C

ATOM 2542 O GLY 154 66.916 64.649 30.830 1.00 0.00 O

ATOM 2543 N THR 155 66.733 62.779 29.530 1.00 0.00 N

ATOM 2544 H THR 155 66.262 61.910 29.323 1.00 0.00 H

ATOM 2545 CA THR 155 67.883 63.125 28.644 1.00 0.00 C

ATOM 2546 HA THR 155 68.087 64.189 28.764 1.00 0.00 H

ATOM 2547 CB THR 155 69.199 62.358 29.004 1.00 0.00 C

ATOM 2548 HB THR 155 69.855 62.477 28.142 1.00 0.00 H

ATOM 2549 CG2 THR 155 70.011 62.914 30.131 1.00 0.00 C

ATOM 2550 HG21 THR 155 69.955 62.308 31.036 1.00 0.00 H

ATOM 2551 HG22 THR 155 71.072 62.903 29.884 1.00 0.00 H

ATOM 2552 HG23 THR 155 69.647 63.890 30.452 1.00 0.00 H

ATOM 2553 OG1 THR 155 68.868 60.994 29.243 1.00 0.00 O

ATOM 2554 HG1 THR 155 69.732 60.622 29.437 1.00 0.00 H

ATOM 2555 C THR 155 67.548 62.801 27.193 1.00 0.00 C

ATOM 2556 O THR 155 66.622 61.953 26.960 1.00 0.00 O

ATOM 2557 N GLY 156 68.254 63.438 26.287 1.00 0.00 N

ATOM 2558 H GLY 156 68.937 64.129 26.561 1.00 0.00 H

ATOM 2559 CA GLY 156 68.185 63.058 24.865 1.00 0.00 C

ATOM 2560 HA2 GLY 156 68.394 62.003 24.689 1.00 0.00 H

ATOM 2561 HA3 GLY 156 67.187 63.292 24.493 1.00 0.00 H

ATOM 2562 C GLY 156 69.153 63.591 23.882 1.00 0.00 C

ATOM 2563 O GLY 156 70.020 64.359 24.276 1.00 0.00 O

ATOM 2564 N VAL 157 69.378 62.837 22.763 1.00 0.00 N

ATOM 2565 H VAL 157 68.650 62.168 22.562 1.00 0.00 H

ATOM 2566 CA VAL 157 70.482 63.174 21.833 1.00 0.00 C

ATOM 2567 HA VAL 157 71.335 63.551 22.398 1.00 0.00 H

ATOM 2568 CB VAL 157 70.883 61.868 21.040 1.00 0.00 C

ATOM 2569 HB VAL 157 69.932 61.572 20.598 1.00 0.00 H

ATOM 2570 CG1 VAL 157 71.929 62.059 19.947 1.00 0.00 C

ATOM 2571 HG11 VAL 157 72.894 62.379 20.338 1.00 0.00 H

ATOM 2572 HG12 VAL 157 71.995 61.147 19.353 1.00 0.00 H

ATOM 2573 HG13 VAL 157 71.677 62.966 19.399 1.00 0.00 H

ATOM 2574 CG2 VAL 157 71.445 60.829 22.065 1.00 0.00 C

ATOM 2575 HG21 VAL 157 71.476 59.825 21.642 1.00 0.00 H

ATOM 2576 HG22 VAL 157 72.417 61.237 22.341 1.00 0.00 H

ATOM 2577 HG23 VAL 157 70.770 60.650 22.901 1.00 0.00 H

ATOM 2578 C VAL 157 70.121 64.450 20.958 1.00 0.00 C

ATOM 2579 O VAL 157 69.055 64.366 20.356 1.00 0.00 O

ATOM 2580 N ARG 158 71.033 65.392 20.823 1.00 0.00 N

ATOM 2581 H ARG 158 71.989 65.073 20.891 1.00 0.00 H

ATOM 2582 CA ARG 158 70.695 66.664 20.119 1.00 0.00 C

ATOM 2583 HA ARG 158 69.740 66.661 19.596 1.00 0.00 H

ATOM 2584 CB ARG 158 70.498 67.760 21.210 1.00 0.00 C

ATOM 2585 HB2 ARG 158 70.428 68.717 20.693 1.00 0.00 H

ATOM 2586 HB3 ARG 158 69.505 67.629 21.639 1.00 0.00 H

ATOM 2587 CG ARG 158 71.468 67.820 22.342 1.00 0.00 C

ATOM 2588 HG2 ARG 158 71.117 68.512 23.107 1.00 0.00 H

ATOM 2589 HG3 ARG 158 71.510 66.815 22.760 1.00 0.00 H

ATOM 2590 CD ARG 158 72.900 68.158 21.972 1.00 0.00 C

ATOM 2591 HD2 ARG 158 73.306 67.411 21.290 1.00 0.00 H

ATOM 2592 HD3 ARG 158 72.935 69.099 21.422 1.00 0.00 H

ATOM 2593 NE ARG 158 73.829 68.095 23.134 1.00 0.00 N

ATOM 2594 HE ARG 158 74.409 67.298 23.354 1.00 0.00 H

ATOM 2595 CZ ARG 158 74.079 69.128 23.930 1.00 0.00 C

ATOM 2596 NH1 ARG 158 73.319 70.160 24.202 1.00 0.00 N

ATOM 2597 HH11 ARG 158 72.642 70.415 23.497 1.00 0.00 H

ATOM 2598 HH12 ARG 158 73.831 70.969 24.523 1.00 0.00 H

ATOM 2599 NH2 ARG 158 75.125 68.960 24.695 1.00 0.00 N

ATOM 2600 HH21 ARG 158 75.703 68.165 24.462 1.00 0.00 H

ATOM 2601 HH22 ARG 158 75.280 69.733 25.326 1.00 0.00 H

ATOM 2602 C ARG 158 71.675 67.019 19.071 1.00 0.00 C

ATOM 2603 O ARG 158 72.572 66.209 18.792 1.00 0.00 O

ATOM 2604 N LYS 159 71.373 68.134 18.389 1.00 0.00 N

ATOM 2605 H LYS 159 70.434 68.493 18.486 1.00 0.00 H

ATOM 2606 CA LYS 159 72.274 68.958 17.593 1.00 0.00 C

ATOM 2607 HA LYS 159 71.596 69.541 16.969 1.00 0.00 H

ATOM 2608 CB LYS 159 73.131 69.840 18.501 1.00 0.00 C

ATOM 2609 HB2 LYS 159 72.411 70.232 19.220 1.00 0.00 H

ATOM 2610 HB3 LYS 159 73.929 69.286 18.996 1.00 0.00 H

ATOM 2611 CG LYS 159 73.774 70.950 17.649 1.00 0.00 C

ATOM 2612 HG2 LYS 159 74.341 70.448 16.865 1.00 0.00 H

ATOM 2613 HG3 LYS 159 72.950 71.516 17.214 1.00 0.00 H

ATOM 2614 CD LYS 159 74.493 71.958 18.570 1.00 0.00 C

ATOM 2615 HD2 LYS 159 74.004 71.861 19.539 1.00 0.00 H

ATOM 2616 HD3 LYS 159 75.488 71.519 18.637 1.00 0.00 H

ATOM 2617 CE LYS 159 74.742 73.363 18.060 1.00 0.00 C

ATOM 2618 HE2 LYS 159 75.675 73.693 18.517 1.00 0.00 H

ATOM 2619 HE3 LYS 159 74.866 73.241 16.984 1.00 0.00 H

ATOM 2620 NZ LYS 159 73.619 74.254 18.256 1.00 0.00 N

ATOM 2621 HZ1 LYS 159 73.013 73.915 18.990 1.00 0.00 H

ATOM 2622 HZ2 LYS 159 74.071 75.122 18.504 1.00 0.00 H

ATOM 2623 HZ3 LYS 159 73.086 74.306 17.400 1.00 0.00 H

ATOM 2624 C LYS 159 73.167 68.132 16.603 1.00 0.00 C

ATOM 2625 O LYS 159 72.757 68.132 15.430 1.00 0.00 O

ATOM 2626 N ASN 160 74.301 67.515 16.963 1.00 0.00 N

ATOM 2627 H ASN 160 74.608 67.565 17.924 1.00 0.00 H

ATOM 2628 CA ASN 160 75.029 66.726 16.003 1.00 0.00 C

ATOM 2629 HA ASN 160 74.444 66.595 15.093 1.00 0.00 H

ATOM 2630 CB ASN 160 76.361 67.392 15.614 1.00 0.00 C

ATOM 2631 HB2 ASN 160 76.243 68.463 15.448 1.00 0.00 H

ATOM 2632 HB3 ASN 160 77.160 67.254 16.343 1.00 0.00 H

ATOM 2633 CG ASN 160 76.888 66.791 14.308 1.00 0.00 C

ATOM 2634 OD1 ASN 160 76.321 66.529 13.256 1.00 0.00 O

ATOM 2635 ND2 ASN 160 78.164 66.568 14.371 1.00 0.00 N

ATOM 2636 HD21 ASN 160 78.599 66.241 13.519 1.00 0.00 H

ATOM 2637 HD22 ASN 160 78.686 66.688 15.227 1.00 0.00 H

ATOM 2638 C ASN 160 75.345 65.267 16.504 1.00 0.00 C

ATOM 2639 O ASN 160 75.176 64.335 15.680 1.00 0.00 O

ATOM 2640 N LYS 161 75.839 65.081 17.736 1.00 0.00 N

ATOM 2641 H LYS 161 76.132 65.952 18.155 1.00 0.00 H

ATOM 2642 CA LYS 161 76.367 63.734 18.216 1.00 0.00 C

ATOM 2643 HA LYS 161 75.936 62.929 17.622 1.00 0.00 H

ATOM 2644 CB LYS 161 77.930 63.621 18.105 1.00 0.00 C

ATOM 2645 HB2 LYS 161 78.369 64.524 18.529 1.00 0.00 H

ATOM 2646 HB3 LYS 161 78.369 62.749 18.590 1.00 0.00 H

ATOM 2647 CG LYS 161 78.339 63.549 16.675 1.00 0.00 C

ATOM 2648 HG2 LYS 161 77.595 63.090 16.024 1.00 0.00 H

ATOM 2649 HG3 LYS 161 78.340 64.571 16.297 1.00 0.00 H

ATOM 2650 CD LYS 161 79.714 62.895 16.462 1.00 0.00 C

ATOM 2651 HD2 LYS 161 80.412 63.473 17.068 1.00 0.00 H

ATOM 2652 HD3 LYS 161 79.667 61.842 16.737 1.00 0.00 H

ATOM 2653 CE LYS 161 80.075 63.008 14.936 1.00 0.00 C

ATOM 2654 HE2 LYS 161 80.109 64.039 14.582 1.00 0.00 H

ATOM 2655 HE3 LYS 161 81.081 62.666 14.696 1.00 0.00 H

ATOM 2656 NZ LYS 161 79.078 62.162 14.119 1.00 0.00 N

ATOM 2657 HZ1 LYS 161 79.145 61.190 14.385 1.00 0.00 H

ATOM 2658 HZ2 LYS 161 78.115 62.346 14.361 1.00 0.00 H

ATOM 2659 HZ3 LYS 161 79.232 62.121 13.121 1.00 0.00 H

ATOM 2660 C LYS 161 75.927 63.501 19.704 1.00 0.00 C

ATOM 2661 O LYS 161 75.527 62.424 20.098 1.00 0.00 O

ATOM 2662 N THR 162 76.025 64.562 20.551 1.00 0.00 N

ATOM 2663 H THR 162 76.410 65.368 20.078 1.00 0.00 H

ATOM 2664 CA THR 162 76.032 64.416 22.034 1.00 0.00 C

ATOM 2665 HA THR 162 76.430 63.464 22.387 1.00 0.00 H

ATOM 2666 CB THR 162 76.878 65.476 22.685 1.00 0.00 C

ATOM 2667 HB THR 162 76.957 65.185 23.733 1.00 0.00 H

ATOM 2668 CG2 THR 162 78.375 65.489 22.264 1.00 0.00 C

ATOM 2669 HG21 THR 162 78.784 66.146 23.032 1.00 0.00 H

ATOM 2670 HG22 THR 162 78.796 64.486 22.196 1.00 0.00 H

ATOM 2671 HG23 THR 162 78.483 65.935 21.275 1.00 0.00 H

ATOM 2672 OG1 THR 162 76.311 66.733 22.720 1.00 0.00 O

ATOM 2673 HG1 THR 162 76.952 67.438 22.836 1.00 0.00 H

ATOM 2674 C THR 162 74.601 64.445 22.621 1.00 0.00 C

ATOM 2675 O THR 162 73.637 64.632 21.866 1.00 0.00 O

ATOM 2676 N ILE 163 74.527 64.154 23.915 1.00 0.00 N

ATOM 2677 H ILE 163 75.420 63.931 24.332 1.00 0.00 H

ATOM 2678 CA ILE 163 73.259 64.093 24.727 1.00 0.00 C

ATOM 2679 HA ILE 163 72.412 63.958 24.053 1.00 0.00 H

ATOM 2680 CB ILE 163 73.453 62.819 25.609 1.00 0.00 C

ATOM 2681 HB ILE 163 73.699 62.006 24.926 1.00 0.00 H

ATOM 2682 CG2 ILE 163 74.469 62.929 26.728 1.00 0.00 C

ATOM 2683 HG21 ILE 163 74.695 61.994 27.241 1.00 0.00 H

ATOM 2684 HG22 ILE 163 75.383 63.462 26.466 1.00 0.00 H

ATOM 2685 HG23 ILE 163 74.032 63.565 27.497 1.00 0.00 H

ATOM 2686 CG1 ILE 163 72.029 62.368 26.110 1.00 0.00 C

ATOM 2687 HG12 ILE 163 71.605 63.034 26.861 1.00 0.00 H

ATOM 2688 HG13 ILE 163 71.305 62.308 25.297 1.00 0.00 H

ATOM 2689 CD1 ILE 163 72.101 61.020 26.747 1.00 0.00 C

ATOM 2690 HD11 ILE 163 71.077 60.713 26.959 1.00 0.00 H

ATOM 2691 HD12 ILE 163 72.585 60.308 26.079 1.00 0.00 H

ATOM 2692 HD13 ILE 163 72.636 61.102 27.693 1.00 0.00 H

ATOM 2693 C ILE 163 73.028 65.500 25.380 1.00 0.00 C

ATOM 2694 O ILE 163 73.985 66.280 25.635 1.00 0.00 O

ATOM 2695 N THR 164 71.782 65.821 25.761 1.00 0.00 N

ATOM 2696 H THR 164 71.059 65.286 25.302 1.00 0.00 H

ATOM 2697 CA THR 164 71.362 66.852 26.746 1.00 0.00 C

ATOM 2698 HA THR 164 72.326 67.174 27.140 1.00 0.00 H

ATOM 2699 CB THR 164 70.866 68.157 26.049 1.00 0.00 C

ATOM 2700 HB THR 164 71.623 68.340 25.287 1.00 0.00 H

ATOM 2701 CG2 THR 164 69.467 68.145 25.475 1.00 0.00 C

ATOM 2702 HG21 THR 164 68.726 68.369 26.242 1.00 0.00 H

ATOM 2703 HG22 THR 164 69.268 68.834 24.654 1.00 0.00 H

ATOM 2704 HG23 THR 164 69.240 67.120 25.183 1.00 0.00 H

ATOM 2705 OG1 THR 164 70.843 69.196 27.031 1.00 0.00 O

ATOM 2706 HG1 THR 164 70.643 70.057 26.658 1.00 0.00 H

ATOM 2707 C THR 164 70.327 66.448 27.772 1.00 0.00 C

ATOM 2708 O THR 164 69.669 65.425 27.607 1.00 0.00 O

ATOM 2709 N CYX 165 70.296 67.135 28.880 1.00 0.00 N

ATOM 2710 H CYX 165 70.705 68.050 28.753 1.00 0.00 H

ATOM 2711 CA CYX 165 69.252 67.102 29.930 1.00 0.00 C

ATOM 2712 HA CYX 165 69.023 66.092 30.269 1.00 0.00 H

ATOM 2713 CB CYX 165 69.748 67.710 31.188 1.00 0.00 C

ATOM 2714 HB2 CYX 165 70.666 67.129 31.280 1.00 0.00 H

ATOM 2715 HB3 CYX 165 69.983 68.768 31.077 1.00 0.00 H

ATOM 2716 SG CYX 165 68.954 67.576 32.807 1.00 0.00 S

ATOM 2717 C CYX 165 67.877 67.634 29.591 1.00 0.00 C

ATOM 2718 O CYX 165 67.238 68.369 30.343 1.00 0.00 O

ATOM 2719 N TYR 166 67.357 67.280 28.465 1.00 0.00 N

ATOM 2720 H TYR 166 67.955 66.693 27.900 1.00 0.00 H

ATOM 2721 CA TYR 166 65.945 67.309 28.170 1.00 0.00 C

ATOM 2722 HA TYR 166 65.387 67.654 29.041 1.00 0.00 H

ATOM 2723 CB TYR 166 65.595 68.278 27.022 1.00 0.00 C

ATOM 2724 HB2 TYR 166 65.889 69.287 27.311 1.00 0.00 H

ATOM 2725 HB3 TYR 166 66.354 68.169 26.248 1.00 0.00 H

ATOM 2726 CG TYR 166 64.069 68.456 26.701 1.00 0.00 C

ATOM 2727 CD1 TYR 166 63.266 69.221 27.541 1.00 0.00 C

ATOM 2728 HD1 TYR 166 63.681 69.579 28.471 1.00 0.00 H

ATOM 2729 CE1 TYR 166 61.976 69.521 27.158 1.00 0.00 C

ATOM 2730 HE1 TYR 166 61.266 69.672 27.957 1.00 0.00 H

ATOM 2731 CZ TYR 166 61.420 68.943 26.087 1.00 0.00 C

ATOM 2732 OH TYR 166 60.141 69.024 25.857 1.00 0.00 O

ATOM 2733 HH TYR 166 59.952 68.303 25.253 1.00 0.00 H

ATOM 2734 CE2 TYR 166 62.241 68.056 25.283 1.00 0.00 C

ATOM 2735 HE2 TYR 166 61.823 67.589 24.403 1.00 0.00 H

ATOM 2736 CD2 TYR 166 63.539 67.811 25.545 1.00 0.00 C

ATOM 2737 HD2 TYR 166 64.148 67.159 24.937 1.00 0.00 H

ATOM 2738 C TYR 166 65.428 65.893 27.859 1.00 0.00 C

ATOM 2739 O TYR 166 65.996 65.288 26.936 1.00 0.00 O

ATOM 2740 N ASP 167 64.345 65.368 28.415 1.00 0.00 N

ATOM 2741 H ASP 167 63.757 65.930 29.012 1.00 0.00 H

ATOM 2742 CA ASP 167 63.697 64.148 27.828 1.00 0.00 C

ATOM 2743 HA ASP 167 64.513 63.427 27.772 1.00 0.00 H

ATOM 2744 CB ASP 167 62.611 63.603 28.859 1.00 0.00 C

ATOM 2745 HB2 ASP 167 63.092 63.271 29.779 1.00 0.00 H

ATOM 2746 HB3 ASP 167 61.892 64.389 29.092 1.00 0.00 H

ATOM 2747 CG ASP 167 61.853 62.448 28.231 1.00 0.00 C

ATOM 2748 OD1 ASP 167 62.494 61.334 28.124 1.00 0.00 O

ATOM 2749 OD2 ASP 167 60.654 62.591 27.908 1.00 0.00 O

ATOM 2750 C ASP 167 63.171 64.393 26.402 1.00 0.00 C

ATOM 2751 O ASP 167 62.296 65.230 26.169 1.00 0.00 O

ATOM 2752 N THR 168 63.735 63.600 25.519 1.00 0.00 N

ATOM 2753 H THR 168 64.467 63.001 25.873 1.00 0.00 H

ATOM 2754 CA THR 168 62.950 63.006 24.319 1.00 0.00 C

ATOM 2755 HA THR 168 62.004 62.698 24.764 1.00 0.00 H

ATOM 2756 CB THR 168 62.642 64.053 23.188 1.00 0.00 C

ATOM 2757 HB THR 168 63.477 64.708 22.937 1.00 0.00 H

ATOM 2758 CG2 THR 168 62.200 63.475 21.886 1.00 0.00 C

ATOM 2759 HG21 THR 168 63.020 62.810 21.614 1.00 0.00 H

ATOM 2760 HG22 THR 168 61.215 63.019 21.987 1.00 0.00 H

ATOM 2761 HG23 THR 168 62.202 64.254 21.125 1.00 0.00 H

ATOM 2762 OG1 THR 168 61.608 64.935 23.542 1.00 0.00 O

ATOM 2763 HG1 THR 168 61.718 65.160 24.469 1.00 0.00 H

ATOM 2764 C THR 168 63.637 61.738 23.843 1.00 0.00 C

ATOM 2765 O THR 168 64.754 61.913 23.410 1.00 0.00 O

ATOM 2766 N THR 169 62.918 60.640 23.692 1.00 0.00 N

ATOM 2767 H THR 169 61.924 60.661 23.872 1.00 0.00 H

ATOM 2768 CA THR 169 63.293 59.359 22.975 1.00 0.00 C

ATOM 2769 HA THR 169 64.225 59.513 22.429 1.00 0.00 H

ATOM 2770 CB THR 169 63.707 58.335 24.021 1.00 0.00 C

ATOM 2771 HB THR 169 63.992 57.513 23.364 1.00 0.00 H

ATOM 2772 CG2 THR 169 64.930 58.746 24.893 1.00 0.00 C

ATOM 2773 HG21 THR 169 65.403 59.640 24.488 1.00 0.00 H

ATOM 2774 HG22 THR 169 64.603 59.088 25.875 1.00 0.00 H

ATOM 2775 HG23 THR 169 65.539 57.844 24.824 1.00 0.00 H

ATOM 2776 OG1 THR 169 62.700 57.877 24.825 1.00 0.00 O

ATOM 2777 HG1 THR 169 62.445 58.600 25.403 1.00 0.00 H

ATOM 2778 C THR 169 62.112 58.798 22.127 1.00 0.00 C

ATOM 2779 O THR 169 62.339 58.428 20.980 1.00 0.00 O

ATOM 2780 N SER 170 60.869 58.952 22.582 1.00 0.00 N

ATOM 2781 H SER 170 60.678 59.530 23.388 1.00 0.00 H

ATOM 2782 CA SER 170 59.672 58.365 21.938 1.00 0.00 C

ATOM 2783 HA SER 170 59.521 58.750 20.929 1.00 0.00 H

ATOM 2784 CB SER 170 59.683 56.815 21.817 1.00 0.00 C

ATOM 2785 HB2 SER 170 58.662 56.528 21.567 1.00 0.00 H

ATOM 2786 HB3 SER 170 60.332 56.460 21.016 1.00 0.00 H

ATOM 2787 OG SER 170 60.070 56.285 23.059 1.00 0.00 O

ATOM 2788 HG SER 170 61.029 56.274 23.097 1.00 0.00 H

ATOM 2789 C SER 170 58.426 58.826 22.623 1.00 0.00 C

ATOM 2790 O SER 170 58.463 59.044 23.841 1.00 0.00 O

ATOM 2791 N ASP 171 57.362 58.871 21.882 1.00 0.00 N

ATOM 2792 H ASP 171 57.538 58.727 20.898 1.00 0.00 H

ATOM 2793 CA ASP 171 55.936 58.913 22.271 1.00 0.00 C

ATOM 2794 HA ASP 171 55.679 59.916 22.610 1.00 0.00 H

ATOM 2795 CB ASP 171 55.028 58.789 21.077 1.00 0.00 C

ATOM 2796 HB2 ASP 171 55.475 59.217 20.179 1.00 0.00 H

ATOM 2797 HB3 ASP 171 54.916 57.715 20.928 1.00 0.00 H

ATOM 2798 CG ASP 171 53.656 59.397 21.337 1.00 0.00 C

ATOM 2799 OD1 ASP 171 53.638 60.609 21.558 1.00 0.00 O

ATOM 2800 OD2 ASP 171 52.602 58.666 21.559 1.00 0.00 O

ATOM 2801 C ASP 171 55.680 57.823 23.322 1.00 0.00 C

ATOM 2802 O ASP 171 55.134 58.045 24.378 1.00 0.00 O

ATOM 2803 N GLU 172 56.085 56.613 22.995 1.00 0.00 N

ATOM 2804 H GLU 172 56.628 56.474 22.155 1.00 0.00 H

ATOM 2805 CA GLU 172 55.930 55.380 23.737 1.00 0.00 C

ATOM 2806 HA GLU 172 54.882 55.087 23.793 1.00 0.00 H

ATOM 2807 CB GLU 172 56.825 54.295 23.048 1.00 0.00 C

ATOM 2808 HB2 GLU 172 57.895 54.495 22.980 1.00 0.00 H

ATOM 2809 HB3 GLU 172 56.787 53.491 23.784 1.00 0.00 H

ATOM 2810 CG GLU 172 56.107 53.787 21.860 1.00 0.00 C

ATOM 2811 HG2 GLU 172 56.626 52.923 21.446 1.00 0.00 H

ATOM 2812 HG3 GLU 172 55.164 53.333 22.164 1.00 0.00 H

ATOM 2813 CD GLU 172 55.903 54.751 20.677 1.00 0.00 C

ATOM 2814 OE1 GLU 172 56.850 55.417 20.288 1.00 0.00 O

ATOM 2815 OE2 GLU 172 54.815 54.985 20.131 1.00 0.00 O

ATOM 2816 C GLU 172 56.291 55.460 25.215 1.00 0.00 C

ATOM 2817 O GLU 172 55.566 54.946 26.072 1.00 0.00 O

ATOM 2818 N TYR 173 57.597 55.871 25.477 1.00 0.00 N

ATOM 2819 H TYR 173 58.216 55.968 24.684 1.00 0.00 H

ATOM 2820 CA TYR 173 58.154 56.019 26.824 1.00 0.00 C

ATOM 2821 HA TYR 173 57.792 55.237 27.492 1.00 0.00 H

ATOM 2822 CB TYR 173 59.643 55.630 26.746 1.00 0.00 C

ATOM 2823 HB2 TYR 173 59.934 55.271 25.760 1.00 0.00 H

ATOM 2824 HB3 TYR 173 60.089 56.608 26.928 1.00 0.00 H

ATOM 2825 CG TYR 173 60.109 54.774 27.884 1.00 0.00 C

ATOM 2826 CD1 TYR 173 60.046 55.274 29.202 1.00 0.00 C

ATOM 2827 HD1 TYR 173 59.770 56.286 29.460 1.00 0.00 H

ATOM 2828 CE1 TYR 173 60.503 54.463 30.262 1.00 0.00 C

ATOM 2829 HE1 TYR 173 60.520 54.670 31.322 1.00 0.00 H

ATOM 2830 CZ TYR 173 60.990 53.142 30.033 1.00 0.00 C

ATOM 2831 OH TYR 173 60.939 52.278 31.059 1.00 0.00 O

ATOM 2832 HH TYR 173 61.339 52.777 31.774 1.00 0.00 H

ATOM 2833 CE2 TYR 173 61.045 52.684 28.722 1.00 0.00 C

ATOM 2834 HE2 TYR 173 61.311 51.665 28.481 1.00 0.00 H

ATOM 2835 CD2 TYR 173 60.779 53.564 27.667 1.00 0.00 C

ATOM 2836 HD2 TYR 173 61.085 53.439 26.639 1.00 0.00 H

ATOM 2837 C TYR 173 57.837 57.380 27.468 1.00 0.00 C

ATOM 2838 O TYR 173 57.558 57.458 28.668 1.00 0.00 O

ATOM 2839 N LEU 174 57.672 58.420 26.684 1.00 0.00 N

ATOM 2840 H LEU 174 57.821 58.349 25.688 1.00 0.00 H

ATOM 2841 CA LEU 174 56.971 59.615 27.220 1.00 0.00 C

ATOM 2842 HA LEU 174 57.598 60.041 28.003 1.00 0.00 H

ATOM 2843 CB LEU 174 57.018 60.684 26.151 1.00 0.00 C

ATOM 2844 HB2 LEU 174 57.930 61.206 26.441 1.00 0.00 H

ATOM 2845 HB3 LEU 174 57.055 60.168 25.192 1.00 0.00 H

ATOM 2846 CG LEU 174 55.849 61.730 26.121 1.00 0.00 C

ATOM 2847 HG LEU 174 54.930 61.173 26.306 1.00 0.00 H

ATOM 2848 CD1 LEU 174 56.038 62.880 27.093 1.00 0.00 C

ATOM 2849 HD11 LEU 174 57.044 63.299 27.088 1.00 0.00 H

ATOM 2850 HD12 LEU 174 55.282 63.660 27.006 1.00 0.00 H

ATOM 2851 HD13 LEU 174 55.842 62.465 28.082 1.00 0.00 H

ATOM 2852 CD2 LEU 174 55.874 62.462 24.761 1.00 0.00 C

ATOM 2853 HD21 LEU 174 55.538 61.708 24.050 1.00 0.00 H

ATOM 2854 HD22 LEU 174 55.073 63.201 24.763 1.00 0.00 H

ATOM 2855 HD23 LEU 174 56.821 62.881 24.420 1.00 0.00 H

ATOM 2856 C LEU 174 55.563 59.440 27.871 1.00 0.00 C

ATOM 2857 O LEU 174 55.425 59.970 28.949 1.00 0.00 O

ATOM 2858 N ARG 175 54.878 58.381 27.428 1.00 0.00 N

ATOM 2859 H ARG 175 55.253 57.984 26.578 1.00 0.00 H

ATOM 2860 CA ARG 175 53.658 57.796 28.033 1.00 0.00 C

ATOM 2861 HA ARG 175 53.002 58.616 28.328 1.00 0.00 H

ATOM 2862 CB ARG 175 52.984 56.837 27.088 1.00 0.00 C

ATOM 2863 HB2 ARG 175 52.808 57.352 26.144 1.00 0.00 H

ATOM 2864 HB3 ARG 175 53.534 55.897 27.034 1.00 0.00 H

ATOM 2865 CG ARG 175 51.646 56.399 27.675 1.00 0.00 C

ATOM 2866 HG2 ARG 175 51.776 55.601 28.405 1.00 0.00 H

ATOM 2867 HG3 ARG 175 51.158 57.275 28.103 1.00 0.00 H

ATOM 2868 CD ARG 175 50.685 55.775 26.576 1.00 0.00 C

ATOM 2869 HD2 ARG 175 51.337 55.041 26.103 1.00 0.00 H

ATOM 2870 HD3 ARG 175 49.939 55.167 27.088 1.00 0.00 H

ATOM 2871 NE ARG 175 49.932 56.760 25.652 1.00 0.00 N

ATOM 2872 HE ARG 175 50.242 56.730 24.692 1.00 0.00 H

ATOM 2873 CZ ARG 175 49.007 57.538 26.058 1.00 0.00 C

ATOM 2874 NH1 ARG 175 48.503 58.473 25.280 1.00 0.00 N

ATOM 2875 HH11 ARG 175 48.592 58.456 24.274 1.00 0.00 H

ATOM 2876 HH12 ARG 175 47.784 59.115 25.583 1.00 0.00 H

ATOM 2877 NH2 ARG 175 48.534 57.517 27.297 1.00 0.00 N

ATOM 2878 HH21 ARG 175 48.630 56.651 27.808 1.00 0.00 H

ATOM 2879 HH22 ARG 175 47.697 58.062 27.444 1.00 0.00 H

ATOM 2880 C ARG 175 53.841 57.244 29.465 1.00 0.00 C

ATOM 2881 O ARG 175 53.194 57.699 30.415 1.00 0.00 O

ATOM 2882 N SER 176 54.683 56.184 29.615 1.00 0.00 N

ATOM 2883 H SER 176 55.198 55.918 28.789 1.00 0.00 H

ATOM 2884 CA SER 176 55.123 55.559 30.879 1.00 0.00 C

ATOM 2885 HA SER 176 54.319 54.984 31.338 1.00 0.00 H

ATOM 2886 CB SER 176 56.360 54.777 30.711 1.00 0.00 C

ATOM 2887 HB2 SER 176 57.209 55.427 30.498 1.00 0.00 H

ATOM 2888 HB3 SER 176 56.484 54.388 31.721 1.00 0.00 H

ATOM 2889 OG SER 176 56.321 53.778 29.673 1.00 0.00 O

ATOM 2890 HG SER 176 55.430 53.713 29.321 1.00 0.00 H

ATOM 2891 C SER 176 55.370 56.527 32.014 1.00 0.00 C

ATOM 2892 O SER 176 54.791 56.293 33.063 1.00 0.00 O

ATOM 2893 N TYR 177 56.334 57.497 31.792 1.00 0.00 N

ATOM 2894 H TYR 177 56.806 57.481 30.898 1.00 0.00 H

ATOM 2895 CA TYR 177 56.738 58.325 32.925 1.00 0.00 C

ATOM 2896 HA TYR 177 56.756 57.744 33.847 1.00 0.00 H

ATOM 2897 CB TYR 177 58.060 58.991 32.687 1.00 0.00 C

ATOM 2898 HB2 TYR 177 58.532 59.338 33.606 1.00 0.00 H

ATOM 2899 HB3 TYR 177 58.805 58.320 32.259 1.00 0.00 H

ATOM 2900 CG TYR 177 58.051 60.314 31.861 1.00 0.00 C

ATOM 2901 CD1 TYR 177 58.343 60.221 30.482 1.00 0.00 C

ATOM 2902 HD1 TYR 177 58.282 59.310 29.905 1.00 0.00 H

ATOM 2903 CE1 TYR 177 58.624 61.416 29.865 1.00 0.00 C

ATOM 2904 HE1 TYR 177 58.835 61.492 28.809 1.00 0.00 H

ATOM 2905 CZ TYR 177 58.727 62.621 30.621 1.00 0.00 C

ATOM 2906 OH TYR 177 59.234 63.709 29.969 1.00 0.00 O

ATOM 2907 HH TYR 177 59.752 63.534 29.180 1.00 0.00 H

ATOM 2908 CE2 TYR 177 58.343 62.677 31.981 1.00 0.00 C

ATOM 2909 HE2 TYR 177 58.398 63.610 32.522 1.00 0.00 H

ATOM 2910 CD2 TYR 177 57.978 61.485 32.580 1.00 0.00 C

ATOM 2911 HD2 TYR 177 57.523 61.555 33.557 1.00 0.00 H

ATOM 2912 C TYR 177 55.660 59.431 33.085 1.00 0.00 C

ATOM 2913 O TYR 177 55.572 59.827 34.253 1.00 0.00 O

ATOM 2914 N PHE 178 54.660 59.755 32.174 1.00 0.00 N

ATOM 2915 H PHE 178 54.800 59.616 31.184 1.00 0.00 H

ATOM 2916 CA PHE 178 53.413 60.596 32.485 1.00 0.00 C

ATOM 2917 HA PHE 178 53.624 61.524 33.015 1.00 0.00 H

ATOM 2918 CB PHE 178 52.824 60.990 31.133 1.00 0.00 C

ATOM 2919 HB2 PHE 178 53.557 61.662 30.686 1.00 0.00 H

ATOM 2920 HB3 PHE 178 52.635 60.105 30.526 1.00 0.00 H

ATOM 2921 CG PHE 178 51.364 61.520 31.280 1.00 0.00 C

ATOM 2922 CD1 PHE 178 50.286 60.844 30.742 1.00 0.00 C

ATOM 2923 HD1 PHE 178 50.560 60.040 30.076 1.00 0.00 H

ATOM 2924 CE1 PHE 178 49.015 61.360 30.908 1.00 0.00 C

ATOM 2925 HE1 PHE 178 48.189 60.800 30.496 1.00 0.00 H

ATOM 2926 CZ PHE 178 48.823 62.506 31.634 1.00 0.00 C

ATOM 2927 HZ PHE 178 47.834 62.906 31.803 1.00 0.00 H

ATOM 2928 CE2 PHE 178 49.901 63.203 32.220 1.00 0.00 C

ATOM 2929 HE2 PHE 178 49.760 64.148 32.724 1.00 0.00 H

ATOM 2930 CD2 PHE 178 51.206 62.780 32.044 1.00 0.00 C

ATOM 2931 HD2 PHE 178 52.031 63.451 32.231 1.00 0.00 H

ATOM 2932 C PHE 178 52.382 59.931 33.376 1.00 0.00 C

ATOM 2933 O PHE 178 52.073 60.417 34.455 1.00 0.00 O

ATOM 2934 N ILE 179 52.246 58.598 33.156 1.00 0.00 N

ATOM 2935 H ILE 179 52.593 58.135 32.329 1.00 0.00 H

ATOM 2936 CA ILE 179 51.492 57.668 34.031 1.00 0.00 C

ATOM 2937 HA ILE 179 50.476 58.040 34.166 1.00 0.00 H

ATOM 2938 CB ILE 179 51.395 56.295 33.331 1.00 0.00 C

ATOM 2939 HB ILE 179 52.331 56.065 32.820 1.00 0.00 H

ATOM 2940 CG2 ILE 179 51.047 55.169 34.354 1.00 0.00 C

ATOM 2941 HG21 ILE 179 51.339 54.175 34.016 1.00 0.00 H

ATOM 2942 HG22 ILE 179 51.512 55.239 35.338 1.00 0.00 H

ATOM 2943 HG23 ILE 179 49.964 55.227 34.462 1.00 0.00 H

ATOM 2944 CG1 ILE 179 50.280 56.432 32.261 1.00 0.00 C

ATOM 2945 HG12 ILE 179 49.402 56.918 32.686 1.00 0.00 H

ATOM 2946 HG13 ILE 179 50.635 57.143 31.515 1.00 0.00 H

ATOM 2947 CD1 ILE 179 49.835 55.163 31.441 1.00 0.00 C

ATOM 2948 HD11 ILE 179 50.657 54.461 31.586 1.00 0.00 H

ATOM 2949 HD12 ILE 179 49.023 54.601 31.903 1.00 0.00 H

ATOM 2950 HD13 ILE 179 49.858 55.381 30.374 1.00 0.00 H

ATOM 2951 C ILE 179 52.112 57.696 35.400 1.00 0.00 C

ATOM 2952 O ILE 179 51.368 57.795 36.425 1.00 0.00 O

ATOM 2953 N TYR 180 53.467 57.731 35.467 1.00 0.00 N

ATOM 2954 H TYR 180 54.070 57.614 34.665 1.00 0.00 H

ATOM 2955 CA TYR 180 54.251 57.627 36.732 1.00 0.00 C

ATOM 2956 HA TYR 180 53.878 56.801 37.338 1.00 0.00 H

ATOM 2957 CB TYR 180 55.738 57.295 36.411 1.00 0.00 C

ATOM 2958 HB2 TYR 180 55.866 56.744 35.479 1.00 0.00 H

ATOM 2959 HB3 TYR 180 56.300 58.202 36.188 1.00 0.00 H

ATOM 2960 CG TYR 180 56.324 56.399 37.490 1.00 0.00 C

ATOM 2961 CD1 TYR 180 56.416 54.973 37.178 1.00 0.00 C

ATOM 2962 HD1 TYR 180 55.766 54.587 36.406 1.00 0.00 H

ATOM 2963 CE1 TYR 180 57.038 54.108 38.036 1.00 0.00 C

ATOM 2964 HE1 TYR 180 57.184 53.086 37.719 1.00 0.00 H

ATOM 2965 CZ TYR 180 57.510 54.639 39.254 1.00 0.00 C

ATOM 2966 OH TYR 180 58.021 53.781 40.156 1.00 0.00 O

ATOM 2967 HH TYR 180 58.582 54.166 40.834 1.00 0.00 H

ATOM 2968 CE2 TYR 180 57.452 56.036 39.483 1.00 0.00 C

ATOM 2969 HE2 TYR 180 58.128 56.371 40.255 1.00 0.00 H

ATOM 2970 CD2 TYR 180 57.058 56.969 38.531 1.00 0.00 C

ATOM 2971 HD2 TYR 180 57.249 58.024 38.657 1.00 0.00 H

ATOM 2972 C TYR 180 54.163 58.967 37.517 1.00 0.00 C

ATOM 2973 O TYR 180 54.082 58.888 38.765 1.00 0.00 O

ATOM 2974 N SER 181 54.222 60.121 36.824 1.00 0.00 N

ATOM 2975 H SER 181 54.447 60.125 35.839 1.00 0.00 H

ATOM 2976 CA SER 181 54.136 61.531 37.275 1.00 0.00 C

ATOM 2977 HA SER 181 54.926 61.812 37.971 1.00 0.00 H

ATOM 2978 CB SER 181 54.347 62.468 36.048 1.00 0.00 C

ATOM 2979 HB2 SER 181 55.340 62.370 35.608 1.00 0.00 H

ATOM 2980 HB3 SER 181 53.606 62.243 35.282 1.00 0.00 H

ATOM 2981 OG SER 181 54.121 63.811 36.369 1.00 0.00 O

ATOM 2982 HG SER 181 54.986 64.224 36.320 1.00 0.00 H

ATOM 2983 C SER 181 52.763 61.835 37.863 1.00 0.00 C

ATOM 2984 O SER 181 52.650 62.562 38.856 1.00 0.00 O

ATOM 2985 N MET 182 51.735 61.062 37.497 1.00 0.00 N

ATOM 2986 H MET 182 51.968 60.565 36.650 1.00 0.00 H

ATOM 2987 CA MET 182 50.421 61.053 38.152 1.00 0.00 C

ATOM 2988 HA MET 182 50.303 62.064 38.541 1.00 0.00 H

ATOM 2989 CB MET 182 49.431 60.607 37.058 1.00 0.00 C

ATOM 2990 HB2 MET 182 49.807 59.691 36.604 1.00 0.00 H

ATOM 2991 HB3 MET 182 48.513 60.531 37.642 1.00 0.00 H

ATOM 2992 CG MET 182 49.257 61.621 35.904 1.00 0.00 C

ATOM 2993 HG2 MET 182 50.250 61.912 35.561 1.00 0.00 H

ATOM 2994 HG3 MET 182 48.695 61.055 35.162 1.00 0.00 H

ATOM 2995 SD MET 182 48.350 63.082 36.616 1.00 0.00 S

ATOM 2996 CE MET 182 49.751 64.246 36.903 1.00 0.00 C

ATOM 2997 HE1 MET 182 49.325 65.172 37.289 1.00 0.00 H

ATOM 2998 HE2 MET 182 50.567 63.721 37.400 1.00 0.00 H

ATOM 2999 HE3 MET 182 50.062 64.494 35.888 1.00 0.00 H

ATOM 3000 C MET 182 50.391 60.101 39.441 1.00 0.00 C

ATOM 3001 O MET 182 49.731 60.522 40.405 1.00 0.00 O

ATOM 3002 N CYX 183 51.214 59.057 39.494 1.00 0.00 N

ATOM 3003 H CYX 183 51.772 58.845 38.679 1.00 0.00 H

ATOM 3004 CA CYX 183 51.368 58.210 40.725 1.00 0.00 C

ATOM 3005 HA CYX 183 50.375 58.187 41.175 1.00 0.00 H

ATOM 3006 CB CYX 183 51.848 56.819 40.305 1.00 0.00 C

ATOM 3007 HB2 CYX 183 52.720 57.035 39.687 1.00 0.00 H

ATOM 3008 HB3 CYX 183 52.325 56.379 41.181 1.00 0.00 H

ATOM 3009 SG CYX 183 50.865 55.650 39.385 1.00 0.00 S

ATOM 3010 C CYX 183 52.243 58.878 41.799 1.00 0.00 C

ATOM 3011 O CYX 183 51.877 58.823 42.991 1.00 0.00 O

ATOM 3012 N THR 184 53.257 59.716 41.483 1.00 0.00 N

ATOM 3013 H THR 184 53.461 59.714 40.493 1.00 0.00 H

ATOM 3014 CA THR 184 53.925 60.641 42.381 1.00 0.00 C

ATOM 3015 HA THR 184 54.100 60.002 43.247 1.00 0.00 H

ATOM 3016 CB THR 184 55.371 60.994 41.997 1.00 0.00 C

ATOM 3017 HB THR 184 55.621 61.633 42.844 1.00 0.00 H

ATOM 3018 CG2 THR 184 56.173 59.814 41.756 1.00 0.00 C

ATOM 3019 HG21 THR 184 57.248 59.950 41.633 1.00 0.00 H

ATOM 3020 HG22 THR 184 56.299 59.198 42.647 1.00 0.00 H

ATOM 3021 HG23 THR 184 55.860 59.195 40.916 1.00 0.00 H

ATOM 3022 OG1 THR 184 55.365 61.668 40.738 1.00 0.00 O

ATOM 3023 HG1 THR 184 56.236 62.035 40.569 1.00 0.00 H

ATOM 3024 C THR 184 53.172 61.798 42.910 1.00 0.00 C

ATOM 3025 O THR 184 53.175 62.019 44.095 1.00 0.00 O

ATOM 3026 N THR 185 52.355 62.387 41.993 1.00 0.00 N

ATOM 3027 H THR 185 52.550 62.298 41.006 1.00 0.00 H

ATOM 3028 CA THR 185 51.537 63.483 42.479 1.00 0.00 C

ATOM 3029 HA THR 185 52.165 64.215 42.986 1.00 0.00 H

ATOM 3030 CB THR 185 50.890 64.196 41.261 1.00 0.00 C

ATOM 3031 HB THR 185 50.362 63.455 40.660 1.00 0.00 H

ATOM 3032 CG2 THR 185 50.160 65.574 41.599 1.00 0.00 C

ATOM 3033 HG21 THR 185 50.829 66.427 41.705 1.00 0.00 H

ATOM 3034 HG22 THR 185 49.464 65.690 40.768 1.00 0.00 H

ATOM 3035 HG23 THR 185 49.516 65.556 42.477 1.00 0.00 H

ATOM 3036 OG1 THR 185 51.920 64.697 40.385 1.00 0.00 O

ATOM 3037 HG1 THR 185 52.153 64.044 39.722 1.00 0.00 H

ATOM 3038 C THR 185 50.440 63.012 43.436 1.00 0.00 C

ATOM 3039 O THR 185 50.271 63.734 44.406 1.00 0.00 O

ATOM 3040 N VAL 186 49.868 61.859 43.315 1.00 0.00 N

ATOM 3041 H VAL 186 50.272 61.317 42.565 1.00 0.00 H

ATOM 3042 CA VAL 186 49.118 61.250 44.412 1.00 0.00 C

ATOM 3043 HA VAL 186 48.369 61.974 44.731 1.00 0.00 H

ATOM 3044 CB VAL 186 48.320 59.999 43.919 1.00 0.00 C

ATOM 3045 HB VAL 186 48.982 59.232 43.517 1.00 0.00 H

ATOM 3046 CG1 VAL 186 47.547 59.341 45.051 1.00 0.00 C

ATOM 3047 HG11 VAL 186 48.169 58.665 45.639 1.00 0.00 H

ATOM 3048 HG12 VAL 186 47.089 60.057 45.733 1.00 0.00 H

ATOM 3049 HG13 VAL 186 46.725 58.793 44.590 1.00 0.00 H

ATOM 3050 CG2 VAL 186 47.370 60.354 42.764 1.00 0.00 C

ATOM 3051 HG21 VAL 186 47.038 59.440 42.273 1.00 0.00 H

ATOM 3052 HG22 VAL 186 46.452 60.743 43.205 1.00 0.00 H

ATOM 3053 HG23 VAL 186 47.792 61.036 42.026 1.00 0.00 H

ATOM 3054 C VAL 186 49.893 60.891 45.685 1.00 0.00 C

ATOM 3055 O VAL 186 49.870 61.591 46.712 1.00 0.00 O

ATOM 3056 N ALA 187 50.773 59.872 45.644 1.00 0.00 N

ATOM 3057 H ALA 187 50.936 59.407 44.762 1.00 0.00 H

ATOM 3058 CA ALA 187 51.444 59.175 46.794 1.00 0.00 C

ATOM 3059 HA ALA 187 50.640 58.958 47.498 1.00 0.00 H

ATOM 3060 CB ALA 187 52.137 57.857 46.427 1.00 0.00 C

ATOM 3061 HB1 ALA 187 52.981 58.151 45.803 1.00 0.00 H

ATOM 3062 HB2 ALA 187 52.466 57.250 47.269 1.00 0.00 H

ATOM 3063 HB3 ALA 187 51.458 57.265 45.813 1.00 0.00 H

ATOM 3064 C ALA 187 52.422 60.142 47.504 1.00 0.00 C

ATOM 3065 O ALA 187 52.499 60.081 48.725 1.00 0.00 O

ATOM 3066 N MET 188 53.110 60.971 46.809 1.00 0.00 N

ATOM 3067 H MET 188 53.000 60.956 45.806 1.00 0.00 H

ATOM 3068 CA MET 188 54.211 61.754 47.382 1.00 0.00 C

ATOM 3069 HA MET 188 54.348 61.589 48.451 1.00 0.00 H

ATOM 3070 CB MET 188 55.465 61.405 46.649 1.00 0.00 C

ATOM 3071 HB2 MET 188 55.259 61.644 45.606 1.00 0.00 H

ATOM 3072 HB3 MET 188 56.182 62.182 46.915 1.00 0.00 H

ATOM 3073 CG MET 188 55.988 60.002 46.717 1.00 0.00 C

ATOM 3074 HG2 MET 188 56.758 60.010 47.488 1.00 0.00 H

ATOM 3075 HG3 MET 188 55.211 59.300 47.019 1.00 0.00 H

ATOM 3076 SD MET 188 56.810 59.628 45.177 1.00 0.00 S

ATOM 3077 CE MET 188 58.513 59.262 45.835 1.00 0.00 C

ATOM 3078 HE1 MET 188 58.552 58.358 46.444 1.00 0.00 H

ATOM 3079 HE2 MET 188 59.101 59.279 44.917 1.00 0.00 H

ATOM 3080 HE3 MET 188 58.954 59.999 46.506 1.00 0.00 H

ATOM 3081 C MET 188 53.943 63.281 47.377 1.00 0.00 C

ATOM 3082 O MET 188 54.698 63.970 47.956 1.00 0.00 O

ATOM 3083 N PHE 189 52.833 63.744 46.826 1.00 0.00 N

ATOM 3084 H PHE 189 52.089 63.163 46.467 1.00 0.00 H

ATOM 3085 CA PHE 189 52.510 65.168 47.129 1.00 0.00 C

ATOM 3086 HA PHE 189 53.185 65.547 47.896 1.00 0.00 H

ATOM 3087 CB PHE 189 52.813 66.014 45.866 1.00 0.00 C

ATOM 3088 HB2 PHE 189 53.901 65.936 45.849 1.00 0.00 H

ATOM 3089 HB3 PHE 189 52.377 65.533 44.990 1.00 0.00 H

ATOM 3090 CG PHE 189 52.533 67.580 45.859 1.00 0.00 C

ATOM 3091 CD1 PHE 189 52.727 68.211 47.051 1.00 0.00 C

ATOM 3092 HD1 PHE 189 53.047 67.578 47.866 1.00 0.00 H

ATOM 3093 CE1 PHE 189 52.414 69.593 47.184 1.00 0.00 C

ATOM 3094 HE1 PHE 189 52.535 70.157 48.097 1.00 0.00 H

ATOM 3095 CZ PHE 189 51.934 70.230 46.048 1.00 0.00 C

ATOM 3096 HZ PHE 189 51.552 71.232 46.175 1.00 0.00 H

ATOM 3097 CE2 PHE 189 51.741 69.585 44.776 1.00 0.00 C

ATOM 3098 HE2 PHE 189 51.383 70.105 43.901 1.00 0.00 H

ATOM 3099 CD2 PHE 189 52.003 68.213 44.768 1.00 0.00 C

ATOM 3100 HD2 PHE 189 52.094 67.725 43.809 1.00 0.00 H

ATOM 3101 C PHE 189 51.064 65.330 47.642 1.00 0.00 C

ATOM 3102 O PHE 189 50.946 65.990 48.642 1.00 0.00 O

ATOM 3103 N CYX 190 50.029 64.688 47.126 1.00 0.00 N

ATOM 3104 H CYX 190 50.144 64.176 46.263 1.00 0.00 H

ATOM 3105 CA CYX 190 48.680 65.000 47.600 1.00 0.00 C

ATOM 3106 HA CYX 190 48.546 66.059 47.823 1.00 0.00 H

ATOM 3107 CB CYX 190 47.716 64.790 46.468 1.00 0.00 C

ATOM 3108 HB2 CYX 190 48.016 64.000 45.778 1.00 0.00 H

ATOM 3109 HB3 CYX 190 46.734 64.535 46.866 1.00 0.00 H

ATOM 3110 SG CYX 190 47.623 66.210 45.337 1.00 0.00 S

ATOM 3111 C CYX 190 48.234 64.139 48.839 1.00 0.00 C

ATOM 3112 O CYX 190 47.584 64.636 49.774 1.00 0.00 O

ATOM 3113 N VAL 191 48.624 62.830 48.910 1.00 0.00 N

ATOM 3114 H VAL 191 49.207 62.479 48.163 1.00 0.00 H

ATOM 3115 CA VAL 191 48.418 61.966 50.040 1.00 0.00 C

ATOM 3116 HA VAL 191 47.365 61.901 50.313 1.00 0.00 H

ATOM 3117 CB VAL 191 48.837 60.569 49.698 1.00 0.00 C

ATOM 3118 HB VAL 191 49.659 60.564 48.981 1.00 0.00 H

ATOM 3119 CG1 VAL 191 49.168 59.690 50.990 1.00 0.00 C

ATOM 3120 HG11 VAL 191 50.201 59.938 51.236 1.00 0.00 H

ATOM 3121 HG12 VAL 191 48.460 59.920 51.786 1.00 0.00 H

ATOM 3122 HG13 VAL 191 49.049 58.624 50.798 1.00 0.00 H

ATOM 3123 CG2 VAL 191 47.619 59.804 49.001 1.00 0.00 C

ATOM 3124 HG21 VAL 191 47.293 60.286 48.080 1.00 0.00 H

ATOM 3125 HG22 VAL 191 47.853 58.775 48.727 1.00 0.00 H

ATOM 3126 HG23 VAL 191 46.765 59.743 49.676 1.00 0.00 H

ATOM 3127 C VAL 191 49.124 62.378 51.299 1.00 0.00 C

ATOM 3128 O VAL 191 48.423 62.495 52.323 1.00 0.00 O

ATOM 3129 N PRO 192 50.441 62.862 51.300 1.00 0.00 N

ATOM 3130 CD PRO 192 51.521 62.738 50.275 1.00 0.00 C

ATOM 3131 HD2 PRO 192 51.140 63.354 49.461 1.00 0.00 H

ATOM 3132 HD3 PRO 192 51.680 61.700 49.982 1.00 0.00 H

ATOM 3133 CG PRO 192 52.730 63.507 50.852 1.00 0.00 C

ATOM 3134 HG2 PRO 192 52.965 64.451 50.359 1.00 0.00 H

ATOM 3135 HG3 PRO 192 53.522 62.800 50.604 1.00 0.00 H

ATOM 3136 CB PRO 192 52.523 63.688 52.333 1.00 0.00 C

ATOM 3137 HB2 PRO 192 52.885 64.656 52.680 1.00 0.00 H

ATOM 3138 HB3 PRO 192 52.984 62.839 52.838 1.00 0.00 H

ATOM 3139 CA PRO 192 51.005 63.581 52.465 1.00 0.00 C

ATOM 3140 HA PRO 192 50.767 62.968 53.334 1.00 0.00 H

ATOM 3141 C PRO 192 50.321 64.967 52.688 1.00 0.00 C

ATOM 3142 O PRO 192 50.075 65.267 53.894 1.00 0.00 O

ATOM 3143 N LEU 193 49.668 65.618 51.688 1.00 0.00 N

ATOM 3144 H LEU 193 49.798 65.268 50.750 1.00 0.00 H

ATOM 3145 CA LEU 193 48.899 66.853 51.866 1.00 0.00 C

ATOM 3146 HA LEU 193 49.598 67.432 52.469 1.00 0.00 H

ATOM 3147 CB LEU 193 48.681 67.659 50.550 1.00 0.00 C

ATOM 3148 HB2 LEU 193 49.316 67.321 49.731 1.00 0.00 H

ATOM 3149 HB3 LEU 193 47.621 67.841 50.373 1.00 0.00 H

ATOM 3150 CG LEU 193 49.115 69.115 50.635 1.00 0.00 C

ATOM 3151 HG LEU 193 50.157 69.177 50.949 1.00 0.00 H

ATOM 3152 CD1 LEU 193 48.819 69.781 49.290 1.00 0.00 C

ATOM 3153 HD11 LEU 193 49.006 70.852 49.369 1.00 0.00 H

ATOM 3154 HD12 LEU 193 49.415 69.304 48.512 1.00 0.00 H

ATOM 3155 HD13 LEU 193 47.745 69.738 49.108 1.00 0.00 H

ATOM 3156 CD2 LEU 193 48.478 69.956 51.677 1.00 0.00 C

ATOM 3157 HD21 LEU 193 47.402 69.782 51.671 1.00 0.00 H

ATOM 3158 HD22 LEU 193 48.868 69.803 52.683 1.00 0.00 H

ATOM 3159 HD23 LEU 193 48.715 71.014 51.571 1.00 0.00 H

ATOM 3160 C LEU 193 47.605 66.661 52.675 1.00 0.00 C

ATOM 3161 O LEU 193 47.223 67.511 53.475 1.00 0.00 O

ATOM 3162 N VAL 194 46.864 65.549 52.345 1.00 0.00 N

ATOM 3163 H VAL 194 47.167 64.967 51.576 1.00 0.00 H

ATOM 3164 CA VAL 194 45.625 65.154 53.044 1.00 0.00 C

ATOM 3165 HA VAL 194 45.116 66.071 53.341 1.00 0.00 H

ATOM 3166 CB VAL 194 44.584 64.339 52.251 1.00 0.00 C

ATOM 3167 HB VAL 194 43.836 63.867 52.888 1.00 0.00 H

ATOM 3168 CG1 VAL 194 43.848 65.169 51.271 1.00 0.00 C

ATOM 3169 HG11 VAL 194 43.071 64.597 50.765 1.00 0.00 H

ATOM 3170 HG12 VAL 194 43.334 65.944 51.840 1.00 0.00 H

ATOM 3171 HG13 VAL 194 44.495 65.496 50.457 1.00 0.00 H

ATOM 3172 CG2 VAL 194 45.205 63.027 51.615 1.00 0.00 C

ATOM 3173 HG21 VAL 194 44.434 62.265 51.504 1.00 0.00 H

ATOM 3174 HG22 VAL 194 45.536 63.318 50.618 1.00 0.00 H

ATOM 3175 HG23 VAL 194 46.016 62.637 52.230 1.00 0.00 H

ATOM 3176 C VAL 194 45.895 64.494 54.459 1.00 0.00 C

ATOM 3177 O VAL 194 45.084 64.713 55.394 1.00 0.00 O

ATOM 3178 N LEU 195 47.123 64.022 54.681 1.00 0.00 N

ATOM 3179 H LEU 195 47.721 63.837 53.888 1.00 0.00 H

ATOM 3180 CA LEU 195 47.561 63.699 56.034 1.00 0.00 C

ATOM 3181 HA LEU 195 46.728 63.092 56.390 1.00 0.00 H

ATOM 3182 CB LEU 195 48.876 62.892 56.104 1.00 0.00 C

ATOM 3183 HB2 LEU 195 49.502 63.128 55.243 1.00 0.00 H

ATOM 3184 HB3 LEU 195 49.425 63.224 56.985 1.00 0.00 H

ATOM 3185 CG LEU 195 48.586 61.383 56.229 1.00 0.00 C

ATOM 3186 HG LEU 195 47.893 60.931 55.519 1.00 0.00 H

ATOM 3187 CD1 LEU 195 49.915 60.632 56.024 1.00 0.00 C

ATOM 3188 HD11 LEU 195 49.795 59.554 55.918 1.00 0.00 H

ATOM 3189 HD12 LEU 195 50.491 60.987 55.169 1.00 0.00 H

ATOM 3190 HD13 LEU 195 50.538 60.815 56.899 1.00 0.00 H

ATOM 3191 CD2 LEU 195 48.048 61.093 57.596 1.00 0.00 C

ATOM 3192 HD21 LEU 195 47.341 61.854 57.927 1.00 0.00 H

ATOM 3193 HD22 LEU 195 47.545 60.128 57.532 1.00 0.00 H

ATOM 3194 HD23 LEU 195 48.855 61.071 58.328 1.00 0.00 H

ATOM 3195 C LEU 195 47.576 64.939 56.894 1.00 0.00 C

ATOM 3196 O LEU 195 47.018 65.011 58.012 1.00 0.00 O

ATOM 3197 N ILE 196 48.444 65.869 56.509 1.00 0.00 N

ATOM 3198 H ILE 196 49.044 65.677 55.719 1.00 0.00 H

ATOM 3199 CA ILE 196 48.714 67.078 57.392 1.00 0.00 C

ATOM 3200 HA ILE 196 48.740 66.692 58.411 1.00 0.00 H

ATOM 3201 CB ILE 196 50.088 67.740 57.076 1.00 0.00 C

ATOM 3202 HB ILE 196 50.375 68.416 57.882 1.00 0.00 H

ATOM 3203 CG2 ILE 196 51.199 66.666 57.142 1.00 0.00 C

ATOM 3204 HG21 ILE 196 52.217 67.053 57.126 1.00 0.00 H

ATOM 3205 HG22 ILE 196 51.067 66.262 58.146 1.00 0.00 H

ATOM 3206 HG23 ILE 196 51.113 65.769 56.528 1.00 0.00 H

ATOM 3207 CG1 ILE 196 50.294 68.423 55.673 1.00 0.00 C

ATOM 3208 HG12 ILE 196 49.806 67.765 54.954 1.00 0.00 H

ATOM 3209 HG13 ILE 196 49.631 69.288 55.669 1.00 0.00 H

ATOM 3210 CD1 ILE 196 51.625 68.894 55.312 1.00 0.00 C

ATOM 3211 HD11 ILE 196 51.978 68.267 54.493 1.00 0.00 H

ATOM 3212 HD12 ILE 196 51.732 69.935 55.005 1.00 0.00 H

ATOM 3213 HD13 ILE 196 52.220 68.814 56.222 1.00 0.00 H

ATOM 3214 C ILE 196 47.523 67.997 57.463 1.00 0.00 C

ATOM 3215 O ILE 196 47.236 68.651 58.448 1.00 0.00 O

ATOM 3216 N LEU 197 46.634 67.967 56.410 1.00 0.00 N

ATOM 3217 H LEU 197 46.876 67.521 55.536 1.00 0.00 H

ATOM 3218 CA LEU 197 45.330 68.686 56.476 1.00 0.00 C

ATOM 3219 HA LEU 197 45.610 69.736 56.560 1.00 0.00 H

ATOM 3220 CB LEU 197 44.676 68.605 55.115 1.00 0.00 C

ATOM 3221 HB2 LEU 197 45.318 69.180 54.449 1.00 0.00 H

ATOM 3222 HB3 LEU 197 44.556 67.580 54.764 1.00 0.00 H

ATOM 3223 CG LEU 197 43.251 69.194 55.088 1.00 0.00 C

ATOM 3224 HG LEU 197 43.110 69.827 55.964 1.00 0.00 H

ATOM 3225 CD1 LEU 197 43.117 70.171 53.901 1.00 0.00 C

ATOM 3226 HD11 LEU 197 42.149 70.664 53.807 1.00 0.00 H

ATOM 3227 HD12 LEU 197 43.899 70.916 54.045 1.00 0.00 H

ATOM 3228 HD13 LEU 197 43.259 69.584 52.993 1.00 0.00 H

ATOM 3229 CD2 LEU 197 42.100 68.259 55.026 1.00 0.00 C

ATOM 3230 HD21 LEU 197 41.225 68.794 55.395 1.00 0.00 H

ATOM 3231 HD22 LEU 197 41.972 67.966 53.983 1.00 0.00 H

ATOM 3232 HD23 LEU 197 42.340 67.393 55.643 1.00 0.00 H

ATOM 3233 C LEU 197 44.550 68.096 57.672 1.00 0.00 C

ATOM 3234 O LEU 197 43.881 68.800 58.414 1.00 0.00 O

ATOM 3235 N GLY 198 44.653 66.711 57.791 1.00 0.00 N

ATOM 3236 H GLY 198 45.053 66.057 57.134 1.00 0.00 H

ATOM 3237 CA GLY 198 43.990 65.879 58.939 1.00 0.00 C

ATOM 3238 HA2 GLY 198 42.910 65.975 58.833 1.00 0.00 H

ATOM 3239 HA3 GLY 198 44.263 64.837 58.768 1.00 0.00 H

ATOM 3240 C GLY 198 44.439 66.251 60.361 1.00 0.00 C

ATOM 3241 O GLY 198 43.711 66.729 61.257 1.00 0.00 O

ATOM 3242 N CYX 199 45.726 66.416 60.447 1.00 0.00 N

ATOM 3243 H CYX 199 46.149 66.472 59.532 1.00 0.00 H

ATOM 3244 CA CYX 199 46.489 66.750 61.577 1.00 0.00 C

ATOM 3245 HA CYX 199 46.130 66.035 62.318 1.00 0.00 H

ATOM 3246 CB CYX 199 48.005 66.519 61.465 1.00 0.00 C

ATOM 3247 HB2 CYX 199 48.197 66.608 60.396 1.00 0.00 H

ATOM 3248 HB3 CYX 199 48.579 67.256 62.028 1.00 0.00 H

ATOM 3249 SG CYX 199 48.461 64.864 61.872 1.00 0.00 S

ATOM 3250 C CYX 199 46.232 68.217 61.988 1.00 0.00 C

ATOM 3251 O CYX 199 46.055 68.483 63.205 1.00 0.00 O

ATOM 3252 N TYR 200 46.187 69.188 61.014 1.00 0.00 N

ATOM 3253 H TYR 200 46.441 68.894 60.082 1.00 0.00 H

ATOM 3254 CA TYR 200 45.727 70.550 61.207 1.00 0.00 C

ATOM 3255 HA TYR 200 46.202 71.087 62.028 1.00 0.00 H

ATOM 3256 CB TYR 200 46.165 71.358 60.002 1.00 0.00 C

ATOM 3257 HB2 TYR 200 45.491 71.144 59.172 1.00 0.00 H

ATOM 3258 HB3 TYR 200 45.838 72.380 60.194 1.00 0.00 H

ATOM 3259 CG TYR 200 47.622 71.309 59.636 1.00 0.00 C

ATOM 3260 CD1 TYR 200 48.642 70.730 60.462 1.00 0.00 C

ATOM 3261 HD1 TYR 200 48.390 70.330 61.433 1.00 0.00 H

ATOM 3262 CE1 TYR 200 49.964 70.691 60.093 1.00 0.00 C

ATOM 3263 HE1 TYR 200 50.845 70.555 60.703 1.00 0.00 H

ATOM 3264 CZ TYR 200 50.281 71.048 58.807 1.00 0.00 C

ATOM 3265 OH TYR 200 51.625 71.109 58.462 1.00 0.00 O

ATOM 3266 HH TYR 200 52.168 70.820 59.198 1.00 0.00 H

ATOM 3267 CE2 TYR 200 49.374 71.670 57.959 1.00 0.00 C

ATOM 3268 HE2 TYR 200 49.674 71.848 56.937 1.00 0.00 H

ATOM 3269 CD2 TYR 200 48.051 71.786 58.375 1.00 0.00 C

ATOM 3270 HD2 TYR 200 47.277 72.042 57.667 1.00 0.00 H

ATOM 3271 C TYR 200 44.310 70.586 61.570 1.00 0.00 C

ATOM 3272 O TYR 200 43.941 71.066 62.658 1.00 0.00 O

ATOM 3273 N GLY 201 43.419 69.808 60.868 1.00 0.00 N

ATOM 3274 H GLY 201 43.716 69.210 60.110 1.00 0.00 H

ATOM 3275 CA GLY 201 41.998 69.900 61.098 1.00 0.00 C

ATOM 3276 HA2 GLY 201 41.626 70.860 60.741 1.00 0.00 H

ATOM 3277 HA3 GLY 201 41.504 69.222 60.403 1.00 0.00 H

ATOM 3278 C GLY 201 41.603 69.577 62.523 1.00 0.00 C

ATOM 3279 O GLY 201 40.804 70.308 63.127 1.00 0.00 O

ATOM 3280 N LEU 202 42.315 68.664 63.164 1.00 0.00 N

ATOM 3281 H LEU 202 42.964 68.118 62.616 1.00 0.00 H

ATOM 3282 CA LEU 202 42.137 68.098 64.558 1.00 0.00 C

ATOM 3283 HA LEU 202 41.083 68.076 64.832 1.00 0.00 H

ATOM 3284 CB LEU 202 42.664 66.666 64.706 1.00 0.00 C

ATOM 3285 HB2 LEU 202 43.702 66.609 64.379 1.00 0.00 H

ATOM 3286 HB3 LEU 202 42.662 66.409 65.765 1.00 0.00 H

ATOM 3287 CG LEU 202 41.840 65.671 63.998 1.00 0.00 C

ATOM 3288 HG LEU 202 41.526 66.117 63.054 1.00 0.00 H

ATOM 3289 CD1 LEU 202 42.620 64.360 63.721 1.00 0.00 C

ATOM 3290 HD11 LEU 202 41.978 63.772 63.065 1.00 0.00 H

ATOM 3291 HD12 LEU 202 43.626 64.466 63.314 1.00 0.00 H

ATOM 3292 HD13 LEU 202 42.945 63.942 64.674 1.00 0.00 H

ATOM 3293 CD2 LEU 202 40.562 65.279 64.645 1.00 0.00 C

ATOM 3294 HD21 LEU 202 40.638 65.160 65.726 1.00 0.00 H

ATOM 3295 HD22 LEU 202 39.859 66.084 64.428 1.00 0.00 H

ATOM 3296 HD23 LEU 202 40.149 64.357 64.236 1.00 0.00 H

ATOM 3297 C LEU 202 42.916 68.958 65.534 1.00 0.00 C

ATOM 3298 O LEU 202 42.456 69.113 66.707 1.00 0.00 O

ATOM 3299 N ILE 203 44.110 69.555 65.247 1.00 0.00 N

ATOM 3300 H ILE 203 44.560 69.233 64.403 1.00 0.00 H

ATOM 3301 CA ILE 203 44.572 70.697 66.074 1.00 0.00 C

ATOM 3302 HA ILE 203 44.808 70.337 67.076 1.00 0.00 H

ATOM 3303 CB ILE 203 45.984 71.203 65.685 1.00 0.00 C

ATOM 3304 HB ILE 203 45.991 71.404 64.614 1.00 0.00 H

ATOM 3305 CG2 ILE 203 46.339 72.512 66.365 1.00 0.00 C

ATOM 3306 HG21 ILE 203 46.403 72.279 67.428 1.00 0.00 H

ATOM 3307 HG22 ILE 203 47.265 72.942 65.983 1.00 0.00 H

ATOM 3308 HG23 ILE 203 45.514 73.222 66.317 1.00 0.00 H

ATOM 3309 CG1 ILE 203 47.059 70.144 66.032 1.00 0.00 C

ATOM 3310 HG12 ILE 203 47.280 70.040 67.094 1.00 0.00 H

ATOM 3311 HG13 ILE 203 46.741 69.168 65.665 1.00 0.00 H

ATOM 3312 CD1 ILE 203 48.353 70.507 65.272 1.00 0.00 C

ATOM 3313 HD11 ILE 203 48.790 71.398 65.724 1.00 0.00 H

ATOM 3314 HD12 ILE 203 49.089 69.752 65.548 1.00 0.00 H

ATOM 3315 HD13 ILE 203 48.122 70.494 64.207 1.00 0.00 H

ATOM 3316 C ILE 203 43.480 71.784 66.167 1.00 0.00 C

ATOM 3317 O ILE 203 43.203 72.213 67.285 1.00 0.00 O

ATOM 3318 N VAL 204 42.809 72.176 65.076 1.00 0.00 N

ATOM 3319 H VAL 204 43.145 71.871 64.174 1.00 0.00 H

ATOM 3320 CA VAL 204 41.751 73.225 65.176 1.00 0.00 C

ATOM 3321 HA VAL 204 42.229 74.049 65.707 1.00 0.00 H

ATOM 3322 CB VAL 204 41.161 73.695 63.849 1.00 0.00 C

ATOM 3323 HB VAL 204 40.412 72.977 63.516 1.00 0.00 H

ATOM 3324 CG1 VAL 204 40.612 75.065 64.221 1.00 0.00 C

ATOM 3325 HG11 VAL 204 39.869 75.347 63.475 1.00 0.00 H

ATOM 3326 HG12 VAL 204 40.142 75.047 65.205 1.00 0.00 H

ATOM 3327 HG13 VAL 204 41.444 75.767 64.275 1.00 0.00 H

ATOM 3328 CG2 VAL 204 42.217 73.852 62.669 1.00 0.00 C

ATOM 3329 HG21 VAL 204 42.333 72.858 62.238 1.00 0.00 H

ATOM 3330 HG22 VAL 204 41.801 74.561 61.952 1.00 0.00 H

ATOM 3331 HG23 VAL 204 43.190 74.206 63.010 1.00 0.00 H

ATOM 3332 C VAL 204 40.570 72.689 65.984 1.00 0.00 C

ATOM 3333 O VAL 204 40.132 73.387 66.942 1.00 0.00 O

ATOM 3334 N ARG 205 40.044 71.447 65.850 1.00 0.00 N

ATOM 3335 H ARG 205 40.478 70.812 65.196 1.00 0.00 H

ATOM 3336 CA ARG 205 38.897 71.048 66.745 1.00 0.00 C

ATOM 3337 HA ARG 205 38.131 71.820 66.665 1.00 0.00 H

ATOM 3338 CB ARG 205 38.218 69.753 66.310 1.00 0.00 C

ATOM 3339 HB2 ARG 205 38.342 69.709 65.228 1.00 0.00 H

ATOM 3340 HB3 ARG 205 38.788 68.889 66.651 1.00 0.00 H

ATOM 3341 CG ARG 205 36.698 69.527 66.519 1.00 0.00 C

ATOM 3342 HG2 ARG 205 36.147 69.886 65.650 1.00 0.00 H

ATOM 3343 HG3 ARG 205 36.583 68.456 66.682 1.00 0.00 H

ATOM 3344 CD ARG 205 36.139 70.229 67.707 1.00 0.00 C

ATOM 3345 HD2 ARG 205 36.626 69.821 68.592 1.00 0.00 H

ATOM 3346 HD3 ARG 205 36.143 71.316 67.626 1.00 0.00 H

ATOM 3347 NE ARG 205 34.740 69.862 67.909 1.00 0.00 N

ATOM 3348 HE ARG 205 34.067 70.596 67.746 1.00 0.00 H

ATOM 3349 CZ ARG 205 34.141 68.711 68.141 1.00 0.00 C

ATOM 3350 NH1 ARG 205 32.834 68.543 67.978 1.00 0.00 N

ATOM 3351 HH11 ARG 205 32.326 69.281 67.513 1.00 0.00 H

ATOM 3352 HH12 ARG 205 32.317 67.677 67.919 1.00 0.00 H

ATOM 3353 NH2 ARG 205 34.834 67.741 68.573 1.00 0.00 N

ATOM 3354 HH21 ARG 205 34.426 66.911 68.979 1.00 0.00 H

ATOM 3355 HH22 ARG 205 35.754 68.039 68.863 1.00 0.00 H

ATOM 3356 C ARG 205 39.342 70.989 68.191 1.00 0.00 C

ATOM 3357 O ARG 205 38.580 71.381 69.063 1.00 0.00 O

ATOM 3358 N ALA 206 40.616 70.650 68.540 1.00 0.00 N

ATOM 3359 H ALA 206 41.191 70.206 67.837 1.00 0.00 H

ATOM 3360 CA ALA 206 41.201 70.802 69.874 1.00 0.00 C

ATOM 3361 HA ALA 206 40.727 70.064 70.522 1.00 0.00 H

ATOM 3362 CB ALA 206 42.697 70.506 69.943 1.00 0.00 C

ATOM 3363 HB1 ALA 206 43.205 71.271 69.355 1.00 0.00 H

ATOM 3364 HB2 ALA 206 43.051 70.438 70.972 1.00 0.00 H

ATOM 3365 HB3 ALA 206 42.781 69.513 69.503 1.00 0.00 H

ATOM 3366 C ALA 206 41.020 72.212 70.528 1.00 0.00 C

ATOM 3367 O ALA 206 40.539 72.462 71.631 1.00 0.00 O

ATOM 3368 N LEU 207 41.377 73.202 69.695 1.00 0.00 N

ATOM 3369 H LEU 207 41.762 73.042 68.775 1.00 0.00 H

ATOM 3370 CA LEU 207 41.317 74.654 70.041 1.00 0.00 C

ATOM 3371 HA LEU 207 41.795 74.731 71.017 1.00 0.00 H

ATOM 3372 CB LEU 207 42.170 75.495 68.941 1.00 0.00 C

ATOM 3373 HB2 LEU 207 42.099 74.933 68.010 1.00 0.00 H

ATOM 3374 HB3 LEU 207 41.676 76.454 68.785 1.00 0.00 H

ATOM 3375 CG LEU 207 43.671 75.769 69.397 1.00 0.00 C

ATOM 3376 HG LEU 207 43.759 76.465 70.231 1.00 0.00 H

ATOM 3377 CD1 LEU 207 44.513 74.458 69.692 1.00 0.00 C

ATOM 3378 HD11 LEU 207 45.303 74.240 68.973 1.00 0.00 H

ATOM 3379 HD12 LEU 207 44.980 74.579 70.669 1.00 0.00 H

ATOM 3380 HD13 LEU 207 43.854 73.600 69.558 1.00 0.00 H

ATOM 3381 CD2 LEU 207 44.232 76.547 68.227 1.00 0.00 C

ATOM 3382 HD21 LEU 207 45.254 76.907 68.354 1.00 0.00 H

ATOM 3383 HD22 LEU 207 44.214 75.905 67.347 1.00 0.00 H

ATOM 3384 HD23 LEU 207 43.610 77.417 68.020 1.00 0.00 H

ATOM 3385 C LEU 207 39.886 75.175 70.125 1.00 0.00 C

ATOM 3386 O LEU 207 39.579 76.079 70.872 1.00 0.00 O

ATOM 3387 N ILE 208 38.989 74.817 69.211 1.00 0.00 N

ATOM 3388 H ILE 208 39.325 74.250 68.445 1.00 0.00 H

ATOM 3389 CA ILE 208 37.511 75.197 69.220 1.00 0.00 C

ATOM 3390 HA ILE 208 37.468 76.279 69.097 1.00 0.00 H

ATOM 3391 CB ILE 208 36.769 74.681 67.926 1.00 0.00 C

ATOM 3392 HB ILE 208 37.109 73.650 67.824 1.00 0.00 H

ATOM 3393 CG2 ILE 208 35.306 74.568 68.055 1.00 0.00 C

ATOM 3394 HG21 ILE 208 34.935 73.945 68.868 1.00 0.00 H

ATOM 3395 HG22 ILE 208 34.755 75.508 68.097 1.00 0.00 H

ATOM 3396 HG23 ILE 208 34.879 74.094 67.171 1.00 0.00 H

ATOM 3397 CG1 ILE 208 37.353 75.547 66.801 1.00 0.00 C

ATOM 3398 HG12 ILE 208 36.912 76.544 66.838 1.00 0.00 H

ATOM 3399 HG13 ILE 208 38.425 75.715 66.899 1.00 0.00 H

ATOM 3400 CD1 ILE 208 36.955 74.898 65.446 1.00 0.00 C

ATOM 3401 HD11 ILE 208 35.884 74.695 65.483 1.00 0.00 H

ATOM 3402 HD12 ILE 208 37.199 75.531 64.593 1.00 0.00 H

ATOM 3403 HD13 ILE 208 37.391 73.901 65.386 1.00 0.00 H

ATOM 3404 C ILE 208 36.972 74.785 70.594 1.00 0.00 C

ATOM 3405 O ILE 208 36.188 75.461 71.282 1.00 0.00 O

ATOM 3406 N TYR 209 37.236 73.506 71.013 1.00 0.00 N

ATOM 3407 H TYR 209 37.963 72.952 70.582 1.00 0.00 H

ATOM 3408 CA TYR 209 36.356 72.637 71.871 1.00 0.00 C

ATOM 3409 HA TYR 209 35.305 72.921 71.821 1.00 0.00 H

ATOM 3410 CB TYR 209 36.439 71.203 71.368 1.00 0.00 C

ATOM 3411 HB2 TYR 209 36.368 71.122 70.284 1.00 0.00 H

ATOM 3412 HB3 TYR 209 37.453 70.847 71.552 1.00 0.00 H

ATOM 3413 CG TYR 209 35.381 70.208 71.891 1.00 0.00 C

ATOM 3414 CD1 TYR 209 35.843 68.944 72.158 1.00 0.00 C

ATOM 3415 HD1 TYR 209 36.711 68.557 71.644 1.00 0.00 H

ATOM 3416 CE1 TYR 209 34.909 68.007 72.797 1.00 0.00 C

ATOM 3417 HE1 TYR 209 35.306 67.007 72.899 1.00 0.00 H

ATOM 3418 CZ TYR 209 33.656 68.399 73.219 1.00 0.00 C

ATOM 3419 OH TYR 209 32.859 67.430 73.644 1.00 0.00 O

ATOM 3420 HH TYR 209 31.987 67.707 73.933 1.00 0.00 H

ATOM 3421 CE2 TYR 209 33.193 69.710 72.885 1.00 0.00 C

ATOM 3422 HE2 TYR 209 32.210 69.903 73.288 1.00 0.00 H

ATOM 3423 CD2 TYR 209 34.082 70.625 72.235 1.00 0.00 C

ATOM 3424 HD2 TYR 209 33.668 71.554 71.871 1.00 0.00 H

ATOM 3425 C TYR 209 36.839 72.727 73.270 1.00 0.00 C

ATOM 3426 O TYR 209 36.093 72.865 74.237 1.00 0.00 O

ATOM 3427 N LYS 210 38.116 72.461 73.427 1.00 0.00 N

ATOM 3428 H LYS 210 38.703 72.709 72.644 1.00 0.00 H

ATOM 3429 CA LYS 210 38.802 72.107 74.756 1.00 0.00 C

ATOM 3430 HA LYS 210 38.096 72.096 75.586 1.00 0.00 H

ATOM 3431 CB LYS 210 39.338 70.664 74.576 1.00 0.00 C

ATOM 3432 HB2 LYS 210 39.855 70.520 73.627 1.00 0.00 H

ATOM 3433 HB3 LYS 210 40.078 70.446 75.346 1.00 0.00 H

ATOM 3434 CG LYS 210 38.183 69.669 74.742 1.00 0.00 C

ATOM 3435 HG2 LYS 210 37.595 69.835 75.644 1.00 0.00 H

ATOM 3436 HG3 LYS 210 37.440 69.713 73.945 1.00 0.00 H

ATOM 3437 CD LYS 210 38.742 68.233 74.801 1.00 0.00 C

ATOM 3438 HD2 LYS 210 39.274 67.906 73.908 1.00 0.00 H

ATOM 3439 HD3 LYS 210 39.466 68.136 75.609 1.00 0.00 H

ATOM 3440 CE LYS 210 37.583 67.254 75.143 1.00 0.00 C

ATOM 3441 HE2 LYS 210 36.695 67.369 74.520 1.00 0.00 H

ATOM 3442 HE3 LYS 210 37.970 66.241 75.034 1.00 0.00 H

ATOM 3443 NZ LYS 210 37.145 67.506 76.491 1.00 0.00 N

ATOM 3444 HZ1 LYS 210 36.688 68.390 76.658 1.00 0.00 H

ATOM 3445 HZ2 LYS 210 36.418 66.851 76.740 1.00 0.00 H

ATOM 3446 HZ3 LYS 210 37.792 67.447 77.264 1.00 0.00 H

ATOM 3447 C LYS 210 39.880 73.037 75.221 1.00 0.00 C

ATOM 3448 O LYS 210 40.111 73.356 76.392 1.00 0.00 O

ATOM 3449 N ASP 211 40.736 73.489 74.304 1.00 0.00 N

ATOM 3450 H ASP 211 40.431 73.297 73.360 1.00 0.00 H

ATOM 3451 CA ASP 211 42.088 73.973 74.611 1.00 0.00 C

ATOM 3452 HA ASP 211 42.486 73.479 75.497 1.00 0.00 H

ATOM 3453 CB ASP 211 43.129 73.609 73.528 1.00 0.00 C

ATOM 3454 HB2 ASP 211 42.711 72.823 72.898 1.00 0.00 H

ATOM 3455 HB3 ASP 211 43.370 74.419 72.839 1.00 0.00 H

ATOM 3456 CG ASP 211 44.486 73.414 74.122 1.00 0.00 C

ATOM 3457 OD1 ASP 211 45.189 74.438 74.322 1.00 0.00 O

ATOM 3458 OD2 ASP 211 44.723 72.306 74.657 1.00 0.00 O

ATOM 3459 C ASP 211 42.056 75.470 74.978 1.00 0.00 C

ATOM 3460 O ASP 211 42.102 76.308 74.049 1.00 0.00 O

ATOM 3461 N LEU 212 42.132 75.885 76.223 1.00 0.00 N

ATOM 3462 H LEU 212 42.330 75.251 76.984 1.00 0.00 H

ATOM 3463 CA LEU 212 41.851 77.348 76.469 1.00 0.00 C

ATOM 3464 HA LEU 212 41.023 77.734 75.875 1.00 0.00 H

ATOM 3465 CB LEU 212 41.511 77.485 77.973 1.00 0.00 C

ATOM 3466 HB2 LEU 212 40.639 76.854 78.149 1.00 0.00 H

ATOM 3467 HB3 LEU 212 42.212 77.145 78.735 1.00 0.00 H

ATOM 3468 CG LEU 212 40.902 78.848 78.338 1.00 0.00 C

ATOM 3469 HG LEU 212 41.724 79.556 78.443 1.00 0.00 H

ATOM 3470 CD1 LEU 212 39.863 79.502 77.388 1.00 0.00 C

ATOM 3471 HD11 LEU 212 39.364 80.358 77.843 1.00 0.00 H

ATOM 3472 HD12 LEU 212 40.250 79.793 76.412 1.00 0.00 H

ATOM 3473 HD13 LEU 212 39.069 78.774 77.223 1.00 0.00 H

ATOM 3474 CD2 LEU 212 40.236 78.839 79.797 1.00 0.00 C

ATOM 3475 HD21 LEU 212 40.167 79.871 80.139 1.00 0.00 H

ATOM 3476 HD22 LEU 212 39.248 78.380 79.749 1.00 0.00 H

ATOM 3477 HD23 LEU 212 40.985 78.331 80.405 1.00 0.00 H

ATOM 3478 C LEU 212 43.090 78.216 76.195 1.00 0.00 C

ATOM 3479 O LEU 212 43.059 79.424 75.992 1.00 0.00 O

ATOM 3480 N ASP 213 44.215 77.590 76.366 1.00 0.00 N

ATOM 3481 H ASP 213 44.085 76.624 76.633 1.00 0.00 H

ATOM 3482 CA ASP 213 45.482 78.177 76.593 1.00 0.00 C

ATOM 3483 HA ASP 213 45.323 79.220 76.863 1.00 0.00 H

ATOM 3484 CB ASP 213 46.064 77.573 77.832 1.00 0.00 C

ATOM 3485 HB2 ASP 213 45.326 77.305 78.588 1.00 0.00 H

ATOM 3486 HB3 ASP 213 46.720 76.732 77.607 1.00 0.00 H

ATOM 3487 CG ASP 213 47.039 78.574 78.462 1.00 0.00 C

ATOM 3488 OD1 ASP 213 48.254 78.374 78.360 1.00 0.00 O

ATOM 3489 OD2 ASP 213 46.573 79.668 78.903 1.00 0.00 O

ATOM 3490 C ASP 213 46.490 78.115 75.435 1.00 0.00 C

ATOM 3491 O ASP 213 47.076 77.045 75.129 1.00 0.00 O

ATOM 3492 N ASN 214 46.941 79.281 75.027 1.00 0.00 N

ATOM 3493 H ASN 214 46.473 80.015 75.541 1.00 0.00 H

ATOM 3494 CA ASN 214 48.021 79.566 74.108 1.00 0.00 C

ATOM 3495 HA ASN 214 47.948 78.898 73.249 1.00 0.00 H

ATOM 3496 CB ASN 214 47.849 81.010 73.488 1.00 0.00 C

ATOM 3497 HB2 ASN 214 47.907 81.762 74.274 1.00 0.00 H

ATOM 3498 HB3 ASN 214 48.613 81.363 72.795 1.00 0.00 H

ATOM 3499 CG ASN 214 46.603 81.295 72.740 1.00 0.00 C

ATOM 3500 OD1 ASN 214 46.593 81.001 71.552 1.00 0.00 O

ATOM 3501 ND2 ASN 214 45.518 81.683 73.382 1.00 0.00 N

ATOM 3502 HD21 ASN 214 45.571 81.790 74.385 1.00 0.00 H

ATOM 3503 HD22 ASN 214 44.603 81.404 73.057 1.00 0.00 H

ATOM 3504 C ASN 214 49.410 79.437 74.705 1.00 0.00 C

ATOM 3505 O ASN 214 50.201 80.393 74.793 1.00 0.00 O

ATOM 3506 N SER 215 49.766 78.174 75.037 1.00 0.00 N

ATOM 3507 H SER 215 49.013 77.506 74.946 1.00 0.00 H

ATOM 3508 CA SER 215 51.020 77.653 75.591 1.00 0.00 C

ATOM 3509 HA SER 215 51.450 78.458 76.188 1.00 0.00 H

ATOM 3510 CB SER 215 50.849 76.418 76.387 1.00 0.00 C

ATOM 3511 HB2 SER 215 50.663 75.629 75.659 1.00 0.00 H

ATOM 3512 HB3 SER 215 51.779 76.193 76.908 1.00 0.00 H

ATOM 3513 OG SER 215 49.715 76.299 77.232 1.00 0.00 O

ATOM 3514 HG SER 215 49.428 77.091 77.692 1.00 0.00 H

ATOM 3515 C SER 215 51.974 77.253 74.421 1.00 0.00 C

ATOM 3516 O SER 215 51.512 76.712 73.419 1.00 0.00 O

ATOM 3517 N PRO 216 53.357 77.306 74.534 1.00 0.00 N

ATOM 3518 CD PRO 216 54.099 77.647 75.734 1.00 0.00 C

ATOM 3519 HD2 PRO 216 54.051 76.880 76.507 1.00 0.00 H

ATOM 3520 HD3 PRO 216 53.710 78.575 76.154 1.00 0.00 H

ATOM 3521 CG PRO 216 55.518 77.931 75.342 1.00 0.00 C

ATOM 3522 HG2 PRO 216 56.172 77.550 76.126 1.00 0.00 H

ATOM 3523 HG3 PRO 216 55.721 79.001 75.293 1.00 0.00 H

ATOM 3524 CB PRO 216 55.718 77.269 73.977 1.00 0.00 C

ATOM 3525 HB2 PRO 216 56.204 76.305 74.132 1.00 0.00 H

ATOM 3526 HB3 PRO 216 56.383 77.737 73.251 1.00 0.00 H

ATOM 3527 CA PRO 216 54.345 77.317 73.385 1.00 0.00 C

ATOM 3528 HA PRO 216 54.287 78.295 72.908 1.00 0.00 H

ATOM 3529 C PRO 216 54.100 76.423 72.122 1.00 0.00 C

ATOM 3530 O PRO 216 53.797 76.877 71.024 1.00 0.00 O

ATOM 3531 N LEU 217 54.127 75.136 72.369 1.00 0.00 N

ATOM 3532 H LEU 217 54.517 74.786 73.232 1.00 0.00 H

ATOM 3533 CA LEU 217 54.007 74.168 71.254 1.00 0.00 C

ATOM 3534 HA LEU 217 54.748 74.322 70.469 1.00 0.00 H

ATOM 3535 CB LEU 217 54.382 72.852 71.872 1.00 0.00 C

ATOM 3536 HB2 LEU 217 55.428 73.062 72.096 1.00 0.00 H

ATOM 3537 HB3 LEU 217 53.804 72.801 72.794 1.00 0.00 H

ATOM 3538 CG LEU 217 54.289 71.626 70.995 1.00 0.00 C

ATOM 3539 HG LEU 217 54.797 70.809 71.508 1.00 0.00 H

ATOM 3540 CD1 LEU 217 52.819 71.256 70.584 1.00 0.00 C

ATOM 3541 HD11 LEU 217 52.217 71.361 71.486 1.00 0.00 H

ATOM 3542 HD12 LEU 217 52.401 71.756 69.710 1.00 0.00 H

ATOM 3543 HD13 LEU 217 52.849 70.190 70.354 1.00 0.00 H

ATOM 3544 CD2 LEU 217 55.042 71.955 69.681 1.00 0.00 C

ATOM 3545 HD21 LEU 217 56.096 72.160 69.868 1.00 0.00 H

ATOM 3546 HD22 LEU 217 54.931 71.061 69.067 1.00 0.00 H

ATOM 3547 HD23 LEU 217 54.477 72.662 69.073 1.00 0.00 H

ATOM 3548 C LEU 217 52.654 74.230 70.575 1.00 0.00 C

ATOM 3549 O LEU 217 52.610 74.261 69.412 1.00 0.00 O

ATOM 3550 N ARG 218 51.590 74.440 71.371 1.00 0.00 N

ATOM 3551 H ARG 218 51.751 74.505 72.366 1.00 0.00 H

ATOM 3552 CA ARG 218 50.215 74.725 70.958 1.00 0.00 C

ATOM 3553 HA ARG 218 49.905 73.924 70.288 1.00 0.00 H

ATOM 3554 CB ARG 218 49.209 74.574 72.132 1.00 0.00 C

ATOM 3555 HB2 ARG 218 49.371 73.610 72.615 1.00 0.00 H

ATOM 3556 HB3 ARG 218 49.510 75.309 72.879 1.00 0.00 H

ATOM 3557 CG ARG 218 47.771 74.642 71.598 1.00 0.00 C

ATOM 3558 HG2 ARG 218 47.673 74.166 70.623 1.00 0.00 H

ATOM 3559 HG3 ARG 218 47.089 73.932 72.064 1.00 0.00 H

ATOM 3560 CD ARG 218 47.175 76.031 71.541 1.00 0.00 C

ATOM 3561 HD2 ARG 218 47.917 76.777 71.826 1.00 0.00 H

ATOM 3562 HD3 ARG 218 46.891 76.348 70.537 1.00 0.00 H

ATOM 3563 NE ARG 218 46.030 76.314 72.387 1.00 0.00 N

ATOM 3564 HE ARG 218 45.810 75.728 73.180 1.00 0.00 H

ATOM 3565 CZ ARG 218 45.267 77.337 72.231 1.00 0.00 C

ATOM 3566 NH1 ARG 218 44.342 77.544 73.118 1.00 0.00 N

ATOM 3567 HH11 ARG 218 43.679 78.282 72.931 1.00 0.00 H

ATOM 3568 HH12 ARG 218 44.076 76.786 73.731 1.00 0.00 H

ATOM 3569 NH2 ARG 218 45.560 78.250 71.453 1.00 0.00 N

ATOM 3570 HH21 ARG 218 45.061 79.120 71.569 1.00 0.00 H

ATOM 3571 HH22 ARG 218 46.416 78.231 70.917 1.00 0.00 H

ATOM 3572 C ARG 218 50.019 76.013 70.084 1.00 0.00 C

ATOM 3573 O ARG 218 49.287 75.874 69.055 1.00 0.00 O

ATOM 3574 N ARG 219 50.687 77.095 70.497 1.00 0.00 N

ATOM 3575 H ARG 219 51.321 76.933 71.267 1.00 0.00 H

ATOM 3576 CA ARG 219 50.844 78.267 69.619 1.00 0.00 C

ATOM 3577 HA ARG 219 49.905 78.796 69.457 1.00 0.00 H

ATOM 3578 CB ARG 219 51.662 79.355 70.374 1.00 0.00 C

ATOM 3579 HB2 ARG 219 52.424 78.813 70.936 1.00 0.00 H

ATOM 3580 HB3 ARG 219 52.280 79.913 69.670 1.00 0.00 H

ATOM 3581 CG ARG 219 50.886 80.277 71.347 1.00 0.00 C

ATOM 3582 HG2 ARG 219 49.964 80.697 70.943 1.00 0.00 H

ATOM 3583 HG3 ARG 219 50.595 79.706 72.228 1.00 0.00 H

ATOM 3584 CD ARG 219 51.858 81.452 71.678 1.00 0.00 C

ATOM 3585 HD2 ARG 219 52.725 81.528 71.022 1.00 0.00 H

ATOM 3586 HD3 ARG 219 51.300 82.386 71.612 1.00 0.00 H

ATOM 3587 NE ARG 219 52.263 81.439 73.087 1.00 0.00 N

ATOM 3588 HE ARG 219 51.566 81.664 73.782 1.00 0.00 H

ATOM 3589 CZ ARG 219 53.393 81.034 73.603 1.00 0.00 C

ATOM 3590 NH1 ARG 219 53.680 81.249 74.873 1.00 0.00 N

ATOM 3591 HH11 ARG 219 54.603 81.116 75.262 1.00 0.00 H

ATOM 3592 HH12 ARG 219 52.894 81.451 75.473 1.00 0.00 H

ATOM 3593 NH2 ARG 219 54.491 80.860 73.030 1.00 0.00 N

ATOM 3594 HH21 ARG 219 55.353 80.885 73.555 1.00 0.00 H

ATOM 3595 HH22 ARG 219 54.760 81.041 72.073 1.00 0.00 H

ATOM 3596 C ARG 219 51.439 77.882 68.189 1.00 0.00 C

ATOM 3597 O ARG 219 50.936 78.268 67.145 1.00 0.00 O

ATOM 3598 N LYS 220 52.652 77.324 68.234 1.00 0.00 N

ATOM 3599 H LYS 220 53.009 77.186 69.169 1.00 0.00 H

ATOM 3600 CA LYS 220 53.585 77.139 67.079 1.00 0.00 C

ATOM 3601 HA LYS 220 53.862 78.106 66.660 1.00 0.00 H

ATOM 3602 CB LYS 220 54.891 76.537 67.702 1.00 0.00 C

ATOM 3603 HB2 LYS 220 54.613 76.021 68.621 1.00 0.00 H

ATOM 3604 HB3 LYS 220 55.387 75.950 66.929 1.00 0.00 H

ATOM 3605 CG LYS 220 55.807 77.757 68.085 1.00 0.00 C

ATOM 3606 HG2 LYS 220 56.101 78.269 67.169 1.00 0.00 H

ATOM 3607 HG3 LYS 220 55.387 78.495 68.769 1.00 0.00 H

ATOM 3608 CD LYS 220 57.022 77.215 68.865 1.00 0.00 C

ATOM 3609 HD2 LYS 220 56.686 76.961 69.870 1.00 0.00 H

ATOM 3610 HD3 LYS 220 57.378 76.291 68.409 1.00 0.00 H

ATOM 3611 CE LYS 220 58.061 78.327 68.952 1.00 0.00 C

ATOM 3612 HE2 LYS 220 57.523 79.256 69.143 1.00 0.00 H

ATOM 3613 HE3 LYS 220 58.602 78.106 69.872 1.00 0.00 H

ATOM 3614 NZ LYS 220 59.065 78.523 67.880 1.00 0.00 N

ATOM 3615 HZ1 LYS 220 58.600 79.027 67.139 1.00 0.00 H

ATOM 3616 HZ2 LYS 220 59.799 79.042 68.340 1.00 0.00 H

ATOM 3617 HZ3 LYS 220 59.350 77.628 67.510 1.00 0.00 H

ATOM 3618 C LYS 220 53.061 76.114 66.021 1.00 0.00 C

ATOM 3619 O LYS 220 53.107 76.446 64.831 1.00 0.00 O

ATOM 3620 N SER 221 52.235 75.203 66.528 1.00 0.00 N

ATOM 3621 H SER 221 52.379 75.031 67.513 1.00 0.00 H

ATOM 3622 CA SER 221 51.298 74.390 65.749 1.00 0.00 C

ATOM 3623 HA SER 221 51.837 73.639 65.171 1.00 0.00 H

ATOM 3624 CB SER 221 50.336 73.667 66.723 1.00 0.00 C

ATOM 3625 HB2 SER 221 49.676 74.362 67.244 1.00 0.00 H

ATOM 3626 HB3 SER 221 49.697 73.006 66.138 1.00 0.00 H

ATOM 3627 OG SER 221 51.069 72.886 67.679 1.00 0.00 O

ATOM 3628 HG SER 221 51.415 73.483 68.346 1.00 0.00 H

ATOM 3629 C SER 221 50.415 75.086 64.745 1.00 0.00 C

ATOM 3630 O SER 221 50.498 74.660 63.584 1.00 0.00 O

ATOM 3631 N ILE 222 49.560 76.103 65.143 1.00 0.00 N

ATOM 3632 H ILE 222 49.538 76.401 66.108 1.00 0.00 H

ATOM 3633 CA ILE 222 48.566 76.662 64.226 1.00 0.00 C

ATOM 3634 HA ILE 222 48.163 75.850 63.621 1.00 0.00 H

ATOM 3635 CB ILE 222 47.246 77.217 64.880 1.00 0.00 C

ATOM 3636 HB ILE 222 46.957 76.565 65.704 1.00 0.00 H

ATOM 3637 CG2 ILE 222 47.394 78.627 65.550 1.00 0.00 C

ATOM 3638 HG21 ILE 222 47.188 79.412 64.823 1.00 0.00 H

ATOM 3639 HG22 ILE 222 46.728 78.807 66.394 1.00 0.00 H

ATOM 3640 HG23 ILE 222 48.414 78.806 65.888 1.00 0.00 H

ATOM 3641 CG1 ILE 222 46.049 77.219 63.837 1.00 0.00 C

ATOM 3642 HG12 ILE 222 45.287 77.927 64.162 1.00 0.00 H

ATOM 3643 HG13 ILE 222 46.333 77.476 62.817 1.00 0.00 H

ATOM 3644 CD1 ILE 222 45.337 75.796 63.671 1.00 0.00 C

ATOM 3645 HD11 ILE 222 44.949 75.505 64.647 1.00 0.00 H

ATOM 3646 HD12 ILE 222 44.464 75.779 63.018 1.00 0.00 H

ATOM 3647 HD13 ILE 222 45.988 74.999 63.312 1.00 0.00 H

ATOM 3648 C ILE 222 49.326 77.669 63.316 1.00 0.00 C

ATOM 3649 O ILE 222 48.979 77.882 62.133 1.00 0.00 O

ATOM 3650 N TYR 223 50.396 78.369 63.731 1.00 0.00 N

ATOM 3651 H TYR 223 50.648 78.343 64.708 1.00 0.00 H

ATOM 3652 CA TYR 223 51.205 79.159 62.876 1.00 0.00 C

ATOM 3653 HA TYR 223 50.512 79.845 62.389 1.00 0.00 H

ATOM 3654 CB TYR 223 52.174 80.117 63.688 1.00 0.00 C

ATOM 3655 HB2 TYR 223 53.162 79.664 63.771 1.00 0.00 H

ATOM 3656 HB3 TYR 223 52.391 80.933 62.999 1.00 0.00 H

ATOM 3657 CG TYR 223 51.783 80.808 65.020 1.00 0.00 C

ATOM 3658 CD1 TYR 223 52.686 80.921 66.068 1.00 0.00 C

ATOM 3659 HD1 TYR 223 53.671 80.500 65.929 1.00 0.00 H

ATOM 3660 CE1 TYR 223 52.376 81.577 67.221 1.00 0.00 C

ATOM 3661 HE1 TYR 223 53.197 81.790 67.889 1.00 0.00 H

ATOM 3662 CZ TYR 223 51.094 81.917 67.484 1.00 0.00 C

ATOM 3663 OH TYR 223 50.583 82.497 68.622 1.00 0.00 O

ATOM 3664 HH TYR 223 49.626 82.524 68.549 1.00 0.00 H

ATOM 3665 CE2 TYR 223 50.036 81.743 66.501 1.00 0.00 C

ATOM 3666 HE2 TYR 223 49.010 81.950 66.767 1.00 0.00 H

ATOM 3667 CD2 TYR 223 50.433 81.233 65.222 1.00 0.00 C

ATOM 3668 HD2 TYR 223 49.741 80.813 64.507 1.00 0.00 H

ATOM 3669 C TYR 223 52.011 78.547 61.799 1.00 0.00 C

ATOM 3670 O TYR 223 52.113 79.156 60.723 1.00 0.00 O

ATOM 3671 N LEU 224 52.490 77.324 61.984 1.00 0.00 N

ATOM 3672 H LEU 224 52.456 76.959 62.925 1.00 0.00 H

ATOM 3673 CA LEU 224 52.965 76.442 60.822 1.00 0.00 C

ATOM 3674 HA LEU 224 53.958 76.782 60.529 1.00 0.00 H

ATOM 3675 CB LEU 224 53.304 75.125 61.421 1.00 0.00 C

ATOM 3676 HB2 LEU 224 53.991 75.312 62.246 1.00 0.00 H

ATOM 3677 HB3 LEU 224 52.417 74.702 61.892 1.00 0.00 H

ATOM 3678 CG LEU 224 54.034 74.111 60.511 1.00 0.00 C

ATOM 3679 HG LEU 224 53.331 73.591 59.860 1.00 0.00 H

ATOM 3680 CD1 LEU 224 55.266 74.597 59.651 1.00 0.00 C

ATOM 3681 HD11 LEU 224 55.078 75.445 58.992 1.00 0.00 H

ATOM 3682 HD12 LEU 224 56.063 74.960 60.300 1.00 0.00 H

ATOM 3683 HD13 LEU 224 55.812 73.823 59.111 1.00 0.00 H

ATOM 3684 CD2 LEU 224 54.533 72.962 61.369 1.00 0.00 C

ATOM 3685 HD21 LEU 224 53.837 72.750 62.181 1.00 0.00 H

ATOM 3686 HD22 LEU 224 54.522 72.049 60.775 1.00 0.00 H

ATOM 3687 HD23 LEU 224 55.587 73.124 61.593 1.00 0.00 H

ATOM 3688 C LEU 224 52.007 76.332 59.610 1.00 0.00 C

ATOM 3689 O LEU 224 52.370 76.583 58.426 1.00 0.00 O

ATOM 3690 N VAL 225 50.740 76.127 59.893 1.00 0.00 N

ATOM 3691 H VAL 225 50.497 76.012 60.866 1.00 0.00 H

ATOM 3692 CA VAL 225 49.738 75.612 58.927 1.00 0.00 C

ATOM 3693 HA VAL 225 50.192 74.770 58.403 1.00 0.00 H

ATOM 3694 CB VAL 225 48.404 75.136 59.584 1.00 0.00 C

ATOM 3695 HB VAL 225 47.995 74.409 58.883 1.00 0.00 H

ATOM 3696 CG1 VAL 225 48.719 74.544 60.985 1.00 0.00 C

ATOM 3697 HG11 VAL 225 47.842 74.030 61.378 1.00 0.00 H

ATOM 3698 HG12 VAL 225 49.436 73.742 60.810 1.00 0.00 H

ATOM 3699 HG13 VAL 225 48.993 75.354 61.662 1.00 0.00 H

ATOM 3700 CG2 VAL 225 47.313 76.198 59.764 1.00 0.00 C

ATOM 3701 HG21 VAL 225 47.625 77.166 60.157 1.00 0.00 H

ATOM 3702 HG22 VAL 225 46.745 76.322 58.842 1.00 0.00 H

ATOM 3703 HG23 VAL 225 46.603 75.796 60.487 1.00 0.00 H

ATOM 3704 C VAL 225 49.461 76.650 57.909 1.00 0.00 C

ATOM 3705 O VAL 225 49.223 76.387 56.724 1.00 0.00 O

ATOM 3706 N ILE 226 49.415 77.918 58.278 1.00 0.00 N

ATOM 3707 H ILE 226 49.533 78.215 59.236 1.00 0.00 H

ATOM 3708 CA ILE 226 49.104 79.077 57.362 1.00 0.00 C

ATOM 3709 HA ILE 226 48.278 78.825 56.696 1.00 0.00 H

ATOM 3710 CB ILE 226 48.495 80.266 58.159 1.00 0.00 C

ATOM 3711 HB ILE 226 48.487 81.214 57.621 1.00 0.00 H

ATOM 3712 CG2 ILE 226 47.028 79.975 58.461 1.00 0.00 C

ATOM 3713 HG21 ILE 226 46.567 79.418 57.645 1.00 0.00 H

ATOM 3714 HG22 ILE 226 46.980 79.409 59.391 1.00 0.00 H

ATOM 3715 HG23 ILE 226 46.540 80.948 58.529 1.00 0.00 H

ATOM 3716 CG1 ILE 226 49.203 80.644 59.505 1.00 0.00 C

ATOM 3717 HG12 ILE 226 49.248 79.759 60.140 1.00 0.00 H

ATOM 3718 HG13 ILE 226 50.248 80.909 59.341 1.00 0.00 H

ATOM 3719 CD1 ILE 226 48.507 81.649 60.401 1.00 0.00 C

ATOM 3720 HD11 ILE 226 48.271 82.530 59.805 1.00 0.00 H

ATOM 3721 HD12 ILE 226 47.562 81.198 60.706 1.00 0.00 H

ATOM 3722 HD13 ILE 226 49.054 81.790 61.334 1.00 0.00 H

ATOM 3723 C ILE 226 50.402 79.538 56.684 1.00 0.00 C

ATOM 3724 O ILE 226 50.243 80.276 55.686 1.00 0.00 O

ATOM 3725 N ILE 227 51.601 78.969 56.911 1.00 0.00 N

ATOM 3726 H ILE 227 51.539 78.111 57.440 1.00 0.00 H

ATOM 3727 CA ILE 227 52.728 79.199 55.968 1.00 0.00 C

ATOM 3728 HA ILE 227 52.497 80.146 55.481 1.00 0.00 H

ATOM 3729 CB ILE 227 54.044 79.358 56.743 1.00 0.00 C

ATOM 3730 HB ILE 227 54.278 78.400 57.207 1.00 0.00 H

ATOM 3731 CG2 ILE 227 55.167 79.801 55.790 1.00 0.00 C

ATOM 3732 HG21 ILE 227 55.353 78.974 55.106 1.00 0.00 H

ATOM 3733 HG22 ILE 227 54.795 80.564 55.105 1.00 0.00 H

ATOM 3734 HG23 ILE 227 56.018 80.074 56.414 1.00 0.00 H

ATOM 3735 CG1 ILE 227 54.086 80.318 57.945 1.00 0.00 C

ATOM 3736 HG12 ILE 227 54.446 81.278 57.574 1.00 0.00 H

ATOM 3737 HG13 ILE 227 53.067 80.580 58.231 1.00 0.00 H

ATOM 3738 CD1 ILE 227 54.829 79.901 59.195 1.00 0.00 C

ATOM 3739 HD11 ILE 227 54.638 78.937 59.667 1.00 0.00 H

ATOM 3740 HD12 ILE 227 55.897 80.054 59.045 1.00 0.00 H

ATOM 3741 HD13 ILE 227 54.590 80.722 59.871 1.00 0.00 H

ATOM 3742 C ILE 227 52.769 78.037 54.972 1.00 0.00 C

ATOM 3743 O ILE 227 52.650 78.308 53.816 1.00 0.00 O

ATOM 3744 N VAL 228 52.713 76.773 55.407 1.00 0.00 N

ATOM 3745 H VAL 228 52.714 76.629 56.407 1.00 0.00 H

ATOM 3746 CA VAL 228 53.037 75.666 54.419 1.00 0.00 C

ATOM 3747 HA VAL 228 53.869 75.989 53.794 1.00 0.00 H

ATOM 3748 CB VAL 228 53.534 74.295 55.112 1.00 0.00 C

ATOM 3749 HB VAL 228 53.810 73.670 54.263 1.00 0.00 H

ATOM 3750 CG1 VAL 228 54.646 74.513 56.128 1.00 0.00 C

ATOM 3751 HG11 VAL 228 54.343 75.333 56.780 1.00 0.00 H

ATOM 3752 HG12 VAL 228 54.915 73.600 56.659 1.00 0.00 H

ATOM 3753 HG13 VAL 228 55.585 74.906 55.739 1.00 0.00 H

ATOM 3754 CG2 VAL 228 52.345 73.672 55.960 1.00 0.00 C

ATOM 3755 HG21 VAL 228 51.736 73.080 55.276 1.00 0.00 H

ATOM 3756 HG22 VAL 228 52.705 73.068 56.792 1.00 0.00 H

ATOM 3757 HG23 VAL 228 51.766 74.464 56.434 1.00 0.00 H

ATOM 3758 C VAL 228 51.927 75.446 53.506 1.00 0.00 C

ATOM 3759 O VAL 228 52.172 75.362 52.320 1.00 0.00 O

ATOM 3760 N LEU 229 50.619 75.498 53.856 1.00 0.00 N

ATOM 3761 H LEU 229 50.562 75.631 54.855 1.00 0.00 H

ATOM 3762 CA LEU 229 49.497 75.264 52.986 1.00 0.00 C

ATOM 3763 HA LEU 229 49.574 74.264 52.558 1.00 0.00 H

ATOM 3764 CB LEU 229 48.168 75.254 53.776 1.00 0.00 C

ATOM 3765 HB2 LEU 229 48.227 76.229 54.262 1.00 0.00 H

ATOM 3766 HB3 LEU 229 47.260 75.225 53.174 1.00 0.00 H

ATOM 3767 CG LEU 229 48.008 74.162 54.795 1.00 0.00 C

ATOM 3768 HG LEU 229 48.875 74.149 55.457 1.00 0.00 H

ATOM 3769 CD1 LEU 229 46.619 74.388 55.573 1.00 0.00 C

ATOM 3770 HD11 LEU 229 46.678 75.393 55.990 1.00 0.00 H

ATOM 3771 HD12 LEU 229 45.800 74.346 54.855 1.00 0.00 H

ATOM 3772 HD13 LEU 229 46.465 73.598 56.308 1.00 0.00 H

ATOM 3773 CD2 LEU 229 48.025 72.785 54.099 1.00 0.00 C

ATOM 3774 HD21 LEU 229 48.300 71.988 54.789 1.00 0.00 H

ATOM 3775 HD22 LEU 229 47.091 72.579 53.575 1.00 0.00 H

ATOM 3776 HD23 LEU 229 48.746 72.665 53.290 1.00 0.00 H

ATOM 3777 C LEU 229 49.472 76.190 51.814 1.00 0.00 C

ATOM 3778 O LEU 229 49.332 75.705 50.645 1.00 0.00 O

ATOM 3779 N THR 230 49.674 77.456 52.109 1.00 0.00 N

ATOM 3780 H THR 230 49.821 77.718 53.073 1.00 0.00 H

ATOM 3781 CA THR 230 49.881 78.551 51.161 1.00 0.00 C

ATOM 3782 HA THR 230 49.004 78.792 50.561 1.00 0.00 H

ATOM 3783 CB THR 230 50.250 79.858 52.006 1.00 0.00 C

ATOM 3784 HB THR 230 51.282 79.773 52.348 1.00 0.00 H

ATOM 3785 CG2 THR 230 49.959 81.103 51.158 1.00 0.00 C

ATOM 3786 HG21 THR 230 50.071 81.956 51.828 1.00 0.00 H

ATOM 3787 HG22 THR 230 50.580 81.231 50.271 1.00 0.00 H

ATOM 3788 HG23 THR 230 48.920 81.032 50.836 1.00 0.00 H

ATOM 3789 OG1 THR 230 49.411 79.782 53.122 1.00 0.00 O

ATOM 3790 HG1 THR 230 49.896 80.025 53.914 1.00 0.00 H

ATOM 3791 C THR 230 50.985 78.250 50.198 1.00 0.00 C

ATOM 3792 O THR 230 50.907 78.601 49.030 1.00 0.00 O

ATOM 3793 N VAL 231 52.135 77.804 50.617 1.00 0.00 N

ATOM 3794 H VAL 231 52.248 77.471 51.564 1.00 0.00 H

ATOM 3795 CA VAL 231 53.211 77.540 49.741 1.00 0.00 C

ATOM 3796 HA VAL 231 53.387 78.459 49.182 1.00 0.00 H

ATOM 3797 CB VAL 231 54.539 77.334 50.534 1.00 0.00 C

ATOM 3798 HB VAL 231 54.397 76.809 51.479 1.00 0.00 H

ATOM 3799 CG1 VAL 231 55.646 76.645 49.685 1.00 0.00 C

ATOM 3800 HG11 VAL 231 55.513 76.729 48.607 1.00 0.00 H

ATOM 3801 HG12 VAL 231 56.651 76.978 49.943 1.00 0.00 H

ATOM 3802 HG13 VAL 231 55.451 75.588 49.865 1.00 0.00 H

ATOM 3803 CG2 VAL 231 54.931 78.736 50.892 1.00 0.00 C

ATOM 3804 HG21 VAL 231 55.648 78.559 51.694 1.00 0.00 H

ATOM 3805 HG22 VAL 231 55.407 79.247 50.056 1.00 0.00 H

ATOM 3806 HG23 VAL 231 54.051 79.234 51.298 1.00 0.00 H

ATOM 3807 C VAL 231 52.825 76.278 48.774 1.00 0.00 C

ATOM 3808 O VAL 231 53.142 76.302 47.558 1.00 0.00 O

ATOM 3809 N PHE 232 52.148 75.255 49.303 1.00 0.00 N

ATOM 3810 H PHE 232 51.747 75.356 50.225 1.00 0.00 H

ATOM 3811 CA PHE 232 51.641 74.157 48.401 1.00 0.00 C

ATOM 3812 HA PHE 232 52.476 73.888 47.755 1.00 0.00 H

ATOM 3813 CB PHE 232 51.092 73.030 49.162 1.00 0.00 C

ATOM 3814 HB2 PHE 232 50.126 73.258 49.614 1.00 0.00 H

ATOM 3815 HB3 PHE 232 50.846 72.272 48.418 1.00 0.00 H

ATOM 3816 CG PHE 232 51.987 72.462 50.266 1.00 0.00 C

ATOM 3817 CD1 PHE 232 53.383 72.178 50.146 1.00 0.00 C

ATOM 3818 HD1 PHE 232 53.925 72.108 49.215 1.00 0.00 H

ATOM 3819 CE1 PHE 232 54.137 71.761 51.254 1.00 0.00 C

ATOM 3820 HE1 PHE 232 55.202 71.596 51.196 1.00 0.00 H

ATOM 3821 CZ PHE 232 53.511 71.416 52.393 1.00 0.00 C

ATOM 3822 HZ PHE 232 54.011 70.990 53.250 1.00 0.00 H

ATOM 3823 CE2 PHE 232 52.150 71.627 52.494 1.00 0.00 C

ATOM 3824 HE2 PHE 232 51.626 71.268 53.368 1.00 0.00 H

ATOM 3825 CD2 PHE 232 51.385 72.104 51.460 1.00 0.00 C

ATOM 3826 HD2 PHE 232 50.360 72.416 51.598 1.00 0.00 H

ATOM 3827 C PHE 232 50.602 74.710 47.442 1.00 0.00 C

ATOM 3828 O PHE 232 50.605 74.271 46.292 1.00 0.00 O

ATOM 3829 N ALA 233 49.816 75.751 47.811 1.00 0.00 N

ATOM 3830 H ALA 233 49.980 76.210 48.695 1.00 0.00 H

ATOM 3831 CA ALA 233 48.889 76.428 46.917 1.00 0.00 C

ATOM 3832 HA ALA 233 48.434 75.649 46.304 1.00 0.00 H

ATOM 3833 CB ALA 233 47.825 76.905 47.903 1.00 0.00 C

ATOM 3834 HB1 ALA 233 47.028 77.431 47.377 1.00 0.00 H

ATOM 3835 HB2 ALA 233 47.523 76.112 48.587 1.00 0.00 H

ATOM 3836 HB3 ALA 233 48.260 77.705 48.502 1.00 0.00 H

ATOM 3837 C ALA 233 49.402 77.434 45.934 1.00 0.00 C

ATOM 3838 O ALA 233 48.904 77.535 44.837 1.00 0.00 O

ATOM 3839 N VAL 234 50.506 78.039 46.306 1.00 0.00 N

ATOM 3840 H VAL 234 50.689 78.095 47.298 1.00 0.00 H

ATOM 3841 CA VAL 234 51.117 79.118 45.476 1.00 0.00 C

ATOM 3842 HA VAL 234 50.335 79.557 44.858 1.00 0.00 H

ATOM 3843 CB VAL 234 51.385 80.265 46.444 1.00 0.00 C

ATOM 3844 HB VAL 234 50.632 80.371 47.225 1.00 0.00 H

ATOM 3845 CG1 VAL 234 52.796 80.079 47.007 1.00 0.00 C

ATOM 3846 HG11 VAL 234 52.908 79.083 47.435 1.00 0.00 H

ATOM 3847 HG12 VAL 234 53.551 80.216 46.233 1.00 0.00 H

ATOM 3848 HG13 VAL 234 52.991 80.814 47.788 1.00 0.00 H

ATOM 3849 CG2 VAL 234 51.451 81.568 45.664 1.00 0.00 C

ATOM 3850 HG21 VAL 234 51.461 82.308 46.465 1.00 0.00 H

ATOM 3851 HG22 VAL 234 52.373 81.579 45.083 1.00 0.00 H

ATOM 3852 HG23 VAL 234 50.606 81.742 44.997 1.00 0.00 H

ATOM 3853 C VAL 234 52.305 78.638 44.671 1.00 0.00 C

ATOM 3854 O VAL 234 52.654 79.269 43.667 1.00 0.00 O

ATOM 3855 N SER 235 52.993 77.540 45.005 1.00 0.00 N

ATOM 3856 H SER 235 52.698 77.209 45.912 1.00 0.00 H

ATOM 3857 CA SER 235 54.123 77.007 44.294 1.00 0.00 C

ATOM 3858 HA SER 235 54.266 77.620 43.404 1.00 0.00 H

ATOM 3859 CB SER 235 55.317 77.035 45.309 1.00 0.00 C

ATOM 3860 HB2 SER 235 55.158 76.276 46.076 1.00 0.00 H

ATOM 3861 HB3 SER 235 56.112 76.643 44.675 1.00 0.00 H

ATOM 3862 OG SER 235 55.562 78.306 45.709 1.00 0.00 O

ATOM 3863 HG SER 235 56.474 78.527 45.913 1.00 0.00 H

ATOM 3864 C SER 235 53.944 75.602 43.738 1.00 0.00 C

ATOM 3865 O SER 235 54.136 75.349 42.573 1.00 0.00 O

ATOM 3866 N TYR 236 53.492 74.669 44.568 1.00 0.00 N

ATOM 3867 H TYR 236 53.049 74.951 45.432 1.00 0.00 H

ATOM 3868 CA TYR 236 53.758 73.216 44.278 1.00 0.00 C

ATOM 3869 HA TYR 236 54.530 73.194 43.509 1.00 0.00 H

ATOM 3870 CB TYR 236 54.361 72.350 45.451 1.00 0.00 C

ATOM 3871 HB2 TYR 236 53.685 72.552 46.282 1.00 0.00 H

ATOM 3872 HB3 TYR 236 54.414 71.280 45.250 1.00 0.00 H

ATOM 3873 CG TYR 236 55.749 72.780 45.851 1.00 0.00 C

ATOM 3874 CD1 TYR 236 55.935 73.352 47.128 1.00 0.00 C

ATOM 3875 HD1 TYR 236 55.113 73.497 47.814 1.00 0.00 H

ATOM 3876 CE1 TYR 236 57.267 73.599 47.504 1.00 0.00 C

ATOM 3877 HE1 TYR 236 57.502 73.979 48.487 1.00 0.00 H

ATOM 3878 CZ TYR 236 58.312 73.143 46.679 1.00 0.00 C

ATOM 3879 OH TYR 236 59.476 73.723 46.892 1.00 0.00 O

ATOM 3880 HH TYR 236 59.981 73.253 46.224 1.00 0.00 H

ATOM 3881 CE2 TYR 236 58.085 72.431 45.471 1.00 0.00 C

ATOM 3882 HE2 TYR 236 58.910 71.962 44.956 1.00 0.00 H

ATOM 3883 CD2 TYR 236 56.808 72.495 44.992 1.00 0.00 C

ATOM 3884 HD2 TYR 236 56.658 72.086 44.004 1.00 0.00 H

ATOM 3885 C TYR 236 52.602 72.686 43.472 1.00 0.00 C

ATOM 3886 O TYR 236 52.855 72.240 42.364 1.00 0.00 O

ATOM 3887 N ILE 237 51.306 72.863 43.961 1.00 0.00 N

ATOM 3888 H ILE 237 51.179 73.410 44.801 1.00 0.00 H

ATOM 3889 CA ILE 237 50.122 72.565 43.106 1.00 0.00 C

ATOM 3890 HA ILE 237 50.220 71.509 42.853 1.00 0.00 H

ATOM 3891 CB ILE 237 48.795 72.741 43.827 1.00 0.00 C

ATOM 3892 HB ILE 237 48.768 73.677 44.385 1.00 0.00 H

ATOM 3893 CG2 ILE 237 47.695 72.632 42.740 1.00 0.00 C

ATOM 3894 HG21 ILE 237 47.824 71.688 42.212 1.00 0.00 H

ATOM 3895 HG22 ILE 237 46.764 72.669 43.305 1.00 0.00 H

ATOM 3896 HG23 ILE 237 47.664 73.504 42.086 1.00 0.00 H

ATOM 3897 CG1 ILE 237 48.757 71.478 44.784 1.00 0.00 C

ATOM 3898 HG12 ILE 237 48.326 70.641 44.234 1.00 0.00 H

ATOM 3899 HG13 ILE 237 49.752 71.203 45.135 1.00 0.00 H

ATOM 3900 CD1 ILE 237 47.956 71.747 46.037 1.00 0.00 C

ATOM 3901 HD11 ILE 237 46.914 71.994 45.832 1.00 0.00 H

ATOM 3902 HD12 ILE 237 47.980 70.768 46.516 1.00 0.00 H

ATOM 3903 HD13 ILE 237 48.571 72.433 46.620 1.00 0.00 H

ATOM 3904 C ILE 237 50.342 73.354 41.744 1.00 0.00 C

ATOM 3905 O ILE 237 50.226 72.684 40.696 1.00 0.00 O

ATOM 3906 N PRO 238 50.661 74.657 41.747 1.00 0.00 N

ATOM 3907 CD PRO 238 50.420 75.642 42.761 1.00 0.00 C

ATOM 3908 HD2 PRO 238 51.242 75.684 43.475 1.00 0.00 H

ATOM 3909 HD3 PRO 238 49.516 75.404 43.321 1.00 0.00 H

ATOM 3910 CG PRO 238 50.367 77.013 42.060 1.00 0.00 C

ATOM 3911 HG2 PRO 238 50.812 77.727 42.753 1.00 0.00 H

ATOM 3912 HG3 PRO 238 49.302 77.055 41.835 1.00 0.00 H

ATOM 3913 CB PRO 238 51.116 76.727 40.771 1.00 0.00 C

ATOM 3914 HB2 PRO 238 52.156 76.949 41.009 1.00 0.00 H

ATOM 3915 HB3 PRO 238 50.747 77.345 39.952 1.00 0.00 H

ATOM 3916 CA PRO 238 50.849 75.274 40.428 1.00 0.00 C

ATOM 3917 HA PRO 238 49.939 75.281 39.828 1.00 0.00 H

ATOM 3918 C PRO 238 51.845 74.608 39.558 1.00 0.00 C

ATOM 3919 O PRO 238 51.500 74.396 38.371 1.00 0.00 O

ATOM 3920 N PHE 239 53.027 74.300 40.037 1.00 0.00 N

ATOM 3921 H PHE 239 53.201 74.576 40.993 1.00 0.00 H

ATOM 3922 CA PHE 239 53.992 73.539 39.230 1.00 0.00 C

ATOM 3923 HA PHE 239 54.135 74.166 38.349 1.00 0.00 H

ATOM 3924 CB PHE 239 55.299 73.539 40.093 1.00 0.00 C

ATOM 3925 HB2 PHE 239 55.698 74.527 40.322 1.00 0.00 H

ATOM 3926 HB3 PHE 239 54.984 73.078 41.029 1.00 0.00 H

ATOM 3927 CG PHE 239 56.549 72.891 39.469 1.00 0.00 C

ATOM 3928 CD1 PHE 239 57.061 73.185 38.129 1.00 0.00 C

ATOM 3929 HD1 PHE 239 56.647 74.039 37.613 1.00 0.00 H

ATOM 3930 CE1 PHE 239 58.104 72.386 37.612 1.00 0.00 C

ATOM 3931 HE1 PHE 239 58.483 72.712 36.655 1.00 0.00 H

ATOM 3932 CZ PHE 239 58.499 71.210 38.306 1.00 0.00 C

ATOM 3933 HZ PHE 239 59.170 70.491 37.861 1.00 0.00 H

ATOM 3934 CE2 PHE 239 58.083 71.019 39.590 1.00 0.00 C

ATOM 3935 HE2 PHE 239 58.438 70.166 40.150 1.00 0.00 H

ATOM 3936 CD2 PHE 239 57.069 71.805 40.175 1.00 0.00 C

ATOM 3937 HD2 PHE 239 56.784 71.671 41.208 1.00 0.00 H

ATOM 3938 C PHE 239 53.581 72.196 38.692 1.00 0.00 C

ATOM 3939 O PHE 239 53.961 71.880 37.544 1.00 0.00 O

ATOM 3940 N HID 240 52.757 71.501 39.520 1.00 0.00 N

ATOM 3941 H HID 240 52.506 71.922 40.402 1.00 0.00 H

ATOM 3942 CA HID 240 52.249 70.157 39.235 1.00 0.00 C

ATOM 3943 HA HID 240 53.089 69.577 38.854 1.00 0.00 H

ATOM 3944 CB HID 240 51.889 69.335 40.443 1.00 0.00 C

ATOM 3945 HB2 HID 240 51.495 69.936 41.263 1.00 0.00 H

ATOM 3946 HB3 HID 240 51.044 68.693 40.195 1.00 0.00 H

ATOM 3947 CG HID 240 53.033 68.588 40.959 1.00 0.00 C

ATOM 3948 ND1 HID 240 53.232 67.234 40.800 1.00 0.00 N

ATOM 3949 HD1 HID 240 52.657 66.632 40.228 1.00 0.00 H

ATOM 3950 CE1 HID 240 54.293 66.779 41.446 1.00 0.00 C

ATOM 3951 HE1 HID 240 54.525 65.746 41.657 1.00 0.00 H

ATOM 3952 NE2 HID 240 54.828 67.932 42.065 1.00 0.00 N

ATOM 3953 CD2 HID 240 54.042 69.079 41.756 1.00 0.00 C

ATOM 3954 HD2 HID 240 54.069 70.071 42.183 1.00 0.00 H

ATOM 3955 C HID 240 51.236 70.248 38.060 1.00 0.00 C

ATOM 3956 O HID 240 51.479 69.555 37.001 1.00 0.00 O

ATOM 3957 N VAL 241 50.241 71.124 38.229 1.00 0.00 N

ATOM 3958 H VAL 241 50.286 71.651 39.090 1.00 0.00 H

ATOM 3959 CA VAL 241 49.344 71.651 37.270 1.00 0.00 C

ATOM 3960 HA VAL 241 48.612 70.874 37.047 1.00 0.00 H

ATOM 3961 CB VAL 241 48.414 72.773 37.853 1.00 0.00 C

ATOM 3962 HB VAL 241 48.975 73.486 38.457 1.00 0.00 H

ATOM 3963 CG1 VAL 241 47.936 73.655 36.706 1.00 0.00 C

ATOM 3964 HG11 VAL 241 47.593 73.080 35.846 1.00 0.00 H

ATOM 3965 HG12 VAL 241 47.160 74.403 36.868 1.00 0.00 H

ATOM 3966 HG13 VAL 241 48.653 74.388 36.338 1.00 0.00 H

ATOM 3967 CG2 VAL 241 47.187 72.169 38.666 1.00 0.00 C

ATOM 3968 HG21 VAL 241 46.795 72.948 39.320 1.00 0.00 H

ATOM 3969 HG22 VAL 241 46.330 71.785 38.111 1.00 0.00 H

ATOM 3970 HG23 VAL 241 47.567 71.336 39.258 1.00 0.00 H

ATOM 3971 C VAL 241 50.109 72.041 35.979 1.00 0.00 C

ATOM 3972 O VAL 241 49.994 71.332 34.972 1.00 0.00 O

ATOM 3973 N MET 242 50.934 73.083 36.017 1.00 0.00 N

ATOM 3974 H MET 242 51.047 73.615 36.868 1.00 0.00 H

ATOM 3975 CA MET 242 51.608 73.539 34.853 1.00 0.00 C

ATOM 3976 HA MET 242 50.787 73.766 34.172 1.00 0.00 H

ATOM 3977 CB MET 242 52.557 74.749 35.117 1.00 0.00 C

ATOM 3978 HB2 MET 242 52.753 74.822 36.186 1.00 0.00 H

ATOM 3979 HB3 MET 242 53.470 74.579 34.547 1.00 0.00 H

ATOM 3980 CG MET 242 52.045 76.139 34.703 1.00 0.00 C

ATOM 3981 HG2 MET 242 52.910 76.788 34.840 1.00 0.00 H

ATOM 3982 HG3 MET 242 51.996 76.020 33.620 1.00 0.00 H

ATOM 3983 SD MET 242 50.458 76.685 35.494 1.00 0.00 S

ATOM 3984 CE MET 242 49.500 76.663 33.913 1.00 0.00 C

ATOM 3985 HE1 MET 242 49.866 77.486 33.300 1.00 0.00 H

ATOM 3986 HE2 MET 242 49.503 75.685 33.432 1.00 0.00 H

ATOM 3987 HE3 MET 242 48.459 76.761 34.221 1.00 0.00 H

ATOM 3988 C MET 242 52.452 72.494 34.164 1.00 0.00 C

ATOM 3989 O MET 242 52.479 72.463 32.947 1.00 0.00 O

ATOM 3990 N LYS 243 53.119 71.517 34.850 1.00 0.00 N

ATOM 3991 H LYS 243 52.973 71.423 35.845 1.00 0.00 H

ATOM 3992 CA LYS 243 53.936 70.469 34.280 1.00 0.00 C

ATOM 3993 HA LYS 243 54.584 70.837 33.485 1.00 0.00 H

ATOM 3994 CB LYS 243 54.745 69.918 35.476 1.00 0.00 C

ATOM 3995 HB2 LYS 243 55.423 70.692 35.836 1.00 0.00 H

ATOM 3996 HB3 LYS 243 54.048 69.671 36.277 1.00 0.00 H

ATOM 3997 CG LYS 243 55.611 68.633 35.154 1.00 0.00 C

ATOM 3998 HG2 LYS 243 54.978 67.751 35.251 1.00 0.00 H

ATOM 3999 HG3 LYS 243 56.151 68.661 34.207 1.00 0.00 H

ATOM 4000 CD LYS 243 56.715 68.483 36.215 1.00 0.00 C

ATOM 4001 HD2 LYS 243 57.404 67.691 35.919 1.00 0.00 H

ATOM 4002 HD3 LYS 243 57.338 69.370 36.328 1.00 0.00 H

ATOM 4003 CE LYS 243 56.056 67.993 37.558 1.00 0.00 C

ATOM 4004 HE2 LYS 243 55.611 68.842 38.076 1.00 0.00 H

ATOM 4005 HE3 LYS 243 55.214 67.362 37.275 1.00 0.00 H

ATOM 4006 NZ LYS 243 57.053 67.238 38.348 1.00 0.00 N

ATOM 4007 HZ1 LYS 243 57.935 67.690 38.155 1.00 0.00 H

ATOM 4008 HZ2 LYS 243 56.819 67.409 39.315 1.00 0.00 H

ATOM 4009 HZ3 LYS 243 57.054 66.310 37.950 1.00 0.00 H

ATOM 4010 C LYS 243 53.120 69.297 33.679 1.00 0.00 C

ATOM 4011 O LYS 243 53.411 68.893 32.553 1.00 0.00 O

ATOM 4012 N THR 244 51.985 69.034 34.357 1.00 0.00 N

ATOM 4013 H THR 244 51.703 69.424 35.245 1.00 0.00 H

ATOM 4014 CA THR 244 50.979 68.020 33.931 1.00 0.00 C

ATOM 4015 HA THR 244 51.455 67.061 33.727 1.00 0.00 H

ATOM 4016 CB THR 244 49.870 67.825 34.900 1.00 0.00 C

ATOM 4017 HB THR 244 49.359 68.776 35.050 1.00 0.00 H

ATOM 4018 CG2 THR 244 48.957 66.711 34.295 1.00 0.00 C

ATOM 4019 HG21 THR 244 48.254 66.372 35.056 1.00 0.00 H

ATOM 4020 HG22 THR 244 48.342 67.271 33.592 1.00 0.00 H

ATOM 4021 HG23 THR 244 49.536 65.970 33.744 1.00 0.00 H

ATOM 4022 OG1 THR 244 50.306 67.435 36.151 1.00 0.00 O

ATOM 4023 HG1 THR 244 50.687 68.201 36.588 1.00 0.00 H

ATOM 4024 C THR 244 50.340 68.503 32.640 1.00 0.00 C

ATOM 4025 O THR 244 50.460 67.722 31.657 1.00 0.00 O

ATOM 4026 N MET 245 49.925 69.725 32.462 1.00 0.00 N

ATOM 4027 H MET 245 50.107 70.418 33.174 1.00 0.00 H

ATOM 4028 CA MET 245 49.724 70.413 31.128 1.00 0.00 C

ATOM 4029 HA MET 245 48.801 70.014 30.709 1.00 0.00 H

ATOM 4030 CB MET 245 49.503 71.901 31.387 1.00 0.00 C

ATOM 4031 HB2 MET 245 50.407 72.418 31.709 1.00 0.00 H

ATOM 4032 HB3 MET 245 49.231 72.331 30.423 1.00 0.00 H

ATOM 4033 CG MET 245 48.308 72.101 32.279 1.00 0.00 C

ATOM 4034 HG2 MET 245 47.509 71.580 31.753 1.00 0.00 H

ATOM 4035 HG3 MET 245 48.458 71.713 33.286 1.00 0.00 H

ATOM 4036 SD MET 245 47.687 73.838 32.472 1.00 0.00 S

ATOM 4037 CE MET 245 47.167 74.382 30.798 1.00 0.00 C

ATOM 4038 HE1 MET 245 46.704 73.542 30.280 1.00 0.00 H

ATOM 4039 HE2 MET 245 46.393 75.135 30.948 1.00 0.00 H

ATOM 4040 HE3 MET 245 47.993 74.769 30.202 1.00 0.00 H

ATOM 4041 C MET 245 50.835 70.200 30.131 1.00 0.00 C

ATOM 4042 O MET 245 50.573 69.671 29.059 1.00 0.00 O

ATOM 4043 N ASN 246 52.104 70.461 30.453 1.00 0.00 N

ATOM 4044 H ASN 246 52.280 70.894 31.348 1.00 0.00 H

ATOM 4045 CA ASN 246 53.222 70.369 29.503 1.00 0.00 C

ATOM 4046 HA ASN 246 53.065 70.915 28.573 1.00 0.00 H

ATOM 4047 CB ASN 246 54.505 71.095 30.108 1.00 0.00 C

ATOM 4048 HB2 ASN 246 54.303 72.154 30.269 1.00 0.00 H

ATOM 4049 HB3 ASN 246 54.776 70.548 31.011 1.00 0.00 H

ATOM 4050 CG ASN 246 55.714 70.946 29.177 1.00 0.00 C

ATOM 4051 OD1 ASN 246 56.386 69.900 29.228 1.00 0.00 O

ATOM 4052 ND2 ASN 246 56.022 71.804 28.298 1.00 0.00 N

ATOM 4053 HD21 ASN 246 56.919 71.845 27.836 1.00 0.00 H

ATOM 4054 HD22 ASN 246 55.314 72.479 28.046 1.00 0.00 H

ATOM 4055 C ASN 246 53.363 68.983 28.964 1.00 0.00 C

ATOM 4056 O ASN 246 53.101 68.763 27.754 1.00 0.00 O

ATOM 4057 N LEU 247 53.564 67.992 29.896 1.00 0.00 N

ATOM 4058 H LEU 247 53.991 68.217 30.783 1.00 0.00 H

ATOM 4059 CA LEU 247 53.770 66.544 29.532 1.00 0.00 C

ATOM 4060 HA LEU 247 54.673 66.436 28.932 1.00 0.00 H

ATOM 4061 CB LEU 247 53.889 65.718 30.831 1.00 0.00 C

ATOM 4062 HB2 LEU 247 53.009 65.726 31.473 1.00 0.00 H

ATOM 4063 HB3 LEU 247 53.989 64.669 30.553 1.00 0.00 H

ATOM 4064 CG LEU 247 55.124 66.004 31.690 1.00 0.00 C

ATOM 4065 HG LEU 247 55.256 66.988 32.140 1.00 0.00 H

ATOM 4066 CD1 LEU 247 55.067 65.034 32.888 1.00 0.00 C

ATOM 4067 HD11 LEU 247 55.051 65.685 33.762 1.00 0.00 H

ATOM 4068 HD12 LEU 247 54.210 64.363 32.830 1.00 0.00 H

ATOM 4069 HD13 LEU 247 55.991 64.456 32.899 1.00 0.00 H

ATOM 4070 CD2 LEU 247 56.407 65.607 30.864 1.00 0.00 C

ATOM 4071 HD21 LEU 247 57.248 65.821 31.525 1.00 0.00 H

ATOM 4072 HD22 LEU 247 56.414 64.565 30.545 1.00 0.00 H

ATOM 4073 HD23 LEU 247 56.460 66.306 30.029 1.00 0.00 H

ATOM 4074 C LEU 247 52.589 66.083 28.690 1.00 0.00 C

ATOM 4075 O LEU 247 52.767 65.642 27.552 1.00 0.00 O

ATOM 4076 N ARG 248 51.367 66.159 29.193 1.00 0.00 N

ATOM 4077 H ARG 248 51.152 66.687 30.027 1.00 0.00 H

ATOM 4078 CA ARG 248 50.143 65.883 28.342 1.00 0.00 C

ATOM 4079 HA ARG 248 50.161 64.805 28.178 1.00 0.00 H

ATOM 4080 CB ARG 248 48.884 66.152 29.193 1.00 0.00 C

ATOM 4081 HB2 ARG 248 48.987 65.513 30.070 1.00 0.00 H

ATOM 4082 HB3 ARG 248 48.868 67.196 29.506 1.00 0.00 H

ATOM 4083 CG ARG 248 47.611 65.796 28.521 1.00 0.00 C

ATOM 4084 HG2 ARG 248 46.860 66.199 29.201 1.00 0.00 H

ATOM 4085 HG3 ARG 248 47.659 66.197 27.509 1.00 0.00 H

ATOM 4086 CD ARG 248 47.448 64.256 28.524 1.00 0.00 C

ATOM 4087 HD2 ARG 248 48.351 63.702 28.269 1.00 0.00 H

ATOM 4088 HD3 ARG 248 47.022 63.996 29.493 1.00 0.00 H

ATOM 4089 NE ARG 248 46.351 63.880 27.573 1.00 0.00 N

ATOM 4090 HE ARG 248 46.712 63.256 26.865 1.00 0.00 H

ATOM 4091 CZ ARG 248 45.079 64.241 27.622 1.00 0.00 C

ATOM 4092 NH1 ARG 248 44.610 64.858 28.664 1.00 0.00 N

ATOM 4093 HH11 ARG 248 45.055 64.516 29.503 1.00 0.00 H

ATOM 4094 HH12 ARG 248 43.626 65.081 28.608 1.00 0.00 H

ATOM 4095 NH2 ARG 248 44.236 63.968 26.632 1.00 0.00 N

ATOM 4096 HH21 ARG 248 44.752 63.664 25.819 1.00 0.00 H

ATOM 4097 HH22 ARG 248 43.674 64.764 26.367 1.00 0.00 H

ATOM 4098 C ARG 248 50.065 66.570 26.970 1.00 0.00 C

ATOM 4099 O ARG 248 49.811 65.905 25.977 1.00 0.00 O

ATOM 4100 N ALA 249 50.521 67.788 26.790 1.00 0.00 N

ATOM 4101 H ALA 249 50.876 68.312 27.577 1.00 0.00 H

ATOM 4102 CA ALA 249 50.574 68.424 25.523 1.00 0.00 C

ATOM 4103 HA ALA 249 49.570 68.463 25.100 1.00 0.00 H

ATOM 4104 CB ALA 249 50.871 69.984 25.656 1.00 0.00 C

ATOM 4105 HB1 ALA 249 50.307 70.303 26.532 1.00 0.00 H

ATOM 4106 HB2 ALA 249 51.904 70.209 25.921 1.00 0.00 H

ATOM 4107 HB3 ALA 249 50.633 70.599 24.788 1.00 0.00 H

ATOM 4108 C ALA 249 51.568 67.845 24.499 1.00 0.00 C

ATOM 4109 O ALA 249 51.128 67.652 23.353 1.00 0.00 O

ATOM 4110 N ARG 250 52.724 67.475 25.080 1.00 0.00 N

ATOM 4111 H ARG 250 52.886 67.616 26.067 1.00 0.00 H

ATOM 4112 CA ARG 250 53.720 66.676 24.328 1.00 0.00 C

ATOM 4113 HA ARG 250 53.739 67.223 23.386 1.00 0.00 H

ATOM 4114 CB ARG 250 55.059 66.640 25.041 1.00 0.00 C

ATOM 4115 HB2 ARG 250 54.969 66.194 26.031 1.00 0.00 H

ATOM 4116 HB3 ARG 250 55.746 65.989 24.500 1.00 0.00 H

ATOM 4117 CG ARG 250 55.661 68.076 25.214 1.00 0.00 C

ATOM 4118 HG2 ARG 250 56.353 68.328 24.411 1.00 0.00 H

ATOM 4119 HG3 ARG 250 54.869 68.824 25.210 1.00 0.00 H

ATOM 4120 CD ARG 250 56.524 68.278 26.387 1.00 0.00 C

ATOM 4121 HD2 ARG 250 56.836 69.321 26.329 1.00 0.00 H

ATOM 4122 HD3 ARG 250 55.794 68.255 27.196 1.00 0.00 H

ATOM 4123 NE ARG 250 57.649 67.347 26.519 1.00 0.00 N

ATOM 4124 HE ARG 250 58.081 66.929 25.708 1.00 0.00 H

ATOM 4125 CZ ARG 250 58.330 67.097 27.613 1.00 0.00 C

ATOM 4126 NH1 ARG 250 58.125 67.840 28.643 1.00 0.00 N

ATOM 4127 HH11 ARG 250 57.367 68.497 28.762 1.00 0.00 H

ATOM 4128 HH12 ARG 250 58.678 67.582 29.448 1.00 0.00 H

ATOM 4129 NH2 ARG 250 59.342 66.195 27.691 1.00 0.00 N

ATOM 4130 HH21 ARG 250 59.557 65.553 26.941 1.00 0.00 H

ATOM 4131 HH22 ARG 250 60.103 66.617 28.204 1.00 0.00 H

ATOM 4132 C ARG 250 53.279 65.167 23.931 1.00 0.00 C

ATOM 4133 O ARG 250 53.666 64.703 22.880 1.00 0.00 O

ATOM 4134 N LEU 251 52.429 64.568 24.685 1.00 0.00 N

ATOM 4135 H LEU 251 52.166 65.029 25.544 1.00 0.00 H

ATOM 4136 CA LEU 251 51.916 63.189 24.534 1.00 0.00 C

ATOM 4137 HA LEU 251 52.763 62.554 24.274 1.00 0.00 H

ATOM 4138 CB LEU 251 51.459 62.840 25.966 1.00 0.00 C

ATOM 4139 HB2 LEU 251 52.315 62.853 26.641 1.00 0.00 H

ATOM 4140 HB3 LEU 251 50.736 63.591 26.283 1.00 0.00 H

ATOM 4141 CG LEU 251 50.788 61.458 26.044 1.00 0.00 C

ATOM 4142 HG LEU 251 49.901 61.424 25.412 1.00 0.00 H

ATOM 4143 CD1 LEU 251 51.701 60.249 25.689 1.00 0.00 C

ATOM 4144 HD11 LEU 251 51.816 60.306 24.606 1.00 0.00 H

ATOM 4145 HD12 LEU 251 52.637 60.328 26.241 1.00 0.00 H

ATOM 4146 HD13 LEU 251 51.147 59.318 25.815 1.00 0.00 H

ATOM 4147 CD2 LEU 251 50.357 61.259 27.500 1.00 0.00 C

ATOM 4148 HD21 LEU 251 49.680 60.406 27.488 1.00 0.00 H

ATOM 4149 HD22 LEU 251 51.219 61.066 28.139 1.00 0.00 H

ATOM 4150 HD23 LEU 251 49.719 62.029 27.933 1.00 0.00 H

ATOM 4151 C LEU 251 50.763 63.179 23.494 1.00 0.00 C

ATOM 4152 O LEU 251 50.730 62.403 22.588 1.00 0.00 O

ATOM 4153 N ASP 252 49.815 64.129 23.605 1.00 0.00 N

ATOM 4154 H ASP 252 50.114 64.868 24.224 1.00 0.00 H

ATOM 4155 CA ASP 252 48.532 64.237 22.913 1.00 0.00 C

ATOM 4156 HA ASP 252 48.474 63.644 22.000 1.00 0.00 H

ATOM 4157 CB ASP 252 47.566 63.507 23.780 1.00 0.00 C

ATOM 4158 HB2 ASP 252 47.810 62.448 23.852 1.00 0.00 H

ATOM 4159 HB3 ASP 252 47.596 63.882 24.803 1.00 0.00 H

ATOM 4160 CG ASP 252 46.171 63.566 23.156 1.00 0.00 C

ATOM 4161 OD1 ASP 252 45.317 63.593 24.067 1.00 0.00 O

ATOM 4162 OD2 ASP 252 45.931 63.276 21.945 1.00 0.00 O

ATOM 4163 C ASP 252 48.078 65.673 22.549 1.00 0.00 C

ATOM 4164 O ASP 252 47.863 66.029 21.350 1.00 0.00 O

ATOM 4165 N PHE 253 47.748 66.572 23.449 1.00 0.00 N

ATOM 4166 H PHE 253 48.150 66.413 24.362 1.00 0.00 H

ATOM 4167 CA PHE 253 46.779 67.694 23.234 1.00 0.00 C

ATOM 4168 HA PHE 253 45.887 67.230 22.812 1.00 0.00 H

ATOM 4169 CB PHE 253 46.473 68.335 24.625 1.00 0.00 C

ATOM 4170 HB2 PHE 253 45.842 67.659 25.202 1.00 0.00 H

ATOM 4171 HB3 PHE 253 47.362 68.394 25.253 1.00 0.00 H

ATOM 4172 CG PHE 253 45.752 69.729 24.620 1.00 0.00 C

ATOM 4173 CD1 PHE 253 44.345 69.834 24.594 1.00 0.00 C

ATOM 4174 HD1 PHE 253 43.768 68.921 24.624 1.00 0.00 H

ATOM 4175 CE1 PHE 253 43.736 71.124 24.683 1.00 0.00 C

ATOM 4176 HE1 PHE 253 42.690 71.263 24.450 1.00 0.00 H

ATOM 4177 CZ PHE 253 44.491 72.334 24.601 1.00 0.00 C

ATOM 4178 HZ PHE 253 44.039 73.269 24.895 1.00 0.00 H

ATOM 4179 CE2 PHE 253 45.847 72.230 24.837 1.00 0.00 C

ATOM 4180 HE2 PHE 253 46.469 73.109 24.925 1.00 0.00 H

ATOM 4181 CD2 PHE 253 46.463 70.952 24.725 1.00 0.00 C

ATOM 4182 HD2 PHE 253 47.542 70.942 24.773 1.00 0.00 H

ATOM 4183 C PHE 253 47.260 68.866 22.329 1.00 0.00 C

ATOM 4184 O PHE 253 46.439 69.504 21.590 1.00 0.00 O

ATOM 4185 N GLN 254 48.601 68.953 22.037 1.00 0.00 N

ATOM 4186 H GLN 254 49.297 68.309 22.382 1.00 0.00 H

ATOM 4187 CA GLN 254 49.178 69.932 21.059 1.00 0.00 C

ATOM 4188 HA GLN 254 48.644 70.869 21.217 1.00 0.00 H

ATOM 4189 CB GLN 254 50.641 70.246 21.384 1.00 0.00 C

ATOM 4190 HB2 GLN 254 50.724 70.446 22.452 1.00 0.00 H

ATOM 4191 HB3 GLN 254 51.296 69.399 21.181 1.00 0.00 H

ATOM 4192 CG GLN 254 51.136 71.371 20.537 1.00 0.00 C

ATOM 4193 HG2 GLN 254 51.251 71.063 19.497 1.00 0.00 H

ATOM 4194 HG3 GLN 254 50.561 72.291 20.640 1.00 0.00 H

ATOM 4195 CD GLN 254 52.483 71.747 21.023 1.00 0.00 C

ATOM 4196 OE1 GLN 254 52.610 72.661 21.883 1.00 0.00 O

ATOM 4197 NE2 GLN 254 53.466 71.081 20.589 1.00 0.00 N

ATOM 4198 HE21 GLN 254 54.338 71.179 21.089 1.00 0.00 H

ATOM 4199 HE22 GLN 254 53.307 70.310 19.957 1.00 0.00 H

ATOM 4200 C GLN 254 48.938 69.678 19.576 1.00 0.00 C

ATOM 4201 O GLN 254 49.737 69.038 18.890 1.00 0.00 O

ATOM 4202 N THR 255 47.922 70.216 19.040 1.00 0.00 N

ATOM 4203 H THR 255 47.062 70.163 19.567 1.00 0.00 H

ATOM 4204 CA THR 255 47.736 70.725 17.685 1.00 0.00 C

ATOM 4205 HA THR 255 48.030 69.996 16.930 1.00 0.00 H

ATOM 4206 CB THR 255 46.309 70.965 17.239 1.00 0.00 C

ATOM 4207 HB THR 255 46.114 70.983 16.167 1.00 0.00 H

ATOM 4208 CG2 THR 255 45.373 69.836 17.563 1.00 0.00 C

ATOM 4209 HG21 THR 255 45.725 68.870 17.200 1.00 0.00 H

ATOM 4210 HG22 THR 255 45.303 69.822 18.651 1.00 0.00 H

ATOM 4211 HG23 THR 255 44.358 70.021 17.210 1.00 0.00 H

ATOM 4212 OG1 THR 255 45.762 72.028 17.998 1.00 0.00 O

ATOM 4213 HG1 THR 255 44.862 72.014 17.664 1.00 0.00 H

ATOM 4214 C THR 255 48.566 72.000 17.437 1.00 0.00 C

ATOM 4215 O THR 255 48.978 72.664 18.359 1.00 0.00 O

ATOM 4216 N PRO 256 48.788 72.356 16.136 1.00 0.00 N

ATOM 4217 CD PRO 256 48.828 71.425 15.024 1.00 0.00 C

ATOM 4218 HD2 PRO 256 47.984 70.739 14.949 1.00 0.00 H

ATOM 4219 HD3 PRO 256 49.618 70.674 15.053 1.00 0.00 H

ATOM 4220 CG PRO 256 48.821 72.214 13.703 1.00 0.00 C

ATOM 4221 HG2 PRO 256 47.847 71.982 13.271 1.00 0.00 H

ATOM 4222 HG3 PRO 256 49.658 72.078 13.018 1.00 0.00 H

ATOM 4223 CB PRO 256 48.950 73.675 14.193 1.00 0.00 C

ATOM 4224 HB2 PRO 256 47.980 74.158 14.079 1.00 0.00 H

ATOM 4225 HB3 PRO 256 49.659 74.324 13.679 1.00 0.00 H

ATOM 4226 CA PRO 256 49.126 73.645 15.657 1.00 0.00 C

ATOM 4227 HA PRO 256 50.177 73.720 15.935 1.00 0.00 H

ATOM 4228 C PRO 256 48.437 74.767 16.351 1.00 0.00 C

ATOM 4229 O PRO 256 49.107 75.709 16.698 1.00 0.00 O

ATOM 4230 N ALA 257 47.105 74.755 16.421 1.00 0.00 N

ATOM 4231 H ALA 257 46.639 74.027 15.899 1.00 0.00 H

ATOM 4232 CA ALA 257 46.385 75.910 16.970 1.00 0.00 C

ATOM 4233 HA ALA 257 46.722 76.823 16.479 1.00 0.00 H

ATOM 4234 CB ALA 257 44.898 75.686 16.482 1.00 0.00 C

ATOM 4235 HB1 ALA 257 44.195 76.446 16.823 1.00 0.00 H

ATOM 4236 HB2 ALA 257 44.862 75.661 15.393 1.00 0.00 H

ATOM 4237 HB3 ALA 257 44.541 74.774 16.960 1.00 0.00 H

ATOM 4238 C ALA 257 46.522 76.003 18.549 1.00 0.00 C

ATOM 4239 O ALA 257 46.510 77.139 19.104 1.00 0.00 O

ATOM 4240 N MET 258 46.736 74.925 19.260 1.00 0.00 N

ATOM 4241 H MET 258 46.789 73.998 18.863 1.00 0.00 H

ATOM 4242 CA MET 258 47.182 74.938 20.660 1.00 0.00 C

ATOM 4243 HA MET 258 46.580 75.644 21.232 1.00 0.00 H

ATOM 4244 CB MET 258 46.935 73.555 21.212 1.00 0.00 C

ATOM 4245 HB2 MET 258 47.387 72.652 20.803 1.00 0.00 H

ATOM 4246 HB3 MET 258 47.227 73.598 22.262 1.00 0.00 H

ATOM 4247 CG MET 258 45.414 73.263 21.281 1.00 0.00 C

ATOM 4248 HG2 MET 258 44.992 73.012 20.308 1.00 0.00 H

ATOM 4249 HG3 MET 258 45.267 72.355 21.866 1.00 0.00 H

ATOM 4250 SD MET 258 44.292 74.512 22.074 1.00 0.00 S

ATOM 4251 CE MET 258 42.823 73.525 21.947 1.00 0.00 C

ATOM 4252 HE1 MET 258 42.998 72.454 21.848 1.00 0.00 H

ATOM 4253 HE2 MET 258 42.280 73.710 22.874 1.00 0.00 H

ATOM 4254 HE3 MET 258 42.334 73.798 21.011 1.00 0.00 H

ATOM 4255 C MET 258 48.618 75.346 20.946 1.00 0.00 C

ATOM 4256 O MET 258 48.966 75.632 22.095 1.00 0.00 O

ATOM 4257 N CYX 259 49.445 75.458 19.892 1.00 0.00 N

ATOM 4258 H CYX 259 49.103 74.962 19.081 1.00 0.00 H

ATOM 4259 CA CYX 259 50.899 75.646 20.102 1.00 0.00 C

ATOM 4260 HA CYX 259 51.380 74.800 20.593 1.00 0.00 H

ATOM 4261 CB CYX 259 51.704 75.608 18.767 1.00 0.00 C

ATOM 4262 HB2 CYX 259 51.554 74.629 18.314 1.00 0.00 H

ATOM 4263 HB3 CYX 259 51.336 76.298 18.008 1.00 0.00 H

ATOM 4264 SG CYX 259 53.483 75.912 18.949 1.00 0.00 S

ATOM 4265 C CYX 259 51.221 76.909 20.895 1.00 0.00 C

ATOM 4266 O CYX 259 52.087 76.851 21.791 1.00 0.00 O

ATOM 4267 N ALA 260 50.670 78.003 20.527 1.00 0.00 N

ATOM 4268 H ALA 260 50.035 77.850 19.757 1.00 0.00 H

ATOM 4269 CA ALA 260 50.908 79.366 21.066 1.00 0.00 C

ATOM 4270 HA ALA 260 51.955 79.671 21.042 1.00 0.00 H

ATOM 4271 CB ALA 260 50.200 80.360 20.209 1.00 0.00 C

ATOM 4272 HB1 ALA 260 50.529 80.251 19.176 1.00 0.00 H

ATOM 4273 HB2 ALA 260 49.133 80.191 20.352 1.00 0.00 H

ATOM 4274 HB3 ALA 260 50.449 81.367 20.543 1.00 0.00 H

ATOM 4275 C ALA 260 50.500 79.304 22.608 1.00 0.00 C

ATOM 4276 O ALA 260 51.274 79.788 23.460 1.00 0.00 O

ATOM 4277 N PHE 261 49.341 78.755 22.967 1.00 0.00 N

ATOM 4278 H PHE 261 48.806 78.450 22.166 1.00 0.00 H

ATOM 4279 CA PHE 261 48.850 78.638 24.393 1.00 0.00 C

ATOM 4280 HA PHE 261 48.726 79.609 24.871 1.00 0.00 H

ATOM 4281 CB PHE 261 47.484 77.931 24.281 1.00 0.00 C

ATOM 4282 HB2 PHE 261 46.727 78.713 24.225 1.00 0.00 H

ATOM 4283 HB3 PHE 261 47.558 77.281 23.409 1.00 0.00 H

ATOM 4284 CG PHE 261 47.117 77.255 25.605 1.00 0.00 C

ATOM 4285 CD1 PHE 261 46.939 75.841 25.610 1.00 0.00 C

ATOM 4286 HD1 PHE 261 46.881 75.264 24.699 1.00 0.00 H

ATOM 4287 CE1 PHE 261 46.403 75.287 26.809 1.00 0.00 C

ATOM 4288 HE1 PHE 261 46.119 74.255 26.960 1.00 0.00 H

ATOM 4289 CZ PHE 261 46.139 76.143 27.923 1.00 0.00 C

ATOM 4290 HZ PHE 261 45.846 75.657 28.842 1.00 0.00 H

ATOM 4291 CE2 PHE 261 46.240 77.520 27.876 1.00 0.00 C

ATOM 4292 HE2 PHE 261 45.903 78.038 28.762 1.00 0.00 H

ATOM 4293 CD2 PHE 261 46.913 78.064 26.767 1.00 0.00 C

ATOM 4294 HD2 PHE 261 47.143 79.117 26.687 1.00 0.00 H

ATOM 4295 C PHE 261 49.913 77.843 25.121 1.00 0.00 C

ATOM 4296 O PHE 261 50.316 78.209 26.232 1.00 0.00 O

ATOM 4297 N ASN 262 50.380 76.658 24.558 1.00 0.00 N

ATOM 4298 H ASN 262 50.168 76.521 23.580 1.00 0.00 H

ATOM 4299 CA ASN 262 51.330 75.707 25.183 1.00 0.00 C

ATOM 4300 HA ASN 262 50.939 75.394 26.151 1.00 0.00 H

ATOM 4301 CB ASN 262 51.459 74.387 24.421 1.00 0.00 C

ATOM 4302 HB2 ASN 262 50.481 74.229 23.965 1.00 0.00 H

ATOM 4303 HB3 ASN 262 52.240 74.502 23.669 1.00 0.00 H

ATOM 4304 CG ASN 262 51.910 73.298 25.261 1.00 0.00 C

ATOM 4305 OD1 ASN 262 51.525 73.160 26.389 1.00 0.00 O

ATOM 4306 ND2 ASN 262 52.928 72.602 24.804 1.00 0.00 N

ATOM 4307 HD21 ASN 262 53.173 71.857 25.441 1.00 0.00 H

ATOM 4308 HD22 ASN 262 53.013 72.543 23.799 1.00 0.00 H

ATOM 4309 C ASN 262 52.697 76.343 25.566 1.00 0.00 C

ATOM 4310 O ASN 262 53.365 75.850 26.531 1.00 0.00 O

ATOM 4311 N ASP 263 53.187 77.322 24.703 1.00 0.00 N

ATOM 4312 H ASP 263 52.731 77.345 23.802 1.00 0.00 H

ATOM 4313 CA ASP 263 54.378 78.153 24.859 1.00 0.00 C

ATOM 4314 HA ASP 263 55.118 77.451 25.242 1.00 0.00 H

ATOM 4315 CB ASP 263 54.708 78.773 23.466 1.00 0.00 C

ATOM 4316 HB2 ASP 263 54.815 77.986 22.719 1.00 0.00 H

ATOM 4317 HB3 ASP 263 53.928 79.454 23.125 1.00 0.00 H

ATOM 4318 CG ASP 263 56.032 79.521 23.448 1.00 0.00 C

ATOM 4319 OD1 ASP 263 57.012 79.087 24.053 1.00 0.00 O

ATOM 4320 OD2 ASP 263 56.085 80.664 22.898 1.00 0.00 O

ATOM 4321 C ASP 263 54.163 79.192 25.997 1.00 0.00 C

ATOM 4322 O ASP 263 55.112 79.437 26.722 1.00 0.00 O

ATOM 4323 N ARG 264 52.890 79.705 26.307 1.00 0.00 N

ATOM 4324 H ARG 264 52.214 79.734 25.557 1.00 0.00 H

ATOM 4325 CA ARG 264 52.562 80.462 27.523 1.00 0.00 C

ATOM 4326 HA ARG 264 53.416 81.124 27.665 1.00 0.00 H

ATOM 4327 CB ARG 264 51.243 81.256 27.449 1.00 0.00 C

ATOM 4328 HB2 ARG 264 50.318 80.680 27.413 1.00 0.00 H

ATOM 4329 HB3 ARG 264 51.165 81.811 28.384 1.00 0.00 H

ATOM 4330 CG ARG 264 51.135 82.239 26.227 1.00 0.00 C

ATOM 4331 HG2 ARG 264 52.025 82.869 26.219 1.00 0.00 H

ATOM 4332 HG3 ARG 264 51.007 81.609 25.346 1.00 0.00 H

ATOM 4333 CD ARG 264 49.928 83.192 26.288 1.00 0.00 C

ATOM 4334 HD2 ARG 264 49.004 82.628 26.419 1.00 0.00 H

ATOM 4335 HD3 ARG 264 50.134 83.670 27.245 1.00 0.00 H

ATOM 4336 NE ARG 264 49.879 84.250 25.364 1.00 0.00 N

ATOM 4337 HE ARG 264 50.226 85.188 25.504 1.00 0.00 H

ATOM 4338 CZ ARG 264 49.538 84.013 24.153 1.00 0.00 C

ATOM 4339 NH1 ARG 264 48.937 82.927 23.737 1.00 0.00 N

ATOM 4340 HH11 ARG 264 48.811 82.863 22.737 1.00 0.00 H

ATOM 4341 HH12 ARG 264 48.262 82.602 24.414 1.00 0.00 H

ATOM 4342 NH2 ARG 264 50.074 84.764 23.238 1.00 0.00 N

ATOM 4343 HH21 ARG 264 50.854 85.348 23.503 1.00 0.00 H

ATOM 4344 HH22 ARG 264 49.945 84.563 22.256 1.00 0.00 H

ATOM 4345 C ARG 264 52.574 79.560 28.728 1.00 0.00 C

ATOM 4346 O ARG 264 53.088 79.903 29.794 1.00 0.00 O

ATOM 4347 N VAL 265 51.964 78.365 28.691 1.00 0.00 N

ATOM 4348 H VAL 265 51.719 78.009 27.779 1.00 0.00 H

ATOM 4349 CA VAL 265 51.985 77.382 29.731 1.00 0.00 C

ATOM 4350 HA VAL 265 51.741 77.897 30.661 1.00 0.00 H

ATOM 4351 CB VAL 265 51.048 76.226 29.467 1.00 0.00 C

ATOM 4352 HB VAL 265 51.466 75.739 28.586 1.00 0.00 H

ATOM 4353 CG1 VAL 265 51.047 75.170 30.589 1.00 0.00 C

ATOM 4354 HG11 VAL 265 50.510 75.411 31.507 1.00 0.00 H

ATOM 4355 HG12 VAL 265 50.586 74.224 30.303 1.00 0.00 H

ATOM 4356 HG13 VAL 265 52.029 74.854 30.942 1.00 0.00 H

ATOM 4357 CG2 VAL 265 49.603 76.661 29.171 1.00 0.00 C

ATOM 4358 HG21 VAL 265 49.046 76.943 30.065 1.00 0.00 H

ATOM 4359 HG22 VAL 265 49.569 77.575 28.578 1.00 0.00 H

ATOM 4360 HG23 VAL 265 49.082 75.876 28.622 1.00 0.00 H

ATOM 4361 C VAL 265 53.404 76.963 29.933 1.00 0.00 C

ATOM 4362 O VAL 265 53.791 76.848 31.039 1.00 0.00 O

ATOM 4363 N TYR 266 54.251 76.860 28.908 1.00 0.00 N

ATOM 4364 H TYR 266 53.943 77.034 27.962 1.00 0.00 H

ATOM 4365 CA TYR 266 55.755 76.651 29.028 1.00 0.00 C

ATOM 4366 HA TYR 266 55.945 75.765 29.634 1.00 0.00 H

ATOM 4367 CB TYR 266 56.592 76.401 27.725 1.00 0.00 C

ATOM 4368 HB2 TYR 266 56.132 75.630 27.108 1.00 0.00 H

ATOM 4369 HB3 TYR 266 56.605 77.356 27.200 1.00 0.00 H

ATOM 4370 CG TYR 266 58.093 76.054 27.905 1.00 0.00 C

ATOM 4371 CD1 TYR 266 58.608 75.065 28.709 1.00 0.00 C

ATOM 4372 HD1 TYR 266 57.957 74.325 29.152 1.00 0.00 H

ATOM 4373 CE1 TYR 266 59.993 74.845 28.854 1.00 0.00 C

ATOM 4374 HE1 TYR 266 60.390 73.944 29.298 1.00 0.00 H

ATOM 4375 CZ TYR 266 60.925 75.635 28.094 1.00 0.00 C

ATOM 4376 OH TYR 266 62.196 75.213 28.090 1.00 0.00 O

ATOM 4377 HH TYR 266 62.187 74.303 28.396 1.00 0.00 H

ATOM 4378 CE2 TYR 266 60.457 76.744 27.385 1.00 0.00 C

ATOM 4379 HE2 TYR 266 61.119 77.497 26.983 1.00 0.00 H

ATOM 4380 CD2 TYR 266 59.048 76.881 27.272 1.00 0.00 C

ATOM 4381 HD2 TYR 266 58.673 77.761 26.770 1.00 0.00 H

ATOM 4382 C TYR 266 56.477 77.768 29.779 1.00 0.00 C

ATOM 4383 O TYR 266 57.207 77.502 30.724 1.00 0.00 O

ATOM 4384 N ALA 267 56.302 79.065 29.422 1.00 0.00 N

ATOM 4385 H ALA 267 55.621 79.234 28.695 1.00 0.00 H

ATOM 4386 CA ALA 267 56.630 80.230 30.293 1.00 0.00 C

ATOM 4387 HA ALA 267 57.705 80.350 30.423 1.00 0.00 H

ATOM 4388 CB ALA 267 56.101 81.446 29.569 1.00 0.00 C

ATOM 4389 HB1 ALA 267 56.289 81.451 28.496 1.00 0.00 H

ATOM 4390 HB2 ALA 267 55.029 81.597 29.696 1.00 0.00 H

ATOM 4391 HB3 ALA 267 56.667 82.302 29.937 1.00 0.00 H

ATOM 4392 C ALA 267 56.172 80.074 31.719 1.00 0.00 C

ATOM 4393 O ALA 267 57.001 80.097 32.659 1.00 0.00 O

ATOM 4394 N THR 268 54.931 79.750 31.829 1.00 0.00 N

ATOM 4395 H THR 268 54.322 80.167 31.140 1.00 0.00 H

ATOM 4396 CA THR 268 54.438 79.522 33.227 1.00 0.00 C

ATOM 4397 HA THR 268 54.755 80.349 33.861 1.00 0.00 H

ATOM 4398 CB THR 268 52.945 79.378 33.308 1.00 0.00 C

ATOM 4399 HB THR 268 52.641 78.350 33.113 1.00 0.00 H

ATOM 4400 CG2 THR 268 52.475 79.854 34.641 1.00 0.00 C

ATOM 4401 HG21 THR 268 52.740 80.911 34.654 1.00 0.00 H

ATOM 4402 HG22 THR 268 51.388 79.863 34.717 1.00 0.00 H

ATOM 4403 HG23 THR 268 52.925 79.297 35.463 1.00 0.00 H

ATOM 4404 OG1 THR 268 52.327 80.239 32.421 1.00 0.00 O

ATOM 4405 HG1 THR 268 52.553 79.995 31.520 1.00 0.00 H

ATOM 4406 C THR 268 55.068 78.338 33.940 1.00 0.00 C

ATOM 4407 O THR 268 55.491 78.436 35.074 1.00 0.00 O

ATOM 4408 N TYR 269 55.389 77.275 33.255 1.00 0.00 N

ATOM 4409 H TYR 269 55.343 77.379 32.252 1.00 0.00 H

ATOM 4410 CA TYR 269 56.054 76.077 33.793 1.00 0.00 C

ATOM 4411 HA TYR 269 55.585 75.638 34.674 1.00 0.00 H

ATOM 4412 CB TYR 269 56.042 74.961 32.686 1.00 0.00 C

ATOM 4413 HB2 TYR 269 55.059 74.562 32.435 1.00 0.00 H

ATOM 4414 HB3 TYR 269 56.253 75.464 31.742 1.00 0.00 H

ATOM 4415 CG TYR 269 57.027 73.851 32.811 1.00 0.00 C

ATOM 4416 CD1 TYR 269 58.281 73.916 32.146 1.00 0.00 C

ATOM 4417 HD1 TYR 269 58.639 74.801 31.641 1.00 0.00 H

ATOM 4418 CE1 TYR 269 59.194 72.829 32.332 1.00 0.00 C

ATOM 4419 HE1 TYR 269 60.164 72.825 31.858 1.00 0.00 H

ATOM 4420 CZ TYR 269 58.842 71.720 33.132 1.00 0.00 C

ATOM 4421 OH TYR 269 59.667 70.697 33.372 1.00 0.00 O

ATOM 4422 HH TYR 269 60.527 70.941 33.021 1.00 0.00 H

ATOM 4423 CE2 TYR 269 57.543 71.702 33.810 1.00 0.00 C

ATOM 4424 HE2 TYR 269 57.204 70.859 34.394 1.00 0.00 H

ATOM 4425 CD2 TYR 269 56.595 72.733 33.555 1.00 0.00 C

ATOM 4426 HD2 TYR 269 55.589 72.726 33.949 1.00 0.00 H

ATOM 4427 C TYR 269 57.504 76.412 34.241 1.00 0.00 C

ATOM 4428 O TYR 269 57.896 76.146 35.378 1.00 0.00 O

ATOM 4429 N GLN 270 58.268 77.179 33.396 1.00 0.00 N

ATOM 4430 H GLN 270 57.966 77.234 32.434 1.00 0.00 H

ATOM 4431 CA GLN 270 59.574 77.777 33.777 1.00 0.00 C

ATOM 4432 HA GLN 270 60.208 76.961 34.124 1.00 0.00 H

ATOM 4433 CB GLN 270 60.274 78.410 32.600 1.00 0.00 C

ATOM 4434 HB2 GLN 270 59.477 79.033 32.196 1.00 0.00 H

ATOM 4435 HB3 GLN 270 61.141 78.942 32.995 1.00 0.00 H

ATOM 4436 CG GLN 270 60.610 77.490 31.451 1.00 0.00 C

ATOM 4437 HG2 GLN 270 59.800 76.768 31.347 1.00 0.00 H

ATOM 4438 HG3 GLN 270 60.895 78.062 30.568 1.00 0.00 H

ATOM 4439 CD GLN 270 61.758 76.542 31.799 1.00 0.00 C

ATOM 4440 OE1 GLN 270 61.629 75.757 32.713 1.00 0.00 O

ATOM 4441 NE2 GLN 270 62.969 76.742 31.282 1.00 0.00 N

ATOM 4442 HE21 GLN 270 63.671 76.087 31.596 1.00 0.00 H

ATOM 4443 HE22 GLN 270 62.929 77.194 30.380 1.00 0.00 H

ATOM 4444 C GLN 270 59.421 78.832 34.869 1.00 0.00 C

ATOM 4445 O GLN 270 60.228 78.762 35.799 1.00 0.00 O

ATOM 4446 N VAL 271 58.453 79.737 34.943 1.00 0.00 N

ATOM 4447 H VAL 271 57.915 79.939 34.113 1.00 0.00 H

ATOM 4448 CA VAL 271 58.112 80.542 36.123 1.00 0.00 C

ATOM 4449 HA VAL 271 58.949 81.198 36.361 1.00 0.00 H

ATOM 4450 CB VAL 271 56.995 81.652 35.811 1.00 0.00 C

ATOM 4451 HB VAL 271 56.214 81.105 35.283 1.00 0.00 H

ATOM 4452 CG1 VAL 271 56.452 82.365 37.143 1.00 0.00 C

ATOM 4453 HG11 VAL 271 56.027 83.363 37.034 1.00 0.00 H

ATOM 4454 HG12 VAL 271 55.735 81.734 37.667 1.00 0.00 H

ATOM 4455 HG13 VAL 271 57.271 82.560 37.836 1.00 0.00 H

ATOM 4456 CG2 VAL 271 57.493 82.867 34.973 1.00 0.00 C

ATOM 4457 HG21 VAL 271 58.220 83.397 35.588 1.00 0.00 H

ATOM 4458 HG22 VAL 271 57.924 82.414 34.081 1.00 0.00 H

ATOM 4459 HG23 VAL 271 56.658 83.436 34.563 1.00 0.00 H

ATOM 4460 C VAL 271 57.766 79.831 37.406 1.00 0.00 C

ATOM 4461 O VAL 271 58.250 80.152 38.511 1.00 0.00 O

ATOM 4462 N THR 272 57.074 78.692 37.347 1.00 0.00 N

ATOM 4463 H THR 272 56.727 78.420 36.439 1.00 0.00 H

ATOM 4464 CA THR 272 56.665 77.826 38.509 1.00 0.00 C

ATOM 4465 HA THR 272 56.562 78.508 39.353 1.00 0.00 H

ATOM 4466 CB THR 272 55.413 77.058 38.363 1.00 0.00 C

ATOM 4467 HB THR 272 55.524 76.337 39.173 1.00 0.00 H

ATOM 4468 CG2 THR 272 54.053 77.697 38.596 1.00 0.00 C

ATOM 4469 HG21 THR 272 53.924 78.524 37.898 1.00 0.00 H

ATOM 4470 HG22 THR 272 53.249 77.018 38.313 1.00 0.00 H

ATOM 4471 HG23 THR 272 53.924 77.967 39.644 1.00 0.00 H

ATOM 4472 OG1 THR 272 55.321 76.385 37.121 1.00 0.00 O

ATOM 4473 HG1 THR 272 55.265 77.148 36.541 1.00 0.00 H

ATOM 4474 C THR 272 57.818 76.987 38.933 1.00 0.00 C

ATOM 4475 O THR 272 57.848 76.740 40.128 1.00 0.00 O

ATOM 4476 N ARG 273 58.783 76.554 38.103 1.00 0.00 N

ATOM 4477 H ARG 273 58.607 76.523 37.108 1.00 0.00 H

ATOM 4478 CA ARG 273 60.082 76.162 38.583 1.00 0.00 C

ATOM 4479 HA ARG 273 59.925 75.267 39.185 1.00 0.00 H

ATOM 4480 CB ARG 273 60.954 75.579 37.394 1.00 0.00 C

ATOM 4481 HB2 ARG 273 60.310 75.349 36.545 1.00 0.00 H

ATOM 4482 HB3 ARG 273 61.741 76.219 36.992 1.00 0.00 H

ATOM 4483 CG ARG 273 61.767 74.294 37.804 1.00 0.00 C

ATOM 4484 HG2 ARG 273 62.210 74.507 38.776 1.00 0.00 H

ATOM 4485 HG3 ARG 273 61.197 73.381 37.976 1.00 0.00 H

ATOM 4486 CD ARG 273 62.986 74.083 36.827 1.00 0.00 C

ATOM 4487 HD2 ARG 273 63.760 74.833 36.991 1.00 0.00 H

ATOM 4488 HD3 ARG 273 63.491 73.152 37.083 1.00 0.00 H

ATOM 4489 NE ARG 273 62.536 74.069 35.456 1.00 0.00 N

ATOM 4490 HE ARG 273 62.297 74.900 34.934 1.00 0.00 H

ATOM 4491 CZ ARG 273 62.670 73.047 34.652 1.00 0.00 C

ATOM 4492 NH1 ARG 273 63.008 71.848 35.088 1.00 0.00 N

ATOM 4493 HH11 ARG 273 63.168 71.700 36.074 1.00 0.00 H

ATOM 4494 HH12 ARG 273 62.855 71.089 34.439 1.00 0.00 H

ATOM 4495 NH2 ARG 273 62.358 73.141 33.387 1.00 0.00 N

ATOM 4496 HH21 ARG 273 61.957 73.960 32.954 1.00 0.00 H

ATOM 4497 HH22 ARG 273 62.481 72.367 32.749 1.00 0.00 H

ATOM 4498 C ARG 273 60.905 77.098 39.452 1.00 0.00 C

ATOM 4499 O ARG 273 61.375 76.686 40.508 1.00 0.00 O

ATOM 4500 N GLY 274 60.704 78.320 39.076 1.00 0.00 N

ATOM 4501 H GLY 274 60.424 78.572 38.139 1.00 0.00 H

ATOM 4502 CA GLY 274 60.905 79.378 40.004 1.00 0.00 C

ATOM 4503 HA2 GLY 274 61.989 79.431 40.095 1.00 0.00 H

ATOM 4504 HA3 GLY 274 60.613 80.313 39.526 1.00 0.00 H

ATOM 4505 C GLY 274 60.200 79.305 41.410 1.00 0.00 C

ATOM 4506 O GLY 274 60.864 79.400 42.475 1.00 0.00 O

ATOM 4507 N LEU 275 58.878 79.021 41.435 1.00 0.00 N

ATOM 4508 H LEU 275 58.447 78.655 40.598 1.00 0.00 H

ATOM 4509 CA LEU 275 57.988 79.049 42.629 1.00 0.00 C

ATOM 4510 HA LEU 275 58.380 79.919 43.155 1.00 0.00 H

ATOM 4511 CB LEU 275 56.519 79.175 42.230 1.00 0.00 C

ATOM 4512 HB2 LEU 275 56.400 78.594 41.316 1.00 0.00 H

ATOM 4513 HB3 LEU 275 55.915 78.562 42.899 1.00 0.00 H

ATOM 4514 CG LEU 275 55.954 80.649 42.013 1.00 0.00 C

ATOM 4515 HG LEU 275 56.828 81.188 41.648 1.00 0.00 H

ATOM 4516 CD1 LEU 275 54.754 80.856 41.110 1.00 0.00 C

ATOM 4517 HD11 LEU 275 54.638 81.931 40.970 1.00 0.00 H

ATOM 4518 HD12 LEU 275 54.752 80.307 40.168 1.00 0.00 H

ATOM 4519 HD13 LEU 275 53.834 80.501 41.575 1.00 0.00 H

ATOM 4520 CD2 LEU 275 55.575 81.169 43.338 1.00 0.00 C

ATOM 4521 HD21 LEU 275 54.871 80.477 43.800 1.00 0.00 H

ATOM 4522 HD22 LEU 275 56.424 81.263 44.015 1.00 0.00 H

ATOM 4523 HD23 LEU 275 55.138 82.167 43.286 1.00 0.00 H

ATOM 4524 C LEU 275 58.197 77.855 43.515 1.00 0.00 C

ATOM 4525 O LEU 275 58.487 78.004 44.695 1.00 0.00 O

ATOM 4526 N ALA 276 58.147 76.650 43.010 1.00 0.00 N

ATOM 4527 H ALA 276 57.896 76.578 42.034 1.00 0.00 H

ATOM 4528 CA ALA 276 58.705 75.424 43.637 1.00 0.00 C

ATOM 4529 HA ALA 276 58.023 75.105 44.426 1.00 0.00 H

ATOM 4530 CB ALA 276 58.642 74.375 42.497 1.00 0.00 C

ATOM 4531 HB1 ALA 276 57.689 73.861 42.371 1.00 0.00 H

ATOM 4532 HB2 ALA 276 58.999 74.866 41.592 1.00 0.00 H

ATOM 4533 HB3 ALA 276 59.354 73.588 42.743 1.00 0.00 H

ATOM 4534 C ALA 276 60.159 75.527 44.057 1.00 0.00 C

ATOM 4535 O ALA 276 60.767 74.549 44.516 1.00 0.00 O

ATOM 4536 N SER 277 60.942 76.549 43.595 1.00 0.00 N

ATOM 4537 H SER 277 60.510 77.126 42.888 1.00 0.00 H

ATOM 4538 CA SER 277 62.341 76.892 44.137 1.00 0.00 C

ATOM 4539 HA SER 277 62.729 75.988 44.606 1.00 0.00 H

ATOM 4540 CB SER 277 63.333 77.345 43.096 1.00 0.00 C

ATOM 4541 HB2 SER 277 63.066 78.305 42.653 1.00 0.00 H

ATOM 4542 HB3 SER 277 64.302 77.420 43.588 1.00 0.00 H

ATOM 4543 OG SER 277 63.573 76.377 41.992 1.00 0.00 O

ATOM 4544 HG SER 277 63.050 76.649 41.234 1.00 0.00 H

ATOM 4545 C SER 277 62.304 77.982 45.223 1.00 0.00 C

ATOM 4546 O SER 277 63.326 78.206 45.852 1.00 0.00 O

ATOM 4547 N LEU 278 61.192 78.573 45.559 1.00 0.00 N

ATOM 4548 H LEU 278 60.308 78.127 45.359 1.00 0.00 H

ATOM 4549 CA LEU 278 61.156 79.605 46.702 1.00 0.00 C

ATOM 4550 HA LEU 278 61.798 80.467 46.523 1.00 0.00 H

ATOM 4551 CB LEU 278 59.708 80.225 46.581 1.00 0.00 C

ATOM 4552 HB2 LEU 278 59.611 80.622 45.571 1.00 0.00 H

ATOM 4553 HB3 LEU 278 58.984 79.413 46.655 1.00 0.00 H

ATOM 4554 CG LEU 278 59.319 81.296 47.644 1.00 0.00 C

ATOM 4555 HG LEU 278 59.362 81.002 48.692 1.00 0.00 H

ATOM 4556 CD1 LEU 278 60.244 82.574 47.617 1.00 0.00 C

ATOM 4557 HD11 LEU 278 59.842 83.286 48.338 1.00 0.00 H

ATOM 4558 HD12 LEU 278 61.245 82.250 47.904 1.00 0.00 H

ATOM 4559 HD13 LEU 278 60.361 82.962 46.605 1.00 0.00 H

ATOM 4560 CD2 LEU 278 57.891 81.657 47.518 1.00 0.00 C

ATOM 4561 HD21 LEU 278 57.270 80.778 47.692 1.00 0.00 H

ATOM 4562 HD22 LEU 278 57.671 82.434 48.250 1.00 0.00 H

ATOM 4563 HD23 LEU 278 57.676 82.010 46.510 1.00 0.00 H

ATOM 4564 C LEU 278 61.458 79.046 48.102 1.00 0.00 C

ATOM 4565 O LEU 278 61.961 79.824 48.944 1.00 0.00 O

ATOM 4566 N ASN 279 61.105 77.775 48.313 1.00 0.00 N

ATOM 4567 H ASN 279 60.926 77.195 47.506 1.00 0.00 H

ATOM 4568 CA ASN 279 60.904 77.166 49.618 1.00 0.00 C

ATOM 4569 HA ASN 279 60.172 77.670 50.249 1.00 0.00 H

ATOM 4570 CB ASN 279 60.310 75.761 49.453 1.00 0.00 C

ATOM 4571 HB2 ASN 279 59.474 75.865 48.761 1.00 0.00 H

ATOM 4572 HB3 ASN 279 61.103 75.139 49.038 1.00 0.00 H

ATOM 4573 CG ASN 279 59.706 75.116 50.713 1.00 0.00 C

ATOM 4574 OD1 ASN 279 58.756 75.631 51.410 1.00 0.00 O

ATOM 4575 ND2 ASN 279 60.294 74.057 51.240 1.00 0.00 N

ATOM 4576 HD21 ASN 279 59.829 73.635 52.031 1.00 0.00 H

ATOM 4577 HD22 ASN 279 61.206 73.755 50.928 1.00 0.00 H

ATOM 4578 C ASN 279 62.193 77.107 50.437 1.00 0.00 C

ATOM 4579 O ASN 279 62.225 77.338 51.661 1.00 0.00 O

ATOM 4580 N SER 280 63.355 76.986 49.791 1.00 0.00 N

ATOM 4581 H SER 280 63.257 76.769 48.810 1.00 0.00 H

ATOM 4582 CA SER 280 64.785 77.045 50.293 1.00 0.00 C

ATOM 4583 HA SER 280 64.810 76.429 51.192 1.00 0.00 H

ATOM 4584 CB SER 280 65.716 76.442 49.173 1.00 0.00 C

ATOM 4585 HB2 SER 280 65.272 76.593 48.189 1.00 0.00 H

ATOM 4586 HB3 SER 280 66.650 77.001 49.202 1.00 0.00 H

ATOM 4587 OG SER 280 65.889 75.075 49.361 1.00 0.00 O

ATOM 4588 HG SER 280 66.278 74.998 50.236 1.00 0.00 H

ATOM 4589 C SER 280 65.227 78.441 50.666 1.00 0.00 C

ATOM 4590 O SER 280 66.335 78.726 51.070 1.00 0.00 O

ATOM 4591 N CYX 281 64.307 79.393 50.696 1.00 0.00 N

ATOM 4592 H CYX 281 63.413 79.200 50.268 1.00 0.00 H

ATOM 4593 CA CYX 281 64.443 80.692 51.346 1.00 0.00 C

ATOM 4594 HA CYX 281 65.431 80.839 51.782 1.00 0.00 H

ATOM 4595 CB CYX 281 64.275 81.814 50.329 1.00 0.00 C

ATOM 4596 HB2 CYX 281 63.365 81.570 49.780 1.00 0.00 H

ATOM 4597 HB3 CYX 281 64.348 82.737 50.905 1.00 0.00 H

ATOM 4598 SG CYX 281 65.492 81.929 48.919 1.00 0.00 S

ATOM 4599 C CYX 281 63.421 80.928 52.485 1.00 0.00 C

ATOM 4600 O CYX 281 63.506 81.864 53.388 1.00 0.00 O

ATOM 4601 N VAL 282 62.446 80.009 52.588 1.00 0.00 N

ATOM 4602 H VAL 282 62.580 79.141 52.089 1.00 0.00 H

ATOM 4603 CA VAL 282 61.358 80.126 53.570 1.00 0.00 C

ATOM 4604 HA VAL 282 61.295 81.101 54.052 1.00 0.00 H

ATOM 4605 CB VAL 282 59.951 79.822 52.915 1.00 0.00 C

ATOM 4606 HB VAL 282 59.899 78.989 52.214 1.00 0.00 H

ATOM 4607 CG1 VAL 282 58.801 79.723 54.001 1.00 0.00 C

ATOM 4608 HG11 VAL 282 58.827 78.754 54.499 1.00 0.00 H

ATOM 4609 HG12 VAL 282 58.986 80.420 54.819 1.00 0.00 H

ATOM 4610 HG13 VAL 282 57.814 79.859 53.559 1.00 0.00 H

ATOM 4611 CG2 VAL 282 59.687 81.068 52.031 1.00 0.00 C

ATOM 4612 HG21 VAL 282 58.642 81.157 51.732 1.00 0.00 H

ATOM 4613 HG22 VAL 282 59.884 81.973 52.605 1.00 0.00 H

ATOM 4614 HG23 VAL 282 60.259 81.103 51.104 1.00 0.00 H

ATOM 4615 C VAL 282 61.647 79.216 54.788 1.00 0.00 C

ATOM 4616 O VAL 282 61.690 79.660 55.914 1.00 0.00 O

ATOM 4617 N ASN 283 61.792 77.938 54.445 1.00 0.00 N

ATOM 4618 H ASN 283 61.676 77.612 53.496 1.00 0.00 H

ATOM 4619 CA ASN 283 62.156 76.940 55.483 1.00 0.00 C

ATOM 4620 HA ASN 283 61.295 76.827 56.141 1.00 0.00 H

ATOM 4621 CB ASN 283 62.266 75.625 54.807 1.00 0.00 C

ATOM 4622 HB2 ASN 283 61.577 75.467 53.978 1.00 0.00 H

ATOM 4623 HB3 ASN 283 63.244 75.589 54.325 1.00 0.00 H

ATOM 4624 CG ASN 283 62.167 74.507 55.801 1.00 0.00 C

ATOM 4625 OD1 ASN 283 61.417 74.467 56.749 1.00 0.00 O

ATOM 4626 ND2 ASN 283 63.066 73.528 55.550 1.00 0.00 N

ATOM 4627 HD21 ASN 283 62.828 72.586 55.826 1.00 0.00 H

ATOM 4628 HD22 ASN 283 63.633 73.659 54.725 1.00 0.00 H

ATOM 4629 C ASN 283 63.331 77.336 56.383 1.00 0.00 C

ATOM 4630 O ASN 283 63.248 77.224 57.585 1.00 0.00 O

ATOM 4631 N PRO 284 64.465 77.905 55.849 1.00 0.00 N

ATOM 4632 CD PRO 284 64.955 77.921 54.435 1.00 0.00 C

ATOM 4633 HD2 PRO 284 64.269 78.513 53.829 1.00 0.00 H

ATOM 4634 HD3 PRO 284 65.014 76.928 53.990 1.00 0.00 H

ATOM 4635 CG PRO 284 66.339 78.564 54.419 1.00 0.00 C

ATOM 4636 HG2 PRO 284 66.337 79.198 53.532 1.00 0.00 H

ATOM 4637 HG3 PRO 284 67.157 77.848 54.501 1.00 0.00 H

ATOM 4638 CB PRO 284 66.343 79.329 55.710 1.00 0.00 C

ATOM 4639 HB2 PRO 284 65.865 80.297 55.557 1.00 0.00 H

ATOM 4640 HB3 PRO 284 67.373 79.256 56.058 1.00 0.00 H

ATOM 4641 CA PRO 284 65.418 78.563 56.717 1.00 0.00 C

ATOM 4642 HA PRO 284 66.134 77.896 57.197 1.00 0.00 H

ATOM 4643 C PRO 284 64.808 79.451 57.956 1.00 0.00 C

ATOM 4644 O PRO 284 65.256 79.465 59.052 1.00 0.00 O

ATOM 4645 N ILE 285 63.808 80.218 57.616 1.00 0.00 N

ATOM 4646 H ILE 285 63.287 79.954 56.793 1.00 0.00 H

ATOM 4647 CA ILE 285 63.243 81.221 58.572 1.00 0.00 C

ATOM 4648 HA ILE 285 63.985 81.595 59.277 1.00 0.00 H

ATOM 4649 CB ILE 285 62.545 82.412 57.845 1.00 0.00 C

ATOM 4650 HB ILE 285 61.574 82.006 57.562 1.00 0.00 H

ATOM 4651 CG2 ILE 285 62.075 83.529 58.778 1.00 0.00 C

ATOM 4652 HG21 ILE 285 61.210 83.167 59.333 1.00 0.00 H

ATOM 4653 HG22 ILE 285 62.840 83.742 59.524 1.00 0.00 H

ATOM 4654 HG23 ILE 285 61.935 84.446 58.206 1.00 0.00 H

ATOM 4655 CG1 ILE 285 63.290 82.767 56.546 1.00 0.00 C

ATOM 4656 HG12 ILE 285 63.050 82.002 55.808 1.00 0.00 H

ATOM 4657 HG13 ILE 285 62.811 83.705 56.265 1.00 0.00 H

ATOM 4658 CD1 ILE 285 64.806 83.038 56.632 1.00 0.00 C

ATOM 4659 HD11 ILE 285 64.811 83.834 57.376 1.00 0.00 H

ATOM 4660 HD12 ILE 285 65.457 82.191 56.846 1.00 0.00 H

ATOM 4661 HD13 ILE 285 65.120 83.369 55.642 1.00 0.00 H

ATOM 4662 C ILE 285 62.293 80.503 59.474 1.00 0.00 C

ATOM 4663 O ILE 285 62.369 80.703 60.691 1.00 0.00 O

ATOM 4664 N LEU 286 61.600 79.518 58.891 1.00 0.00 N

ATOM 4665 H LEU 286 61.745 79.351 57.905 1.00 0.00 H

ATOM 4666 CA LEU 286 60.591 78.585 59.535 1.00 0.00 C

ATOM 4667 HA LEU 286 59.793 79.223 59.916 1.00 0.00 H

ATOM 4668 CB LEU 286 59.951 77.790 58.373 1.00 0.00 C

ATOM 4669 HB2 LEU 286 59.666 78.424 57.534 1.00 0.00 H

ATOM 4670 HB3 LEU 286 60.691 77.032 58.116 1.00 0.00 H

ATOM 4671 CG LEU 286 58.769 76.883 58.783 1.00 0.00 C

ATOM 4672 HG LEU 286 59.208 76.262 59.564 1.00 0.00 H

ATOM 4673 CD1 LEU 286 57.663 77.656 59.430 1.00 0.00 C

ATOM 4674 HD11 LEU 286 57.710 77.575 60.516 1.00 0.00 H

ATOM 4675 HD12 LEU 286 57.700 78.715 59.172 1.00 0.00 H

ATOM 4676 HD13 LEU 286 56.698 77.335 59.037 1.00 0.00 H

ATOM 4677 CD2 LEU 286 58.229 76.058 57.588 1.00 0.00 C

ATOM 4678 HD21 LEU 286 57.941 76.772 56.816 1.00 0.00 H

ATOM 4679 HD22 LEU 286 59.071 75.530 57.140 1.00 0.00 H

ATOM 4680 HD23 LEU 286 57.365 75.418 57.761 1.00 0.00 H

ATOM 4681 C LEU 286 61.109 77.792 60.719 1.00 0.00 C

ATOM 4682 O LEU 286 60.363 77.761 61.727 1.00 0.00 O

ATOM 4683 N TYR 287 62.305 77.141 60.584 1.00 0.00 N

ATOM 4684 H TYR 287 62.682 77.241 59.652 1.00 0.00 H

ATOM 4685 CA TYR 287 62.848 76.110 61.438 1.00 0.00 C

ATOM 4686 HA TYR 287 62.386 75.144 61.231 1.00 0.00 H

ATOM 4687 CB TYR 287 64.336 75.802 61.138 1.00 0.00 C

ATOM 4688 HB2 TYR 287 64.962 76.658 61.392 1.00 0.00 H

ATOM 4689 HB3 TYR 287 64.630 75.111 61.927 1.00 0.00 H

ATOM 4690 CG TYR 287 64.732 75.232 59.828 1.00 0.00 C

ATOM 4691 CD1 TYR 287 64.334 73.932 59.484 1.00 0.00 C

ATOM 4692 HD1 TYR 287 63.779 73.226 60.084 1.00 0.00 H

ATOM 4693 CE1 TYR 287 64.820 73.246 58.338 1.00 0.00 C

ATOM 4694 HE1 TYR 287 64.695 72.196 58.121 1.00 0.00 H

ATOM 4695 CZ TYR 287 65.667 73.956 57.480 1.00 0.00 C

ATOM 4696 OH TYR 287 66.110 73.431 56.347 1.00 0.00 O

ATOM 4697 HH TYR 287 66.683 74.046 55.884 1.00 0.00 H

ATOM 4698 CE2 TYR 287 66.150 75.228 57.896 1.00 0.00 C

ATOM 4699 HE2 TYR 287 67.077 75.526 57.429 1.00 0.00 H

ATOM 4700 CD2 TYR 287 65.657 75.926 59.063 1.00 0.00 C

ATOM 4701 HD2 TYR 287 66.146 76.830 59.394 1.00 0.00 H

ATOM 4702 C TYR 287 62.596 76.330 62.985 1.00 0.00 C

ATOM 4703 O TYR 287 61.889 75.565 63.566 1.00 0.00 O

ATOM 4704 N PHE 288 63.214 77.323 63.549 1.00 0.00 N

ATOM 4705 H PHE 288 64.069 77.652 63.123 1.00 0.00 H

ATOM 4706 CA PHE 288 63.170 77.412 65.056 1.00 0.00 C

ATOM 4707 HA PHE 288 63.046 76.462 65.576 1.00 0.00 H

ATOM 4708 CB PHE 288 64.431 78.007 65.563 1.00 0.00 C

ATOM 4709 HB2 PHE 288 64.510 78.971 65.059 1.00 0.00 H

ATOM 4710 HB3 PHE 288 64.493 78.147 66.642 1.00 0.00 H

ATOM 4711 CG PHE 288 65.625 77.050 65.166 1.00 0.00 C

ATOM 4712 CD1 PHE 288 65.754 75.775 65.723 1.00 0.00 C

ATOM 4713 HD1 PHE 288 65.190 75.584 66.624 1.00 0.00 H

ATOM 4714 CE1 PHE 288 66.648 74.798 65.188 1.00 0.00 C

ATOM 4715 HE1 PHE 288 66.733 73.810 65.615 1.00 0.00 H

ATOM 4716 CZ PHE 288 67.450 75.119 64.108 1.00 0.00 C

ATOM 4717 HZ PHE 288 68.262 74.439 63.894 1.00 0.00 H

ATOM 4718 CE2 PHE 288 67.283 76.295 63.526 1.00 0.00 C

ATOM 4719 HE2 PHE 288 67.712 76.502 62.557 1.00 0.00 H

ATOM 4720 CD2 PHE 288 66.472 77.327 64.098 1.00 0.00 C

ATOM 4721 HD2 PHE 288 66.398 78.190 63.453 1.00 0.00 H

ATOM 4722 C PHE 288 61.909 78.199 65.490 1.00 0.00 C

ATOM 4723 O PHE 288 61.799 78.515 66.728 1.00 0.00 O

ATOM 4724 N LEU 289 61.054 78.627 64.582 1.00 0.00 N

ATOM 4725 H LEU 289 61.440 78.501 63.657 1.00 0.00 H

ATOM 4726 CA LEU 289 60.243 79.758 64.748 1.00 0.00 C

ATOM 4727 HA LEU 289 60.291 80.179 65.753 1.00 0.00 H

ATOM 4728 CB LEU 289 60.759 80.786 63.661 1.00 0.00 C

ATOM 4729 HB2 LEU 289 61.821 80.983 63.811 1.00 0.00 H

ATOM 4730 HB3 LEU 289 60.682 80.274 62.702 1.00 0.00 H

ATOM 4731 CG LEU 289 59.890 82.037 63.513 1.00 0.00 C

ATOM 4732 HG LEU 289 58.858 81.691 63.570 1.00 0.00 H

ATOM 4733 CD1 LEU 289 59.960 82.987 64.731 1.00 0.00 C

ATOM 4734 HD11 LEU 289 59.421 82.497 65.542 1.00 0.00 H

ATOM 4735 HD12 LEU 289 60.981 83.163 65.070 1.00 0.00 H

ATOM 4736 HD13 LEU 289 59.319 83.859 64.599 1.00 0.00 H

ATOM 4737 CD2 LEU 289 60.213 82.801 62.233 1.00 0.00 C

ATOM 4738 HD21 LEU 289 61.270 82.943 62.009 1.00 0.00 H

ATOM 4739 HD22 LEU 289 59.747 82.337 61.363 1.00 0.00 H

ATOM 4740 HD23 LEU 289 59.765 83.793 62.288 1.00 0.00 H

ATOM 4741 C LEU 289 58.760 79.382 64.713 1.00 0.00 C

ATOM 4742 O LEU 289 58.053 79.739 65.660 1.00 0.00 O

ATOM 4743 N ALA 290 58.322 78.602 63.737 1.00 0.00 N

ATOM 4744 H ALA 290 58.925 78.332 62.973 1.00 0.00 H

ATOM 4745 CA ALA 290 57.152 77.758 63.957 1.00 0.00 C

ATOM 4746 HA ALA 290 57.211 77.463 65.005 1.00 0.00 H

ATOM 4747 CB ALA 290 55.832 78.534 63.679 1.00 0.00 C

ATOM 4748 HB1 ALA 290 55.597 79.207 64.503 1.00 0.00 H

ATOM 4749 HB2 ALA 290 55.904 78.997 62.695 1.00 0.00 H

ATOM 4750 HB3 ALA 290 55.032 77.808 63.826 1.00 0.00 H

ATOM 4751 C ALA 290 57.194 76.475 63.089 1.00 0.00 C

ATOM 4752 O ALA 290 56.282 75.723 63.008 1.00 0.00 O

ATOM 4753 N GLY 291 58.381 76.035 62.660 1.00 0.00 N

ATOM 4754 H GLY 291 59.159 76.516 63.089 1.00 0.00 H

ATOM 4755 CA GLY 291 58.678 74.604 62.430 1.00 0.00 C

ATOM 4756 HA2 GLY 291 57.921 74.147 61.792 1.00 0.00 H

ATOM 4757 HA3 GLY 291 59.650 74.509 61.947 1.00 0.00 H

ATOM 4758 C GLY 291 58.623 73.835 63.753 1.00 0.00 C

ATOM 4759 O GLY 291 57.702 73.103 64.042 1.00 0.00 O

ATOM 4760 N ASP 292 59.662 74.034 64.583 1.00 0.00 N

ATOM 4761 H ASP 292 60.357 74.662 64.205 1.00 0.00 H

ATOM 4762 CA ASP 292 59.759 73.737 66.002 1.00 0.00 C

ATOM 4763 HA ASP 292 58.829 73.348 66.418 1.00 0.00 H

ATOM 4764 CB ASP 292 60.821 72.643 66.056 1.00 0.00 C

ATOM 4765 HB2 ASP 292 60.556 71.943 65.263 1.00 0.00 H

ATOM 4766 HB3 ASP 292 61.798 73.057 65.807 1.00 0.00 H

ATOM 4767 CG ASP 292 60.868 71.831 67.314 1.00 0.00 C

ATOM 4768 OD1 ASP 292 61.553 72.226 68.252 1.00 0.00 O

ATOM 4769 OD2 ASP 292 59.869 71.122 67.594 1.00 0.00 O

ATOM 4770 C ASP 292 60.199 75.062 66.732 1.00 0.00 C

ATOM 4771 O ASP 292 59.488 76.064 66.742 1.00 0.00 O

ATOM 4772 N THR 293 61.260 75.011 67.548 1.00 0.00 N

ATOM 4773 H THR 293 61.700 74.104 67.613 1.00 0.00 H

ATOM 4774 CA THR 293 61.273 76.041 68.660 1.00 0.00 C

ATOM 4775 HA THR 293 60.797 76.994 68.431 1.00 0.00 H

ATOM 4776 CB THR 293 60.628 75.343 69.995 1.00 0.00 C

ATOM 4777 HB THR 293 61.014 74.328 70.086 1.00 0.00 H

ATOM 4778 CG2 THR 293 60.882 76.063 71.375 1.00 0.00 C

ATOM 4779 HG21 THR 293 60.712 77.135 71.274 1.00 0.00 H

ATOM 4780 HG22 THR 293 60.228 75.610 72.120 1.00 0.00 H

ATOM 4781 HG23 THR 293 61.912 75.790 71.603 1.00 0.00 H

ATOM 4782 OG1 THR 293 59.170 75.296 69.865 1.00 0.00 O

ATOM 4783 HG1 THR 293 58.913 74.432 69.534 1.00 0.00 H

ATOM 4784 C THR 293 62.716 76.446 69.086 1.00 0.00 C

ATOM 4785 O THR 293 63.462 75.459 69.167 1.00 0.00 O

ATOM 4786 N PHE 294 63.028 77.648 69.546 1.00 0.00 N

ATOM 4787 H PHE 294 62.349 78.392 69.474 1.00 0.00 H

ATOM 4788 CA PHE 294 64.176 78.014 70.360 1.00 0.00 C

ATOM 4789 HA PHE 294 64.879 77.201 70.541 1.00 0.00 H

ATOM 4790 CB PHE 294 64.963 79.173 69.651 1.00 0.00 C

ATOM 4791 HB2 PHE 294 65.324 78.798 68.693 1.00 0.00 H

ATOM 4792 HB3 PHE 294 64.315 80.028 69.457 1.00 0.00 H

ATOM 4793 CG PHE 294 66.242 79.580 70.410 1.00 0.00 C

ATOM 4794 CD1 PHE 294 66.224 80.790 71.083 1.00 0.00 C

ATOM 4795 HD1 PHE 294 65.263 81.282 71.110 1.00 0.00 H

ATOM 4796 CE1 PHE 294 67.313 81.256 71.793 1.00 0.00 C

ATOM 4797 HE1 PHE 294 67.284 82.101 72.466 1.00 0.00 H

ATOM 4798 CZ PHE 294 68.461 80.396 71.908 1.00 0.00 C

ATOM 4799 HZ PHE 294 69.214 80.624 72.647 1.00 0.00 H

ATOM 4800 CE2 PHE 294 68.463 79.129 71.319 1.00 0.00 C

ATOM 4801 HE2 PHE 294 69.293 78.448 71.441 1.00 0.00 H

ATOM 4802 CD2 PHE 294 67.443 78.762 70.398 1.00 0.00 C

ATOM 4803 HD2 PHE 294 67.560 77.964 69.680 1.00 0.00 H

ATOM 4804 C PHE 294 63.767 78.482 71.757 1.00 0.00 C

ATOM 4805 O PHE 294 63.044 79.502 71.881 1.00 0.00 O

ATOM 4806 N ARG 295 64.160 77.795 72.848 1.00 0.00 N

ATOM 4807 H ARG 295 64.664 76.927 72.743 1.00 0.00 H

ATOM 4808 CA ARG 295 64.075 78.235 74.244 1.00 0.00 C

ATOM 4809 HA ARG 295 64.252 77.334 74.831 1.00 0.00 H

ATOM 4810 CB ARG 295 65.097 79.312 74.557 1.00 0.00 C

ATOM 4811 HB2 ARG 295 65.568 79.789 73.698 1.00 0.00 H

ATOM 4812 HB3 ARG 295 64.665 80.105 75.167 1.00 0.00 H

ATOM 4813 CG ARG 295 66.278 78.768 75.437 1.00 0.00 C

ATOM 4814 HG2 ARG 295 65.833 78.415 76.368 1.00 0.00 H

ATOM 4815 HG3 ARG 295 66.740 77.930 74.915 1.00 0.00 H

ATOM 4816 CD ARG 295 67.334 79.832 75.910 1.00 0.00 C

ATOM 4817 HD2 ARG 295 68.199 79.790 75.249 1.00 0.00 H

ATOM 4818 HD3 ARG 295 66.768 80.749 75.753 1.00 0.00 H

ATOM 4819 NE ARG 295 67.485 79.625 77.386 1.00 0.00 N

ATOM 4820 HE ARG 295 66.915 79.020 77.960 1.00 0.00 H

ATOM 4821 CZ ARG 295 68.368 80.209 78.095 1.00 0.00 C

ATOM 4822 NH1 ARG 295 68.227 80.127 79.384 1.00 0.00 N

ATOM 4823 HH11 ARG 295 67.348 79.917 79.835 1.00 0.00 H

ATOM 4824 HH12 ARG 295 69.064 80.387 79.886 1.00 0.00 H

ATOM 4825 NH2 ARG 295 69.169 81.068 77.567 1.00 0.00 N

ATOM 4826 HH21 ARG 295 68.935 81.304 76.613 1.00 0.00 H

ATOM 4827 HH22 ARG 295 69.556 81.757 78.196 1.00 0.00 H

ATOM 4828 C ARG 295 62.653 78.656 74.648 1.00 0.00 C

ATOM 4829 O ARG 295 62.191 79.769 74.357 1.00 0.00 O

ATOM 4830 N ARG 296 61.843 77.778 75.220 1.00 0.00 N

ATOM 4831 H ARG 296 62.252 76.868 75.377 1.00 0.00 H

ATOM 4832 CA ARG 296 60.394 77.889 75.326 1.00 0.00 C

ATOM 4833 HA ARG 296 59.913 77.891 74.347 1.00 0.00 H

ATOM 4834 CB ARG 296 60.057 76.572 76.030 1.00 0.00 C

ATOM 4835 HB2 ARG 296 60.761 76.361 76.835 1.00 0.00 H

ATOM 4836 HB3 ARG 296 59.070 76.703 76.475 1.00 0.00 H

ATOM 4837 CG ARG 296 59.673 75.397 75.130 1.00 0.00 C

ATOM 4838 HG2 ARG 296 58.753 75.426 74.546 1.00 0.00 H

ATOM 4839 HG3 ARG 296 60.511 75.288 74.442 1.00 0.00 H

ATOM 4840 CD ARG 296 59.469 74.114 75.953 1.00 0.00 C

ATOM 4841 HD2 ARG 296 59.154 73.371 75.221 1.00 0.00 H

ATOM 4842 HD3 ARG 296 60.413 73.788 76.391 1.00 0.00 H

ATOM 4843 NE ARG 296 58.360 74.250 76.957 1.00 0.00 N

ATOM 4844 HE ARG 296 58.537 74.333 77.948 1.00 0.00 H

ATOM 4845 CZ ARG 296 57.073 74.328 76.759 1.00 0.00 C

ATOM 4846 NH1 ARG 296 56.370 74.849 77.674 1.00 0.00 N

ATOM 4847 HH11 ARG 296 55.433 74.485 77.769 1.00 0.00 H

ATOM 4848 HH12 ARG 296 56.794 75.231 78.508 1.00 0.00 H

ATOM 4849 NH2 ARG 296 56.443 73.841 75.731 1.00 0.00 N

ATOM 4850 HH21 ARG 296 55.522 74.206 75.532 1.00 0.00 H

ATOM 4851 HH22 ARG 296 56.806 73.022 75.265 1.00 0.00 H

ATOM 4852 C ARG 296 59.736 79.093 76.078 1.00 0.00 C

ATOM 4853 O ARG 296 59.766 79.222 77.328 1.00 0.00 O

ATOM 4854 N ARG 297 59.155 80.015 75.301 1.00 0.00 N

ATOM 4855 H ARG 297 59.078 79.774 74.323 1.00 0.00 H

ATOM 4856 CA ARG 297 58.652 81.384 75.745 1.00 0.00 C

ATOM 4857 HA ARG 297 58.627 81.637 76.806 1.00 0.00 H

ATOM 4858 CB ARG 297 59.410 82.447 74.962 1.00 0.00 C

ATOM 4859 HB2 ARG 297 59.629 81.924 74.031 1.00 0.00 H

ATOM 4860 HB3 ARG 297 58.810 83.352 74.871 1.00 0.00 H

ATOM 4861 CG ARG 297 60.697 82.737 75.720 1.00 0.00 C

ATOM 4862 HG2 ARG 297 60.511 83.263 76.656 1.00 0.00 H

ATOM 4863 HG3 ARG 297 61.171 81.853 76.146 1.00 0.00 H

ATOM 4864 CD ARG 297 61.653 83.562 74.687 1.00 0.00 C

ATOM 4865 HD2 ARG 297 61.086 84.231 74.040 1.00 0.00 H

ATOM 4866 HD3 ARG 297 62.319 84.137 75.329 1.00 0.00 H

ATOM 4867 NE ARG 297 62.542 82.747 73.852 1.00 0.00 N

ATOM 4868 HE ARG 297 62.210 81.795 73.787 1.00 0.00 H

ATOM 4869 CZ ARG 297 62.985 83.205 72.715 1.00 0.00 C

ATOM 4870 NH1 ARG 297 63.320 84.425 72.552 1.00 0.00 N

ATOM 4871 HH11 ARG 297 63.701 84.766 71.680 1.00 0.00 H

ATOM 4872 HH12 ARG 297 63.725 84.891 73.351 1.00 0.00 H

ATOM 4873 NH2 ARG 297 63.158 82.426 71.692 1.00 0.00 N

ATOM 4874 HH21 ARG 297 63.077 81.420 71.715 1.00 0.00 H

ATOM 4875 HH22 ARG 297 63.607 82.805 70.870 1.00 0.00 H

ATOM 4876 C ARG 297 57.215 81.381 75.172 1.00 0.00 C

ATOM 4877 O ARG 297 56.253 81.094 75.913 1.00 0.00 O

ATOM 4878 OXT ARG 297 56.957 81.452 73.967 1.00 0.00 O

TER

ATOM 4879 O2B 6AD 298 62.302 71.076 31.631 1.00 0.00 O

ATOM 4880 PB 6AD 298 62.744 69.752 32.107 1.00 0.00 P

ATOM 4881 O1B 6AD 298 64.183 69.525 31.858 1.00 0.00 O

ATOM 4882 O3B 6AD 298 62.436 69.619 33.513 1.00 0.00 O

ATOM 4883 O3A 6AD 298 61.815 69.137 31.120 1.00 0.00 O

ATOM 4884 PA 6AD 298 62.007 67.664 30.726 1.00 0.00 P

ATOM 4885 O1A 6AD 298 63.376 67.328 30.294 1.00 0.00 O

ATOM 4886 O2A 6AD 298 60.951 67.302 29.765 1.00 0.00 O

ATOM 4887 O5' 6AD 298 61.647 66.913 32.118 1.00 0.00 O

ATOM 4888 C5' 6AD 298 61.335 65.486 32.164 1.00 0.00 C

ATOM 4889 C4' 6AD 298 61.496 65.111 33.612 1.00 0.00 C

ATOM 4890 O4' 6AD 298 60.238 65.311 34.325 1.00 0.00 O

ATOM 4891 C3' 6AD 298 61.650 63.590 33.803 1.00 0.00 C

ATOM 4892 O3' 6AD 298 62.996 63.177 33.740 1.00 0.00 O

ATOM 4893 C2' 6AD 298 60.857 63.107 35.109 1.00 0.00 C

ATOM 4894 O2' 6AD 298 61.768 62.680 36.069 1.00 0.00 O

ATOM 4895 C1' 6AD 298 60.186 64.461 35.564 1.00 0.00 C

ATOM 4896 N9 6AD 298 58.862 64.405 36.139 1.00 0.00 N

ATOM 4897 C8 6AD 298 57.688 65.024 35.715 1.00 0.00 C

ATOM 4898 N7 6AD 298 56.694 64.949 36.635 1.00 0.00 N

ATOM 4899 C5 6AD 298 57.240 64.141 37.524 1.00 0.00 C

ATOM 4900 C4 6AD 298 58.509 63.695 37.184 1.00 0.00 C

ATOM 4901 N3 6AD 298 59.271 62.915 37.950 1.00 0.00 N

ATOM 4902 C2 6AD 298 58.741 62.510 39.162 1.00 0.00 C

ATOM 4903 S1 6AD 298 59.611 61.256 40.071 1.00 0.00 S

ATOM 4904 C6 6AD 298 59.455 59.815 39.021 1.00 0.00 C

ATOM 4905 N1 6AD 298 57.568 62.986 39.637 1.00 0.00 N

ATOM 4906 C7 6AD 298 56.869 63.844 38.810 1.00 0.00 C

ATOM 4907 N6 6AD 298 55.640 64.098 39.233 1.00 0.00 N

ATOM 4908 H30 6AD 298 60.394 65.219 31.676 1.00 0.00 H

ATOM 4909 H31 6AD 298 62.157 65.069 31.577 1.00 0.00 H

ATOM 4910 H32 6AD 298 62.286 65.671 34.120 1.00 0.00 H

ATOM 4911 H33 6AD 298 61.116 63.135 32.965 1.00 0.00 H

ATOM 4912 H34 6AD 298 63.048 62.771 34.624 1.00 0.00 H

ATOM 4913 H35 6AD 298 60.117 62.325 34.918 1.00 0.00 H

ATOM 4914 H36 6AD 298 61.390 62.740 36.965 1.00 0.00 H

ATOM 4915 H37 6AD 298 60.674 64.904 36.445 1.00 0.00 H

ATOM 4916 H38 6AD 298 57.580 65.469 34.738 1.00 0.00 H

ATOM 4917 H39 6AD 298 60.405 59.643 38.509 1.00 0.00 H

ATOM 4918 H40 6AD 298 59.313 58.876 39.563 1.00 0.00 H

ATOM 4919 H41 6AD 298 58.658 59.999 38.296 1.00 0.00 H

ATOM 4920 H42 6AD 298 55.176 64.720 38.582 1.00 0.00 H

ATOM 4921 H43 6AD 298 55.045 63.346 39.563 1.00 0.00 H

END
