# Supplementary material for: Identification of a Different Agonist-Binding Site and Activation Mechanism of the Human P2Y1 Receptor
Source: Sci Rep. 2017 Oct 23;7:13764. doi: 10.1038/s41598-017-14268-1 (PMC5653743; doi:10.1038/s41598-017-14268-1)
Supplement: Supplementary file 3 — pdb file of the intermediate state [file 41598_2017_14268_MOESM3_ESM.doc]

# Supplementary Information

Identification of a Different Agonist-Binding Site and Activation Mechanism of the Human P2Y_1_ Receptor

Yang Li, Can Yin, Pi Liu, Dongmei Li* and Jianping Lin*

- Coordinates of the intermediate state of the 2MeSADP-P2Y_12_R system (pdb file).

INTERMEDIATE STATE

ATOM 1 N SER 1 66.260 9.540 12.489 1.00 0.00 N

ATOM 2 H1 SER 1 65.955 9.928 13.370 1.00 0.00 H

ATOM 3 H2 SER 1 66.972 8.827 12.556 1.00 0.00 H

ATOM 4 H3 SER 1 65.393 9.152 12.147 1.00 0.00 H

ATOM 5 CA SER 1 66.780 10.486 11.520 1.00 0.00 C

ATOM 6 HA SER 1 66.555 10.015 10.563 1.00 0.00 H

ATOM 7 CB SER 1 66.174 11.885 11.520 1.00 0.00 C

ATOM 8 HB2 SER 1 65.087 11.802 11.527 1.00 0.00 H

ATOM 9 HB3 SER 1 66.528 12.324 12.453 1.00 0.00 H

ATOM 10 OG SER 1 66.642 12.572 10.400 1.00 0.00 O

ATOM 11 HG SER 1 67.102 13.307 10.813 1.00 0.00 H

ATOM 12 C SER 1 68.331 10.470 11.419 1.00 0.00 C

ATOM 13 O SER 1 68.934 11.148 12.246 1.00 0.00 O

ATOM 14 N SER 2 68.907 9.608 10.567 1.00 0.00 N

ATOM 15 H SER 2 68.354 8.978 10.004 1.00 0.00 H

ATOM 16 CA SER 2 70.266 9.706 10.164 1.00 0.00 C

ATOM 17 HA SER 2 70.965 9.744 11.000 1.00 0.00 H

ATOM 18 CB SER 2 70.708 8.503 9.423 1.00 0.00 C

ATOM 19 HB2 SER 2 71.706 8.724 9.043 1.00 0.00 H

ATOM 20 HB3 SER 2 70.747 7.615 10.055 1.00 0.00 H

ATOM 21 OG SER 2 69.779 8.322 8.304 1.00 0.00 O

ATOM 22 HG SER 2 70.267 8.069 7.517 1.00 0.00 H

ATOM 23 C SER 2 70.606 11.068 9.414 1.00 0.00 C

ATOM 24 O SER 2 71.571 11.678 9.858 1.00 0.00 O

ATOM 25 N PHE 3 69.771 11.626 8.448 1.00 0.00 N

ATOM 26 H PHE 3 68.890 11.152 8.316 1.00 0.00 H

ATOM 27 CA PHE 3 70.115 12.881 7.830 1.00 0.00 C

ATOM 28 HA PHE 3 70.524 13.580 8.559 1.00 0.00 H

ATOM 29 CB PHE 3 71.287 12.748 6.726 1.00 0.00 C

ATOM 30 HB2 PHE 3 71.611 13.766 6.513 1.00 0.00 H

ATOM 31 HB3 PHE 3 72.132 12.222 7.171 1.00 0.00 H

ATOM 32 CG PHE 3 70.842 11.971 5.464 1.00 0.00 C

ATOM 33 CD1 PHE 3 70.599 12.585 4.230 1.00 0.00 C

ATOM 34 HD1 PHE 3 70.699 13.649 4.076 1.00 0.00 H

ATOM 35 CE1 PHE 3 70.213 11.754 3.175 1.00 0.00 C

ATOM 36 HE1 PHE 3 69.949 12.256 2.256 1.00 0.00 H

ATOM 37 CZ PHE 3 69.911 10.360 3.290 1.00 0.00 C

ATOM 38 HZ PHE 3 69.720 9.777 2.401 1.00 0.00 H

ATOM 39 CE2 PHE 3 70.262 9.758 4.502 1.00 0.00 C

ATOM 40 HE2 PHE 3 70.064 8.702 4.609 1.00 0.00 H

ATOM 41 CD2 PHE 3 70.726 10.534 5.552 1.00 0.00 C

ATOM 42 HD2 PHE 3 71.103 10.068 6.450 1.00 0.00 H

ATOM 43 C PHE 3 68.879 13.662 7.310 1.00 0.00 C

ATOM 44 O PHE 3 68.855 14.814 7.554 1.00 0.00 O

ATOM 45 N LYS 4 67.900 12.966 6.640 1.00 0.00 N

ATOM 46 H LYS 4 67.948 11.958 6.604 1.00 0.00 H

ATOM 47 CA LYS 4 66.671 13.529 6.012 1.00 0.00 C

ATOM 48 HA LYS 4 66.945 14.014 5.075 1.00 0.00 H

ATOM 49 CB LYS 4 65.804 12.282 5.662 1.00 0.00 C

ATOM 50 HB2 LYS 4 65.626 11.839 6.642 1.00 0.00 H

ATOM 51 HB3 LYS 4 64.843 12.651 5.304 1.00 0.00 H

ATOM 52 CG LYS 4 66.387 11.326 4.645 1.00 0.00 C

ATOM 53 HG2 LYS 4 66.795 12.021 3.912 1.00 0.00 H

ATOM 54 HG3 LYS 4 67.298 10.860 5.022 1.00 0.00 H

ATOM 55 CD LYS 4 65.262 10.376 4.280 1.00 0.00 C

ATOM 56 HD2 LYS 4 65.243 9.559 5.001 1.00 0.00 H

ATOM 57 HD3 LYS 4 64.268 10.818 4.200 1.00 0.00 H

ATOM 58 CE LYS 4 65.774 10.079 2.922 1.00 0.00 C

ATOM 59 HE2 LYS 4 65.789 10.954 2.272 1.00 0.00 H

ATOM 60 HE3 LYS 4 66.828 9.866 3.100 1.00 0.00 H

ATOM 61 NZ LYS 4 64.979 9.020 2.306 1.00 0.00 N

ATOM 62 HZ1 LYS 4 64.009 9.130 2.566 1.00 0.00 H

ATOM 63 HZ2 LYS 4 65.044 9.096 1.301 1.00 0.00 H

ATOM 64 HZ3 LYS 4 65.198 8.112 2.689 1.00 0.00 H

ATOM 65 C LYS 4 65.961 14.693 6.826 1.00 0.00 C

ATOM 66 O LYS 4 65.458 15.610 6.271 1.00 0.00 O

ATOM 67 N CYX 5 65.921 14.524 8.093 1.00 0.00 N

ATOM 68 H CYX 5 65.868 13.594 8.483 1.00 0.00 H

ATOM 69 CA CYX 5 65.883 15.593 9.160 1.00 0.00 C

ATOM 70 HA CYX 5 65.597 16.507 8.641 1.00 0.00 H

ATOM 71 CB CYX 5 64.686 15.287 10.063 1.00 0.00 C

ATOM 72 HB2 CYX 5 63.849 15.448 9.383 1.00 0.00 H

ATOM 73 HB3 CYX 5 64.811 14.241 10.343 1.00 0.00 H

ATOM 74 SG CYX 5 64.458 16.337 11.446 1.00 0.00 S

ATOM 75 C CYX 5 67.230 15.983 9.822 1.00 0.00 C

ATOM 76 O CYX 5 67.844 15.110 10.480 1.00 0.00 O

ATOM 77 N ALA 6 67.735 17.190 9.596 1.00 0.00 N

ATOM 78 H ALA 6 67.160 17.688 8.932 1.00 0.00 H

ATOM 79 CA ALA 6 68.865 17.832 10.305 1.00 0.00 C

ATOM 80 HA ALA 6 69.591 17.084 10.621 1.00 0.00 H

ATOM 81 CB ALA 6 69.672 18.696 9.322 1.00 0.00 C

ATOM 82 HB1 ALA 6 68.947 19.372 8.869 1.00 0.00 H

ATOM 83 HB2 ALA 6 70.526 19.203 9.770 1.00 0.00 H

ATOM 84 HB3 ALA 6 70.031 17.992 8.571 1.00 0.00 H

ATOM 85 C ALA 6 68.431 18.660 11.580 1.00 0.00 C

ATOM 86 O ALA 6 67.697 19.645 11.433 1.00 0.00 O

ATOM 87 N LEU 7 69.039 18.433 12.723 1.00 0.00 N

ATOM 88 H LEU 7 69.878 17.873 12.756 1.00 0.00 H

ATOM 89 CA LEU 7 69.058 19.425 13.846 1.00 0.00 C

ATOM 90 HA LEU 7 68.314 20.219 13.781 1.00 0.00 H

ATOM 91 CB LEU 7 68.654 18.711 15.136 1.00 0.00 C

ATOM 92 HB2 LEU 7 68.782 19.405 15.968 1.00 0.00 H

ATOM 93 HB3 LEU 7 67.571 18.593 15.149 1.00 0.00 H

ATOM 94 CG LEU 7 69.359 17.388 15.374 1.00 0.00 C

ATOM 95 HG LEU 7 69.191 16.692 14.553 1.00 0.00 H

ATOM 96 CD1 LEU 7 70.943 17.458 15.400 1.00 0.00 C

ATOM 97 HD11 LEU 7 71.218 18.450 15.757 1.00 0.00 H

ATOM 98 HD12 LEU 7 71.374 16.652 15.994 1.00 0.00 H

ATOM 99 HD13 LEU 7 71.390 17.338 14.414 1.00 0.00 H

ATOM 100 CD2 LEU 7 68.762 16.760 16.662 1.00 0.00 C

ATOM 101 HD21 LEU 7 67.729 17.078 16.803 1.00 0.00 H

ATOM 102 HD22 LEU 7 68.851 15.698 16.436 1.00 0.00 H

ATOM 103 HD23 LEU 7 69.352 17.078 17.522 1.00 0.00 H

ATOM 104 C LEU 7 70.381 20.245 13.955 1.00 0.00 C

ATOM 105 O LEU 7 71.241 19.821 13.188 1.00 0.00 O

ATOM 106 N THR 8 70.496 21.291 14.774 1.00 0.00 N

ATOM 107 H THR 8 69.626 21.439 15.265 1.00 0.00 H

ATOM 108 CA THR 8 71.598 22.291 14.797 1.00 0.00 C

ATOM 109 HA THR 8 71.791 22.662 13.790 1.00 0.00 H

ATOM 110 CB THR 8 71.294 23.394 15.783 1.00 0.00 C

ATOM 111 HB THR 8 71.175 23.007 16.795 1.00 0.00 H

ATOM 112 CG2 THR 8 72.384 24.466 15.940 1.00 0.00 C

ATOM 113 HG21 THR 8 71.892 25.231 16.541 1.00 0.00 H

ATOM 114 HG22 THR 8 73.251 24.151 16.521 1.00 0.00 H

ATOM 115 HG23 THR 8 72.705 24.942 15.014 1.00 0.00 H

ATOM 116 OG1 THR 8 70.139 24.080 15.316 1.00 0.00 O

ATOM 117 HG1 THR 8 69.370 23.540 15.510 1.00 0.00 H

ATOM 118 C THR 8 72.882 21.575 15.227 1.00 0.00 C

ATOM 119 O THR 8 72.881 21.162 16.389 1.00 0.00 O

ATOM 120 N LYS 9 73.895 21.321 14.422 1.00 0.00 N

ATOM 121 H LYS 9 73.732 21.595 13.464 1.00 0.00 H

ATOM 122 CA LYS 9 75.170 20.853 14.855 1.00 0.00 C

ATOM 123 HA LYS 9 75.120 20.062 15.603 1.00 0.00 H

ATOM 124 CB LYS 9 76.029 20.290 13.694 1.00 0.00 C

ATOM 125 HB2 LYS 9 75.507 19.370 13.432 1.00 0.00 H

ATOM 126 HB3 LYS 9 76.066 20.848 12.759 1.00 0.00 H

ATOM 127 CG LYS 9 77.487 19.970 14.079 1.00 0.00 C

ATOM 128 HG2 LYS 9 77.991 20.879 14.407 1.00 0.00 H

ATOM 129 HG3 LYS 9 77.472 19.222 14.872 1.00 0.00 H

ATOM 130 CD LYS 9 78.177 19.375 12.860 1.00 0.00 C

ATOM 131 HD2 LYS 9 79.197 19.142 13.168 1.00 0.00 H

ATOM 132 HD3 LYS 9 77.668 18.469 12.533 1.00 0.00 H

ATOM 133 CE LYS 9 78.154 20.176 11.581 1.00 0.00 C

ATOM 134 HE2 LYS 9 77.214 20.070 11.039 1.00 0.00 H

ATOM 135 HE3 LYS 9 78.225 21.230 11.851 1.00 0.00 H

ATOM 136 NZ LYS 9 79.378 19.779 10.837 1.00 0.00 N

ATOM 137 HZ1 LYS 9 80.226 19.993 11.342 1.00 0.00 H

ATOM 138 HZ2 LYS 9 79.357 18.802 10.579 1.00 0.00 H

ATOM 139 HZ3 LYS 9 79.522 20.307 9.988 1.00 0.00 H

ATOM 140 C LYS 9 75.933 21.858 15.680 1.00 0.00 C

ATOM 141 O LYS 9 76.750 21.351 16.462 1.00 0.00 O

ATOM 142 N THR 10 75.874 23.205 15.486 1.00 0.00 N

ATOM 143 H THR 10 75.269 23.557 14.757 1.00 0.00 H

ATOM 144 CA THR 10 76.744 24.172 16.196 1.00 0.00 C

ATOM 145 HA THR 10 77.355 23.714 16.974 1.00 0.00 H

ATOM 146 CB THR 10 77.949 24.775 15.486 1.00 0.00 C

ATOM 147 HB THR 10 78.343 25.617 16.054 1.00 0.00 H

ATOM 148 CG2 THR 10 79.057 23.815 15.173 1.00 0.00 C

ATOM 149 HG21 THR 10 79.887 24.308 14.667 1.00 0.00 H

ATOM 150 HG22 THR 10 79.393 23.419 16.131 1.00 0.00 H

ATOM 151 HG23 THR 10 78.793 22.947 14.570 1.00 0.00 H

ATOM 152 OG1 THR 10 77.443 25.208 14.234 1.00 0.00 O

ATOM 153 HG1 THR 10 77.462 26.168 14.217 1.00 0.00 H

ATOM 154 C THR 10 76.054 25.271 17.025 1.00 0.00 C

ATOM 155 O THR 10 76.394 25.354 18.197 1.00 0.00 O

ATOM 156 N GLY 11 75.204 26.188 16.527 1.00 0.00 N

ATOM 157 H GLY 11 75.076 25.950 15.554 1.00 0.00 H

ATOM 158 CA GLY 11 75.110 27.590 16.905 1.00 0.00 C

ATOM 159 HA2 GLY 11 76.139 27.934 16.801 1.00 0.00 H

ATOM 160 HA3 GLY 11 74.536 28.247 16.252 1.00 0.00 H

ATOM 161 C GLY 11 74.708 27.780 18.384 1.00 0.00 C

ATOM 162 O GLY 11 75.013 28.762 19.015 1.00 0.00 O

ATOM 163 N PHE 12 73.880 26.796 18.824 1.00 0.00 N

ATOM 164 H PHE 12 73.693 26.059 18.159 1.00 0.00 H

ATOM 165 CA PHE 12 73.299 26.693 20.167 1.00 0.00 C

ATOM 166 HA PHE 12 73.048 27.709 20.472 1.00 0.00 H

ATOM 167 CB PHE 12 71.977 25.944 20.089 1.00 0.00 C

ATOM 168 HB2 PHE 12 71.619 25.754 19.077 1.00 0.00 H

ATOM 169 HB3 PHE 12 72.101 25.000 20.621 1.00 0.00 H

ATOM 170 CG PHE 12 70.850 26.738 20.689 1.00 0.00 C

ATOM 171 CD1 PHE 12 70.252 26.316 21.953 1.00 0.00 C

ATOM 172 HD1 PHE 12 70.716 25.560 22.570 1.00 0.00 H

ATOM 173 CE1 PHE 12 69.226 27.043 22.580 1.00 0.00 C

ATOM 174 HE1 PHE 12 68.728 26.667 23.462 1.00 0.00 H

ATOM 175 CZ PHE 12 68.771 28.231 22.027 1.00 0.00 C

ATOM 176 HZ PHE 12 68.115 28.916 22.544 1.00 0.00 H

ATOM 177 CE2 PHE 12 69.189 28.574 20.721 1.00 0.00 C

ATOM 178 HE2 PHE 12 68.881 29.518 20.296 1.00 0.00 H

ATOM 179 CD2 PHE 12 70.331 27.907 20.213 1.00 0.00 C

ATOM 180 HD2 PHE 12 70.809 28.329 19.341 1.00 0.00 H

ATOM 181 C PHE 12 74.348 26.180 21.208 1.00 0.00 C

ATOM 182 O PHE 12 74.726 26.885 22.123 1.00 0.00 O

ATOM 183 N GLN 13 75.004 25.109 20.809 1.00 0.00 N

ATOM 184 H GLN 13 74.970 24.849 19.834 1.00 0.00 H

ATOM 185 CA GLN 13 75.978 24.420 21.609 1.00 0.00 C

ATOM 186 HA GLN 13 75.551 24.411 22.612 1.00 0.00 H

ATOM 187 CB GLN 13 75.914 22.888 21.275 1.00 0.00 C

ATOM 188 HB2 GLN 13 76.438 22.265 21.999 1.00 0.00 H

ATOM 189 HB3 GLN 13 74.869 22.665 21.489 1.00 0.00 H

ATOM 190 CG GLN 13 76.181 22.322 19.832 1.00 0.00 C

ATOM 191 HG2 GLN 13 77.003 22.956 19.501 1.00 0.00 H

ATOM 192 HG3 GLN 13 76.535 21.293 19.889 1.00 0.00 H

ATOM 193 CD GLN 13 74.977 22.255 18.892 1.00 0.00 C

ATOM 194 OE1 GLN 13 74.434 23.221 18.395 1.00 0.00 O

ATOM 195 NE2 GLN 13 74.505 21.020 18.751 1.00 0.00 N

ATOM 196 HE21 GLN 13 73.816 20.977 18.014 1.00 0.00 H

ATOM 197 HE22 GLN 13 75.100 20.211 18.859 1.00 0.00 H

ATOM 198 C GLN 13 77.383 24.926 21.758 1.00 0.00 C

ATOM 199 O GLN 13 77.895 24.926 22.859 1.00 0.00 O

ATOM 200 N PHE 14 77.899 25.557 20.679 1.00 0.00 N

ATOM 201 H PHE 14 77.276 25.701 19.897 1.00 0.00 H

ATOM 202 CA PHE 14 79.380 25.557 20.392 1.00 0.00 C

ATOM 203 HA PHE 14 79.809 24.664 20.848 1.00 0.00 H

ATOM 204 CB PHE 14 79.640 25.649 18.900 1.00 0.00 C

ATOM 205 HB2 PHE 14 80.621 25.230 18.677 1.00 0.00 H

ATOM 206 HB3 PHE 14 78.973 24.940 18.409 1.00 0.00 H

ATOM 207 CG PHE 14 79.509 27.108 18.276 1.00 0.00 C

ATOM 208 CD1 PHE 14 80.340 27.544 17.256 1.00 0.00 C

ATOM 209 HD1 PHE 14 81.163 26.968 16.858 1.00 0.00 H

ATOM 210 CE1 PHE 14 80.202 28.823 16.681 1.00 0.00 C

ATOM 211 HE1 PHE 14 80.786 29.033 15.797 1.00 0.00 H

ATOM 212 CZ PHE 14 79.149 29.635 17.092 1.00 0.00 C

ATOM 213 HZ PHE 14 78.843 30.468 16.477 1.00 0.00 H

ATOM 214 CE2 PHE 14 78.345 29.275 18.245 1.00 0.00 C

ATOM 215 HE2 PHE 14 77.470 29.814 18.578 1.00 0.00 H

ATOM 216 CD2 PHE 14 78.435 27.917 18.715 1.00 0.00 C

ATOM 217 HD2 PHE 14 77.703 27.503 19.392 1.00 0.00 H

ATOM 218 C PHE 14 80.256 26.659 21.184 1.00 0.00 C

ATOM 219 O PHE 14 81.452 26.468 21.151 1.00 0.00 O

ATOM 220 N TYR 15 79.697 27.561 22.013 1.00 0.00 N

ATOM 221 H TYR 15 78.798 27.936 21.747 1.00 0.00 H

ATOM 222 CA TYR 15 80.389 28.318 23.026 1.00 0.00 C

ATOM 223 HA TYR 15 81.426 27.988 23.089 1.00 0.00 H

ATOM 224 CB TYR 15 80.418 29.800 22.870 1.00 0.00 C

ATOM 225 HB2 TYR 15 79.374 30.073 22.716 1.00 0.00 H

ATOM 226 HB3 TYR 15 80.732 30.239 23.817 1.00 0.00 H

ATOM 227 CG TYR 15 81.253 30.457 21.747 1.00 0.00 C

ATOM 228 CD1 TYR 15 80.843 30.417 20.364 1.00 0.00 C

ATOM 229 HD1 TYR 15 79.797 30.238 20.162 1.00 0.00 H

ATOM 230 CE1 TYR 15 81.589 30.975 19.406 1.00 0.00 C

ATOM 231 HE1 TYR 15 81.193 30.962 18.402 1.00 0.00 H

ATOM 232 CZ TYR 15 82.830 31.655 19.712 1.00 0.00 C

ATOM 233 OH TYR 15 83.416 32.389 18.666 1.00 0.00 O

ATOM 234 HH TYR 15 83.072 32.140 17.805 1.00 0.00 H

ATOM 235 CE2 TYR 15 83.355 31.584 20.981 1.00 0.00 C

ATOM 236 HE2 TYR 15 84.275 32.102 21.209 1.00 0.00 H

ATOM 237 CD2 TYR 15 82.549 30.968 22.005 1.00 0.00 C

ATOM 238 HD2 TYR 15 82.955 31.027 23.004 1.00 0.00 H

ATOM 239 C TYR 15 79.885 27.997 24.420 1.00 0.00 C

ATOM 240 O TYR 15 80.651 27.962 25.384 1.00 0.00 O

ATOM 241 N TYR 16 78.612 27.770 24.497 1.00 0.00 N

ATOM 242 H TYR 16 78.052 27.819 23.658 1.00 0.00 H

ATOM 243 CA TYR 16 77.866 27.738 25.778 1.00 0.00 C

ATOM 244 HA TYR 16 78.325 28.540 26.356 1.00 0.00 H

ATOM 245 CB TYR 16 76.380 28.114 25.483 1.00 0.00 C

ATOM 246 HB2 TYR 16 76.284 29.186 25.651 1.00 0.00 H

ATOM 247 HB3 TYR 16 76.070 27.830 24.477 1.00 0.00 H

ATOM 248 CG TYR 16 75.460 27.382 26.481 1.00 0.00 C

ATOM 249 CD1 TYR 16 75.188 25.982 26.369 1.00 0.00 C

ATOM 250 HD1 TYR 16 75.531 25.400 25.526 1.00 0.00 H

ATOM 251 CE1 TYR 16 74.388 25.292 27.318 1.00 0.00 C

ATOM 252 HE1 TYR 16 74.122 24.257 27.161 1.00 0.00 H

ATOM 253 CZ TYR 16 73.973 25.967 28.467 1.00 0.00 C

ATOM 254 OH TYR 16 73.226 25.354 29.436 1.00 0.00 O

ATOM 255 HH TYR 16 72.755 25.913 30.059 1.00 0.00 H

ATOM 256 CE2 TYR 16 74.425 27.282 28.681 1.00 0.00 C

ATOM 257 HE2 TYR 16 74.164 27.743 29.622 1.00 0.00 H

ATOM 258 CD2 TYR 16 75.180 28.019 27.734 1.00 0.00 C

ATOM 259 HD2 TYR 16 75.305 29.086 27.847 1.00 0.00 H

ATOM 260 C TYR 16 78.126 26.489 26.579 1.00 0.00 C

ATOM 261 O TYR 16 78.461 26.678 27.755 1.00 0.00 O

ATOM 262 N LEU 17 78.410 25.334 25.952 1.00 0.00 N

ATOM 263 H LEU 17 78.015 25.240 25.027 1.00 0.00 H

ATOM 264 CA LEU 17 78.829 24.093 26.633 1.00 0.00 C

ATOM 265 HA LEU 17 78.260 24.178 27.559 1.00 0.00 H

ATOM 266 CB LEU 17 78.422 22.820 25.908 1.00 0.00 C

ATOM 267 HB2 LEU 17 78.539 23.069 24.853 1.00 0.00 H

ATOM 268 HB3 LEU 17 78.935 21.958 26.333 1.00 0.00 H

ATOM 269 CG LEU 17 76.886 22.503 26.122 1.00 0.00 C

ATOM 270 HG LEU 17 76.288 23.403 25.981 1.00 0.00 H

ATOM 271 CD1 LEU 17 76.438 21.374 25.170 1.00 0.00 C

ATOM 272 HD11 LEU 17 76.332 21.843 24.192 1.00 0.00 H

ATOM 273 HD12 LEU 17 77.196 20.595 25.245 1.00 0.00 H

ATOM 274 HD13 LEU 17 75.519 21.012 25.632 1.00 0.00 H

ATOM 275 CD2 LEU 17 76.586 22.059 27.555 1.00 0.00 C

ATOM 276 HD21 LEU 17 75.634 21.549 27.699 1.00 0.00 H

ATOM 277 HD22 LEU 17 77.301 21.276 27.809 1.00 0.00 H

ATOM 278 HD23 LEU 17 76.621 23.015 28.077 1.00 0.00 H

ATOM 279 C LEU 17 80.269 24.080 27.100 1.00 0.00 C

ATOM 280 O LEU 17 80.509 23.755 28.240 1.00 0.00 O

ATOM 281 N PRO 18 81.305 24.544 26.381 1.00 0.00 N

ATOM 282 CD PRO 18 81.314 24.565 24.926 1.00 0.00 C

ATOM 283 HD2 PRO 18 80.512 25.269 24.702 1.00 0.00 H

ATOM 284 HD3 PRO 18 81.131 23.550 24.575 1.00 0.00 H

ATOM 285 CG PRO 18 82.745 24.918 24.448 1.00 0.00 C

ATOM 286 HG2 PRO 18 82.734 25.644 23.636 1.00 0.00 H

ATOM 287 HG3 PRO 18 83.241 24.016 24.089 1.00 0.00 H

ATOM 288 CB PRO 18 83.445 25.473 25.710 1.00 0.00 C

ATOM 289 HB2 PRO 18 83.404 26.562 25.681 1.00 0.00 H

ATOM 290 HB3 PRO 18 84.476 25.118 25.721 1.00 0.00 H

ATOM 291 CA PRO 18 82.674 24.856 26.893 1.00 0.00 C

ATOM 292 HA PRO 18 83.164 23.904 27.094 1.00 0.00 H

ATOM 293 C PRO 18 82.613 25.642 28.164 1.00 0.00 C

ATOM 294 O PRO 18 83.212 25.273 29.133 1.00 0.00 O

ATOM 295 N ALA 19 81.769 26.686 28.154 1.00 0.00 N

ATOM 296 H ALA 19 81.185 26.705 27.331 1.00 0.00 H

ATOM 297 CA ALA 19 81.794 27.755 29.111 1.00 0.00 C

ATOM 298 HA ALA 19 82.868 27.917 29.194 1.00 0.00 H

ATOM 299 CB ALA 19 81.154 29.033 28.553 1.00 0.00 C

ATOM 300 HB1 ALA 19 81.357 29.785 29.316 1.00 0.00 H

ATOM 301 HB2 ALA 19 81.722 29.272 27.654 1.00 0.00 H

ATOM 302 HB3 ALA 19 80.114 28.860 28.280 1.00 0.00 H

ATOM 303 C ALA 19 81.197 27.318 30.476 1.00 0.00 C

ATOM 304 O ALA 19 81.698 27.829 31.494 1.00 0.00 O

ATOM 305 N VAL 20 80.323 26.343 30.533 1.00 0.00 N

ATOM 306 H VAL 20 80.165 25.830 29.677 1.00 0.00 H

ATOM 307 CA VAL 20 79.735 26.002 31.808 1.00 0.00 C

ATOM 308 HA VAL 20 79.951 26.723 32.596 1.00 0.00 H

ATOM 309 CB VAL 20 78.262 25.859 31.685 1.00 0.00 C

ATOM 310 HB VAL 20 77.918 25.460 32.640 1.00 0.00 H

ATOM 311 CG1 VAL 20 77.418 27.134 31.451 1.00 0.00 C

ATOM 312 HG11 VAL 20 77.592 27.848 32.256 1.00 0.00 H

ATOM 313 HG12 VAL 20 77.879 27.559 30.560 1.00 0.00 H

ATOM 314 HG13 VAL 20 76.366 26.899 31.293 1.00 0.00 H

ATOM 315 CG2 VAL 20 77.889 24.781 30.591 1.00 0.00 C

ATOM 316 HG21 VAL 20 77.899 25.267 29.615 1.00 0.00 H

ATOM 317 HG22 VAL 20 78.663 24.027 30.449 1.00 0.00 H

ATOM 318 HG23 VAL 20 76.838 24.504 30.681 1.00 0.00 H

ATOM 319 C VAL 20 80.374 24.655 32.302 1.00 0.00 C

ATOM 320 O VAL 20 80.684 24.523 33.495 1.00 0.00 O

ATOM 321 N TYR 21 80.759 23.746 31.434 1.00 0.00 N

ATOM 322 H TYR 21 80.769 23.969 30.449 1.00 0.00 H

ATOM 323 CA TYR 21 81.707 22.609 31.758 1.00 0.00 C

ATOM 324 HA TYR 21 81.274 22.071 32.601 1.00 0.00 H

ATOM 325 CB TYR 21 81.834 21.638 30.524 1.00 0.00 C

ATOM 326 HB2 TYR 21 81.933 22.350 29.705 1.00 0.00 H

ATOM 327 HB3 TYR 21 82.675 20.992 30.776 1.00 0.00 H

ATOM 328 CG TYR 21 80.630 20.839 30.065 1.00 0.00 C

ATOM 329 CD1 TYR 21 79.789 20.138 30.929 1.00 0.00 C

ATOM 330 HD1 TYR 21 79.874 20.363 31.982 1.00 0.00 H

ATOM 331 CE1 TYR 21 78.846 19.255 30.372 1.00 0.00 C

ATOM 332 HE1 TYR 21 78.194 18.629 30.963 1.00 0.00 H

ATOM 333 CZ TYR 21 78.709 19.106 28.985 1.00 0.00 C

ATOM 334 OH TYR 21 77.606 18.364 28.586 1.00 0.00 O

ATOM 335 HH TYR 21 76.992 18.153 29.293 1.00 0.00 H

ATOM 336 CE2 TYR 21 79.638 19.776 28.151 1.00 0.00 C

ATOM 337 HE2 TYR 21 79.597 19.614 27.084 1.00 0.00 H

ATOM 338 CD2 TYR 21 80.705 20.531 28.679 1.00 0.00 C

ATOM 339 HD2 TYR 21 81.428 21.020 28.044 1.00 0.00 H

ATOM 340 C TYR 21 83.041 23.092 32.355 1.00 0.00 C

ATOM 341 O TYR 21 83.545 22.478 33.279 1.00 0.00 O

ATOM 342 N ILE 22 83.688 24.199 31.842 1.00 0.00 N

ATOM 343 H ILE 22 83.141 24.617 31.103 1.00 0.00 H

ATOM 344 CA ILE 22 84.983 24.706 32.318 1.00 0.00 C

ATOM 345 HA ILE 22 85.520 23.765 32.438 1.00 0.00 H

ATOM 346 CB ILE 22 85.727 25.598 31.317 1.00 0.00 C

ATOM 347 HB ILE 22 85.712 25.097 30.349 1.00 0.00 H

ATOM 348 CG2 ILE 22 85.003 27.012 31.227 1.00 0.00 C

ATOM 349 HG21 ILE 22 84.953 27.525 32.187 1.00 0.00 H

ATOM 350 HG22 ILE 22 85.458 27.645 30.465 1.00 0.00 H

ATOM 351 HG23 ILE 22 83.968 26.878 30.912 1.00 0.00 H

ATOM 352 CG1 ILE 22 87.160 25.816 31.824 1.00 0.00 C

ATOM 353 HG12 ILE 22 87.144 26.419 32.732 1.00 0.00 H

ATOM 354 HG13 ILE 22 87.523 24.810 32.035 1.00 0.00 H

ATOM 355 CD1 ILE 22 88.007 26.404 30.704 1.00 0.00 C

ATOM 356 HD11 ILE 22 89.018 26.632 31.042 1.00 0.00 H

ATOM 357 HD12 ILE 22 88.100 25.726 29.857 1.00 0.00 H

ATOM 358 HD13 ILE 22 87.740 27.399 30.346 1.00 0.00 H

ATOM 359 C ILE 22 84.810 25.361 33.754 1.00 0.00 C

ATOM 360 O ILE 22 85.670 25.307 34.592 1.00 0.00 O

ATOM 361 N LEU 23 83.573 25.754 34.152 1.00 0.00 N

ATOM 362 H LEU 23 82.750 25.561 33.599 1.00 0.00 H

ATOM 363 CA LEU 23 83.237 26.145 35.559 1.00 0.00 C

ATOM 364 HA LEU 23 84.054 26.749 35.954 1.00 0.00 H

ATOM 365 CB LEU 23 81.940 26.976 35.609 1.00 0.00 C

ATOM 366 HB2 LEU 23 82.041 27.927 35.086 1.00 0.00 H

ATOM 367 HB3 LEU 23 81.106 26.531 35.067 1.00 0.00 H

ATOM 368 CG LEU 23 81.446 27.281 37.042 1.00 0.00 C

ATOM 369 HG LEU 23 81.383 26.328 37.568 1.00 0.00 H

ATOM 370 CD1 LEU 23 82.515 28.201 37.729 1.00 0.00 C

ATOM 371 HD11 LEU 23 82.074 28.764 38.551 1.00 0.00 H

ATOM 372 HD12 LEU 23 83.417 27.704 38.087 1.00 0.00 H

ATOM 373 HD13 LEU 23 82.746 28.996 37.020 1.00 0.00 H

ATOM 374 CD2 LEU 23 79.999 27.891 36.995 1.00 0.00 C

ATOM 375 HD21 LEU 23 79.579 27.603 37.958 1.00 0.00 H

ATOM 376 HD22 LEU 23 80.165 28.963 36.882 1.00 0.00 H

ATOM 377 HD23 LEU 23 79.507 27.321 36.206 1.00 0.00 H

ATOM 378 C LEU 23 83.227 24.926 36.518 1.00 0.00 C

ATOM 379 O LEU 23 83.772 24.874 37.613 1.00 0.00 O

ATOM 380 N VAL 24 82.509 23.865 36.082 1.00 0.00 N

ATOM 381 H VAL 24 82.151 23.857 35.138 1.00 0.00 H

ATOM 382 CA VAL 24 82.385 22.640 36.865 1.00 0.00 C

ATOM 383 HA VAL 24 82.013 22.834 37.871 1.00 0.00 H

ATOM 384 CB VAL 24 81.413 21.662 36.168 1.00 0.00 C

ATOM 385 HB VAL 24 81.841 21.295 35.236 1.00 0.00 H

ATOM 386 CG1 VAL 24 81.134 20.369 36.943 1.00 0.00 C

ATOM 387 HG11 VAL 24 80.860 20.492 37.991 1.00 0.00 H

ATOM 388 HG12 VAL 24 80.274 19.871 36.496 1.00 0.00 H

ATOM 389 HG13 VAL 24 82.016 19.735 37.021 1.00 0.00 H

ATOM 390 CG2 VAL 24 80.021 22.315 35.820 1.00 0.00 C

ATOM 391 HG21 VAL 24 80.086 23.382 36.036 1.00 0.00 H

ATOM 392 HG22 VAL 24 79.644 21.933 34.871 1.00 0.00 H

ATOM 393 HG23 VAL 24 79.264 21.988 36.533 1.00 0.00 H

ATOM 394 C VAL 24 83.808 21.976 37.016 1.00 0.00 C

ATOM 395 O VAL 24 84.116 21.626 38.178 1.00 0.00 O

ATOM 396 N PHE 25 84.640 21.862 35.926 1.00 0.00 N

ATOM 397 H PHE 25 84.233 22.222 35.074 1.00 0.00 H

ATOM 398 CA PHE 25 86.011 21.319 35.984 1.00 0.00 C

ATOM 399 HA PHE 25 85.944 20.265 36.255 1.00 0.00 H

ATOM 400 CB PHE 25 86.638 21.534 34.541 1.00 0.00 C

ATOM 401 HB2 PHE 25 86.424 20.646 33.946 1.00 0.00 H

ATOM 402 HB3 PHE 25 86.152 22.386 34.066 1.00 0.00 H

ATOM 403 CG PHE 25 88.200 21.612 34.374 1.00 0.00 C

ATOM 404 CD1 PHE 25 88.750 22.462 33.370 1.00 0.00 C

ATOM 405 HD1 PHE 25 88.043 22.778 32.617 1.00 0.00 H

ATOM 406 CE1 PHE 25 90.160 22.627 33.194 1.00 0.00 C

ATOM 407 HE1 PHE 25 90.524 23.274 32.409 1.00 0.00 H

ATOM 408 CZ PHE 25 90.984 21.920 34.179 1.00 0.00 C

ATOM 409 HZ PHE 25 92.050 22.085 34.214 1.00 0.00 H

ATOM 410 CE2 PHE 25 90.366 21.127 35.191 1.00 0.00 C

ATOM 411 HE2 PHE 25 90.899 20.495 35.887 1.00 0.00 H

ATOM 412 CD2 PHE 25 89.007 20.794 35.149 1.00 0.00 C

ATOM 413 HD2 PHE 25 88.525 20.220 35.926 1.00 0.00 H

ATOM 414 C PHE 25 86.826 21.926 37.121 1.00 0.00 C

ATOM 415 O PHE 25 87.015 21.176 38.069 1.00 0.00 O

ATOM 416 N ILE 26 87.280 23.198 37.106 1.00 0.00 N

ATOM 417 H ILE 26 87.173 23.698 36.235 1.00 0.00 H

ATOM 418 CA ILE 26 88.031 23.876 38.153 1.00 0.00 C

ATOM 419 HA ILE 26 88.961 23.319 38.272 1.00 0.00 H

ATOM 420 CB ILE 26 88.315 25.320 37.764 1.00 0.00 C

ATOM 421 HB ILE 26 88.913 25.702 38.592 1.00 0.00 H

ATOM 422 CG2 ILE 26 89.059 25.286 36.334 1.00 0.00 C

ATOM 423 HG21 ILE 26 88.245 25.301 35.610 1.00 0.00 H

ATOM 424 HG22 ILE 26 89.650 26.184 36.152 1.00 0.00 H

ATOM 425 HG23 ILE 26 89.745 24.443 36.251 1.00 0.00 H

ATOM 426 CG1 ILE 26 87.078 26.262 37.753 1.00 0.00 C

ATOM 427 HG12 ILE 26 86.324 25.826 37.098 1.00 0.00 H

ATOM 428 HG13 ILE 26 86.551 26.394 38.698 1.00 0.00 H

ATOM 429 CD1 ILE 26 87.323 27.660 37.231 1.00 0.00 C

ATOM 430 HD11 ILE 26 86.394 28.229 37.262 1.00 0.00 H

ATOM 431 HD12 ILE 26 88.064 28.217 37.804 1.00 0.00 H

ATOM 432 HD13 ILE 26 87.719 27.555 36.221 1.00 0.00 H

ATOM 433 C ILE 26 87.444 23.742 39.533 1.00 0.00 C

ATOM 434 O ILE 26 88.077 23.305 40.491 1.00 0.00 O

ATOM 435 N ILE 27 86.088 23.874 39.680 1.00 0.00 N

ATOM 436 H ILE 27 85.417 23.952 38.929 1.00 0.00 H

ATOM 437 CA ILE 27 85.389 23.653 40.936 1.00 0.00 C

ATOM 438 HA ILE 27 85.908 24.400 41.537 1.00 0.00 H

ATOM 439 CB ILE 27 83.926 24.036 40.841 1.00 0.00 C

ATOM 440 HB ILE 27 83.622 23.625 39.878 1.00 0.00 H

ATOM 441 CG2 ILE 27 82.980 23.350 41.896 1.00 0.00 C

ATOM 442 HG21 ILE 27 82.947 22.275 41.719 1.00 0.00 H

ATOM 443 HG22 ILE 27 83.277 23.611 42.912 1.00 0.00 H

ATOM 444 HG23 ILE 27 81.933 23.554 41.674 1.00 0.00 H

ATOM 445 CG1 ILE 27 83.594 25.528 40.855 1.00 0.00 C

ATOM 446 HG12 ILE 27 83.995 25.962 41.771 1.00 0.00 H

ATOM 447 HG13 ILE 27 84.175 25.948 40.034 1.00 0.00 H

ATOM 448 CD1 ILE 27 82.150 25.951 40.637 1.00 0.00 C

ATOM 449 HD11 ILE 27 81.612 25.965 41.584 1.00 0.00 H

ATOM 450 HD12 ILE 27 82.177 26.926 40.148 1.00 0.00 H

ATOM 451 HD13 ILE 27 81.633 25.215 40.022 1.00 0.00 H

ATOM 452 C ILE 27 85.643 22.291 41.605 1.00 0.00 C

ATOM 453 O ILE 27 85.999 22.259 42.773 1.00 0.00 O

ATOM 454 N GLY 28 85.473 21.152 40.889 1.00 0.00 N

ATOM 455 H GLY 28 85.192 21.245 39.923 1.00 0.00 H

ATOM 456 CA GLY 28 85.777 19.795 41.364 1.00 0.00 C

ATOM 457 HA2 GLY 28 85.116 19.624 42.213 1.00 0.00 H

ATOM 458 HA3 GLY 28 85.426 19.093 40.607 1.00 0.00 H

ATOM 459 C GLY 28 87.289 19.532 41.556 1.00 0.00 C

ATOM 460 O GLY 28 87.829 19.196 42.614 1.00 0.00 O

ATOM 461 N PHE 29 88.037 19.795 40.485 1.00 0.00 N

ATOM 462 H PHE 29 87.578 20.127 39.649 1.00 0.00 H

ATOM 463 CA PHE 29 89.538 19.749 40.352 1.00 0.00 C

ATOM 464 HA PHE 29 89.689 18.673 40.435 1.00 0.00 H

ATOM 465 CB PHE 29 89.876 20.158 38.918 1.00 0.00 C

ATOM 466 HB2 PHE 29 89.114 19.690 38.295 1.00 0.00 H

ATOM 467 HB3 PHE 29 89.879 21.246 38.985 1.00 0.00 H

ATOM 468 CG PHE 29 91.228 19.627 38.452 1.00 0.00 C

ATOM 469 CD1 PHE 29 91.456 18.212 38.389 1.00 0.00 C

ATOM 470 HD1 PHE 29 90.652 17.516 38.579 1.00 0.00 H

ATOM 471 CE1 PHE 29 92.742 17.712 38.098 1.00 0.00 C

ATOM 472 HE1 PHE 29 92.867 16.642 38.163 1.00 0.00 H

ATOM 473 CZ PHE 29 93.732 18.610 37.746 1.00 0.00 C

ATOM 474 HZ PHE 29 94.740 18.311 37.503 1.00 0.00 H

ATOM 475 CE2 PHE 29 93.498 20.008 37.929 1.00 0.00 C

ATOM 476 HE2 PHE 29 94.321 20.698 37.810 1.00 0.00 H

ATOM 477 CD2 PHE 29 92.278 20.537 38.322 1.00 0.00 C

ATOM 478 HD2 PHE 29 92.150 21.598 38.482 1.00 0.00 H

ATOM 479 C PHE 29 90.310 20.531 41.353 1.00 0.00 C

ATOM 480 O PHE 29 91.478 20.178 41.618 1.00 0.00 O

ATOM 481 N LEU 30 89.703 21.515 42.049 1.00 0.00 N

ATOM 482 H LEU 30 88.840 21.887 41.681 1.00 0.00 H

ATOM 483 CA LEU 30 90.162 21.944 43.358 1.00 0.00 C

ATOM 484 HA LEU 30 91.223 21.723 43.473 1.00 0.00 H

ATOM 485 CB LEU 30 89.966 23.468 43.306 1.00 0.00 C

ATOM 486 HB2 LEU 30 90.432 23.873 42.408 1.00 0.00 H

ATOM 487 HB3 LEU 30 88.916 23.666 43.090 1.00 0.00 H

ATOM 488 CG LEU 30 90.537 24.202 44.547 1.00 0.00 C

ATOM 489 HG LEU 30 90.522 25.272 44.340 1.00 0.00 H

ATOM 490 CD1 LEU 30 89.767 24.034 45.901 1.00 0.00 C

ATOM 491 HD11 LEU 30 89.974 24.766 46.681 1.00 0.00 H

ATOM 492 HD12 LEU 30 88.701 23.984 45.676 1.00 0.00 H

ATOM 493 HD13 LEU 30 89.926 23.099 46.439 1.00 0.00 H

ATOM 494 CD2 LEU 30 91.950 23.979 44.949 1.00 0.00 C

ATOM 495 HD21 LEU 30 92.286 24.601 45.779 1.00 0.00 H

ATOM 496 HD22 LEU 30 92.102 22.946 45.261 1.00 0.00 H

ATOM 497 HD23 LEU 30 92.597 24.130 44.084 1.00 0.00 H

ATOM 498 C LEU 30 89.250 21.247 44.490 1.00 0.00 C

ATOM 499 O LEU 30 89.701 20.360 45.228 1.00 0.00 O

ATOM 500 N GLY 31 87.960 21.524 44.570 1.00 0.00 N

ATOM 501 H GLY 31 87.581 22.157 43.881 1.00 0.00 H

ATOM 502 CA GLY 31 87.161 21.265 45.773 1.00 0.00 C

ATOM 503 HA2 GLY 31 87.442 21.793 46.684 1.00 0.00 H

ATOM 504 HA3 GLY 31 86.106 21.520 45.669 1.00 0.00 H

ATOM 505 C GLY 31 87.039 19.766 46.126 1.00 0.00 C

ATOM 506 O GLY 31 87.225 19.367 47.314 1.00 0.00 O

ATOM 507 N ASN 32 86.648 18.894 45.163 1.00 0.00 N

ATOM 508 H ASN 32 86.838 19.112 44.195 1.00 0.00 H

ATOM 509 CA ASN 32 86.537 17.411 45.284 1.00 0.00 C

ATOM 510 HA ASN 32 86.004 17.171 46.204 1.00 0.00 H

ATOM 511 CB ASN 32 85.585 16.892 44.186 1.00 0.00 C

ATOM 512 HB2 ASN 32 84.571 17.291 44.167 1.00 0.00 H

ATOM 513 HB3 ASN 32 86.075 16.990 43.217 1.00 0.00 H

ATOM 514 CG ASN 32 85.342 15.400 44.421 1.00 0.00 C

ATOM 515 OD1 ASN 32 84.633 15.001 45.255 1.00 0.00 O

ATOM 516 ND2 ASN 32 85.995 14.620 43.584 1.00 0.00 N

ATOM 517 HD21 ASN 32 86.682 15.078 43.002 1.00 0.00 H

ATOM 518 HD22 ASN 32 85.758 13.643 43.482 1.00 0.00 H

ATOM 519 C ASN 32 87.973 16.782 45.415 1.00 0.00 C

ATOM 520 O ASN 32 88.140 15.668 45.970 1.00 0.00 O

ATOM 521 N SER 33 88.938 17.512 44.906 1.00 0.00 N

ATOM 522 H SER 33 88.684 18.324 44.362 1.00 0.00 H

ATOM 523 CA SER 33 90.375 17.117 44.970 1.00 0.00 C

ATOM 524 HA SER 33 90.506 16.052 44.778 1.00 0.00 H

ATOM 525 CB SER 33 91.002 17.759 43.764 1.00 0.00 C

ATOM 526 HB2 SER 33 90.891 18.841 43.694 1.00 0.00 H

ATOM 527 HB3 SER 33 92.087 17.677 43.686 1.00 0.00 H

ATOM 528 OG SER 33 90.511 17.211 42.566 1.00 0.00 O

ATOM 529 HG SER 33 90.622 17.876 41.883 1.00 0.00 H

ATOM 530 C SER 33 91.050 17.576 46.243 1.00 0.00 C

ATOM 531 O SER 33 92.016 16.916 46.656 1.00 0.00 O

ATOM 532 N VAL 34 90.476 18.467 47.010 1.00 0.00 N

ATOM 533 H VAL 34 89.973 19.229 46.577 1.00 0.00 H

ATOM 534 CA VAL 34 90.676 18.556 48.440 1.00 0.00 C

ATOM 535 HA VAL 34 91.727 18.403 48.687 1.00 0.00 H

ATOM 536 CB VAL 34 90.293 19.918 48.998 1.00 0.00 C

ATOM 537 HB VAL 34 89.243 19.972 48.709 1.00 0.00 H

ATOM 538 CG1 VAL 34 90.357 20.079 50.489 1.00 0.00 C

ATOM 539 HG11 VAL 34 91.334 20.103 50.971 1.00 0.00 H

ATOM 540 HG12 VAL 34 89.906 20.964 50.937 1.00 0.00 H

ATOM 541 HG13 VAL 34 89.811 19.247 50.936 1.00 0.00 H

ATOM 542 CG2 VAL 34 91.078 21.061 48.330 1.00 0.00 C

ATOM 543 HG21 VAL 34 91.997 21.197 48.900 1.00 0.00 H

ATOM 544 HG22 VAL 34 91.310 21.024 47.266 1.00 0.00 H

ATOM 545 HG23 VAL 34 90.492 21.961 48.518 1.00 0.00 H

ATOM 546 C VAL 34 89.895 17.474 49.248 1.00 0.00 C

ATOM 547 O VAL 34 90.510 16.570 49.765 1.00 0.00 O

ATOM 548 N ALA 35 88.557 17.414 49.066 1.00 0.00 N

ATOM 549 H ALA 35 88.227 18.080 48.382 1.00 0.00 H

ATOM 550 CA ALA 35 87.649 16.436 49.706 1.00 0.00 C

ATOM 551 HA ALA 35 87.459 16.692 50.748 1.00 0.00 H

ATOM 552 CB ALA 35 86.284 16.438 49.064 1.00 0.00 C

ATOM 553 HB1 ALA 35 85.857 17.439 48.997 1.00 0.00 H

ATOM 554 HB2 ALA 35 86.322 15.930 48.100 1.00 0.00 H

ATOM 555 HB3 ALA 35 85.666 15.798 49.694 1.00 0.00 H

ATOM 556 C ALA 35 88.188 14.958 49.692 1.00 0.00 C

ATOM 557 O ALA 35 88.228 14.336 50.738 1.00 0.00 O

ATOM 558 N ILE 36 88.570 14.398 48.520 1.00 0.00 N

ATOM 559 H ILE 36 88.492 14.969 47.691 1.00 0.00 H

ATOM 560 CA ILE 36 89.200 13.121 48.330 1.00 0.00 C

ATOM 561 HA ILE 36 88.469 12.397 48.689 1.00 0.00 H

ATOM 562 CB ILE 36 89.362 12.799 46.831 1.00 0.00 C

ATOM 563 HB ILE 36 88.425 13.090 46.357 1.00 0.00 H

ATOM 564 CG2 ILE 36 90.521 13.462 46.008 1.00 0.00 C

ATOM 565 HG21 ILE 36 90.481 14.499 46.339 1.00 0.00 H

ATOM 566 HG22 ILE 36 91.531 13.054 46.040 1.00 0.00 H

ATOM 567 HG23 ILE 36 90.258 13.480 44.950 1.00 0.00 H

ATOM 568 CG1 ILE 36 89.591 11.234 46.786 1.00 0.00 C

ATOM 569 HG12 ILE 36 90.522 10.968 47.286 1.00 0.00 H

ATOM 570 HG13 ILE 36 88.737 10.742 47.251 1.00 0.00 H

ATOM 571 CD1 ILE 36 89.718 10.530 45.380 1.00 0.00 C

ATOM 572 HD11 ILE 36 89.758 9.472 45.637 1.00 0.00 H

ATOM 573 HD12 ILE 36 88.822 10.743 44.798 1.00 0.00 H

ATOM 574 HD13 ILE 36 90.601 10.787 44.795 1.00 0.00 H

ATOM 575 C ILE 36 90.593 12.902 49.052 1.00 0.00 C

ATOM 576 O ILE 36 90.828 11.863 49.671 1.00 0.00 O

ATOM 577 N TRP 37 91.246 13.998 49.287 1.00 0.00 N

ATOM 578 H TRP 37 90.757 14.855 49.070 1.00 0.00 H

ATOM 579 CA TRP 37 92.541 14.036 49.929 1.00 0.00 C

ATOM 580 HA TRP 37 93.090 13.106 49.783 1.00 0.00 H

ATOM 581 CB TRP 37 93.333 15.234 49.443 1.00 0.00 C

ATOM 582 HB2 TRP 37 93.125 15.536 48.417 1.00 0.00 H

ATOM 583 HB3 TRP 37 92.890 16.071 49.983 1.00 0.00 H

ATOM 584 CG TRP 37 94.821 15.089 49.399 1.00 0.00 C

ATOM 585 CD1 TRP 37 95.677 14.370 50.249 1.00 0.00 C

ATOM 586 HD1 TRP 37 95.354 14.035 51.223 1.00 0.00 H

ATOM 587 NE1 TRP 37 96.950 14.504 49.795 1.00 0.00 N

ATOM 588 HE1 TRP 37 97.722 14.294 50.411 1.00 0.00 H

ATOM 589 CE2 TRP 37 97.035 15.196 48.608 1.00 0.00 C

ATOM 590 CZ2 TRP 37 98.079 15.401 47.746 1.00 0.00 C

ATOM 591 HZ2 TRP 37 99.071 15.048 47.983 1.00 0.00 H

ATOM 592 CH2 TRP 37 97.858 15.923 46.444 1.00 0.00 C

ATOM 593 HH2 TRP 37 98.624 15.974 45.685 1.00 0.00 H

ATOM 594 CZ3 TRP 37 96.476 16.246 46.109 1.00 0.00 C

ATOM 595 HZ3 TRP 37 96.274 16.496 45.078 1.00 0.00 H

ATOM 596 CE3 TRP 37 95.377 15.998 46.998 1.00 0.00 C

ATOM 597 HE3 TRP 37 94.369 16.207 46.672 1.00 0.00 H

ATOM 598 CD2 TRP 37 95.718 15.473 48.282 1.00 0.00 C

ATOM 599 C TRP 37 92.318 14.165 51.469 1.00 0.00 C

ATOM 600 O TRP 37 93.185 13.812 52.227 1.00 0.00 O

ATOM 601 N MET 38 91.230 14.757 51.912 1.00 0.00 N

ATOM 602 H MET 38 90.614 15.303 51.325 1.00 0.00 H

ATOM 603 CA MET 38 90.916 14.902 53.330 1.00 0.00 C

ATOM 604 HA MET 38 91.820 15.008 53.930 1.00 0.00 H

ATOM 605 CB MET 38 90.197 16.247 53.417 1.00 0.00 C

ATOM 606 HB2 MET 38 90.800 17.100 53.106 1.00 0.00 H

ATOM 607 HB3 MET 38 89.343 16.168 52.744 1.00 0.00 H

ATOM 608 CG MET 38 89.693 16.526 54.829 1.00 0.00 C

ATOM 609 HG2 MET 38 89.315 15.623 55.309 1.00 0.00 H

ATOM 610 HG3 MET 38 90.444 17.030 55.437 1.00 0.00 H

ATOM 611 SD MET 38 88.337 17.784 54.944 1.00 0.00 S

ATOM 612 CE MET 38 88.014 17.634 56.729 1.00 0.00 C

ATOM 613 HE1 MET 38 87.821 18.605 57.185 1.00 0.00 H

ATOM 614 HE2 MET 38 87.213 16.894 56.706 1.00 0.00 H

ATOM 615 HE3 MET 38 88.865 17.144 57.201 1.00 0.00 H

ATOM 616 C MET 38 90.165 13.697 53.930 1.00 0.00 C

ATOM 617 O MET 38 90.344 13.306 55.107 1.00 0.00 O

ATOM 618 N PHE 39 89.371 13.000 53.094 1.00 0.00 N

ATOM 619 H PHE 39 89.245 13.386 52.170 1.00 0.00 H

ATOM 620 CA PHE 39 88.715 11.731 53.533 1.00 0.00 C

ATOM 621 HA PHE 39 88.184 11.830 54.480 1.00 0.00 H

ATOM 622 CB PHE 39 87.706 11.189 52.413 1.00 0.00 C

ATOM 623 HB2 PHE 39 87.980 11.509 51.407 1.00 0.00 H

ATOM 624 HB3 PHE 39 87.751 10.103 52.332 1.00 0.00 H

ATOM 625 CG PHE 39 86.314 11.627 52.759 1.00 0.00 C

ATOM 626 CD1 PHE 39 85.559 12.561 52.063 1.00 0.00 C

ATOM 627 HD1 PHE 39 86.091 13.016 51.240 1.00 0.00 H

ATOM 628 CE1 PHE 39 84.354 13.014 52.611 1.00 0.00 C

ATOM 629 HE1 PHE 39 83.841 13.677 51.930 1.00 0.00 H

ATOM 630 CZ PHE 39 83.855 12.492 53.855 1.00 0.00 C

ATOM 631 HZ PHE 39 82.850 12.714 54.181 1.00 0.00 H

ATOM 632 CE2 PHE 39 84.462 11.425 54.398 1.00 0.00 C

ATOM 633 HE2 PHE 39 84.114 11.207 55.396 1.00 0.00 H

ATOM 634 CD2 PHE 39 85.704 10.987 53.925 1.00 0.00 C

ATOM 635 HD2 PHE 39 86.293 10.273 54.482 1.00 0.00 H

ATOM 636 C PHE 39 89.806 10.716 53.883 1.00 0.00 C

ATOM 637 O PHE 39 89.589 9.797 54.695 1.00 0.00 O

ATOM 638 N VAL 40 90.957 10.676 53.208 1.00 0.00 N

ATOM 639 H VAL 40 91.202 11.438 52.592 1.00 0.00 H

ATOM 640 CA VAL 40 91.888 9.549 53.301 1.00 0.00 C

ATOM 641 HA VAL 40 91.290 8.683 53.584 1.00 0.00 H

ATOM 642 CB VAL 40 92.590 9.198 51.932 1.00 0.00 C

ATOM 643 HB VAL 40 93.161 8.280 52.069 1.00 0.00 H

ATOM 644 CG1 VAL 40 91.491 8.919 50.837 1.00 0.00 C

ATOM 645 HG11 VAL 40 90.979 7.978 51.040 1.00 0.00 H

ATOM 646 HG12 VAL 40 90.702 9.668 50.892 1.00 0.00 H

ATOM 647 HG13 VAL 40 91.934 8.991 49.844 1.00 0.00 H

ATOM 648 CG2 VAL 40 93.529 10.324 51.574 1.00 0.00 C

ATOM 649 HG21 VAL 40 94.447 10.368 52.161 1.00 0.00 H

ATOM 650 HG22 VAL 40 93.938 10.162 50.577 1.00 0.00 H

ATOM 651 HG23 VAL 40 92.887 11.205 51.557 1.00 0.00 H

ATOM 652 C VAL 40 92.940 9.695 54.472 1.00 0.00 C

ATOM 653 O VAL 40 93.459 8.689 54.900 1.00 0.00 O

ATOM 654 N PHE 41 93.183 10.946 54.967 1.00 0.00 N

ATOM 655 H PHE 41 92.728 11.735 54.530 1.00 0.00 H

ATOM 656 CA PHE 41 94.032 11.245 56.103 1.00 0.00 C

ATOM 657 HA PHE 41 94.712 10.409 56.266 1.00 0.00 H

ATOM 658 CB PHE 41 95.026 12.266 55.744 1.00 0.00 C

ATOM 659 HB2 PHE 41 94.558 13.205 55.448 1.00 0.00 H

ATOM 660 HB3 PHE 41 95.436 12.497 56.727 1.00 0.00 H

ATOM 661 CG PHE 41 96.197 11.800 54.900 1.00 0.00 C

ATOM 662 CD1 PHE 41 96.266 12.165 53.560 1.00 0.00 C

ATOM 663 HD1 PHE 41 95.612 12.891 53.101 1.00 0.00 H

ATOM 664 CE1 PHE 41 97.122 11.587 52.740 1.00 0.00 C

ATOM 665 HE1 PHE 41 97.036 11.918 51.715 1.00 0.00 H

ATOM 666 CZ PHE 41 98.120 10.721 53.145 1.00 0.00 C

ATOM 667 HZ PHE 41 98.873 10.352 52.463 1.00 0.00 H

ATOM 668 CE2 PHE 41 98.101 10.267 54.498 1.00 0.00 C

ATOM 669 HE2 PHE 41 98.744 9.478 54.860 1.00 0.00 H

ATOM 670 CD2 PHE 41 97.149 10.836 55.392 1.00 0.00 C

ATOM 671 HD2 PHE 41 97.002 10.480 56.401 1.00 0.00 H

ATOM 672 C PHE 41 93.337 11.419 57.419 1.00 0.00 C

ATOM 673 O PHE 41 93.247 10.589 58.280 1.00 0.00 O

ATOM 674 N HID 42 92.621 12.502 57.409 1.00 0.00 N

ATOM 675 H HID 42 92.613 13.013 56.538 1.00 0.00 H

ATOM 676 CA HID 42 92.224 13.153 58.645 1.00 0.00 C

ATOM 677 HA HID 42 93.044 13.092 59.361 1.00 0.00 H

ATOM 678 CB HID 42 91.723 14.570 58.359 1.00 0.00 C

ATOM 679 HB2 HID 42 92.474 15.122 57.794 1.00 0.00 H

ATOM 680 HB3 HID 42 90.810 14.607 57.765 1.00 0.00 H

ATOM 681 CG HID 42 91.529 15.329 59.600 1.00 0.00 C

ATOM 682 ND1 HID 42 92.584 15.876 60.297 1.00 0.00 N

ATOM 683 HD1 HID 42 93.447 15.410 60.541 1.00 0.00 H

ATOM 684 CE1 HID 42 92.025 16.750 61.131 1.00 0.00 C

ATOM 685 HE1 HID 42 92.503 17.208 61.985 1.00 0.00 H

ATOM 686 NE2 HID 42 90.655 16.883 60.963 1.00 0.00 N

ATOM 687 CD2 HID 42 90.401 15.997 59.910 1.00 0.00 C

ATOM 688 HD2 HID 42 89.514 15.886 59.304 1.00 0.00 H

ATOM 689 C HID 42 91.097 12.295 59.286 1.00 0.00 C

ATOM 690 O HID 42 91.145 11.915 60.467 1.00 0.00 O

ATOM 691 N MET 43 90.126 11.965 58.463 1.00 0.00 N

ATOM 692 H MET 43 90.262 12.315 57.526 1.00 0.00 H

ATOM 693 CA MET 43 88.767 11.478 58.841 1.00 0.00 C

ATOM 694 HA MET 43 88.330 12.316 59.385 1.00 0.00 H

ATOM 695 CB MET 43 87.909 11.240 57.594 1.00 0.00 C

ATOM 696 HB2 MET 43 88.409 10.514 56.953 1.00 0.00 H

ATOM 697 HB3 MET 43 87.035 10.702 57.961 1.00 0.00 H

ATOM 698 CG MET 43 87.526 12.483 56.912 1.00 0.00 C

ATOM 699 HG2 MET 43 88.457 12.965 56.615 1.00 0.00 H

ATOM 700 HG3 MET 43 86.925 12.129 56.074 1.00 0.00 H

ATOM 701 SD MET 43 86.611 13.722 57.788 1.00 0.00 S

ATOM 702 CE MET 43 84.976 12.832 57.939 1.00 0.00 C

ATOM 703 HE1 MET 43 84.353 13.444 58.592 1.00 0.00 H

ATOM 704 HE2 MET 43 84.473 12.721 56.978 1.00 0.00 H

ATOM 705 HE3 MET 43 85.081 11.854 58.410 1.00 0.00 H

ATOM 706 C MET 43 88.875 10.225 59.762 1.00 0.00 C

ATOM 707 O MET 43 89.174 9.170 59.160 1.00 0.00 O

ATOM 708 N LYS 44 88.748 10.355 61.080 1.00 0.00 N

ATOM 709 H LYS 44 88.598 11.261 61.502 1.00 0.00 H

ATOM 710 CA LYS 44 88.981 9.160 61.994 1.00 0.00 C

ATOM 711 HA LYS 44 89.038 8.231 61.428 1.00 0.00 H

ATOM 712 CB LYS 44 90.358 9.343 62.752 1.00 0.00 C

ATOM 713 HB2 LYS 44 91.211 9.254 62.079 1.00 0.00 H

ATOM 714 HB3 LYS 44 90.403 10.290 63.289 1.00 0.00 H

ATOM 715 CG LYS 44 90.543 8.339 63.951 1.00 0.00 C

ATOM 716 HG2 LYS 44 89.856 8.614 64.752 1.00 0.00 H

ATOM 717 HG3 LYS 44 90.397 7.283 63.724 1.00 0.00 H

ATOM 718 CD LYS 44 92.006 8.463 64.420 1.00 0.00 C

ATOM 719 HD2 LYS 44 92.097 9.435 64.905 1.00 0.00 H

ATOM 720 HD3 LYS 44 92.078 7.778 65.265 1.00 0.00 H

ATOM 721 CE LYS 44 93.104 8.099 63.472 1.00 0.00 C

ATOM 722 HE2 LYS 44 92.729 7.300 62.832 1.00 0.00 H

ATOM 723 HE3 LYS 44 93.407 8.985 62.914 1.00 0.00 H

ATOM 724 NZ LYS 44 94.299 7.611 64.147 1.00 0.00 N

ATOM 725 HZ1 LYS 44 94.585 8.153 64.950 1.00 0.00 H

ATOM 726 HZ2 LYS 44 93.938 6.821 64.663 1.00 0.00 H

ATOM 727 HZ3 LYS 44 94.941 7.298 63.433 1.00 0.00 H

ATOM 728 C LYS 44 87.839 8.977 63.026 1.00 0.00 C

ATOM 729 O LYS 44 87.355 9.926 63.639 1.00 0.00 O

ATOM 730 N PRO 45 87.415 7.672 63.313 1.00 0.00 N

ATOM 731 CD PRO 45 86.675 7.325 64.549 1.00 0.00 C

ATOM 732 HD2 PRO 45 85.667 7.737 64.488 1.00 0.00 H

ATOM 733 HD3 PRO 45 87.161 7.611 65.482 1.00 0.00 H

ATOM 734 CG PRO 45 86.592 5.778 64.623 1.00 0.00 C

ATOM 735 HG2 PRO 45 85.728 5.516 65.234 1.00 0.00 H

ATOM 736 HG3 PRO 45 87.562 5.368 64.904 1.00 0.00 H

ATOM 737 CB PRO 45 86.525 5.500 63.140 1.00 0.00 C

ATOM 738 HB2 PRO 45 85.471 5.517 62.866 1.00 0.00 H

ATOM 739 HB3 PRO 45 87.006 4.537 62.966 1.00 0.00 H

ATOM 740 CA PRO 45 87.282 6.574 62.385 1.00 0.00 C

ATOM 741 HA PRO 45 88.243 6.100 62.189 1.00 0.00 H

ATOM 742 C PRO 45 86.739 6.855 61.035 1.00 0.00 C

ATOM 743 O PRO 45 86.270 7.958 60.700 1.00 0.00 O

ATOM 744 N TRP 46 86.787 5.923 60.046 1.00 0.00 N

ATOM 745 H TRP 46 87.223 5.069 60.366 1.00 0.00 H

ATOM 746 CA TRP 46 85.642 5.775 59.129 1.00 0.00 C

ATOM 747 HA TRP 46 85.206 6.759 58.956 1.00 0.00 H

ATOM 748 CB TRP 46 85.978 5.178 57.713 1.00 0.00 C

ATOM 749 HB2 TRP 46 86.230 4.151 57.976 1.00 0.00 H

ATOM 750 HB3 TRP 46 85.033 5.114 57.173 1.00 0.00 H

ATOM 751 CG TRP 46 86.905 5.983 56.947 1.00 0.00 C

ATOM 752 CD1 TRP 46 87.282 7.286 57.142 1.00 0.00 C

ATOM 753 HD1 TRP 46 86.987 7.925 57.961 1.00 0.00 H

ATOM 754 NE1 TRP 46 88.116 7.694 56.107 1.00 0.00 N

ATOM 755 HE1 TRP 46 88.823 8.410 56.195 1.00 0.00 H

ATOM 756 CE2 TRP 46 87.989 6.814 55.040 1.00 0.00 C

ATOM 757 CZ2 TRP 46 88.445 6.868 53.733 1.00 0.00 C

ATOM 758 HZ2 TRP 46 88.778 7.827 53.364 1.00 0.00 H

ATOM 759 CH2 TRP 46 88.132 5.802 52.894 1.00 0.00 C

ATOM 760 HH2 TRP 46 88.342 5.908 51.840 1.00 0.00 H

ATOM 761 CZ3 TRP 46 87.316 4.744 53.319 1.00 0.00 C

ATOM 762 HZ3 TRP 46 87.181 3.865 52.707 1.00 0.00 H

ATOM 763 CE3 TRP 46 87.003 4.604 54.674 1.00 0.00 C

ATOM 764 HE3 TRP 46 86.450 3.763 55.065 1.00 0.00 H

ATOM 765 CD2 TRP 46 87.334 5.651 55.554 1.00 0.00 C

ATOM 766 C TRP 46 84.458 5.083 59.826 1.00 0.00 C

ATOM 767 O TRP 46 84.618 4.178 60.642 1.00 0.00 O

ATOM 768 N SER 47 83.260 5.249 59.215 1.00 0.00 N

ATOM 769 H SER 47 83.318 5.897 58.443 1.00 0.00 H

ATOM 770 CA SER 47 82.015 4.437 59.245 1.00 0.00 C

ATOM 771 HA SER 47 82.171 3.616 59.944 1.00 0.00 H

ATOM 772 CB SER 47 80.748 5.132 59.738 1.00 0.00 C

ATOM 773 HB2 SER 47 79.997 4.349 59.641 1.00 0.00 H

ATOM 774 HB3 SER 47 80.841 5.369 60.798 1.00 0.00 H

ATOM 775 OG SER 47 80.291 6.079 58.911 1.00 0.00 O

ATOM 776 HG SER 47 79.751 6.664 59.446 1.00 0.00 H

ATOM 777 C SER 47 81.739 3.885 57.830 1.00 0.00 C

ATOM 778 O SER 47 82.499 4.124 56.849 1.00 0.00 O

ATOM 779 N GLY 48 80.616 3.168 57.662 1.00 0.00 N

ATOM 780 H GLY 48 80.004 3.018 58.451 1.00 0.00 H

ATOM 781 CA GLY 48 80.229 2.790 56.345 1.00 0.00 C

ATOM 782 HA2 GLY 48 80.996 2.203 55.841 1.00 0.00 H

ATOM 783 HA3 GLY 48 79.354 2.199 56.617 1.00 0.00 H

ATOM 784 C GLY 48 79.893 3.977 55.428 1.00 0.00 C

ATOM 785 O GLY 48 80.150 3.953 54.227 1.00 0.00 O

ATOM 786 N ILE 49 79.649 5.152 56.030 1.00 0.00 N

ATOM 787 H ILE 49 79.828 5.235 57.021 1.00 0.00 H

ATOM 788 CA ILE 49 79.356 6.376 55.293 1.00 0.00 C

ATOM 789 HA ILE 49 79.011 6.014 54.325 1.00 0.00 H

ATOM 790 CB ILE 49 78.192 7.078 55.892 1.00 0.00 C

ATOM 791 HB ILE 49 78.170 7.015 56.980 1.00 0.00 H

ATOM 792 CG2 ILE 49 78.274 8.567 55.712 1.00 0.00 C

ATOM 793 HG21 ILE 49 78.995 9.124 56.310 1.00 0.00 H

ATOM 794 HG22 ILE 49 78.146 8.933 54.693 1.00 0.00 H

ATOM 795 HG23 ILE 49 77.470 8.964 56.332 1.00 0.00 H

ATOM 796 CG1 ILE 49 76.914 6.483 55.237 1.00 0.00 C

ATOM 797 HG12 ILE 49 76.938 6.529 54.148 1.00 0.00 H

ATOM 798 HG13 ILE 49 76.823 5.463 55.613 1.00 0.00 H

ATOM 799 CD1 ILE 49 75.640 7.160 55.689 1.00 0.00 C

ATOM 800 HD11 ILE 49 75.590 8.242 55.566 1.00 0.00 H

ATOM 801 HD12 ILE 49 74.787 6.825 55.098 1.00 0.00 H

ATOM 802 HD13 ILE 49 75.597 6.915 56.750 1.00 0.00 H

ATOM 803 C ILE 49 80.640 7.104 54.815 1.00 0.00 C

ATOM 804 O ILE 49 80.583 7.517 53.715 1.00 0.00 O

ATOM 805 N SER 50 81.705 7.208 55.551 1.00 0.00 N

ATOM 806 H SER 50 81.760 6.811 56.478 1.00 0.00 H

ATOM 807 CA SER 50 83.001 7.670 54.997 1.00 0.00 C

ATOM 808 HA SER 50 82.860 8.684 54.622 1.00 0.00 H

ATOM 809 CB SER 50 83.983 7.667 56.207 1.00 0.00 C

ATOM 810 HB2 SER 50 84.478 6.713 56.386 1.00 0.00 H

ATOM 811 HB3 SER 50 84.792 8.339 55.920 1.00 0.00 H

ATOM 812 OG SER 50 83.410 7.997 57.510 1.00 0.00 O

ATOM 813 HG SER 50 83.023 8.874 57.451 1.00 0.00 H

ATOM 814 C SER 50 83.568 6.776 53.811 1.00 0.00 C

ATOM 815 O SER 50 83.998 7.305 52.793 1.00 0.00 O

ATOM 816 N VAL 51 83.477 5.468 53.945 1.00 0.00 N

ATOM 817 H VAL 51 82.839 5.192 54.679 1.00 0.00 H

ATOM 818 CA VAL 51 83.694 4.607 52.818 1.00 0.00 C

ATOM 819 HA VAL 51 84.741 4.634 52.516 1.00 0.00 H

ATOM 820 CB VAL 51 83.261 3.238 53.261 1.00 0.00 C

ATOM 821 HB VAL 51 82.290 3.386 53.734 1.00 0.00 H

ATOM 822 CG1 VAL 51 82.998 2.234 52.092 1.00 0.00 C

ATOM 823 HG11 VAL 51 82.118 2.618 51.575 1.00 0.00 H

ATOM 824 HG12 VAL 51 83.818 2.087 51.389 1.00 0.00 H

ATOM 825 HG13 VAL 51 82.850 1.212 52.441 1.00 0.00 H

ATOM 826 CG2 VAL 51 84.191 2.473 54.248 1.00 0.00 C

ATOM 827 HG21 VAL 51 84.223 2.934 55.236 1.00 0.00 H

ATOM 828 HG22 VAL 51 83.728 1.494 54.377 1.00 0.00 H

ATOM 829 HG23 VAL 51 85.133 2.339 53.717 1.00 0.00 H

ATOM 830 C VAL 51 82.855 4.942 51.591 1.00 0.00 C

ATOM 831 O VAL 51 83.435 4.947 50.534 1.00 0.00 O

ATOM 832 N TYR 52 81.523 5.258 51.620 1.00 0.00 N

ATOM 833 H TYR 52 81.138 5.379 52.546 1.00 0.00 H

ATOM 834 CA TYR 52 80.804 5.724 50.423 1.00 0.00 C

ATOM 835 HA TYR 52 81.075 5.012 49.643 1.00 0.00 H

ATOM 836 CB TYR 52 79.311 5.751 50.640 1.00 0.00 C

ATOM 837 HB2 TYR 52 79.038 6.551 51.329 1.00 0.00 H

ATOM 838 HB3 TYR 52 78.797 6.167 49.772 1.00 0.00 H

ATOM 839 CG TYR 52 78.664 4.433 50.970 1.00 0.00 C

ATOM 840 CD1 TYR 52 78.831 3.202 50.269 1.00 0.00 C

ATOM 841 HD1 TYR 52 79.603 3.146 49.516 1.00 0.00 H

ATOM 842 CE1 TYR 52 78.187 2.047 50.691 1.00 0.00 C

ATOM 843 HE1 TYR 52 78.203 1.144 50.099 1.00 0.00 H

ATOM 844 CZ TYR 52 77.333 2.086 51.825 1.00 0.00 C

ATOM 845 OH TYR 52 76.692 0.942 52.129 1.00 0.00 O

ATOM 846 HH TYR 52 76.196 0.946 52.951 1.00 0.00 H

ATOM 847 CE2 TYR 52 77.060 3.370 52.455 1.00 0.00 C

ATOM 848 HE2 TYR 52 76.271 3.411 53.192 1.00 0.00 H

ATOM 849 CD2 TYR 52 77.777 4.532 51.993 1.00 0.00 C

ATOM 850 HD2 TYR 52 77.498 5.518 52.336 1.00 0.00 H

ATOM 851 C TYR 52 81.207 7.097 49.981 1.00 0.00 C

ATOM 852 O TYR 52 80.985 7.361 48.805 1.00 0.00 O

ATOM 853 N MET 53 81.487 8.086 50.859 1.00 0.00 N

ATOM 854 H MET 53 81.157 7.869 51.788 1.00 0.00 H

ATOM 855 CA MET 53 81.755 9.455 50.467 1.00 0.00 C

ATOM 856 HA MET 53 80.950 9.877 49.865 1.00 0.00 H

ATOM 857 CB MET 53 82.087 10.380 51.668 1.00 0.00 C

ATOM 858 HB2 MET 53 82.977 10.080 52.220 1.00 0.00 H

ATOM 859 HB3 MET 53 82.265 11.382 51.278 1.00 0.00 H

ATOM 860 CG MET 53 80.968 10.677 52.674 1.00 0.00 C

ATOM 861 HG2 MET 53 80.234 9.873 52.722 1.00 0.00 H

ATOM 862 HG3 MET 53 81.496 10.695 53.627 1.00 0.00 H

ATOM 863 SD MET 53 80.097 12.218 52.421 1.00 0.00 S

ATOM 864 CE MET 53 78.483 11.603 51.896 1.00 0.00 C

ATOM 865 HE1 MET 53 78.561 10.847 51.116 1.00 0.00 H

ATOM 866 HE2 MET 53 77.974 11.036 52.675 1.00 0.00 H

ATOM 867 HE3 MET 53 77.961 12.522 51.630 1.00 0.00 H

ATOM 868 C MET 53 82.984 9.478 49.568 1.00 0.00 C

ATOM 869 O MET 53 83.105 10.099 48.490 1.00 0.00 O

ATOM 870 N PHE 54 84.042 8.729 49.948 1.00 0.00 N

ATOM 871 H PHE 54 84.000 8.301 50.862 1.00 0.00 H

ATOM 872 CA PHE 54 85.359 8.718 49.319 1.00 0.00 C

ATOM 873 HA PHE 54 85.659 9.764 49.242 1.00 0.00 H

ATOM 874 CB PHE 54 86.252 7.896 50.231 1.00 0.00 C

ATOM 875 HB2 PHE 54 86.608 8.551 51.025 1.00 0.00 H

ATOM 876 HB3 PHE 54 85.705 7.083 50.708 1.00 0.00 H

ATOM 877 CG PHE 54 87.484 7.323 49.465 1.00 0.00 C

ATOM 878 CD1 PHE 54 88.540 8.279 49.189 1.00 0.00 C

ATOM 879 HD1 PHE 54 88.349 9.299 49.490 1.00 0.00 H

ATOM 880 CE1 PHE 54 89.613 7.894 48.427 1.00 0.00 C

ATOM 881 HE1 PHE 54 90.280 8.657 48.052 1.00 0.00 H

ATOM 882 CZ PHE 54 89.680 6.566 47.925 1.00 0.00 C

ATOM 883 HZ PHE 54 90.466 6.322 47.226 1.00 0.00 H

ATOM 884 CE2 PHE 54 88.619 5.661 48.173 1.00 0.00 C

ATOM 885 HE2 PHE 54 88.649 4.670 47.744 1.00 0.00 H

ATOM 886 CD2 PHE 54 87.562 6.056 48.968 1.00 0.00 C

ATOM 887 HD2 PHE 54 86.812 5.280 49.007 1.00 0.00 H

ATOM 888 C PHE 54 85.173 8.148 47.909 1.00 0.00 C

ATOM 889 O PHE 54 85.551 8.825 46.949 1.00 0.00 O

ATOM 890 N ASN 55 84.561 6.965 47.754 1.00 0.00 N

ATOM 891 H ASN 55 84.181 6.642 48.633 1.00 0.00 H

ATOM 892 CA ASN 55 83.912 6.313 46.615 1.00 0.00 C

ATOM 893 HA ASN 55 84.676 5.802 46.030 1.00 0.00 H

ATOM 894 CB ASN 55 83.100 5.053 47.125 1.00 0.00 C

ATOM 895 HB2 ASN 55 82.401 5.242 47.940 1.00 0.00 H

ATOM 896 HB3 ASN 55 82.254 4.985 46.441 1.00 0.00 H

ATOM 897 CG ASN 55 83.785 3.769 47.213 1.00 0.00 C

ATOM 898 OD1 ASN 55 84.188 3.172 46.221 1.00 0.00 O

ATOM 899 ND2 ASN 55 84.318 3.494 48.386 1.00 0.00 N

ATOM 900 HD21 ASN 55 84.922 2.685 48.364 1.00 0.00 H

ATOM 901 HD22 ASN 55 84.045 4.048 49.185 1.00 0.00 H

ATOM 902 C ASN 55 83.067 7.198 45.693 1.00 0.00 C

ATOM 903 O ASN 55 83.344 7.276 44.465 1.00 0.00 O

ATOM 904 N LEU 56 82.113 7.956 46.213 1.00 0.00 N

ATOM 905 H LEU 56 82.006 7.787 47.203 1.00 0.00 H

ATOM 906 CA LEU 56 81.308 8.903 45.438 1.00 0.00 C

ATOM 907 HA LEU 56 80.889 8.420 44.555 1.00 0.00 H

ATOM 908 CB LEU 56 80.100 9.378 46.347 1.00 0.00 C

ATOM 909 HB2 LEU 56 79.349 8.601 46.498 1.00 0.00 H

ATOM 910 HB3 LEU 56 80.323 9.590 47.393 1.00 0.00 H

ATOM 911 CG LEU 56 79.251 10.540 45.771 1.00 0.00 C

ATOM 912 HG LEU 56 79.250 10.471 44.684 1.00 0.00 H

ATOM 913 CD1 LEU 56 77.761 10.374 46.218 1.00 0.00 C

ATOM 914 HD11 LEU 56 77.110 11.228 46.030 1.00 0.00 H

ATOM 915 HD12 LEU 56 77.451 9.463 45.705 1.00 0.00 H

ATOM 916 HD13 LEU 56 77.850 10.300 47.302 1.00 0.00 H

ATOM 917 CD2 LEU 56 79.713 11.852 46.424 1.00 0.00 C

ATOM 918 HD21 LEU 56 80.756 12.001 46.148 1.00 0.00 H

ATOM 919 HD22 LEU 56 79.015 12.551 45.964 1.00 0.00 H

ATOM 920 HD23 LEU 56 79.684 11.851 47.514 1.00 0.00 H

ATOM 921 C LEU 56 82.200 10.053 44.958 1.00 0.00 C

ATOM 922 O LEU 56 81.980 10.547 43.844 1.00 0.00 O

ATOM 923 N ALA 57 83.303 10.377 45.659 1.00 0.00 N

ATOM 924 H ALA 57 83.419 10.007 46.592 1.00 0.00 H

ATOM 925 CA ALA 57 84.174 11.400 45.130 1.00 0.00 C

ATOM 926 HA ALA 57 83.552 12.155 44.648 1.00 0.00 H

ATOM 927 CB ALA 57 85.085 12.048 46.140 1.00 0.00 C

ATOM 928 HB1 ALA 57 84.546 12.281 47.059 1.00 0.00 H

ATOM 929 HB2 ALA 57 85.873 11.351 46.424 1.00 0.00 H

ATOM 930 HB3 ALA 57 85.461 13.015 45.805 1.00 0.00 H

ATOM 931 C ALA 57 85.090 10.823 43.958 1.00 0.00 C

ATOM 932 O ALA 57 85.299 11.564 43.040 1.00 0.00 O

ATOM 933 N LEU 58 85.533 9.593 44.069 1.00 0.00 N

ATOM 934 H LEU 58 85.283 9.109 44.920 1.00 0.00 H

ATOM 935 CA LEU 58 86.247 8.806 43.054 1.00 0.00 C

ATOM 936 HA LEU 58 87.054 9.435 42.677 1.00 0.00 H

ATOM 937 CB LEU 58 86.836 7.525 43.654 1.00 0.00 C

ATOM 938 HB2 LEU 58 87.430 7.910 44.482 1.00 0.00 H

ATOM 939 HB3 LEU 58 86.135 6.896 44.203 1.00 0.00 H

ATOM 940 CG LEU 58 87.729 6.709 42.729 1.00 0.00 C

ATOM 941 HG LEU 58 87.089 6.396 41.904 1.00 0.00 H

ATOM 942 CD1 LEU 58 88.757 7.686 42.187 1.00 0.00 C

ATOM 943 HD11 LEU 58 89.546 7.115 41.697 1.00 0.00 H

ATOM 944 HD12 LEU 58 88.262 8.184 41.352 1.00 0.00 H

ATOM 945 HD13 LEU 58 89.016 8.419 42.950 1.00 0.00 H

ATOM 946 CD2 LEU 58 88.538 5.523 43.362 1.00 0.00 C

ATOM 947 HD21 LEU 58 89.332 5.968 43.962 1.00 0.00 H

ATOM 948 HD22 LEU 58 87.850 4.892 43.924 1.00 0.00 H

ATOM 949 HD23 LEU 58 88.981 4.942 42.553 1.00 0.00 H

ATOM 950 C LEU 58 85.486 8.605 41.745 1.00 0.00 C

ATOM 951 O LEU 58 86.021 8.745 40.692 1.00 0.00 O

ATOM 952 N ALA 59 84.159 8.478 41.842 1.00 0.00 N

ATOM 953 H ALA 59 83.808 8.201 42.748 1.00 0.00 H

ATOM 954 CA ALA 59 83.233 8.522 40.663 1.00 0.00 C

ATOM 955 HA ALA 59 83.658 7.780 39.986 1.00 0.00 H

ATOM 956 CB ALA 59 81.848 8.047 41.223 1.00 0.00 C

ATOM 957 HB1 ALA 59 81.976 7.187 41.880 1.00 0.00 H

ATOM 958 HB2 ALA 59 81.565 8.898 41.843 1.00 0.00 H

ATOM 959 HB3 ALA 59 81.090 7.880 40.458 1.00 0.00 H

ATOM 960 C ALA 59 83.083 9.892 40.030 1.00 0.00 C

ATOM 961 O ALA 59 83.118 10.005 38.859 1.00 0.00 O

ATOM 962 N ASP 60 83.082 10.952 40.867 1.00 0.00 N

ATOM 963 H ASP 60 82.983 10.643 41.824 1.00 0.00 H

ATOM 964 CA ASP 60 83.119 12.365 40.449 1.00 0.00 C

ATOM 965 HA ASP 60 82.426 12.525 39.623 1.00 0.00 H

ATOM 966 CB ASP 60 82.542 13.291 41.532 1.00 0.00 C

ATOM 967 HB2 ASP 60 82.239 12.822 42.468 1.00 0.00 H

ATOM 968 HB3 ASP 60 83.288 13.907 42.033 1.00 0.00 H

ATOM 969 CG ASP 60 81.186 14.005 41.167 1.00 0.00 C

ATOM 970 OD1 ASP 60 80.183 13.428 40.721 1.00 0.00 O

ATOM 971 OD2 ASP 60 81.084 15.208 41.396 1.00 0.00 O

ATOM 972 C ASP 60 84.483 12.824 39.877 1.00 0.00 C

ATOM 973 O ASP 60 84.576 13.463 38.835 1.00 0.00 O

ATOM 974 N PHE 61 85.648 12.557 40.543 1.00 0.00 N

ATOM 975 H PHE 61 85.516 12.063 41.414 1.00 0.00 H

ATOM 976 CA PHE 61 87.023 12.724 40.216 1.00 0.00 C

ATOM 977 HA PHE 61 87.290 13.778 40.301 1.00 0.00 H

ATOM 978 CB PHE 61 87.993 11.923 41.132 1.00 0.00 C

ATOM 979 HB2 PHE 61 87.730 12.129 42.170 1.00 0.00 H

ATOM 980 HB3 PHE 61 87.684 10.882 41.041 1.00 0.00 H

ATOM 981 CG PHE 61 89.467 12.091 40.931 1.00 0.00 C

ATOM 982 CD1 PHE 61 90.258 13.058 41.641 1.00 0.00 C

ATOM 983 HD1 PHE 61 89.755 13.595 42.432 1.00 0.00 H

ATOM 984 CE1 PHE 61 91.609 13.138 41.291 1.00 0.00 C

ATOM 985 HE1 PHE 61 92.275 13.684 41.943 1.00 0.00 H

ATOM 986 CZ PHE 61 92.166 12.321 40.321 1.00 0.00 C

ATOM 987 HZ PHE 61 93.225 12.322 40.110 1.00 0.00 H

ATOM 988 CE2 PHE 61 91.388 11.500 39.570 1.00 0.00 C

ATOM 989 HE2 PHE 61 91.911 10.881 38.856 1.00 0.00 H

ATOM 990 CD2 PHE 61 90.017 11.397 39.799 1.00 0.00 C

ATOM 991 HD2 PHE 61 89.455 10.544 39.449 1.00 0.00 H

ATOM 992 C PHE 61 87.270 12.386 38.708 1.00 0.00 C

ATOM 993 O PHE 61 87.874 13.190 37.997 1.00 0.00 O

ATOM 994 N LEU 62 86.860 11.155 38.318 1.00 0.00 N

ATOM 995 H LEU 62 86.712 10.450 39.026 1.00 0.00 H

ATOM 996 CA LEU 62 86.920 10.592 36.980 1.00 0.00 C

ATOM 997 HA LEU 62 87.974 10.576 36.703 1.00 0.00 H

ATOM 998 CB LEU 62 86.242 9.244 37.071 1.00 0.00 C

ATOM 999 HB2 LEU 62 86.806 8.531 37.672 1.00 0.00 H

ATOM 1000 HB3 LEU 62 85.297 9.310 37.611 1.00 0.00 H

ATOM 1001 CG LEU 62 86.074 8.519 35.722 1.00 0.00 C

ATOM 1002 HG LEU 62 85.364 9.082 35.117 1.00 0.00 H

ATOM 1003 CD1 LEU 62 87.408 8.193 35.001 1.00 0.00 C

ATOM 1004 HD11 LEU 62 87.234 7.534 34.151 1.00 0.00 H

ATOM 1005 HD12 LEU 62 87.776 9.177 34.710 1.00 0.00 H

ATOM 1006 HD13 LEU 62 88.077 7.621 35.644 1.00 0.00 H

ATOM 1007 CD2 LEU 62 85.466 7.181 36.113 1.00 0.00 C

ATOM 1008 HD21 LEU 62 86.176 6.368 36.265 1.00 0.00 H

ATOM 1009 HD22 LEU 62 84.783 7.279 36.956 1.00 0.00 H

ATOM 1010 HD23 LEU 62 84.833 6.895 35.273 1.00 0.00 H

ATOM 1011 C LEU 62 86.188 11.433 35.963 1.00 0.00 C

ATOM 1012 O LEU 62 86.713 11.628 34.848 1.00 0.00 O

ATOM 1013 N TYR 63 84.953 11.848 36.206 1.00 0.00 N

ATOM 1014 H TYR 63 84.611 11.618 37.128 1.00 0.00 H

ATOM 1015 CA TYR 63 84.214 12.856 35.405 1.00 0.00 C

ATOM 1016 HA TYR 63 84.213 12.420 34.406 1.00 0.00 H

ATOM 1017 CB TYR 63 82.742 13.101 35.884 1.00 0.00 C

ATOM 1018 HB2 TYR 63 82.287 12.131 36.082 1.00 0.00 H

ATOM 1019 HB3 TYR 63 82.672 13.666 36.814 1.00 0.00 H

ATOM 1020 CG TYR 63 81.799 13.799 35.000 1.00 0.00 C

ATOM 1021 CD1 TYR 63 81.502 13.172 33.792 1.00 0.00 C

ATOM 1022 HD1 TYR 63 81.987 12.353 33.282 1.00 0.00 H

ATOM 1023 CE1 TYR 63 80.748 13.885 32.782 1.00 0.00 C

ATOM 1024 HE1 TYR 63 80.447 13.508 31.816 1.00 0.00 H

ATOM 1025 CZ TYR 63 80.251 15.102 33.152 1.00 0.00 C

ATOM 1026 OH TYR 63 79.616 15.804 32.203 1.00 0.00 O

ATOM 1027 HH TYR 63 79.129 16.536 32.589 1.00 0.00 H

ATOM 1028 CE2 TYR 63 80.575 15.805 34.356 1.00 0.00 C

ATOM 1029 HE2 TYR 63 79.933 16.659 34.517 1.00 0.00 H

ATOM 1030 CD2 TYR 63 81.310 15.092 35.325 1.00 0.00 C

ATOM 1031 HD2 TYR 63 81.571 15.508 36.287 1.00 0.00 H

ATOM 1032 C TYR 63 84.936 14.167 35.240 1.00 0.00 C

ATOM 1033 O TYR 63 85.142 14.609 34.095 1.00 0.00 O

ATOM 1034 N VAL 64 85.398 14.828 36.314 1.00 0.00 N

ATOM 1035 H VAL 64 85.266 14.375 37.207 1.00 0.00 H

ATOM 1036 CA VAL 64 85.702 16.283 36.252 1.00 0.00 C

ATOM 1037 HA VAL 64 85.135 16.635 35.390 1.00 0.00 H

ATOM 1038 CB VAL 64 85.223 17.108 37.450 1.00 0.00 C

ATOM 1039 HB VAL 64 85.232 18.188 37.308 1.00 0.00 H

ATOM 1040 CG1 VAL 64 83.725 16.708 37.522 1.00 0.00 C

ATOM 1041 HG11 VAL 64 83.164 17.454 38.085 1.00 0.00 H

ATOM 1042 HG12 VAL 64 83.203 16.764 36.567 1.00 0.00 H

ATOM 1043 HG13 VAL 64 83.555 15.852 38.176 1.00 0.00 H

ATOM 1044 CG2 VAL 64 86.046 16.736 38.689 1.00 0.00 C

ATOM 1045 HG21 VAL 64 87.075 17.095 38.647 1.00 0.00 H

ATOM 1046 HG22 VAL 64 85.554 17.205 39.542 1.00 0.00 H

ATOM 1047 HG23 VAL 64 86.016 15.663 38.878 1.00 0.00 H

ATOM 1048 C VAL 64 87.155 16.345 35.820 1.00 0.00 C

ATOM 1049 O VAL 64 87.661 17.286 35.221 1.00 0.00 O

ATOM 1050 N LEU 65 87.973 15.282 35.912 1.00 0.00 N

ATOM 1051 H LEU 65 87.654 14.623 36.608 1.00 0.00 H

ATOM 1052 CA LEU 65 89.360 15.326 35.286 1.00 0.00 C

ATOM 1053 HA LEU 65 89.854 16.258 35.563 1.00 0.00 H

ATOM 1054 CB LEU 65 90.149 14.173 36.059 1.00 0.00 C

ATOM 1055 HB2 LEU 65 90.409 14.596 37.029 1.00 0.00 H

ATOM 1056 HB3 LEU 65 89.615 13.243 36.254 1.00 0.00 H

ATOM 1057 CG LEU 65 91.378 13.690 35.260 1.00 0.00 C

ATOM 1058 HG LEU 65 91.080 13.428 34.244 1.00 0.00 H

ATOM 1059 CD1 LEU 65 92.519 14.722 35.099 1.00 0.00 C

ATOM 1060 HD11 LEU 65 93.467 14.276 34.798 1.00 0.00 H

ATOM 1061 HD12 LEU 65 92.402 15.567 34.421 1.00 0.00 H

ATOM 1062 HD13 LEU 65 92.850 15.144 36.048 1.00 0.00 H

ATOM 1063 CD2 LEU 65 92.075 12.414 35.748 1.00 0.00 C

ATOM 1064 HD21 LEU 65 91.408 11.556 35.835 1.00 0.00 H

ATOM 1065 HD22 LEU 65 92.651 12.044 34.899 1.00 0.00 H

ATOM 1066 HD23 LEU 65 92.694 12.626 36.620 1.00 0.00 H

ATOM 1067 C LEU 65 89.263 14.975 33.813 1.00 0.00 C

ATOM 1068 O LEU 65 90.163 15.469 33.083 1.00 0.00 O

ATOM 1069 N THR 66 88.226 14.329 33.295 1.00 0.00 N

ATOM 1070 H THR 66 87.481 14.020 33.903 1.00 0.00 H

ATOM 1071 CA THR 66 88.193 13.991 31.878 1.00 0.00 C

ATOM 1072 HA THR 66 89.154 14.017 31.364 1.00 0.00 H

ATOM 1073 CB THR 66 87.902 12.547 31.769 1.00 0.00 C

ATOM 1074 HB THR 66 87.757 12.365 30.704 1.00 0.00 H

ATOM 1075 CG2 THR 66 89.035 11.634 32.353 1.00 0.00 C

ATOM 1076 HG21 THR 66 90.008 12.112 32.465 1.00 0.00 H

ATOM 1077 HG22 THR 66 88.596 11.192 33.247 1.00 0.00 H

ATOM 1078 HG23 THR 66 89.276 10.877 31.606 1.00 0.00 H

ATOM 1079 OG1 THR 66 86.613 12.291 32.354 1.00 0.00 O

ATOM 1080 HG1 THR 66 86.826 11.956 33.228 1.00 0.00 H

ATOM 1081 C THR 66 87.285 14.822 31.030 1.00 0.00 C

ATOM 1082 O THR 66 87.381 14.871 29.813 1.00 0.00 O

ATOM 1083 N LEU 67 86.523 15.677 31.706 1.00 0.00 N

ATOM 1084 H LEU 67 86.509 15.628 32.715 1.00 0.00 H

ATOM 1085 CA LEU 67 85.743 16.764 31.238 1.00 0.00 C

ATOM 1086 HA LEU 67 84.920 16.324 30.675 1.00 0.00 H

ATOM 1087 CB LEU 67 85.120 17.520 32.403 1.00 0.00 C

ATOM 1088 HB2 LEU 67 84.547 16.690 32.816 1.00 0.00 H

ATOM 1089 HB3 LEU 67 85.793 17.831 33.203 1.00 0.00 H

ATOM 1090 CG LEU 67 84.126 18.623 32.091 1.00 0.00 C

ATOM 1091 HG LEU 67 84.805 19.323 31.604 1.00 0.00 H

ATOM 1092 CD1 LEU 67 83.045 18.270 31.077 1.00 0.00 C

ATOM 1093 HD11 LEU 67 82.916 17.203 30.897 1.00 0.00 H

ATOM 1094 HD12 LEU 67 82.084 18.717 31.329 1.00 0.00 H

ATOM 1095 HD13 LEU 67 83.287 18.650 30.084 1.00 0.00 H

ATOM 1096 CD2 LEU 67 83.592 19.074 33.382 1.00 0.00 C

ATOM 1097 HD21 LEU 67 82.786 19.806 33.345 1.00 0.00 H

ATOM 1098 HD22 LEU 67 83.099 18.225 33.855 1.00 0.00 H

ATOM 1099 HD23 LEU 67 84.458 19.391 33.964 1.00 0.00 H

ATOM 1100 C LEU 67 86.400 17.643 30.213 1.00 0.00 C

ATOM 1101 O LEU 67 85.706 17.911 29.250 1.00 0.00 O

ATOM 1102 N PRO 68 87.714 17.944 30.320 1.00 0.00 N

ATOM 1103 CD PRO 68 88.680 17.579 31.388 1.00 0.00 C

ATOM 1104 HD2 PRO 68 88.936 16.534 31.216 1.00 0.00 H

ATOM 1105 HD3 PRO 68 88.264 17.702 32.388 1.00 0.00 H

ATOM 1106 CG PRO 68 89.806 18.558 31.241 1.00 0.00 C

ATOM 1107 HG2 PRO 68 90.782 18.174 31.537 1.00 0.00 H

ATOM 1108 HG3 PRO 68 89.562 19.495 31.740 1.00 0.00 H

ATOM 1109 CB PRO 68 89.957 18.707 29.728 1.00 0.00 C

ATOM 1110 HB2 PRO 68 90.509 17.843 29.357 1.00 0.00 H

ATOM 1111 HB3 PRO 68 90.493 19.639 29.552 1.00 0.00 H

ATOM 1112 CA PRO 68 88.462 18.660 29.262 1.00 0.00 C

ATOM 1113 HA PRO 68 88.056 19.664 29.389 1.00 0.00 H

ATOM 1114 C PRO 68 88.259 18.265 27.793 1.00 0.00 C

ATOM 1115 O PRO 68 88.307 19.175 26.964 1.00 0.00 O

ATOM 1116 N ALA 69 88.063 16.960 27.573 1.00 0.00 N

ATOM 1117 H ALA 69 87.783 16.318 28.300 1.00 0.00 H

ATOM 1118 CA ALA 69 87.755 16.521 26.188 1.00 0.00 C

ATOM 1119 HA ALA 69 88.491 17.030 25.565 1.00 0.00 H

ATOM 1120 CB ALA 69 87.973 14.948 26.157 1.00 0.00 C

ATOM 1121 HB1 ALA 69 89.000 14.744 26.461 1.00 0.00 H

ATOM 1122 HB2 ALA 69 87.221 14.428 26.751 1.00 0.00 H

ATOM 1123 HB3 ALA 69 87.914 14.473 25.178 1.00 0.00 H

ATOM 1124 C ALA 69 86.367 16.869 25.664 1.00 0.00 C

ATOM 1125 O ALA 69 86.321 17.442 24.627 1.00 0.00 O

ATOM 1126 N LEU 70 85.338 16.896 26.505 1.00 0.00 N

ATOM 1127 H LEU 70 85.430 16.886 27.511 1.00 0.00 H

ATOM 1128 CA LEU 70 83.966 17.406 26.233 1.00 0.00 C

ATOM 1129 HA LEU 70 83.764 17.170 25.188 1.00 0.00 H

ATOM 1130 CB LEU 70 83.115 16.716 27.203 1.00 0.00 C

ATOM 1131 HB2 LEU 70 83.187 15.633 27.110 1.00 0.00 H

ATOM 1132 HB3 LEU 70 83.242 16.984 28.252 1.00 0.00 H

ATOM 1133 CG LEU 70 81.643 16.998 26.925 1.00 0.00 C

ATOM 1134 HG LEU 70 81.339 18.037 26.798 1.00 0.00 H

ATOM 1135 CD1 LEU 70 81.278 16.123 25.609 1.00 0.00 C

ATOM 1136 HD11 LEU 70 81.673 15.107 25.634 1.00 0.00 H

ATOM 1137 HD12 LEU 70 80.191 16.054 25.567 1.00 0.00 H

ATOM 1138 HD13 LEU 70 81.670 16.618 24.721 1.00 0.00 H

ATOM 1139 CD2 LEU 70 80.793 16.389 28.009 1.00 0.00 C

ATOM 1140 HD21 LEU 70 80.492 15.356 27.836 1.00 0.00 H

ATOM 1141 HD22 LEU 70 81.353 16.552 28.930 1.00 0.00 H

ATOM 1142 HD23 LEU 70 79.898 17.008 27.949 1.00 0.00 H

ATOM 1143 C LEU 70 83.823 19.007 26.299 1.00 0.00 C

ATOM 1144 O LEU 70 82.950 19.532 25.674 1.00 0.00 O

ATOM 1145 N ILE 71 84.797 19.688 27.016 1.00 0.00 N

ATOM 1146 H ILE 71 85.256 19.330 27.841 1.00 0.00 H

ATOM 1147 CA ILE 71 85.100 21.157 26.813 1.00 0.00 C

ATOM 1148 HA ILE 71 84.159 21.705 26.857 1.00 0.00 H

ATOM 1149 CB ILE 71 85.918 21.782 28.011 1.00 0.00 C

ATOM 1150 HB ILE 71 86.894 21.297 28.008 1.00 0.00 H

ATOM 1151 CG2 ILE 71 86.250 23.256 27.892 1.00 0.00 C

ATOM 1152 HG21 ILE 71 86.813 23.416 26.973 1.00 0.00 H

ATOM 1153 HG22 ILE 71 85.346 23.847 28.041 1.00 0.00 H

ATOM 1154 HG23 ILE 71 86.878 23.428 28.766 1.00 0.00 H

ATOM 1155 CG1 ILE 71 85.250 21.473 29.373 1.00 0.00 C

ATOM 1156 HG12 ILE 71 84.476 22.220 29.547 1.00 0.00 H

ATOM 1157 HG13 ILE 71 84.710 20.526 29.355 1.00 0.00 H

ATOM 1158 CD1 ILE 71 86.186 21.581 30.549 1.00 0.00 C

ATOM 1159 HD11 ILE 71 85.786 20.978 31.364 1.00 0.00 H

ATOM 1160 HD12 ILE 71 87.170 21.283 30.188 1.00 0.00 H

ATOM 1161 HD13 ILE 71 86.009 22.614 30.851 1.00 0.00 H

ATOM 1162 C ILE 71 85.711 21.528 25.446 1.00 0.00 C

ATOM 1163 O ILE 71 85.194 22.347 24.677 1.00 0.00 O

ATOM 1164 N PHE 72 86.803 20.811 25.016 1.00 0.00 N

ATOM 1165 H PHE 72 87.247 20.158 25.645 1.00 0.00 H

ATOM 1166 CA PHE 72 87.521 21.034 23.769 1.00 0.00 C

ATOM 1167 HA PHE 72 87.791 22.076 23.596 1.00 0.00 H

ATOM 1168 CB PHE 72 88.793 20.148 23.589 1.00 0.00 C

ATOM 1169 HB2 PHE 72 89.367 20.092 24.514 1.00 0.00 H

ATOM 1170 HB3 PHE 72 88.579 19.083 23.507 1.00 0.00 H

ATOM 1171 CG PHE 72 89.750 20.645 22.437 1.00 0.00 C

ATOM 1172 CD1 PHE 72 90.705 21.643 22.587 1.00 0.00 C

ATOM 1173 HD1 PHE 72 90.839 22.057 23.576 1.00 0.00 H

ATOM 1174 CE1 PHE 72 91.471 22.232 21.548 1.00 0.00 C

ATOM 1175 HE1 PHE 72 92.149 23.046 21.756 1.00 0.00 H

ATOM 1176 CZ PHE 72 91.243 21.685 20.248 1.00 0.00 C

ATOM 1177 HZ PHE 72 91.735 22.138 19.400 1.00 0.00 H

ATOM 1178 CE2 PHE 72 90.504 20.519 20.127 1.00 0.00 C

ATOM 1179 HE2 PHE 72 90.445 20.002 19.181 1.00 0.00 H

ATOM 1180 CD2 PHE 72 89.749 20.009 21.202 1.00 0.00 C

ATOM 1181 HD2 PHE 72 89.129 19.125 21.225 1.00 0.00 H

ATOM 1182 C PHE 72 86.605 20.691 22.601 1.00 0.00 C

ATOM 1183 O PHE 72 86.588 21.359 21.574 1.00 0.00 O

ATOM 1184 N TYR 73 85.726 19.667 22.675 1.00 0.00 N

ATOM 1185 H TYR 73 85.825 19.042 23.462 1.00 0.00 H

ATOM 1186 CA TYR 73 84.972 19.141 21.527 1.00 0.00 C

ATOM 1187 HA TYR 73 85.603 18.763 20.723 1.00 0.00 H

ATOM 1188 CB TYR 73 84.209 17.892 22.015 1.00 0.00 C

ATOM 1189 HB2 TYR 73 84.905 17.053 21.978 1.00 0.00 H

ATOM 1190 HB3 TYR 73 83.871 17.928 23.051 1.00 0.00 H

ATOM 1191 CG TYR 73 83.039 17.422 21.157 1.00 0.00 C

ATOM 1192 CD1 TYR 73 83.255 17.186 19.808 1.00 0.00 C

ATOM 1193 HD1 TYR 73 84.205 17.325 19.314 1.00 0.00 H

ATOM 1194 CE1 TYR 73 82.142 16.733 19.009 1.00 0.00 C

ATOM 1195 HE1 TYR 73 82.163 16.537 17.947 1.00 0.00 H

ATOM 1196 CZ TYR 73 80.892 16.327 19.664 1.00 0.00 C

ATOM 1197 OH TYR 73 79.862 15.877 18.897 1.00 0.00 O

ATOM 1198 HH TYR 73 78.975 15.947 19.258 1.00 0.00 H

ATOM 1199 CE2 TYR 73 80.768 16.509 21.045 1.00 0.00 C

ATOM 1200 HE2 TYR 73 79.965 16.027 21.582 1.00 0.00 H

ATOM 1201 CD2 TYR 73 81.798 17.218 21.745 1.00 0.00 C

ATOM 1202 HD2 TYR 73 81.564 17.541 22.748 1.00 0.00 H

ATOM 1203 C TYR 73 83.976 20.144 20.871 1.00 0.00 C

ATOM 1204 O TYR 73 84.012 20.314 19.637 1.00 0.00 O

ATOM 1205 N TYR 74 83.080 20.780 21.625 1.00 0.00 N

ATOM 1206 H TYR 74 83.120 20.596 22.617 1.00 0.00 H

ATOM 1207 CA TYR 74 82.071 21.722 21.134 1.00 0.00 C

ATOM 1208 HA TYR 74 81.494 21.261 20.332 1.00 0.00 H

ATOM 1209 CB TYR 74 81.120 21.948 22.296 1.00 0.00 C

ATOM 1210 HB2 TYR 74 81.718 21.934 23.207 1.00 0.00 H

ATOM 1211 HB3 TYR 74 80.513 22.850 22.215 1.00 0.00 H

ATOM 1212 CG TYR 74 80.168 20.790 22.452 1.00 0.00 C

ATOM 1213 CD1 TYR 74 79.264 20.443 21.435 1.00 0.00 C

ATOM 1214 HD1 TYR 74 79.276 20.829 20.426 1.00 0.00 H

ATOM 1215 CE1 TYR 74 78.267 19.548 21.703 1.00 0.00 C

ATOM 1216 HE1 TYR 74 77.467 19.415 20.991 1.00 0.00 H

ATOM 1217 CZ TYR 74 78.216 18.769 22.866 1.00 0.00 C

ATOM 1218 OH TYR 74 77.216 17.851 23.174 1.00 0.00 O

ATOM 1219 HH TYR 74 77.130 17.662 24.111 1.00 0.00 H

ATOM 1220 CE2 TYR 74 79.268 18.928 23.784 1.00 0.00 C

ATOM 1221 HE2 TYR 74 79.194 18.435 24.742 1.00 0.00 H

ATOM 1222 CD2 TYR 74 80.204 19.978 23.607 1.00 0.00 C

ATOM 1223 HD2 TYR 74 81.107 20.146 24.176 1.00 0.00 H

ATOM 1224 C TYR 74 82.714 23.007 20.617 1.00 0.00 C

ATOM 1225 O TYR 74 82.343 23.467 19.536 1.00 0.00 O

ATOM 1226 N PHE 75 83.760 23.467 21.384 1.00 0.00 N

ATOM 1227 H PHE 75 83.922 22.991 22.259 1.00 0.00 H

ATOM 1228 CA PHE 75 84.613 24.621 21.083 1.00 0.00 C

ATOM 1229 HA PHE 75 83.935 25.461 20.930 1.00 0.00 H

ATOM 1230 CB PHE 75 85.567 24.944 22.236 1.00 0.00 C

ATOM 1231 HB2 PHE 75 85.002 25.231 23.123 1.00 0.00 H

ATOM 1232 HB3 PHE 75 86.069 24.034 22.565 1.00 0.00 H

ATOM 1233 CG PHE 75 86.552 25.987 22.003 1.00 0.00 C

ATOM 1234 CD1 PHE 75 86.069 27.302 22.030 1.00 0.00 C

ATOM 1235 HD1 PHE 75 84.997 27.429 22.052 1.00 0.00 H

ATOM 1236 CE1 PHE 75 86.963 28.359 22.177 1.00 0.00 C

ATOM 1237 HE1 PHE 75 86.593 29.365 22.307 1.00 0.00 H

ATOM 1238 CZ PHE 75 88.363 28.124 22.068 1.00 0.00 C

ATOM 1239 HZ PHE 75 89.091 28.912 22.190 1.00 0.00 H

ATOM 1240 CE2 PHE 75 88.852 26.794 21.797 1.00 0.00 C

ATOM 1241 HE2 PHE 75 89.902 26.612 21.625 1.00 0.00 H

ATOM 1242 CD2 PHE 75 87.913 25.722 21.863 1.00 0.00 C

ATOM 1243 HD2 PHE 75 88.187 24.714 21.587 1.00 0.00 H

ATOM 1244 C PHE 75 85.434 24.382 19.791 1.00 0.00 C

ATOM 1245 O PHE 75 85.337 25.088 18.831 1.00 0.00 O

ATOM 1246 N ASN 76 85.981 23.189 19.548 1.00 0.00 N

ATOM 1247 H ASN 76 86.007 22.577 20.351 1.00 0.00 H

ATOM 1248 CA ASN 76 86.541 22.774 18.214 1.00 0.00 C

ATOM 1249 HA ASN 76 87.243 23.572 17.968 1.00 0.00 H

ATOM 1250 CB ASN 76 87.454 21.489 18.328 1.00 0.00 C

ATOM 1251 HB2 ASN 76 88.180 21.666 19.121 1.00 0.00 H

ATOM 1252 HB3 ASN 76 86.874 20.658 18.731 1.00 0.00 H

ATOM 1253 CG ASN 76 88.074 21.027 16.995 1.00 0.00 C

ATOM 1254 OD1 ASN 76 88.540 21.799 16.203 1.00 0.00 O

ATOM 1255 ND2 ASN 76 88.130 19.727 16.818 1.00 0.00 N

ATOM 1256 HD21 ASN 76 88.226 19.455 15.851 1.00 0.00 H

ATOM 1257 HD22 ASN 76 87.522 19.159 17.391 1.00 0.00 H

ATOM 1258 C ASN 76 85.457 22.620 17.097 1.00 0.00 C

ATOM 1259 O ASN 76 85.573 21.840 16.142 1.00 0.00 O

ATOM 1260 N LYS 77 84.266 23.175 17.258 1.00 0.00 N

ATOM 1261 H LYS 77 84.184 23.859 17.996 1.00 0.00 H

ATOM 1262 CA LYS 77 83.213 23.298 16.272 1.00 0.00 C

ATOM 1263 HA LYS 77 82.457 23.923 16.749 1.00 0.00 H

ATOM 1264 CB LYS 77 83.636 24.018 14.988 1.00 0.00 C

ATOM 1265 HB2 LYS 77 84.319 23.394 14.412 1.00 0.00 H

ATOM 1266 HB3 LYS 77 82.792 24.287 14.353 1.00 0.00 H

ATOM 1267 CG LYS 77 84.445 25.348 15.240 1.00 0.00 C

ATOM 1268 HG2 LYS 77 85.256 25.102 15.926 1.00 0.00 H

ATOM 1269 HG3 LYS 77 84.791 25.663 14.256 1.00 0.00 H

ATOM 1270 CD LYS 77 83.659 26.520 15.850 1.00 0.00 C

ATOM 1271 HD2 LYS 77 82.790 26.747 15.233 1.00 0.00 H

ATOM 1272 HD3 LYS 77 83.158 26.197 16.763 1.00 0.00 H

ATOM 1273 CE LYS 77 84.456 27.753 16.151 1.00 0.00 C

ATOM 1274 HE2 LYS 77 85.169 27.980 15.358 1.00 0.00 H

ATOM 1275 HE3 LYS 77 83.762 28.578 16.307 1.00 0.00 H

ATOM 1276 NZ LYS 77 85.185 27.568 17.471 1.00 0.00 N

ATOM 1277 HZ1 LYS 77 85.408 26.594 17.622 1.00 0.00 H

ATOM 1278 HZ2 LYS 77 86.087 28.014 17.551 1.00 0.00 H

ATOM 1279 HZ3 LYS 77 84.608 27.812 18.263 1.00 0.00 H

ATOM 1280 C LYS 77 82.553 21.983 15.989 1.00 0.00 C

ATOM 1281 O LYS 77 82.476 21.504 14.849 1.00 0.00 O

ATOM 1282 N THR 78 82.166 21.340 17.115 1.00 0.00 N

ATOM 1283 H THR 78 82.201 21.893 17.960 1.00 0.00 H

ATOM 1284 CA THR 78 81.552 20.024 17.273 1.00 0.00 C

ATOM 1285 HA THR 78 81.654 19.711 18.312 1.00 0.00 H

ATOM 1286 CB THR 78 80.009 19.914 17.052 1.00 0.00 C

ATOM 1287 HB THR 78 79.846 19.781 15.982 1.00 0.00 H

ATOM 1288 CG2 THR 78 79.353 18.789 17.824 1.00 0.00 C

ATOM 1289 HG21 THR 78 78.291 19.012 17.929 1.00 0.00 H

ATOM 1290 HG22 THR 78 79.444 17.818 17.338 1.00 0.00 H

ATOM 1291 HG23 THR 78 79.834 18.767 18.802 1.00 0.00 H

ATOM 1292 OG1 THR 78 79.311 21.062 17.538 1.00 0.00 O

ATOM 1293 HG1 THR 78 78.670 21.256 16.850 1.00 0.00 H

ATOM 1294 C THR 78 82.390 18.883 16.661 1.00 0.00 C

ATOM 1295 O THR 78 81.807 17.991 16.013 1.00 0.00 O

ATOM 1296 N ASP 79 83.716 18.853 16.707 1.00 0.00 N

ATOM 1297 H ASP 79 84.199 19.599 17.187 1.00 0.00 H

ATOM 1298 CA ASP 79 84.616 17.831 16.096 1.00 0.00 C

ATOM 1299 HA ASP 79 83.908 17.048 15.822 1.00 0.00 H

ATOM 1300 CB ASP 79 85.285 18.412 14.838 1.00 0.00 C

ATOM 1301 HB2 ASP 79 84.534 18.909 14.224 1.00 0.00 H

ATOM 1302 HB3 ASP 79 85.986 19.172 15.181 1.00 0.00 H

ATOM 1303 CG ASP 79 85.828 17.414 13.851 1.00 0.00 C

ATOM 1304 OD1 ASP 79 86.940 16.876 13.998 1.00 0.00 O

ATOM 1305 OD2 ASP 79 85.086 16.990 12.949 1.00 0.00 O

ATOM 1306 C ASP 79 85.495 17.191 17.204 1.00 0.00 C

ATOM 1307 O ASP 79 86.387 17.811 17.828 1.00 0.00 O

ATOM 1308 N TRP 80 85.206 15.886 17.421 1.00 0.00 N

ATOM 1309 H TRP 80 84.415 15.527 16.906 1.00 0.00 H

ATOM 1310 CA TRP 80 85.927 15.100 18.380 1.00 0.00 C

ATOM 1311 HA TRP 80 86.357 15.790 19.106 1.00 0.00 H

ATOM 1312 CB TRP 80 85.003 14.058 18.964 1.00 0.00 C

ATOM 1313 HB2 TRP 80 84.226 14.666 19.429 1.00 0.00 H

ATOM 1314 HB3 TRP 80 84.572 13.411 18.201 1.00 0.00 H

ATOM 1315 CG TRP 80 85.751 13.194 20.031 1.00 0.00 C

ATOM 1316 CD1 TRP 80 86.428 12.045 19.824 1.00 0.00 C

ATOM 1317 HD1 TRP 80 86.519 11.608 18.840 1.00 0.00 H

ATOM 1318 NE1 TRP 80 87.027 11.631 20.957 1.00 0.00 N

ATOM 1319 HE1 TRP 80 87.439 10.709 21.001 1.00 0.00 H

ATOM 1320 CE2 TRP 80 86.552 12.402 22.061 1.00 0.00 C

ATOM 1321 CZ2 TRP 80 86.596 12.250 23.486 1.00 0.00 C

ATOM 1322 HZ2 TRP 80 87.367 11.570 23.816 1.00 0.00 H

ATOM 1323 CH2 TRP 80 85.885 13.153 24.299 1.00 0.00 C

ATOM 1324 HH2 TRP 80 85.928 13.058 25.374 1.00 0.00 H

ATOM 1325 CZ3 TRP 80 85.179 14.207 23.747 1.00 0.00 C

ATOM 1326 HZ3 TRP 80 84.926 15.031 24.397 1.00 0.00 H

ATOM 1327 CE3 TRP 80 84.989 14.273 22.347 1.00 0.00 C

ATOM 1328 HE3 TRP 80 84.590 15.117 21.804 1.00 0.00 H

ATOM 1329 CD2 TRP 80 85.665 13.334 21.483 1.00 0.00 C

ATOM 1330 C TRP 80 87.226 14.493 17.815 1.00 0.00 C

ATOM 1331 O TRP 80 87.220 13.438 17.204 1.00 0.00 O

ATOM 1332 N ILE 81 88.386 15.109 18.071 1.00 0.00 N

ATOM 1333 H ILE 81 88.295 15.947 18.628 1.00 0.00 H

ATOM 1334 CA ILE 81 89.722 14.796 17.629 1.00 0.00 C

ATOM 1335 HA ILE 81 89.533 14.431 16.619 1.00 0.00 H

ATOM 1336 CB ILE 81 90.614 16.020 17.493 1.00 0.00 C

ATOM 1337 HB ILE 81 91.605 15.627 17.269 1.00 0.00 H

ATOM 1338 CG2 ILE 81 90.339 16.808 16.240 1.00 0.00 C

ATOM 1339 HG21 ILE 81 91.106 17.582 16.224 1.00 0.00 H

ATOM 1340 HG22 ILE 81 90.215 16.147 15.382 1.00 0.00 H

ATOM 1341 HG23 ILE 81 89.412 17.380 16.293 1.00 0.00 H

ATOM 1342 CG1 ILE 81 90.670 16.909 18.735 1.00 0.00 C

ATOM 1343 HG12 ILE 81 90.176 17.873 18.619 1.00 0.00 H

ATOM 1344 HG13 ILE 81 90.336 16.351 19.610 1.00 0.00 H

ATOM 1345 CD1 ILE 81 92.092 17.336 18.962 1.00 0.00 C

ATOM 1346 HD11 ILE 81 92.698 16.433 19.039 1.00 0.00 H

ATOM 1347 HD12 ILE 81 92.464 17.924 18.124 1.00 0.00 H

ATOM 1348 HD13 ILE 81 92.094 18.086 19.753 1.00 0.00 H

ATOM 1349 C ILE 81 90.504 13.671 18.333 1.00 0.00 C

ATOM 1350 O ILE 81 91.457 13.106 17.727 1.00 0.00 O

ATOM 1351 N PHE 82 89.993 13.221 19.475 1.00 0.00 N

ATOM 1352 H PHE 82 89.183 13.620 19.930 1.00 0.00 H

ATOM 1353 CA PHE 82 90.720 12.209 20.298 1.00 0.00 C

ATOM 1354 HA PHE 82 91.766 12.336 20.019 1.00 0.00 H

ATOM 1355 CB PHE 82 90.419 12.422 21.770 1.00 0.00 C

ATOM 1356 HB2 PHE 82 89.357 12.236 21.929 1.00 0.00 H

ATOM 1357 HB3 PHE 82 90.901 11.710 22.441 1.00 0.00 H

ATOM 1358 CG PHE 82 90.745 13.807 22.295 1.00 0.00 C

ATOM 1359 CD1 PHE 82 89.726 14.611 22.763 1.00 0.00 C

ATOM 1360 HD1 PHE 82 88.783 14.089 22.705 1.00 0.00 H

ATOM 1361 CE1 PHE 82 89.688 15.997 22.753 1.00 0.00 C

ATOM 1362 HE1 PHE 82 88.697 16.425 22.711 1.00 0.00 H

ATOM 1363 CZ PHE 82 90.903 16.670 22.567 1.00 0.00 C

ATOM 1364 HZ PHE 82 90.809 17.728 22.367 1.00 0.00 H

ATOM 1365 CE2 PHE 82 91.972 15.931 22.032 1.00 0.00 C

ATOM 1366 HE2 PHE 82 92.882 16.443 21.756 1.00 0.00 H

ATOM 1367 CD2 PHE 82 91.883 14.507 21.838 1.00 0.00 C

ATOM 1368 HD2 PHE 82 92.723 14.003 21.382 1.00 0.00 H

ATOM 1369 C PHE 82 90.382 10.780 19.908 1.00 0.00 C

ATOM 1370 O PHE 82 91.065 9.863 20.308 1.00 0.00 O

ATOM 1371 N GLY 83 89.358 10.629 19.080 1.00 0.00 N

ATOM 1372 H GLY 83 89.100 11.484 18.607 1.00 0.00 H

ATOM 1373 CA GLY 83 89.042 9.305 18.455 1.00 0.00 C

ATOM 1374 HA2 GLY 83 88.942 9.471 17.383 1.00 0.00 H

ATOM 1375 HA3 GLY 83 89.870 8.607 18.578 1.00 0.00 H

ATOM 1376 C GLY 83 87.847 8.537 18.902 1.00 0.00 C

ATOM 1377 O GLY 83 87.228 8.845 19.938 1.00 0.00 O

ATOM 1378 N ASP 84 87.540 7.460 18.199 1.00 0.00 N

ATOM 1379 H ASP 84 88.162 7.398 17.406 1.00 0.00 H

ATOM 1380 CA ASP 84 86.248 6.740 18.198 1.00 0.00 C

ATOM 1381 HA ASP 84 85.463 7.454 17.947 1.00 0.00 H

ATOM 1382 CB ASP 84 86.177 5.724 17.034 1.00 0.00 C

ATOM 1383 HB2 ASP 84 86.217 6.180 16.044 1.00 0.00 H

ATOM 1384 HB3 ASP 84 87.116 5.170 17.027 1.00 0.00 H

ATOM 1385 CG ASP 84 84.851 4.883 17.058 1.00 0.00 C

ATOM 1386 OD1 ASP 84 83.813 5.451 16.718 1.00 0.00 O

ATOM 1387 OD2 ASP 84 84.942 3.650 17.245 1.00 0.00 O

ATOM 1388 C ASP 84 85.951 6.084 19.553 1.00 0.00 C

ATOM 1389 O ASP 84 85.080 6.557 20.286 1.00 0.00 O

ATOM 1390 N ALA 85 86.700 5.052 20.001 1.00 0.00 N

ATOM 1391 H ALA 85 87.491 4.722 19.468 1.00 0.00 H

ATOM 1392 CA ALA 85 86.617 4.439 21.288 1.00 0.00 C

ATOM 1393 HA ALA 85 85.630 4.002 21.438 1.00 0.00 H

ATOM 1394 CB ALA 85 87.646 3.313 21.650 1.00 0.00 C

ATOM 1395 HB1 ALA 85 87.933 2.841 20.710 1.00 0.00 H

ATOM 1396 HB2 ALA 85 88.488 3.729 22.203 1.00 0.00 H

ATOM 1397 HB3 ALA 85 87.142 2.593 22.293 1.00 0.00 H

ATOM 1398 C ALA 85 86.624 5.474 22.436 1.00 0.00 C

ATOM 1399 O ALA 85 86.231 5.169 23.533 1.00 0.00 O

ATOM 1400 N MET 86 87.374 6.613 22.294 1.00 0.00 N

ATOM 1401 H MET 86 87.758 6.824 21.384 1.00 0.00 H

ATOM 1402 CA MET 86 87.671 7.576 23.283 1.00 0.00 C

ATOM 1403 HA MET 86 87.834 6.986 24.185 1.00 0.00 H

ATOM 1404 CB MET 86 88.938 8.325 22.928 1.00 0.00 C

ATOM 1405 HB2 MET 86 88.938 8.706 21.907 1.00 0.00 H

ATOM 1406 HB3 MET 86 88.865 9.256 23.490 1.00 0.00 H

ATOM 1407 CG MET 86 90.330 7.699 23.344 1.00 0.00 C

ATOM 1408 HG2 MET 86 90.331 6.674 22.972 1.00 0.00 H

ATOM 1409 HG3 MET 86 91.109 8.223 22.790 1.00 0.00 H

ATOM 1410 SD MET 86 90.840 7.466 25.111 1.00 0.00 S

ATOM 1411 CE MET 86 90.245 5.729 25.312 1.00 0.00 C

ATOM 1412 HE1 MET 86 90.789 5.360 26.181 1.00 0.00 H

ATOM 1413 HE2 MET 86 89.158 5.641 25.303 1.00 0.00 H

ATOM 1414 HE3 MET 86 90.645 5.138 24.488 1.00 0.00 H

ATOM 1415 C MET 86 86.426 8.482 23.457 1.00 0.00 C

ATOM 1416 O MET 86 85.987 8.466 24.595 1.00 0.00 O

ATOM 1417 N CYX 87 85.793 8.949 22.446 1.00 0.00 N

ATOM 1418 H CYX 87 86.182 8.815 21.524 1.00 0.00 H

ATOM 1419 CA CYX 87 84.416 9.459 22.509 1.00 0.00 C

ATOM 1420 HA CYX 87 84.556 10.303 23.185 1.00 0.00 H

ATOM 1421 CB CYX 87 84.020 9.812 21.078 1.00 0.00 C

ATOM 1422 HB2 CYX 87 84.300 10.843 20.859 1.00 0.00 H

ATOM 1423 HB3 CYX 87 84.395 9.101 20.342 1.00 0.00 H

ATOM 1424 SG CYX 87 82.204 9.918 20.939 1.00 0.00 S

ATOM 1425 C CYX 87 83.481 8.357 23.137 1.00 0.00 C

ATOM 1426 O CYX 87 82.907 8.747 24.176 1.00 0.00 O

ATOM 1427 N LYS 88 83.398 7.135 22.668 1.00 0.00 N

ATOM 1428 H LYS 88 83.801 6.902 21.772 1.00 0.00 H

ATOM 1429 CA LYS 88 82.540 6.147 23.366 1.00 0.00 C

ATOM 1430 HA LYS 88 81.535 6.563 23.429 1.00 0.00 H

ATOM 1431 CB LYS 88 82.510 4.859 22.578 1.00 0.00 C

ATOM 1432 HB2 LYS 88 83.456 4.317 22.581 1.00 0.00 H

ATOM 1433 HB3 LYS 88 81.747 4.217 23.017 1.00 0.00 H

ATOM 1434 CG LYS 88 81.968 5.140 21.049 1.00 0.00 C

ATOM 1435 HG2 LYS 88 80.939 5.499 21.016 1.00 0.00 H

ATOM 1436 HG3 LYS 88 82.547 5.918 20.553 1.00 0.00 H

ATOM 1437 CD LYS 88 82.124 3.901 20.063 1.00 0.00 C

ATOM 1438 HD2 LYS 88 83.193 3.755 19.905 1.00 0.00 H

ATOM 1439 HD3 LYS 88 81.799 2.996 20.577 1.00 0.00 H

ATOM 1440 CE LYS 88 81.285 4.091 18.788 1.00 0.00 C

ATOM 1441 HE2 LYS 88 80.248 3.771 18.884 1.00 0.00 H

ATOM 1442 HE3 LYS 88 81.328 5.174 18.673 1.00 0.00 H

ATOM 1443 NZ LYS 88 81.929 3.313 17.704 1.00 0.00 N

ATOM 1444 HZ1 LYS 88 82.887 3.633 17.699 1.00 0.00 H

ATOM 1445 HZ2 LYS 88 81.883 2.315 17.851 1.00 0.00 H

ATOM 1446 HZ3 LYS 88 81.439 3.511 16.843 1.00 0.00 H

ATOM 1447 C LYS 88 82.908 5.856 24.805 1.00 0.00 C

ATOM 1448 O LYS 88 82.013 5.697 25.617 1.00 0.00 O

ATOM 1449 N LEU 89 84.154 5.983 25.260 1.00 0.00 N

ATOM 1450 H LEU 89 84.894 6.105 24.583 1.00 0.00 H

ATOM 1451 CA LEU 89 84.560 5.861 26.646 1.00 0.00 C

ATOM 1452 HA LEU 89 83.980 5.032 27.050 1.00 0.00 H

ATOM 1453 CB LEU 89 86.054 5.496 26.798 1.00 0.00 C

ATOM 1454 HB2 LEU 89 86.270 4.693 26.093 1.00 0.00 H

ATOM 1455 HB3 LEU 89 86.639 6.340 26.430 1.00 0.00 H

ATOM 1456 CG LEU 89 86.549 5.072 28.211 1.00 0.00 C

ATOM 1457 HG LEU 89 86.142 5.642 29.047 1.00 0.00 H

ATOM 1458 CD1 LEU 89 86.481 3.638 28.516 1.00 0.00 C

ATOM 1459 HD11 LEU 89 86.810 3.484 29.544 1.00 0.00 H

ATOM 1460 HD12 LEU 89 85.434 3.337 28.509 1.00 0.00 H

ATOM 1461 HD13 LEU 89 86.967 3.042 27.743 1.00 0.00 H

ATOM 1462 CD2 LEU 89 88.048 5.257 28.515 1.00 0.00 C

ATOM 1463 HD21 LEU 89 88.669 4.709 27.806 1.00 0.00 H

ATOM 1464 HD22 LEU 89 88.249 6.329 28.523 1.00 0.00 H

ATOM 1465 HD23 LEU 89 88.412 4.891 29.475 1.00 0.00 H

ATOM 1466 C LEU 89 84.267 7.086 27.481 1.00 0.00 C

ATOM 1467 O LEU 89 83.608 6.861 28.504 1.00 0.00 O

ATOM 1468 N GLN 90 84.389 8.335 26.919 1.00 0.00 N

ATOM 1469 H GLN 90 84.691 8.475 25.965 1.00 0.00 H

ATOM 1470 CA GLN 90 83.911 9.520 27.556 1.00 0.00 C

ATOM 1471 HA GLN 90 84.294 9.482 28.575 1.00 0.00 H

ATOM 1472 CB GLN 90 84.346 10.896 26.949 1.00 0.00 C

ATOM 1473 HB2 GLN 90 85.396 10.829 26.665 1.00 0.00 H

ATOM 1474 HB3 GLN 90 83.836 11.138 26.017 1.00 0.00 H

ATOM 1475 CG GLN 90 84.102 11.993 28.039 1.00 0.00 C

ATOM 1476 HG2 GLN 90 84.431 11.804 29.061 1.00 0.00 H

ATOM 1477 HG3 GLN 90 84.742 12.835 27.775 1.00 0.00 H

ATOM 1478 CD GLN 90 82.741 12.576 27.988 1.00 0.00 C

ATOM 1479 OE1 GLN 90 81.951 12.267 27.157 1.00 0.00 O

ATOM 1480 NE2 GLN 90 82.397 13.332 29.004 1.00 0.00 N

ATOM 1481 HE21 GLN 90 81.552 13.878 28.911 1.00 0.00 H

ATOM 1482 HE22 GLN 90 83.103 13.464 29.714 1.00 0.00 H

ATOM 1483 C GLN 90 82.414 9.380 27.745 1.00 0.00 C

ATOM 1484 O GLN 90 81.914 9.723 28.860 1.00 0.00 O

ATOM 1485 N ARG 91 81.605 8.986 26.792 1.00 0.00 N

ATOM 1486 H ARG 91 82.103 8.576 26.015 1.00 0.00 H

ATOM 1487 CA ARG 91 80.171 8.703 26.898 1.00 0.00 C

ATOM 1488 HA ARG 91 79.638 9.566 27.296 1.00 0.00 H

ATOM 1489 CB ARG 91 79.612 8.293 25.565 1.00 0.00 C

ATOM 1490 HB2 ARG 91 80.202 7.454 25.193 1.00 0.00 H

ATOM 1491 HB3 ARG 91 78.572 7.988 25.681 1.00 0.00 H

ATOM 1492 CG ARG 91 79.634 9.402 24.561 1.00 0.00 C

ATOM 1493 HG2 ARG 91 78.964 10.173 24.941 1.00 0.00 H

ATOM 1494 HG3 ARG 91 80.677 9.669 24.387 1.00 0.00 H

ATOM 1495 CD ARG 91 79.026 9.057 23.187 1.00 0.00 C

ATOM 1496 HD2 ARG 91 79.307 9.813 22.454 1.00 0.00 H

ATOM 1497 HD3 ARG 91 79.436 8.089 22.896 1.00 0.00 H

ATOM 1498 NE ARG 91 77.594 8.876 23.211 1.00 0.00 N

ATOM 1499 HE ARG 91 77.203 7.987 23.486 1.00 0.00 H

ATOM 1500 CZ ARG 91 76.632 9.787 23.216 1.00 0.00 C

ATOM 1501 NH1 ARG 91 75.450 9.556 23.714 1.00 0.00 N

ATOM 1502 HH11 ARG 91 74.605 9.708 23.183 1.00 0.00 H

ATOM 1503 HH12 ARG 91 75.501 8.560 23.877 1.00 0.00 H

ATOM 1504 NH2 ARG 91 76.808 10.951 22.683 1.00 0.00 N

ATOM 1505 HH21 ARG 91 77.634 11.116 22.127 1.00 0.00 H

ATOM 1506 HH22 ARG 91 76.005 11.563 22.708 1.00 0.00 H

ATOM 1507 C ARG 91 79.837 7.637 27.902 1.00 0.00 C

ATOM 1508 O ARG 91 78.717 7.711 28.371 1.00 0.00 O

ATOM 1509 N PHE 92 80.653 6.604 28.146 1.00 0.00 N

ATOM 1510 H PHE 92 81.314 6.393 27.412 1.00 0.00 H

ATOM 1511 CA PHE 92 80.664 5.737 29.312 1.00 0.00 C

ATOM 1512 HA PHE 92 79.608 5.487 29.411 1.00 0.00 H

ATOM 1513 CB PHE 92 81.500 4.496 29.070 1.00 0.00 C

ATOM 1514 HB2 PHE 92 80.870 3.797 28.521 1.00 0.00 H

ATOM 1515 HB3 PHE 92 82.338 4.656 28.392 1.00 0.00 H

ATOM 1516 CG PHE 92 81.946 3.742 30.281 1.00 0.00 C

ATOM 1517 CD1 PHE 92 81.088 2.854 30.818 1.00 0.00 C

ATOM 1518 HD1 PHE 92 80.075 2.831 30.445 1.00 0.00 H

ATOM 1519 CE1 PHE 92 81.482 1.970 31.873 1.00 0.00 C

ATOM 1520 HE1 PHE 92 80.815 1.312 32.410 1.00 0.00 H

ATOM 1521 CZ PHE 92 82.866 1.961 32.291 1.00 0.00 C

ATOM 1522 HZ PHE 92 83.262 1.403 33.127 1.00 0.00 H

ATOM 1523 CE2 PHE 92 83.796 2.835 31.643 1.00 0.00 C

ATOM 1524 HE2 PHE 92 84.785 3.028 32.031 1.00 0.00 H

ATOM 1525 CD2 PHE 92 83.354 3.683 30.640 1.00 0.00 C

ATOM 1526 HD2 PHE 92 84.004 4.416 30.186 1.00 0.00 H

ATOM 1527 C PHE 92 80.879 6.567 30.594 1.00 0.00 C

ATOM 1528 O PHE 92 79.980 6.639 31.393 1.00 0.00 O

ATOM 1529 N ILE 93 82.002 7.250 30.766 1.00 0.00 N

ATOM 1530 H ILE 93 82.507 7.463 29.918 1.00 0.00 H

ATOM 1531 CA ILE 93 82.353 8.027 31.971 1.00 0.00 C

ATOM 1532 HA ILE 93 82.420 7.247 32.729 1.00 0.00 H

ATOM 1533 CB ILE 93 83.705 8.760 31.775 1.00 0.00 C

ATOM 1534 HB ILE 93 83.638 9.134 30.754 1.00 0.00 H

ATOM 1535 CG2 ILE 93 84.005 9.897 32.742 1.00 0.00 C

ATOM 1536 HG21 ILE 93 85.076 10.090 32.798 1.00 0.00 H

ATOM 1537 HG22 ILE 93 83.538 10.840 32.454 1.00 0.00 H

ATOM 1538 HG23 ILE 93 83.708 9.635 33.758 1.00 0.00 H

ATOM 1539 CG1 ILE 93 84.854 7.710 31.974 1.00 0.00 C

ATOM 1540 HG12 ILE 93 85.042 7.630 33.045 1.00 0.00 H

ATOM 1541 HG13 ILE 93 84.565 6.688 31.727 1.00 0.00 H

ATOM 1542 CD1 ILE 93 86.128 8.144 31.170 1.00 0.00 C

ATOM 1543 HD11 ILE 93 86.599 8.999 31.655 1.00 0.00 H

ATOM 1544 HD12 ILE 93 86.857 7.335 31.129 1.00 0.00 H

ATOM 1545 HD13 ILE 93 85.940 8.414 30.131 1.00 0.00 H

ATOM 1546 C ILE 93 81.291 9.060 32.340 1.00 0.00 C

ATOM 1547 O ILE 93 80.827 9.079 33.487 1.00 0.00 O

ATOM 1548 N PHE 94 80.661 9.715 31.296 1.00 0.00 N

ATOM 1549 H PHE 94 81.042 9.581 30.370 1.00 0.00 H

ATOM 1550 CA PHE 94 79.443 10.491 31.522 1.00 0.00 C

ATOM 1551 HA PHE 94 79.750 11.324 32.154 1.00 0.00 H

ATOM 1552 CB PHE 94 79.027 11.095 30.200 1.00 0.00 C

ATOM 1553 HB2 PHE 94 79.849 11.648 29.745 1.00 0.00 H

ATOM 1554 HB3 PHE 94 78.762 10.325 29.476 1.00 0.00 H

ATOM 1555 CG PHE 94 77.786 12.020 30.188 1.00 0.00 C

ATOM 1556 CD1 PHE 94 77.937 13.371 29.850 1.00 0.00 C

ATOM 1557 HD1 PHE 94 78.919 13.784 29.675 1.00 0.00 H

ATOM 1558 CE1 PHE 94 76.871 14.289 29.685 1.00 0.00 C

ATOM 1559 HE1 PHE 94 77.117 15.284 29.344 1.00 0.00 H

ATOM 1560 CZ PHE 94 75.527 13.809 29.999 1.00 0.00 C

ATOM 1561 HZ PHE 94 74.744 14.553 29.975 1.00 0.00 H

ATOM 1562 CE2 PHE 94 75.309 12.471 30.373 1.00 0.00 C

ATOM 1563 HE2 PHE 94 74.325 12.025 30.372 1.00 0.00 H

ATOM 1564 CD2 PHE 94 76.451 11.583 30.463 1.00 0.00 C

ATOM 1565 HD2 PHE 94 76.154 10.575 30.712 1.00 0.00 H

ATOM 1566 C PHE 94 78.369 9.599 32.191 1.00 0.00 C

ATOM 1567 O PHE 94 77.750 10.087 33.177 1.00 0.00 O

ATOM 1568 N HID 95 78.016 8.411 31.716 1.00 0.00 N

ATOM 1569 H HID 95 78.417 7.957 30.908 1.00 0.00 H

ATOM 1570 CA HID 95 76.756 7.715 32.242 1.00 0.00 C

ATOM 1571 HA HID 95 76.063 8.495 32.557 1.00 0.00 H

ATOM 1572 CB HID 95 76.152 6.772 31.149 1.00 0.00 C

ATOM 1573 HB2 HID 95 76.959 6.158 30.749 1.00 0.00 H

ATOM 1574 HB3 HID 95 75.413 6.108 31.596 1.00 0.00 H

ATOM 1575 CG HID 95 75.385 7.469 30.067 1.00 0.00 C

ATOM 1576 ND1 HID 95 75.863 7.645 28.809 1.00 0.00 N

ATOM 1577 HD1 HID 95 76.825 7.446 28.576 1.00 0.00 H

ATOM 1578 CE1 HID 95 75.042 8.497 28.137 1.00 0.00 C

ATOM 1579 HE1 HID 95 75.119 8.923 27.147 1.00 0.00 H

ATOM 1580 NE2 HID 95 73.958 8.746 28.930 1.00 0.00 N

ATOM 1581 CD2 HID 95 74.147 8.026 30.067 1.00 0.00 C

ATOM 1582 HD2 HID 95 73.584 8.250 30.961 1.00 0.00 H

ATOM 1583 C HID 95 77.091 6.895 33.506 1.00 0.00 C

ATOM 1584 O HID 95 76.257 6.754 34.362 1.00 0.00 O

ATOM 1585 N VAL 96 78.355 6.533 33.672 1.00 0.00 N

ATOM 1586 H VAL 96 78.935 6.602 32.848 1.00 0.00 H

ATOM 1587 CA VAL 96 78.998 6.084 34.908 1.00 0.00 C

ATOM 1588 HA VAL 96 78.612 5.112 35.217 1.00 0.00 H

ATOM 1589 CB VAL 96 80.547 5.988 34.693 1.00 0.00 C

ATOM 1590 HB VAL 96 80.820 6.659 33.878 1.00 0.00 H

ATOM 1591 CG1 VAL 96 81.323 6.076 35.987 1.00 0.00 C

ATOM 1592 HG11 VAL 96 81.353 7.162 36.081 1.00 0.00 H

ATOM 1593 HG12 VAL 96 80.864 5.743 36.918 1.00 0.00 H

ATOM 1594 HG13 VAL 96 82.339 5.716 35.826 1.00 0.00 H

ATOM 1595 CG2 VAL 96 80.746 4.577 34.202 1.00 0.00 C

ATOM 1596 HG21 VAL 96 81.808 4.364 34.079 1.00 0.00 H

ATOM 1597 HG22 VAL 96 80.392 3.899 34.979 1.00 0.00 H

ATOM 1598 HG23 VAL 96 80.232 4.420 33.254 1.00 0.00 H

ATOM 1599 C VAL 96 78.701 7.209 35.938 1.00 0.00 C

ATOM 1600 O VAL 96 78.145 6.907 37.010 1.00 0.00 O

ATOM 1601 N ASN 97 79.028 8.467 35.652 1.00 0.00 N

ATOM 1602 H ASN 97 79.412 8.714 34.751 1.00 0.00 H

ATOM 1603 CA ASN 97 78.937 9.508 36.642 1.00 0.00 C

ATOM 1604 HA ASN 97 79.335 9.137 37.586 1.00 0.00 H

ATOM 1605 CB ASN 97 79.688 10.669 36.170 1.00 0.00 C

ATOM 1606 HB2 ASN 97 80.679 10.320 35.876 1.00 0.00 H

ATOM 1607 HB3 ASN 97 79.257 10.963 35.213 1.00 0.00 H

ATOM 1608 CG ASN 97 79.443 11.838 37.118 1.00 0.00 C

ATOM 1609 OD1 ASN 97 78.577 12.658 36.990 1.00 0.00 O

ATOM 1610 ND2 ASN 97 80.164 11.819 38.251 1.00 0.00 N

ATOM 1611 HD21 ASN 97 80.701 11.024 38.568 1.00 0.00 H

ATOM 1612 HD22 ASN 97 79.767 12.272 39.062 1.00 0.00 H

ATOM 1613 C ASN 97 77.452 9.769 37.057 1.00 0.00 C

ATOM 1614 O ASN 97 77.080 9.576 38.254 1.00 0.00 O

ATOM 1615 N LEU 98 76.580 10.017 36.045 1.00 0.00 N

ATOM 1616 H LEU 98 76.893 10.046 35.085 1.00 0.00 H

ATOM 1617 CA LEU 98 75.221 10.338 36.189 1.00 0.00 C

ATOM 1618 HA LEU 98 75.300 11.250 36.781 1.00 0.00 H

ATOM 1619 CB LEU 98 74.552 10.583 34.744 1.00 0.00 C

ATOM 1620 HB2 LEU 98 75.154 11.320 34.213 1.00 0.00 H

ATOM 1621 HB3 LEU 98 74.631 9.582 34.319 1.00 0.00 H

ATOM 1622 CG LEU 98 73.190 11.111 34.830 1.00 0.00 C

ATOM 1623 HG LEU 98 72.727 10.799 35.767 1.00 0.00 H

ATOM 1624 CD1 LEU 98 73.093 12.645 34.754 1.00 0.00 C

ATOM 1625 HD11 LEU 98 73.345 13.085 35.719 1.00 0.00 H

ATOM 1626 HD12 LEU 98 73.776 13.142 34.065 1.00 0.00 H

ATOM 1627 HD13 LEU 98 72.115 12.861 34.324 1.00 0.00 H

ATOM 1628 CD2 LEU 98 72.447 10.638 33.563 1.00 0.00 C

ATOM 1629 HD21 LEU 98 72.414 9.551 33.478 1.00 0.00 H

ATOM 1630 HD22 LEU 98 71.397 10.915 33.661 1.00 0.00 H

ATOM 1631 HD23 LEU 98 72.828 11.147 32.678 1.00 0.00 H

ATOM 1632 C LEU 98 74.487 9.296 37.001 1.00 0.00 C

ATOM 1633 O LEU 98 73.630 9.690 37.738 1.00 0.00 O

ATOM 1634 N TYR 99 74.663 7.955 36.748 1.00 0.00 N

ATOM 1635 H TYR 99 75.408 7.684 36.122 1.00 0.00 H

ATOM 1636 CA TYR 99 74.014 6.905 37.577 1.00 0.00 C

ATOM 1637 HA TYR 99 73.007 7.197 37.874 1.00 0.00 H

ATOM 1638 CB TYR 99 73.950 5.533 36.811 1.00 0.00 C

ATOM 1639 HB2 TYR 99 74.932 5.254 36.431 1.00 0.00 H

ATOM 1640 HB3 TYR 99 73.532 4.743 37.434 1.00 0.00 H

ATOM 1641 CG TYR 99 73.072 5.660 35.595 1.00 0.00 C

ATOM 1642 CD1 TYR 99 71.899 6.439 35.641 1.00 0.00 C

ATOM 1643 HD1 TYR 99 71.509 6.814 36.575 1.00 0.00 H

ATOM 1644 CE1 TYR 99 71.213 6.832 34.470 1.00 0.00 C

ATOM 1645 HE1 TYR 99 70.372 7.508 34.428 1.00 0.00 H

ATOM 1646 CZ TYR 99 71.802 6.469 33.213 1.00 0.00 C

ATOM 1647 OH TYR 99 71.317 7.039 32.058 1.00 0.00 O

ATOM 1648 HH TYR 99 71.677 6.566 31.304 1.00 0.00 H

ATOM 1649 CE2 TYR 99 72.904 5.673 33.182 1.00 0.00 C

ATOM 1650 HE2 TYR 99 73.212 5.448 32.172 1.00 0.00 H

ATOM 1651 CD2 TYR 99 73.555 5.269 34.333 1.00 0.00 C

ATOM 1652 HD2 TYR 99 74.421 4.629 34.247 1.00 0.00 H

ATOM 1653 C TYR 99 74.758 6.563 38.850 1.00 0.00 C

ATOM 1654 O TYR 99 74.133 6.386 39.901 1.00 0.00 O

ATOM 1655 N GLY 100 76.071 6.575 38.928 1.00 0.00 N

ATOM 1656 H GLY 100 76.626 6.823 38.121 1.00 0.00 H

ATOM 1657 CA GLY 100 76.842 6.320 40.169 1.00 0.00 C

ATOM 1658 HA2 GLY 100 76.635 5.314 40.534 1.00 0.00 H

ATOM 1659 HA3 GLY 100 77.902 6.216 39.937 1.00 0.00 H

ATOM 1660 C GLY 100 76.559 7.421 41.216 1.00 0.00 C

ATOM 1661 O GLY 100 76.325 6.982 42.317 1.00 0.00 O

ATOM 1662 N SER 101 76.736 8.687 41.054 1.00 0.00 N

ATOM 1663 H SER 101 77.282 8.975 40.255 1.00 0.00 H

ATOM 1664 CA SER 101 76.237 9.801 41.809 1.00 0.00 C

ATOM 1665 HA SER 101 76.957 9.901 42.622 1.00 0.00 H

ATOM 1666 CB SER 101 76.210 11.113 41.067 1.00 0.00 C

ATOM 1667 HB2 SER 101 75.810 11.948 41.643 1.00 0.00 H

ATOM 1668 HB3 SER 101 77.201 11.283 40.647 1.00 0.00 H

ATOM 1669 OG SER 101 75.329 11.069 39.960 1.00 0.00 O

ATOM 1670 HG SER 101 75.763 10.673 39.200 1.00 0.00 H

ATOM 1671 C SER 101 74.960 9.514 42.513 1.00 0.00 C

ATOM 1672 O SER 101 74.973 9.472 43.748 1.00 0.00 O

ATOM 1673 N ILE 102 73.812 9.221 41.890 1.00 0.00 N

ATOM 1674 H ILE 102 73.789 9.156 40.882 1.00 0.00 H

ATOM 1675 CA ILE 102 72.597 8.898 42.620 1.00 0.00 C

ATOM 1676 HA ILE 102 72.314 9.605 43.400 1.00 0.00 H

ATOM 1677 CB ILE 102 71.402 8.934 41.664 1.00 0.00 C

ATOM 1678 HB ILE 102 71.539 9.943 41.275 1.00 0.00 H

ATOM 1679 CG2 ILE 102 71.375 8.030 40.410 1.00 0.00 C

ATOM 1680 HG21 ILE 102 71.119 6.986 40.590 1.00 0.00 H

ATOM 1681 HG22 ILE 102 70.683 8.517 39.723 1.00 0.00 H

ATOM 1682 HG23 ILE 102 72.405 8.116 40.063 1.00 0.00 H

ATOM 1683 CG1 ILE 102 70.104 9.056 42.532 1.00 0.00 C

ATOM 1684 HG12 ILE 102 70.014 8.090 43.028 1.00 0.00 H

ATOM 1685 HG13 ILE 102 70.239 9.856 43.260 1.00 0.00 H

ATOM 1686 CD1 ILE 102 68.950 9.373 41.642 1.00 0.00 C

ATOM 1687 HD11 ILE 102 68.634 8.569 40.978 1.00 0.00 H

ATOM 1688 HD12 ILE 102 68.090 9.615 42.267 1.00 0.00 H

ATOM 1689 HD13 ILE 102 69.151 10.215 40.981 1.00 0.00 H

ATOM 1690 C ILE 102 72.763 7.589 43.340 1.00 0.00 C

ATOM 1691 O ILE 102 72.152 7.404 44.384 1.00 0.00 O

ATOM 1692 N LEU 103 73.370 6.607 42.698 1.00 0.00 N

ATOM 1693 H LEU 103 73.862 6.643 41.817 1.00 0.00 H

ATOM 1694 CA LEU 103 73.256 5.205 43.203 1.00 0.00 C

ATOM 1695 HA LEU 103 72.190 5.035 43.353 1.00 0.00 H

ATOM 1696 CB LEU 103 73.751 4.179 42.262 1.00 0.00 C

ATOM 1697 HB2 LEU 103 74.729 4.535 41.936 1.00 0.00 H

ATOM 1698 HB3 LEU 103 73.863 3.238 42.801 1.00 0.00 H

ATOM 1699 CG LEU 103 72.822 3.752 41.037 1.00 0.00 C

ATOM 1700 HG LEU 103 72.407 4.570 40.448 1.00 0.00 H

ATOM 1701 CD1 LEU 103 73.657 2.847 40.087 1.00 0.00 C

ATOM 1702 HD11 LEU 103 73.232 3.079 39.110 1.00 0.00 H

ATOM 1703 HD12 LEU 103 74.713 3.112 40.127 1.00 0.00 H

ATOM 1704 HD13 LEU 103 73.543 1.764 40.145 1.00 0.00 H

ATOM 1705 CD2 LEU 103 71.616 3.013 41.513 1.00 0.00 C

ATOM 1706 HD21 LEU 103 70.901 2.770 40.727 1.00 0.00 H

ATOM 1707 HD22 LEU 103 71.903 2.022 41.865 1.00 0.00 H

ATOM 1708 HD23 LEU 103 71.015 3.539 42.255 1.00 0.00 H

ATOM 1709 C LEU 103 73.985 5.030 44.584 1.00 0.00 C

ATOM 1710 O LEU 103 73.342 4.716 45.571 1.00 0.00 O

ATOM 1711 N PHE 104 75.295 5.422 44.682 1.00 0.00 N

ATOM 1712 H PHE 104 75.702 5.909 43.896 1.00 0.00 H

ATOM 1713 CA PHE 104 75.959 5.542 45.970 1.00 0.00 C

ATOM 1714 HA PHE 104 76.075 4.527 46.350 1.00 0.00 H

ATOM 1715 CB PHE 104 77.351 6.117 45.930 1.00 0.00 C

ATOM 1716 HB2 PHE 104 77.461 7.066 45.404 1.00 0.00 H

ATOM 1717 HB3 PHE 104 77.664 6.336 46.951 1.00 0.00 H

ATOM 1718 CG PHE 104 78.422 5.187 45.372 1.00 0.00 C

ATOM 1719 CD1 PHE 104 78.905 4.158 46.243 1.00 0.00 C

ATOM 1720 HD1 PHE 104 78.401 3.974 47.180 1.00 0.00 H

ATOM 1721 CE1 PHE 104 79.917 3.294 45.863 1.00 0.00 C

ATOM 1722 HE1 PHE 104 80.236 2.558 46.586 1.00 0.00 H

ATOM 1723 CZ PHE 104 80.586 3.530 44.652 1.00 0.00 C

ATOM 1724 HZ PHE 104 81.437 2.931 44.363 1.00 0.00 H

ATOM 1725 CE2 PHE 104 80.210 4.579 43.768 1.00 0.00 C

ATOM 1726 HE2 PHE 104 80.604 4.749 42.778 1.00 0.00 H

ATOM 1727 CD2 PHE 104 79.087 5.394 44.069 1.00 0.00 C

ATOM 1728 HD2 PHE 104 78.496 5.907 43.325 1.00 0.00 H

ATOM 1729 C PHE 104 75.173 6.381 47.009 1.00 0.00 C

ATOM 1730 O PHE 104 74.903 5.940 48.148 1.00 0.00 O

ATOM 1731 N LEU 105 74.649 7.558 46.633 1.00 0.00 N

ATOM 1732 H LEU 105 74.933 7.889 45.722 1.00 0.00 H

ATOM 1733 CA LEU 105 73.796 8.411 47.425 1.00 0.00 C

ATOM 1734 HA LEU 105 74.298 8.594 48.374 1.00 0.00 H

ATOM 1735 CB LEU 105 73.576 9.809 46.797 1.00 0.00 C

ATOM 1736 HB2 LEU 105 74.521 10.218 46.440 1.00 0.00 H

ATOM 1737 HB3 LEU 105 72.873 9.834 45.964 1.00 0.00 H

ATOM 1738 CG LEU 105 73.011 10.836 47.798 1.00 0.00 C

ATOM 1739 HG LEU 105 71.982 10.504 47.932 1.00 0.00 H

ATOM 1740 CD1 LEU 105 73.854 11.016 49.081 1.00 0.00 C

ATOM 1741 HD11 LEU 105 74.917 10.903 48.866 1.00 0.00 H

ATOM 1742 HD12 LEU 105 73.684 11.899 49.697 1.00 0.00 H

ATOM 1743 HD13 LEU 105 73.424 10.205 49.668 1.00 0.00 H

ATOM 1744 CD2 LEU 105 73.009 12.179 47.051 1.00 0.00 C

ATOM 1745 HD21 LEU 105 72.586 12.927 47.722 1.00 0.00 H

ATOM 1746 HD22 LEU 105 74.007 12.568 46.846 1.00 0.00 H

ATOM 1747 HD23 LEU 105 72.474 11.984 46.122 1.00 0.00 H

ATOM 1748 C LEU 105 72.468 7.781 47.875 1.00 0.00 C

ATOM 1749 O LEU 105 71.969 8.088 48.939 1.00 0.00 O

ATOM 1750 N THR 106 72.127 6.734 47.223 1.00 0.00 N

ATOM 1751 H THR 106 72.532 6.473 46.335 1.00 0.00 H

ATOM 1752 CA THR 106 70.963 5.880 47.620 1.00 0.00 C

ATOM 1753 HA THR 106 70.338 6.566 48.192 1.00 0.00 H

ATOM 1754 CB THR 106 70.165 5.308 46.395 1.00 0.00 C

ATOM 1755 HB THR 106 70.740 4.533 45.888 1.00 0.00 H

ATOM 1756 CG2 THR 106 68.713 4.877 46.755 1.00 0.00 C

ATOM 1757 HG21 THR 106 68.690 4.501 47.778 1.00 0.00 H

ATOM 1758 HG22 THR 106 68.039 5.733 46.769 1.00 0.00 H

ATOM 1759 HG23 THR 106 68.381 4.178 45.986 1.00 0.00 H

ATOM 1760 OG1 THR 106 69.973 6.293 45.450 1.00 0.00 O

ATOM 1761 HG1 THR 106 70.804 6.589 45.071 1.00 0.00 H

ATOM 1762 C THR 106 71.353 4.917 48.729 1.00 0.00 C

ATOM 1763 O THR 106 70.599 4.608 49.606 1.00 0.00 O

ATOM 1764 N CYX 107 72.571 4.408 48.666 1.00 0.00 N

ATOM 1765 H CYX 107 73.243 4.797 48.021 1.00 0.00 H

ATOM 1766 CA CYX 107 73.181 3.594 49.761 1.00 0.00 C

ATOM 1767 HA CYX 107 72.464 2.807 49.996 1.00 0.00 H

ATOM 1768 CB CYX 107 74.530 2.983 49.270 1.00 0.00 C

ATOM 1769 HB2 CYX 107 75.292 3.762 49.250 1.00 0.00 H

ATOM 1770 HB3 CYX 107 74.853 2.365 50.107 1.00 0.00 H

ATOM 1771 SG CYX 107 74.639 1.800 47.844 1.00 0.00 S

ATOM 1772 C CYX 107 73.353 4.533 50.978 1.00 0.00 C

ATOM 1773 O CYX 107 72.869 4.156 52.031 1.00 0.00 O

ATOM 1774 N ILE 108 73.897 5.764 50.783 1.00 0.00 N

ATOM 1775 H ILE 108 74.026 6.122 49.847 1.00 0.00 H

ATOM 1776 CA ILE 108 73.955 6.745 51.904 1.00 0.00 C

ATOM 1777 HA ILE 108 74.569 6.212 52.630 1.00 0.00 H

ATOM 1778 CB ILE 108 74.629 8.063 51.476 1.00 0.00 C

ATOM 1779 HB ILE 108 74.050 8.300 50.583 1.00 0.00 H

ATOM 1780 CG2 ILE 108 74.413 9.198 52.523 1.00 0.00 C

ATOM 1781 HG21 ILE 108 74.940 9.063 53.467 1.00 0.00 H

ATOM 1782 HG22 ILE 108 74.675 10.169 52.101 1.00 0.00 H

ATOM 1783 HG23 ILE 108 73.374 9.205 52.854 1.00 0.00 H

ATOM 1784 CG1 ILE 108 76.074 7.805 51.044 1.00 0.00 C

ATOM 1785 HG12 ILE 108 76.660 7.736 51.960 1.00 0.00 H

ATOM 1786 HG13 ILE 108 76.215 6.896 50.461 1.00 0.00 H

ATOM 1787 CD1 ILE 108 76.823 8.839 50.197 1.00 0.00 C

ATOM 1788 HD11 ILE 108 76.615 8.506 49.180 1.00 0.00 H

ATOM 1789 HD12 ILE 108 76.343 9.816 50.257 1.00 0.00 H

ATOM 1790 HD13 ILE 108 77.881 8.817 50.458 1.00 0.00 H

ATOM 1791 C ILE 108 72.561 6.869 52.492 1.00 0.00 C

ATOM 1792 O ILE 108 72.453 6.696 53.675 1.00 0.00 O

ATOM 1793 N SER 109 71.564 6.979 51.656 1.00 0.00 N

ATOM 1794 H SER 109 71.847 6.872 50.692 1.00 0.00 H

ATOM 1795 CA SER 109 70.215 7.231 52.217 1.00 0.00 C

ATOM 1796 HA SER 109 70.270 8.044 52.941 1.00 0.00 H

ATOM 1797 CB SER 109 69.355 7.752 51.124 1.00 0.00 C

ATOM 1798 HB2 SER 109 69.351 6.992 50.341 1.00 0.00 H

ATOM 1799 HB3 SER 109 68.372 7.979 51.536 1.00 0.00 H

ATOM 1800 OG SER 109 69.963 8.924 50.578 1.00 0.00 O

ATOM 1801 HG SER 109 70.516 8.612 49.858 1.00 0.00 H

ATOM 1802 C SER 109 69.562 6.072 52.928 1.00 0.00 C

ATOM 1803 O SER 109 68.959 6.186 54.083 1.00 0.00 O

ATOM 1804 N ALA 110 69.788 4.875 52.368 1.00 0.00 N

ATOM 1805 H ALA 110 70.256 4.842 51.473 1.00 0.00 H

ATOM 1806 CA ALA 110 69.572 3.634 53.048 1.00 0.00 C

ATOM 1807 HA ALA 110 68.508 3.579 53.280 1.00 0.00 H

ATOM 1808 CB ALA 110 69.836 2.459 52.044 1.00 0.00 C

ATOM 1809 HB1 ALA 110 70.893 2.410 51.782 1.00 0.00 H

ATOM 1810 HB2 ALA 110 69.606 1.490 52.487 1.00 0.00 H

ATOM 1811 HB3 ALA 110 69.210 2.533 51.155 1.00 0.00 H

ATOM 1812 C ALA 110 70.376 3.369 54.300 1.00 0.00 C

ATOM 1813 O ALA 110 69.918 2.615 55.170 1.00 0.00 O

ATOM 1814 N HID 111 71.555 4.015 54.479 1.00 0.00 N

ATOM 1815 H HID 111 71.974 4.526 53.716 1.00 0.00 H

ATOM 1816 CA HID 111 72.438 3.874 55.608 1.00 0.00 C

ATOM 1817 HA HID 111 72.353 2.897 56.084 1.00 0.00 H

ATOM 1818 CB HID 111 73.911 3.909 55.104 1.00 0.00 C

ATOM 1819 HB2 HID 111 74.040 4.353 54.117 1.00 0.00 H

ATOM 1820 HB3 HID 111 74.317 4.545 55.891 1.00 0.00 H

ATOM 1821 CG HID 111 74.579 2.566 55.071 1.00 0.00 C

ATOM 1822 ND1 HID 111 75.748 2.364 55.820 1.00 0.00 N

ATOM 1823 HD1 HID 111 76.053 2.890 56.627 1.00 0.00 H

ATOM 1824 CE1 HID 111 76.254 1.131 55.445 1.00 0.00 C

ATOM 1825 HE1 HID 111 77.099 0.702 55.962 1.00 0.00 H

ATOM 1826 NE2 HID 111 75.479 0.626 54.487 1.00 0.00 N

ATOM 1827 CD2 HID 111 74.433 1.523 54.169 1.00 0.00 C

ATOM 1828 HD2 HID 111 73.701 1.557 53.377 1.00 0.00 H

ATOM 1829 C HID 111 72.170 4.928 56.723 1.00 0.00 C

ATOM 1830 O HID 111 71.940 4.460 57.813 1.00 0.00 O

ATOM 1831 N ARG 112 71.822 6.177 56.366 1.00 0.00 N

ATOM 1832 H ARG 112 72.045 6.507 55.438 1.00 0.00 H

ATOM 1833 CA ARG 112 71.189 7.111 57.340 1.00 0.00 C

ATOM 1834 HA ARG 112 71.813 7.256 58.222 1.00 0.00 H

ATOM 1835 CB ARG 112 71.026 8.507 56.546 1.00 0.00 C

ATOM 1836 HB2 ARG 112 70.421 8.236 55.681 1.00 0.00 H

ATOM 1837 HB3 ARG 112 70.368 9.078 57.201 1.00 0.00 H

ATOM 1838 CG ARG 112 72.188 9.289 56.198 1.00 0.00 C

ATOM 1839 HG2 ARG 112 72.956 8.635 55.786 1.00 0.00 H

ATOM 1840 HG3 ARG 112 72.034 10.049 55.432 1.00 0.00 H

ATOM 1841 CD ARG 112 72.716 10.060 57.432 1.00 0.00 C

ATOM 1842 HD2 ARG 112 71.862 10.676 57.715 1.00 0.00 H

ATOM 1843 HD3 ARG 112 72.889 9.379 58.265 1.00 0.00 H

ATOM 1844 NE ARG 112 73.925 10.842 57.228 1.00 0.00 N

ATOM 1845 HE ARG 112 73.963 11.506 56.467 1.00 0.00 H

ATOM 1846 CZ ARG 112 75.001 10.778 58.012 1.00 0.00 C

ATOM 1847 NH1 ARG 112 75.292 9.726 58.814 1.00 0.00 N

ATOM 1848 HH11 ARG 112 74.738 8.883 58.756 1.00 0.00 H

ATOM 1849 HH12 ARG 112 75.832 10.015 59.617 1.00 0.00 H

ATOM 1850 NH2 ARG 112 75.927 11.644 58.122 1.00 0.00 N

ATOM 1851 HH21 ARG 112 76.831 11.407 58.505 1.00 0.00 H

ATOM 1852 HH22 ARG 112 75.917 12.445 57.507 1.00 0.00 H

ATOM 1853 C ARG 112 69.933 6.507 57.885 1.00 0.00 C

ATOM 1854 O ARG 112 69.689 6.696 59.047 1.00 0.00 O

ATOM 1855 N TYR 113 69.132 5.796 57.053 1.00 0.00 N

ATOM 1856 H TYR 113 69.387 5.790 56.076 1.00 0.00 H

ATOM 1857 CA TYR 113 67.850 5.184 57.474 1.00 0.00 C

ATOM 1858 HA TYR 113 67.158 5.850 57.990 1.00 0.00 H

ATOM 1859 CB TYR 113 67.048 4.652 56.229 1.00 0.00 C

ATOM 1860 HB2 TYR 113 66.490 5.495 55.821 1.00 0.00 H

ATOM 1861 HB3 TYR 113 67.749 4.288 55.478 1.00 0.00 H

ATOM 1862 CG TYR 113 66.034 3.515 56.436 1.00 0.00 C

ATOM 1863 CD1 TYR 113 66.397 2.115 56.401 1.00 0.00 C

ATOM 1864 HD1 TYR 113 67.351 1.790 56.012 1.00 0.00 H

ATOM 1865 CE1 TYR 113 65.489 1.099 56.908 1.00 0.00 C

ATOM 1866 HE1 TYR 113 65.749 0.052 56.963 1.00 0.00 H

ATOM 1867 CZ TYR 113 64.237 1.606 57.370 1.00 0.00 C

ATOM 1868 OH TYR 113 63.192 0.762 57.732 1.00 0.00 O

ATOM 1869 HH TYR 113 63.480 -0.154 57.728 1.00 0.00 H

ATOM 1870 CE2 TYR 113 63.847 2.946 57.313 1.00 0.00 C

ATOM 1871 HE2 TYR 113 62.815 3.201 57.508 1.00 0.00 H

ATOM 1872 CD2 TYR 113 64.742 3.892 56.811 1.00 0.00 C

ATOM 1873 HD2 TYR 113 64.477 4.914 56.582 1.00 0.00 H

ATOM 1874 C TYR 113 68.125 4.071 58.526 1.00 0.00 C

ATOM 1875 O TYR 113 67.717 4.253 59.671 1.00 0.00 O

ATOM 1876 N SER 114 68.948 3.015 58.195 1.00 0.00 N

ATOM 1877 H SER 114 69.258 2.901 57.241 1.00 0.00 H

ATOM 1878 CA SER 114 69.399 2.049 59.175 1.00 0.00 C

ATOM 1879 HA SER 114 68.566 1.350 59.251 1.00 0.00 H

ATOM 1880 CB SER 114 70.475 1.241 58.516 1.00 0.00 C

ATOM 1881 HB2 SER 114 70.845 1.831 57.678 1.00 0.00 H

ATOM 1882 HB3 SER 114 71.285 0.941 59.181 1.00 0.00 H

ATOM 1883 OG SER 114 69.934 0.007 58.094 1.00 0.00 O

ATOM 1884 HG SER 114 70.404 -0.357 57.339 1.00 0.00 H

ATOM 1885 C SER 114 69.977 2.684 60.445 1.00 0.00 C

ATOM 1886 O SER 114 69.711 2.150 61.560 1.00 0.00 O

ATOM 1887 N GLY 115 70.715 3.793 60.277 1.00 0.00 N

ATOM 1888 H GLY 115 70.732 4.192 59.350 1.00 0.00 H

ATOM 1889 CA GLY 115 71.329 4.652 61.357 1.00 0.00 C

ATOM 1890 HA2 GLY 115 71.894 4.047 62.066 1.00 0.00 H

ATOM 1891 HA3 GLY 115 72.002 5.330 60.832 1.00 0.00 H

ATOM 1892 C GLY 115 70.361 5.407 62.338 1.00 0.00 C

ATOM 1893 O GLY 115 70.547 5.237 63.543 1.00 0.00 O

ATOM 1894 N VAL 116 69.196 5.872 61.864 1.00 0.00 N

ATOM 1895 H VAL 116 69.247 6.117 60.886 1.00 0.00 H

ATOM 1896 CA VAL 116 68.145 6.369 62.639 1.00 0.00 C

ATOM 1897 HA VAL 116 68.523 6.845 63.543 1.00 0.00 H

ATOM 1898 CB VAL 116 67.201 7.390 61.934 1.00 0.00 C

ATOM 1899 HB VAL 116 66.724 6.888 61.092 1.00 0.00 H

ATOM 1900 CG1 VAL 116 66.023 7.832 62.855 1.00 0.00 C

ATOM 1901 HG11 VAL 116 65.232 7.094 62.728 1.00 0.00 H

ATOM 1902 HG12 VAL 116 66.464 8.023 63.834 1.00 0.00 H

ATOM 1903 HG13 VAL 116 65.587 8.803 62.621 1.00 0.00 H

ATOM 1904 CG2 VAL 116 68.089 8.586 61.587 1.00 0.00 C

ATOM 1905 HG21 VAL 116 68.864 8.370 60.852 1.00 0.00 H

ATOM 1906 HG22 VAL 116 67.450 9.383 61.208 1.00 0.00 H

ATOM 1907 HG23 VAL 116 68.606 8.853 62.509 1.00 0.00 H

ATOM 1908 C VAL 116 67.288 5.215 63.259 1.00 0.00 C

ATOM 1909 O VAL 116 66.866 5.343 64.370 1.00 0.00 O

ATOM 1910 N VAL 117 66.912 4.251 62.392 1.00 0.00 N

ATOM 1911 H VAL 117 67.305 4.281 61.462 1.00 0.00 H

ATOM 1912 CA VAL 117 65.893 3.208 62.728 1.00 0.00 C

ATOM 1913 HA VAL 117 65.061 3.634 63.288 1.00 0.00 H

ATOM 1914 CB VAL 117 65.261 2.814 61.364 1.00 0.00 C

ATOM 1915 HB VAL 117 66.043 2.733 60.610 1.00 0.00 H

ATOM 1916 CG1 VAL 117 64.498 1.449 61.299 1.00 0.00 C

ATOM 1917 HG11 VAL 117 64.085 1.251 60.310 1.00 0.00 H

ATOM 1918 HG12 VAL 117 65.145 0.698 61.753 1.00 0.00 H

ATOM 1919 HG13 VAL 117 63.651 1.406 61.983 1.00 0.00 H

ATOM 1920 CG2 VAL 117 64.404 3.956 60.896 1.00 0.00 C

ATOM 1921 HG21 VAL 117 64.999 4.845 60.687 1.00 0.00 H

ATOM 1922 HG22 VAL 117 63.907 3.675 59.967 1.00 0.00 H

ATOM 1923 HG23 VAL 117 63.630 4.255 61.603 1.00 0.00 H

ATOM 1924 C VAL 117 66.351 2.040 63.596 1.00 0.00 C

ATOM 1925 O VAL 117 65.771 1.782 64.631 1.00 0.00 O

ATOM 1926 N TYR 118 67.443 1.426 63.198 1.00 0.00 N

ATOM 1927 H TYR 118 67.737 1.602 62.248 1.00 0.00 H

ATOM 1928 CA TYR 118 68.190 0.371 63.902 1.00 0.00 C

ATOM 1929 HA TYR 118 67.826 0.198 64.914 1.00 0.00 H

ATOM 1930 CB TYR 118 67.881 -1.003 63.240 1.00 0.00 C

ATOM 1931 HB2 TYR 118 68.216 -0.844 62.215 1.00 0.00 H

ATOM 1932 HB3 TYR 118 68.588 -1.700 63.691 1.00 0.00 H

ATOM 1933 CG TYR 118 66.486 -1.624 63.373 1.00 0.00 C

ATOM 1934 CD1 TYR 118 65.680 -1.746 62.267 1.00 0.00 C

ATOM 1935 HD1 TYR 118 66.116 -1.542 61.300 1.00 0.00 H

ATOM 1936 CE1 TYR 118 64.404 -2.403 62.387 1.00 0.00 C

ATOM 1937 HE1 TYR 118 63.659 -2.356 61.607 1.00 0.00 H

ATOM 1938 CZ TYR 118 64.064 -3.017 63.589 1.00 0.00 C

ATOM 1939 OH TYR 118 62.750 -3.436 63.787 1.00 0.00 O

ATOM 1940 HH TYR 118 62.182 -3.515 63.016 1.00 0.00 H

ATOM 1941 CE2 TYR 118 64.862 -2.739 64.714 1.00 0.00 C

ATOM 1942 HE2 TYR 118 64.492 -3.086 65.668 1.00 0.00 H

ATOM 1943 CD2 TYR 118 66.102 -2.046 64.633 1.00 0.00 C

ATOM 1944 HD2 TYR 118 66.695 -1.861 65.516 1.00 0.00 H

ATOM 1945 C TYR 118 69.673 0.719 64.116 1.00 0.00 C

ATOM 1946 O TYR 118 70.464 0.104 63.401 1.00 0.00 O

ATOM 1947 N PRO 119 70.086 1.522 65.129 1.00 0.00 N

ATOM 1948 CD PRO 119 69.199 2.414 65.835 1.00 0.00 C

ATOM 1949 HD2 PRO 119 68.707 1.916 66.671 1.00 0.00 H

ATOM 1950 HD3 PRO 119 68.419 2.875 65.228 1.00 0.00 H

ATOM 1951 CG PRO 119 70.095 3.517 66.426 1.00 0.00 C

ATOM 1952 HG2 PRO 119 69.935 3.622 67.499 1.00 0.00 H

ATOM 1953 HG3 PRO 119 69.949 4.405 65.810 1.00 0.00 H

ATOM 1954 CB PRO 119 71.494 3.035 66.158 1.00 0.00 C

ATOM 1955 HB2 PRO 119 71.996 2.845 67.107 1.00 0.00 H

ATOM 1956 HB3 PRO 119 72.000 3.822 65.599 1.00 0.00 H

ATOM 1957 CA PRO 119 71.532 1.902 65.201 1.00 0.00 C

ATOM 1958 HA PRO 119 72.009 2.185 64.263 1.00 0.00 H

ATOM 1959 C PRO 119 72.485 0.766 65.751 1.00 0.00 C

ATOM 1960 O PRO 119 73.717 0.821 65.531 1.00 0.00 O

ATOM 1961 N LEU 120 71.976 -0.250 66.471 1.00 0.00 N

ATOM 1962 H LEU 120 71.010 -0.126 66.742 1.00 0.00 H

ATOM 1963 CA LEU 120 72.735 -1.394 67.099 1.00 0.00 C

ATOM 1964 HA LEU 120 73.802 -1.233 66.949 1.00 0.00 H

ATOM 1965 CB LEU 120 72.430 -1.516 68.582 1.00 0.00 C

ATOM 1966 HB2 LEU 120 71.355 -1.614 68.726 1.00 0.00 H

ATOM 1967 HB3 LEU 120 72.792 -2.500 68.879 1.00 0.00 H

ATOM 1968 CG LEU 120 72.944 -0.576 69.612 1.00 0.00 C

ATOM 1969 HG LEU 120 74.022 -0.481 69.483 1.00 0.00 H

ATOM 1970 CD1 LEU 120 72.409 0.862 69.451 1.00 0.00 C

ATOM 1971 HD11 LEU 120 72.888 1.230 68.544 1.00 0.00 H

ATOM 1972 HD12 LEU 120 71.324 0.761 69.429 1.00 0.00 H

ATOM 1973 HD13 LEU 120 72.757 1.386 70.342 1.00 0.00 H

ATOM 1974 CD2 LEU 120 72.574 -0.971 70.994 1.00 0.00 C

ATOM 1975 HD21 LEU 120 73.027 -0.312 71.734 1.00 0.00 H

ATOM 1976 HD22 LEU 120 71.486 -0.928 71.051 1.00 0.00 H

ATOM 1977 HD23 LEU 120 72.865 -1.986 71.268 1.00 0.00 H

ATOM 1978 C LEU 120 72.352 -2.674 66.435 1.00 0.00 C

ATOM 1979 O LEU 120 73.250 -3.529 66.282 1.00 0.00 O

ATOM 1980 N LYS 121 71.169 -2.817 65.861 1.00 0.00 N

ATOM 1981 H LYS 121 70.465 -2.143 66.126 1.00 0.00 H

ATOM 1982 CA LYS 121 70.730 -3.809 64.927 1.00 0.00 C

ATOM 1983 HA LYS 121 71.313 -4.671 65.252 1.00 0.00 H

ATOM 1984 CB LYS 121 69.270 -4.225 65.071 1.00 0.00 C

ATOM 1985 HB2 LYS 121 68.694 -3.371 65.425 1.00 0.00 H

ATOM 1986 HB3 LYS 121 68.906 -4.594 64.112 1.00 0.00 H

ATOM 1987 CG LYS 121 69.017 -5.448 65.992 1.00 0.00 C

ATOM 1988 HG2 LYS 121 69.706 -6.207 65.622 1.00 0.00 H

ATOM 1989 HG3 LYS 121 69.224 -5.021 66.973 1.00 0.00 H

ATOM 1990 CD LYS 121 67.555 -5.941 65.967 1.00 0.00 C

ATOM 1991 HD2 LYS 121 66.920 -5.163 66.390 1.00 0.00 H

ATOM 1992 HD3 LYS 121 67.258 -6.303 64.983 1.00 0.00 H

ATOM 1993 CE LYS 121 67.496 -7.223 66.806 1.00 0.00 C

ATOM 1994 HE2 LYS 121 67.857 -7.978 66.106 1.00 0.00 H

ATOM 1995 HE3 LYS 121 68.185 -7.245 67.651 1.00 0.00 H

ATOM 1996 NZ LYS 121 66.130 -7.507 67.359 1.00 0.00 N

ATOM 1997 HZ1 LYS 121 65.717 -6.711 67.823 1.00 0.00 H

ATOM 1998 HZ2 LYS 121 65.440 -7.603 66.627 1.00 0.00 H

ATOM 1999 HZ3 LYS 121 66.033 -8.311 67.963 1.00 0.00 H

ATOM 2000 C LYS 121 71.374 -3.612 63.501 1.00 0.00 C

ATOM 2001 O LYS 121 72.546 -3.950 63.351 1.00 0.00 O

ATOM 2002 N SER 122 70.649 -2.961 62.583 1.00 0.00 N

ATOM 2003 H SER 122 69.712 -2.627 62.758 1.00 0.00 H

ATOM 2004 CA SER 122 71.084 -2.843 61.206 1.00 0.00 C

ATOM 2005 HA SER 122 71.240 -3.792 60.692 1.00 0.00 H

ATOM 2006 CB SER 122 70.027 -2.143 60.370 1.00 0.00 C

ATOM 2007 HB2 SER 122 69.882 -1.124 60.730 1.00 0.00 H

ATOM 2008 HB3 SER 122 70.278 -2.024 59.316 1.00 0.00 H

ATOM 2009 OG SER 122 68.831 -2.829 60.551 1.00 0.00 O

ATOM 2010 HG SER 122 68.256 -2.787 59.783 1.00 0.00 H

ATOM 2011 C SER 122 72.386 -2.049 61.062 1.00 0.00 C

ATOM 2012 O SER 122 73.170 -2.497 60.245 1.00 0.00 O

ATOM 2013 N LEU 123 72.684 -0.974 61.854 1.00 0.00 N

ATOM 2014 H LEU 123 71.994 -0.527 62.441 1.00 0.00 H

ATOM 2015 CA LEU 123 73.933 -0.258 61.671 1.00 0.00 C

ATOM 2016 HA LEU 123 74.284 -0.389 60.647 1.00 0.00 H

ATOM 2017 CB LEU 123 73.666 1.222 62.057 1.00 0.00 C

ATOM 2018 HB2 LEU 123 72.674 1.529 61.725 1.00 0.00 H

ATOM 2019 HB3 LEU 123 73.710 1.338 63.140 1.00 0.00 H

ATOM 2020 CG LEU 123 74.651 2.062 61.216 1.00 0.00 C

ATOM 2021 HG LEU 123 75.609 1.699 61.585 1.00 0.00 H

ATOM 2022 CD1 LEU 123 74.588 1.914 59.659 1.00 0.00 C

ATOM 2023 HD11 LEU 123 73.580 1.697 59.307 1.00 0.00 H

ATOM 2024 HD12 LEU 123 74.757 2.948 59.359 1.00 0.00 H

ATOM 2025 HD13 LEU 123 75.418 1.303 59.304 1.00 0.00 H

ATOM 2026 CD2 LEU 123 74.545 3.512 61.723 1.00 0.00 C

ATOM 2027 HD21 LEU 123 74.124 4.107 60.913 1.00 0.00 H

ATOM 2028 HD22 LEU 123 74.120 3.590 62.724 1.00 0.00 H

ATOM 2029 HD23 LEU 123 75.546 3.942 61.719 1.00 0.00 H

ATOM 2030 C LEU 123 75.103 -0.939 62.415 1.00 0.00 C

ATOM 2031 O LEU 123 76.231 -0.933 61.911 1.00 0.00 O

ATOM 2032 N GLY 124 74.897 -1.573 63.530 1.00 0.00 N

ATOM 2033 H GLY 124 73.971 -1.441 63.910 1.00 0.00 H

ATOM 2034 CA GLY 124 75.866 -2.488 64.095 1.00 0.00 C

ATOM 2035 HA2 GLY 124 76.799 -1.942 64.236 1.00 0.00 H

ATOM 2036 HA3 GLY 124 75.512 -2.908 65.036 1.00 0.00 H

ATOM 2037 C GLY 124 76.189 -3.747 63.309 1.00 0.00 C

ATOM 2038 O GLY 124 77.261 -4.267 63.549 1.00 0.00 O

ATOM 2039 N ARG 125 75.382 -4.168 62.445 1.00 0.00 N

ATOM 2040 H ARG 125 74.428 -3.842 62.493 1.00 0.00 H

ATOM 2041 CA ARG 125 75.667 -5.200 61.432 1.00 0.00 C

ATOM 2042 HA ARG 125 76.345 -5.917 61.896 1.00 0.00 H

ATOM 2043 CB ARG 125 74.457 -5.988 61.086 1.00 0.00 C

ATOM 2044 HB2 ARG 125 73.736 -5.277 60.684 1.00 0.00 H

ATOM 2045 HB3 ARG 125 74.704 -6.603 60.221 1.00 0.00 H

ATOM 2046 CG ARG 125 73.743 -6.734 62.253 1.00 0.00 C

ATOM 2047 HG2 ARG 125 74.299 -6.704 63.190 1.00 0.00 H

ATOM 2048 HG3 ARG 125 72.805 -6.221 62.464 1.00 0.00 H

ATOM 2049 CD ARG 125 73.379 -8.176 61.948 1.00 0.00 C

ATOM 2050 HD2 ARG 125 73.455 -8.798 62.840 1.00 0.00 H

ATOM 2051 HD3 ARG 125 72.344 -8.108 61.615 1.00 0.00 H

ATOM 2052 NE ARG 125 74.083 -8.776 60.850 1.00 0.00 N

ATOM 2053 HE ARG 125 74.735 -8.203 60.333 1.00 0.00 H

ATOM 2054 CZ ARG 125 73.937 -10.047 60.484 1.00 0.00 C

ATOM 2055 NH1 ARG 125 74.884 -10.704 59.898 1.00 0.00 N

ATOM 2056 HH11 ARG 125 74.741 -11.631 59.522 1.00 0.00 H

ATOM 2057 HH12 ARG 125 75.640 -10.101 59.605 1.00 0.00 H

ATOM 2058 NH2 ARG 125 72.842 -10.668 60.826 1.00 0.00 N

ATOM 2059 HH21 ARG 125 72.504 -11.488 60.342 1.00 0.00 H

ATOM 2060 HH22 ARG 125 72.188 -10.061 61.300 1.00 0.00 H

ATOM 2061 C ARG 125 76.399 -4.575 60.214 1.00 0.00 C

ATOM 2062 O ARG 125 77.263 -5.254 59.665 1.00 0.00 O

ATOM 2063 N LEU 126 75.956 -3.374 59.870 1.00 0.00 N

ATOM 2064 H LEU 126 75.129 -3.062 60.360 1.00 0.00 H

ATOM 2065 CA LEU 126 76.466 -2.645 58.653 1.00 0.00 C

ATOM 2066 HA LEU 126 76.996 -3.391 58.062 1.00 0.00 H

ATOM 2067 CB LEU 126 75.263 -2.065 57.876 1.00 0.00 C

ATOM 2068 HB2 LEU 126 74.659 -1.481 58.570 1.00 0.00 H

ATOM 2069 HB3 LEU 126 75.670 -1.474 57.055 1.00 0.00 H

ATOM 2070 CG LEU 126 74.344 -3.024 57.147 1.00 0.00 C

ATOM 2071 HG LEU 126 73.830 -3.560 57.945 1.00 0.00 H

ATOM 2072 CD1 LEU 126 73.333 -2.120 56.396 1.00 0.00 C

ATOM 2073 HD11 LEU 126 73.886 -1.314 55.914 1.00 0.00 H

ATOM 2074 HD12 LEU 126 72.858 -2.677 55.589 1.00 0.00 H

ATOM 2075 HD13 LEU 126 72.657 -1.758 57.170 1.00 0.00 H

ATOM 2076 CD2 LEU 126 75.196 -3.935 56.139 1.00 0.00 C

ATOM 2077 HD21 LEU 126 75.813 -3.351 55.457 1.00 0.00 H

ATOM 2078 HD22 LEU 126 75.779 -4.758 56.554 1.00 0.00 H

ATOM 2079 HD23 LEU 126 74.494 -4.424 55.465 1.00 0.00 H

ATOM 2080 C LEU 126 77.614 -1.658 58.978 1.00 0.00 C

ATOM 2081 O LEU 126 77.331 -0.434 58.721 1.00 0.00 O

ATOM 2082 N LYS 127 78.701 -2.213 59.502 1.00 0.00 N

ATOM 2083 H LYS 127 78.544 -3.084 59.989 1.00 0.00 H

ATOM 2084 CA LYS 127 79.924 -1.449 59.680 1.00 0.00 C

ATOM 2085 HA LYS 127 79.555 -0.500 60.071 1.00 0.00 H

ATOM 2086 CB LYS 127 80.758 -2.142 60.825 1.00 0.00 C

ATOM 2087 HB2 LYS 127 81.088 -3.149 60.569 1.00 0.00 H

ATOM 2088 HB3 LYS 127 81.616 -1.486 60.967 1.00 0.00 H

ATOM 2089 CG LYS 127 79.998 -2.154 62.120 1.00 0.00 C

ATOM 2090 HG2 LYS 127 79.762 -1.116 62.354 1.00 0.00 H

ATOM 2091 HG3 LYS 127 79.074 -2.731 62.096 1.00 0.00 H

ATOM 2092 CD LYS 127 80.912 -2.498 63.286 1.00 0.00 C

ATOM 2093 HD2 LYS 127 81.716 -3.147 62.939 1.00 0.00 H

ATOM 2094 HD3 LYS 127 81.352 -1.566 63.641 1.00 0.00 H

ATOM 2095 CE LYS 127 80.212 -3.254 64.367 1.00 0.00 C

ATOM 2096 HE2 LYS 127 79.254 -2.772 64.562 1.00 0.00 H

ATOM 2097 HE3 LYS 127 79.947 -4.224 63.947 1.00 0.00 H

ATOM 2098 NZ LYS 127 81.014 -3.360 65.638 1.00 0.00 N

ATOM 2099 HZ1 LYS 127 81.263 -2.481 66.068 1.00 0.00 H

ATOM 2100 HZ2 LYS 127 80.483 -3.806 66.372 1.00 0.00 H

ATOM 2101 HZ3 LYS 127 81.877 -3.878 65.554 1.00 0.00 H

ATOM 2102 C LYS 127 80.758 -1.174 58.422 1.00 0.00 C

ATOM 2103 O LYS 127 80.437 -1.661 57.338 1.00 0.00 O

ATOM 2104 N LYS 128 81.868 -0.413 58.575 1.00 0.00 N

ATOM 2105 H LYS 128 82.173 -0.270 59.527 1.00 0.00 H

ATOM 2106 CA LYS 128 82.753 -0.084 57.412 1.00 0.00 C

ATOM 2107 HA LYS 128 82.175 0.532 56.724 1.00 0.00 H

ATOM 2108 CB LYS 128 84.000 0.764 57.736 1.00 0.00 C

ATOM 2109 HB2 LYS 128 84.632 0.974 56.873 1.00 0.00 H

ATOM 2110 HB3 LYS 128 83.725 1.793 57.968 1.00 0.00 H

ATOM 2111 CG LYS 128 84.808 0.347 59.023 1.00 0.00 C

ATOM 2112 HG2 LYS 128 84.126 0.623 59.826 1.00 0.00 H

ATOM 2113 HG3 LYS 128 84.933 -0.733 58.937 1.00 0.00 H

ATOM 2114 CD LYS 128 86.089 1.112 59.104 1.00 0.00 C

ATOM 2115 HD2 LYS 128 86.631 0.961 58.171 1.00 0.00 H

ATOM 2116 HD3 LYS 128 85.756 2.150 59.117 1.00 0.00 H

ATOM 2117 CE LYS 128 86.698 0.769 60.419 1.00 0.00 C

ATOM 2118 HE2 LYS 128 86.339 -0.185 60.804 1.00 0.00 H

ATOM 2119 HE3 LYS 128 87.758 0.552 60.284 1.00 0.00 H

ATOM 2120 NZ LYS 128 86.416 1.854 61.421 1.00 0.00 N

ATOM 2121 HZ1 LYS 128 86.717 2.790 61.188 1.00 0.00 H

ATOM 2122 HZ2 LYS 128 85.409 1.882 61.493 1.00 0.00 H

ATOM 2123 HZ3 LYS 128 86.798 1.518 62.294 1.00 0.00 H

ATOM 2124 C LYS 128 83.175 -1.213 56.427 1.00 0.00 C

ATOM 2125 O LYS 128 83.294 -1.004 55.217 1.00 0.00 O

ATOM 2126 N LYS 129 83.269 -2.395 56.911 1.00 0.00 N

ATOM 2127 H LYS 129 83.291 -2.400 57.921 1.00 0.00 H

ATOM 2128 CA LYS 129 83.583 -3.593 56.127 1.00 0.00 C

ATOM 2129 HA LYS 129 84.306 -3.300 55.366 1.00 0.00 H

ATOM 2130 CB LYS 129 84.299 -4.652 56.954 1.00 0.00 C

ATOM 2131 HB2 LYS 129 83.767 -4.879 57.878 1.00 0.00 H

ATOM 2132 HB3 LYS 129 84.263 -5.619 56.453 1.00 0.00 H

ATOM 2133 CG LYS 129 85.746 -4.212 57.378 1.00 0.00 C

ATOM 2134 HG2 LYS 129 85.799 -3.126 57.448 1.00 0.00 H

ATOM 2135 HG3 LYS 129 85.933 -4.594 58.382 1.00 0.00 H

ATOM 2136 CD LYS 129 86.803 -4.657 56.288 1.00 0.00 C

ATOM 2137 HD2 LYS 129 86.661 -5.727 56.137 1.00 0.00 H

ATOM 2138 HD3 LYS 129 86.703 -4.099 55.357 1.00 0.00 H

ATOM 2139 CE LYS 129 88.183 -4.371 56.824 1.00 0.00 C

ATOM 2140 HE2 LYS 129 88.678 -3.901 55.974 1.00 0.00 H

ATOM 2141 HE3 LYS 129 88.106 -3.617 57.607 1.00 0.00 H

ATOM 2142 NZ LYS 129 88.856 -5.544 57.256 1.00 0.00 N

ATOM 2143 HZ1 LYS 129 89.844 -5.398 57.107 1.00 0.00 H

ATOM 2144 HZ2 LYS 129 88.556 -5.899 58.153 1.00 0.00 H

ATOM 2145 HZ3 LYS 129 88.761 -6.286 56.577 1.00 0.00 H

ATOM 2146 C LYS 129 82.438 -4.132 55.314 1.00 0.00 C

ATOM 2147 O LYS 129 82.671 -4.358 54.105 1.00 0.00 O

ATOM 2148 N ASN 130 81.225 -4.104 55.886 1.00 0.00 N

ATOM 2149 H ASN 130 81.043 -3.890 56.856 1.00 0.00 H

ATOM 2150 CA ASN 130 80.047 -4.394 55.040 1.00 0.00 C

ATOM 2151 HA ASN 130 80.284 -5.300 54.482 1.00 0.00 H

ATOM 2152 CB ASN 130 78.827 -4.651 55.973 1.00 0.00 C

ATOM 2153 HB2 ASN 130 78.572 -3.629 56.255 1.00 0.00 H

ATOM 2154 HB3 ASN 130 77.979 -5.054 55.419 1.00 0.00 H

ATOM 2155 CG ASN 130 79.114 -5.572 57.084 1.00 0.00 C

ATOM 2156 OD1 ASN 130 79.768 -5.331 58.075 1.00 0.00 O

ATOM 2157 ND2 ASN 130 78.489 -6.727 56.860 1.00 0.00 N

ATOM 2158 HD21 ASN 130 78.035 -7.032 56.011 1.00 0.00 H

ATOM 2159 HD22 ASN 130 78.973 -7.372 57.468 1.00 0.00 H

ATOM 2160 C ASN 130 79.790 -3.344 53.954 1.00 0.00 C

ATOM 2161 O ASN 130 79.525 -3.624 52.823 1.00 0.00 O

ATOM 2162 N ALA 131 79.854 -1.993 54.369 1.00 0.00 N

ATOM 2163 H ALA 131 80.342 -1.904 55.249 1.00 0.00 H

ATOM 2164 CA ALA 131 79.716 -0.905 53.465 1.00 0.00 C

ATOM 2165 HA ALA 131 78.706 -0.865 53.055 1.00 0.00 H

ATOM 2166 CB ALA 131 79.920 0.419 54.275 1.00 0.00 C

ATOM 2167 HB1 ALA 131 79.697 1.317 53.699 1.00 0.00 H

ATOM 2168 HB2 ALA 131 79.340 0.422 55.198 1.00 0.00 H

ATOM 2169 HB3 ALA 131 80.937 0.428 54.665 1.00 0.00 H

ATOM 2170 C ALA 131 80.733 -0.919 52.365 1.00 0.00 C

ATOM 2171 O ALA 131 80.596 -0.392 51.292 1.00 0.00 O

ATOM 2172 N ILE 132 81.982 -1.265 52.644 1.00 0.00 N

ATOM 2173 H ILE 132 82.368 -1.337 53.574 1.00 0.00 H

ATOM 2174 CA ILE 132 82.930 -1.442 51.499 1.00 0.00 C

ATOM 2175 HA ILE 132 82.853 -0.509 50.942 1.00 0.00 H

ATOM 2176 CB ILE 132 84.443 -1.307 51.913 1.00 0.00 C

ATOM 2177 HB ILE 132 84.443 -0.536 52.683 1.00 0.00 H

ATOM 2178 CG2 ILE 132 85.102 -2.597 52.358 1.00 0.00 C

ATOM 2179 HG21 ILE 132 84.718 -2.860 53.343 1.00 0.00 H

ATOM 2180 HG22 ILE 132 85.049 -3.411 51.635 1.00 0.00 H

ATOM 2181 HG23 ILE 132 86.156 -2.443 52.590 1.00 0.00 H

ATOM 2182 CG1 ILE 132 85.377 -0.627 50.903 1.00 0.00 C

ATOM 2183 HG12 ILE 132 84.971 0.350 50.639 1.00 0.00 H

ATOM 2184 HG13 ILE 132 86.323 -0.352 51.369 1.00 0.00 H

ATOM 2185 CD1 ILE 132 85.662 -1.405 49.659 1.00 0.00 C

ATOM 2186 HD11 ILE 132 84.792 -1.499 49.010 1.00 0.00 H

ATOM 2187 HD12 ILE 132 86.493 -0.857 49.215 1.00 0.00 H

ATOM 2188 HD13 ILE 132 86.175 -2.357 49.797 1.00 0.00 H

ATOM 2189 C ILE 132 82.627 -2.628 50.525 1.00 0.00 C

ATOM 2190 O ILE 132 82.832 -2.432 49.320 1.00 0.00 O

ATOM 2191 N CYX 133 82.192 -3.823 50.969 1.00 0.00 N

ATOM 2192 H CYX 133 82.453 -4.089 51.908 1.00 0.00 H

ATOM 2193 CA CYX 133 81.688 -4.891 50.049 1.00 0.00 C

ATOM 2194 HA CYX 133 82.538 -5.065 49.390 1.00 0.00 H

ATOM 2195 CB CYX 133 81.368 -6.075 50.940 1.00 0.00 C

ATOM 2196 HB2 CYX 133 82.077 -6.136 51.766 1.00 0.00 H

ATOM 2197 HB3 CYX 133 80.301 -5.985 51.146 1.00 0.00 H

ATOM 2198 SG CYX 133 81.282 -7.750 50.150 1.00 0.00 S

ATOM 2199 C CYX 133 80.562 -4.477 49.117 1.00 0.00 C

ATOM 2200 O CYX 133 80.678 -4.637 47.892 1.00 0.00 O

ATOM 2201 N ILE 134 79.650 -3.695 49.663 1.00 0.00 N

ATOM 2202 H ILE 134 79.763 -3.385 50.618 1.00 0.00 H

ATOM 2203 CA ILE 134 78.553 -3.003 48.948 1.00 0.00 C

ATOM 2204 HA ILE 134 78.018 -3.787 48.413 1.00 0.00 H

ATOM 2205 CB ILE 134 77.629 -2.269 49.975 1.00 0.00 C

ATOM 2206 HB ILE 134 78.110 -1.530 50.615 1.00 0.00 H

ATOM 2207 CG2 ILE 134 76.629 -1.401 49.187 1.00 0.00 C

ATOM 2208 HG21 ILE 134 76.076 -2.058 48.516 1.00 0.00 H

ATOM 2209 HG22 ILE 134 75.988 -0.789 49.822 1.00 0.00 H

ATOM 2210 HG23 ILE 134 77.179 -0.621 48.661 1.00 0.00 H

ATOM 2211 CG1 ILE 134 76.945 -3.267 50.866 1.00 0.00 C

ATOM 2212 HG12 ILE 134 76.273 -3.793 50.188 1.00 0.00 H

ATOM 2213 HG13 ILE 134 77.683 -4.030 51.115 1.00 0.00 H

ATOM 2214 CD1 ILE 134 76.244 -2.869 52.192 1.00 0.00 C

ATOM 2215 HD11 ILE 134 76.992 -2.744 52.975 1.00 0.00 H

ATOM 2216 HD12 ILE 134 75.713 -1.926 52.059 1.00 0.00 H

ATOM 2217 HD13 ILE 134 75.421 -3.476 52.569 1.00 0.00 H

ATOM 2218 C ILE 134 79.136 -2.027 47.906 1.00 0.00 C

ATOM 2219 O ILE 134 78.821 -2.115 46.759 1.00 0.00 O

ATOM 2220 N SER 135 80.013 -1.156 48.395 1.00 0.00 N

ATOM 2221 H SER 135 80.310 -1.173 49.360 1.00 0.00 H

ATOM 2222 CA SER 135 80.723 -0.124 47.540 1.00 0.00 C

ATOM 2223 HA SER 135 79.913 0.574 47.329 1.00 0.00 H

ATOM 2224 CB SER 135 81.880 0.623 48.162 1.00 0.00 C

ATOM 2225 HB2 SER 135 82.687 -0.046 48.462 1.00 0.00 H

ATOM 2226 HB3 SER 135 82.264 1.424 47.530 1.00 0.00 H

ATOM 2227 OG SER 135 81.532 1.303 49.311 1.00 0.00 O

ATOM 2228 HG SER 135 81.510 0.705 50.062 1.00 0.00 H

ATOM 2229 C SER 135 81.247 -0.713 46.223 1.00 0.00 C

ATOM 2230 O SER 135 81.102 -0.211 45.117 1.00 0.00 O

ATOM 2231 N VAL 136 82.127 -1.701 46.406 1.00 0.00 N

ATOM 2232 H VAL 136 82.382 -2.011 47.333 1.00 0.00 H

ATOM 2233 CA VAL 136 82.615 -2.439 45.252 1.00 0.00 C

ATOM 2234 HA VAL 136 83.154 -1.714 44.642 1.00 0.00 H

ATOM 2235 CB VAL 136 83.754 -3.337 45.570 1.00 0.00 C

ATOM 2236 HB VAL 136 84.114 -3.930 44.729 1.00 0.00 H

ATOM 2237 CG1 VAL 136 84.913 -2.490 46.130 1.00 0.00 C

ATOM 2238 HG11 VAL 136 84.576 -1.651 46.738 1.00 0.00 H

ATOM 2239 HG12 VAL 136 85.557 -3.096 46.767 1.00 0.00 H

ATOM 2240 HG13 VAL 136 85.415 -1.852 45.402 1.00 0.00 H

ATOM 2241 CG2 VAL 136 83.394 -4.430 46.600 1.00 0.00 C

ATOM 2242 HG21 VAL 136 82.580 -4.993 46.142 1.00 0.00 H

ATOM 2243 HG22 VAL 136 84.164 -5.199 46.674 1.00 0.00 H

ATOM 2244 HG23 VAL 136 83.182 -3.908 47.533 1.00 0.00 H

ATOM 2245 C VAL 136 81.634 -3.190 44.298 1.00 0.00 C

ATOM 2246 O VAL 136 81.830 -3.089 43.103 1.00 0.00 O

ATOM 2247 N LEU 137 80.587 -3.748 44.913 1.00 0.00 N

ATOM 2248 H LEU 137 80.619 -3.701 45.922 1.00 0.00 H

ATOM 2249 CA LEU 137 79.411 -4.401 44.176 1.00 0.00 C

ATOM 2250 HA LEU 137 79.884 -5.231 43.651 1.00 0.00 H

ATOM 2251 CB LEU 137 78.577 -5.023 45.338 1.00 0.00 C

ATOM 2252 HB2 LEU 137 79.179 -5.241 46.221 1.00 0.00 H

ATOM 2253 HB3 LEU 137 77.801 -4.299 45.585 1.00 0.00 H

ATOM 2254 CG LEU 137 77.857 -6.359 45.040 1.00 0.00 C

ATOM 2255 HG LEU 137 78.612 -6.983 44.562 1.00 0.00 H

ATOM 2256 CD1 LEU 137 77.470 -7.086 46.267 1.00 0.00 C

ATOM 2257 HD11 LEU 137 76.654 -6.586 46.789 1.00 0.00 H

ATOM 2258 HD12 LEU 137 77.324 -8.128 45.982 1.00 0.00 H

ATOM 2259 HD13 LEU 137 78.386 -7.240 46.838 1.00 0.00 H

ATOM 2260 CD2 LEU 137 76.546 -6.160 44.204 1.00 0.00 C

ATOM 2261 HD21 LEU 137 76.786 -5.965 43.159 1.00 0.00 H

ATOM 2262 HD22 LEU 137 76.041 -7.125 44.176 1.00 0.00 H

ATOM 2263 HD23 LEU 137 75.837 -5.444 44.620 1.00 0.00 H

ATOM 2264 C LEU 137 78.802 -3.315 43.349 1.00 0.00 C

ATOM 2265 O LEU 137 78.609 -3.584 42.148 1.00 0.00 O

ATOM 2266 N VAL 138 78.587 -2.087 43.910 1.00 0.00 N

ATOM 2267 H VAL 138 78.783 -1.852 44.873 1.00 0.00 H

ATOM 2268 CA VAL 138 78.182 -0.907 43.085 1.00 0.00 C

ATOM 2269 HA VAL 138 77.240 -1.200 42.621 1.00 0.00 H

ATOM 2270 CB VAL 138 77.855 0.313 43.972 1.00 0.00 C

ATOM 2271 HB VAL 138 78.753 0.808 44.341 1.00 0.00 H

ATOM 2272 CG1 VAL 138 77.147 1.380 43.136 1.00 0.00 C

ATOM 2273 HG11 VAL 138 77.960 1.902 42.631 1.00 0.00 H

ATOM 2274 HG12 VAL 138 76.332 0.998 42.521 1.00 0.00 H

ATOM 2275 HG13 VAL 138 76.700 2.120 43.799 1.00 0.00 H

ATOM 2276 CG2 VAL 138 76.972 0.009 45.209 1.00 0.00 C

ATOM 2277 HG21 VAL 138 76.595 -1.014 45.233 1.00 0.00 H

ATOM 2278 HG22 VAL 138 77.651 0.261 46.023 1.00 0.00 H

ATOM 2279 HG23 VAL 138 76.093 0.652 45.257 1.00 0.00 H

ATOM 2280 C VAL 138 79.130 -0.589 42.011 1.00 0.00 C

ATOM 2281 O VAL 138 78.670 -0.199 40.905 1.00 0.00 O

ATOM 2282 N TRP 139 80.497 -0.659 42.147 1.00 0.00 N

ATOM 2283 H TRP 139 80.839 -0.928 43.059 1.00 0.00 H

ATOM 2284 CA TRP 139 81.509 -0.369 41.093 1.00 0.00 C

ATOM 2285 HA TRP 139 81.171 0.577 40.669 1.00 0.00 H

ATOM 2286 CB TRP 139 82.887 -0.277 41.757 1.00 0.00 C

ATOM 2287 HB2 TRP 139 83.012 -0.901 42.642 1.00 0.00 H

ATOM 2288 HB3 TRP 139 83.623 -0.672 41.057 1.00 0.00 H

ATOM 2289 CG TRP 139 83.252 1.146 42.083 1.00 0.00 C

ATOM 2290 CD1 TRP 139 83.644 1.519 43.263 1.00 0.00 C

ATOM 2291 HD1 TRP 139 83.724 0.955 44.181 1.00 0.00 H

ATOM 2292 NE1 TRP 139 83.730 2.885 43.263 1.00 0.00 N

ATOM 2293 HE1 TRP 139 83.912 3.454 44.078 1.00 0.00 H

ATOM 2294 CE2 TRP 139 83.832 3.373 41.947 1.00 0.00 C

ATOM 2295 CZ2 TRP 139 84.140 4.659 41.379 1.00 0.00 C

ATOM 2296 HZ2 TRP 139 84.093 5.468 42.093 1.00 0.00 H

ATOM 2297 CH2 TRP 139 83.841 4.821 40.022 1.00 0.00 C

ATOM 2298 HH2 TRP 139 83.687 5.753 39.499 1.00 0.00 H

ATOM 2299 CZ3 TRP 139 83.524 3.688 39.192 1.00 0.00 C

ATOM 2300 HZ3 TRP 139 83.429 3.859 38.130 1.00 0.00 H

ATOM 2301 CE3 TRP 139 83.393 2.428 39.784 1.00 0.00 C

ATOM 2302 HE3 TRP 139 83.272 1.541 39.179 1.00 0.00 H

ATOM 2303 CD2 TRP 139 83.449 2.240 41.192 1.00 0.00 C

ATOM 2304 C TRP 139 81.433 -1.413 39.953 1.00 0.00 C

ATOM 2305 O TRP 139 81.205 -1.038 38.807 1.00 0.00 O

ATOM 2306 N LEU 140 81.470 -2.714 40.333 1.00 0.00 N

ATOM 2307 H LEU 140 81.363 -2.991 41.298 1.00 0.00 H

ATOM 2308 CA LEU 140 81.177 -3.773 39.325 1.00 0.00 C

ATOM 2309 HA LEU 140 81.984 -3.896 38.602 1.00 0.00 H

ATOM 2310 CB LEU 140 81.005 -5.094 40.113 1.00 0.00 C

ATOM 2311 HB2 LEU 140 81.748 -5.086 40.911 1.00 0.00 H

ATOM 2312 HB3 LEU 140 80.020 -5.247 40.552 1.00 0.00 H

ATOM 2313 CG LEU 140 81.297 -6.294 39.317 1.00 0.00 C

ATOM 2314 HG LEU 140 80.619 -6.413 38.472 1.00 0.00 H

ATOM 2315 CD1 LEU 140 82.808 -6.262 38.767 1.00 0.00 C

ATOM 2316 HD11 LEU 140 83.438 -5.400 38.987 1.00 0.00 H

ATOM 2317 HD12 LEU 140 83.373 -7.079 39.217 1.00 0.00 H

ATOM 2318 HD13 LEU 140 82.643 -6.470 37.710 1.00 0.00 H

ATOM 2319 CD2 LEU 140 81.069 -7.632 40.058 1.00 0.00 C

ATOM 2320 HD21 LEU 140 81.351 -8.516 39.487 1.00 0.00 H

ATOM 2321 HD22 LEU 140 81.394 -7.520 41.092 1.00 0.00 H

ATOM 2322 HD23 LEU 140 80.036 -7.876 40.308 1.00 0.00 H

ATOM 2323 C LEU 140 79.882 -3.498 38.467 1.00 0.00 C

ATOM 2324 O LEU 140 79.894 -3.489 37.196 1.00 0.00 O

ATOM 2325 N ILE 141 78.771 -3.154 39.120 1.00 0.00 N

ATOM 2326 H ILE 141 78.894 -3.018 40.113 1.00 0.00 H

ATOM 2327 CA ILE 141 77.449 -2.718 38.550 1.00 0.00 C

ATOM 2328 HA ILE 141 77.279 -3.515 37.826 1.00 0.00 H

ATOM 2329 CB ILE 141 76.421 -2.742 39.660 1.00 0.00 C

ATOM 2330 HB ILE 141 76.940 -2.287 40.503 1.00 0.00 H

ATOM 2331 CG2 ILE 141 75.152 -1.810 39.598 1.00 0.00 C

ATOM 2332 HG21 ILE 141 74.405 -2.118 38.866 1.00 0.00 H

ATOM 2333 HG22 ILE 141 74.661 -1.710 40.566 1.00 0.00 H

ATOM 2334 HG23 ILE 141 75.499 -0.817 39.314 1.00 0.00 H

ATOM 2335 CG1 ILE 141 76.084 -4.140 40.208 1.00 0.00 C

ATOM 2336 HG12 ILE 141 77.017 -4.699 40.129 1.00 0.00 H

ATOM 2337 HG13 ILE 141 75.690 -3.963 41.209 1.00 0.00 H

ATOM 2338 CD1 ILE 141 75.013 -4.803 39.212 1.00 0.00 C

ATOM 2339 HD11 ILE 141 74.576 -4.080 38.522 1.00 0.00 H

ATOM 2340 HD12 ILE 141 75.533 -5.382 38.449 1.00 0.00 H

ATOM 2341 HD13 ILE 141 74.193 -5.285 39.744 1.00 0.00 H

ATOM 2342 C ILE 141 77.394 -1.395 37.723 1.00 0.00 C

ATOM 2343 O ILE 141 77.006 -1.548 36.544 1.00 0.00 O

ATOM 2344 N VAL 142 78.045 -0.307 38.152 1.00 0.00 N

ATOM 2345 H VAL 142 78.312 -0.265 39.125 1.00 0.00 H

ATOM 2346 CA VAL 142 78.176 0.885 37.284 1.00 0.00 C

ATOM 2347 HA VAL 142 77.153 1.173 37.039 1.00 0.00 H

ATOM 2348 CB VAL 142 78.717 2.158 38.056 1.00 0.00 C

ATOM 2349 HB VAL 142 78.781 1.884 39.110 1.00 0.00 H

ATOM 2350 CG1 VAL 142 80.189 2.446 37.825 1.00 0.00 C

ATOM 2351 HG11 VAL 142 80.265 2.786 36.792 1.00 0.00 H

ATOM 2352 HG12 VAL 142 80.552 3.235 38.484 1.00 0.00 H

ATOM 2353 HG13 VAL 142 80.759 1.545 38.048 1.00 0.00 H

ATOM 2354 CG2 VAL 142 77.923 3.447 37.811 1.00 0.00 C

ATOM 2355 HG21 VAL 142 78.449 4.175 38.429 1.00 0.00 H

ATOM 2356 HG22 VAL 142 77.891 3.692 36.749 1.00 0.00 H

ATOM 2357 HG23 VAL 142 76.866 3.284 38.019 1.00 0.00 H

ATOM 2358 C VAL 142 78.855 0.596 35.964 1.00 0.00 C

ATOM 2359 O VAL 142 78.498 1.191 35.003 1.00 0.00 O

ATOM 2360 N VAL 143 79.833 -0.340 35.999 1.00 0.00 N

ATOM 2361 H VAL 143 80.027 -0.775 36.890 1.00 0.00 H

ATOM 2362 CA VAL 143 80.580 -0.938 34.841 1.00 0.00 C

ATOM 2363 HA VAL 143 80.778 -0.084 34.194 1.00 0.00 H

ATOM 2364 CB VAL 143 81.922 -1.501 35.269 1.00 0.00 C

ATOM 2365 HB VAL 143 81.792 -2.330 35.965 1.00 0.00 H

ATOM 2366 CG1 VAL 143 82.651 -1.885 34.068 1.00 0.00 C

ATOM 2367 HG11 VAL 143 82.338 -1.257 33.234 1.00 0.00 H

ATOM 2368 HG12 VAL 143 83.716 -1.736 34.245 1.00 0.00 H

ATOM 2369 HG13 VAL 143 82.397 -2.899 33.762 1.00 0.00 H

ATOM 2370 CG2 VAL 143 82.577 -0.263 35.963 1.00 0.00 C

ATOM 2371 HG21 VAL 143 82.211 -0.053 36.968 1.00 0.00 H

ATOM 2372 HG22 VAL 143 83.610 -0.479 36.235 1.00 0.00 H

ATOM 2373 HG23 VAL 143 82.528 0.694 35.443 1.00 0.00 H

ATOM 2374 C VAL 143 79.712 -1.854 33.961 1.00 0.00 C

ATOM 2375 O VAL 143 79.333 -1.506 32.847 1.00 0.00 O

ATOM 2376 N VAL 144 79.223 -3.005 34.484 1.00 0.00 N

ATOM 2377 H VAL 144 79.266 -3.098 35.489 1.00 0.00 H

ATOM 2378 CA VAL 144 78.549 -4.016 33.726 1.00 0.00 C

ATOM 2379 HA VAL 144 79.156 -4.184 32.836 1.00 0.00 H

ATOM 2380 CB VAL 144 78.513 -5.421 34.439 1.00 0.00 C

ATOM 2381 HB VAL 144 77.977 -6.141 33.820 1.00 0.00 H

ATOM 2382 CG1 VAL 144 79.899 -5.921 34.748 1.00 0.00 C

ATOM 2383 HG11 VAL 144 80.527 -6.102 33.875 1.00 0.00 H

ATOM 2384 HG12 VAL 144 80.423 -5.194 35.368 1.00 0.00 H

ATOM 2385 HG13 VAL 144 79.806 -6.906 35.205 1.00 0.00 H

ATOM 2386 CG2 VAL 144 77.721 -5.516 35.767 1.00 0.00 C

ATOM 2387 HG21 VAL 144 76.960 -6.296 35.749 1.00 0.00 H

ATOM 2388 HG22 VAL 144 78.395 -5.583 36.621 1.00 0.00 H

ATOM 2389 HG23 VAL 144 77.119 -4.641 36.011 1.00 0.00 H

ATOM 2390 C VAL 144 77.164 -3.628 33.218 1.00 0.00 C

ATOM 2391 O VAL 144 76.756 -3.955 32.098 1.00 0.00 O

ATOM 2392 N ALA 145 76.362 -2.813 33.923 1.00 0.00 N

ATOM 2393 H ALA 145 76.733 -2.449 34.790 1.00 0.00 H

ATOM 2394 CA ALA 145 75.007 -2.490 33.508 1.00 0.00 C

ATOM 2395 HA ALA 145 74.361 -3.358 33.373 1.00 0.00 H

ATOM 2396 CB ALA 145 74.352 -1.725 34.652 1.00 0.00 C

ATOM 2397 HB1 ALA 145 74.185 -2.369 35.515 1.00 0.00 H

ATOM 2398 HB2 ALA 145 75.017 -0.896 34.895 1.00 0.00 H

ATOM 2399 HB3 ALA 145 73.379 -1.293 34.418 1.00 0.00 H

ATOM 2400 C ALA 145 75.040 -1.750 32.123 1.00 0.00 C

ATOM 2401 O ALA 145 74.142 -1.889 31.387 1.00 0.00 O

ATOM 2402 N ILE 146 76.094 -0.971 31.779 1.00 0.00 N

ATOM 2403 H ILE 146 76.776 -0.782 32.499 1.00 0.00 H

ATOM 2404 CA ILE 146 76.028 0.114 30.793 1.00 0.00 C

ATOM 2405 HA ILE 146 75.073 0.080 30.270 1.00 0.00 H

ATOM 2406 CB ILE 146 75.927 1.542 31.365 1.00 0.00 C

ATOM 2407 HB ILE 146 75.903 2.270 30.554 1.00 0.00 H

ATOM 2408 CG2 ILE 146 74.577 1.696 32.064 1.00 0.00 C

ATOM 2409 HG21 ILE 146 74.358 2.764 32.029 1.00 0.00 H

ATOM 2410 HG22 ILE 146 73.825 1.230 31.428 1.00 0.00 H

ATOM 2411 HG23 ILE 146 74.578 1.389 33.109 1.00 0.00 H

ATOM 2412 CG1 ILE 146 77.206 1.786 32.264 1.00 0.00 C

ATOM 2413 HG12 ILE 146 76.882 1.332 33.200 1.00 0.00 H

ATOM 2414 HG13 ILE 146 78.065 1.239 31.874 1.00 0.00 H

ATOM 2415 CD1 ILE 146 77.611 3.246 32.589 1.00 0.00 C

ATOM 2416 HD11 ILE 146 77.928 3.704 31.652 1.00 0.00 H

ATOM 2417 HD12 ILE 146 76.801 3.819 33.040 1.00 0.00 H

ATOM 2418 HD13 ILE 146 78.492 3.295 33.228 1.00 0.00 H

ATOM 2419 C ILE 146 77.071 -0.079 29.668 1.00 0.00 C

ATOM 2420 O ILE 146 76.755 0.354 28.546 1.00 0.00 O

ATOM 2421 N SER 147 78.057 -0.901 29.938 1.00 0.00 N

ATOM 2422 H SER 147 78.116 -1.396 30.816 1.00 0.00 H

ATOM 2423 CA SER 147 79.154 -1.141 29.025 1.00 0.00 C

ATOM 2424 HA SER 147 79.720 -0.214 28.933 1.00 0.00 H

ATOM 2425 CB SER 147 80.205 -2.168 29.661 1.00 0.00 C

ATOM 2426 HB2 SER 147 79.613 -2.979 30.084 1.00 0.00 H

ATOM 2427 HB3 SER 147 80.982 -2.423 28.940 1.00 0.00 H

ATOM 2428 OG SER 147 80.860 -1.587 30.771 1.00 0.00 O

ATOM 2429 HG SER 147 80.222 -1.298 31.429 1.00 0.00 H

ATOM 2430 C SER 147 78.753 -1.642 27.537 1.00 0.00 C

ATOM 2431 O SER 147 79.296 -1.035 26.616 1.00 0.00 O

ATOM 2432 N PRO 148 77.918 -2.634 27.306 1.00 0.00 N

ATOM 2433 CD PRO 148 77.044 -3.197 28.310 1.00 0.00 C

ATOM 2434 HD2 PRO 148 76.130 -2.649 28.540 1.00 0.00 H

ATOM 2435 HD3 PRO 148 77.509 -3.352 29.284 1.00 0.00 H

ATOM 2436 CG PRO 148 76.540 -4.529 27.762 1.00 0.00 C

ATOM 2437 HG2 PRO 148 75.510 -4.416 27.425 1.00 0.00 H

ATOM 2438 HG3 PRO 148 76.499 -5.323 28.507 1.00 0.00 H

ATOM 2439 CB PRO 148 77.528 -4.782 26.567 1.00 0.00 C

ATOM 2440 HB2 PRO 148 77.104 -5.304 25.708 1.00 0.00 H

ATOM 2441 HB3 PRO 148 78.274 -5.417 27.044 1.00 0.00 H

ATOM 2442 CA PRO 148 78.019 -3.378 26.138 1.00 0.00 C

ATOM 2443 HA PRO 148 79.084 -3.563 25.996 1.00 0.00 H

ATOM 2444 C PRO 148 77.284 -2.802 24.838 1.00 0.00 C

ATOM 2445 O PRO 148 77.454 -3.234 23.672 1.00 0.00 O

ATOM 2446 N ILE 149 76.459 -1.743 24.916 1.00 0.00 N

ATOM 2447 H ILE 149 76.149 -1.509 25.848 1.00 0.00 H

ATOM 2448 CA ILE 149 75.784 -1.073 23.792 1.00 0.00 C

ATOM 2449 HA ILE 149 75.884 -1.777 22.966 1.00 0.00 H

ATOM 2450 CB ILE 149 74.296 -0.874 23.964 1.00 0.00 C

ATOM 2451 HB ILE 149 73.846 -1.731 24.465 1.00 0.00 H

ATOM 2452 CG2 ILE 149 74.000 0.327 24.822 1.00 0.00 C

ATOM 2453 HG21 ILE 149 74.231 1.236 24.266 1.00 0.00 H

ATOM 2454 HG22 ILE 149 72.976 0.367 25.194 1.00 0.00 H

ATOM 2455 HG23 ILE 149 74.545 0.198 25.757 1.00 0.00 H

ATOM 2456 CG1 ILE 149 73.603 -0.747 22.568 1.00 0.00 C

ATOM 2457 HG12 ILE 149 72.649 -0.242 22.721 1.00 0.00 H

ATOM 2458 HG13 ILE 149 74.154 -0.104 21.883 1.00 0.00 H

ATOM 2459 CD1 ILE 149 73.318 -1.990 21.745 1.00 0.00 C

ATOM 2460 HD11 ILE 149 72.655 -2.608 22.349 1.00 0.00 H

ATOM 2461 HD12 ILE 149 72.838 -1.785 20.788 1.00 0.00 H

ATOM 2462 HD13 ILE 149 74.200 -2.553 21.438 1.00 0.00 H

ATOM 2463 C ILE 149 76.499 0.251 23.494 1.00 0.00 C

ATOM 2464 O ILE 149 76.469 0.775 22.363 1.00 0.00 O

ATOM 2465 N LEU 150 77.387 0.723 24.445 1.00 0.00 N

ATOM 2466 H LEU 150 77.246 0.410 25.395 1.00 0.00 H

ATOM 2467 CA LEU 150 78.416 1.794 24.324 1.00 0.00 C

ATOM 2468 HA LEU 150 77.997 2.659 23.810 1.00 0.00 H

ATOM 2469 CB LEU 150 78.732 2.319 25.792 1.00 0.00 C

ATOM 2470 HB2 LEU 150 79.247 1.520 26.325 1.00 0.00 H

ATOM 2471 HB3 LEU 150 79.478 3.113 25.757 1.00 0.00 H

ATOM 2472 CG LEU 150 77.461 2.777 26.640 1.00 0.00 C

ATOM 2473 HG LEU 150 76.807 1.925 26.824 1.00 0.00 H

ATOM 2474 CD1 LEU 150 77.972 3.567 27.810 1.00 0.00 C

ATOM 2475 HD11 LEU 150 78.586 4.396 27.458 1.00 0.00 H

ATOM 2476 HD12 LEU 150 77.160 4.031 28.370 1.00 0.00 H

ATOM 2477 HD13 LEU 150 78.396 2.890 28.552 1.00 0.00 H

ATOM 2478 CD2 LEU 150 76.471 3.622 25.829 1.00 0.00 C

ATOM 2479 HD21 LEU 150 76.186 3.227 24.854 1.00 0.00 H

ATOM 2480 HD22 LEU 150 75.517 3.788 26.329 1.00 0.00 H

ATOM 2481 HD23 LEU 150 76.936 4.575 25.578 1.00 0.00 H

ATOM 2482 C LEU 150 79.640 1.374 23.593 1.00 0.00 C

ATOM 2483 O LEU 150 80.334 2.081 22.944 1.00 0.00 O

ATOM 2484 N PHE 151 79.931 0.019 23.753 1.00 0.00 N

ATOM 2485 H PHE 151 79.741 -0.300 24.693 1.00 0.00 H

ATOM 2486 CA PHE 151 80.811 -0.888 23.041 1.00 0.00 C

ATOM 2487 HA PHE 151 81.849 -0.622 23.237 1.00 0.00 H

ATOM 2488 CB PHE 151 80.653 -2.320 23.546 1.00 0.00 C

ATOM 2489 HB2 PHE 151 80.041 -2.328 24.448 1.00 0.00 H

ATOM 2490 HB3 PHE 151 80.132 -2.847 22.747 1.00 0.00 H

ATOM 2491 CG PHE 151 81.868 -3.128 23.929 1.00 0.00 C

ATOM 2492 CD1 PHE 151 82.569 -2.756 25.105 1.00 0.00 C

ATOM 2493 HD1 PHE 151 82.359 -1.793 25.547 1.00 0.00 H

ATOM 2494 CE1 PHE 151 83.661 -3.592 25.414 1.00 0.00 C

ATOM 2495 HE1 PHE 151 84.380 -3.333 26.177 1.00 0.00 H

ATOM 2496 CZ PHE 151 84.014 -4.712 24.630 1.00 0.00 C

ATOM 2497 HZ PHE 151 84.877 -5.305 24.894 1.00 0.00 H

ATOM 2498 CE2 PHE 151 83.275 -5.055 23.455 1.00 0.00 C

ATOM 2499 HE2 PHE 151 83.441 -5.948 22.870 1.00 0.00 H

ATOM 2500 CD2 PHE 151 82.189 -4.218 23.180 1.00 0.00 C

ATOM 2501 HD2 PHE 151 81.503 -4.666 22.476 1.00 0.00 H

ATOM 2502 C PHE 151 80.704 -0.829 21.528 1.00 0.00 C

ATOM 2503 O PHE 151 81.674 -0.935 20.797 1.00 0.00 O

ATOM 2504 N TYR 152 79.515 -0.406 21.059 1.00 0.00 N

ATOM 2505 H TYR 152 78.910 -0.209 21.843 1.00 0.00 H

ATOM 2506 CA TYR 152 79.046 -0.323 19.674 1.00 0.00 C

ATOM 2507 HA TYR 152 79.815 -0.726 19.015 1.00 0.00 H

ATOM 2508 CB TYR 152 77.876 -1.319 19.561 1.00 0.00 C

ATOM 2509 HB2 TYR 152 77.304 -1.299 20.489 1.00 0.00 H

ATOM 2510 HB3 TYR 152 77.337 -0.951 18.688 1.00 0.00 H

ATOM 2511 CG TYR 152 78.323 -2.702 19.092 1.00 0.00 C

ATOM 2512 CD1 TYR 152 78.708 -3.675 20.010 1.00 0.00 C

ATOM 2513 HD1 TYR 152 78.779 -3.402 21.053 1.00 0.00 H

ATOM 2514 CE1 TYR 152 78.860 -4.979 19.613 1.00 0.00 C

ATOM 2515 HE1 TYR 152 79.406 -5.640 20.271 1.00 0.00 H

ATOM 2516 CZ TYR 152 78.644 -5.281 18.246 1.00 0.00 C

ATOM 2517 OH TYR 152 78.742 -6.564 17.824 1.00 0.00 O

ATOM 2518 HH TYR 152 78.155 -6.727 17.081 1.00 0.00 H

ATOM 2519 CE2 TYR 152 78.283 -4.284 17.275 1.00 0.00 C

ATOM 2520 HE2 TYR 152 78.073 -4.684 16.294 1.00 0.00 H

ATOM 2521 CD2 TYR 152 78.233 -2.959 17.681 1.00 0.00 C

ATOM 2522 HD2 TYR 152 77.912 -2.163 17.026 1.00 0.00 H

ATOM 2523 C TYR 152 78.800 1.136 19.383 1.00 0.00 C

ATOM 2524 O TYR 152 79.388 1.657 18.422 1.00 0.00 O

ATOM 2525 N SER 153 77.664 1.660 19.856 1.00 0.00 N

ATOM 2526 H SER 153 77.245 1.309 20.705 1.00 0.00 H

ATOM 2527 CA SER 153 77.022 2.790 19.197 1.00 0.00 C

ATOM 2528 HA SER 153 77.261 2.870 18.137 1.00 0.00 H

ATOM 2529 CB SER 153 75.507 2.787 19.456 1.00 0.00 C

ATOM 2530 HB2 SER 153 75.140 1.865 19.906 1.00 0.00 H

ATOM 2531 HB3 SER 153 75.270 3.630 20.105 1.00 0.00 H

ATOM 2532 OG SER 153 74.685 2.950 18.354 1.00 0.00 O

ATOM 2533 HG SER 153 74.879 3.701 17.788 1.00 0.00 H

ATOM 2534 C SER 153 77.661 4.015 19.908 1.00 0.00 C

ATOM 2535 O SER 153 78.401 3.996 20.905 1.00 0.00 O

ATOM 2536 N GLY 154 77.466 5.204 19.320 1.00 0.00 N

ATOM 2537 H GLY 154 76.878 5.244 18.500 1.00 0.00 H

ATOM 2538 CA GLY 154 77.804 6.513 19.904 1.00 0.00 C

ATOM 2539 HA2 GLY 154 76.888 7.090 20.028 1.00 0.00 H

ATOM 2540 HA3 GLY 154 78.335 6.348 20.841 1.00 0.00 H

ATOM 2541 C GLY 154 78.775 7.448 19.203 1.00 0.00 C

ATOM 2542 O GLY 154 79.399 8.276 19.857 1.00 0.00 O

ATOM 2543 N THR 155 79.139 7.168 18.009 1.00 0.00 N

ATOM 2544 H THR 155 78.690 6.380 17.565 1.00 0.00 H

ATOM 2545 CA THR 155 79.869 8.134 17.162 1.00 0.00 C

ATOM 2546 HA THR 155 79.794 9.060 17.732 1.00 0.00 H

ATOM 2547 CB THR 155 81.286 7.688 16.917 1.00 0.00 C

ATOM 2548 HB THR 155 81.696 8.030 15.967 1.00 0.00 H

ATOM 2549 CG2 THR 155 82.232 8.035 18.043 1.00 0.00 C

ATOM 2550 HG21 THR 155 82.277 9.117 18.166 1.00 0.00 H

ATOM 2551 HG22 THR 155 81.893 7.581 18.975 1.00 0.00 H

ATOM 2552 HG23 THR 155 83.231 7.671 17.804 1.00 0.00 H

ATOM 2553 OG1 THR 155 81.379 6.274 16.747 1.00 0.00 O

ATOM 2554 HG1 THR 155 82.308 6.041 16.683 1.00 0.00 H

ATOM 2555 C THR 155 79.267 8.283 15.751 1.00 0.00 C

ATOM 2556 O THR 155 78.544 7.400 15.366 1.00 0.00 O

ATOM 2557 N GLY 156 79.449 9.454 15.130 1.00 0.00 N

ATOM 2558 H GLY 156 80.130 10.056 15.572 1.00 0.00 H

ATOM 2559 CA GLY 156 78.918 9.619 13.779 1.00 0.00 C

ATOM 2560 HA2 GLY 156 78.857 8.709 13.182 1.00 0.00 H

ATOM 2561 HA3 GLY 156 77.870 9.867 13.950 1.00 0.00 H

ATOM 2562 C GLY 156 79.567 10.739 12.998 1.00 0.00 C

ATOM 2563 O GLY 156 80.319 11.478 13.576 1.00 0.00 O

ATOM 2564 N VAL 157 79.174 10.958 11.708 1.00 0.00 N

ATOM 2565 H VAL 157 78.495 10.301 11.352 1.00 0.00 H

ATOM 2566 CA VAL 157 79.591 12.098 10.840 1.00 0.00 C

ATOM 2567 HA VAL 157 80.107 12.875 11.405 1.00 0.00 H

ATOM 2568 CB VAL 157 80.594 11.584 9.800 1.00 0.00 C

ATOM 2569 HB VAL 157 80.688 12.265 8.955 1.00 0.00 H

ATOM 2570 CG1 VAL 157 81.952 11.525 10.418 1.00 0.00 C

ATOM 2571 HG11 VAL 157 82.573 12.280 9.935 1.00 0.00 H

ATOM 2572 HG12 VAL 157 81.900 11.729 11.488 1.00 0.00 H

ATOM 2573 HG13 VAL 157 82.382 10.533 10.280 1.00 0.00 H

ATOM 2574 CG2 VAL 157 80.301 10.235 9.150 1.00 0.00 C

ATOM 2575 HG21 VAL 157 81.069 10.010 8.410 1.00 0.00 H

ATOM 2576 HG22 VAL 157 80.126 9.422 9.856 1.00 0.00 H

ATOM 2577 HG23 VAL 157 79.291 10.265 8.743 1.00 0.00 H

ATOM 2578 C VAL 157 78.329 12.700 10.117 1.00 0.00 C

ATOM 2579 O VAL 157 77.275 12.058 9.891 1.00 0.00 O

ATOM 2580 N ARG 158 78.436 14.044 9.948 1.00 0.00 N

ATOM 2581 H ARG 158 79.312 14.497 10.168 1.00 0.00 H

ATOM 2582 CA ARG 158 77.528 15.127 9.371 1.00 0.00 C

ATOM 2583 HA ARG 158 77.053 14.769 8.457 1.00 0.00 H

ATOM 2584 CB ARG 158 76.439 15.434 10.371 1.00 0.00 C

ATOM 2585 HB2 ARG 158 75.907 16.306 9.992 1.00 0.00 H

ATOM 2586 HB3 ARG 158 75.774 14.572 10.316 1.00 0.00 H

ATOM 2587 CG ARG 158 76.789 15.759 11.852 1.00 0.00 C

ATOM 2588 HG2 ARG 158 77.678 16.389 11.823 1.00 0.00 H

ATOM 2589 HG3 ARG 158 75.944 16.305 12.272 1.00 0.00 H

ATOM 2590 CD ARG 158 77.095 14.596 12.768 1.00 0.00 C

ATOM 2591 HD2 ARG 158 77.945 14.004 12.427 1.00 0.00 H

ATOM 2592 HD3 ARG 158 77.238 15.174 13.681 1.00 0.00 H

ATOM 2593 NE ARG 158 75.996 13.665 12.814 1.00 0.00 N

ATOM 2594 HE ARG 158 75.252 13.875 12.164 1.00 0.00 H

ATOM 2595 CZ ARG 158 75.826 12.605 13.553 1.00 0.00 C

ATOM 2596 NH1 ARG 158 74.659 12.042 13.481 1.00 0.00 N

ATOM 2597 HH11 ARG 158 74.438 11.321 14.153 1.00 0.00 H

ATOM 2598 HH12 ARG 158 73.935 12.349 12.847 1.00 0.00 H

ATOM 2599 NH2 ARG 158 76.707 12.156 14.301 1.00 0.00 N

ATOM 2600 HH21 ARG 158 77.403 12.800 14.649 1.00 0.00 H

ATOM 2601 HH22 ARG 158 76.318 11.596 15.046 1.00 0.00 H

ATOM 2602 C ARG 158 78.400 16.377 9.032 1.00 0.00 C

ATOM 2603 O ARG 158 78.606 17.342 9.811 1.00 0.00 O

ATOM 2604 N LYS 159 79.137 16.222 7.923 1.00 0.00 N

ATOM 2605 H LYS 159 79.147 15.290 7.533 1.00 0.00 H

ATOM 2606 CA LYS 159 79.981 17.166 7.218 1.00 0.00 C

ATOM 2607 HA LYS 159 80.695 16.725 6.523 1.00 0.00 H

ATOM 2608 CB LYS 159 79.128 18.214 6.451 1.00 0.00 C

ATOM 2609 HB2 LYS 159 78.628 18.966 7.061 1.00 0.00 H

ATOM 2610 HB3 LYS 159 79.742 18.848 5.811 1.00 0.00 H

ATOM 2611 CG LYS 159 78.112 17.671 5.479 1.00 0.00 C

ATOM 2612 HG2 LYS 159 78.642 17.656 4.526 1.00 0.00 H

ATOM 2613 HG3 LYS 159 77.786 16.685 5.808 1.00 0.00 H

ATOM 2614 CD LYS 159 76.864 18.519 5.430 1.00 0.00 C

ATOM 2615 HD2 LYS 159 76.635 18.702 6.479 1.00 0.00 H

ATOM 2616 HD3 LYS 159 77.092 19.438 4.891 1.00 0.00 H

ATOM 2617 CE LYS 159 75.790 17.620 4.628 1.00 0.00 C

ATOM 2618 HE2 LYS 159 76.307 17.204 3.764 1.00 0.00 H

ATOM 2619 HE3 LYS 159 75.566 16.744 5.236 1.00 0.00 H

ATOM 2620 NZ LYS 159 74.586 18.344 4.166 1.00 0.00 N

ATOM 2621 HZ1 LYS 159 73.810 18.223 4.801 1.00 0.00 H

ATOM 2622 HZ2 LYS 159 74.671 19.344 4.060 1.00 0.00 H

ATOM 2623 HZ3 LYS 159 74.195 17.927 3.334 1.00 0.00 H

ATOM 2624 C LYS 159 81.047 17.820 8.192 1.00 0.00 C

ATOM 2625 O LYS 159 80.947 18.976 8.505 1.00 0.00 O

ATOM 2626 N ASN 160 81.983 17.054 8.679 1.00 0.00 N

ATOM 2627 H ASN 160 82.090 16.135 8.273 1.00 0.00 H

ATOM 2628 CA ASN 160 82.894 17.382 9.737 1.00 0.00 C

ATOM 2629 HA ASN 160 83.092 18.453 9.705 1.00 0.00 H

ATOM 2630 CB ASN 160 82.196 17.043 11.057 1.00 0.00 C

ATOM 2631 HB2 ASN 160 82.812 17.532 11.812 1.00 0.00 H

ATOM 2632 HB3 ASN 160 81.173 17.416 11.101 1.00 0.00 H

ATOM 2633 CG ASN 160 82.241 15.545 11.167 1.00 0.00 C

ATOM 2634 OD1 ASN 160 81.481 14.743 10.677 1.00 0.00 O

ATOM 2635 ND2 ASN 160 83.239 15.064 11.901 1.00 0.00 N

ATOM 2636 HD21 ASN 160 83.513 14.139 11.603 1.00 0.00 H

ATOM 2637 HD22 ASN 160 83.889 15.724 12.306 1.00 0.00 H

ATOM 2638 C ASN 160 84.292 16.758 9.489 1.00 0.00 C

ATOM 2639 O ASN 160 84.433 15.723 8.829 1.00 0.00 O

ATOM 2640 N LYS 161 85.253 17.238 10.233 1.00 0.00 N

ATOM 2641 H LYS 161 84.923 17.925 10.896 1.00 0.00 H

ATOM 2642 CA LYS 161 86.653 17.245 9.762 1.00 0.00 C

ATOM 2643 HA LYS 161 86.800 17.195 8.683 1.00 0.00 H

ATOM 2644 CB LYS 161 87.414 18.409 10.419 1.00 0.00 C

ATOM 2645 HB2 LYS 161 87.551 18.176 11.475 1.00 0.00 H

ATOM 2646 HB3 LYS 161 88.431 18.527 10.046 1.00 0.00 H

ATOM 2647 CG LYS 161 86.666 19.782 10.335 1.00 0.00 C

ATOM 2648 HG2 LYS 161 86.129 19.717 9.389 1.00 0.00 H

ATOM 2649 HG3 LYS 161 85.947 19.866 11.150 1.00 0.00 H

ATOM 2650 CD LYS 161 87.736 20.841 10.271 1.00 0.00 C

ATOM 2651 HD2 LYS 161 88.560 20.595 10.942 1.00 0.00 H

ATOM 2652 HD3 LYS 161 88.200 20.918 9.287 1.00 0.00 H

ATOM 2653 CE LYS 161 86.893 22.111 10.480 1.00 0.00 C

ATOM 2654 HE2 LYS 161 86.630 22.617 9.551 1.00 0.00 H

ATOM 2655 HE3 LYS 161 86.003 21.785 11.018 1.00 0.00 H

ATOM 2656 NZ LYS 161 87.616 23.029 11.349 1.00 0.00 N

ATOM 2657 HZ1 LYS 161 88.403 23.313 10.782 1.00 0.00 H

ATOM 2658 HZ2 LYS 161 87.078 23.840 11.620 1.00 0.00 H

ATOM 2659 HZ3 LYS 161 87.991 22.561 12.161 1.00 0.00 H

ATOM 2660 C LYS 161 87.228 15.934 10.150 1.00 0.00 C

ATOM 2661 O LYS 161 88.126 15.508 9.382 1.00 0.00 O

ATOM 2662 N THR 162 86.880 15.389 11.348 1.00 0.00 N

ATOM 2663 H THR 162 86.372 15.947 12.020 1.00 0.00 H

ATOM 2664 CA THR 162 87.142 14.011 11.750 1.00 0.00 C

ATOM 2665 HA THR 162 87.535 13.466 10.891 1.00 0.00 H

ATOM 2666 CB THR 162 88.209 13.875 12.793 1.00 0.00 C

ATOM 2667 HB THR 162 88.370 12.803 12.902 1.00 0.00 H

ATOM 2668 CG2 THR 162 89.478 14.563 12.449 1.00 0.00 C

ATOM 2669 HG21 THR 162 89.364 15.645 12.384 1.00 0.00 H

ATOM 2670 HG22 THR 162 90.222 14.349 13.216 1.00 0.00 H

ATOM 2671 HG23 THR 162 89.702 14.130 11.474 1.00 0.00 H

ATOM 2672 OG1 THR 162 87.735 14.305 14.105 1.00 0.00 O

ATOM 2673 HG1 THR 162 87.423 15.208 14.007 1.00 0.00 H

ATOM 2674 C THR 162 85.821 13.237 12.118 1.00 0.00 C

ATOM 2675 O THR 162 85.184 12.867 11.109 1.00 0.00 O

ATOM 2676 N ILE 163 85.440 13.060 13.396 1.00 0.00 N

ATOM 2677 H ILE 163 86.086 13.372 14.107 1.00 0.00 H

ATOM 2678 CA ILE 163 84.162 12.461 13.867 1.00 0.00 C

ATOM 2679 HA ILE 163 83.485 12.313 13.026 1.00 0.00 H

ATOM 2680 CB ILE 163 84.478 11.050 14.439 1.00 0.00 C

ATOM 2681 HB ILE 163 83.599 10.423 14.288 1.00 0.00 H

ATOM 2682 CG2 ILE 163 85.633 10.393 13.567 1.00 0.00 C

ATOM 2683 HG21 ILE 163 86.646 10.723 13.797 1.00 0.00 H

ATOM 2684 HG22 ILE 163 85.620 9.338 13.840 1.00 0.00 H

ATOM 2685 HG23 ILE 163 85.378 10.404 12.507 1.00 0.00 H

ATOM 2686 CG1 ILE 163 84.877 11.188 15.933 1.00 0.00 C

ATOM 2687 HG12 ILE 163 85.581 11.999 16.115 1.00 0.00 H

ATOM 2688 HG13 ILE 163 84.030 11.509 16.539 1.00 0.00 H

ATOM 2689 CD1 ILE 163 85.378 9.835 16.505 1.00 0.00 C

ATOM 2690 HD11 ILE 163 85.645 10.064 17.536 1.00 0.00 H

ATOM 2691 HD12 ILE 163 84.594 9.095 16.668 1.00 0.00 H

ATOM 2692 HD13 ILE 163 86.184 9.480 15.864 1.00 0.00 H

ATOM 2693 C ILE 163 83.383 13.330 14.858 1.00 0.00 C

ATOM 2694 O ILE 163 83.956 14.094 15.658 1.00 0.00 O

ATOM 2695 N THR 164 82.047 13.333 14.835 1.00 0.00 N

ATOM 2696 H THR 164 81.568 12.835 14.099 1.00 0.00 H

ATOM 2697 CA THR 164 81.317 13.947 15.877 1.00 0.00 C

ATOM 2698 HA THR 164 81.996 14.735 16.201 1.00 0.00 H

ATOM 2699 CB THR 164 79.931 14.631 15.300 1.00 0.00 C

ATOM 2700 HB THR 164 79.513 15.293 16.059 1.00 0.00 H

ATOM 2701 CG2 THR 164 80.109 15.468 14.073 1.00 0.00 C

ATOM 2702 HG21 THR 164 80.261 14.761 13.257 1.00 0.00 H

ATOM 2703 HG22 THR 164 79.267 16.139 13.907 1.00 0.00 H

ATOM 2704 HG23 THR 164 80.898 16.221 14.076 1.00 0.00 H

ATOM 2705 OG1 THR 164 79.079 13.573 14.903 1.00 0.00 O

ATOM 2706 HG1 THR 164 79.641 12.924 14.473 1.00 0.00 H

ATOM 2707 C THR 164 80.948 12.874 16.931 1.00 0.00 C

ATOM 2708 O THR 164 80.666 11.725 16.664 1.00 0.00 O

ATOM 2709 N CYX 165 80.681 13.364 18.193 1.00 0.00 N

ATOM 2710 H CYX 165 80.835 14.353 18.325 1.00 0.00 H

ATOM 2711 CA CYX 165 80.444 12.488 19.401 1.00 0.00 C

ATOM 2712 HA CYX 165 80.921 11.551 19.113 1.00 0.00 H

ATOM 2713 CB CYX 165 81.449 12.905 20.502 1.00 0.00 C

ATOM 2714 HB2 CYX 165 82.406 13.279 20.138 1.00 0.00 H

ATOM 2715 HB3 CYX 165 80.973 13.807 20.887 1.00 0.00 H

ATOM 2716 SG CYX 165 81.801 11.738 21.858 1.00 0.00 S

ATOM 2717 C CYX 165 78.911 12.283 19.703 1.00 0.00 C

ATOM 2718 O CYX 165 78.578 12.110 20.890 1.00 0.00 O

ATOM 2719 N TYR 166 78.167 11.871 18.734 1.00 0.00 N

ATOM 2720 H TYR 166 78.611 11.571 17.877 1.00 0.00 H

ATOM 2721 CA TYR 166 76.843 11.312 18.816 1.00 0.00 C

ATOM 2722 HA TYR 166 76.892 10.590 19.631 1.00 0.00 H

ATOM 2723 CB TYR 166 75.809 12.364 19.066 1.00 0.00 C

ATOM 2724 HB2 TYR 166 74.937 11.896 19.523 1.00 0.00 H

ATOM 2725 HB3 TYR 166 76.139 13.038 19.857 1.00 0.00 H

ATOM 2726 CG TYR 166 75.545 13.281 17.878 1.00 0.00 C

ATOM 2727 CD1 TYR 166 74.597 12.954 16.898 1.00 0.00 C

ATOM 2728 HD1 TYR 166 74.081 12.005 16.920 1.00 0.00 H

ATOM 2729 CE1 TYR 166 74.254 13.884 15.923 1.00 0.00 C

ATOM 2730 HE1 TYR 166 73.416 13.692 15.269 1.00 0.00 H

ATOM 2731 CZ TYR 166 75.047 15.052 15.766 1.00 0.00 C

ATOM 2732 OH TYR 166 74.970 15.889 14.729 1.00 0.00 O

ATOM 2733 HH TYR 166 74.166 15.714 14.234 1.00 0.00 H

ATOM 2734 CE2 TYR 166 75.951 15.389 16.771 1.00 0.00 C

ATOM 2735 HE2 TYR 166 76.471 16.328 16.658 1.00 0.00 H

ATOM 2736 CD2 TYR 166 76.246 14.552 17.831 1.00 0.00 C

ATOM 2737 HD2 TYR 166 77.044 14.832 18.504 1.00 0.00 H

ATOM 2738 C TYR 166 76.414 10.564 17.611 1.00 0.00 C

ATOM 2739 O TYR 166 76.975 10.686 16.538 1.00 0.00 O

ATOM 2740 N ASP 167 75.405 9.759 17.767 1.00 0.00 N

ATOM 2741 H ASP 167 74.716 10.039 18.451 1.00 0.00 H

ATOM 2742 CA ASP 167 75.313 8.513 16.988 1.00 0.00 C

ATOM 2743 HA ASP 167 76.238 7.936 16.967 1.00 0.00 H

ATOM 2744 CB ASP 167 74.488 7.540 17.778 1.00 0.00 C

ATOM 2745 HB2 ASP 167 74.805 7.418 18.814 1.00 0.00 H

ATOM 2746 HB3 ASP 167 73.526 8.047 17.853 1.00 0.00 H

ATOM 2747 CG ASP 167 74.428 6.173 17.092 1.00 0.00 C

ATOM 2748 OD1 ASP 167 75.459 5.410 17.174 1.00 0.00 O

ATOM 2749 OD2 ASP 167 73.400 5.981 16.379 1.00 0.00 O

ATOM 2750 C ASP 167 74.856 8.711 15.405 1.00 0.00 C

ATOM 2751 O ASP 167 74.352 9.720 14.998 1.00 0.00 O

ATOM 2752 N THR 168 74.939 7.648 14.592 1.00 0.00 N

ATOM 2753 H THR 168 75.644 6.981 14.873 1.00 0.00 H

ATOM 2754 CA THR 168 74.319 7.497 13.256 1.00 0.00 C

ATOM 2755 HA THR 168 74.632 8.311 12.602 1.00 0.00 H

ATOM 2756 CB THR 168 74.510 6.056 12.582 1.00 0.00 C

ATOM 2757 HB THR 168 73.664 5.413 12.825 1.00 0.00 H

ATOM 2758 CG2 THR 168 74.542 6.392 11.111 1.00 0.00 C

ATOM 2759 HG21 THR 168 73.724 7.069 10.866 1.00 0.00 H

ATOM 2760 HG22 THR 168 75.528 6.777 10.851 1.00 0.00 H

ATOM 2761 HG23 THR 168 74.528 5.480 10.514 1.00 0.00 H

ATOM 2762 OG1 THR 168 75.803 5.616 12.968 1.00 0.00 O

ATOM 2763 HG1 THR 168 75.820 4.979 13.686 1.00 0.00 H

ATOM 2764 C THR 168 72.794 7.698 13.276 1.00 0.00 C

ATOM 2765 O THR 168 72.259 8.271 12.365 1.00 0.00 O

ATOM 2766 N THR 169 72.158 7.559 14.418 1.00 0.00 N

ATOM 2767 H THR 169 72.622 7.183 15.233 1.00 0.00 H

ATOM 2768 CA THR 169 70.725 7.873 14.722 1.00 0.00 C

ATOM 2769 HA THR 169 70.372 7.216 15.517 1.00 0.00 H

ATOM 2770 CB THR 169 70.534 9.375 15.182 1.00 0.00 C

ATOM 2771 HB THR 169 69.490 9.685 15.132 1.00 0.00 H

ATOM 2772 CG2 THR 169 71.053 9.615 16.620 1.00 0.00 C

ATOM 2773 HG21 THR 169 70.587 10.551 16.926 1.00 0.00 H

ATOM 2774 HG22 THR 169 70.765 8.820 17.307 1.00 0.00 H

ATOM 2775 HG23 THR 169 72.138 9.715 16.622 1.00 0.00 H

ATOM 2776 OG1 THR 169 71.065 10.372 14.306 1.00 0.00 O

ATOM 2777 HG1 THR 169 71.094 9.914 13.462 1.00 0.00 H

ATOM 2778 C THR 169 69.724 7.413 13.616 1.00 0.00 C

ATOM 2779 O THR 169 68.715 8.143 13.352 1.00 0.00 O

ATOM 2780 N SER 170 69.976 6.238 13.111 1.00 0.00 N

ATOM 2781 H SER 170 70.841 5.794 13.384 1.00 0.00 H

ATOM 2782 CA SER 170 69.177 5.502 12.142 1.00 0.00 C

ATOM 2783 HA SER 170 68.725 6.154 11.394 1.00 0.00 H

ATOM 2784 CB SER 170 70.118 4.471 11.462 1.00 0.00 C

ATOM 2785 HB2 SER 170 70.472 3.775 12.222 1.00 0.00 H

ATOM 2786 HB3 SER 170 69.493 4.056 10.672 1.00 0.00 H

ATOM 2787 OG SER 170 71.251 5.178 10.896 1.00 0.00 O

ATOM 2788 HG SER 170 71.678 4.548 10.310 1.00 0.00 H

ATOM 2789 C SER 170 68.028 4.655 12.770 1.00 0.00 C

ATOM 2790 O SER 170 68.302 4.222 13.878 1.00 0.00 O

ATOM 2791 N ASP 171 66.941 4.368 12.085 1.00 0.00 N

ATOM 2792 H ASP 171 66.989 4.670 11.122 1.00 0.00 H

ATOM 2793 CA ASP 171 65.567 4.218 12.651 1.00 0.00 C

ATOM 2794 HA ASP 171 65.360 5.121 13.225 1.00 0.00 H

ATOM 2795 CB ASP 171 64.564 4.073 11.502 1.00 0.00 C

ATOM 2796 HB2 ASP 171 64.889 4.784 10.742 1.00 0.00 H

ATOM 2797 HB3 ASP 171 64.579 3.114 10.986 1.00 0.00 H

ATOM 2798 CG ASP 171 63.205 4.333 11.943 1.00 0.00 C

ATOM 2799 OD1 ASP 171 62.571 3.355 12.441 1.00 0.00 O

ATOM 2800 OD2 ASP 171 62.714 5.492 11.768 1.00 0.00 O

ATOM 2801 C ASP 171 65.388 2.967 13.510 1.00 0.00 C

ATOM 2802 O ASP 171 65.306 2.934 14.741 1.00 0.00 O

ATOM 2803 N GLU 172 65.674 1.757 12.842 1.00 0.00 N

ATOM 2804 H GLU 172 65.699 1.698 11.834 1.00 0.00 H

ATOM 2805 CA GLU 172 65.805 0.474 13.639 1.00 0.00 C

ATOM 2806 HA GLU 172 64.832 0.118 13.979 1.00 0.00 H

ATOM 2807 CB GLU 172 66.268 -0.691 12.703 1.00 0.00 C

ATOM 2808 HB2 GLU 172 67.238 -0.524 12.234 1.00 0.00 H

ATOM 2809 HB3 GLU 172 66.372 -1.597 13.299 1.00 0.00 H

ATOM 2810 CG GLU 172 65.134 -1.013 11.653 1.00 0.00 C

ATOM 2811 HG2 GLU 172 64.866 -2.066 11.561 1.00 0.00 H

ATOM 2812 HG3 GLU 172 64.161 -0.641 11.974 1.00 0.00 H

ATOM 2813 CD GLU 172 65.466 -0.339 10.274 1.00 0.00 C

ATOM 2814 OE1 GLU 172 66.377 -0.819 9.555 1.00 0.00 O

ATOM 2815 OE2 GLU 172 64.988 0.741 9.905 1.00 0.00 O

ATOM 2816 C GLU 172 66.740 0.598 14.848 1.00 0.00 C

ATOM 2817 O GLU 172 66.528 -0.014 15.943 1.00 0.00 O

ATOM 2818 N TYR 173 67.847 1.362 14.681 1.00 0.00 N

ATOM 2819 H TYR 173 67.896 2.103 13.997 1.00 0.00 H

ATOM 2820 CA TYR 173 69.019 1.343 15.678 1.00 0.00 C

ATOM 2821 HA TYR 173 69.037 0.398 16.220 1.00 0.00 H

ATOM 2822 CB TYR 173 70.365 1.558 14.978 1.00 0.00 C

ATOM 2823 HB2 TYR 173 70.420 2.588 14.625 1.00 0.00 H

ATOM 2824 HB3 TYR 173 71.188 1.453 15.686 1.00 0.00 H

ATOM 2825 CG TYR 173 70.660 0.502 13.908 1.00 0.00 C

ATOM 2826 CD1 TYR 173 69.982 0.723 12.740 1.00 0.00 C

ATOM 2827 HD1 TYR 173 69.487 1.665 12.558 1.00 0.00 H

ATOM 2828 CE1 TYR 173 69.835 -0.298 11.818 1.00 0.00 C

ATOM 2829 HE1 TYR 173 69.210 -0.301 10.937 1.00 0.00 H

ATOM 2830 CZ TYR 173 70.593 -1.467 12.054 1.00 0.00 C

ATOM 2831 OH TYR 173 70.686 -2.313 10.982 1.00 0.00 O

ATOM 2832 HH TYR 173 71.125 -3.142 11.185 1.00 0.00 H

ATOM 2833 CE2 TYR 173 71.526 -1.612 13.121 1.00 0.00 C

ATOM 2834 HE2 TYR 173 72.270 -2.395 13.145 1.00 0.00 H

ATOM 2835 CD2 TYR 173 71.538 -0.659 14.121 1.00 0.00 C

ATOM 2836 HD2 TYR 173 71.936 -0.809 15.114 1.00 0.00 H

ATOM 2837 C TYR 173 68.774 2.305 16.822 1.00 0.00 C

ATOM 2838 O TYR 173 69.043 1.904 17.938 1.00 0.00 O

ATOM 2839 N LEU 174 68.508 3.613 16.637 1.00 0.00 N

ATOM 2840 H LEU 174 68.627 3.900 15.676 1.00 0.00 H

ATOM 2841 CA LEU 174 67.807 4.468 17.487 1.00 0.00 C

ATOM 2842 HA LEU 174 68.511 4.922 18.185 1.00 0.00 H

ATOM 2843 CB LEU 174 67.158 5.636 16.619 1.00 0.00 C

ATOM 2844 HB2 LEU 174 67.851 5.614 15.778 1.00 0.00 H

ATOM 2845 HB3 LEU 174 66.161 5.308 16.326 1.00 0.00 H

ATOM 2846 CG LEU 174 67.199 7.031 17.262 1.00 0.00 C

ATOM 2847 HG LEU 174 68.233 7.177 17.573 1.00 0.00 H

ATOM 2848 CD1 LEU 174 66.996 8.148 16.265 1.00 0.00 C

ATOM 2849 HD11 LEU 174 67.571 8.022 15.348 1.00 0.00 H

ATOM 2850 HD12 LEU 174 65.926 8.179 16.060 1.00 0.00 H

ATOM 2851 HD13 LEU 174 67.258 9.108 16.711 1.00 0.00 H

ATOM 2852 CD2 LEU 174 66.280 7.140 18.473 1.00 0.00 C

ATOM 2853 HD21 LEU 174 65.473 6.423 18.321 1.00 0.00 H

ATOM 2854 HD22 LEU 174 66.821 6.730 19.326 1.00 0.00 H

ATOM 2855 HD23 LEU 174 65.815 8.111 18.640 1.00 0.00 H

ATOM 2856 C LEU 174 66.728 3.760 18.301 1.00 0.00 C

ATOM 2857 O LEU 174 66.715 3.983 19.483 1.00 0.00 O

ATOM 2858 N ARG 175 65.714 3.137 17.659 1.00 0.00 N

ATOM 2859 H ARG 175 65.778 3.304 16.665 1.00 0.00 H

ATOM 2860 CA ARG 175 64.586 2.436 18.322 1.00 0.00 C

ATOM 2861 HA ARG 175 64.000 3.118 18.937 1.00 0.00 H

ATOM 2862 CB ARG 175 63.639 1.802 17.265 1.00 0.00 C

ATOM 2863 HB2 ARG 175 63.228 2.597 16.642 1.00 0.00 H

ATOM 2864 HB3 ARG 175 64.206 1.293 16.486 1.00 0.00 H

ATOM 2865 CG ARG 175 62.579 0.879 17.984 1.00 0.00 C

ATOM 2866 HG2 ARG 175 63.121 -0.032 18.240 1.00 0.00 H

ATOM 2867 HG3 ARG 175 62.173 1.338 18.886 1.00 0.00 H

ATOM 2868 CD ARG 175 61.376 0.523 17.064 1.00 0.00 C

ATOM 2869 HD2 ARG 175 60.823 -0.363 17.374 1.00 0.00 H

ATOM 2870 HD3 ARG 175 60.679 1.356 17.139 1.00 0.00 H

ATOM 2871 NE ARG 175 61.796 0.293 15.706 1.00 0.00 N

ATOM 2872 HE ARG 175 62.198 -0.611 15.507 1.00 0.00 H

ATOM 2873 CZ ARG 175 61.767 1.130 14.727 1.00 0.00 C

ATOM 2874 NH1 ARG 175 62.372 0.896 13.603 1.00 0.00 N

ATOM 2875 HH11 ARG 175 62.408 1.700 12.993 1.00 0.00 H

ATOM 2876 HH12 ARG 175 62.933 0.065 13.484 1.00 0.00 H

ATOM 2877 NH2 ARG 175 61.144 2.271 14.802 1.00 0.00 N

ATOM 2878 HH21 ARG 175 61.169 2.895 14.008 1.00 0.00 H

ATOM 2879 HH22 ARG 175 60.276 2.306 15.317 1.00 0.00 H

ATOM 2880 C ARG 175 65.080 1.313 19.229 1.00 0.00 C

ATOM 2881 O ARG 175 64.458 1.141 20.282 1.00 0.00 O

ATOM 2882 N SER 176 66.130 0.618 18.832 1.00 0.00 N

ATOM 2883 H SER 176 66.413 0.638 17.863 1.00 0.00 H

ATOM 2884 CA SER 176 66.762 -0.412 19.716 1.00 0.00 C

ATOM 2885 HA SER 176 65.944 -0.996 20.136 1.00 0.00 H

ATOM 2886 CB SER 176 67.650 -1.349 18.765 1.00 0.00 C

ATOM 2887 HB2 SER 176 68.262 -0.714 18.124 1.00 0.00 H

ATOM 2888 HB3 SER 176 68.222 -2.002 19.423 1.00 0.00 H

ATOM 2889 OG SER 176 66.793 -2.181 17.958 1.00 0.00 O

ATOM 2890 HG SER 176 66.717 -1.702 17.129 1.00 0.00 H

ATOM 2891 C SER 176 67.559 0.158 20.829 1.00 0.00 C

ATOM 2892 O SER 176 67.302 -0.247 21.939 1.00 0.00 O

ATOM 2893 N TYR 177 68.301 1.189 20.624 1.00 0.00 N

ATOM 2894 H TYR 177 68.474 1.600 19.718 1.00 0.00 H

ATOM 2895 CA TYR 177 69.036 1.716 21.818 1.00 0.00 C

ATOM 2896 HA TYR 177 69.181 0.849 22.462 1.00 0.00 H

ATOM 2897 CB TYR 177 70.485 2.163 21.441 1.00 0.00 C

ATOM 2898 HB2 TYR 177 70.972 2.629 22.298 1.00 0.00 H

ATOM 2899 HB3 TYR 177 71.131 1.306 21.244 1.00 0.00 H

ATOM 2900 CG TYR 177 70.653 3.211 20.375 1.00 0.00 C

ATOM 2901 CD1 TYR 177 71.692 3.099 19.404 1.00 0.00 C

ATOM 2902 HD1 TYR 177 72.159 2.185 19.068 1.00 0.00 H

ATOM 2903 CE1 TYR 177 71.795 4.160 18.536 1.00 0.00 C

ATOM 2904 HE1 TYR 177 72.361 4.010 17.629 1.00 0.00 H

ATOM 2905 CZ TYR 177 71.132 5.403 18.762 1.00 0.00 C

ATOM 2906 OH TYR 177 71.381 6.437 17.859 1.00 0.00 O

ATOM 2907 HH TYR 177 72.171 6.271 17.339 1.00 0.00 H

ATOM 2908 CE2 TYR 177 70.323 5.601 19.917 1.00 0.00 C

ATOM 2909 HE2 TYR 177 69.698 6.465 20.092 1.00 0.00 H

ATOM 2910 CD2 TYR 177 70.188 4.551 20.821 1.00 0.00 C

ATOM 2911 HD2 TYR 177 69.502 4.570 21.655 1.00 0.00 H

ATOM 2912 C TYR 177 68.317 2.738 22.754 1.00 0.00 C

ATOM 2913 O TYR 177 68.726 2.850 23.860 1.00 0.00 O

ATOM 2914 N PHE 178 67.223 3.343 22.301 1.00 0.00 N

ATOM 2915 H PHE 178 67.101 3.406 21.300 1.00 0.00 H

ATOM 2916 CA PHE 178 66.108 3.900 23.130 1.00 0.00 C

ATOM 2917 HA PHE 178 66.790 4.580 23.641 1.00 0.00 H

ATOM 2918 CB PHE 178 65.248 4.746 22.155 1.00 0.00 C

ATOM 2919 HB2 PHE 178 65.891 5.326 21.493 1.00 0.00 H

ATOM 2920 HB3 PHE 178 64.733 3.988 21.565 1.00 0.00 H

ATOM 2921 CG PHE 178 64.075 5.578 22.677 1.00 0.00 C

ATOM 2922 CD1 PHE 178 63.079 6.030 21.750 1.00 0.00 C

ATOM 2923 HD1 PHE 178 63.259 5.760 20.720 1.00 0.00 H

ATOM 2924 CE1 PHE 178 61.865 6.525 22.082 1.00 0.00 C

ATOM 2925 HE1 PHE 178 61.218 6.792 21.259 1.00 0.00 H

ATOM 2926 CZ PHE 178 61.606 6.797 23.440 1.00 0.00 C

ATOM 2927 HZ PHE 178 60.678 7.212 23.804 1.00 0.00 H

ATOM 2928 CE2 PHE 178 62.560 6.354 24.464 1.00 0.00 C

ATOM 2929 HE2 PHE 178 62.437 6.597 25.510 1.00 0.00 H

ATOM 2930 CD2 PHE 178 63.795 5.739 24.073 1.00 0.00 C

ATOM 2931 HD2 PHE 178 64.653 5.552 24.702 1.00 0.00 H

ATOM 2932 C PHE 178 65.435 2.987 24.169 1.00 0.00 C

ATOM 2933 O PHE 178 65.389 3.348 25.381 1.00 0.00 O

ATOM 2934 N ILE 179 65.207 1.758 23.751 1.00 0.00 N

ATOM 2935 H ILE 179 65.113 1.552 22.767 1.00 0.00 H

ATOM 2936 CA ILE 179 64.734 0.747 24.762 1.00 0.00 C

ATOM 2937 HA ILE 179 63.806 1.123 25.193 1.00 0.00 H

ATOM 2938 CB ILE 179 64.415 -0.640 24.026 1.00 0.00 C

ATOM 2939 HB ILE 179 65.214 -0.993 23.374 1.00 0.00 H

ATOM 2940 CG2 ILE 179 64.299 -1.791 25.052 1.00 0.00 C

ATOM 2941 HG21 ILE 179 63.853 -1.354 25.946 1.00 0.00 H

ATOM 2942 HG22 ILE 179 63.740 -2.621 24.620 1.00 0.00 H

ATOM 2943 HG23 ILE 179 65.289 -2.091 25.395 1.00 0.00 H

ATOM 2944 CG1 ILE 179 63.117 -0.408 23.214 1.00 0.00 C

ATOM 2945 HG12 ILE 179 62.255 -0.289 23.870 1.00 0.00 H

ATOM 2946 HG13 ILE 179 63.221 0.518 22.648 1.00 0.00 H

ATOM 2947 CD1 ILE 179 62.904 -1.585 22.195 1.00 0.00 C

ATOM 2948 HD11 ILE 179 61.922 -1.495 21.731 1.00 0.00 H

ATOM 2949 HD12 ILE 179 63.665 -1.654 21.417 1.00 0.00 H

ATOM 2950 HD13 ILE 179 62.832 -2.523 22.746 1.00 0.00 H

ATOM 2951 C ILE 179 65.748 0.597 25.956 1.00 0.00 C

ATOM 2952 O ILE 179 65.251 0.463 27.090 1.00 0.00 O

ATOM 2953 N TYR 180 67.057 0.651 25.837 1.00 0.00 N

ATOM 2954 H TYR 180 67.392 0.485 24.899 1.00 0.00 H

ATOM 2955 CA TYR 180 67.995 0.493 26.915 1.00 0.00 C

ATOM 2956 HA TYR 180 67.618 -0.326 27.527 1.00 0.00 H

ATOM 2957 CB TYR 180 69.376 0.236 26.236 1.00 0.00 C

ATOM 2958 HB2 TYR 180 69.146 -0.366 25.357 1.00 0.00 H

ATOM 2959 HB3 TYR 180 69.708 1.159 25.758 1.00 0.00 H

ATOM 2960 CG TYR 180 70.506 -0.545 26.875 1.00 0.00 C

ATOM 2961 CD1 TYR 180 70.811 -0.308 28.261 1.00 0.00 C

ATOM 2962 HD1 TYR 180 70.280 0.469 28.791 1.00 0.00 H

ATOM 2963 CE1 TYR 180 71.804 -0.988 28.901 1.00 0.00 C

ATOM 2964 HE1 TYR 180 72.183 -0.600 29.835 1.00 0.00 H

ATOM 2965 CZ TYR 180 72.533 -1.966 28.168 1.00 0.00 C

ATOM 2966 OH TYR 180 73.618 -2.549 28.753 1.00 0.00 O

ATOM 2967 HH TYR 180 73.606 -2.167 29.634 1.00 0.00 H

ATOM 2968 CE2 TYR 180 72.381 -2.139 26.812 1.00 0.00 C

ATOM 2969 HE2 TYR 180 73.117 -2.723 26.280 1.00 0.00 H

ATOM 2970 CD2 TYR 180 71.280 -1.506 26.147 1.00 0.00 C

ATOM 2971 HD2 TYR 180 71.006 -1.712 25.123 1.00 0.00 H

ATOM 2972 C TYR 180 68.122 1.861 27.670 1.00 0.00 C

ATOM 2973 O TYR 180 68.138 1.877 28.884 1.00 0.00 O

ATOM 2974 N SER 181 68.079 2.973 26.949 1.00 0.00 N

ATOM 2975 H SER 181 67.886 2.901 25.960 1.00 0.00 H

ATOM 2976 CA SER 181 67.992 4.326 27.564 1.00 0.00 C

ATOM 2977 HA SER 181 68.903 4.536 28.123 1.00 0.00 H

ATOM 2978 CB SER 181 67.831 5.438 26.448 1.00 0.00 C

ATOM 2979 HB2 SER 181 66.945 5.154 25.880 1.00 0.00 H

ATOM 2980 HB3 SER 181 67.691 6.388 26.964 1.00 0.00 H

ATOM 2981 OG SER 181 68.949 5.617 25.584 1.00 0.00 O

ATOM 2982 HG SER 181 69.472 6.376 25.853 1.00 0.00 H

ATOM 2983 C SER 181 66.787 4.560 28.486 1.00 0.00 C

ATOM 2984 O SER 181 66.883 5.206 29.518 1.00 0.00 O

ATOM 2985 N MET 182 65.661 3.960 28.086 1.00 0.00 N

ATOM 2986 H MET 182 65.558 3.715 27.111 1.00 0.00 H

ATOM 2987 CA MET 182 64.508 3.730 28.925 1.00 0.00 C

ATOM 2988 HA MET 182 64.192 4.663 29.391 1.00 0.00 H

ATOM 2989 CB MET 182 63.324 3.194 28.058 1.00 0.00 C

ATOM 2990 HB2 MET 182 63.235 3.888 27.222 1.00 0.00 H

ATOM 2991 HB3 MET 182 63.508 2.207 27.634 1.00 0.00 H

ATOM 2992 CG MET 182 61.959 3.128 28.809 1.00 0.00 C

ATOM 2993 HG2 MET 182 61.769 4.060 29.343 1.00 0.00 H

ATOM 2994 HG3 MET 182 61.165 2.968 28.080 1.00 0.00 H

ATOM 2995 SD MET 182 61.804 1.871 30.121 1.00 0.00 S

ATOM 2996 CE MET 182 62.048 0.214 29.286 1.00 0.00 C

ATOM 2997 HE1 MET 182 61.361 -0.572 29.597 1.00 0.00 H

ATOM 2998 HE2 MET 182 62.003 0.480 28.230 1.00 0.00 H

ATOM 2999 HE3 MET 182 63.053 -0.116 29.548 1.00 0.00 H

ATOM 3000 C MET 182 64.949 2.867 30.130 1.00 0.00 C

ATOM 3001 O MET 182 64.916 3.370 31.281 1.00 0.00 O

ATOM 3002 N CYX 183 65.451 1.645 29.988 1.00 0.00 N

ATOM 3003 H CYX 183 65.450 1.272 29.049 1.00 0.00 H

ATOM 3004 CA CYX 183 65.799 0.747 31.161 1.00 0.00 C

ATOM 3005 HA CYX 183 64.884 0.489 31.695 1.00 0.00 H

ATOM 3006 CB CYX 183 66.450 -0.634 30.673 1.00 0.00 C

ATOM 3007 HB2 CYX 183 67.181 -0.354 29.915 1.00 0.00 H

ATOM 3008 HB3 CYX 183 66.950 -1.165 31.483 1.00 0.00 H

ATOM 3009 SG CYX 183 65.322 -1.757 29.854 1.00 0.00 S

ATOM 3010 C CYX 183 66.770 1.362 32.171 1.00 0.00 C

ATOM 3011 O CYX 183 66.459 1.318 33.349 1.00 0.00 O

ATOM 3012 N THR 184 67.863 2.000 31.721 1.00 0.00 N

ATOM 3013 H THR 184 67.995 2.046 30.721 1.00 0.00 H

ATOM 3014 CA THR 184 68.762 2.947 32.537 1.00 0.00 C

ATOM 3015 HA THR 184 69.241 2.385 33.338 1.00 0.00 H

ATOM 3016 CB THR 184 69.866 3.485 31.705 1.00 0.00 C

ATOM 3017 HB THR 184 70.399 4.202 32.330 1.00 0.00 H

ATOM 3018 CG2 THR 184 70.871 2.472 31.115 1.00 0.00 C

ATOM 3019 HG21 THR 184 71.234 2.785 30.136 1.00 0.00 H

ATOM 3020 HG22 THR 184 71.744 2.566 31.760 1.00 0.00 H

ATOM 3021 HG23 THR 184 70.451 1.474 30.987 1.00 0.00 H

ATOM 3022 OG1 THR 184 69.369 4.202 30.607 1.00 0.00 O

ATOM 3023 HG1 THR 184 70.069 4.078 29.962 1.00 0.00 H

ATOM 3024 C THR 184 67.997 4.100 33.181 1.00 0.00 C

ATOM 3025 O THR 184 68.231 4.318 34.390 1.00 0.00 O

ATOM 3026 N THR 185 67.127 4.909 32.511 1.00 0.00 N

ATOM 3027 H THR 185 67.261 4.885 31.511 1.00 0.00 H

ATOM 3028 CA THR 185 66.311 5.912 33.141 1.00 0.00 C

ATOM 3029 HA THR 185 66.877 6.701 33.637 1.00 0.00 H

ATOM 3030 CB THR 185 65.462 6.629 32.067 1.00 0.00 C

ATOM 3031 HB THR 185 64.812 5.922 31.551 1.00 0.00 H

ATOM 3032 CG2 THR 185 64.664 7.795 32.693 1.00 0.00 C

ATOM 3033 HG21 THR 185 63.645 7.440 32.846 1.00 0.00 H

ATOM 3034 HG22 THR 185 65.158 8.174 33.588 1.00 0.00 H

ATOM 3035 HG23 THR 185 64.454 8.600 31.989 1.00 0.00 H

ATOM 3036 OG1 THR 185 66.329 7.241 31.140 1.00 0.00 O

ATOM 3037 HG1 THR 185 66.655 6.522 30.594 1.00 0.00 H

ATOM 3038 C THR 185 65.354 5.396 34.254 1.00 0.00 C

ATOM 3039 O THR 185 65.327 5.920 35.350 1.00 0.00 O

ATOM 3040 N VAL 186 64.700 4.253 34.042 1.00 0.00 N

ATOM 3041 H VAL 186 64.822 3.764 33.167 1.00 0.00 H

ATOM 3042 CA VAL 186 63.760 3.743 35.025 1.00 0.00 C

ATOM 3043 HA VAL 186 63.033 4.464 35.397 1.00 0.00 H

ATOM 3044 CB VAL 186 62.844 2.692 34.342 1.00 0.00 C

ATOM 3045 HB VAL 186 63.402 1.948 33.773 1.00 0.00 H

ATOM 3046 CG1 VAL 186 61.896 1.985 35.346 1.00 0.00 C

ATOM 3047 HG11 VAL 186 61.290 1.382 34.670 1.00 0.00 H

ATOM 3048 HG12 VAL 186 62.465 1.287 35.959 1.00 0.00 H

ATOM 3049 HG13 VAL 186 61.224 2.645 35.897 1.00 0.00 H

ATOM 3050 CG2 VAL 186 61.916 3.464 33.247 1.00 0.00 C

ATOM 3051 HG21 VAL 186 61.519 4.438 33.535 1.00 0.00 H

ATOM 3052 HG22 VAL 186 62.536 3.702 32.383 1.00 0.00 H

ATOM 3053 HG23 VAL 186 61.074 2.877 32.880 1.00 0.00 H

ATOM 3054 C VAL 186 64.495 3.119 36.230 1.00 0.00 C

ATOM 3055 O VAL 186 64.161 3.436 37.384 1.00 0.00 O

ATOM 3056 N ALA 187 65.549 2.344 35.933 1.00 0.00 N

ATOM 3057 H ALA 187 65.633 2.057 34.968 1.00 0.00 H

ATOM 3058 CA ALA 187 66.151 1.552 36.998 1.00 0.00 C

ATOM 3059 HA ALA 187 65.442 1.363 37.804 1.00 0.00 H

ATOM 3060 CB ALA 187 66.476 0.122 36.611 1.00 0.00 C

ATOM 3061 HB1 ALA 187 65.938 -0.048 35.679 1.00 0.00 H

ATOM 3062 HB2 ALA 187 67.546 -0.068 36.537 1.00 0.00 H

ATOM 3063 HB3 ALA 187 66.049 -0.527 37.377 1.00 0.00 H

ATOM 3064 C ALA 187 67.284 2.183 37.743 1.00 0.00 C

ATOM 3065 O ALA 187 67.398 2.060 38.953 1.00 0.00 O

ATOM 3066 N MET 188 68.230 2.822 37.026 1.00 0.00 N

ATOM 3067 H MET 188 68.074 2.976 36.040 1.00 0.00 H

ATOM 3068 CA MET 188 69.383 3.474 37.626 1.00 0.00 C

ATOM 3069 HA MET 188 69.554 2.970 38.577 1.00 0.00 H

ATOM 3070 CB MET 188 70.559 3.338 36.689 1.00 0.00 C

ATOM 3071 HB2 MET 188 70.408 3.998 35.834 1.00 0.00 H

ATOM 3072 HB3 MET 188 71.424 3.826 37.137 1.00 0.00 H

ATOM 3073 CG MET 188 71.001 1.956 36.226 1.00 0.00 C

ATOM 3074 HG2 MET 188 70.179 1.241 36.202 1.00 0.00 H

ATOM 3075 HG3 MET 188 71.340 2.048 35.194 1.00 0.00 H

ATOM 3076 SD MET 188 72.343 1.228 37.133 1.00 0.00 S

ATOM 3077 CE MET 188 71.264 0.385 38.447 1.00 0.00 C

ATOM 3078 HE1 MET 188 70.717 1.236 38.855 1.00 0.00 H

ATOM 3079 HE2 MET 188 70.641 -0.386 37.993 1.00 0.00 H

ATOM 3080 HE3 MET 188 71.915 -0.019 39.222 1.00 0.00 H

ATOM 3081 C MET 188 69.258 4.912 38.073 1.00 0.00 C

ATOM 3082 O MET 188 70.060 5.330 38.919 1.00 0.00 O

ATOM 3083 N PHE 189 68.237 5.645 37.687 1.00 0.00 N

ATOM 3084 H PHE 189 67.545 5.165 37.129 1.00 0.00 H

ATOM 3085 CA PHE 189 67.914 6.981 38.221 1.00 0.00 C

ATOM 3086 HA PHE 189 68.714 7.333 38.873 1.00 0.00 H

ATOM 3087 CB PHE 189 67.956 7.981 37.055 1.00 0.00 C

ATOM 3088 HB2 PHE 189 68.988 8.134 36.738 1.00 0.00 H

ATOM 3089 HB3 PHE 189 67.552 7.573 36.128 1.00 0.00 H

ATOM 3090 CG PHE 189 67.412 9.434 37.318 1.00 0.00 C

ATOM 3091 CD1 PHE 189 68.271 10.314 38.052 1.00 0.00 C

ATOM 3092 HD1 PHE 189 69.223 9.872 38.306 1.00 0.00 H

ATOM 3093 CE1 PHE 189 67.798 11.571 38.573 1.00 0.00 C

ATOM 3094 HE1 PHE 189 68.380 12.305 39.110 1.00 0.00 H

ATOM 3095 CZ PHE 189 66.520 12.057 38.198 1.00 0.00 C

ATOM 3096 HZ PHE 189 66.129 12.975 38.610 1.00 0.00 H

ATOM 3097 CE2 PHE 189 65.696 11.145 37.522 1.00 0.00 C

ATOM 3098 HE2 PHE 189 64.717 11.531 37.278 1.00 0.00 H

ATOM 3099 CD2 PHE 189 66.107 9.889 36.988 1.00 0.00 C

ATOM 3100 HD2 PHE 189 65.561 9.287 36.278 1.00 0.00 H

ATOM 3101 C PHE 189 66.567 7.025 38.851 1.00 0.00 C

ATOM 3102 O PHE 189 66.415 7.495 39.993 1.00 0.00 O

ATOM 3103 N CYX 190 65.502 6.473 38.248 1.00 0.00 N

ATOM 3104 H CYX 190 65.603 6.359 37.249 1.00 0.00 H

ATOM 3105 CA CYX 190 64.182 6.806 38.777 1.00 0.00 C

ATOM 3106 HA CYX 190 64.235 7.884 38.929 1.00 0.00 H

ATOM 3107 CB CYX 190 63.199 6.437 37.702 1.00 0.00 C

ATOM 3108 HB2 CYX 190 63.593 6.442 36.686 1.00 0.00 H

ATOM 3109 HB3 CYX 190 62.819 5.447 37.954 1.00 0.00 H

ATOM 3110 SG CYX 190 61.720 7.508 37.591 1.00 0.00 S

ATOM 3111 C CYX 190 63.801 6.110 40.130 1.00 0.00 C

ATOM 3112 O CYX 190 63.334 6.815 41.019 1.00 0.00 O

ATOM 3113 N VAL 191 64.029 4.810 40.179 1.00 0.00 N

ATOM 3114 H VAL 191 64.280 4.460 39.266 1.00 0.00 H

ATOM 3115 CA VAL 191 63.932 3.968 41.472 1.00 0.00 C

ATOM 3116 HA VAL 191 62.875 4.115 41.698 1.00 0.00 H

ATOM 3117 CB VAL 191 64.169 2.424 41.254 1.00 0.00 C

ATOM 3118 HB VAL 191 65.170 2.262 40.855 1.00 0.00 H

ATOM 3119 CG1 VAL 191 64.006 1.796 42.613 1.00 0.00 C

ATOM 3120 HG11 VAL 191 64.943 1.823 43.168 1.00 0.00 H

ATOM 3121 HG12 VAL 191 63.198 2.247 43.189 1.00 0.00 H

ATOM 3122 HG13 VAL 191 63.782 0.730 42.556 1.00 0.00 H

ATOM 3123 CG2 VAL 191 63.094 1.860 40.314 1.00 0.00 C

ATOM 3124 HG21 VAL 191 62.226 2.519 40.314 1.00 0.00 H

ATOM 3125 HG22 VAL 191 63.354 1.616 39.284 1.00 0.00 H

ATOM 3126 HG23 VAL 191 62.685 0.940 40.731 1.00 0.00 H

ATOM 3127 C VAL 191 64.720 4.664 42.588 1.00 0.00 C

ATOM 3128 O VAL 191 64.135 5.001 43.620 1.00 0.00 O

ATOM 3129 N PRO 192 66.023 4.803 42.415 1.00 0.00 N

ATOM 3130 CD PRO 192 66.782 4.052 41.512 1.00 0.00 C

ATOM 3131 HD2 PRO 192 66.311 4.027 40.529 1.00 0.00 H

ATOM 3132 HD3 PRO 192 66.743 3.033 41.896 1.00 0.00 H

ATOM 3133 CG PRO 192 68.158 4.753 41.457 1.00 0.00 C

ATOM 3134 HG2 PRO 192 67.889 5.474 40.686 1.00 0.00 H

ATOM 3135 HG3 PRO 192 69.049 4.147 41.290 1.00 0.00 H

ATOM 3136 CB PRO 192 68.280 5.516 42.730 1.00 0.00 C

ATOM 3137 HB2 PRO 192 68.715 6.512 42.651 1.00 0.00 H

ATOM 3138 HB3 PRO 192 68.939 4.968 43.404 1.00 0.00 H

ATOM 3139 CA PRO 192 66.879 5.556 43.394 1.00 0.00 C

ATOM 3140 HA PRO 192 66.906 5.088 44.378 1.00 0.00 H

ATOM 3141 C PRO 192 66.491 6.965 43.783 1.00 0.00 C

ATOM 3142 O PRO 192 66.564 7.346 44.881 1.00 0.00 O

ATOM 3143 N LEU 193 65.928 7.775 42.846 1.00 0.00 N

ATOM 3144 H LEU 193 65.964 7.615 41.849 1.00 0.00 H

ATOM 3145 CA LEU 193 65.281 9.081 43.063 1.00 0.00 C

ATOM 3146 HA LEU 193 66.009 9.696 43.592 1.00 0.00 H

ATOM 3147 CB LEU 193 64.972 9.730 41.714 1.00 0.00 C

ATOM 3148 HB2 LEU 193 65.899 10.098 41.275 1.00 0.00 H

ATOM 3149 HB3 LEU 193 64.513 9.081 40.968 1.00 0.00 H

ATOM 3150 CG LEU 193 63.960 10.911 41.825 1.00 0.00 C

ATOM 3151 HG LEU 193 62.999 10.442 42.037 1.00 0.00 H

ATOM 3152 CD1 LEU 193 64.291 12.079 42.818 1.00 0.00 C

ATOM 3153 HD11 LEU 193 63.474 12.790 42.692 1.00 0.00 H

ATOM 3154 HD12 LEU 193 64.249 11.823 43.876 1.00 0.00 H

ATOM 3155 HD13 LEU 193 65.212 12.595 42.546 1.00 0.00 H

ATOM 3156 CD2 LEU 193 63.672 11.542 40.452 1.00 0.00 C

ATOM 3157 HD21 LEU 193 64.482 12.196 40.131 1.00 0.00 H

ATOM 3158 HD22 LEU 193 63.523 10.677 39.806 1.00 0.00 H

ATOM 3159 HD23 LEU 193 62.752 12.125 40.505 1.00 0.00 H

ATOM 3160 C LEU 193 64.100 8.959 44.042 1.00 0.00 C

ATOM 3161 O LEU 193 64.117 9.666 45.035 1.00 0.00 O

ATOM 3162 N VAL 194 63.182 8.004 43.898 1.00 0.00 N

ATOM 3163 H VAL 194 63.141 7.437 43.063 1.00 0.00 H

ATOM 3164 CA VAL 194 62.057 7.748 44.886 1.00 0.00 C

ATOM 3165 HA VAL 194 61.818 8.773 45.167 1.00 0.00 H

ATOM 3166 CB VAL 194 60.827 7.066 44.311 1.00 0.00 C

ATOM 3167 HB VAL 194 60.052 6.961 45.071 1.00 0.00 H

ATOM 3168 CG1 VAL 194 60.356 7.797 43.024 1.00 0.00 C

ATOM 3169 HG11 VAL 194 59.460 7.349 42.593 1.00 0.00 H

ATOM 3170 HG12 VAL 194 60.067 8.775 43.410 1.00 0.00 H

ATOM 3171 HG13 VAL 194 61.116 7.852 42.244 1.00 0.00 H

ATOM 3172 CG2 VAL 194 61.029 5.628 43.819 1.00 0.00 C

ATOM 3173 HG21 VAL 194 60.120 5.056 43.630 1.00 0.00 H

ATOM 3174 HG22 VAL 194 61.602 5.659 42.892 1.00 0.00 H

ATOM 3175 HG23 VAL 194 61.646 5.056 44.511 1.00 0.00 H

ATOM 3176 C VAL 194 62.473 7.008 46.137 1.00 0.00 C

ATOM 3177 O VAL 194 61.748 7.077 47.123 1.00 0.00 O

ATOM 3178 N LEU 195 63.627 6.309 46.153 1.00 0.00 N

ATOM 3179 H LEU 195 64.059 6.198 45.247 1.00 0.00 H

ATOM 3180 CA LEU 195 64.194 5.503 47.206 1.00 0.00 C

ATOM 3181 HA LEU 195 63.388 5.246 47.893 1.00 0.00 H

ATOM 3182 CB LEU 195 64.957 4.318 46.708 1.00 0.00 C

ATOM 3183 HB2 LEU 195 64.531 3.968 45.768 1.00 0.00 H

ATOM 3184 HB3 LEU 195 65.995 4.583 46.507 1.00 0.00 H

ATOM 3185 CG LEU 195 65.005 3.114 47.659 1.00 0.00 C

ATOM 3186 HG LEU 195 64.239 3.134 48.433 1.00 0.00 H

ATOM 3187 CD1 LEU 195 64.753 1.692 47.117 1.00 0.00 C

ATOM 3188 HD11 LEU 195 65.644 1.481 46.525 1.00 0.00 H

ATOM 3189 HD12 LEU 195 64.638 1.012 47.961 1.00 0.00 H

ATOM 3190 HD13 LEU 195 63.840 1.695 46.522 1.00 0.00 H

ATOM 3191 CD2 LEU 195 66.330 3.181 48.439 1.00 0.00 C

ATOM 3192 HD21 LEU 195 66.660 4.180 48.726 1.00 0.00 H

ATOM 3193 HD22 LEU 195 66.125 2.604 49.341 1.00 0.00 H

ATOM 3194 HD23 LEU 195 67.197 2.737 47.951 1.00 0.00 H

ATOM 3195 C LEU 195 65.067 6.400 48.174 1.00 0.00 C

ATOM 3196 O LEU 195 65.032 6.188 49.389 1.00 0.00 O

ATOM 3197 N ILE 196 65.753 7.418 47.665 1.00 0.00 N

ATOM 3198 H ILE 196 65.903 7.333 46.670 1.00 0.00 H

ATOM 3199 CA ILE 196 66.246 8.566 48.492 1.00 0.00 C

ATOM 3200 HA ILE 196 66.949 8.231 49.254 1.00 0.00 H

ATOM 3201 CB ILE 196 67.031 9.543 47.561 1.00 0.00 C

ATOM 3202 HB ILE 196 66.407 9.603 46.669 1.00 0.00 H

ATOM 3203 CG2 ILE 196 67.036 11.004 48.140 1.00 0.00 C

ATOM 3204 HG21 ILE 196 67.442 11.048 49.151 1.00 0.00 H

ATOM 3205 HG22 ILE 196 67.580 11.694 47.494 1.00 0.00 H

ATOM 3206 HG23 ILE 196 66.022 11.399 48.199 1.00 0.00 H

ATOM 3207 CG1 ILE 196 68.476 9.078 47.273 1.00 0.00 C

ATOM 3208 HG12 ILE 196 69.086 9.341 48.137 1.00 0.00 H

ATOM 3209 HG13 ILE 196 68.379 7.993 47.231 1.00 0.00 H

ATOM 3210 CD1 ILE 196 69.076 9.471 45.920 1.00 0.00 C

ATOM 3211 HD11 ILE 196 68.358 9.172 45.156 1.00 0.00 H

ATOM 3212 HD12 ILE 196 69.120 10.544 45.733 1.00 0.00 H

ATOM 3213 HD13 ILE 196 70.073 9.039 45.829 1.00 0.00 H

ATOM 3214 C ILE 196 65.016 9.140 49.211 1.00 0.00 C

ATOM 3215 O ILE 196 65.166 9.301 50.431 1.00 0.00 O

ATOM 3216 N LEU 197 63.868 9.383 48.516 1.00 0.00 N

ATOM 3217 H LEU 197 63.883 9.167 47.530 1.00 0.00 H

ATOM 3218 CA LEU 197 62.702 10.030 49.077 1.00 0.00 C

ATOM 3219 HA LEU 197 62.991 10.998 49.486 1.00 0.00 H

ATOM 3220 CB LEU 197 61.917 10.376 47.812 1.00 0.00 C

ATOM 3221 HB2 LEU 197 62.078 9.603 47.061 1.00 0.00 H

ATOM 3222 HB3 LEU 197 60.875 10.340 48.128 1.00 0.00 H

ATOM 3223 CG LEU 197 61.970 11.839 47.292 1.00 0.00 C

ATOM 3224 HG LEU 197 61.482 12.585 47.919 1.00 0.00 H

ATOM 3225 CD1 LEU 197 63.396 12.325 47.119 1.00 0.00 C

ATOM 3226 HD11 LEU 197 63.530 13.075 46.339 1.00 0.00 H

ATOM 3227 HD12 LEU 197 63.909 12.634 48.029 1.00 0.00 H

ATOM 3228 HD13 LEU 197 63.902 11.486 46.641 1.00 0.00 H

ATOM 3229 CD2 LEU 197 61.269 11.923 45.912 1.00 0.00 C

ATOM 3230 HD21 LEU 197 60.243 11.644 46.150 1.00 0.00 H

ATOM 3231 HD22 LEU 197 61.248 12.938 45.515 1.00 0.00 H

ATOM 3232 HD23 LEU 197 61.631 11.179 45.203 1.00 0.00 H

ATOM 3233 C LEU 197 61.983 9.205 50.109 1.00 0.00 C

ATOM 3234 O LEU 197 61.475 9.813 51.095 1.00 0.00 O

ATOM 3235 N GLY 198 61.966 7.901 50.020 1.00 0.00 N

ATOM 3236 H GLY 198 62.453 7.511 49.226 1.00 0.00 H

ATOM 3237 CA GLY 198 61.498 6.995 51.100 1.00 0.00 C

ATOM 3238 HA2 GLY 198 60.447 7.166 51.333 1.00 0.00 H

ATOM 3239 HA3 GLY 198 61.409 5.957 50.780 1.00 0.00 H

ATOM 3240 C GLY 198 62.312 7.029 52.329 1.00 0.00 C

ATOM 3241 O GLY 198 61.718 7.247 53.387 1.00 0.00 O

ATOM 3242 N CYX 199 63.594 6.808 52.285 1.00 0.00 N

ATOM 3243 H CYX 199 64.058 6.672 51.398 1.00 0.00 H

ATOM 3244 CA CYX 199 64.487 7.014 53.357 1.00 0.00 C

ATOM 3245 HA CYX 199 63.971 6.241 53.926 1.00 0.00 H

ATOM 3246 CB CYX 199 65.901 6.512 53.101 1.00 0.00 C

ATOM 3247 HB2 CYX 199 66.503 7.186 52.490 1.00 0.00 H

ATOM 3248 HB3 CYX 199 66.452 6.513 54.041 1.00 0.00 H

ATOM 3249 SG CYX 199 65.920 4.923 52.366 1.00 0.00 S

ATOM 3250 C CYX 199 64.462 8.404 53.933 1.00 0.00 C

ATOM 3251 O CYX 199 64.351 8.460 55.139 1.00 0.00 O

ATOM 3252 N TYR 200 64.693 9.493 53.233 1.00 0.00 N

ATOM 3253 H TYR 200 65.113 9.375 52.322 1.00 0.00 H

ATOM 3254 CA TYR 200 64.711 10.819 53.776 1.00 0.00 C

ATOM 3255 HA TYR 200 65.418 10.837 54.605 1.00 0.00 H

ATOM 3256 CB TYR 200 65.390 11.756 52.804 1.00 0.00 C

ATOM 3257 HB2 TYR 200 65.356 11.382 51.781 1.00 0.00 H

ATOM 3258 HB3 TYR 200 64.794 12.666 52.875 1.00 0.00 H

ATOM 3259 CG TYR 200 66.850 12.044 53.189 1.00 0.00 C

ATOM 3260 CD1 TYR 200 67.914 11.586 52.340 1.00 0.00 C

ATOM 3261 HD1 TYR 200 67.681 11.133 51.388 1.00 0.00 H

ATOM 3262 CE1 TYR 200 69.201 11.600 52.797 1.00 0.00 C

ATOM 3263 HE1 TYR 200 70.069 11.223 52.277 1.00 0.00 H

ATOM 3264 CZ TYR 200 69.449 12.196 54.076 1.00 0.00 C

ATOM 3265 OH TYR 200 70.686 12.131 54.613 1.00 0.00 O

ATOM 3266 HH TYR 200 70.862 12.412 55.514 1.00 0.00 H

ATOM 3267 CE2 TYR 200 68.403 12.860 54.765 1.00 0.00 C

ATOM 3268 HE2 TYR 200 68.599 13.449 55.649 1.00 0.00 H

ATOM 3269 CD2 TYR 200 67.121 12.732 54.374 1.00 0.00 C

ATOM 3270 HD2 TYR 200 66.303 13.184 54.915 1.00 0.00 H

ATOM 3271 C TYR 200 63.295 11.251 54.317 1.00 0.00 C

ATOM 3272 O TYR 200 63.225 11.862 55.371 1.00 0.00 O

ATOM 3273 N GLY 201 62.164 10.867 53.703 1.00 0.00 N

ATOM 3274 H GLY 201 62.171 10.189 52.954 1.00 0.00 H

ATOM 3275 CA GLY 201 60.868 11.087 54.306 1.00 0.00 C

ATOM 3276 HA2 GLY 201 60.661 12.145 54.470 1.00 0.00 H

ATOM 3277 HA3 GLY 201 60.216 10.664 53.542 1.00 0.00 H

ATOM 3278 C GLY 201 60.531 10.327 55.560 1.00 0.00 C

ATOM 3279 O GLY 201 59.902 10.872 56.456 1.00 0.00 O

ATOM 3280 N LEU 202 61.268 9.223 55.710 1.00 0.00 N

ATOM 3281 H LEU 202 61.564 8.740 54.874 1.00 0.00 H

ATOM 3282 CA LEU 202 61.423 8.492 56.909 1.00 0.00 C

ATOM 3283 HA LEU 202 60.431 8.396 57.351 1.00 0.00 H

ATOM 3284 CB LEU 202 61.742 7.003 56.698 1.00 0.00 C

ATOM 3285 HB2 LEU 202 61.207 6.785 55.773 1.00 0.00 H

ATOM 3286 HB3 LEU 202 62.827 7.046 56.600 1.00 0.00 H

ATOM 3287 CG LEU 202 61.404 6.040 57.822 1.00 0.00 C

ATOM 3288 HG LEU 202 61.536 5.040 57.409 1.00 0.00 H

ATOM 3289 CD1 LEU 202 62.280 6.284 59.027 1.00 0.00 C

ATOM 3290 HD11 LEU 202 63.234 6.720 58.731 1.00 0.00 H

ATOM 3291 HD12 LEU 202 61.759 7.001 59.661 1.00 0.00 H

ATOM 3292 HD13 LEU 202 62.614 5.345 59.469 1.00 0.00 H

ATOM 3293 CD2 LEU 202 59.912 6.070 58.275 1.00 0.00 C

ATOM 3294 HD21 LEU 202 59.345 6.212 57.355 1.00 0.00 H

ATOM 3295 HD22 LEU 202 59.697 5.116 58.757 1.00 0.00 H

ATOM 3296 HD23 LEU 202 59.624 6.822 59.010 1.00 0.00 H

ATOM 3297 C LEU 202 62.179 9.264 57.918 1.00 0.00 C

ATOM 3298 O LEU 202 61.684 9.462 58.988 1.00 0.00 O

ATOM 3299 N ILE 203 63.480 9.640 57.631 1.00 0.00 N

ATOM 3300 H ILE 203 63.960 9.365 56.786 1.00 0.00 H

ATOM 3301 CA ILE 203 64.332 10.478 58.524 1.00 0.00 C

ATOM 3302 HA ILE 203 64.560 10.096 59.519 1.00 0.00 H

ATOM 3303 CB ILE 203 65.680 10.745 57.838 1.00 0.00 C

ATOM 3304 HB ILE 203 65.572 10.944 56.772 1.00 0.00 H

ATOM 3305 CG2 ILE 203 66.589 11.862 58.385 1.00 0.00 C

ATOM 3306 HG21 ILE 203 65.998 12.735 58.662 1.00 0.00 H

ATOM 3307 HG22 ILE 203 67.196 11.550 59.235 1.00 0.00 H

ATOM 3308 HG23 ILE 203 67.309 12.227 57.653 1.00 0.00 H

ATOM 3309 CG1 ILE 203 66.531 9.384 57.832 1.00 0.00 C

ATOM 3310 HG12 ILE 203 67.095 9.439 58.764 1.00 0.00 H

ATOM 3311 HG13 ILE 203 65.839 8.557 57.989 1.00 0.00 H

ATOM 3312 CD1 ILE 203 67.511 9.225 56.638 1.00 0.00 C

ATOM 3313 HD11 ILE 203 68.208 10.056 56.752 1.00 0.00 H

ATOM 3314 HD12 ILE 203 68.050 8.278 56.627 1.00 0.00 H

ATOM 3315 HD13 ILE 203 66.990 9.291 55.684 1.00 0.00 H

ATOM 3316 C ILE 203 63.527 11.749 58.839 1.00 0.00 C

ATOM 3317 O ILE 203 63.747 12.342 59.886 1.00 0.00 O

ATOM 3318 N VAL 204 62.642 12.312 57.983 1.00 0.00 N

ATOM 3319 H VAL 204 62.558 11.887 57.071 1.00 0.00 H

ATOM 3320 CA VAL 204 61.648 13.337 58.392 1.00 0.00 C

ATOM 3321 HA VAL 204 62.252 14.171 58.749 1.00 0.00 H

ATOM 3322 CB VAL 204 60.813 14.009 57.360 1.00 0.00 C

ATOM 3323 HB VAL 204 60.198 13.283 56.828 1.00 0.00 H

ATOM 3324 CG1 VAL 204 59.821 15.079 57.946 1.00 0.00 C

ATOM 3325 HG11 VAL 204 59.045 14.469 58.409 1.00 0.00 H

ATOM 3326 HG12 VAL 204 60.321 15.767 58.628 1.00 0.00 H

ATOM 3327 HG13 VAL 204 59.459 15.688 57.118 1.00 0.00 H

ATOM 3328 CG2 VAL 204 61.776 14.812 56.456 1.00 0.00 C

ATOM 3329 HG21 VAL 204 62.380 14.158 55.826 1.00 0.00 H

ATOM 3330 HG22 VAL 204 61.211 15.390 55.725 1.00 0.00 H

ATOM 3331 HG23 VAL 204 62.288 15.521 57.107 1.00 0.00 H

ATOM 3332 C VAL 204 60.802 12.846 59.573 1.00 0.00 C

ATOM 3333 O VAL 204 60.833 13.438 60.678 1.00 0.00 O

ATOM 3334 N ARG 205 59.924 11.863 59.248 1.00 0.00 N

ATOM 3335 H ARG 205 60.020 11.404 58.353 1.00 0.00 H

ATOM 3336 CA ARG 205 58.881 11.220 60.064 1.00 0.00 C

ATOM 3337 HA ARG 205 58.073 11.917 60.282 1.00 0.00 H

ATOM 3338 CB ARG 205 58.216 10.035 59.290 1.00 0.00 C

ATOM 3339 HB2 ARG 205 57.965 10.314 58.267 1.00 0.00 H

ATOM 3340 HB3 ARG 205 58.900 9.186 59.287 1.00 0.00 H

ATOM 3341 CG ARG 205 56.948 9.456 59.838 1.00 0.00 C

ATOM 3342 HG2 ARG 205 56.715 8.700 59.089 1.00 0.00 H

ATOM 3343 HG3 ARG 205 57.094 9.059 60.843 1.00 0.00 H

ATOM 3344 CD ARG 205 55.787 10.372 59.862 1.00 0.00 C

ATOM 3345 HD2 ARG 205 55.963 11.152 60.602 1.00 0.00 H

ATOM 3346 HD3 ARG 205 55.675 10.888 58.908 1.00 0.00 H

ATOM 3347 NE ARG 205 54.538 9.752 60.178 1.00 0.00 N

ATOM 3348 HE ARG 205 54.602 8.758 60.346 1.00 0.00 H

ATOM 3349 CZ ARG 205 53.268 10.149 60.256 1.00 0.00 C

ATOM 3350 NH1 ARG 205 52.315 9.362 60.638 1.00 0.00 N

ATOM 3351 HH11 ARG 205 52.568 8.384 60.650 1.00 0.00 H

ATOM 3352 HH12 ARG 205 51.367 9.706 60.696 1.00 0.00 H

ATOM 3353 NH2 ARG 205 53.036 11.331 59.848 1.00 0.00 N

ATOM 3354 HH21 ARG 205 52.049 11.492 59.709 1.00 0.00 H

ATOM 3355 HH22 ARG 205 53.750 11.878 59.389 1.00 0.00 H

ATOM 3356 C ARG 205 59.377 10.834 61.450 1.00 0.00 C

ATOM 3357 O ARG 205 58.567 10.979 62.388 1.00 0.00 O

ATOM 3358 N ALA 206 60.591 10.344 61.628 1.00 0.00 N

ATOM 3359 H ALA 206 60.850 9.805 60.814 1.00 0.00 H

ATOM 3360 CA ALA 206 61.232 10.150 62.980 1.00 0.00 C

ATOM 3361 HA ALA 206 60.316 10.065 63.566 1.00 0.00 H

ATOM 3362 CB ALA 206 61.938 8.762 63.009 1.00 0.00 C

ATOM 3363 HB1 ALA 206 61.282 7.913 63.205 1.00 0.00 H

ATOM 3364 HB2 ALA 206 62.452 8.565 62.069 1.00 0.00 H

ATOM 3365 HB3 ALA 206 62.683 8.790 63.804 1.00 0.00 H

ATOM 3366 C ALA 206 62.055 11.393 63.450 1.00 0.00 C

ATOM 3367 O ALA 206 61.744 12.105 64.421 1.00 0.00 O

ATOM 3368 N LEU 207 63.212 11.590 62.858 1.00 0.00 N

ATOM 3369 H LEU 207 63.187 11.334 61.881 1.00 0.00 H

ATOM 3370 CA LEU 207 64.350 12.443 63.302 1.00 0.00 C

ATOM 3371 HA LEU 207 64.613 12.131 64.313 1.00 0.00 H

ATOM 3372 CB LEU 207 65.633 12.079 62.460 1.00 0.00 C

ATOM 3373 HB2 LEU 207 65.834 11.022 62.636 1.00 0.00 H

ATOM 3374 HB3 LEU 207 65.523 12.255 61.390 1.00 0.00 H

ATOM 3375 CG LEU 207 66.908 12.815 62.836 1.00 0.00 C

ATOM 3376 HG LEU 207 66.649 13.874 62.819 1.00 0.00 H

ATOM 3377 CD1 LEU 207 67.284 12.585 64.301 1.00 0.00 C

ATOM 3378 HD11 LEU 207 68.291 12.975 64.446 1.00 0.00 H

ATOM 3379 HD12 LEU 207 66.630 13.117 64.991 1.00 0.00 H

ATOM 3380 HD13 LEU 207 67.215 11.513 64.488 1.00 0.00 H

ATOM 3381 CD2 LEU 207 68.145 12.684 62.062 1.00 0.00 C

ATOM 3382 HD21 LEU 207 68.398 11.633 61.917 1.00 0.00 H

ATOM 3383 HD22 LEU 207 68.099 13.066 61.043 1.00 0.00 H

ATOM 3384 HD23 LEU 207 69.057 13.099 62.493 1.00 0.00 H

ATOM 3385 C LEU 207 63.863 13.884 63.412 1.00 0.00 C

ATOM 3386 O LEU 207 64.460 14.583 64.212 1.00 0.00 O

ATOM 3387 N ILE 208 62.963 14.394 62.603 1.00 0.00 N

ATOM 3388 H ILE 208 62.622 13.748 61.906 1.00 0.00 H

ATOM 3389 CA ILE 208 62.575 15.758 62.481 1.00 0.00 C

ATOM 3390 HA ILE 208 63.357 16.360 62.945 1.00 0.00 H

ATOM 3391 CB ILE 208 62.561 16.281 60.977 1.00 0.00 C

ATOM 3392 HB ILE 208 62.069 15.500 60.398 1.00 0.00 H

ATOM 3393 CG2 ILE 208 61.748 17.538 60.713 1.00 0.00 C

ATOM 3394 HG21 ILE 208 62.085 18.436 61.231 1.00 0.00 H

ATOM 3395 HG22 ILE 208 61.513 17.681 59.658 1.00 0.00 H

ATOM 3396 HG23 ILE 208 60.702 17.402 60.986 1.00 0.00 H

ATOM 3397 CG1 ILE 208 64.005 16.600 60.419 1.00 0.00 C

ATOM 3398 HG12 ILE 208 64.216 17.630 60.707 1.00 0.00 H

ATOM 3399 HG13 ILE 208 64.798 15.908 60.702 1.00 0.00 H

ATOM 3400 CD1 ILE 208 64.046 16.620 58.955 1.00 0.00 C

ATOM 3401 HD11 ILE 208 63.331 17.257 58.434 1.00 0.00 H

ATOM 3402 HD12 ILE 208 64.994 17.031 58.610 1.00 0.00 H

ATOM 3403 HD13 ILE 208 64.078 15.575 58.649 1.00 0.00 H

ATOM 3404 C ILE 208 61.267 15.970 63.278 1.00 0.00 C

ATOM 3405 O ILE 208 61.265 16.851 64.151 1.00 0.00 O

ATOM 3406 N TYR 209 60.233 15.146 63.005 1.00 0.00 N

ATOM 3407 H TYR 209 60.332 14.408 62.322 1.00 0.00 H

ATOM 3408 CA TYR 209 58.864 15.305 63.526 1.00 0.00 C

ATOM 3409 HA TYR 209 58.581 16.357 63.485 1.00 0.00 H

ATOM 3410 CB TYR 209 58.068 14.566 62.441 1.00 0.00 C

ATOM 3411 HB2 TYR 209 58.376 15.035 61.507 1.00 0.00 H

ATOM 3412 HB3 TYR 209 58.407 13.533 62.369 1.00 0.00 H

ATOM 3413 CG TYR 209 56.569 14.508 62.481 1.00 0.00 C

ATOM 3414 CD1 TYR 209 55.766 15.665 62.288 1.00 0.00 C

ATOM 3415 HD1 TYR 209 56.140 16.612 61.930 1.00 0.00 H

ATOM 3416 CE1 TYR 209 54.409 15.673 62.632 1.00 0.00 C

ATOM 3417 HE1 TYR 209 53.919 16.634 62.681 1.00 0.00 H

ATOM 3418 CZ TYR 209 53.735 14.520 62.980 1.00 0.00 C

ATOM 3419 OH TYR 209 52.478 14.574 63.322 1.00 0.00 O

ATOM 3420 HH TYR 209 52.052 13.731 63.153 1.00 0.00 H

ATOM 3421 CE2 TYR 209 54.484 13.303 63.122 1.00 0.00 C

ATOM 3422 HE2 TYR 209 54.020 12.345 63.302 1.00 0.00 H

ATOM 3423 CD2 TYR 209 55.880 13.282 62.748 1.00 0.00 C

ATOM 3424 HD2 TYR 209 56.415 12.345 62.712 1.00 0.00 H

ATOM 3425 C TYR 209 58.632 14.810 64.994 1.00 0.00 C

ATOM 3426 O TYR 209 57.694 15.339 65.605 1.00 0.00 O

ATOM 3427 N LYS 210 59.494 13.835 65.403 1.00 0.00 N

ATOM 3428 H LYS 210 60.344 13.707 64.873 1.00 0.00 H

ATOM 3429 CA LYS 210 59.340 13.106 66.691 1.00 0.00 C

ATOM 3430 HA LYS 210 58.662 13.698 67.305 1.00 0.00 H

ATOM 3431 CB LYS 210 58.908 11.654 66.394 1.00 0.00 C

ATOM 3432 HB2 LYS 210 59.482 11.430 65.495 1.00 0.00 H

ATOM 3433 HB3 LYS 210 59.254 10.938 67.140 1.00 0.00 H

ATOM 3434 CG LYS 210 57.413 11.487 66.112 1.00 0.00 C

ATOM 3435 HG2 LYS 210 56.754 11.925 66.862 1.00 0.00 H

ATOM 3436 HG3 LYS 210 57.213 12.051 65.202 1.00 0.00 H

ATOM 3437 CD LYS 210 57.013 10.056 65.833 1.00 0.00 C

ATOM 3438 HD2 LYS 210 55.959 10.006 65.562 1.00 0.00 H

ATOM 3439 HD3 LYS 210 57.667 9.600 65.090 1.00 0.00 H

ATOM 3440 CE LYS 210 57.143 9.101 67.049 1.00 0.00 C

ATOM 3441 HE2 LYS 210 58.117 8.622 66.951 1.00 0.00 H

ATOM 3442 HE3 LYS 210 57.303 9.576 68.017 1.00 0.00 H

ATOM 3443 NZ LYS 210 55.959 8.201 67.063 1.00 0.00 N

ATOM 3444 HZ1 LYS 210 56.199 7.746 66.194 1.00 0.00 H

ATOM 3445 HZ2 LYS 210 55.966 7.678 67.927 1.00 0.00 H

ATOM 3446 HZ3 LYS 210 55.143 8.795 67.024 1.00 0.00 H

ATOM 3447 C LYS 210 60.500 12.986 67.641 1.00 0.00 C

ATOM 3448 O LYS 210 60.298 12.849 68.808 1.00 0.00 O

ATOM 3449 N ASP 211 61.727 12.988 67.110 1.00 0.00 N

ATOM 3450 H ASP 211 61.844 12.928 66.109 1.00 0.00 H

ATOM 3451 CA ASP 211 62.855 13.371 67.925 1.00 0.00 C

ATOM 3452 HA ASP 211 62.885 12.548 68.639 1.00 0.00 H

ATOM 3453 CB ASP 211 64.178 13.278 67.162 1.00 0.00 C

ATOM 3454 HB2 ASP 211 64.113 12.649 66.274 1.00 0.00 H

ATOM 3455 HB3 ASP 211 64.413 14.289 66.830 1.00 0.00 H

ATOM 3456 CG ASP 211 65.368 12.927 68.021 1.00 0.00 C

ATOM 3457 OD1 ASP 211 65.184 12.182 69.022 1.00 0.00 O

ATOM 3458 OD2 ASP 211 66.520 13.188 67.603 1.00 0.00 O

ATOM 3459 C ASP 211 62.653 14.593 68.758 1.00 0.00 C

ATOM 3460 O ASP 211 61.926 15.477 68.428 1.00 0.00 O

ATOM 3461 N LEU 212 63.129 14.464 69.987 1.00 0.00 N

ATOM 3462 H LEU 212 63.284 13.498 70.235 1.00 0.00 H

ATOM 3463 CA LEU 212 62.852 15.418 71.049 1.00 0.00 C

ATOM 3464 HA LEU 212 61.943 15.923 70.721 1.00 0.00 H

ATOM 3465 CB LEU 212 62.493 14.605 72.233 1.00 0.00 C

ATOM 3466 HB2 LEU 212 61.747 13.839 72.019 1.00 0.00 H

ATOM 3467 HB3 LEU 212 63.442 14.207 72.592 1.00 0.00 H

ATOM 3468 CG LEU 212 62.004 15.565 73.412 1.00 0.00 C

ATOM 3469 HG LEU 212 61.494 16.366 72.877 1.00 0.00 H

ATOM 3470 CD1 LEU 212 61.102 14.761 74.286 1.00 0.00 C

ATOM 3471 HD11 LEU 212 61.641 13.839 74.504 1.00 0.00 H

ATOM 3472 HD12 LEU 212 60.777 15.310 75.170 1.00 0.00 H

ATOM 3473 HD13 LEU 212 60.172 14.515 73.773 1.00 0.00 H

ATOM 3474 CD2 LEU 212 63.230 16.027 74.261 1.00 0.00 C

ATOM 3475 HD21 LEU 212 64.209 15.725 73.891 1.00 0.00 H

ATOM 3476 HD22 LEU 212 63.454 17.090 74.170 1.00 0.00 H

ATOM 3477 HD23 LEU 212 63.308 15.675 75.290 1.00 0.00 H

ATOM 3478 C LEU 212 64.030 16.386 71.118 1.00 0.00 C

ATOM 3479 O LEU 212 63.794 17.537 71.439 1.00 0.00 O

ATOM 3480 N ASP 213 65.237 15.880 71.166 1.00 0.00 N

ATOM 3481 H ASP 213 65.395 14.882 71.189 1.00 0.00 H

ATOM 3482 CA ASP 213 66.412 16.738 71.023 1.00 0.00 C

ATOM 3483 HA ASP 213 66.411 17.317 71.947 1.00 0.00 H

ATOM 3484 CB ASP 213 67.719 15.949 70.960 1.00 0.00 C

ATOM 3485 HB2 ASP 213 67.750 15.204 71.754 1.00 0.00 H

ATOM 3486 HB3 ASP 213 67.870 15.494 69.980 1.00 0.00 H

ATOM 3487 CG ASP 213 68.881 16.947 71.203 1.00 0.00 C

ATOM 3488 OD1 ASP 213 70.038 16.597 70.846 1.00 0.00 O

ATOM 3489 OD2 ASP 213 68.717 17.994 71.869 1.00 0.00 O

ATOM 3490 C ASP 213 66.403 17.579 69.747 1.00 0.00 C

ATOM 3491 O ASP 213 65.757 17.244 68.756 1.00 0.00 O

ATOM 3492 N ASN 214 67.188 18.634 69.571 1.00 0.00 N

ATOM 3493 H ASN 214 67.747 18.836 70.387 1.00 0.00 H

ATOM 3494 CA ASN 214 67.675 19.171 68.326 1.00 0.00 C

ATOM 3495 HA ASN 214 67.210 18.536 67.572 1.00 0.00 H

ATOM 3496 CB ASN 214 67.168 20.604 68.264 1.00 0.00 C

ATOM 3497 HB2 ASN 214 66.159 20.636 68.676 1.00 0.00 H

ATOM 3498 HB3 ASN 214 67.796 21.217 68.910 1.00 0.00 H

ATOM 3499 CG ASN 214 67.131 21.110 66.860 1.00 0.00 C

ATOM 3500 OD1 ASN 214 66.962 20.414 65.921 1.00 0.00 O

ATOM 3501 ND2 ASN 214 67.266 22.401 66.597 1.00 0.00 N

ATOM 3502 HD21 ASN 214 67.080 23.106 67.296 1.00 0.00 H

ATOM 3503 HD22 ASN 214 67.233 22.683 65.628 1.00 0.00 H

ATOM 3504 C ASN 214 69.158 18.917 68.128 1.00 0.00 C

ATOM 3505 O ASN 214 69.991 19.813 68.362 1.00 0.00 O

ATOM 3506 N SER 215 69.505 17.669 67.714 1.00 0.00 N

ATOM 3507 H SER 215 68.770 17.211 67.194 1.00 0.00 H

ATOM 3508 CA SER 215 70.854 17.226 67.298 1.00 0.00 C

ATOM 3509 HA SER 215 71.590 17.437 68.074 1.00 0.00 H

ATOM 3510 CB SER 215 70.816 15.725 66.910 1.00 0.00 C

ATOM 3511 HB2 SER 215 71.739 15.597 66.345 1.00 0.00 H

ATOM 3512 HB3 SER 215 70.785 15.141 67.830 1.00 0.00 H

ATOM 3513 OG SER 215 69.807 15.336 66.045 1.00 0.00 O

ATOM 3514 HG SER 215 68.970 15.348 66.514 1.00 0.00 H

ATOM 3515 C SER 215 71.292 17.920 65.968 1.00 0.00 C

ATOM 3516 O SER 215 70.464 18.215 65.173 1.00 0.00 O

ATOM 3517 N PRO 216 72.649 17.996 65.711 1.00 0.00 N

ATOM 3518 CD PRO 216 73.665 17.849 66.740 1.00 0.00 C

ATOM 3519 HD2 PRO 216 73.928 16.803 66.896 1.00 0.00 H

ATOM 3520 HD3 PRO 216 73.286 18.469 67.553 1.00 0.00 H

ATOM 3521 CG PRO 216 74.909 18.520 66.208 1.00 0.00 C

ATOM 3522 HG2 PRO 216 75.801 17.943 66.453 1.00 0.00 H

ATOM 3523 HG3 PRO 216 75.085 19.508 66.632 1.00 0.00 H

ATOM 3524 CB PRO 216 74.651 18.577 64.676 1.00 0.00 C

ATOM 3525 HB2 PRO 216 75.084 17.665 64.265 1.00 0.00 H

ATOM 3526 HB3 PRO 216 75.153 19.432 64.222 1.00 0.00 H

ATOM 3527 CA PRO 216 73.181 18.462 64.458 1.00 0.00 C

ATOM 3528 HA PRO 216 72.750 19.461 64.390 1.00 0.00 H

ATOM 3529 C PRO 216 72.796 17.576 63.215 1.00 0.00 C

ATOM 3530 O PRO 216 72.609 18.203 62.146 1.00 0.00 O

ATOM 3531 N LEU 217 72.539 16.289 63.323 1.00 0.00 N

ATOM 3532 H LEU 217 72.994 15.793 64.076 1.00 0.00 H

ATOM 3533 CA LEU 217 71.996 15.483 62.292 1.00 0.00 C

ATOM 3534 HA LEU 217 72.576 15.745 61.407 1.00 0.00 H

ATOM 3535 CB LEU 217 72.265 13.969 62.609 1.00 0.00 C

ATOM 3536 HB2 LEU 217 73.319 13.863 62.866 1.00 0.00 H

ATOM 3537 HB3 LEU 217 71.677 13.660 63.473 1.00 0.00 H

ATOM 3538 CG LEU 217 72.004 12.981 61.379 1.00 0.00 C

ATOM 3539 HG LEU 217 70.956 12.932 61.085 1.00 0.00 H

ATOM 3540 CD1 LEU 217 72.814 13.261 60.207 1.00 0.00 C

ATOM 3541 HD11 LEU 217 72.575 14.255 59.828 1.00 0.00 H

ATOM 3542 HD12 LEU 217 73.862 13.288 60.505 1.00 0.00 H

ATOM 3543 HD13 LEU 217 72.472 12.512 59.493 1.00 0.00 H

ATOM 3544 CD2 LEU 217 72.204 11.608 61.851 1.00 0.00 C

ATOM 3545 HD21 LEU 217 71.577 11.456 62.729 1.00 0.00 H

ATOM 3546 HD22 LEU 217 71.884 11.049 60.970 1.00 0.00 H

ATOM 3547 HD23 LEU 217 73.241 11.360 62.077 1.00 0.00 H

ATOM 3548 C LEU 217 70.505 15.776 61.986 1.00 0.00 C

ATOM 3549 O LEU 217 70.109 15.719 60.840 1.00 0.00 O

ATOM 3550 N ARG 218 69.675 15.988 62.998 1.00 0.00 N

ATOM 3551 H ARG 218 70.055 16.128 63.923 1.00 0.00 H

ATOM 3552 CA ARG 218 68.370 16.614 62.795 1.00 0.00 C

ATOM 3553 HA ARG 218 67.741 16.019 62.133 1.00 0.00 H

ATOM 3554 CB ARG 218 67.599 16.807 64.118 1.00 0.00 C

ATOM 3555 HB2 ARG 218 67.605 15.813 64.567 1.00 0.00 H

ATOM 3556 HB3 ARG 218 68.123 17.483 64.794 1.00 0.00 H

ATOM 3557 CG ARG 218 66.176 17.297 63.914 1.00 0.00 C

ATOM 3558 HG2 ARG 218 66.230 18.273 63.432 1.00 0.00 H

ATOM 3559 HG3 ARG 218 65.652 16.607 63.252 1.00 0.00 H

ATOM 3560 CD ARG 218 65.419 17.428 65.247 1.00 0.00 C

ATOM 3561 HD2 ARG 218 65.288 16.414 65.627 1.00 0.00 H

ATOM 3562 HD3 ARG 218 65.947 18.117 65.906 1.00 0.00 H

ATOM 3563 NE ARG 218 64.049 17.960 64.964 1.00 0.00 N

ATOM 3564 HE ARG 218 63.415 17.180 64.866 1.00 0.00 H

ATOM 3565 CZ ARG 218 63.590 19.144 64.735 1.00 0.00 C

ATOM 3566 NH1 ARG 218 62.367 19.358 64.352 1.00 0.00 N

ATOM 3567 HH11 ARG 218 61.974 20.288 64.357 1.00 0.00 H

ATOM 3568 HH12 ARG 218 61.766 18.659 63.938 1.00 0.00 H

ATOM 3569 NH2 ARG 218 64.340 20.196 64.853 1.00 0.00 N

ATOM 3570 HH21 ARG 218 65.330 20.102 65.033 1.00 0.00 H

ATOM 3571 HH22 ARG 218 63.939 21.120 64.782 1.00 0.00 H

ATOM 3572 C ARG 218 68.472 17.926 62.087 1.00 0.00 C

ATOM 3573 O ARG 218 67.954 18.081 60.974 1.00 0.00 O

ATOM 3574 N ARG 219 69.333 18.824 62.635 1.00 0.00 N

ATOM 3575 H ARG 219 69.652 18.611 63.569 1.00 0.00 H

ATOM 3576 CA ARG 219 69.461 20.264 62.222 1.00 0.00 C

ATOM 3577 HA ARG 219 68.451 20.672 62.174 1.00 0.00 H

ATOM 3578 CB ARG 219 70.346 21.123 63.080 1.00 0.00 C

ATOM 3579 HB2 ARG 219 71.313 20.633 63.190 1.00 0.00 H

ATOM 3580 HB3 ARG 219 70.549 22.056 62.555 1.00 0.00 H

ATOM 3581 CG ARG 219 69.747 21.377 64.483 1.00 0.00 C

ATOM 3582 HG2 ARG 219 68.917 22.064 64.323 1.00 0.00 H

ATOM 3583 HG3 ARG 219 69.289 20.477 64.893 1.00 0.00 H

ATOM 3584 CD ARG 219 70.730 22.001 65.443 1.00 0.00 C

ATOM 3585 HD2 ARG 219 70.375 21.775 66.448 1.00 0.00 H

ATOM 3586 HD3 ARG 219 71.692 21.566 65.172 1.00 0.00 H

ATOM 3587 NE ARG 219 70.815 23.461 65.328 1.00 0.00 N

ATOM 3588 HE ARG 219 70.543 23.917 64.469 1.00 0.00 H

ATOM 3589 CZ ARG 219 71.069 24.327 66.285 1.00 0.00 C

ATOM 3590 NH1 ARG 219 71.340 23.892 67.483 1.00 0.00 N

ATOM 3591 HH11 ARG 219 71.494 24.653 68.130 1.00 0.00 H

ATOM 3592 HH12 ARG 219 71.973 23.106 67.435 1.00 0.00 H

ATOM 3593 NH2 ARG 219 70.817 25.548 66.157 1.00 0.00 N

ATOM 3594 HH21 ARG 219 71.152 26.215 66.838 1.00 0.00 H

ATOM 3595 HH22 ARG 219 70.498 25.854 65.249 1.00 0.00 H

ATOM 3596 C ARG 219 69.946 20.357 60.734 1.00 0.00 C

ATOM 3597 O ARG 219 69.284 21.010 59.939 1.00 0.00 O

ATOM 3598 N LYS 220 70.871 19.477 60.314 1.00 0.00 N

ATOM 3599 H LYS 220 71.463 19.190 61.080 1.00 0.00 H

ATOM 3600 CA LYS 220 71.363 19.210 58.944 1.00 0.00 C

ATOM 3601 HA LYS 220 71.943 20.071 58.613 1.00 0.00 H

ATOM 3602 CB LYS 220 72.382 18.102 58.896 1.00 0.00 C

ATOM 3603 HB2 LYS 220 72.094 17.247 59.508 1.00 0.00 H

ATOM 3604 HB3 LYS 220 72.480 17.841 57.842 1.00 0.00 H

ATOM 3605 CG LYS 220 73.787 18.488 59.365 1.00 0.00 C

ATOM 3606 HG2 LYS 220 74.228 19.098 58.576 1.00 0.00 H

ATOM 3607 HG3 LYS 220 73.762 19.152 60.229 1.00 0.00 H

ATOM 3608 CD LYS 220 74.644 17.228 59.723 1.00 0.00 C

ATOM 3609 HD2 LYS 220 74.243 16.680 60.576 1.00 0.00 H

ATOM 3610 HD3 LYS 220 74.560 16.657 58.799 1.00 0.00 H

ATOM 3611 CE LYS 220 76.236 17.609 59.835 1.00 0.00 C

ATOM 3612 HE2 LYS 220 76.798 16.683 59.708 1.00 0.00 H

ATOM 3613 HE3 LYS 220 76.618 18.293 59.077 1.00 0.00 H

ATOM 3614 NZ LYS 220 76.574 18.169 61.201 1.00 0.00 N

ATOM 3615 HZ1 LYS 220 76.121 17.584 61.888 1.00 0.00 H

ATOM 3616 HZ2 LYS 220 77.561 18.255 61.402 1.00 0.00 H

ATOM 3617 HZ3 LYS 220 76.248 19.120 61.304 1.00 0.00 H

ATOM 3618 C LYS 220 70.259 18.832 57.981 1.00 0.00 C

ATOM 3619 O LYS 220 70.139 19.288 56.828 1.00 0.00 O

ATOM 3620 N SER 221 69.494 17.886 58.455 1.00 0.00 N

ATOM 3621 H SER 221 69.691 17.602 59.404 1.00 0.00 H

ATOM 3622 CA SER 221 68.520 17.218 57.628 1.00 0.00 C

ATOM 3623 HA SER 221 69.027 16.833 56.743 1.00 0.00 H

ATOM 3624 CB SER 221 67.945 16.037 58.402 1.00 0.00 C

ATOM 3625 HB2 SER 221 67.657 16.270 59.427 1.00 0.00 H

ATOM 3626 HB3 SER 221 67.079 15.660 57.859 1.00 0.00 H

ATOM 3627 OG SER 221 68.970 15.063 58.421 1.00 0.00 O

ATOM 3628 HG SER 221 69.611 15.262 59.107 1.00 0.00 H

ATOM 3629 C SER 221 67.435 18.081 57.134 1.00 0.00 C

ATOM 3630 O SER 221 66.915 17.802 56.034 1.00 0.00 O

ATOM 3631 N ILE 222 67.030 19.148 57.866 1.00 0.00 N

ATOM 3632 H ILE 222 67.608 19.292 58.682 1.00 0.00 H

ATOM 3633 CA ILE 222 65.938 20.108 57.592 1.00 0.00 C

ATOM 3634 HA ILE 222 64.989 19.585 57.476 1.00 0.00 H

ATOM 3635 CB ILE 222 65.741 21.126 58.727 1.00 0.00 C

ATOM 3636 HB ILE 222 66.649 21.485 59.212 1.00 0.00 H

ATOM 3637 CG2 ILE 222 65.085 22.426 58.304 1.00 0.00 C

ATOM 3638 HG21 ILE 222 64.180 22.084 57.803 1.00 0.00 H

ATOM 3639 HG22 ILE 222 64.957 23.054 59.186 1.00 0.00 H

ATOM 3640 HG23 ILE 222 65.670 23.094 57.672 1.00 0.00 H

ATOM 3641 CG1 ILE 222 64.886 20.621 59.891 1.00 0.00 C

ATOM 3642 HG12 ILE 222 63.896 20.448 59.470 1.00 0.00 H

ATOM 3643 HG13 ILE 222 65.257 19.665 60.259 1.00 0.00 H

ATOM 3644 CD1 ILE 222 64.847 21.536 61.088 1.00 0.00 C

ATOM 3645 HD11 ILE 222 63.943 22.146 61.066 1.00 0.00 H

ATOM 3646 HD12 ILE 222 64.925 20.843 61.926 1.00 0.00 H

ATOM 3647 HD13 ILE 222 65.704 22.209 61.109 1.00 0.00 H

ATOM 3648 C ILE 222 66.235 20.881 56.367 1.00 0.00 C

ATOM 3649 O ILE 222 65.346 20.997 55.484 1.00 0.00 O

ATOM 3650 N TYR 223 67.431 21.411 56.141 1.00 0.00 N

ATOM 3651 H TYR 223 68.028 21.424 56.956 1.00 0.00 H

ATOM 3652 CA TYR 223 67.975 22.058 55.010 1.00 0.00 C

ATOM 3653 HA TYR 223 67.121 22.636 54.658 1.00 0.00 H

ATOM 3654 CB TYR 223 69.141 22.937 55.434 1.00 0.00 C

ATOM 3655 HB2 TYR 223 69.898 22.440 56.041 1.00 0.00 H

ATOM 3656 HB3 TYR 223 69.643 23.363 54.565 1.00 0.00 H

ATOM 3657 CG TYR 223 68.733 24.081 56.297 1.00 0.00 C

ATOM 3658 CD1 TYR 223 68.758 23.902 57.650 1.00 0.00 C

ATOM 3659 HD1 TYR 223 68.823 22.933 58.121 1.00 0.00 H

ATOM 3660 CE1 TYR 223 68.472 25.000 58.481 1.00 0.00 C

ATOM 3661 HE1 TYR 223 68.541 24.792 59.538 1.00 0.00 H

ATOM 3662 CZ TYR 223 68.050 26.251 57.989 1.00 0.00 C

ATOM 3663 OH TYR 223 68.057 27.360 58.814 1.00 0.00 O

ATOM 3664 HH TYR 223 68.155 27.063 59.721 1.00 0.00 H

ATOM 3665 CE2 TYR 223 67.992 26.379 56.621 1.00 0.00 C

ATOM 3666 HE2 TYR 223 67.588 27.296 56.219 1.00 0.00 H

ATOM 3667 CD2 TYR 223 68.401 25.346 55.738 1.00 0.00 C

ATOM 3668 HD2 TYR 223 68.408 25.402 54.660 1.00 0.00 H

ATOM 3669 C TYR 223 68.163 21.048 53.784 1.00 0.00 C

ATOM 3670 O TYR 223 67.703 21.334 52.671 1.00 0.00 O

ATOM 3671 N LEU 224 68.854 19.943 54.072 1.00 0.00 N

ATOM 3672 H LEU 224 69.442 20.037 54.889 1.00 0.00 H

ATOM 3673 CA LEU 224 69.293 18.893 53.109 1.00 0.00 C

ATOM 3674 HA LEU 224 70.062 19.309 52.458 1.00 0.00 H

ATOM 3675 CB LEU 224 70.095 17.783 53.862 1.00 0.00 C

ATOM 3676 HB2 LEU 224 70.760 18.249 54.590 1.00 0.00 H

ATOM 3677 HB3 LEU 224 69.330 17.347 54.505 1.00 0.00 H

ATOM 3678 CG LEU 224 70.844 16.770 52.968 1.00 0.00 C

ATOM 3679 HG LEU 224 70.206 16.259 52.247 1.00 0.00 H

ATOM 3680 CD1 LEU 224 72.181 17.398 52.365 1.00 0.00 C

ATOM 3681 HD11 LEU 224 72.107 18.093 51.529 1.00 0.00 H

ATOM 3682 HD12 LEU 224 72.783 17.836 53.162 1.00 0.00 H

ATOM 3683 HD13 LEU 224 72.697 16.552 51.911 1.00 0.00 H

ATOM 3684 CD2 LEU 224 71.215 15.544 53.774 1.00 0.00 C

ATOM 3685 HD21 LEU 224 71.430 14.759 53.049 1.00 0.00 H

ATOM 3686 HD22 LEU 224 71.946 15.684 54.570 1.00 0.00 H

ATOM 3687 HD23 LEU 224 70.248 15.211 54.151 1.00 0.00 H

ATOM 3688 C LEU 224 68.206 18.253 52.275 1.00 0.00 C

ATOM 3689 O LEU 224 68.319 18.326 51.060 1.00 0.00 O

ATOM 3690 N VAL 225 67.142 17.733 52.972 1.00 0.00 N

ATOM 3691 H VAL 225 67.157 17.433 53.936 1.00 0.00 H

ATOM 3692 CA VAL 225 65.891 17.460 52.205 1.00 0.00 C

ATOM 3693 HA VAL 225 66.121 16.471 51.809 1.00 0.00 H

ATOM 3694 CB VAL 225 64.729 17.134 53.135 1.00 0.00 C

ATOM 3695 HB VAL 225 63.946 16.897 52.414 1.00 0.00 H

ATOM 3696 CG1 VAL 225 64.782 15.805 53.958 1.00 0.00 C

ATOM 3697 HG11 VAL 225 64.890 14.918 53.334 1.00 0.00 H

ATOM 3698 HG12 VAL 225 65.647 15.859 54.618 1.00 0.00 H

ATOM 3699 HG13 VAL 225 63.952 15.693 54.655 1.00 0.00 H

ATOM 3700 CG2 VAL 225 64.282 18.215 54.205 1.00 0.00 C

ATOM 3701 HG21 VAL 225 63.279 17.904 54.496 1.00 0.00 H

ATOM 3702 HG22 VAL 225 64.919 18.241 55.090 1.00 0.00 H

ATOM 3703 HG23 VAL 225 64.122 19.186 53.737 1.00 0.00 H

ATOM 3704 C VAL 225 65.521 18.496 51.112 1.00 0.00 C

ATOM 3705 O VAL 225 65.194 18.071 49.981 1.00 0.00 O

ATOM 3706 N ILE 226 65.400 19.807 51.424 1.00 0.00 N

ATOM 3707 H ILE 226 65.948 20.155 52.197 1.00 0.00 H

ATOM 3708 CA ILE 226 64.849 20.751 50.481 1.00 0.00 C

ATOM 3709 HA ILE 226 64.027 20.245 49.975 1.00 0.00 H

ATOM 3710 CB ILE 226 64.242 22.068 51.211 1.00 0.00 C

ATOM 3711 HB ILE 226 65.136 22.487 51.673 1.00 0.00 H

ATOM 3712 CG2 ILE 226 63.665 23.155 50.289 1.00 0.00 C

ATOM 3713 HG21 ILE 226 62.645 23.013 49.930 1.00 0.00 H

ATOM 3714 HG22 ILE 226 63.810 24.158 50.690 1.00 0.00 H

ATOM 3715 HG23 ILE 226 64.260 23.241 49.380 1.00 0.00 H

ATOM 3716 CG1 ILE 226 63.102 21.814 52.213 1.00 0.00 C

ATOM 3717 HG12 ILE 226 62.150 21.733 51.687 1.00 0.00 H

ATOM 3718 HG13 ILE 226 63.234 20.889 52.774 1.00 0.00 H

ATOM 3719 CD1 ILE 226 62.800 22.944 53.232 1.00 0.00 C

ATOM 3720 HD11 ILE 226 61.993 22.593 53.875 1.00 0.00 H

ATOM 3721 HD12 ILE 226 63.618 23.177 53.914 1.00 0.00 H

ATOM 3722 HD13 ILE 226 62.484 23.869 52.749 1.00 0.00 H

ATOM 3723 C ILE 226 65.880 21.085 49.424 1.00 0.00 C

ATOM 3724 O ILE 226 65.602 21.229 48.233 1.00 0.00 O

ATOM 3725 N ILE 227 67.141 21.119 49.793 1.00 0.00 N

ATOM 3726 H ILE 227 67.364 20.753 50.707 1.00 0.00 H

ATOM 3727 CA ILE 227 68.262 21.384 48.798 1.00 0.00 C

ATOM 3728 HA ILE 227 67.969 22.259 48.219 1.00 0.00 H

ATOM 3729 CB ILE 227 69.602 21.544 49.479 1.00 0.00 C

ATOM 3730 HB ILE 227 69.823 20.628 50.026 1.00 0.00 H

ATOM 3731 CG2 ILE 227 70.808 21.661 48.539 1.00 0.00 C

ATOM 3732 HG21 ILE 227 71.699 21.788 49.154 1.00 0.00 H

ATOM 3733 HG22 ILE 227 70.894 20.826 47.844 1.00 0.00 H

ATOM 3734 HG23 ILE 227 70.729 22.554 47.919 1.00 0.00 H

ATOM 3735 CG1 ILE 227 69.523 22.893 50.296 1.00 0.00 C

ATOM 3736 HG12 ILE 227 69.473 23.728 49.597 1.00 0.00 H

ATOM 3737 HG13 ILE 227 68.681 22.849 50.987 1.00 0.00 H

ATOM 3738 CD1 ILE 227 70.724 23.034 51.310 1.00 0.00 C

ATOM 3739 HD11 ILE 227 70.459 23.480 52.269 1.00 0.00 H

ATOM 3740 HD12 ILE 227 71.167 22.049 51.459 1.00 0.00 H

ATOM 3741 HD13 ILE 227 71.386 23.754 50.827 1.00 0.00 H

ATOM 3742 C ILE 227 68.378 20.301 47.724 1.00 0.00 C

ATOM 3743 O ILE 227 68.335 20.540 46.533 1.00 0.00 O

ATOM 3744 N VAL 228 68.571 19.047 48.148 1.00 0.00 N

ATOM 3745 H VAL 228 68.404 18.909 49.135 1.00 0.00 H

ATOM 3746 CA VAL 228 68.946 18.037 47.155 1.00 0.00 C

ATOM 3747 HA VAL 228 69.595 18.497 46.410 1.00 0.00 H

ATOM 3748 CB VAL 228 69.481 16.755 47.851 1.00 0.00 C

ATOM 3749 HB VAL 228 68.778 16.320 48.561 1.00 0.00 H

ATOM 3750 CG1 VAL 228 69.990 15.641 46.887 1.00 0.00 C

ATOM 3751 HG11 VAL 228 70.960 15.854 46.437 1.00 0.00 H

ATOM 3752 HG12 VAL 228 70.198 14.828 47.583 1.00 0.00 H

ATOM 3753 HG13 VAL 228 69.244 15.276 46.181 1.00 0.00 H

ATOM 3754 CG2 VAL 228 70.832 17.153 48.604 1.00 0.00 C

ATOM 3755 HG21 VAL 228 71.436 16.294 48.896 1.00 0.00 H

ATOM 3756 HG22 VAL 228 71.526 17.804 48.072 1.00 0.00 H

ATOM 3757 HG23 VAL 228 70.619 17.647 49.552 1.00 0.00 H

ATOM 3758 C VAL 228 67.712 17.763 46.373 1.00 0.00 C

ATOM 3759 O VAL 228 67.782 17.437 45.192 1.00 0.00 O

ATOM 3760 N LEU 229 66.464 17.909 46.939 1.00 0.00 N

ATOM 3761 H LEU 229 66.448 18.234 47.895 1.00 0.00 H

ATOM 3762 CA LEU 229 65.214 17.868 46.148 1.00 0.00 C

ATOM 3763 HA LEU 229 65.337 16.995 45.508 1.00 0.00 H

ATOM 3764 CB LEU 229 63.992 17.669 47.052 1.00 0.00 C

ATOM 3765 HB2 LEU 229 64.201 16.901 47.797 1.00 0.00 H

ATOM 3766 HB3 LEU 229 63.935 18.548 47.694 1.00 0.00 H

ATOM 3767 CG LEU 229 62.584 17.450 46.393 1.00 0.00 C

ATOM 3768 HG LEU 229 62.332 18.284 45.738 1.00 0.00 H

ATOM 3769 CD1 LEU 229 62.617 16.258 45.459 1.00 0.00 C

ATOM 3770 HD11 LEU 229 61.612 15.968 45.152 1.00 0.00 H

ATOM 3771 HD12 LEU 229 63.228 16.602 44.624 1.00 0.00 H

ATOM 3772 HD13 LEU 229 63.074 15.401 45.954 1.00 0.00 H

ATOM 3773 CD2 LEU 229 61.426 17.166 47.412 1.00 0.00 C

ATOM 3774 HD21 LEU 229 61.379 16.096 47.616 1.00 0.00 H

ATOM 3775 HD22 LEU 229 61.699 17.585 48.380 1.00 0.00 H

ATOM 3776 HD23 LEU 229 60.479 17.548 47.032 1.00 0.00 H

ATOM 3777 C LEU 229 64.992 19.041 45.195 1.00 0.00 C

ATOM 3778 O LEU 229 64.490 18.807 44.112 1.00 0.00 O

ATOM 3779 N THR 230 65.499 20.282 45.512 1.00 0.00 N

ATOM 3780 H THR 230 65.924 20.442 46.414 1.00 0.00 H

ATOM 3781 CA THR 230 65.484 21.367 44.465 1.00 0.00 C

ATOM 3782 HA THR 230 64.465 21.447 44.086 1.00 0.00 H

ATOM 3783 CB THR 230 65.943 22.709 45.118 1.00 0.00 C

ATOM 3784 HB THR 230 66.992 22.690 45.411 1.00 0.00 H

ATOM 3785 CG2 THR 230 65.764 23.863 44.095 1.00 0.00 C

ATOM 3786 HG21 THR 230 64.912 23.675 43.441 1.00 0.00 H

ATOM 3787 HG22 THR 230 65.570 24.783 44.647 1.00 0.00 H

ATOM 3788 HG23 THR 230 66.701 23.920 43.542 1.00 0.00 H

ATOM 3789 OG1 THR 230 65.143 22.971 46.282 1.00 0.00 O

ATOM 3790 HG1 THR 230 65.341 22.395 47.025 1.00 0.00 H

ATOM 3791 C THR 230 66.436 21.062 43.295 1.00 0.00 C

ATOM 3792 O THR 230 65.990 21.140 42.184 1.00 0.00 O

ATOM 3793 N VAL 231 67.659 20.598 43.586 1.00 0.00 N

ATOM 3794 H VAL 231 67.937 20.317 44.515 1.00 0.00 H

ATOM 3795 CA VAL 231 68.600 20.060 42.571 1.00 0.00 C

ATOM 3796 HA VAL 231 68.865 20.891 41.918 1.00 0.00 H

ATOM 3797 CB VAL 231 69.924 19.572 43.289 1.00 0.00 C

ATOM 3798 HB VAL 231 69.834 18.750 44.000 1.00 0.00 H

ATOM 3799 CG1 VAL 231 70.843 18.918 42.221 1.00 0.00 C

ATOM 3800 HG11 VAL 231 70.523 17.891 42.046 1.00 0.00 H

ATOM 3801 HG12 VAL 231 70.768 19.370 41.232 1.00 0.00 H

ATOM 3802 HG13 VAL 231 71.866 18.948 42.595 1.00 0.00 H

ATOM 3803 CG2 VAL 231 70.744 20.666 43.936 1.00 0.00 C

ATOM 3804 HG21 VAL 231 71.529 20.189 44.522 1.00 0.00 H

ATOM 3805 HG22 VAL 231 71.176 21.306 43.167 1.00 0.00 H

ATOM 3806 HG23 VAL 231 70.159 21.361 44.538 1.00 0.00 H

ATOM 3807 C VAL 231 67.958 19.065 41.638 1.00 0.00 C

ATOM 3808 O VAL 231 68.086 19.318 40.478 1.00 0.00 O

ATOM 3809 N PHE 232 67.429 17.934 42.110 1.00 0.00 N

ATOM 3810 H PHE 232 67.480 17.854 43.115 1.00 0.00 H

ATOM 3811 CA PHE 232 66.536 17.077 41.253 1.00 0.00 C

ATOM 3812 HA PHE 232 67.120 16.486 40.547 1.00 0.00 H

ATOM 3813 CB PHE 232 65.908 15.998 42.229 1.00 0.00 C

ATOM 3814 HB2 PHE 232 65.288 16.593 42.901 1.00 0.00 H

ATOM 3815 HB3 PHE 232 65.122 15.423 41.739 1.00 0.00 H

ATOM 3816 CG PHE 232 66.829 15.088 42.935 1.00 0.00 C

ATOM 3817 CD1 PHE 232 66.562 14.663 44.288 1.00 0.00 C

ATOM 3818 HD1 PHE 232 65.593 14.867 44.719 1.00 0.00 H

ATOM 3819 CE1 PHE 232 67.460 13.877 44.894 1.00 0.00 C

ATOM 3820 HE1 PHE 232 67.177 13.559 45.886 1.00 0.00 H

ATOM 3821 CZ PHE 232 68.604 13.450 44.285 1.00 0.00 C

ATOM 3822 HZ PHE 232 69.354 12.829 44.751 1.00 0.00 H

ATOM 3823 CE2 PHE 232 68.748 13.635 42.911 1.00 0.00 C

ATOM 3824 HE2 PHE 232 69.477 13.075 42.344 1.00 0.00 H

ATOM 3825 CD2 PHE 232 67.869 14.466 42.258 1.00 0.00 C

ATOM 3826 HD2 PHE 232 68.175 14.753 41.262 1.00 0.00 H

ATOM 3827 C PHE 232 65.441 17.751 40.417 1.00 0.00 C

ATOM 3828 O PHE 232 65.301 17.443 39.211 1.00 0.00 O

ATOM 3829 N ALA 233 64.733 18.823 40.900 1.00 0.00 N

ATOM 3830 H ALA 233 64.985 19.108 41.835 1.00 0.00 H

ATOM 3831 CA ALA 233 63.696 19.531 40.179 1.00 0.00 C

ATOM 3832 HA ALA 233 62.972 18.858 39.719 1.00 0.00 H

ATOM 3833 CB ALA 233 62.925 20.396 41.176 1.00 0.00 C

ATOM 3834 HB1 ALA 233 61.888 20.575 40.893 1.00 0.00 H

ATOM 3835 HB2 ALA 233 62.946 19.988 42.186 1.00 0.00 H

ATOM 3836 HB3 ALA 233 63.319 21.405 41.296 1.00 0.00 H

ATOM 3837 C ALA 233 64.126 20.341 38.992 1.00 0.00 C

ATOM 3838 O ALA 233 63.546 20.265 37.915 1.00 0.00 O

ATOM 3839 N VAL 234 65.195 21.134 39.152 1.00 0.00 N

ATOM 3840 H VAL 234 65.658 21.079 40.048 1.00 0.00 H

ATOM 3841 CA VAL 234 65.649 22.051 38.058 1.00 0.00 C

ATOM 3842 HA VAL 234 64.769 22.505 37.603 1.00 0.00 H

ATOM 3843 CB VAL 234 66.335 23.333 38.769 1.00 0.00 C

ATOM 3844 HB VAL 234 66.787 23.961 38.002 1.00 0.00 H

ATOM 3845 CG1 VAL 234 65.320 24.201 39.497 1.00 0.00 C

ATOM 3846 HG11 VAL 234 64.512 24.473 38.818 1.00 0.00 H

ATOM 3847 HG12 VAL 234 65.053 23.630 40.386 1.00 0.00 H

ATOM 3848 HG13 VAL 234 65.798 25.080 39.929 1.00 0.00 H

ATOM 3849 CG2 VAL 234 67.585 22.943 39.574 1.00 0.00 C

ATOM 3850 HG21 VAL 234 68.476 23.035 38.952 1.00 0.00 H

ATOM 3851 HG22 VAL 234 67.573 23.636 40.415 1.00 0.00 H

ATOM 3852 HG23 VAL 234 67.509 21.897 39.872 1.00 0.00 H

ATOM 3853 C VAL 234 66.655 21.349 37.114 1.00 0.00 C

ATOM 3854 O VAL 234 66.732 21.752 35.970 1.00 0.00 O

ATOM 3855 N SER 235 67.429 20.330 37.638 1.00 0.00 N

ATOM 3856 H SER 235 67.260 20.229 38.628 1.00 0.00 H

ATOM 3857 CA SER 235 68.353 19.481 36.748 1.00 0.00 C

ATOM 3858 HA SER 235 68.687 20.078 35.899 1.00 0.00 H

ATOM 3859 CB SER 235 69.669 19.274 37.488 1.00 0.00 C

ATOM 3860 HB2 SER 235 69.434 18.778 38.430 1.00 0.00 H

ATOM 3861 HB3 SER 235 70.216 18.548 36.885 1.00 0.00 H

ATOM 3862 OG SER 235 70.354 20.486 37.641 1.00 0.00 O

ATOM 3863 HG SER 235 71.110 20.437 37.051 1.00 0.00 H

ATOM 3864 C SER 235 67.700 18.195 36.357 1.00 0.00 C

ATOM 3865 O SER 235 67.078 18.211 35.334 1.00 0.00 O

ATOM 3866 N TYR 236 67.676 17.203 37.236 1.00 0.00 N

ATOM 3867 H TYR 236 68.007 17.478 38.150 1.00 0.00 H

ATOM 3868 CA TYR 236 67.704 15.774 36.871 1.00 0.00 C

ATOM 3869 HA TYR 236 68.258 15.838 35.935 1.00 0.00 H

ATOM 3870 CB TYR 236 68.477 15.003 37.941 1.00 0.00 C

ATOM 3871 HB2 TYR 236 67.792 14.985 38.789 1.00 0.00 H

ATOM 3872 HB3 TYR 236 68.665 14.002 37.553 1.00 0.00 H

ATOM 3873 CG TYR 236 69.795 15.670 38.161 1.00 0.00 C

ATOM 3874 CD1 TYR 236 70.760 15.478 37.120 1.00 0.00 C

ATOM 3875 HD1 TYR 236 70.476 14.987 36.201 1.00 0.00 H

ATOM 3876 CE1 TYR 236 71.938 16.235 37.215 1.00 0.00 C

ATOM 3877 HE1 TYR 236 72.767 16.012 36.560 1.00 0.00 H

ATOM 3878 CZ TYR 236 72.280 16.956 38.300 1.00 0.00 C

ATOM 3879 OH TYR 236 73.491 17.544 38.354 1.00 0.00 O

ATOM 3880 HH TYR 236 74.137 17.093 37.805 1.00 0.00 H

ATOM 3881 CE2 TYR 236 71.402 17.064 39.383 1.00 0.00 C

ATOM 3882 HE2 TYR 236 71.753 17.563 40.274 1.00 0.00 H

ATOM 3883 CD2 TYR 236 70.107 16.497 39.260 1.00 0.00 C

ATOM 3884 HD2 TYR 236 69.385 16.567 40.060 1.00 0.00 H

ATOM 3885 C TYR 236 66.399 15.067 36.318 1.00 0.00 C

ATOM 3886 O TYR 236 66.486 14.100 35.569 1.00 0.00 O

ATOM 3887 N ILE 237 65.228 15.657 36.624 1.00 0.00 N

ATOM 3888 H ILE 237 65.277 16.543 37.106 1.00 0.00 H

ATOM 3889 CA ILE 237 63.901 15.326 36.043 1.00 0.00 C

ATOM 3890 HA ILE 237 63.880 14.257 35.832 1.00 0.00 H

ATOM 3891 CB ILE 237 62.843 15.715 37.119 1.00 0.00 C

ATOM 3892 HB ILE 237 63.170 16.670 37.528 1.00 0.00 H

ATOM 3893 CG2 ILE 237 61.363 15.683 36.634 1.00 0.00 C

ATOM 3894 HG21 ILE 237 61.102 16.579 36.070 1.00 0.00 H

ATOM 3895 HG22 ILE 237 61.185 14.858 35.944 1.00 0.00 H

ATOM 3896 HG23 ILE 237 60.685 15.540 37.476 1.00 0.00 H

ATOM 3897 CG1 ILE 237 62.917 14.842 38.381 1.00 0.00 C

ATOM 3898 HG12 ILE 237 62.233 14.002 38.261 1.00 0.00 H

ATOM 3899 HG13 ILE 237 63.927 14.455 38.516 1.00 0.00 H

ATOM 3900 CD1 ILE 237 62.556 15.520 39.710 1.00 0.00 C

ATOM 3901 HD11 ILE 237 61.476 15.626 39.812 1.00 0.00 H

ATOM 3902 HD12 ILE 237 63.071 14.914 40.455 1.00 0.00 H

ATOM 3903 HD13 ILE 237 62.977 16.525 39.708 1.00 0.00 H

ATOM 3904 C ILE 237 63.602 16.047 34.675 1.00 0.00 C

ATOM 3905 O ILE 237 63.387 15.364 33.647 1.00 0.00 O

ATOM 3906 N PRO 238 63.810 17.349 34.518 1.00 0.00 N

ATOM 3907 CD PRO 238 63.756 18.318 35.585 1.00 0.00 C

ATOM 3908 HD2 PRO 238 64.540 18.137 36.320 1.00 0.00 H

ATOM 3909 HD3 PRO 238 62.775 18.325 36.061 1.00 0.00 H

ATOM 3910 CG PRO 238 63.873 19.717 34.958 1.00 0.00 C

ATOM 3911 HG2 PRO 238 64.862 20.070 35.249 1.00 0.00 H

ATOM 3912 HG3 PRO 238 63.085 20.388 35.301 1.00 0.00 H

ATOM 3913 CB PRO 238 63.856 19.476 33.418 1.00 0.00 C

ATOM 3914 HB2 PRO 238 64.624 19.981 32.832 1.00 0.00 H

ATOM 3915 HB3 PRO 238 62.965 19.958 33.014 1.00 0.00 H

ATOM 3916 CA PRO 238 63.847 18.021 33.219 1.00 0.00 C

ATOM 3917 HA PRO 238 62.885 17.757 32.781 1.00 0.00 H

ATOM 3918 C PRO 238 64.918 17.467 32.304 1.00 0.00 C

ATOM 3919 O PRO 238 64.651 17.407 31.112 1.00 0.00 O

ATOM 3920 N PHE 239 66.146 17.147 32.803 1.00 0.00 N

ATOM 3921 H PHE 239 66.370 17.600 33.678 1.00 0.00 H

ATOM 3922 CA PHE 239 67.069 16.302 32.015 1.00 0.00 C

ATOM 3923 HA PHE 239 67.276 16.950 31.163 1.00 0.00 H

ATOM 3924 CB PHE 239 68.341 16.007 32.859 1.00 0.00 C

ATOM 3925 HB2 PHE 239 68.845 16.950 33.074 1.00 0.00 H

ATOM 3926 HB3 PHE 239 68.212 15.477 33.803 1.00 0.00 H

ATOM 3927 CG PHE 239 69.399 15.358 31.949 1.00 0.00 C

ATOM 3928 CD1 PHE 239 69.916 15.985 30.808 1.00 0.00 C

ATOM 3929 HD1 PHE 239 69.311 16.812 30.466 1.00 0.00 H

ATOM 3930 CE1 PHE 239 70.953 15.377 30.043 1.00 0.00 C

ATOM 3931 HE1 PHE 239 71.354 15.908 29.192 1.00 0.00 H

ATOM 3932 CZ PHE 239 71.482 14.113 30.388 1.00 0.00 C

ATOM 3933 HZ PHE 239 72.333 13.683 29.880 1.00 0.00 H

ATOM 3934 CE2 PHE 239 70.869 13.455 31.437 1.00 0.00 C

ATOM 3935 HE2 PHE 239 71.085 12.427 31.689 1.00 0.00 H

ATOM 3936 CD2 PHE 239 69.883 14.105 32.186 1.00 0.00 C

ATOM 3937 HD2 PHE 239 69.342 13.633 32.993 1.00 0.00 H

ATOM 3938 C PHE 239 66.477 15.082 31.326 1.00 0.00 C

ATOM 3939 O PHE 239 66.507 14.963 30.090 1.00 0.00 O

ATOM 3940 N HID 240 66.055 14.159 32.150 1.00 0.00 N

ATOM 3941 H HID 240 66.025 14.425 33.124 1.00 0.00 H

ATOM 3942 CA HID 240 65.552 12.879 31.720 1.00 0.00 C

ATOM 3943 HA HID 240 66.370 12.430 31.156 1.00 0.00 H

ATOM 3944 CB HID 240 65.218 11.988 32.907 1.00 0.00 C

ATOM 3945 HB2 HID 240 64.784 12.458 33.790 1.00 0.00 H

ATOM 3946 HB3 HID 240 64.547 11.184 32.604 1.00 0.00 H

ATOM 3947 CG HID 240 66.508 11.341 33.275 1.00 0.00 C

ATOM 3948 ND1 HID 240 67.373 11.759 34.313 1.00 0.00 N

ATOM 3949 HD1 HID 240 67.284 12.568 34.911 1.00 0.00 H

ATOM 3950 CE1 HID 240 68.550 11.084 34.287 1.00 0.00 C

ATOM 3951 HE1 HID 240 69.392 11.251 34.943 1.00 0.00 H

ATOM 3952 NE2 HID 240 68.501 10.158 33.316 1.00 0.00 N

ATOM 3953 CD2 HID 240 67.243 10.430 32.621 1.00 0.00 C

ATOM 3954 HD2 HID 240 66.998 9.821 31.763 1.00 0.00 H

ATOM 3955 C HID 240 64.355 13.012 30.796 1.00 0.00 C

ATOM 3956 O HID 240 64.347 12.459 29.723 1.00 0.00 O

ATOM 3957 N VAL 241 63.348 13.830 31.204 1.00 0.00 N

ATOM 3958 H VAL 241 63.398 14.175 32.152 1.00 0.00 H

ATOM 3959 CA VAL 241 62.242 14.163 30.340 1.00 0.00 C

ATOM 3960 HA VAL 241 61.684 13.232 30.242 1.00 0.00 H

ATOM 3961 CB VAL 241 61.236 15.103 31.018 1.00 0.00 C

ATOM 3962 HB VAL 241 61.725 16.014 31.362 1.00 0.00 H

ATOM 3963 CG1 VAL 241 60.140 15.530 30.003 1.00 0.00 C

ATOM 3964 HG11 VAL 241 59.563 14.703 29.589 1.00 0.00 H

ATOM 3965 HG12 VAL 241 59.538 16.144 30.672 1.00 0.00 H

ATOM 3966 HG13 VAL 241 60.503 16.037 29.108 1.00 0.00 H

ATOM 3967 CG2 VAL 241 60.597 14.363 32.175 1.00 0.00 C

ATOM 3968 HG21 VAL 241 61.147 14.548 33.098 1.00 0.00 H

ATOM 3969 HG22 VAL 241 59.568 14.684 32.337 1.00 0.00 H

ATOM 3970 HG23 VAL 241 60.532 13.275 32.164 1.00 0.00 H

ATOM 3971 C VAL 241 62.612 14.661 28.932 1.00 0.00 C

ATOM 3972 O VAL 241 62.287 14.053 27.920 1.00 0.00 O

ATOM 3973 N MET 242 63.319 15.822 28.911 1.00 0.00 N

ATOM 3974 H MET 242 63.543 16.278 29.784 1.00 0.00 H

ATOM 3975 CA MET 242 63.782 16.495 27.671 1.00 0.00 C

ATOM 3976 HA MET 242 62.809 16.690 27.219 1.00 0.00 H

ATOM 3977 CB MET 242 64.504 17.779 27.990 1.00 0.00 C

ATOM 3978 HB2 MET 242 64.075 18.306 28.842 1.00 0.00 H

ATOM 3979 HB3 MET 242 65.511 17.475 28.273 1.00 0.00 H

ATOM 3980 CG MET 242 64.610 18.617 26.744 1.00 0.00 C

ATOM 3981 HG2 MET 242 65.188 19.507 26.991 1.00 0.00 H

ATOM 3982 HG3 MET 242 65.162 18.223 25.890 1.00 0.00 H

ATOM 3983 SD MET 242 62.924 19.261 26.468 1.00 0.00 S

ATOM 3984 CE MET 242 62.854 18.663 24.749 1.00 0.00 C

ATOM 3985 HE1 MET 242 63.288 17.669 24.642 1.00 0.00 H

ATOM 3986 HE2 MET 242 61.864 18.693 24.295 1.00 0.00 H

ATOM 3987 HE3 MET 242 63.468 19.339 24.154 1.00 0.00 H

ATOM 3988 C MET 242 64.579 15.460 26.789 1.00 0.00 C

ATOM 3989 O MET 242 64.313 15.406 25.587 1.00 0.00 O

ATOM 3990 N LYS 243 65.415 14.623 27.483 1.00 0.00 N

ATOM 3991 H LYS 243 65.554 14.792 28.469 1.00 0.00 H

ATOM 3992 CA LYS 243 66.225 13.507 26.855 1.00 0.00 C

ATOM 3993 HA LYS 243 66.835 13.996 26.096 1.00 0.00 H

ATOM 3994 CB LYS 243 67.117 12.861 27.819 1.00 0.00 C

ATOM 3995 HB2 LYS 243 67.552 13.650 28.432 1.00 0.00 H

ATOM 3996 HB3 LYS 243 66.675 12.120 28.485 1.00 0.00 H

ATOM 3997 CG LYS 243 68.296 12.153 27.092 1.00 0.00 C

ATOM 3998 HG2 LYS 243 67.932 11.320 26.490 1.00 0.00 H

ATOM 3999 HG3 LYS 243 68.778 12.839 26.395 1.00 0.00 H

ATOM 4000 CD LYS 243 69.282 11.622 28.035 1.00 0.00 C

ATOM 4001 HD2 LYS 243 69.462 12.348 28.827 1.00 0.00 H

ATOM 4002 HD3 LYS 243 68.879 10.710 28.476 1.00 0.00 H

ATOM 4003 CE LYS 243 70.574 11.193 27.351 1.00 0.00 C

ATOM 4004 HE2 LYS 243 70.337 11.101 26.291 1.00 0.00 H

ATOM 4005 HE3 LYS 243 71.250 12.047 27.385 1.00 0.00 H

ATOM 4006 NZ LYS 243 71.175 10.005 27.926 1.00 0.00 N

ATOM 4007 HZ1 LYS 243 70.711 9.715 28.775 1.00 0.00 H

ATOM 4008 HZ2 LYS 243 71.196 9.240 27.267 1.00 0.00 H

ATOM 4009 HZ3 LYS 243 72.145 10.140 28.172 1.00 0.00 H

ATOM 4010 C LYS 243 65.398 12.423 26.233 1.00 0.00 C

ATOM 4011 O LYS 243 65.652 12.121 25.083 1.00 0.00 O

ATOM 4012 N THR 244 64.257 12.161 26.835 1.00 0.00 N

ATOM 4013 H THR 244 64.094 12.647 27.705 1.00 0.00 H

ATOM 4014 CA THR 244 63.339 10.996 26.454 1.00 0.00 C

ATOM 4015 HA THR 244 63.930 10.116 26.199 1.00 0.00 H

ATOM 4016 CB THR 244 62.438 10.615 27.560 1.00 0.00 C

ATOM 4017 HB THR 244 61.680 11.396 27.625 1.00 0.00 H

ATOM 4018 CG2 THR 244 61.634 9.346 27.289 1.00 0.00 C

ATOM 4019 HG21 THR 244 62.257 8.452 27.243 1.00 0.00 H

ATOM 4020 HG22 THR 244 60.908 9.171 28.083 1.00 0.00 H

ATOM 4021 HG23 THR 244 61.149 9.404 26.314 1.00 0.00 H

ATOM 4022 OG1 THR 244 63.184 10.293 28.778 1.00 0.00 O

ATOM 4023 HG1 THR 244 63.661 11.093 29.010 1.00 0.00 H

ATOM 4024 C THR 244 62.421 11.472 25.244 1.00 0.00 C

ATOM 4025 O THR 244 62.482 10.944 24.131 1.00 0.00 O

ATOM 4026 N MET 245 61.942 12.781 25.361 1.00 0.00 N

ATOM 4027 H MET 245 61.920 13.177 26.290 1.00 0.00 H

ATOM 4028 CA MET 245 61.441 13.581 24.290 1.00 0.00 C

ATOM 4029 HA MET 245 60.492 13.172 23.943 1.00 0.00 H

ATOM 4030 CB MET 245 61.086 14.948 24.795 1.00 0.00 C

ATOM 4031 HB2 MET 245 61.873 15.179 25.512 1.00 0.00 H

ATOM 4032 HB3 MET 245 61.117 15.714 24.019 1.00 0.00 H

ATOM 4033 CG MET 245 59.685 15.065 25.510 1.00 0.00 C

ATOM 4034 HG2 MET 245 58.956 14.471 24.958 1.00 0.00 H

ATOM 4035 HG3 MET 245 59.848 14.614 26.489 1.00 0.00 H

ATOM 4036 SD MET 245 59.076 16.800 25.516 1.00 0.00 S

ATOM 4037 CE MET 245 58.313 16.832 23.853 1.00 0.00 C

ATOM 4038 HE1 MET 245 58.942 16.414 23.067 1.00 0.00 H

ATOM 4039 HE2 MET 245 57.536 16.077 23.736 1.00 0.00 H

ATOM 4040 HE3 MET 245 57.994 17.852 23.642 1.00 0.00 H

ATOM 4041 C MET 245 62.409 13.588 23.121 1.00 0.00 C

ATOM 4042 O MET 245 62.004 13.210 22.016 1.00 0.00 O

ATOM 4043 N ASN 246 63.698 13.888 23.320 1.00 0.00 N

ATOM 4044 H ASN 246 63.958 14.095 24.274 1.00 0.00 H

ATOM 4045 CA ASN 246 64.727 13.848 22.279 1.00 0.00 C

ATOM 4046 HA ASN 246 64.411 14.488 21.456 1.00 0.00 H

ATOM 4047 CB ASN 246 66.023 14.513 22.865 1.00 0.00 C

ATOM 4048 HB2 ASN 246 66.141 15.489 22.394 1.00 0.00 H

ATOM 4049 HB3 ASN 246 65.961 14.507 23.953 1.00 0.00 H

ATOM 4050 CG ASN 246 67.259 13.727 22.436 1.00 0.00 C

ATOM 4051 OD1 ASN 246 68.063 14.295 21.717 1.00 0.00 O

ATOM 4052 ND2 ASN 246 67.547 12.613 23.027 1.00 0.00 N

ATOM 4053 HD21 ASN 246 68.526 12.420 23.182 1.00 0.00 H

ATOM 4054 HD22 ASN 246 66.996 12.362 23.836 1.00 0.00 H

ATOM 4055 C ASN 246 64.908 12.509 21.611 1.00 0.00 C

ATOM 4056 O ASN 246 65.158 12.437 20.429 1.00 0.00 O

ATOM 4057 N LEU 247 64.681 11.365 22.294 1.00 0.00 N

ATOM 4058 H LEU 247 64.560 11.437 23.294 1.00 0.00 H

ATOM 4059 CA LEU 247 64.761 9.976 21.786 1.00 0.00 C

ATOM 4060 HA LEU 247 65.603 9.848 21.105 1.00 0.00 H

ATOM 4061 CB LEU 247 65.002 9.070 22.946 1.00 0.00 C

ATOM 4062 HB2 LEU 247 64.246 9.301 23.697 1.00 0.00 H

ATOM 4063 HB3 LEU 247 64.704 8.102 22.543 1.00 0.00 H

ATOM 4064 CG LEU 247 66.430 8.980 23.546 1.00 0.00 C

ATOM 4065 HG LEU 247 66.911 9.939 23.350 1.00 0.00 H

ATOM 4066 CD1 LEU 247 66.389 8.858 25.037 1.00 0.00 C

ATOM 4067 HD11 LEU 247 65.884 7.932 25.310 1.00 0.00 H

ATOM 4068 HD12 LEU 247 67.408 9.061 25.367 1.00 0.00 H

ATOM 4069 HD13 LEU 247 65.728 9.620 25.450 1.00 0.00 H

ATOM 4070 CD2 LEU 247 67.328 7.829 22.979 1.00 0.00 C

ATOM 4071 HD21 LEU 247 66.879 6.892 23.309 1.00 0.00 H

ATOM 4072 HD22 LEU 247 67.262 7.841 21.891 1.00 0.00 H

ATOM 4073 HD23 LEU 247 68.347 7.828 23.365 1.00 0.00 H

ATOM 4074 C LEU 247 63.565 9.557 20.889 1.00 0.00 C

ATOM 4075 O LEU 247 63.792 9.158 19.703 1.00 0.00 O

ATOM 4076 N ARG 248 62.399 10.094 21.238 1.00 0.00 N

ATOM 4077 H ARG 248 62.233 10.242 22.224 1.00 0.00 H

ATOM 4078 CA ARG 248 61.176 9.912 20.413 1.00 0.00 C

ATOM 4079 HA ARG 248 61.112 8.844 20.203 1.00 0.00 H

ATOM 4080 CB ARG 248 59.949 10.296 21.248 1.00 0.00 C

ATOM 4081 HB2 ARG 248 59.924 9.697 22.159 1.00 0.00 H

ATOM 4082 HB3 ARG 248 59.984 11.314 21.636 1.00 0.00 H

ATOM 4083 CG ARG 248 58.578 10.128 20.778 1.00 0.00 C

ATOM 4084 HG2 ARG 248 58.189 9.245 21.284 1.00 0.00 H

ATOM 4085 HG3 ARG 248 58.043 11.026 21.086 1.00 0.00 H

ATOM 4086 CD ARG 248 58.298 10.011 19.238 1.00 0.00 C

ATOM 4087 HD2 ARG 248 58.759 10.808 18.654 1.00 0.00 H

ATOM 4088 HD3 ARG 248 58.663 9.035 18.918 1.00 0.00 H

ATOM 4089 NE ARG 248 56.815 9.985 19.099 1.00 0.00 N

ATOM 4090 HE ARG 248 56.332 10.832 19.364 1.00 0.00 H

ATOM 4091 CZ ARG 248 56.118 8.898 18.771 1.00 0.00 C

ATOM 4092 NH1 ARG 248 56.680 7.797 18.408 1.00 0.00 N

ATOM 4093 HH11 ARG 248 56.059 7.006 18.314 1.00 0.00 H

ATOM 4094 HH12 ARG 248 57.593 7.501 18.723 1.00 0.00 H

ATOM 4095 NH2 ARG 248 54.796 8.891 18.819 1.00 0.00 N

ATOM 4096 HH21 ARG 248 54.431 9.777 19.140 1.00 0.00 H

ATOM 4097 HH22 ARG 248 54.328 7.997 18.861 1.00 0.00 H

ATOM 4098 C ARG 248 61.349 10.835 19.177 1.00 0.00 C

ATOM 4099 O ARG 248 61.063 10.343 18.093 1.00 0.00 O

ATOM 4100 N ALA 249 61.775 12.117 19.354 1.00 0.00 N

ATOM 4101 H ALA 249 61.799 12.392 20.326 1.00 0.00 H

ATOM 4102 CA ALA 249 61.781 13.176 18.396 1.00 0.00 C

ATOM 4103 HA ALA 249 60.792 13.442 18.023 1.00 0.00 H

ATOM 4104 CB ALA 249 62.358 14.439 19.081 1.00 0.00 C

ATOM 4105 HB1 ALA 249 62.470 15.209 18.318 1.00 0.00 H

ATOM 4106 HB2 ALA 249 61.583 14.692 19.805 1.00 0.00 H

ATOM 4107 HB3 ALA 249 63.295 14.057 19.485 1.00 0.00 H

ATOM 4108 C ALA 249 62.642 12.841 17.156 1.00 0.00 C

ATOM 4109 O ALA 249 62.291 12.984 16.014 1.00 0.00 O

ATOM 4110 N ARG 250 63.828 12.317 17.483 1.00 0.00 N

ATOM 4111 H ARG 250 64.066 12.273 18.464 1.00 0.00 H

ATOM 4112 CA ARG 250 64.820 11.947 16.506 1.00 0.00 C

ATOM 4113 HA ARG 250 64.908 12.704 15.726 1.00 0.00 H

ATOM 4114 CB ARG 250 66.190 11.782 17.162 1.00 0.00 C

ATOM 4115 HB2 ARG 250 66.075 11.105 18.009 1.00 0.00 H

ATOM 4116 HB3 ARG 250 66.868 11.252 16.493 1.00 0.00 H

ATOM 4117 CG ARG 250 66.777 13.138 17.616 1.00 0.00 C

ATOM 4118 HG2 ARG 250 67.225 13.593 16.732 1.00 0.00 H

ATOM 4119 HG3 ARG 250 66.038 13.922 17.781 1.00 0.00 H

ATOM 4120 CD ARG 250 67.748 13.234 18.748 1.00 0.00 C

ATOM 4121 HD2 ARG 250 67.521 14.214 19.168 1.00 0.00 H

ATOM 4122 HD3 ARG 250 67.482 12.595 19.590 1.00 0.00 H

ATOM 4123 NE ARG 250 69.101 13.103 18.367 1.00 0.00 N

ATOM 4124 HE ARG 250 69.323 12.931 17.397 1.00 0.00 H

ATOM 4125 CZ ARG 250 70.176 13.228 19.138 1.00 0.00 C

ATOM 4126 NH1 ARG 250 70.341 13.464 20.399 1.00 0.00 N

ATOM 4127 HH11 ARG 250 69.521 13.582 20.977 1.00 0.00 H

ATOM 4128 HH12 ARG 250 71.205 13.293 20.893 1.00 0.00 H

ATOM 4129 NH2 ARG 250 71.256 12.782 18.685 1.00 0.00 N

ATOM 4130 HH21 ARG 250 72.007 12.523 19.309 1.00 0.00 H

ATOM 4131 HH22 ARG 250 71.162 12.131 17.918 1.00 0.00 H

ATOM 4132 C ARG 250 64.316 10.768 15.719 1.00 0.00 C

ATOM 4133 O ARG 250 64.761 10.526 14.611 1.00 0.00 O

ATOM 4134 N LEU 251 63.497 9.897 16.344 1.00 0.00 N

ATOM 4135 H LEU 251 63.475 9.947 17.353 1.00 0.00 H

ATOM 4136 CA LEU 251 62.950 8.721 15.647 1.00 0.00 C

ATOM 4137 HA LEU 251 63.693 8.319 14.959 1.00 0.00 H

ATOM 4138 CB LEU 251 62.565 7.767 16.678 1.00 0.00 C

ATOM 4139 HB2 LEU 251 63.447 7.642 17.306 1.00 0.00 H

ATOM 4140 HB3 LEU 251 61.805 8.218 17.316 1.00 0.00 H

ATOM 4141 CG LEU 251 62.089 6.402 16.232 1.00 0.00 C

ATOM 4142 HG LEU 251 61.090 6.592 15.839 1.00 0.00 H

ATOM 4143 CD1 LEU 251 62.761 5.776 15.054 1.00 0.00 C

ATOM 4144 HD11 LEU 251 62.489 6.272 14.122 1.00 0.00 H

ATOM 4145 HD12 LEU 251 63.833 5.642 15.195 1.00 0.00 H

ATOM 4146 HD13 LEU 251 62.472 4.732 14.933 1.00 0.00 H

ATOM 4147 CD2 LEU 251 62.002 5.438 17.450 1.00 0.00 C

ATOM 4148 HD21 LEU 251 63.033 5.203 17.716 1.00 0.00 H

ATOM 4149 HD22 LEU 251 61.571 5.911 18.332 1.00 0.00 H

ATOM 4150 HD23 LEU 251 61.466 4.557 17.097 1.00 0.00 H

ATOM 4151 C LEU 251 61.796 9.192 14.696 1.00 0.00 C

ATOM 4152 O LEU 251 61.694 8.697 13.551 1.00 0.00 O

ATOM 4153 N ASP 252 60.915 10.069 15.199 1.00 0.00 N

ATOM 4154 H ASP 252 60.964 10.058 16.208 1.00 0.00 H

ATOM 4155 CA ASP 252 59.564 10.138 14.687 1.00 0.00 C

ATOM 4156 HA ASP 252 59.352 9.353 13.961 1.00 0.00 H

ATOM 4157 CB ASP 252 58.669 9.970 15.906 1.00 0.00 C

ATOM 4158 HB2 ASP 252 59.012 9.154 16.542 1.00 0.00 H

ATOM 4159 HB3 ASP 252 58.771 10.909 16.450 1.00 0.00 H

ATOM 4160 CG ASP 252 57.215 9.811 15.504 1.00 0.00 C

ATOM 4161 OD1 ASP 252 56.472 10.805 15.433 1.00 0.00 O

ATOM 4162 OD2 ASP 252 56.747 8.700 15.266 1.00 0.00 O

ATOM 4163 C ASP 252 59.322 11.487 14.004 1.00 0.00 C

ATOM 4164 O ASP 252 58.591 11.471 12.959 1.00 0.00 O

ATOM 4165 N PHE 253 59.871 12.577 14.549 1.00 0.00 N

ATOM 4166 H PHE 253 60.596 12.509 15.249 1.00 0.00 H

ATOM 4167 CA PHE 253 59.606 13.969 14.085 1.00 0.00 C

ATOM 4168 HA PHE 253 58.604 14.144 13.693 1.00 0.00 H

ATOM 4169 CB PHE 253 59.758 14.966 15.259 1.00 0.00 C

ATOM 4170 HB2 PHE 253 60.797 14.905 15.583 1.00 0.00 H

ATOM 4171 HB3 PHE 253 59.398 15.943 14.936 1.00 0.00 H

ATOM 4172 CG PHE 253 58.954 14.716 16.496 1.00 0.00 C

ATOM 4173 CD1 PHE 253 58.003 13.634 16.623 1.00 0.00 C

ATOM 4174 HD1 PHE 253 57.706 12.976 15.819 1.00 0.00 H

ATOM 4175 CE1 PHE 253 57.498 13.325 17.917 1.00 0.00 C

ATOM 4176 HE1 PHE 253 56.902 12.425 17.947 1.00 0.00 H

ATOM 4177 CZ PHE 253 57.706 14.279 19.000 1.00 0.00 C

ATOM 4178 HZ PHE 253 57.411 14.033 20.009 1.00 0.00 H

ATOM 4179 CE2 PHE 253 58.445 15.443 18.796 1.00 0.00 C

ATOM 4180 HE2 PHE 253 58.631 16.052 19.669 1.00 0.00 H

ATOM 4181 CD2 PHE 253 59.149 15.602 17.583 1.00 0.00 C

ATOM 4182 HD2 PHE 253 59.594 16.570 17.404 1.00 0.00 H

ATOM 4183 C PHE 253 60.371 14.342 12.849 1.00 0.00 C

ATOM 4184 O PHE 253 61.181 15.287 12.794 1.00 0.00 O

ATOM 4185 N GLN 254 60.126 13.586 11.744 1.00 0.00 N

ATOM 4186 H GLN 254 59.437 12.866 11.907 1.00 0.00 H

ATOM 4187 CA GLN 254 60.929 13.529 10.546 1.00 0.00 C

ATOM 4188 HA GLN 254 61.942 13.902 10.700 1.00 0.00 H

ATOM 4189 CB GLN 254 61.129 12.047 10.077 1.00 0.00 C

ATOM 4190 HB2 GLN 254 60.210 11.513 9.834 1.00 0.00 H

ATOM 4191 HB3 GLN 254 61.852 11.911 9.272 1.00 0.00 H

ATOM 4192 CG GLN 254 61.934 11.347 11.154 1.00 0.00 C

ATOM 4193 HG2 GLN 254 62.794 11.883 11.555 1.00 0.00 H

ATOM 4194 HG3 GLN 254 61.401 11.005 12.041 1.00 0.00 H

ATOM 4195 CD GLN 254 62.549 10.071 10.748 1.00 0.00 C

ATOM 4196 OE1 GLN 254 63.766 9.934 10.672 1.00 0.00 O

ATOM 4197 NE2 GLN 254 61.791 9.104 10.367 1.00 0.00 N

ATOM 4198 HE21 GLN 254 62.314 8.253 10.215 1.00 0.00 H

ATOM 4199 HE22 GLN 254 60.826 9.019 10.650 1.00 0.00 H

ATOM 4200 C GLN 254 60.362 14.329 9.269 1.00 0.00 C

ATOM 4201 O GLN 254 61.177 14.723 8.482 1.00 0.00 O

ATOM 4202 N THR 255 59.016 14.619 9.267 1.00 0.00 N

ATOM 4203 H THR 255 58.332 14.430 9.985 1.00 0.00 H

ATOM 4204 CA THR 255 58.386 15.626 8.295 1.00 0.00 C

ATOM 4205 HA THR 255 58.766 15.406 7.298 1.00 0.00 H

ATOM 4206 CB THR 255 56.910 15.629 8.304 1.00 0.00 C

ATOM 4207 HB THR 255 56.515 16.521 7.819 1.00 0.00 H

ATOM 4208 CG2 THR 255 56.374 14.420 7.523 1.00 0.00 C

ATOM 4209 HG21 THR 255 56.146 14.646 6.481 1.00 0.00 H

ATOM 4210 HG22 THR 255 57.043 13.560 7.553 1.00 0.00 H

ATOM 4211 HG23 THR 255 55.508 13.961 7.999 1.00 0.00 H

ATOM 4212 OG1 THR 255 56.239 15.687 9.551 1.00 0.00 O

ATOM 4213 HG1 THR 255 56.365 14.831 9.965 1.00 0.00 H

ATOM 4214 C THR 255 58.844 17.117 8.541 1.00 0.00 C

ATOM 4215 O THR 255 59.158 17.400 9.703 1.00 0.00 O

ATOM 4216 N PRO 256 58.817 17.971 7.475 1.00 0.00 N

ATOM 4217 CD PRO 256 58.533 17.599 6.111 1.00 0.00 C

ATOM 4218 HD2 PRO 256 57.472 17.362 6.186 1.00 0.00 H

ATOM 4219 HD3 PRO 256 59.107 16.713 5.840 1.00 0.00 H

ATOM 4220 CG PRO 256 58.892 18.831 5.241 1.00 0.00 C

ATOM 4221 HG2 PRO 256 58.267 18.917 4.352 1.00 0.00 H

ATOM 4222 HG3 PRO 256 59.898 18.696 4.843 1.00 0.00 H

ATOM 4223 CB PRO 256 58.966 19.997 6.249 1.00 0.00 C

ATOM 4224 HB2 PRO 256 57.990 20.480 6.191 1.00 0.00 H

ATOM 4225 HB3 PRO 256 59.788 20.674 6.014 1.00 0.00 H

ATOM 4226 CA PRO 256 59.340 19.294 7.552 1.00 0.00 C

ATOM 4227 HA PRO 256 60.417 19.215 7.406 1.00 0.00 H

ATOM 4228 C PRO 256 59.022 20.137 8.886 1.00 0.00 C

ATOM 4229 O PRO 256 60.005 20.680 9.412 1.00 0.00 O

ATOM 4230 N ALA 257 57.733 20.310 9.295 1.00 0.00 N

ATOM 4231 H ALA 257 56.985 19.912 8.744 1.00 0.00 H

ATOM 4232 CA ALA 257 57.383 21.092 10.484 1.00 0.00 C

ATOM 4233 HA ALA 257 57.920 22.040 10.456 1.00 0.00 H

ATOM 4234 CB ALA 257 55.906 21.426 10.612 1.00 0.00 C

ATOM 4235 HB1 ALA 257 55.693 21.810 11.609 1.00 0.00 H

ATOM 4236 HB2 ALA 257 55.634 22.172 9.865 1.00 0.00 H

ATOM 4237 HB3 ALA 257 55.335 20.512 10.448 1.00 0.00 H

ATOM 4238 C ALA 257 57.876 20.355 11.744 1.00 0.00 C

ATOM 4239 O ALA 257 58.324 20.933 12.763 1.00 0.00 O

ATOM 4240 N MET 258 57.752 19.051 11.761 1.00 0.00 N

ATOM 4241 H MET 258 57.308 18.644 10.950 1.00 0.00 H

ATOM 4242 CA MET 258 58.053 18.216 12.881 1.00 0.00 C

ATOM 4243 HA MET 258 57.477 18.607 13.719 1.00 0.00 H

ATOM 4244 CB MET 258 57.432 16.840 12.695 1.00 0.00 C

ATOM 4245 HB2 MET 258 57.876 16.335 11.838 1.00 0.00 H

ATOM 4246 HB3 MET 258 57.658 16.237 13.574 1.00 0.00 H

ATOM 4247 CG MET 258 55.914 16.842 12.551 1.00 0.00 C

ATOM 4248 HG2 MET 258 55.623 17.746 12.015 1.00 0.00 H

ATOM 4249 HG3 MET 258 55.444 16.011 12.026 1.00 0.00 H

ATOM 4250 SD MET 258 55.109 17.260 14.069 1.00 0.00 S

ATOM 4251 CE MET 258 55.011 15.558 14.720 1.00 0.00 C

ATOM 4252 HE1 MET 258 55.986 15.165 15.009 1.00 0.00 H

ATOM 4253 HE2 MET 258 54.395 15.729 15.602 1.00 0.00 H

ATOM 4254 HE3 MET 258 54.604 14.933 13.925 1.00 0.00 H

ATOM 4255 C MET 258 59.507 18.229 13.123 1.00 0.00 C

ATOM 4256 O MET 258 59.897 18.245 14.277 1.00 0.00 O

ATOM 4257 N CYX 259 60.393 18.199 12.114 1.00 0.00 N

ATOM 4258 H CYX 259 59.907 18.105 11.234 1.00 0.00 H

ATOM 4259 CA CYX 259 61.821 18.371 12.139 1.00 0.00 C

ATOM 4260 HA CYX 259 62.166 17.464 12.635 1.00 0.00 H

ATOM 4261 CB CYX 259 62.463 18.385 10.727 1.00 0.00 C

ATOM 4262 HB2 CYX 259 61.932 17.698 10.068 1.00 0.00 H

ATOM 4263 HB3 CYX 259 62.306 19.395 10.348 1.00 0.00 H

ATOM 4264 SG CYX 259 64.241 18.117 10.736 1.00 0.00 S

ATOM 4265 C CYX 259 62.271 19.577 12.927 1.00 0.00 C

ATOM 4266 O CYX 259 63.147 19.508 13.786 1.00 0.00 O

ATOM 4267 N ALA 260 61.613 20.736 12.641 1.00 0.00 N

ATOM 4268 H ALA 260 61.050 20.849 11.810 1.00 0.00 H

ATOM 4269 CA ALA 260 61.760 21.900 13.492 1.00 0.00 C

ATOM 4270 HA ALA 260 62.782 22.276 13.463 1.00 0.00 H

ATOM 4271 CB ALA 260 60.947 23.095 12.826 1.00 0.00 C

ATOM 4272 HB1 ALA 260 61.639 23.887 12.540 1.00 0.00 H

ATOM 4273 HB2 ALA 260 60.393 22.755 11.951 1.00 0.00 H

ATOM 4274 HB3 ALA 260 60.334 23.519 13.621 1.00 0.00 H

ATOM 4275 C ALA 260 61.374 21.775 14.930 1.00 0.00 C

ATOM 4276 O ALA 260 62.076 22.134 15.864 1.00 0.00 O

ATOM 4277 N PHE 261 60.276 21.053 15.251 1.00 0.00 N

ATOM 4278 H PHE 261 59.767 20.596 14.508 1.00 0.00 H

ATOM 4279 CA PHE 261 59.936 20.691 16.609 1.00 0.00 C

ATOM 4280 HA PHE 261 59.770 21.538 17.275 1.00 0.00 H

ATOM 4281 CB PHE 261 58.691 19.846 16.552 1.00 0.00 C

ATOM 4282 HB2 PHE 261 58.068 20.239 15.748 1.00 0.00 H

ATOM 4283 HB3 PHE 261 58.928 18.807 16.325 1.00 0.00 H

ATOM 4284 CG PHE 261 57.766 20.100 17.826 1.00 0.00 C

ATOM 4285 CD1 PHE 261 56.646 20.960 17.765 1.00 0.00 C

ATOM 4286 HD1 PHE 261 56.427 21.406 16.806 1.00 0.00 H

ATOM 4287 CE1 PHE 261 56.123 21.483 18.953 1.00 0.00 C

ATOM 4288 HE1 PHE 261 55.418 22.301 18.919 1.00 0.00 H

ATOM 4289 CZ PHE 261 56.660 21.062 20.207 1.00 0.00 C

ATOM 4290 HZ PHE 261 56.249 21.432 21.134 1.00 0.00 H

ATOM 4291 CE2 PHE 261 57.841 20.254 20.265 1.00 0.00 C

ATOM 4292 HE2 PHE 261 58.386 19.917 21.135 1.00 0.00 H

ATOM 4293 CD2 PHE 261 58.266 19.646 19.062 1.00 0.00 C

ATOM 4294 HD2 PHE 261 59.185 19.078 19.089 1.00 0.00 H

ATOM 4295 C PHE 261 61.081 19.831 17.262 1.00 0.00 C

ATOM 4296 O PHE 261 61.516 20.239 18.368 1.00 0.00 O

ATOM 4297 N ASN 262 61.586 18.856 16.525 1.00 0.00 N

ATOM 4298 H ASN 262 61.022 18.656 15.712 1.00 0.00 H

ATOM 4299 CA ASN 262 62.708 18.032 16.884 1.00 0.00 C

ATOM 4300 HA ASN 262 62.542 17.521 17.833 1.00 0.00 H

ATOM 4301 CB ASN 262 62.917 17.067 15.673 1.00 0.00 C

ATOM 4302 HB2 ASN 262 62.464 16.136 16.016 1.00 0.00 H

ATOM 4303 HB3 ASN 262 62.394 17.455 14.799 1.00 0.00 H

ATOM 4304 CG ASN 262 64.375 16.732 15.381 1.00 0.00 C

ATOM 4305 OD1 ASN 262 64.976 15.801 15.883 1.00 0.00 O

ATOM 4306 ND2 ASN 262 64.873 17.409 14.377 1.00 0.00 N

ATOM 4307 HD21 ASN 262 64.251 18.093 13.971 1.00 0.00 H

ATOM 4308 HD22 ASN 262 65.724 17.094 13.933 1.00 0.00 H

ATOM 4309 C ASN 262 63.975 18.933 17.037 1.00 0.00 C

ATOM 4310 O ASN 262 64.942 18.487 17.606 1.00 0.00 O

ATOM 4311 N ASP 263 64.084 20.159 16.377 1.00 0.00 N

ATOM 4312 H ASP 263 63.271 20.446 15.852 1.00 0.00 H

ATOM 4313 CA ASP 263 65.265 21.056 16.528 1.00 0.00 C

ATOM 4314 HA ASP 263 66.188 20.541 16.795 1.00 0.00 H

ATOM 4315 CB ASP 263 65.675 21.764 15.200 1.00 0.00 C

ATOM 4316 HB2 ASP 263 66.192 21.022 14.591 1.00 0.00 H

ATOM 4317 HB3 ASP 263 64.784 22.065 14.649 1.00 0.00 H

ATOM 4318 CG ASP 263 66.664 22.975 15.437 1.00 0.00 C

ATOM 4319 OD1 ASP 263 66.262 24.101 15.756 1.00 0.00 O

ATOM 4320 OD2 ASP 263 67.864 22.678 15.226 1.00 0.00 O

ATOM 4321 C ASP 263 65.237 21.980 17.771 1.00 0.00 C

ATOM 4322 O ASP 263 66.235 22.047 18.576 1.00 0.00 O

ATOM 4323 N ARG 264 64.088 22.521 18.047 1.00 0.00 N

ATOM 4324 H ARG 264 63.405 22.649 17.313 1.00 0.00 H

ATOM 4325 CA ARG 264 63.758 23.223 19.323 1.00 0.00 C

ATOM 4326 HA ARG 264 64.612 23.847 19.589 1.00 0.00 H

ATOM 4327 CB ARG 264 62.484 23.959 19.069 1.00 0.00 C

ATOM 4328 HB2 ARG 264 61.770 23.231 18.682 1.00 0.00 H

ATOM 4329 HB3 ARG 264 62.136 24.407 20.000 1.00 0.00 H

ATOM 4330 CG ARG 264 62.672 25.055 18.098 1.00 0.00 C

ATOM 4331 HG2 ARG 264 63.275 25.911 18.401 1.00 0.00 H

ATOM 4332 HG3 ARG 264 63.056 24.649 17.162 1.00 0.00 H

ATOM 4333 CD ARG 264 61.413 25.679 17.514 1.00 0.00 C

ATOM 4334 HD2 ARG 264 61.721 26.459 16.818 1.00 0.00 H

ATOM 4335 HD3 ARG 264 60.851 24.891 17.013 1.00 0.00 H

ATOM 4336 NE ARG 264 60.685 26.272 18.613 1.00 0.00 N

ATOM 4337 HE ARG 264 60.599 25.756 19.477 1.00 0.00 H

ATOM 4338 CZ ARG 264 59.827 27.262 18.467 1.00 0.00 C

ATOM 4339 NH1 ARG 264 58.996 27.466 19.434 1.00 0.00 N

ATOM 4340 HH11 ARG 264 58.318 28.214 19.413 1.00 0.00 H

ATOM 4341 HH12 ARG 264 58.988 26.823 20.213 1.00 0.00 H

ATOM 4342 NH2 ARG 264 59.822 28.059 17.468 1.00 0.00 N

ATOM 4343 HH21 ARG 264 58.947 28.497 17.218 1.00 0.00 H

ATOM 4344 HH22 ARG 264 60.678 28.322 17.002 1.00 0.00 H

ATOM 4345 C ARG 264 63.660 22.274 20.548 1.00 0.00 C

ATOM 4346 O ARG 264 64.132 22.693 21.603 1.00 0.00 O

ATOM 4347 N VAL 265 63.403 21.007 20.213 1.00 0.00 N

ATOM 4348 H VAL 265 63.034 20.834 19.289 1.00 0.00 H

ATOM 4349 CA VAL 265 63.529 19.897 21.153 1.00 0.00 C

ATOM 4350 HA VAL 265 63.021 20.223 22.061 1.00 0.00 H

ATOM 4351 CB VAL 265 62.798 18.632 20.714 1.00 0.00 C

ATOM 4352 HB VAL 265 63.003 18.676 19.644 1.00 0.00 H

ATOM 4353 CG1 VAL 265 63.382 17.271 21.322 1.00 0.00 C

ATOM 4354 HG11 VAL 265 63.598 17.450 22.375 1.00 0.00 H

ATOM 4355 HG12 VAL 265 62.568 16.581 21.100 1.00 0.00 H

ATOM 4356 HG13 VAL 265 64.100 16.949 20.567 1.00 0.00 H

ATOM 4357 CG2 VAL 265 61.260 18.797 21.077 1.00 0.00 C

ATOM 4358 HG21 VAL 265 61.066 18.704 22.146 1.00 0.00 H

ATOM 4359 HG22 VAL 265 60.904 19.766 20.727 1.00 0.00 H

ATOM 4360 HG23 VAL 265 60.673 18.049 20.544 1.00 0.00 H

ATOM 4361 C VAL 265 64.973 19.729 21.496 1.00 0.00 C

ATOM 4362 O VAL 265 65.364 19.749 22.697 1.00 0.00 O

ATOM 4363 N TYR 266 65.854 19.689 20.451 1.00 0.00 N

ATOM 4364 H TYR 266 65.548 19.777 19.493 1.00 0.00 H

ATOM 4365 CA TYR 266 67.262 19.443 20.685 1.00 0.00 C

ATOM 4366 HA TYR 266 67.408 18.634 21.401 1.00 0.00 H

ATOM 4367 CB TYR 266 67.865 19.065 19.311 1.00 0.00 C

ATOM 4368 HB2 TYR 266 67.345 18.197 18.907 1.00 0.00 H

ATOM 4369 HB3 TYR 266 67.786 19.874 18.584 1.00 0.00 H

ATOM 4370 CG TYR 266 69.360 18.726 19.534 1.00 0.00 C

ATOM 4371 CD1 TYR 266 70.357 19.490 18.915 1.00 0.00 C

ATOM 4372 HD1 TYR 266 70.051 20.379 18.382 1.00 0.00 H

ATOM 4373 CE1 TYR 266 71.672 18.945 18.866 1.00 0.00 C

ATOM 4374 HE1 TYR 266 72.369 19.471 18.231 1.00 0.00 H

ATOM 4375 CZ TYR 266 72.014 17.721 19.555 1.00 0.00 C

ATOM 4376 OH TYR 266 73.326 17.304 19.681 1.00 0.00 O

ATOM 4377 HH TYR 266 73.346 16.468 20.154 1.00 0.00 H

ATOM 4378 CE2 TYR 266 70.998 17.007 20.221 1.00 0.00 C

ATOM 4379 HE2 TYR 266 71.109 16.024 20.655 1.00 0.00 H

ATOM 4380 CD2 TYR 266 69.688 17.476 20.162 1.00 0.00 C

ATOM 4381 HD2 TYR 266 68.886 16.862 20.544 1.00 0.00 H

ATOM 4382 C TYR 266 67.961 20.638 21.349 1.00 0.00 C

ATOM 4383 O TYR 266 68.765 20.418 22.240 1.00 0.00 O

ATOM 4384 N ALA 267 67.642 21.839 20.968 1.00 0.00 N

ATOM 4385 H ALA 267 67.116 21.882 20.108 1.00 0.00 H

ATOM 4386 CA ALA 267 68.080 23.079 21.602 1.00 0.00 C

ATOM 4387 HA ALA 267 69.156 23.067 21.431 1.00 0.00 H

ATOM 4388 CB ALA 267 67.590 24.266 20.876 1.00 0.00 C

ATOM 4389 HB1 ALA 267 67.655 24.075 19.805 1.00 0.00 H

ATOM 4390 HB2 ALA 267 66.534 24.351 21.130 1.00 0.00 H

ATOM 4391 HB3 ALA 267 68.185 25.095 21.259 1.00 0.00 H

ATOM 4392 C ALA 267 67.783 23.057 23.074 1.00 0.00 C

ATOM 4393 O ALA 267 68.698 23.334 23.825 1.00 0.00 O

ATOM 4394 N THR 268 66.572 22.877 23.475 1.00 0.00 N

ATOM 4395 H THR 268 65.802 23.118 22.867 1.00 0.00 H

ATOM 4396 CA THR 268 66.196 22.574 24.863 1.00 0.00 C

ATOM 4397 HA THR 268 66.428 23.441 25.482 1.00 0.00 H

ATOM 4398 CB THR 268 64.634 22.308 24.895 1.00 0.00 C

ATOM 4399 HB THR 268 64.383 21.349 24.442 1.00 0.00 H

ATOM 4400 CG2 THR 268 64.016 22.434 26.346 1.00 0.00 C

ATOM 4401 HG21 THR 268 62.944 22.307 26.194 1.00 0.00 H

ATOM 4402 HG22 THR 268 64.378 21.678 27.043 1.00 0.00 H

ATOM 4403 HG23 THR 268 64.311 23.410 26.732 1.00 0.00 H

ATOM 4404 OG1 THR 268 63.941 23.216 24.190 1.00 0.00 O

ATOM 4405 HG1 THR 268 64.099 22.899 23.297 1.00 0.00 H

ATOM 4406 C THR 268 66.893 21.448 25.552 1.00 0.00 C

ATOM 4407 O THR 268 67.151 21.394 26.775 1.00 0.00 O

ATOM 4408 N TYR 269 67.290 20.420 24.764 1.00 0.00 N

ATOM 4409 H TYR 269 67.150 20.501 23.767 1.00 0.00 H

ATOM 4410 CA TYR 269 67.954 19.200 25.327 1.00 0.00 C

ATOM 4411 HA TYR 269 67.365 18.858 26.177 1.00 0.00 H

ATOM 4412 CB TYR 269 67.921 18.039 24.333 1.00 0.00 C

ATOM 4413 HB2 TYR 269 66.916 17.642 24.192 1.00 0.00 H

ATOM 4414 HB3 TYR 269 68.157 18.285 23.298 1.00 0.00 H

ATOM 4415 CG TYR 269 68.876 16.859 24.690 1.00 0.00 C

ATOM 4416 CD1 TYR 269 68.871 16.224 25.944 1.00 0.00 C

ATOM 4417 HD1 TYR 269 68.104 16.508 26.649 1.00 0.00 H

ATOM 4418 CE1 TYR 269 69.829 15.262 26.263 1.00 0.00 C

ATOM 4419 HE1 TYR 269 69.860 14.791 27.234 1.00 0.00 H

ATOM 4420 CZ TYR 269 70.720 14.725 25.308 1.00 0.00 C

ATOM 4421 OH TYR 269 71.417 13.604 25.521 1.00 0.00 O

ATOM 4422 HH TYR 269 71.723 13.294 24.666 1.00 0.00 H

ATOM 4423 CE2 TYR 269 70.541 15.128 23.998 1.00 0.00 C

ATOM 4424 HE2 TYR 269 71.069 14.589 23.225 1.00 0.00 H

ATOM 4425 CD2 TYR 269 69.657 16.259 23.714 1.00 0.00 C

ATOM 4426 HD2 TYR 269 69.425 16.578 22.709 1.00 0.00 H

ATOM 4427 C TYR 269 69.448 19.521 25.731 1.00 0.00 C

ATOM 4428 O TYR 269 69.825 19.312 26.822 1.00 0.00 O

ATOM 4429 N GLN 270 70.192 20.144 24.829 1.00 0.00 N

ATOM 4430 H GLN 270 69.751 20.392 23.956 1.00 0.00 H

ATOM 4431 CA GLN 270 71.457 20.817 25.079 1.00 0.00 C

ATOM 4432 HA GLN 270 72.187 20.056 25.356 1.00 0.00 H

ATOM 4433 CB GLN 270 71.930 21.442 23.784 1.00 0.00 C

ATOM 4434 HB2 GLN 270 71.218 22.232 23.544 1.00 0.00 H

ATOM 4435 HB3 GLN 270 72.808 22.070 23.935 1.00 0.00 H

ATOM 4436 CG GLN 270 72.175 20.435 22.675 1.00 0.00 C

ATOM 4437 HG2 GLN 270 71.415 19.655 22.636 1.00 0.00 H

ATOM 4438 HG3 GLN 270 72.186 21.047 21.774 1.00 0.00 H

ATOM 4439 CD GLN 270 73.496 19.725 22.795 1.00 0.00 C

ATOM 4440 OE1 GLN 270 73.802 19.093 23.830 1.00 0.00 O

ATOM 4441 NE2 GLN 270 74.231 19.558 21.763 1.00 0.00 N

ATOM 4442 HE21 GLN 270 73.988 20.092 20.941 1.00 0.00 H

ATOM 4443 HE22 GLN 270 75.178 19.221 21.852 1.00 0.00 H

ATOM 4444 C GLN 270 71.482 21.772 26.305 1.00 0.00 C

ATOM 4445 O GLN 270 72.416 21.673 27.201 1.00 0.00 O

ATOM 4446 N VAL 271 70.511 22.735 26.549 1.00 0.00 N

ATOM 4447 H VAL 271 69.862 23.059 25.846 1.00 0.00 H

ATOM 4448 CA VAL 271 70.611 23.641 27.766 1.00 0.00 C

ATOM 4449 HA VAL 271 71.650 23.925 27.931 1.00 0.00 H

ATOM 4450 CB VAL 271 69.948 25.015 27.564 1.00 0.00 C

ATOM 4451 HB VAL 271 70.272 25.554 28.454 1.00 0.00 H

ATOM 4452 CG1 VAL 271 70.445 25.618 26.291 1.00 0.00 C

ATOM 4453 HG11 VAL 271 70.233 25.069 25.373 1.00 0.00 H

ATOM 4454 HG12 VAL 271 69.957 26.592 26.255 1.00 0.00 H

ATOM 4455 HG13 VAL 271 71.530 25.720 26.334 1.00 0.00 H

ATOM 4456 CG2 VAL 271 68.446 24.945 27.390 1.00 0.00 C

ATOM 4457 HG21 VAL 271 67.968 24.639 28.320 1.00 0.00 H

ATOM 4458 HG22 VAL 271 68.024 25.868 26.992 1.00 0.00 H

ATOM 4459 HG23 VAL 271 68.239 24.131 26.695 1.00 0.00 H

ATOM 4460 C VAL 271 70.233 22.979 29.119 1.00 0.00 C

ATOM 4461 O VAL 271 70.788 23.246 30.214 1.00 0.00 O

ATOM 4462 N THR 272 69.453 21.965 29.035 1.00 0.00 N

ATOM 4463 H THR 272 69.255 21.527 28.147 1.00 0.00 H

ATOM 4464 CA THR 272 69.271 20.993 30.061 1.00 0.00 C

ATOM 4465 HA THR 272 69.185 21.462 31.041 1.00 0.00 H

ATOM 4466 CB THR 272 67.902 20.277 30.109 1.00 0.00 C

ATOM 4467 HB THR 272 67.995 19.643 30.991 1.00 0.00 H

ATOM 4468 CG2 THR 272 66.745 21.232 30.161 1.00 0.00 C

ATOM 4469 HG21 THR 272 66.214 21.288 29.210 1.00 0.00 H

ATOM 4470 HG22 THR 272 66.027 20.862 30.892 1.00 0.00 H

ATOM 4471 HG23 THR 272 67.100 22.213 30.476 1.00 0.00 H

ATOM 4472 OG1 THR 272 67.913 19.345 29.031 1.00 0.00 O

ATOM 4473 HG1 THR 272 67.401 19.719 28.310 1.00 0.00 H

ATOM 4474 C THR 272 70.388 19.991 30.265 1.00 0.00 C

ATOM 4475 O THR 272 70.640 19.633 31.403 1.00 0.00 O

ATOM 4476 N ARG 273 71.193 19.521 29.223 1.00 0.00 N

ATOM 4477 H ARG 273 70.894 19.420 28.264 1.00 0.00 H

ATOM 4478 CA ARG 273 72.405 18.776 29.399 1.00 0.00 C

ATOM 4479 HA ARG 273 72.255 17.962 30.108 1.00 0.00 H

ATOM 4480 CB ARG 273 72.804 18.132 28.070 1.00 0.00 C

ATOM 4481 HB2 ARG 273 71.988 17.628 27.552 1.00 0.00 H

ATOM 4482 HB3 ARG 273 72.988 18.955 27.378 1.00 0.00 H

ATOM 4483 CG ARG 273 74.074 17.227 27.956 1.00 0.00 C

ATOM 4484 HG2 ARG 273 74.988 17.762 27.697 1.00 0.00 H

ATOM 4485 HG3 ARG 273 74.174 16.706 28.908 1.00 0.00 H

ATOM 4486 CD ARG 273 73.879 16.145 26.900 1.00 0.00 C

ATOM 4487 HD2 ARG 273 74.658 15.415 27.119 1.00 0.00 H

ATOM 4488 HD3 ARG 273 72.874 15.765 27.086 1.00 0.00 H

ATOM 4489 NE ARG 273 74.028 16.677 25.487 1.00 0.00 N

ATOM 4490 HE ARG 273 74.012 17.686 25.519 1.00 0.00 H

ATOM 4491 CZ ARG 273 74.131 15.840 24.438 1.00 0.00 C

ATOM 4492 NH1 ARG 273 74.183 14.603 24.634 1.00 0.00 N

ATOM 4493 HH11 ARG 273 74.377 14.052 23.810 1.00 0.00 H

ATOM 4494 HH12 ARG 273 74.190 14.133 25.529 1.00 0.00 H

ATOM 4495 NH2 ARG 273 74.329 16.387 23.289 1.00 0.00 N

ATOM 4496 HH21 ARG 273 74.421 15.842 22.444 1.00 0.00 H

ATOM 4497 HH22 ARG 273 74.236 17.393 23.274 1.00 0.00 H

ATOM 4498 C ARG 273 73.484 19.705 29.999 1.00 0.00 C

ATOM 4499 O ARG 273 74.426 19.193 30.664 1.00 0.00 O

ATOM 4500 N GLY 274 73.401 21.040 29.770 1.00 0.00 N

ATOM 4501 H GLY 274 72.798 21.436 29.063 1.00 0.00 H

ATOM 4502 CA GLY 274 73.965 22.044 30.652 1.00 0.00 C

ATOM 4503 HA2 GLY 274 75.054 22.025 30.609 1.00 0.00 H

ATOM 4504 HA3 GLY 274 73.701 23.029 30.266 1.00 0.00 H

ATOM 4505 C GLY 274 73.602 21.883 32.149 1.00 0.00 C

ATOM 4506 O GLY 274 74.442 21.574 32.986 1.00 0.00 O

ATOM 4507 N LEU 275 72.295 21.952 32.446 1.00 0.00 N

ATOM 4508 H LEU 275 71.647 22.079 31.681 1.00 0.00 H

ATOM 4509 CA LEU 275 71.840 21.653 33.769 1.00 0.00 C

ATOM 4510 HA LEU 275 72.177 22.366 34.521 1.00 0.00 H

ATOM 4511 CB LEU 275 70.329 22.010 33.806 1.00 0.00 C

ATOM 4512 HB2 LEU 275 69.785 21.452 33.044 1.00 0.00 H

ATOM 4513 HB3 LEU 275 69.932 21.595 34.732 1.00 0.00 H

ATOM 4514 CG LEU 275 69.989 23.476 33.722 1.00 0.00 C

ATOM 4515 HG LEU 275 70.729 23.963 33.086 1.00 0.00 H

ATOM 4516 CD1 LEU 275 68.659 23.728 33.095 1.00 0.00 C

ATOM 4517 HD11 LEU 275 68.422 24.758 32.832 1.00 0.00 H

ATOM 4518 HD12 LEU 275 68.461 23.180 32.173 1.00 0.00 H

ATOM 4519 HD13 LEU 275 67.789 23.490 33.706 1.00 0.00 H

ATOM 4520 CD2 LEU 275 70.081 24.137 35.136 1.00 0.00 C

ATOM 4521 HD21 LEU 275 70.362 25.185 35.031 1.00 0.00 H

ATOM 4522 HD22 LEU 275 69.070 24.176 35.539 1.00 0.00 H

ATOM 4523 HD23 LEU 275 70.814 23.569 35.710 1.00 0.00 H

ATOM 4524 C LEU 275 72.157 20.344 34.426 1.00 0.00 C

ATOM 4525 O LEU 275 72.157 20.240 35.651 1.00 0.00 O

ATOM 4526 N ALA 276 72.224 19.270 33.723 1.00 0.00 N

ATOM 4527 H ALA 276 71.964 19.373 32.753 1.00 0.00 H

ATOM 4528 CA ALA 276 72.960 18.025 34.091 1.00 0.00 C

ATOM 4529 HA ALA 276 72.438 17.685 34.985 1.00 0.00 H

ATOM 4530 CB ALA 276 72.617 16.889 33.074 1.00 0.00 C

ATOM 4531 HB1 ALA 276 71.536 16.858 32.938 1.00 0.00 H

ATOM 4532 HB2 ALA 276 73.136 16.940 32.116 1.00 0.00 H

ATOM 4533 HB3 ALA 276 72.794 15.991 33.666 1.00 0.00 H

ATOM 4534 C ALA 276 74.456 18.173 34.446 1.00 0.00 C

ATOM 4535 O ALA 276 75.325 17.555 33.829 1.00 0.00 O

ATOM 4536 N SER 277 74.728 19.248 35.248 1.00 0.00 N

ATOM 4537 H SER 277 73.959 19.850 35.507 1.00 0.00 H

ATOM 4538 CA SER 277 75.990 19.592 35.825 1.00 0.00 C

ATOM 4539 HA SER 277 76.700 18.773 35.711 1.00 0.00 H

ATOM 4540 CB SER 277 76.671 20.745 35.024 1.00 0.00 C

ATOM 4541 HB2 SER 277 76.197 21.719 35.148 1.00 0.00 H

ATOM 4542 HB3 SER 277 77.636 21.022 35.447 1.00 0.00 H

ATOM 4543 OG SER 277 76.818 20.393 33.612 1.00 0.00 O

ATOM 4544 HG SER 277 76.030 20.695 33.154 1.00 0.00 H

ATOM 4545 C SER 277 75.993 20.031 37.285 1.00 0.00 C

ATOM 4546 O SER 277 77.057 20.416 37.796 1.00 0.00 O

ATOM 4547 N LEU 278 74.785 20.162 37.816 1.00 0.00 N

ATOM 4548 H LEU 278 73.930 19.845 37.383 1.00 0.00 H

ATOM 4549 CA LEU 278 74.681 20.973 39.043 1.00 0.00 C

ATOM 4550 HA LEU 278 75.201 21.919 38.892 1.00 0.00 H

ATOM 4551 CB LEU 278 73.168 21.337 39.306 1.00 0.00 C

ATOM 4552 HB2 LEU 278 72.891 22.041 38.522 1.00 0.00 H

ATOM 4553 HB3 LEU 278 72.586 20.432 39.132 1.00 0.00 H

ATOM 4554 CG LEU 278 72.791 22.069 40.608 1.00 0.00 C

ATOM 4555 HG LEU 278 72.989 21.367 41.418 1.00 0.00 H

ATOM 4556 CD1 LEU 278 73.637 23.306 40.731 1.00 0.00 C

ATOM 4557 HD11 LEU 278 74.676 23.198 41.044 1.00 0.00 H

ATOM 4558 HD12 LEU 278 73.783 23.852 39.799 1.00 0.00 H

ATOM 4559 HD13 LEU 278 73.236 23.923 41.536 1.00 0.00 H

ATOM 4560 CD2 LEU 278 71.297 22.464 40.565 1.00 0.00 C

ATOM 4561 HD21 LEU 278 70.832 22.651 41.533 1.00 0.00 H

ATOM 4562 HD22 LEU 278 71.264 23.398 40.004 1.00 0.00 H

ATOM 4563 HD23 LEU 278 70.772 21.681 40.017 1.00 0.00 H

ATOM 4564 C LEU 278 75.167 20.332 40.286 1.00 0.00 C

ATOM 4565 O LEU 278 76.011 20.874 40.990 1.00 0.00 O

ATOM 4566 N ASN 279 74.775 19.001 40.462 1.00 0.00 N

ATOM 4567 H ASN 279 74.311 18.668 39.629 1.00 0.00 H

ATOM 4568 CA ASN 279 75.110 18.163 41.606 1.00 0.00 C

ATOM 4569 HA ASN 279 74.709 18.696 42.468 1.00 0.00 H

ATOM 4570 CB ASN 279 74.643 16.790 41.514 1.00 0.00 C

ATOM 4571 HB2 ASN 279 73.629 16.747 41.117 1.00 0.00 H

ATOM 4572 HB3 ASN 279 75.132 16.223 40.722 1.00 0.00 H

ATOM 4573 CG ASN 279 74.777 16.090 42.862 1.00 0.00 C

ATOM 4574 OD1 ASN 279 75.735 15.981 43.598 1.00 0.00 O

ATOM 4575 ND2 ASN 279 73.816 15.247 43.022 1.00 0.00 N

ATOM 4576 HD21 ASN 279 73.794 14.714 43.879 1.00 0.00 H

ATOM 4577 HD22 ASN 279 73.132 15.184 42.281 1.00 0.00 H

ATOM 4578 C ASN 279 76.650 18.158 41.761 1.00 0.00 C

ATOM 4579 O ASN 279 77.069 18.478 42.850 1.00 0.00 O

ATOM 4580 N SER 280 77.512 17.941 40.816 1.00 0.00 N

ATOM 4581 H SER 280 77.113 17.809 39.898 1.00 0.00 H

ATOM 4582 CA SER 280 79.053 18.071 40.894 1.00 0.00 C

ATOM 4583 HA SER 280 79.333 17.594 41.833 1.00 0.00 H

ATOM 4584 CB SER 280 79.713 17.399 39.692 1.00 0.00 C

ATOM 4585 HB2 SER 280 79.407 17.998 38.833 1.00 0.00 H

ATOM 4586 HB3 SER 280 80.789 17.542 39.781 1.00 0.00 H

ATOM 4587 OG SER 280 79.409 16.057 39.465 1.00 0.00 O

ATOM 4588 HG SER 280 79.868 15.543 40.133 1.00 0.00 H

ATOM 4589 C SER 280 79.639 19.540 41.041 1.00 0.00 C

ATOM 4590 O SER 280 80.799 19.653 41.341 1.00 0.00 O

ATOM 4591 N CYX 281 78.828 20.592 41.147 1.00 0.00 N

ATOM 4592 H CYX 281 78.024 20.604 40.537 1.00 0.00 H

ATOM 4593 CA CYX 281 79.247 21.874 41.732 1.00 0.00 C

ATOM 4594 HA CYX 281 80.313 22.090 41.658 1.00 0.00 H

ATOM 4595 CB CYX 281 78.556 23.011 40.946 1.00 0.00 C

ATOM 4596 HB2 CYX 281 77.474 22.883 40.974 1.00 0.00 H

ATOM 4597 HB3 CYX 281 78.769 23.945 41.466 1.00 0.00 H

ATOM 4598 SG CYX 281 79.137 23.353 39.308 1.00 0.00 S

ATOM 4599 C CYX 281 78.885 22.016 43.266 1.00 0.00 C

ATOM 4600 O CYX 281 79.615 22.762 43.952 1.00 0.00 O

ATOM 4601 N VAL 282 77.832 21.324 43.717 1.00 0.00 N

ATOM 4602 H VAL 282 77.460 20.622 43.093 1.00 0.00 H

ATOM 4603 CA VAL 282 77.390 21.466 45.158 1.00 0.00 C

ATOM 4604 HA VAL 282 77.692 22.465 45.470 1.00 0.00 H

ATOM 4605 CB VAL 282 75.894 21.276 45.155 1.00 0.00 C

ATOM 4606 HB VAL 282 75.738 20.238 44.860 1.00 0.00 H

ATOM 4607 CG1 VAL 282 75.214 21.288 46.559 1.00 0.00 C

ATOM 4608 HG11 VAL 282 75.607 20.507 47.209 1.00 0.00 H

ATOM 4609 HG12 VAL 282 75.093 22.261 47.037 1.00 0.00 H

ATOM 4610 HG13 VAL 282 74.179 20.956 46.476 1.00 0.00 H

ATOM 4611 CG2 VAL 282 75.091 22.349 44.280 1.00 0.00 C

ATOM 4612 HG21 VAL 282 75.864 22.879 43.722 1.00 0.00 H

ATOM 4613 HG22 VAL 282 74.453 21.744 43.636 1.00 0.00 H

ATOM 4614 HG23 VAL 282 74.501 23.059 44.858 1.00 0.00 H

ATOM 4615 C VAL 282 77.985 20.429 46.131 1.00 0.00 C

ATOM 4616 O VAL 282 78.480 20.688 47.269 1.00 0.00 O

ATOM 4617 N ASN 283 78.141 19.207 45.606 1.00 0.00 N

ATOM 4618 H ASN 283 77.775 19.029 44.682 1.00 0.00 H

ATOM 4619 CA ASN 283 78.761 18.111 46.337 1.00 0.00 C

ATOM 4620 HA ASN 283 78.231 18.019 47.285 1.00 0.00 H

ATOM 4621 CB ASN 283 78.712 16.761 45.456 1.00 0.00 C

ATOM 4622 HB2 ASN 283 77.666 16.561 45.226 1.00 0.00 H

ATOM 4623 HB3 ASN 283 79.234 16.799 44.500 1.00 0.00 H

ATOM 4624 CG ASN 283 79.102 15.501 46.249 1.00 0.00 C

ATOM 4625 OD1 ASN 283 78.653 15.251 47.343 1.00 0.00 O

ATOM 4626 ND2 ASN 283 80.089 14.777 45.800 1.00 0.00 N

ATOM 4627 HD21 ASN 283 80.388 13.990 46.357 1.00 0.00 H

ATOM 4628 HD22 ASN 283 80.795 15.071 45.140 1.00 0.00 H

ATOM 4629 C ASN 283 80.211 18.302 46.792 1.00 0.00 C

ATOM 4630 O ASN 283 80.363 18.052 47.980 1.00 0.00 O

ATOM 4631 N PRO 284 81.136 19.010 46.070 1.00 0.00 N

ATOM 4632 CD PRO 284 81.037 19.230 44.625 1.00 0.00 C

ATOM 4633 HD2 PRO 284 80.252 19.976 44.504 1.00 0.00 H

ATOM 4634 HD3 PRO 284 80.802 18.372 43.995 1.00 0.00 H

ATOM 4635 CG PRO 284 82.280 19.938 44.245 1.00 0.00 C

ATOM 4636 HG2 PRO 284 81.973 20.898 43.829 1.00 0.00 H

ATOM 4637 HG3 PRO 284 82.765 19.392 43.436 1.00 0.00 H

ATOM 4638 CB PRO 284 83.204 20.119 45.408 1.00 0.00 C

ATOM 4639 HB2 PRO 284 83.280 21.195 45.566 1.00 0.00 H

ATOM 4640 HB3 PRO 284 84.122 19.590 45.153 1.00 0.00 H

ATOM 4641 CA PRO 284 82.435 19.458 46.590 1.00 0.00 C

ATOM 4642 HA PRO 284 82.928 18.544 46.922 1.00 0.00 H

ATOM 4643 C PRO 284 82.287 20.180 47.929 1.00 0.00 C

ATOM 4644 O PRO 284 82.887 19.786 48.929 1.00 0.00 O

ATOM 4645 N ILE 285 81.420 21.175 48.009 1.00 0.00 N

ATOM 4646 H ILE 285 80.772 21.123 47.236 1.00 0.00 H

ATOM 4647 CA ILE 285 81.236 22.101 49.132 1.00 0.00 C

ATOM 4648 HA ILE 285 82.228 22.194 49.574 1.00 0.00 H

ATOM 4649 CB ILE 285 80.884 23.508 48.621 1.00 0.00 C

ATOM 4650 HB ILE 285 81.650 23.724 47.877 1.00 0.00 H

ATOM 4651 CG2 ILE 285 79.576 23.575 47.808 1.00 0.00 C

ATOM 4652 HG21 ILE 285 79.693 22.938 46.931 1.00 0.00 H

ATOM 4653 HG22 ILE 285 78.767 23.375 48.509 1.00 0.00 H

ATOM 4654 HG23 ILE 285 79.255 24.519 47.368 1.00 0.00 H

ATOM 4655 CG1 ILE 285 80.960 24.685 49.720 1.00 0.00 C

ATOM 4656 HG12 ILE 285 81.009 25.609 49.144 1.00 0.00 H

ATOM 4657 HG13 ILE 285 80.057 24.645 50.330 1.00 0.00 H

ATOM 4658 CD1 ILE 285 82.177 24.670 50.599 1.00 0.00 C

ATOM 4659 HD11 ILE 285 81.891 23.961 51.376 1.00 0.00 H

ATOM 4660 HD12 ILE 285 83.021 24.402 49.963 1.00 0.00 H

ATOM 4661 HD13 ILE 285 82.417 25.643 51.027 1.00 0.00 H

ATOM 4662 C ILE 285 80.392 21.429 50.325 1.00 0.00 C

ATOM 4663 O ILE 285 80.617 21.739 51.491 1.00 0.00 O

ATOM 4664 N LEU 286 79.469 20.459 50.055 1.00 0.00 N

ATOM 4665 H LEU 286 79.159 20.369 49.098 1.00 0.00 H

ATOM 4666 CA LEU 286 78.700 19.809 51.068 1.00 0.00 C

ATOM 4667 HA LEU 286 78.552 20.604 51.799 1.00 0.00 H

ATOM 4668 CB LEU 286 77.216 19.511 50.499 1.00 0.00 C

ATOM 4669 HB2 LEU 286 76.872 20.328 49.865 1.00 0.00 H

ATOM 4670 HB3 LEU 286 77.229 18.558 49.971 1.00 0.00 H

ATOM 4671 CG LEU 286 76.003 19.479 51.475 1.00 0.00 C

ATOM 4672 HG LEU 286 76.115 18.657 52.182 1.00 0.00 H

ATOM 4673 CD1 LEU 286 75.807 20.778 52.230 1.00 0.00 C

ATOM 4674 HD11 LEU 286 74.997 20.636 52.946 1.00 0.00 H

ATOM 4675 HD12 LEU 286 76.699 20.971 52.827 1.00 0.00 H

ATOM 4676 HD13 LEU 286 75.686 21.559 51.480 1.00 0.00 H

ATOM 4677 CD2 LEU 286 74.657 19.241 50.701 1.00 0.00 C

ATOM 4678 HD21 LEU 286 73.792 19.548 51.289 1.00 0.00 H

ATOM 4679 HD22 LEU 286 74.659 19.858 49.802 1.00 0.00 H

ATOM 4680 HD23 LEU 286 74.596 18.176 50.478 1.00 0.00 H

ATOM 4681 C LEU 286 79.272 18.774 51.978 1.00 0.00 C

ATOM 4682 O LEU 286 78.830 18.628 53.138 1.00 0.00 O

ATOM 4683 N TYR 287 80.315 17.991 51.536 1.00 0.00 N

ATOM 4684 H TYR 287 80.812 18.306 50.715 1.00 0.00 H

ATOM 4685 CA TYR 287 80.976 16.876 52.323 1.00 0.00 C

ATOM 4686 HA TYR 287 80.378 15.970 52.225 1.00 0.00 H

ATOM 4687 CB TYR 287 82.367 16.540 51.682 1.00 0.00 C

ATOM 4688 HB2 TYR 287 82.900 17.489 51.621 1.00 0.00 H

ATOM 4689 HB3 TYR 287 82.964 15.869 52.299 1.00 0.00 H

ATOM 4690 CG TYR 287 82.270 15.862 50.376 1.00 0.00 C

ATOM 4691 CD1 TYR 287 81.889 14.503 50.261 1.00 0.00 C

ATOM 4692 HD1 TYR 287 81.723 14.063 51.233 1.00 0.00 H

ATOM 4693 CE1 TYR 287 81.975 13.790 49.054 1.00 0.00 C

ATOM 4694 HE1 TYR 287 81.850 12.719 49.006 1.00 0.00 H

ATOM 4695 CZ TYR 287 82.435 14.453 47.912 1.00 0.00 C

ATOM 4696 OH TYR 287 82.619 13.722 46.777 1.00 0.00 O

ATOM 4697 HH TYR 287 83.149 14.223 46.153 1.00 0.00 H

ATOM 4698 CE2 TYR 287 82.970 15.757 47.975 1.00 0.00 C

ATOM 4699 HE2 TYR 287 83.208 16.323 47.087 1.00 0.00 H

ATOM 4700 CD2 TYR 287 82.895 16.449 49.235 1.00 0.00 C

ATOM 4701 HD2 TYR 287 83.432 17.384 49.175 1.00 0.00 H

ATOM 4702 C TYR 287 81.095 17.074 53.834 1.00 0.00 C

ATOM 4703 O TYR 287 80.396 16.378 54.639 1.00 0.00 O

ATOM 4704 N PHE 288 81.780 18.107 54.253 1.00 0.00 N

ATOM 4705 H PHE 288 82.288 18.744 53.657 1.00 0.00 H

ATOM 4706 CA PHE 288 82.118 18.256 55.654 1.00 0.00 C

ATOM 4707 HA PHE 288 82.057 17.271 56.116 1.00 0.00 H

ATOM 4708 CB PHE 288 83.615 18.525 55.827 1.00 0.00 C

ATOM 4709 HB2 PHE 288 83.892 19.572 55.709 1.00 0.00 H

ATOM 4710 HB3 PHE 288 83.822 18.237 56.858 1.00 0.00 H

ATOM 4711 CG PHE 288 84.496 17.679 54.935 1.00 0.00 C

ATOM 4712 CD1 PHE 288 85.008 18.305 53.796 1.00 0.00 C

ATOM 4713 HD1 PHE 288 84.755 19.333 53.584 1.00 0.00 H

ATOM 4714 CE1 PHE 288 85.717 17.567 52.831 1.00 0.00 C

ATOM 4715 HE1 PHE 288 86.139 18.078 51.978 1.00 0.00 H

ATOM 4716 CZ PHE 288 85.928 16.201 53.049 1.00 0.00 C

ATOM 4717 HZ PHE 288 86.506 15.668 52.309 1.00 0.00 H

ATOM 4718 CE2 PHE 288 85.605 15.623 54.289 1.00 0.00 C

ATOM 4719 HE2 PHE 288 85.926 14.636 54.588 1.00 0.00 H

ATOM 4720 CD2 PHE 288 84.817 16.355 55.271 1.00 0.00 C

ATOM 4721 HD2 PHE 288 84.328 15.825 56.075 1.00 0.00 H

ATOM 4722 C PHE 288 81.227 19.210 56.511 1.00 0.00 C

ATOM 4723 O PHE 288 81.649 19.919 57.425 1.00 0.00 O

ATOM 4724 N LEU 289 79.936 19.193 56.190 1.00 0.00 N

ATOM 4725 H LEU 289 79.627 18.533 55.490 1.00 0.00 H

ATOM 4726 CA LEU 289 78.886 20.213 56.492 1.00 0.00 C

ATOM 4727 HA LEU 289 79.146 20.826 57.355 1.00 0.00 H

ATOM 4728 CB LEU 289 78.942 21.259 55.386 1.00 0.00 C

ATOM 4729 HB2 LEU 289 79.854 21.846 55.499 1.00 0.00 H

ATOM 4730 HB3 LEU 289 79.054 20.776 54.415 1.00 0.00 H

ATOM 4731 CG LEU 289 77.799 22.373 55.422 1.00 0.00 C

ATOM 4732 HG LEU 289 76.885 21.885 55.083 1.00 0.00 H

ATOM 4733 CD1 LEU 289 77.749 23.018 56.810 1.00 0.00 C

ATOM 4734 HD11 LEU 289 77.084 22.490 57.493 1.00 0.00 H

ATOM 4735 HD12 LEU 289 78.706 23.071 57.329 1.00 0.00 H

ATOM 4736 HD13 LEU 289 77.342 24.028 56.792 1.00 0.00 H

ATOM 4737 CD2 LEU 289 78.198 23.480 54.399 1.00 0.00 C

ATOM 4738 HD21 LEU 289 77.572 24.359 54.550 1.00 0.00 H

ATOM 4739 HD22 LEU 289 79.238 23.799 54.460 1.00 0.00 H

ATOM 4740 HD23 LEU 289 78.085 23.074 53.393 1.00 0.00 H

ATOM 4741 C LEU 289 77.483 19.591 56.666 1.00 0.00 C

ATOM 4742 O LEU 289 76.672 19.922 57.457 1.00 0.00 O

ATOM 4743 N ALA 290 77.161 18.670 55.661 1.00 0.00 N

ATOM 4744 H ALA 290 77.498 18.908 54.739 1.00 0.00 H

ATOM 4745 CA ALA 290 76.066 17.727 55.777 1.00 0.00 C

ATOM 4746 HA ALA 290 76.096 17.299 56.779 1.00 0.00 H

ATOM 4747 CB ALA 290 74.659 18.411 55.530 1.00 0.00 C

ATOM 4748 HB1 ALA 290 74.483 19.187 56.276 1.00 0.00 H

ATOM 4749 HB2 ALA 290 74.824 18.940 54.591 1.00 0.00 H

ATOM 4750 HB3 ALA 290 73.864 17.668 55.482 1.00 0.00 H

ATOM 4751 C ALA 290 76.262 16.348 54.961 1.00 0.00 C

ATOM 4752 O ALA 290 75.420 15.479 54.941 1.00 0.00 O

ATOM 4753 N GLY 291 77.505 16.130 54.492 1.00 0.00 N

ATOM 4754 H GLY 291 78.123 16.921 54.378 1.00 0.00 H

ATOM 4755 CA GLY 291 78.084 14.844 54.211 1.00 0.00 C

ATOM 4756 HA2 GLY 291 77.353 14.340 53.579 1.00 0.00 H

ATOM 4757 HA3 GLY 291 78.960 14.934 53.569 1.00 0.00 H

ATOM 4758 C GLY 291 78.439 14.052 55.471 1.00 0.00 C

ATOM 4759 O GLY 291 77.495 13.541 56.091 1.00 0.00 O

ATOM 4760 N ASP 292 79.651 13.990 55.886 1.00 0.00 N

ATOM 4761 H ASP 292 80.177 14.841 55.747 1.00 0.00 H

ATOM 4762 CA ASP 292 80.151 13.022 56.929 1.00 0.00 C

ATOM 4763 HA ASP 292 79.236 12.586 57.331 1.00 0.00 H

ATOM 4764 CB ASP 292 80.996 11.870 56.411 1.00 0.00 C

ATOM 4765 HB2 ASP 292 80.580 11.203 55.656 1.00 0.00 H

ATOM 4766 HB3 ASP 292 81.930 12.228 55.978 1.00 0.00 H

ATOM 4767 CG ASP 292 81.299 10.902 57.532 1.00 0.00 C

ATOM 4768 OD1 ASP 292 80.365 10.501 58.291 1.00 0.00 O

ATOM 4769 OD2 ASP 292 82.490 10.553 57.677 1.00 0.00 O

ATOM 4770 C ASP 292 80.770 13.834 58.104 1.00 0.00 C

ATOM 4771 O ASP 292 81.120 15.002 57.920 1.00 0.00 O

ATOM 4772 N THR 293 80.995 13.333 59.312 1.00 0.00 N

ATOM 4773 H THR 293 81.087 12.346 59.504 1.00 0.00 H

ATOM 4774 CA THR 293 81.297 14.218 60.483 1.00 0.00 C

ATOM 4775 HA THR 293 80.908 15.227 60.345 1.00 0.00 H

ATOM 4776 CB THR 293 80.624 13.808 61.795 1.00 0.00 C

ATOM 4777 HB THR 293 81.287 14.075 62.618 1.00 0.00 H

ATOM 4778 CG2 THR 293 79.357 14.585 62.055 1.00 0.00 C

ATOM 4779 HG21 THR 293 78.749 14.595 61.150 1.00 0.00 H

ATOM 4780 HG22 THR 293 78.718 14.061 62.765 1.00 0.00 H

ATOM 4781 HG23 THR 293 79.448 15.651 62.266 1.00 0.00 H

ATOM 4782 OG1 THR 293 80.356 12.430 61.886 1.00 0.00 O

ATOM 4783 HG1 THR 293 80.333 12.262 62.831 1.00 0.00 H

ATOM 4784 C THR 293 82.806 14.280 60.647 1.00 0.00 C

ATOM 4785 O THR 293 83.542 13.278 60.761 1.00 0.00 O

ATOM 4786 N PHE 294 83.296 15.480 60.554 1.00 0.00 N

ATOM 4787 H PHE 294 82.666 16.264 60.657 1.00 0.00 H

ATOM 4788 CA PHE 294 84.669 15.880 60.529 1.00 0.00 C

ATOM 4789 HA PHE 294 85.296 15.088 60.119 1.00 0.00 H

ATOM 4790 CB PHE 294 84.856 17.118 59.625 1.00 0.00 C

ATOM 4791 HB2 PHE 294 85.635 16.798 58.933 1.00 0.00 H

ATOM 4792 HB3 PHE 294 83.950 17.243 59.031 1.00 0.00 H

ATOM 4793 CG PHE 294 85.250 18.463 60.372 1.00 0.00 C

ATOM 4794 CD1 PHE 294 84.477 19.569 60.437 1.00 0.00 C

ATOM 4795 HD1 PHE 294 83.502 19.554 59.972 1.00 0.00 H

ATOM 4796 CE1 PHE 294 84.878 20.788 60.968 1.00 0.00 C

ATOM 4797 HE1 PHE 294 84.228 21.647 61.040 1.00 0.00 H

ATOM 4798 CZ PHE 294 86.193 20.937 61.400 1.00 0.00 C

ATOM 4799 HZ PHE 294 86.461 21.789 62.009 1.00 0.00 H

ATOM 4800 CE2 PHE 294 87.118 19.890 61.149 1.00 0.00 C

ATOM 4801 HE2 PHE 294 88.167 19.969 61.395 1.00 0.00 H

ATOM 4802 CD2 PHE 294 86.639 18.637 60.695 1.00 0.00 C

ATOM 4803 HD2 PHE 294 87.334 17.822 60.556 1.00 0.00 H

ATOM 4804 C PHE 294 85.332 16.012 61.909 1.00 0.00 C

ATOM 4805 O PHE 294 86.513 15.714 62.026 1.00 0.00 O

ATOM 4806 N ARG 295 84.506 16.195 62.951 1.00 0.00 N

ATOM 4807 H ARG 295 83.567 16.545 62.824 1.00 0.00 H

ATOM 4808 CA ARG 295 84.838 15.634 64.252 1.00 0.00 C

ATOM 4809 HA ARG 295 85.830 15.205 64.109 1.00 0.00 H

ATOM 4810 CB ARG 295 84.753 16.753 65.315 1.00 0.00 C

ATOM 4811 HB2 ARG 295 83.685 16.859 65.504 1.00 0.00 H

ATOM 4812 HB3 ARG 295 85.074 16.264 66.234 1.00 0.00 H

ATOM 4813 CG ARG 295 85.528 18.066 65.176 1.00 0.00 C

ATOM 4814 HG2 ARG 295 84.949 18.701 64.506 1.00 0.00 H

ATOM 4815 HG3 ARG 295 85.487 18.603 66.124 1.00 0.00 H

ATOM 4816 CD ARG 295 86.910 17.977 64.567 1.00 0.00 C

ATOM 4817 HD2 ARG 295 87.509 17.455 65.313 1.00 0.00 H

ATOM 4818 HD3 ARG 295 87.010 17.363 63.672 1.00 0.00 H

ATOM 4819 NE ARG 295 87.598 19.287 64.441 1.00 0.00 N

ATOM 4820 HE ARG 295 87.080 20.105 64.152 1.00 0.00 H

ATOM 4821 CZ ARG 295 88.883 19.517 64.653 1.00 0.00 C

ATOM 4822 NH1 ARG 295 89.786 18.614 64.770 1.00 0.00 N

ATOM 4823 HH11 ARG 295 89.700 17.679 64.397 1.00 0.00 H

ATOM 4824 HH12 ARG 295 90.704 18.951 65.024 1.00 0.00 H

ATOM 4825 NH2 ARG 295 89.225 20.765 64.476 1.00 0.00 N

ATOM 4826 HH21 ARG 295 89.989 21.058 65.068 1.00 0.00 H

ATOM 4827 HH22 ARG 295 88.432 21.367 64.303 1.00 0.00 H

ATOM 4828 C ARG 295 83.890 14.450 64.574 1.00 0.00 C

ATOM 4829 O ARG 295 82.690 14.601 64.316 1.00 0.00 O

ATOM 4830 N ARG 296 84.422 13.343 65.103 1.00 0.00 N

ATOM 4831 H ARG 296 85.406 13.343 65.332 1.00 0.00 H

ATOM 4832 CA ARG 296 83.593 12.219 65.608 1.00 0.00 C

ATOM 4833 HA ARG 296 82.522 12.424 65.602 1.00 0.00 H

ATOM 4834 CB ARG 296 84.043 10.988 64.743 1.00 0.00 C

ATOM 4835 HB2 ARG 296 85.122 10.999 64.584 1.00 0.00 H

ATOM 4836 HB3 ARG 296 83.915 10.061 65.303 1.00 0.00 H

ATOM 4837 CG ARG 296 83.380 10.777 63.353 1.00 0.00 C

ATOM 4838 HG2 ARG 296 82.313 10.594 63.479 1.00 0.00 H

ATOM 4839 HG3 ARG 296 83.594 11.572 62.638 1.00 0.00 H

ATOM 4840 CD ARG 296 83.975 9.509 62.694 1.00 0.00 C

ATOM 4841 HD2 ARG 296 84.863 9.806 62.137 1.00 0.00 H

ATOM 4842 HD3 ARG 296 84.377 8.810 63.428 1.00 0.00 H

ATOM 4843 NE ARG 296 83.031 8.753 61.868 1.00 0.00 N

ATOM 4844 HE ARG 296 82.561 7.962 62.285 1.00 0.00 H

ATOM 4845 CZ ARG 296 82.719 9.217 60.702 1.00 0.00 C

ATOM 4846 NH1 ARG 296 83.645 9.860 60.061 1.00 0.00 N

ATOM 4847 HH11 ARG 296 83.430 10.067 59.096 1.00 0.00 H

ATOM 4848 HH12 ARG 296 84.573 10.044 60.414 1.00 0.00 H

ATOM 4849 NH2 ARG 296 81.521 8.924 60.204 1.00 0.00 N

ATOM 4850 HH21 ARG 296 81.199 9.483 59.427 1.00 0.00 H

ATOM 4851 HH22 ARG 296 80.941 8.273 60.715 1.00 0.00 H

ATOM 4852 C ARG 296 83.736 11.954 67.097 1.00 0.00 C

ATOM 4853 O ARG 296 82.813 11.245 67.636 1.00 0.00 O

ATOM 4854 N ARG 297 84.686 12.607 67.718 1.00 0.00 N

ATOM 4855 H ARG 297 85.138 13.392 67.273 1.00 0.00 H

ATOM 4856 CA ARG 297 84.825 12.660 69.199 1.00 0.00 C

ATOM 4857 HA ARG 297 84.832 11.608 69.485 1.00 0.00 H

ATOM 4858 CB ARG 297 86.183 13.273 69.512 1.00 0.00 C

ATOM 4859 HB2 ARG 297 86.389 14.154 68.904 1.00 0.00 H

ATOM 4860 HB3 ARG 297 86.179 13.533 70.570 1.00 0.00 H

ATOM 4861 CG ARG 297 87.402 12.404 69.312 1.00 0.00 C

ATOM 4862 HG2 ARG 297 87.403 11.996 68.301 1.00 0.00 H

ATOM 4863 HG3 ARG 297 88.339 12.944 69.448 1.00 0.00 H

ATOM 4864 CD ARG 297 87.586 11.210 70.286 1.00 0.00 C

ATOM 4865 HD2 ARG 297 87.780 11.499 71.319 1.00 0.00 H

ATOM 4866 HD3 ARG 297 86.623 10.704 70.358 1.00 0.00 H

ATOM 4867 NE ARG 297 88.534 10.195 69.724 1.00 0.00 N

ATOM 4868 HE ARG 297 88.097 9.355 69.373 1.00 0.00 H

ATOM 4869 CZ ARG 297 89.841 10.351 69.478 1.00 0.00 C

ATOM 4870 NH1 ARG 297 90.460 9.373 68.918 1.00 0.00 N

ATOM 4871 HH11 ARG 297 90.032 8.499 68.649 1.00 0.00 H

ATOM 4872 HH12 ARG 297 91.402 9.721 68.812 1.00 0.00 H

ATOM 4873 NH2 ARG 297 90.601 11.353 69.837 1.00 0.00 N

ATOM 4874 HH21 ARG 297 90.316 11.702 70.740 1.00 0.00 H

ATOM 4875 HH22 ARG 297 91.521 11.362 69.421 1.00 0.00 H

ATOM 4876 C ARG 297 83.622 13.343 69.768 1.00 0.00 C

ATOM 4877 O ARG 297 83.134 14.276 69.167 1.00 0.00 O

ATOM 4878 OXT ARG 297 83.173 12.795 70.754 1.00 0.00 O

TER

ATOM 4879 O2B 6AD 298 74.646 12.674 22.549 1.00 0.00 O

ATOM 4880 PB 6AD 298 73.291 12.214 22.269 1.00 0.00 P

ATOM 4881 O1B 6AD 298 72.457 12.390 23.484 1.00 0.00 O

ATOM 4882 O3B 6AD 298 72.871 13.072 21.181 1.00 0.00 O

ATOM 4883 O3A 6AD 298 73.471 10.697 21.912 1.00 0.00 O

ATOM 4884 PA 6AD 298 72.506 10.020 20.875 1.00 0.00 P

ATOM 4885 O1A 6AD 298 72.759 10.626 19.517 1.00 0.00 O

ATOM 4886 O2A 6AD 298 71.127 9.778 21.272 1.00 0.00 O

ATOM 4887 O5' 6AD 298 73.337 8.672 20.761 1.00 0.00 O

ATOM 4888 C5' 6AD 298 72.837 7.405 21.161 1.00 0.00 C

ATOM 4889 C4' 6AD 298 73.912 6.824 22.064 1.00 0.00 C

ATOM 4890 O4' 6AD 298 73.812 7.308 23.482 1.00 0.00 O

ATOM 4891 C3' 6AD 298 73.662 5.306 22.134 1.00 0.00 C

ATOM 4892 O3' 6AD 298 74.932 4.604 22.201 1.00 0.00 O

ATOM 4893 C2' 6AD 298 72.845 5.099 23.423 1.00 0.00 C

ATOM 4894 O2' 6AD 298 73.144 3.834 23.923 1.00 0.00 O

ATOM 4895 C1' 6AD 298 73.374 6.215 24.309 1.00 0.00 C

ATOM 4896 N9 6AD 298 72.406 6.608 25.248 1.00 0.00 N

ATOM 4897 C8 6AD 298 71.465 7.608 25.148 1.00 0.00 C

ATOM 4898 N7 6AD 298 70.601 7.711 26.166 1.00 0.00 N

ATOM 4899 C5 6AD 298 71.354 6.958 27.035 1.00 0.00 C

ATOM 4900 C4 6AD 298 72.459 6.335 26.514 1.00 0.00 C

ATOM 4901 N3 6AD 298 73.058 5.298 27.132 1.00 0.00 N

ATOM 4902 C2 6AD 298 72.744 5.249 28.419 1.00 0.00 C

ATOM 4903 S1 6AD 298 73.500 4.084 29.484 1.00 0.00 S

ATOM 4904 C6 6AD 298 73.507 2.578 28.421 1.00 0.00 C

ATOM 4905 N1 6AD 298 71.705 5.890 29.086 1.00 0.00 N

ATOM 4906 C7 6AD 298 71.108 6.775 28.383 1.00 0.00 C

ATOM 4907 N6 6AD 298 70.048 7.291 28.968 1.00 0.00 N

ATOM 4908 H30 6AD 298 71.950 7.443 21.798 1.00 0.00 H

ATOM 4909 H31 6AD 298 72.666 6.836 20.244 1.00 0.00 H

ATOM 4910 H32 6AD 298 74.870 6.934 21.548 1.00 0.00 H

ATOM 4911 H33 6AD 298 73.123 4.762 21.354 1.00 0.00 H

ATOM 4912 H34 6AD 298 74.749 3.814 22.740 1.00 0.00 H

ATOM 4913 H35 6AD 298 71.793 5.282 23.192 1.00 0.00 H

ATOM 4914 H36 6AD 298 72.618 3.680 24.728 1.00 0.00 H

ATOM 4915 H37 6AD 298 74.228 5.858 24.904 1.00 0.00 H

ATOM 4916 H38 6AD 298 71.530 8.200 24.249 1.00 0.00 H

ATOM 4917 H39 6AD 298 72.776 2.721 27.621 1.00 0.00 H

ATOM 4918 H40 6AD 298 74.539 2.547 28.064 1.00 0.00 H

ATOM 4919 H41 6AD 298 73.320 1.641 28.952 1.00 0.00 H

ATOM 4920 H42 6AD 298 69.868 6.759 29.812 1.00 0.00 H

ATOM 4921 H43 6AD 298 69.342 7.475 28.263 1.00 0.00 H

END
