# Supplementary material for: Identification of a Different Agonist-Binding Site and Activation Mechanism of the Human P2Y1 Receptor
Source: Sci Rep. 2017 Oct 23;7:13764. doi: 10.1038/s41598-017-14268-1 (PMC5653743; doi:10.1038/s41598-017-14268-1)
Supplement: Supplementary file 4 — pdb file of the active state [file 41598_2017_14268_MOESM4_ESM.doc]

# Supplementary Information

Identification of a Different Agonist-Binding Site and Activation Mechanism of the Human P2Y_1_ Receptor

Yang Li, Can Yin, Pi Liu, Dongmei Li* and Jianping Lin*

- Coordinates of the active state of the 2MeSADP-P2Y_12_R system (pdb file).

ACTIVE STATE

ATOM 1 N SER 1 32.527 32.919 1.199 1.00 0.00 N

ATOM 2 H1 SER 1 32.240 32.745 0.247 1.00 0.00 H

ATOM 3 H2 SER 1 31.746 32.745 1.816 1.00 0.00 H

ATOM 4 H3 SER 1 33.302 32.315 1.433 1.00 0.00 H

ATOM 5 CA SER 1 32.965 34.267 1.426 1.00 0.00 C

ATOM 6 HA SER 1 32.209 35.040 1.286 1.00 0.00 H

ATOM 7 CB SER 1 33.338 34.439 2.892 1.00 0.00 C

ATOM 8 HB2 SER 1 34.076 35.237 2.971 1.00 0.00 H

ATOM 9 HB3 SER 1 32.438 34.733 3.433 1.00 0.00 H

ATOM 10 OG SER 1 33.829 33.165 3.418 1.00 0.00 O

ATOM 11 HG SER 1 33.664 33.223 4.362 1.00 0.00 H

ATOM 12 C SER 1 34.239 34.497 0.635 1.00 0.00 C

ATOM 13 O SER 1 35.135 33.669 0.630 1.00 0.00 O

ATOM 14 N SER 2 34.262 35.545 -0.209 1.00 0.00 N

ATOM 15 H SER 2 33.479 36.178 -0.286 1.00 0.00 H

ATOM 16 CA SER 2 35.388 36.129 -0.930 1.00 0.00 C

ATOM 17 HA SER 2 36.223 35.443 -0.787 1.00 0.00 H

ATOM 18 CB SER 2 34.966 36.238 -2.414 1.00 0.00 C

ATOM 19 HB2 SER 2 33.988 36.709 -2.511 1.00 0.00 H

ATOM 20 HB3 SER 2 35.725 36.776 -2.983 1.00 0.00 H

ATOM 21 OG SER 2 34.888 34.879 -2.904 1.00 0.00 O

ATOM 22 HG SER 2 35.334 34.870 -3.754 1.00 0.00 H

ATOM 23 C SER 2 35.776 37.531 -0.405 1.00 0.00 C

ATOM 24 O SER 2 35.069 38.515 -0.575 1.00 0.00 O

ATOM 25 N PHE 3 36.925 37.582 0.295 1.00 0.00 N

ATOM 26 H PHE 3 37.619 36.881 0.074 1.00 0.00 H

ATOM 27 CA PHE 3 37.261 38.693 1.151 1.00 0.00 C

ATOM 28 HA PHE 3 36.378 39.122 1.624 1.00 0.00 H

ATOM 29 CB PHE 3 38.314 38.282 2.185 1.00 0.00 C

ATOM 30 HB2 PHE 3 39.172 37.701 1.845 1.00 0.00 H

ATOM 31 HB3 PHE 3 38.757 39.160 2.655 1.00 0.00 H

ATOM 32 CG PHE 3 37.671 37.519 3.249 1.00 0.00 C

ATOM 33 CD1 PHE 3 37.426 36.193 3.136 1.00 0.00 C

ATOM 34 HD1 PHE 3 37.732 35.650 2.255 1.00 0.00 H

ATOM 35 CE1 PHE 3 36.672 35.537 4.066 1.00 0.00 C

ATOM 36 HE1 PHE 3 36.326 34.543 3.825 1.00 0.00 H

ATOM 37 CZ PHE 3 36.069 36.221 5.132 1.00 0.00 C

ATOM 38 HZ PHE 3 35.326 35.767 5.770 1.00 0.00 H

ATOM 39 CE2 PHE 3 36.400 37.561 5.257 1.00 0.00 C

ATOM 40 HE2 PHE 3 35.875 38.248 5.904 1.00 0.00 H

ATOM 41 CD2 PHE 3 37.156 38.218 4.313 1.00 0.00 C

ATOM 42 HD2 PHE 3 37.356 39.276 4.396 1.00 0.00 H

ATOM 43 C PHE 3 37.882 39.760 0.274 1.00 0.00 C

ATOM 44 O PHE 3 38.786 39.625 -0.574 1.00 0.00 O

ATOM 45 N LYS 4 37.398 40.987 0.380 1.00 0.00 N

ATOM 46 H LYS 4 36.790 41.085 1.180 1.00 0.00 H

ATOM 47 CA LYS 4 37.506 42.063 -0.574 1.00 0.00 C

ATOM 48 HA LYS 4 38.438 41.968 -1.132 1.00 0.00 H

ATOM 49 CB LYS 4 36.280 41.938 -1.541 1.00 0.00 C

ATOM 50 HB2 LYS 4 36.491 42.665 -2.325 1.00 0.00 H

ATOM 51 HB3 LYS 4 36.134 40.966 -2.013 1.00 0.00 H

ATOM 52 CG LYS 4 34.987 42.336 -0.952 1.00 0.00 C

ATOM 53 HG2 LYS 4 34.556 41.435 -0.516 1.00 0.00 H

ATOM 54 HG3 LYS 4 35.194 43.183 -0.297 1.00 0.00 H

ATOM 55 CD LYS 4 34.008 42.852 -1.963 1.00 0.00 C

ATOM 56 HD2 LYS 4 33.167 43.427 -1.574 1.00 0.00 H

ATOM 57 HD3 LYS 4 34.464 43.749 -2.380 1.00 0.00 H

ATOM 58 CE LYS 4 33.754 41.854 -3.064 1.00 0.00 C

ATOM 59 HE2 LYS 4 33.618 42.389 -4.004 1.00 0.00 H

ATOM 60 HE3 LYS 4 34.594 41.167 -3.175 1.00 0.00 H

ATOM 61 NZ LYS 4 32.504 41.061 -2.810 1.00 0.00 N

ATOM 62 HZ1 LYS 4 31.660 41.616 -2.813 1.00 0.00 H

ATOM 63 HZ2 LYS 4 32.427 40.392 -3.563 1.00 0.00 H

ATOM 64 HZ3 LYS 4 32.496 40.602 -1.911 1.00 0.00 H

ATOM 65 C LYS 4 37.763 43.469 -0.064 1.00 0.00 C

ATOM 66 O LYS 4 37.477 44.508 -0.747 1.00 0.00 O

ATOM 67 N CYX 5 38.277 43.626 1.142 1.00 0.00 N

ATOM 68 H CYX 5 38.581 42.767 1.578 1.00 0.00 H

ATOM 69 CA CYX 5 38.439 44.868 1.830 1.00 0.00 C

ATOM 70 HA CYX 5 37.999 45.597 1.150 1.00 0.00 H

ATOM 71 CB CYX 5 37.485 44.831 3.015 1.00 0.00 C

ATOM 72 HB2 CYX 5 36.489 44.715 2.590 1.00 0.00 H

ATOM 73 HB3 CYX 5 37.665 43.930 3.600 1.00 0.00 H

ATOM 74 SG CYX 5 37.609 46.139 4.262 1.00 0.00 S

ATOM 75 C CYX 5 39.882 45.190 2.128 1.00 0.00 C

ATOM 76 O CYX 5 40.602 44.243 2.550 1.00 0.00 O

ATOM 77 N ALA 6 40.343 46.431 1.895 1.00 0.00 N

ATOM 78 H ALA 6 39.660 47.044 1.474 1.00 0.00 H

ATOM 79 CA ALA 6 41.520 47.075 2.316 1.00 0.00 C

ATOM 80 HA ALA 6 42.254 46.335 2.635 1.00 0.00 H

ATOM 81 CB ALA 6 42.082 47.829 1.104 1.00 0.00 C

ATOM 82 HB1 ALA 6 42.559 47.020 0.551 1.00 0.00 H

ATOM 83 HB2 ALA 6 41.357 48.321 0.455 1.00 0.00 H

ATOM 84 HB3 ALA 6 42.894 48.483 1.421 1.00 0.00 H

ATOM 85 C ALA 6 41.326 47.961 3.624 1.00 0.00 C

ATOM 86 O ALA 6 40.974 49.165 3.553 1.00 0.00 O

ATOM 87 N LEU 7 41.847 47.351 4.725 1.00 0.00 N

ATOM 88 H LEU 7 42.141 46.385 4.719 1.00 0.00 H

ATOM 89 CA LEU 7 42.269 48.021 5.872 1.00 0.00 C

ATOM 90 HA LEU 7 41.385 48.644 6.017 1.00 0.00 H

ATOM 91 CB LEU 7 42.423 47.088 7.131 1.00 0.00 C

ATOM 92 HB2 LEU 7 43.368 46.548 7.076 1.00 0.00 H

ATOM 93 HB3 LEU 7 42.656 47.774 7.945 1.00 0.00 H

ATOM 94 CG LEU 7 41.285 46.142 7.406 1.00 0.00 C

ATOM 95 HG LEU 7 41.478 45.194 6.905 1.00 0.00 H

ATOM 96 CD1 LEU 7 41.480 45.822 8.919 1.00 0.00 C

ATOM 97 HD11 LEU 7 42.500 45.482 9.098 1.00 0.00 H

ATOM 98 HD12 LEU 7 41.339 46.695 9.556 1.00 0.00 H

ATOM 99 HD13 LEU 7 40.843 44.993 9.225 1.00 0.00 H

ATOM 100 CD2 LEU 7 39.854 46.561 7.259 1.00 0.00 C

ATOM 101 HD21 LEU 7 39.220 45.678 7.336 1.00 0.00 H

ATOM 102 HD22 LEU 7 39.534 47.318 7.975 1.00 0.00 H

ATOM 103 HD23 LEU 7 39.700 46.906 6.236 1.00 0.00 H

ATOM 104 C LEU 7 43.510 48.869 5.586 1.00 0.00 C

ATOM 105 O LEU 7 44.478 48.312 5.079 1.00 0.00 O

ATOM 106 N THR 8 43.530 50.121 5.981 1.00 0.00 N

ATOM 107 H THR 8 42.660 50.390 6.419 1.00 0.00 H

ATOM 108 CA THR 8 44.666 51.056 5.953 1.00 0.00 C

ATOM 109 HA THR 8 44.844 51.196 4.887 1.00 0.00 H

ATOM 110 CB THR 8 44.319 52.442 6.388 1.00 0.00 C

ATOM 111 HB THR 8 43.934 52.419 7.407 1.00 0.00 H

ATOM 112 CG2 THR 8 45.337 53.561 6.385 1.00 0.00 C

ATOM 113 HG21 THR 8 45.633 53.938 5.406 1.00 0.00 H

ATOM 114 HG22 THR 8 45.034 54.482 6.883 1.00 0.00 H

ATOM 115 HG23 THR 8 46.166 53.261 7.026 1.00 0.00 H

ATOM 116 OG1 THR 8 43.191 52.783 5.601 1.00 0.00 O

ATOM 117 HG1 THR 8 42.449 52.502 6.142 1.00 0.00 H

ATOM 118 C THR 8 45.952 50.469 6.644 1.00 0.00 C

ATOM 119 O THR 8 46.085 50.656 7.868 1.00 0.00 O

ATOM 120 N LYS 9 46.790 49.691 5.992 1.00 0.00 N

ATOM 121 H LYS 9 46.492 49.355 5.087 1.00 0.00 H

ATOM 122 CA LYS 9 48.085 49.094 6.469 1.00 0.00 C

ATOM 123 HA LYS 9 47.792 48.488 7.326 1.00 0.00 H

ATOM 124 CB LYS 9 48.576 48.137 5.424 1.00 0.00 C

ATOM 125 HB2 LYS 9 47.918 47.289 5.235 1.00 0.00 H

ATOM 126 HB3 LYS 9 48.766 48.788 4.570 1.00 0.00 H

ATOM 127 CG LYS 9 49.891 47.460 5.969 1.00 0.00 C

ATOM 128 HG2 LYS 9 50.614 48.239 6.211 1.00 0.00 H

ATOM 129 HG3 LYS 9 49.666 46.884 6.867 1.00 0.00 H

ATOM 130 CD LYS 9 50.377 46.532 4.866 1.00 0.00 C

ATOM 131 HD2 LYS 9 49.556 45.999 4.388 1.00 0.00 H

ATOM 132 HD3 LYS 9 50.805 47.231 4.146 1.00 0.00 H

ATOM 133 CE LYS 9 51.490 45.577 5.272 1.00 0.00 C

ATOM 134 HE2 LYS 9 52.138 46.081 5.990 1.00 0.00 H

ATOM 135 HE3 LYS 9 51.100 44.686 5.764 1.00 0.00 H

ATOM 136 NZ LYS 9 52.300 45.143 4.125 1.00 0.00 N

ATOM 137 HZ1 LYS 9 52.401 44.140 4.075 1.00 0.00 H

ATOM 138 HZ2 LYS 9 51.905 45.345 3.217 1.00 0.00 H

ATOM 139 HZ3 LYS 9 53.164 45.664 4.074 1.00 0.00 H

ATOM 140 C LYS 9 49.168 50.054 7.019 1.00 0.00 C

ATOM 141 O LYS 9 49.620 49.872 8.172 1.00 0.00 O

ATOM 142 N THR 10 49.260 51.183 6.304 1.00 0.00 N

ATOM 143 H THR 10 48.750 51.231 5.433 1.00 0.00 H

ATOM 144 CA THR 10 50.420 52.124 6.455 1.00 0.00 C

ATOM 145 HA THR 10 51.070 51.727 7.234 1.00 0.00 H

ATOM 146 CB THR 10 51.334 52.226 5.258 1.00 0.00 C

ATOM 147 HB THR 10 52.180 52.907 5.345 1.00 0.00 H

ATOM 148 CG2 THR 10 51.975 50.891 4.904 1.00 0.00 C

ATOM 149 HG21 THR 10 52.756 51.009 4.153 1.00 0.00 H

ATOM 150 HG22 THR 10 52.460 50.406 5.751 1.00 0.00 H

ATOM 151 HG23 THR 10 51.270 50.171 4.489 1.00 0.00 H

ATOM 152 OG1 THR 10 50.750 52.478 4.122 1.00 0.00 O

ATOM 153 HG1 THR 10 51.446 52.848 3.574 1.00 0.00 H

ATOM 154 C THR 10 50.041 53.492 6.990 1.00 0.00 C

ATOM 155 O THR 10 50.484 53.873 8.089 1.00 0.00 O

ATOM 156 N GLY 11 49.199 54.198 6.257 1.00 0.00 N

ATOM 157 H GLY 11 49.302 54.087 5.259 1.00 0.00 H

ATOM 158 CA GLY 11 48.505 55.375 6.749 1.00 0.00 C

ATOM 159 HA2 GLY 11 48.075 55.818 5.850 1.00 0.00 H

ATOM 160 HA3 GLY 11 47.637 55.132 7.361 1.00 0.00 H

ATOM 161 C GLY 11 49.335 56.390 7.544 1.00 0.00 C

ATOM 162 O GLY 11 50.355 56.911 7.111 1.00 0.00 O

ATOM 163 N PHE 12 48.851 56.854 8.660 1.00 0.00 N

ATOM 164 H PHE 12 48.008 56.420 9.008 1.00 0.00 H

ATOM 165 CA PHE 12 49.677 57.408 9.748 1.00 0.00 C

ATOM 166 HA PHE 12 50.456 58.085 9.398 1.00 0.00 H

ATOM 167 CB PHE 12 48.880 58.471 10.545 1.00 0.00 C

ATOM 168 HB2 PHE 12 48.183 58.016 11.249 1.00 0.00 H

ATOM 169 HB3 PHE 12 49.588 58.877 11.267 1.00 0.00 H

ATOM 170 CG PHE 12 48.183 59.528 9.692 1.00 0.00 C

ATOM 171 CD1 PHE 12 46.811 59.365 9.437 1.00 0.00 C

ATOM 172 HD1 PHE 12 46.214 58.630 9.956 1.00 0.00 H

ATOM 173 CE1 PHE 12 46.140 60.298 8.600 1.00 0.00 C

ATOM 174 HE1 PHE 12 45.114 60.099 8.326 1.00 0.00 H

ATOM 175 CZ PHE 12 46.860 61.274 7.995 1.00 0.00 C

ATOM 176 HZ PHE 12 46.367 61.985 7.347 1.00 0.00 H

ATOM 177 CE2 PHE 12 48.154 61.560 8.379 1.00 0.00 C

ATOM 178 HE2 PHE 12 48.592 62.473 8.004 1.00 0.00 H

ATOM 179 CD2 PHE 12 48.850 60.635 9.138 1.00 0.00 C

ATOM 180 HD2 PHE 12 49.892 60.844 9.331 1.00 0.00 H

ATOM 181 C PHE 12 50.432 56.395 10.581 1.00 0.00 C

ATOM 182 O PHE 12 51.528 56.535 11.111 1.00 0.00 O

ATOM 183 N GLN 13 49.818 55.232 10.864 1.00 0.00 N

ATOM 184 H GLN 13 48.873 55.344 10.527 1.00 0.00 H

ATOM 185 CA GLN 13 50.153 54.355 11.987 1.00 0.00 C

ATOM 186 HA GLN 13 50.042 54.896 12.926 1.00 0.00 H

ATOM 187 CB GLN 13 49.103 53.275 12.081 1.00 0.00 C

ATOM 188 HB2 GLN 13 49.154 52.794 13.059 1.00 0.00 H

ATOM 189 HB3 GLN 13 48.113 53.731 12.097 1.00 0.00 H

ATOM 190 CG GLN 13 49.195 52.298 10.866 1.00 0.00 C

ATOM 191 HG2 GLN 13 48.511 52.566 10.061 1.00 0.00 H

ATOM 192 HG3 GLN 13 50.133 52.094 10.348 1.00 0.00 H

ATOM 193 CD GLN 13 48.788 50.908 11.348 1.00 0.00 C

ATOM 194 OE1 GLN 13 49.010 50.558 12.488 1.00 0.00 O

ATOM 195 NE2 GLN 13 48.057 50.216 10.507 1.00 0.00 N

ATOM 196 HE21 GLN 13 47.497 49.473 10.902 1.00 0.00 H

ATOM 197 HE22 GLN 13 48.122 50.375 9.512 1.00 0.00 H

ATOM 198 C GLN 13 51.562 53.946 11.997 1.00 0.00 C

ATOM 199 O GLN 13 52.187 53.893 13.083 1.00 0.00 O

ATOM 200 N PHE 14 52.146 53.596 10.831 1.00 0.00 N

ATOM 201 H PHE 14 51.619 53.726 9.980 1.00 0.00 H

ATOM 202 CA PHE 14 53.387 52.878 10.699 1.00 0.00 C

ATOM 203 HA PHE 14 53.386 51.996 11.340 1.00 0.00 H

ATOM 204 CB PHE 14 53.582 52.441 9.235 1.00 0.00 C

ATOM 205 HB2 PHE 14 53.806 51.381 9.115 1.00 0.00 H

ATOM 206 HB3 PHE 14 52.630 52.435 8.703 1.00 0.00 H

ATOM 207 CG PHE 14 54.589 53.318 8.413 1.00 0.00 C

ATOM 208 CD1 PHE 14 54.059 54.380 7.664 1.00 0.00 C

ATOM 209 HD1 PHE 14 52.993 54.558 7.669 1.00 0.00 H

ATOM 210 CE1 PHE 14 54.927 55.315 7.004 1.00 0.00 C

ATOM 211 HE1 PHE 14 54.426 56.188 6.612 1.00 0.00 H

ATOM 212 CZ PHE 14 56.298 55.186 7.138 1.00 0.00 C

ATOM 213 HZ PHE 14 56.916 55.847 6.549 1.00 0.00 H

ATOM 214 CE2 PHE 14 56.859 54.045 7.811 1.00 0.00 C

ATOM 215 HE2 PHE 14 57.883 53.708 7.754 1.00 0.00 H

ATOM 216 CD2 PHE 14 55.977 53.130 8.436 1.00 0.00 C

ATOM 217 HD2 PHE 14 56.349 52.189 8.816 1.00 0.00 H

ATOM 218 C PHE 14 54.525 53.726 11.251 1.00 0.00 C

ATOM 219 O PHE 14 55.609 53.167 11.438 1.00 0.00 O

ATOM 220 N TYR 15 54.399 55.080 11.256 1.00 0.00 N

ATOM 221 H TYR 15 53.490 55.395 10.948 1.00 0.00 H

ATOM 222 CA TYR 15 55.374 55.971 11.940 1.00 0.00 C

ATOM 223 HA TYR 15 56.214 55.368 12.286 1.00 0.00 H

ATOM 224 CB TYR 15 55.986 57.172 11.079 1.00 0.00 C

ATOM 225 HB2 TYR 15 56.826 57.681 11.551 1.00 0.00 H

ATOM 226 HB3 TYR 15 56.420 56.893 10.119 1.00 0.00 H

ATOM 227 CG TYR 15 55.044 58.311 10.832 1.00 0.00 C

ATOM 228 CD1 TYR 15 55.090 59.458 11.593 1.00 0.00 C

ATOM 229 HD1 TYR 15 55.882 59.657 12.299 1.00 0.00 H

ATOM 230 CE1 TYR 15 54.030 60.362 11.447 1.00 0.00 C

ATOM 231 HE1 TYR 15 54.033 61.217 12.106 1.00 0.00 H

ATOM 232 CZ TYR 15 53.033 60.184 10.521 1.00 0.00 C

ATOM 233 OH TYR 15 52.068 61.128 10.300 1.00 0.00 O

ATOM 234 HH TYR 15 52.113 61.976 10.748 1.00 0.00 H

ATOM 235 CE2 TYR 15 52.995 58.965 9.787 1.00 0.00 C

ATOM 236 HE2 TYR 15 52.227 58.831 9.040 1.00 0.00 H

ATOM 237 CD2 TYR 15 53.933 57.982 9.979 1.00 0.00 C

ATOM 238 HD2 TYR 15 53.853 57.033 9.469 1.00 0.00 H

ATOM 239 C TYR 15 54.960 56.561 13.215 1.00 0.00 C

ATOM 240 O TYR 15 55.775 56.472 14.167 1.00 0.00 O

ATOM 241 N TYR 16 53.673 56.735 13.415 1.00 0.00 N

ATOM 242 H TYR 16 53.136 56.683 12.561 1.00 0.00 H

ATOM 243 CA TYR 16 53.097 57.146 14.700 1.00 0.00 C

ATOM 244 HA TYR 16 53.650 58.037 14.997 1.00 0.00 H

ATOM 245 CB TYR 16 51.569 57.648 14.622 1.00 0.00 C

ATOM 246 HB2 TYR 16 51.237 57.416 13.610 1.00 0.00 H

ATOM 247 HB3 TYR 16 50.991 57.110 15.374 1.00 0.00 H

ATOM 248 CG TYR 16 51.335 59.115 14.957 1.00 0.00 C

ATOM 249 CD1 TYR 16 51.384 59.991 13.901 1.00 0.00 C

ATOM 250 HD1 TYR 16 51.681 59.561 12.956 1.00 0.00 H

ATOM 251 CE1 TYR 16 50.679 61.166 13.987 1.00 0.00 C

ATOM 252 HE1 TYR 16 50.546 61.769 13.101 1.00 0.00 H

ATOM 253 CZ TYR 16 50.406 61.720 15.271 1.00 0.00 C

ATOM 254 OH TYR 16 50.190 63.067 15.422 1.00 0.00 O

ATOM 255 HH TYR 16 50.373 63.631 14.666 1.00 0.00 H

ATOM 256 CE2 TYR 16 50.372 60.844 16.395 1.00 0.00 C

ATOM 257 HE2 TYR 16 49.900 61.114 17.328 1.00 0.00 H

ATOM 258 CD2 TYR 16 50.881 59.551 16.197 1.00 0.00 C

ATOM 259 HD2 TYR 16 51.024 58.912 17.056 1.00 0.00 H

ATOM 260 C TYR 16 53.396 56.325 16.012 1.00 0.00 C

ATOM 261 O TYR 16 53.610 56.804 17.087 1.00 0.00 O

ATOM 262 N LEU 17 53.205 55.026 15.797 1.00 0.00 N

ATOM 263 H LEU 17 52.926 54.673 14.892 1.00 0.00 H

ATOM 264 CA LEU 17 53.315 53.977 16.808 1.00 0.00 C

ATOM 265 HA LEU 17 52.875 54.395 17.713 1.00 0.00 H

ATOM 266 CB LEU 17 52.502 52.701 16.350 1.00 0.00 C

ATOM 267 HB2 LEU 17 52.894 52.462 15.362 1.00 0.00 H

ATOM 268 HB3 LEU 17 52.774 51.795 16.892 1.00 0.00 H

ATOM 269 CG LEU 17 50.941 52.774 16.477 1.00 0.00 C

ATOM 270 HG LEU 17 50.671 53.728 16.024 1.00 0.00 H

ATOM 271 CD1 LEU 17 50.205 51.810 15.664 1.00 0.00 C

ATOM 272 HD11 LEU 17 50.305 50.833 16.137 1.00 0.00 H

ATOM 273 HD12 LEU 17 49.171 52.144 15.585 1.00 0.00 H

ATOM 274 HD13 LEU 17 50.634 51.870 14.664 1.00 0.00 H

ATOM 275 CD2 LEU 17 50.416 52.903 17.904 1.00 0.00 C

ATOM 276 HD21 LEU 17 50.073 53.874 18.259 1.00 0.00 H

ATOM 277 HD22 LEU 17 49.565 52.236 18.041 1.00 0.00 H

ATOM 278 HD23 LEU 17 51.161 52.458 18.564 1.00 0.00 H

ATOM 279 C LEU 17 54.743 53.738 17.199 1.00 0.00 C

ATOM 280 O LEU 17 54.970 53.942 18.344 1.00 0.00 O

ATOM 281 N PRO 18 55.729 53.723 16.279 1.00 0.00 N

ATOM 282 CD PRO 18 55.572 53.212 14.908 1.00 0.00 C

ATOM 283 HD2 PRO 18 54.897 53.835 14.322 1.00 0.00 H

ATOM 284 HD3 PRO 18 55.056 52.253 14.955 1.00 0.00 H

ATOM 285 CG PRO 18 56.982 53.025 14.325 1.00 0.00 C

ATOM 286 HG2 PRO 18 57.195 53.193 13.269 1.00 0.00 H

ATOM 287 HG3 PRO 18 57.430 52.045 14.490 1.00 0.00 H

ATOM 288 CB PRO 18 57.823 54.018 15.270 1.00 0.00 C

ATOM 289 HB2 PRO 18 57.869 55.028 14.863 1.00 0.00 H

ATOM 290 HB3 PRO 18 58.856 53.670 15.298 1.00 0.00 H

ATOM 291 CA PRO 18 57.103 53.895 16.611 1.00 0.00 C

ATOM 292 HA PRO 18 57.461 53.014 17.143 1.00 0.00 H

ATOM 293 C PRO 18 57.516 55.095 17.490 1.00 0.00 C

ATOM 294 O PRO 18 58.227 54.964 18.497 1.00 0.00 O

ATOM 295 N ALA 19 56.884 56.220 17.188 1.00 0.00 N

ATOM 296 H ALA 19 56.372 56.239 16.318 1.00 0.00 H

ATOM 297 CA ALA 19 56.932 57.501 17.987 1.00 0.00 C

ATOM 298 HA ALA 19 57.964 57.849 18.049 1.00 0.00 H

ATOM 299 CB ALA 19 56.252 58.609 17.199 1.00 0.00 C

ATOM 300 HB1 ALA 19 56.777 59.563 17.148 1.00 0.00 H

ATOM 301 HB2 ALA 19 56.168 58.219 16.185 1.00 0.00 H

ATOM 302 HB3 ALA 19 55.266 58.731 17.646 1.00 0.00 H

ATOM 303 C ALA 19 56.366 57.248 19.419 1.00 0.00 C

ATOM 304 O ALA 19 57.196 57.368 20.362 1.00 0.00 O

ATOM 305 N VAL 20 55.089 56.840 19.523 1.00 0.00 N

ATOM 306 H VAL 20 54.654 56.478 18.687 1.00 0.00 H

ATOM 307 CA VAL 20 54.324 56.544 20.737 1.00 0.00 C

ATOM 308 HA VAL 20 54.283 57.458 21.329 1.00 0.00 H

ATOM 309 CB VAL 20 52.885 56.046 20.269 1.00 0.00 C

ATOM 310 HB VAL 20 52.922 55.624 19.265 1.00 0.00 H

ATOM 311 CG1 VAL 20 52.210 54.978 21.225 1.00 0.00 C

ATOM 312 HG11 VAL 20 52.532 53.950 21.064 1.00 0.00 H

ATOM 313 HG12 VAL 20 52.451 55.141 22.275 1.00 0.00 H

ATOM 314 HG13 VAL 20 51.129 54.969 21.082 1.00 0.00 H

ATOM 315 CG2 VAL 20 51.727 57.112 20.006 1.00 0.00 C

ATOM 316 HG21 VAL 20 50.926 56.535 19.544 1.00 0.00 H

ATOM 317 HG22 VAL 20 51.543 57.572 20.977 1.00 0.00 H

ATOM 318 HG23 VAL 20 52.199 57.826 19.330 1.00 0.00 H

ATOM 319 C VAL 20 55.120 55.569 21.569 1.00 0.00 C

ATOM 320 O VAL 20 55.422 55.853 22.661 1.00 0.00 O

ATOM 321 N TYR 21 55.571 54.428 20.992 1.00 0.00 N

ATOM 322 H TYR 21 55.180 54.248 20.078 1.00 0.00 H

ATOM 323 CA TYR 21 56.109 53.257 21.707 1.00 0.00 C

ATOM 324 HA TYR 21 55.480 53.115 22.586 1.00 0.00 H

ATOM 325 CB TYR 21 55.982 51.933 20.983 1.00 0.00 C

ATOM 326 HB2 TYR 21 56.422 52.049 19.993 1.00 0.00 H

ATOM 327 HB3 TYR 21 56.551 51.228 21.589 1.00 0.00 H

ATOM 328 CG TYR 21 54.655 51.302 20.765 1.00 0.00 C

ATOM 329 CD1 TYR 21 54.354 50.732 19.485 1.00 0.00 C

ATOM 330 HD1 TYR 21 55.012 50.797 18.632 1.00 0.00 H

ATOM 331 CE1 TYR 21 53.063 50.334 19.161 1.00 0.00 C

ATOM 332 HE1 TYR 21 52.918 49.857 18.203 1.00 0.00 H

ATOM 333 CZ TYR 21 52.006 50.414 20.154 1.00 0.00 C

ATOM 334 OH TYR 21 50.814 49.960 19.775 1.00 0.00 O

ATOM 335 HH TYR 21 50.238 49.880 20.538 1.00 0.00 H

ATOM 336 CE2 TYR 21 52.374 50.906 21.423 1.00 0.00 C

ATOM 337 HE2 TYR 21 51.608 51.035 22.173 1.00 0.00 H

ATOM 338 CD2 TYR 21 53.708 51.265 21.796 1.00 0.00 C

ATOM 339 HD2 TYR 21 53.919 51.692 22.766 1.00 0.00 H

ATOM 340 C TYR 21 57.573 53.442 22.062 1.00 0.00 C

ATOM 341 O TYR 21 58.038 52.946 23.038 1.00 0.00 O

ATOM 342 N ILE 22 58.413 54.100 21.241 1.00 0.00 N

ATOM 343 H ILE 22 58.048 54.558 20.418 1.00 0.00 H

ATOM 344 CA ILE 22 59.849 54.386 21.571 1.00 0.00 C

ATOM 345 HA ILE 22 60.232 53.439 21.950 1.00 0.00 H

ATOM 346 CB ILE 22 60.741 54.769 20.354 1.00 0.00 C

ATOM 347 HB ILE 22 60.355 54.232 19.487 1.00 0.00 H

ATOM 348 CG2 ILE 22 60.710 56.207 19.985 1.00 0.00 C

ATOM 349 HG21 ILE 22 59.707 56.634 19.954 1.00 0.00 H

ATOM 350 HG22 ILE 22 61.154 56.828 20.763 1.00 0.00 H

ATOM 351 HG23 ILE 22 61.158 56.492 19.033 1.00 0.00 H

ATOM 352 CG1 ILE 22 62.217 54.399 20.626 1.00 0.00 C

ATOM 353 HG12 ILE 22 62.717 55.303 20.975 1.00 0.00 H

ATOM 354 HG13 ILE 22 62.311 53.704 21.460 1.00 0.00 H

ATOM 355 CD1 ILE 22 62.867 54.000 19.302 1.00 0.00 C

ATOM 356 HD11 ILE 22 63.811 53.634 19.707 1.00 0.00 H

ATOM 357 HD12 ILE 22 62.317 53.254 18.728 1.00 0.00 H

ATOM 358 HD13 ILE 22 62.931 54.903 18.695 1.00 0.00 H

ATOM 359 C ILE 22 59.971 55.173 22.894 1.00 0.00 C

ATOM 360 O ILE 22 60.759 54.779 23.766 1.00 0.00 O

ATOM 361 N LEU 23 59.112 56.170 23.098 1.00 0.00 N

ATOM 362 H LEU 23 58.549 56.531 22.341 1.00 0.00 H

ATOM 363 CA LEU 23 59.227 57.014 24.330 1.00 0.00 C

ATOM 364 HA LEU 23 60.243 57.387 24.464 1.00 0.00 H

ATOM 365 CB LEU 23 58.319 58.242 24.221 1.00 0.00 C

ATOM 366 HB2 LEU 23 58.265 58.461 23.155 1.00 0.00 H

ATOM 367 HB3 LEU 23 57.314 58.003 24.570 1.00 0.00 H

ATOM 368 CG LEU 23 58.793 59.500 24.970 1.00 0.00 C

ATOM 369 HG LEU 23 59.877 59.616 24.944 1.00 0.00 H

ATOM 370 CD1 LEU 23 58.349 60.758 24.274 1.00 0.00 C

ATOM 371 HD11 LEU 23 59.055 60.846 23.448 1.00 0.00 H

ATOM 372 HD12 LEU 23 57.348 60.515 23.917 1.00 0.00 H

ATOM 373 HD13 LEU 23 58.318 61.680 24.853 1.00 0.00 H

ATOM 374 CD2 LEU 23 58.317 59.431 26.398 1.00 0.00 C

ATOM 375 HD21 LEU 23 58.070 60.464 26.644 1.00 0.00 H

ATOM 376 HD22 LEU 23 57.460 58.761 26.456 1.00 0.00 H

ATOM 377 HD23 LEU 23 59.067 59.000 27.061 1.00 0.00 H

ATOM 378 C LEU 23 58.864 56.144 25.564 1.00 0.00 C

ATOM 379 O LEU 23 59.553 56.213 26.550 1.00 0.00 O

ATOM 380 N VAL 24 57.753 55.358 25.539 1.00 0.00 N

ATOM 381 H VAL 24 57.329 55.230 24.631 1.00 0.00 H

ATOM 382 CA VAL 24 57.495 54.410 26.733 1.00 0.00 C

ATOM 383 HA VAL 24 57.435 54.958 27.673 1.00 0.00 H

ATOM 384 CB VAL 24 56.095 53.869 26.599 1.00 0.00 C

ATOM 385 HB VAL 24 56.271 53.027 25.930 1.00 0.00 H

ATOM 386 CG1 VAL 24 55.376 53.412 27.903 1.00 0.00 C

ATOM 387 HG11 VAL 24 56.092 52.849 28.502 1.00 0.00 H

ATOM 388 HG12 VAL 24 54.909 54.212 28.476 1.00 0.00 H

ATOM 389 HG13 VAL 24 54.645 52.660 27.606 1.00 0.00 H

ATOM 390 CG2 VAL 24 55.113 54.881 25.980 1.00 0.00 C

ATOM 391 HG21 VAL 24 55.133 55.891 26.390 1.00 0.00 H

ATOM 392 HG22 VAL 24 55.254 54.995 24.905 1.00 0.00 H

ATOM 393 HG23 VAL 24 54.081 54.562 26.124 1.00 0.00 H

ATOM 394 C VAL 24 58.473 53.245 26.867 1.00 0.00 C

ATOM 395 O VAL 24 58.764 52.894 28.036 1.00 0.00 O

ATOM 396 N PHE 25 59.037 52.811 25.735 1.00 0.00 N

ATOM 397 H PHE 25 58.581 52.950 24.845 1.00 0.00 H

ATOM 398 CA PHE 25 60.042 51.767 25.748 1.00 0.00 C

ATOM 399 HA PHE 25 59.494 50.921 26.163 1.00 0.00 H

ATOM 400 CB PHE 25 60.452 51.266 24.333 1.00 0.00 C

ATOM 401 HB2 PHE 25 59.617 50.691 23.933 1.00 0.00 H

ATOM 402 HB3 PHE 25 60.649 52.160 23.741 1.00 0.00 H

ATOM 403 CG PHE 25 61.712 50.372 24.254 1.00 0.00 C

ATOM 404 CD1 PHE 25 61.626 49.151 24.939 1.00 0.00 C

ATOM 405 HD1 PHE 25 60.649 48.893 25.321 1.00 0.00 H

ATOM 406 CE1 PHE 25 62.830 48.395 25.002 1.00 0.00 C

ATOM 407 HE1 PHE 25 62.716 47.347 25.235 1.00 0.00 H

ATOM 408 CZ PHE 25 64.056 48.846 24.389 1.00 0.00 C

ATOM 409 HZ PHE 25 64.871 48.170 24.174 1.00 0.00 H

ATOM 410 CE2 PHE 25 64.132 50.104 23.869 1.00 0.00 C

ATOM 411 HE2 PHE 25 65.073 50.458 23.474 1.00 0.00 H

ATOM 412 CD2 PHE 25 62.935 50.798 23.745 1.00 0.00 C

ATOM 413 HD2 PHE 25 63.053 51.871 23.723 1.00 0.00 H

ATOM 414 C PHE 25 61.328 52.165 26.596 1.00 0.00 C

ATOM 415 O PHE 25 61.654 51.455 27.517 1.00 0.00 O

ATOM 416 N ILE 26 61.960 53.335 26.325 1.00 0.00 N

ATOM 417 H ILE 26 61.476 54.010 25.751 1.00 0.00 H

ATOM 418 CA ILE 26 63.181 53.773 26.993 1.00 0.00 C

ATOM 419 HA ILE 26 64.033 53.099 26.908 1.00 0.00 H

ATOM 420 CB ILE 26 63.680 55.079 26.305 1.00 0.00 C

ATOM 421 HB ILE 26 63.828 54.767 25.271 1.00 0.00 H

ATOM 422 CG2 ILE 26 62.758 56.283 26.174 1.00 0.00 C

ATOM 423 HG21 ILE 26 61.846 55.960 25.671 1.00 0.00 H

ATOM 424 HG22 ILE 26 62.660 56.766 27.146 1.00 0.00 H

ATOM 425 HG23 ILE 26 63.432 56.970 25.663 1.00 0.00 H

ATOM 426 CG1 ILE 26 65.070 55.396 26.772 1.00 0.00 C

ATOM 427 HG12 ILE 26 65.585 54.444 26.901 1.00 0.00 H

ATOM 428 HG13 ILE 26 65.658 55.908 26.010 1.00 0.00 H

ATOM 429 CD1 ILE 26 65.289 56.228 28.124 1.00 0.00 C

ATOM 430 HD11 ILE 26 66.310 56.075 28.476 1.00 0.00 H

ATOM 431 HD12 ILE 26 65.308 57.277 27.826 1.00 0.00 H

ATOM 432 HD13 ILE 26 64.446 56.131 28.807 1.00 0.00 H

ATOM 433 C ILE 26 63.079 53.953 28.522 1.00 0.00 C

ATOM 434 O ILE 26 64.006 53.536 29.291 1.00 0.00 O

ATOM 435 N ILE 27 61.903 54.505 28.998 1.00 0.00 N

ATOM 436 H ILE 27 61.156 54.619 28.328 1.00 0.00 H

ATOM 437 CA ILE 27 61.706 54.825 30.413 1.00 0.00 C

ATOM 438 HA ILE 27 62.643 55.076 30.912 1.00 0.00 H

ATOM 439 CB ILE 27 60.737 56.003 30.696 1.00 0.00 C

ATOM 440 HB ILE 27 60.713 56.041 31.785 1.00 0.00 H

ATOM 441 CG2 ILE 27 61.282 57.382 30.198 1.00 0.00 C

ATOM 442 HG21 ILE 27 60.715 58.180 30.678 1.00 0.00 H

ATOM 443 HG22 ILE 27 62.340 57.442 30.454 1.00 0.00 H

ATOM 444 HG23 ILE 27 61.194 57.430 29.113 1.00 0.00 H

ATOM 445 CG1 ILE 27 59.268 55.614 30.169 1.00 0.00 C

ATOM 446 HG12 ILE 27 59.190 55.551 29.084 1.00 0.00 H

ATOM 447 HG13 ILE 27 59.031 54.662 30.645 1.00 0.00 H

ATOM 448 CD1 ILE 27 58.141 56.624 30.685 1.00 0.00 C

ATOM 449 HD11 ILE 27 57.173 56.170 30.473 1.00 0.00 H

ATOM 450 HD12 ILE 27 58.228 56.864 31.744 1.00 0.00 H

ATOM 451 HD13 ILE 27 58.303 57.525 30.092 1.00 0.00 H

ATOM 452 C ILE 27 61.416 53.487 31.192 1.00 0.00 C

ATOM 453 O ILE 27 61.848 53.351 32.336 1.00 0.00 O

ATOM 454 N GLY 28 60.974 52.408 30.575 1.00 0.00 N

ATOM 455 H GLY 28 60.637 52.594 29.641 1.00 0.00 H

ATOM 456 CA GLY 28 60.925 51.063 31.171 1.00 0.00 C

ATOM 457 HA2 GLY 28 60.552 51.095 32.195 1.00 0.00 H

ATOM 458 HA3 GLY 28 60.201 50.397 30.701 1.00 0.00 H

ATOM 459 C GLY 28 62.279 50.278 31.149 1.00 0.00 C

ATOM 460 O GLY 28 62.653 49.876 32.222 1.00 0.00 O

ATOM 461 N PHE 29 62.970 50.227 29.985 1.00 0.00 N

ATOM 462 H PHE 29 62.353 50.551 29.253 1.00 0.00 H

ATOM 463 CA PHE 29 64.250 49.666 29.687 1.00 0.00 C

ATOM 464 HA PHE 29 64.250 48.612 29.965 1.00 0.00 H

ATOM 465 CB PHE 29 64.466 49.863 28.222 1.00 0.00 C

ATOM 466 HB2 PHE 29 63.559 49.684 27.644 1.00 0.00 H

ATOM 467 HB3 PHE 29 64.887 50.857 28.075 1.00 0.00 H

ATOM 468 CG PHE 29 65.486 48.926 27.622 1.00 0.00 C

ATOM 469 CD1 PHE 29 65.133 47.567 27.554 1.00 0.00 C

ATOM 470 HD1 PHE 29 64.215 47.297 28.056 1.00 0.00 H

ATOM 471 CE1 PHE 29 65.952 46.699 26.794 1.00 0.00 C

ATOM 472 HE1 PHE 29 65.630 45.684 26.612 1.00 0.00 H

ATOM 473 CZ PHE 29 66.983 47.185 26.080 1.00 0.00 C

ATOM 474 HZ PHE 29 67.558 46.500 25.475 1.00 0.00 H

ATOM 475 CE2 PHE 29 67.427 48.533 26.175 1.00 0.00 C

ATOM 476 HE2 PHE 29 68.383 48.916 25.852 1.00 0.00 H

ATOM 477 CD2 PHE 29 66.626 49.427 26.950 1.00 0.00 C

ATOM 478 HD2 PHE 29 67.011 50.419 27.133 1.00 0.00 H

ATOM 479 C PHE 29 65.263 50.230 30.680 1.00 0.00 C

ATOM 480 O PHE 29 66.179 49.541 31.104 1.00 0.00 O

ATOM 481 N LEU 30 65.252 51.542 31.016 1.00 0.00 N

ATOM 482 H LEU 30 64.861 52.123 30.289 1.00 0.00 H

ATOM 483 CA LEU 30 66.106 52.241 32.031 1.00 0.00 C

ATOM 484 HA LEU 30 67.052 51.705 32.098 1.00 0.00 H

ATOM 485 CB LEU 30 66.314 53.676 31.642 1.00 0.00 C

ATOM 486 HB2 LEU 30 66.736 53.695 30.638 1.00 0.00 H

ATOM 487 HB3 LEU 30 65.279 53.993 31.514 1.00 0.00 H

ATOM 488 CG LEU 30 67.127 54.630 32.540 1.00 0.00 C

ATOM 489 HG LEU 30 66.688 54.782 33.526 1.00 0.00 H

ATOM 490 CD1 LEU 30 68.536 54.113 32.766 1.00 0.00 C

ATOM 491 HD11 LEU 30 68.925 54.120 31.748 1.00 0.00 H

ATOM 492 HD12 LEU 30 69.097 54.845 33.347 1.00 0.00 H

ATOM 493 HD13 LEU 30 68.567 53.143 33.261 1.00 0.00 H

ATOM 494 CD2 LEU 30 67.391 56.052 31.913 1.00 0.00 C

ATOM 495 HD21 LEU 30 66.628 56.278 31.168 1.00 0.00 H

ATOM 496 HD22 LEU 30 67.403 56.696 32.792 1.00 0.00 H

ATOM 497 HD23 LEU 30 68.334 56.185 31.381 1.00 0.00 H

ATOM 498 C LEU 30 65.647 51.929 33.414 1.00 0.00 C

ATOM 499 O LEU 30 66.475 51.582 34.285 1.00 0.00 O

ATOM 500 N GLY 31 64.336 51.977 33.672 1.00 0.00 N

ATOM 501 H GLY 31 63.822 52.105 32.812 1.00 0.00 H

ATOM 502 CA GLY 31 63.594 52.339 34.875 1.00 0.00 C

ATOM 503 HA2 GLY 31 64.243 52.845 35.590 1.00 0.00 H

ATOM 504 HA3 GLY 31 62.794 53.032 34.616 1.00 0.00 H

ATOM 505 C GLY 31 62.956 51.221 35.593 1.00 0.00 C

ATOM 506 O GLY 31 63.293 50.911 36.740 1.00 0.00 O

ATOM 507 N ASN 32 62.196 50.398 34.910 1.00 0.00 N

ATOM 508 H ASN 32 61.965 50.689 33.971 1.00 0.00 H

ATOM 509 CA ASN 32 61.735 49.131 35.421 1.00 0.00 C

ATOM 510 HA ASN 32 61.460 49.455 36.425 1.00 0.00 H

ATOM 511 CB ASN 32 60.459 48.652 34.716 1.00 0.00 C

ATOM 512 HB2 ASN 32 59.737 49.453 34.555 1.00 0.00 H

ATOM 513 HB3 ASN 32 60.674 48.307 33.705 1.00 0.00 H

ATOM 514 CG ASN 32 59.742 47.511 35.319 1.00 0.00 C

ATOM 515 OD1 ASN 32 59.583 47.372 36.551 1.00 0.00 O

ATOM 516 ND2 ASN 32 58.899 46.827 34.575 1.00 0.00 N

ATOM 517 HD21 ASN 32 58.899 46.863 33.566 1.00 0.00 H

ATOM 518 HD22 ASN 32 58.344 46.057 34.922 1.00 0.00 H

ATOM 519 C ASN 32 62.876 48.027 35.552 1.00 0.00 C

ATOM 520 O ASN 32 62.734 47.034 36.279 1.00 0.00 O

ATOM 521 N SER 33 63.900 48.167 34.683 1.00 0.00 N

ATOM 522 H SER 33 63.887 48.970 34.070 1.00 0.00 H

ATOM 523 CA SER 33 65.071 47.280 34.849 1.00 0.00 C

ATOM 524 HA SER 33 64.855 46.212 34.858 1.00 0.00 H

ATOM 525 CB SER 33 66.009 47.407 33.596 1.00 0.00 C

ATOM 526 HB2 SER 33 66.165 46.418 33.166 1.00 0.00 H

ATOM 527 HB3 SER 33 65.510 47.907 32.766 1.00 0.00 H

ATOM 528 OG SER 33 67.271 47.950 33.879 1.00 0.00 O

ATOM 529 HG SER 33 67.596 48.495 33.159 1.00 0.00 H

ATOM 530 C SER 33 65.828 47.484 36.181 1.00 0.00 C

ATOM 531 O SER 33 65.982 46.442 36.829 1.00 0.00 O

ATOM 532 N VAL 34 66.109 48.683 36.707 1.00 0.00 N

ATOM 533 H VAL 34 66.127 49.553 36.194 1.00 0.00 H

ATOM 534 CA VAL 34 66.565 48.869 38.101 1.00 0.00 C

ATOM 535 HA VAL 34 67.478 48.301 38.283 1.00 0.00 H

ATOM 536 CB VAL 34 66.899 50.331 38.383 1.00 0.00 C

ATOM 537 HB VAL 34 65.957 50.724 38.768 1.00 0.00 H

ATOM 538 CG1 VAL 34 68.069 50.628 39.313 1.00 0.00 C

ATOM 539 HG11 VAL 34 68.986 50.578 38.726 1.00 0.00 H

ATOM 540 HG12 VAL 34 67.914 51.616 39.746 1.00 0.00 H

ATOM 541 HG13 VAL 34 68.071 49.857 40.084 1.00 0.00 H

ATOM 542 CG2 VAL 34 67.071 51.267 37.182 1.00 0.00 C

ATOM 543 HG21 VAL 34 66.086 51.658 36.924 1.00 0.00 H

ATOM 544 HG22 VAL 34 67.764 52.052 37.482 1.00 0.00 H

ATOM 545 HG23 VAL 34 67.512 50.677 36.379 1.00 0.00 H

ATOM 546 C VAL 34 65.607 48.251 39.155 1.00 0.00 C

ATOM 547 O VAL 34 66.121 47.838 40.204 1.00 0.00 O

ATOM 548 N ALA 35 64.308 48.465 38.871 1.00 0.00 N

ATOM 549 H ALA 35 64.012 48.766 37.954 1.00 0.00 H

ATOM 550 CA ALA 35 63.224 47.966 39.793 1.00 0.00 C

ATOM 551 HA ALA 35 63.419 48.479 40.735 1.00 0.00 H

ATOM 552 CB ALA 35 61.882 48.515 39.251 1.00 0.00 C

ATOM 553 HB1 ALA 35 61.674 48.065 38.281 1.00 0.00 H

ATOM 554 HB2 ALA 35 61.130 48.155 39.953 1.00 0.00 H

ATOM 555 HB3 ALA 35 61.895 49.605 39.256 1.00 0.00 H

ATOM 556 C ALA 35 63.335 46.475 40.015 1.00 0.00 C

ATOM 557 O ALA 35 63.380 46.104 41.196 1.00 0.00 O

ATOM 558 N ILE 36 63.324 45.696 38.977 1.00 0.00 N

ATOM 559 H ILE 36 63.196 46.090 38.056 1.00 0.00 H

ATOM 560 CA ILE 36 63.385 44.290 39.143 1.00 0.00 C

ATOM 561 HA ILE 36 62.721 44.007 39.960 1.00 0.00 H

ATOM 562 CB ILE 36 62.992 43.522 37.897 1.00 0.00 C

ATOM 563 HB ILE 36 63.219 42.503 38.209 1.00 0.00 H

ATOM 564 CG2 ILE 36 61.441 43.565 37.706 1.00 0.00 C

ATOM 565 HG21 ILE 36 61.015 42.771 37.091 1.00 0.00 H

ATOM 566 HG22 ILE 36 60.995 43.570 38.700 1.00 0.00 H

ATOM 567 HG23 ILE 36 61.155 44.490 37.206 1.00 0.00 H

ATOM 568 CG1 ILE 36 63.823 43.867 36.641 1.00 0.00 C

ATOM 569 HG12 ILE 36 63.877 44.906 36.315 1.00 0.00 H

ATOM 570 HG13 ILE 36 64.866 43.643 36.866 1.00 0.00 H

ATOM 571 CD1 ILE 36 63.359 43.091 35.435 1.00 0.00 C

ATOM 572 HD11 ILE 36 62.316 43.344 35.245 1.00 0.00 H

ATOM 573 HD12 ILE 36 64.008 43.353 34.600 1.00 0.00 H

ATOM 574 HD13 ILE 36 63.536 42.046 35.689 1.00 0.00 H

ATOM 575 C ILE 36 64.751 43.745 39.690 1.00 0.00 C

ATOM 576 O ILE 36 64.831 42.723 40.302 1.00 0.00 O

ATOM 577 N TRP 37 65.802 44.536 39.528 1.00 0.00 N

ATOM 578 H TRP 37 65.763 45.275 38.841 1.00 0.00 H

ATOM 579 CA TRP 37 67.055 44.357 40.359 1.00 0.00 C

ATOM 580 HA TRP 37 67.345 43.308 40.298 1.00 0.00 H

ATOM 581 CB TRP 37 68.187 45.210 39.747 1.00 0.00 C

ATOM 582 HB2 TRP 37 68.081 45.293 38.665 1.00 0.00 H

ATOM 583 HB3 TRP 37 68.210 46.200 40.203 1.00 0.00 H

ATOM 584 CG TRP 37 69.463 44.616 40.031 1.00 0.00 C

ATOM 585 CD1 TRP 37 70.164 44.896 41.163 1.00 0.00 C

ATOM 586 HD1 TRP 37 70.036 45.677 41.897 1.00 0.00 H

ATOM 587 NE1 TRP 37 71.318 44.122 41.162 1.00 0.00 N

ATOM 588 HE1 TRP 37 71.796 44.102 42.051 1.00 0.00 H

ATOM 589 CE2 TRP 37 71.279 43.071 40.289 1.00 0.00 C

ATOM 590 CZ2 TRP 37 72.017 41.925 40.048 1.00 0.00 C

ATOM 591 HZ2 TRP 37 73.015 41.876 40.458 1.00 0.00 H

ATOM 592 CH2 TRP 37 71.616 41.149 38.994 1.00 0.00 C

ATOM 593 HH2 TRP 37 72.187 40.302 38.640 1.00 0.00 H

ATOM 594 CZ3 TRP 37 70.360 41.400 38.289 1.00 0.00 C

ATOM 595 HZ3 TRP 37 69.985 40.721 37.538 1.00 0.00 H

ATOM 596 CE3 TRP 37 69.561 42.515 38.642 1.00 0.00 C

ATOM 597 HE3 TRP 37 68.597 42.699 38.190 1.00 0.00 H

ATOM 598 CD2 TRP 37 70.042 43.373 39.586 1.00 0.00 C

ATOM 599 C TRP 37 66.859 44.659 41.855 1.00 0.00 C

ATOM 600 O TRP 37 66.977 43.749 42.729 1.00 0.00 O

ATOM 601 N MET 38 66.465 45.864 42.110 1.00 0.00 N

ATOM 602 H MET 38 66.171 46.473 41.360 1.00 0.00 H

ATOM 603 CA MET 38 66.311 46.348 43.511 1.00 0.00 C

ATOM 604 HA MET 38 67.306 46.231 43.940 1.00 0.00 H

ATOM 605 CB MET 38 65.923 47.813 43.709 1.00 0.00 C

ATOM 606 HB2 MET 38 64.902 47.969 43.362 1.00 0.00 H

ATOM 607 HB3 MET 38 65.919 48.036 44.776 1.00 0.00 H

ATOM 608 CG MET 38 66.845 48.845 42.970 1.00 0.00 C

ATOM 609 HG2 MET 38 67.502 48.411 42.216 1.00 0.00 H

ATOM 610 HG3 MET 38 66.157 49.486 42.417 1.00 0.00 H

ATOM 611 SD MET 38 67.803 49.786 44.270 1.00 0.00 S

ATOM 612 CE MET 38 67.012 51.393 44.024 1.00 0.00 C

ATOM 613 HE1 MET 38 67.578 52.132 44.591 1.00 0.00 H

ATOM 614 HE2 MET 38 66.827 51.594 42.969 1.00 0.00 H

ATOM 615 HE3 MET 38 66.099 51.277 44.609 1.00 0.00 H

ATOM 616 C MET 38 65.337 45.487 44.361 1.00 0.00 C

ATOM 617 O MET 38 65.667 45.179 45.516 1.00 0.00 O

ATOM 618 N PHE 39 64.280 45.006 43.699 1.00 0.00 N

ATOM 619 H PHE 39 64.168 45.357 42.758 1.00 0.00 H

ATOM 620 CA PHE 39 63.252 44.130 44.228 1.00 0.00 C

ATOM 621 HA PHE 39 62.665 44.700 44.948 1.00 0.00 H

ATOM 622 CB PHE 39 62.190 43.609 43.180 1.00 0.00 C

ATOM 623 HB2 PHE 39 62.460 43.790 42.140 1.00 0.00 H

ATOM 624 HB3 PHE 39 62.067 42.530 43.271 1.00 0.00 H

ATOM 625 CG PHE 39 60.743 44.225 43.221 1.00 0.00 C

ATOM 626 CD1 PHE 39 59.911 43.902 44.327 1.00 0.00 C

ATOM 627 HD1 PHE 39 60.119 43.063 44.974 1.00 0.00 H

ATOM 628 CE1 PHE 39 58.704 44.559 44.477 1.00 0.00 C

ATOM 629 HE1 PHE 39 58.009 44.181 45.212 1.00 0.00 H

ATOM 630 CZ PHE 39 58.395 45.618 43.623 1.00 0.00 C

ATOM 631 HZ PHE 39 57.448 46.125 43.739 1.00 0.00 H

ATOM 632 CE2 PHE 39 59.153 45.787 42.463 1.00 0.00 C

ATOM 633 HE2 PHE 39 58.749 46.354 41.637 1.00 0.00 H

ATOM 634 CD2 PHE 39 60.427 45.356 42.413 1.00 0.00 C

ATOM 635 HD2 PHE 39 60.982 45.703 41.554 1.00 0.00 H

ATOM 636 C PHE 39 63.870 42.886 44.977 1.00 0.00 C

ATOM 637 O PHE 39 63.441 42.471 46.082 1.00 0.00 O

ATOM 638 N VAL 40 64.908 42.246 44.349 1.00 0.00 N

ATOM 639 H VAL 40 65.319 42.694 43.541 1.00 0.00 H

ATOM 640 CA VAL 40 65.357 40.854 44.647 1.00 0.00 C

ATOM 641 HA VAL 40 64.661 40.369 45.330 1.00 0.00 H

ATOM 642 CB VAL 40 65.415 40.080 43.331 1.00 0.00 C

ATOM 643 HB VAL 40 65.311 39.044 43.652 1.00 0.00 H

ATOM 644 CG1 VAL 40 64.150 40.257 42.473 1.00 0.00 C

ATOM 645 HG11 VAL 40 63.290 40.217 43.142 1.00 0.00 H

ATOM 646 HG12 VAL 40 64.323 41.258 42.079 1.00 0.00 H

ATOM 647 HG13 VAL 40 64.056 39.557 41.643 1.00 0.00 H

ATOM 648 CG2 VAL 40 66.665 40.366 42.466 1.00 0.00 C

ATOM 649 HG21 VAL 40 66.459 40.369 41.395 1.00 0.00 H

ATOM 650 HG22 VAL 40 66.977 41.383 42.703 1.00 0.00 H

ATOM 651 HG23 VAL 40 67.460 39.651 42.679 1.00 0.00 H

ATOM 652 C VAL 40 66.730 40.786 45.369 1.00 0.00 C

ATOM 653 O VAL 40 67.013 39.702 45.881 1.00 0.00 O

ATOM 654 N PHE 41 67.522 41.904 45.520 1.00 0.00 N

ATOM 655 H PHE 41 67.241 42.722 44.998 1.00 0.00 H

ATOM 656 CA PHE 41 68.842 42.071 46.196 1.00 0.00 C

ATOM 657 HA PHE 41 69.094 41.144 46.711 1.00 0.00 H

ATOM 658 CB PHE 41 69.821 42.338 45.012 1.00 0.00 C

ATOM 659 HB2 PHE 41 69.567 43.227 44.434 1.00 0.00 H

ATOM 660 HB3 PHE 41 70.833 42.471 45.395 1.00 0.00 H

ATOM 661 CG PHE 41 70.074 41.117 44.081 1.00 0.00 C

ATOM 662 CD1 PHE 41 69.790 41.112 42.694 1.00 0.00 C

ATOM 663 HD1 PHE 41 69.493 41.997 42.151 1.00 0.00 H

ATOM 664 CE1 PHE 41 70.274 40.063 41.920 1.00 0.00 C

ATOM 665 HE1 PHE 41 70.160 40.010 40.847 1.00 0.00 H

ATOM 666 CZ PHE 41 70.929 38.914 42.457 1.00 0.00 C

ATOM 667 HZ PHE 41 71.073 37.995 41.907 1.00 0.00 H

ATOM 668 CE2 PHE 41 71.147 38.845 43.858 1.00 0.00 C

ATOM 669 HE2 PHE 41 71.900 38.114 44.113 1.00 0.00 H

ATOM 670 CD2 PHE 41 70.783 39.998 44.634 1.00 0.00 C

ATOM 671 HD2 PHE 41 71.032 39.902 45.681 1.00 0.00 H

ATOM 672 C PHE 41 68.707 43.150 47.265 1.00 0.00 C

ATOM 673 O PHE 41 69.209 42.912 48.384 1.00 0.00 O

ATOM 674 N HID 42 68.206 44.364 46.988 1.00 0.00 N

ATOM 675 H HID 42 67.892 44.558 46.048 1.00 0.00 H

ATOM 676 CA HID 42 68.424 45.553 47.826 1.00 0.00 C

ATOM 677 HA HID 42 69.341 45.386 48.392 1.00 0.00 H

ATOM 678 CB HID 42 68.911 46.658 46.878 1.00 0.00 C

ATOM 679 HB2 HID 42 68.109 46.854 46.166 1.00 0.00 H

ATOM 680 HB3 HID 42 69.181 47.586 47.383 1.00 0.00 H

ATOM 681 CG HID 42 70.118 46.165 46.076 1.00 0.00 C

ATOM 682 ND1 HID 42 71.282 45.855 46.660 1.00 0.00 N

ATOM 683 HD1 HID 42 71.548 46.071 47.610 1.00 0.00 H

ATOM 684 CE1 HID 42 72.120 45.442 45.675 1.00 0.00 C

ATOM 685 HE1 HID 42 73.133 45.176 45.939 1.00 0.00 H

ATOM 686 NE2 HID 42 71.562 45.536 44.436 1.00 0.00 N

ATOM 687 CD2 HID 42 70.297 46.085 44.748 1.00 0.00 C

ATOM 688 HD2 HID 42 69.507 46.132 44.013 1.00 0.00 H

ATOM 689 C HID 42 67.272 45.748 48.866 1.00 0.00 C

ATOM 690 O HID 42 67.534 46.306 49.892 1.00 0.00 O

ATOM 691 N MET 43 66.068 45.209 48.566 1.00 0.00 N

ATOM 692 H MET 43 66.045 44.563 47.789 1.00 0.00 H

ATOM 693 CA MET 43 64.829 45.429 49.265 1.00 0.00 C

ATOM 694 HA MET 43 65.067 46.303 49.871 1.00 0.00 H

ATOM 695 CB MET 43 63.804 45.735 48.145 1.00 0.00 C

ATOM 696 HB2 MET 43 64.101 46.564 47.501 1.00 0.00 H

ATOM 697 HB3 MET 43 63.825 44.806 47.576 1.00 0.00 H

ATOM 698 CG MET 43 62.405 45.912 48.584 1.00 0.00 C

ATOM 699 HG2 MET 43 62.325 45.505 49.591 1.00 0.00 H

ATOM 700 HG3 MET 43 62.096 46.957 48.575 1.00 0.00 H

ATOM 701 SD MET 43 61.325 44.893 47.442 1.00 0.00 S

ATOM 702 CE MET 43 59.681 45.293 48.040 1.00 0.00 C

ATOM 703 HE1 MET 43 59.509 46.369 48.014 1.00 0.00 H

ATOM 704 HE2 MET 43 58.952 44.833 47.372 1.00 0.00 H

ATOM 705 HE3 MET 43 59.561 44.881 49.042 1.00 0.00 H

ATOM 706 C MET 43 64.573 44.266 50.258 1.00 0.00 C

ATOM 707 O MET 43 64.241 43.205 49.822 1.00 0.00 O

ATOM 708 N LYS 44 64.725 44.569 51.566 1.00 0.00 N

ATOM 709 H LYS 44 64.951 45.527 51.793 1.00 0.00 H

ATOM 710 CA LYS 44 64.413 43.513 52.647 1.00 0.00 C

ATOM 711 HA LYS 44 63.869 42.694 52.176 1.00 0.00 H

ATOM 712 CB LYS 44 65.737 42.837 53.197 1.00 0.00 C

ATOM 713 HB2 LYS 44 65.748 43.103 54.254 1.00 0.00 H

ATOM 714 HB3 LYS 44 65.681 41.749 53.236 1.00 0.00 H

ATOM 715 CG LYS 44 67.055 43.272 52.576 1.00 0.00 C

ATOM 716 HG2 LYS 44 67.835 42.535 52.765 1.00 0.00 H

ATOM 717 HG3 LYS 44 66.962 43.181 51.493 1.00 0.00 H

ATOM 718 CD LYS 44 67.612 44.616 53.112 1.00 0.00 C

ATOM 719 HD2 LYS 44 66.895 45.437 53.084 1.00 0.00 H

ATOM 720 HD3 LYS 44 67.813 44.382 54.157 1.00 0.00 H

ATOM 721 CE LYS 44 68.903 44.930 52.496 1.00 0.00 C

ATOM 722 HE2 LYS 44 69.460 43.993 52.483 1.00 0.00 H

ATOM 723 HE3 LYS 44 68.644 45.321 51.512 1.00 0.00 H

ATOM 724 NZ LYS 44 69.543 46.015 53.319 1.00 0.00 N

ATOM 725 HZ1 LYS 44 69.510 45.844 54.313 1.00 0.00 H

ATOM 726 HZ2 LYS 44 70.509 46.103 53.038 1.00 0.00 H

ATOM 727 HZ3 LYS 44 69.098 46.909 53.169 1.00 0.00 H

ATOM 728 C LYS 44 63.651 44.141 53.829 1.00 0.00 C

ATOM 729 O LYS 44 63.902 45.297 54.178 1.00 0.00 O

ATOM 730 N PRO 45 62.644 43.409 54.410 1.00 0.00 N

ATOM 731 CD PRO 45 62.054 43.700 55.685 1.00 0.00 C

ATOM 732 HD2 PRO 45 61.292 44.462 55.518 1.00 0.00 H

ATOM 733 HD3 PRO 45 62.819 44.001 56.400 1.00 0.00 H

ATOM 734 CG PRO 45 61.402 42.434 56.248 1.00 0.00 C

ATOM 735 HG2 PRO 45 60.329 42.566 56.112 1.00 0.00 H

ATOM 736 HG3 PRO 45 61.665 42.216 57.283 1.00 0.00 H

ATOM 737 CB PRO 45 61.947 41.332 55.424 1.00 0.00 C

ATOM 738 HB2 PRO 45 61.277 40.491 55.242 1.00 0.00 H

ATOM 739 HB3 PRO 45 62.851 41.013 55.942 1.00 0.00 H

ATOM 740 CA PRO 45 62.455 42.045 54.124 1.00 0.00 C

ATOM 741 HA PRO 45 63.438 41.656 53.857 1.00 0.00 H

ATOM 742 C PRO 45 61.495 41.785 52.946 1.00 0.00 C

ATOM 743 O PRO 45 61.067 42.751 52.351 1.00 0.00 O

ATOM 744 N TRP 46 61.063 40.524 52.759 1.00 0.00 N

ATOM 745 H TRP 46 61.353 39.867 53.469 1.00 0.00 H

ATOM 746 CA TRP 46 60.269 40.115 51.633 1.00 0.00 C

ATOM 747 HA TRP 46 59.750 40.899 51.082 1.00 0.00 H

ATOM 748 CB TRP 46 61.107 39.328 50.552 1.00 0.00 C

ATOM 749 HB2 TRP 46 62.142 39.183 50.862 1.00 0.00 H

ATOM 750 HB3 TRP 46 60.798 38.283 50.524 1.00 0.00 H

ATOM 751 CG TRP 46 61.086 39.943 49.188 1.00 0.00 C

ATOM 752 CD1 TRP 46 61.465 41.178 48.943 1.00 0.00 C

ATOM 753 HD1 TRP 46 61.992 41.836 49.619 1.00 0.00 H

ATOM 754 NE1 TRP 46 61.499 41.286 47.584 1.00 0.00 N

ATOM 755 HE1 TRP 46 62.071 41.921 47.046 1.00 0.00 H

ATOM 756 CE2 TRP 46 60.769 40.332 46.934 1.00 0.00 C

ATOM 757 CZ2 TRP 46 60.445 40.128 45.618 1.00 0.00 C

ATOM 758 HZ2 TRP 46 60.832 40.771 44.841 1.00 0.00 H

ATOM 759 CH2 TRP 46 59.853 38.965 45.172 1.00 0.00 C

ATOM 760 HH2 TRP 46 59.552 38.843 44.142 1.00 0.00 H

ATOM 761 CZ3 TRP 46 59.430 38.083 46.197 1.00 0.00 C

ATOM 762 HZ3 TRP 46 58.866 37.192 45.966 1.00 0.00 H

ATOM 763 CE3 TRP 46 59.889 38.176 47.581 1.00 0.00 C

ATOM 764 HE3 TRP 46 59.777 37.282 48.176 1.00 0.00 H

ATOM 765 CD2 TRP 46 60.537 39.368 47.972 1.00 0.00 C

ATOM 766 C TRP 46 59.176 39.156 52.126 1.00 0.00 C

ATOM 767 O TRP 46 59.348 38.265 52.927 1.00 0.00 O

ATOM 768 N SER 47 57.924 39.483 51.790 1.00 0.00 N

ATOM 769 H SER 47 57.847 40.312 51.217 1.00 0.00 H

ATOM 770 CA SER 47 56.680 38.962 52.226 1.00 0.00 C

ATOM 771 HA SER 47 56.813 38.127 52.914 1.00 0.00 H

ATOM 772 CB SER 47 55.850 40.079 53.071 1.00 0.00 C

ATOM 773 HB2 SER 47 55.340 40.767 52.396 1.00 0.00 H

ATOM 774 HB3 SER 47 55.089 39.509 53.604 1.00 0.00 H

ATOM 775 OG SER 47 56.686 40.901 53.963 1.00 0.00 O

ATOM 776 HG SER 47 57.065 40.273 54.582 1.00 0.00 H

ATOM 777 C SER 47 55.748 38.407 51.145 1.00 0.00 C

ATOM 778 O SER 47 56.062 38.553 49.992 1.00 0.00 O

ATOM 779 N GLY 48 54.665 37.785 51.511 1.00 0.00 N

ATOM 780 H GLY 48 54.424 37.758 52.491 1.00 0.00 H

ATOM 781 CA GLY 48 53.585 37.309 50.660 1.00 0.00 C

ATOM 782 HA2 GLY 48 53.758 36.298 50.290 1.00 0.00 H

ATOM 783 HA3 GLY 48 52.661 37.254 51.235 1.00 0.00 H

ATOM 784 C GLY 48 53.321 38.317 49.533 1.00 0.00 C

ATOM 785 O GLY 48 53.388 37.960 48.362 1.00 0.00 O

ATOM 786 N ILE 49 53.071 39.592 49.875 1.00 0.00 N

ATOM 787 H ILE 49 52.955 39.741 50.867 1.00 0.00 H

ATOM 788 CA ILE 49 52.697 40.693 48.948 1.00 0.00 C

ATOM 789 HA ILE 49 51.943 40.183 48.348 1.00 0.00 H

ATOM 790 CB ILE 49 51.930 41.891 49.738 1.00 0.00 C

ATOM 791 HB ILE 49 51.211 41.359 50.361 1.00 0.00 H

ATOM 792 CG2 ILE 49 52.836 42.632 50.774 1.00 0.00 C

ATOM 793 HG21 ILE 49 53.390 43.450 50.315 1.00 0.00 H

ATOM 794 HG22 ILE 49 52.192 42.869 51.621 1.00 0.00 H

ATOM 795 HG23 ILE 49 53.516 41.932 51.260 1.00 0.00 H

ATOM 796 CG1 ILE 49 51.320 43.012 48.788 1.00 0.00 C

ATOM 797 HG12 ILE 49 51.138 43.883 49.417 1.00 0.00 H

ATOM 798 HG13 ILE 49 51.934 43.131 47.895 1.00 0.00 H

ATOM 799 CD1 ILE 49 49.940 42.558 48.267 1.00 0.00 C

ATOM 800 HD11 ILE 49 49.364 43.483 48.237 1.00 0.00 H

ATOM 801 HD12 ILE 49 50.044 42.068 47.299 1.00 0.00 H

ATOM 802 HD13 ILE 49 49.603 41.809 48.984 1.00 0.00 H

ATOM 803 C ILE 49 53.858 41.252 48.045 1.00 0.00 C

ATOM 804 O ILE 49 53.578 41.781 46.985 1.00 0.00 O

ATOM 805 N SER 50 55.121 41.007 48.401 1.00 0.00 N

ATOM 806 H SER 50 55.228 40.545 49.293 1.00 0.00 H

ATOM 807 CA SER 50 56.348 41.371 47.715 1.00 0.00 C

ATOM 808 HA SER 50 56.149 42.419 47.489 1.00 0.00 H

ATOM 809 CB SER 50 57.614 41.203 48.602 1.00 0.00 C

ATOM 810 HB2 SER 50 57.763 40.124 48.567 1.00 0.00 H

ATOM 811 HB3 SER 50 58.425 41.766 48.139 1.00 0.00 H

ATOM 812 OG SER 50 57.342 41.668 49.918 1.00 0.00 O

ATOM 813 HG SER 50 57.236 42.622 49.915 1.00 0.00 H

ATOM 814 C SER 50 56.367 40.587 46.439 1.00 0.00 C

ATOM 815 O SER 50 56.739 41.083 45.355 1.00 0.00 O

ATOM 816 N VAL 51 55.893 39.319 46.490 1.00 0.00 N

ATOM 817 H VAL 51 55.551 39.022 47.393 1.00 0.00 H

ATOM 818 CA VAL 51 55.943 38.431 45.365 1.00 0.00 C

ATOM 819 HA VAL 51 56.946 38.313 44.956 1.00 0.00 H

ATOM 820 CB VAL 51 55.474 36.982 45.803 1.00 0.00 C

ATOM 821 HB VAL 51 54.398 36.818 45.852 1.00 0.00 H

ATOM 822 CG1 VAL 51 55.894 35.989 44.686 1.00 0.00 C

ATOM 823 HG11 VAL 51 55.382 36.290 43.773 1.00 0.00 H

ATOM 824 HG12 VAL 51 56.971 35.937 44.526 1.00 0.00 H

ATOM 825 HG13 VAL 51 55.417 35.026 44.869 1.00 0.00 H

ATOM 826 CG2 VAL 51 56.093 36.411 47.072 1.00 0.00 C

ATOM 827 HG21 VAL 51 55.588 35.455 47.217 1.00 0.00 H

ATOM 828 HG22 VAL 51 57.168 36.278 46.951 1.00 0.00 H

ATOM 829 HG23 VAL 51 55.881 37.000 47.964 1.00 0.00 H

ATOM 830 C VAL 51 55.034 38.900 44.182 1.00 0.00 C

ATOM 831 O VAL 51 55.586 38.873 43.068 1.00 0.00 O

ATOM 832 N TYR 52 53.805 39.461 44.428 1.00 0.00 N

ATOM 833 H TYR 52 53.301 39.355 45.296 1.00 0.00 H

ATOM 834 CA TYR 52 52.912 39.923 43.494 1.00 0.00 C

ATOM 835 HA TYR 52 52.888 39.177 42.700 1.00 0.00 H

ATOM 836 CB TYR 52 51.468 39.980 44.022 1.00 0.00 C

ATOM 837 HB2 TYR 52 51.307 40.952 44.490 1.00 0.00 H

ATOM 838 HB3 TYR 52 50.772 40.039 43.186 1.00 0.00 H

ATOM 839 CG TYR 52 51.048 38.819 44.891 1.00 0.00 C

ATOM 840 CD1 TYR 52 51.037 37.510 44.325 1.00 0.00 C

ATOM 841 HD1 TYR 52 51.466 37.397 43.341 1.00 0.00 H

ATOM 842 CE1 TYR 52 50.243 36.470 44.960 1.00 0.00 C

ATOM 843 HE1 TYR 52 50.440 35.423 44.787 1.00 0.00 H

ATOM 844 CZ TYR 52 49.262 36.789 45.959 1.00 0.00 C

ATOM 845 OH TYR 52 48.328 35.898 46.425 1.00 0.00 O

ATOM 846 HH TYR 52 48.437 34.998 46.109 1.00 0.00 H

ATOM 847 CE2 TYR 52 49.294 38.086 46.507 1.00 0.00 C

ATOM 848 HE2 TYR 52 48.551 38.377 47.234 1.00 0.00 H

ATOM 849 CD2 TYR 52 50.158 39.068 45.983 1.00 0.00 C

ATOM 850 HD2 TYR 52 50.121 40.097 46.307 1.00 0.00 H

ATOM 851 C TYR 52 53.333 41.298 42.893 1.00 0.00 C

ATOM 852 O TYR 52 53.269 41.508 41.720 1.00 0.00 O

ATOM 853 N MET 53 53.708 42.240 43.707 1.00 0.00 N

ATOM 854 H MET 53 53.675 42.077 44.704 1.00 0.00 H

ATOM 855 CA MET 53 54.333 43.479 43.369 1.00 0.00 C

ATOM 856 HA MET 53 53.565 43.967 42.769 1.00 0.00 H

ATOM 857 CB MET 53 54.716 44.272 44.538 1.00 0.00 C

ATOM 858 HB2 MET 53 55.253 43.718 45.308 1.00 0.00 H

ATOM 859 HB3 MET 53 55.127 45.225 44.204 1.00 0.00 H

ATOM 860 CG MET 53 53.347 44.856 45.107 1.00 0.00 C

ATOM 861 HG2 MET 53 52.995 45.656 44.455 1.00 0.00 H

ATOM 862 HG3 MET 53 52.569 44.094 45.044 1.00 0.00 H

ATOM 863 SD MET 53 53.504 45.598 46.731 1.00 0.00 S

ATOM 864 CE MET 53 54.530 46.924 46.402 1.00 0.00 C

ATOM 865 HE1 MET 53 54.744 47.635 47.200 1.00 0.00 H

ATOM 866 HE2 MET 53 55.552 46.601 46.205 1.00 0.00 H

ATOM 867 HE3 MET 53 54.159 47.373 45.481 1.00 0.00 H

ATOM 868 C MET 53 55.564 43.325 42.465 1.00 0.00 C

ATOM 869 O MET 53 55.695 43.982 41.446 1.00 0.00 O

ATOM 870 N PHE 54 56.428 42.354 42.781 1.00 0.00 N

ATOM 871 H PHE 54 56.081 41.772 43.529 1.00 0.00 H

ATOM 872 CA PHE 54 57.493 41.961 41.919 1.00 0.00 C

ATOM 873 HA PHE 54 57.963 42.871 41.547 1.00 0.00 H

ATOM 874 CB PHE 54 58.498 41.045 42.656 1.00 0.00 C

ATOM 875 HB2 PHE 54 59.021 41.548 43.469 1.00 0.00 H

ATOM 876 HB3 PHE 54 57.917 40.289 43.185 1.00 0.00 H

ATOM 877 CG PHE 54 59.571 40.449 41.761 1.00 0.00 C

ATOM 878 CD1 PHE 54 60.421 41.251 40.994 1.00 0.00 C

ATOM 879 HD1 PHE 54 60.327 42.303 41.218 1.00 0.00 H

ATOM 880 CE1 PHE 54 61.344 40.707 40.151 1.00 0.00 C

ATOM 881 HE1 PHE 54 62.074 41.397 39.752 1.00 0.00 H

ATOM 882 CZ PHE 54 61.514 39.325 40.042 1.00 0.00 C

ATOM 883 HZ PHE 54 62.379 38.837 39.617 1.00 0.00 H

ATOM 884 CE2 PHE 54 60.484 38.527 40.589 1.00 0.00 C

ATOM 885 HE2 PHE 54 60.553 37.458 40.453 1.00 0.00 H

ATOM 886 CD2 PHE 54 59.573 39.114 41.466 1.00 0.00 C

ATOM 887 HD2 PHE 54 58.787 38.530 41.922 1.00 0.00 H

ATOM 888 C PHE 54 57.100 41.204 40.705 1.00 0.00 C

ATOM 889 O PHE 54 57.592 41.607 39.565 1.00 0.00 O

ATOM 890 N ASN 55 56.130 40.324 40.809 1.00 0.00 N

ATOM 891 H ASN 55 55.622 40.263 41.680 1.00 0.00 H

ATOM 892 CA ASN 55 55.642 39.513 39.701 1.00 0.00 C

ATOM 893 HA ASN 55 56.533 39.226 39.142 1.00 0.00 H

ATOM 894 CB ASN 55 54.877 38.231 40.036 1.00 0.00 C

ATOM 895 HB2 ASN 55 54.335 38.326 40.976 1.00 0.00 H

ATOM 896 HB3 ASN 55 54.292 37.940 39.163 1.00 0.00 H

ATOM 897 CG ASN 55 55.741 37.010 40.133 1.00 0.00 C

ATOM 898 OD1 ASN 55 55.791 36.215 39.207 1.00 0.00 O

ATOM 899 ND2 ASN 55 56.449 36.966 41.265 1.00 0.00 N

ATOM 900 HD21 ASN 55 56.210 37.614 42.002 1.00 0.00 H

ATOM 901 HD22 ASN 55 56.981 36.150 41.530 1.00 0.00 H

ATOM 902 C ASN 55 54.996 40.402 38.672 1.00 0.00 C

ATOM 903 O ASN 55 55.235 40.231 37.452 1.00 0.00 O

ATOM 904 N LEU 56 54.218 41.363 39.102 1.00 0.00 N

ATOM 905 H LEU 56 53.850 41.340 40.042 1.00 0.00 H

ATOM 906 CA LEU 56 53.827 42.432 38.146 1.00 0.00 C

ATOM 907 HA LEU 56 53.198 41.974 37.384 1.00 0.00 H

ATOM 908 CB LEU 56 53.012 43.471 39.009 1.00 0.00 C

ATOM 909 HB2 LEU 56 52.389 43.011 39.776 1.00 0.00 H

ATOM 910 HB3 LEU 56 53.662 44.179 39.522 1.00 0.00 H

ATOM 911 CG LEU 56 51.990 44.422 38.332 1.00 0.00 C

ATOM 912 HG LEU 56 52.455 45.216 37.749 1.00 0.00 H

ATOM 913 CD1 LEU 56 51.180 43.741 37.201 1.00 0.00 C

ATOM 914 HD11 LEU 56 50.839 42.766 37.550 1.00 0.00 H

ATOM 915 HD12 LEU 56 50.372 44.424 36.937 1.00 0.00 H

ATOM 916 HD13 LEU 56 51.867 43.491 36.392 1.00 0.00 H

ATOM 917 CD2 LEU 56 51.011 45.113 39.250 1.00 0.00 C

ATOM 918 HD21 LEU 56 50.901 46.147 38.922 1.00 0.00 H

ATOM 919 HD22 LEU 56 49.992 44.749 39.118 1.00 0.00 H

ATOM 920 HD23 LEU 56 51.220 45.100 40.320 1.00 0.00 H

ATOM 921 C LEU 56 55.072 43.226 37.541 1.00 0.00 C

ATOM 922 O LEU 56 55.009 43.454 36.345 1.00 0.00 O

ATOM 923 N ALA 57 55.991 43.740 38.391 1.00 0.00 N

ATOM 924 H ALA 57 55.887 43.789 39.394 1.00 0.00 H

ATOM 925 CA ALA 57 57.172 44.434 37.779 1.00 0.00 C

ATOM 926 HA ALA 57 56.840 45.408 37.419 1.00 0.00 H

ATOM 927 CB ALA 57 58.248 44.779 38.787 1.00 0.00 C

ATOM 928 HB1 ALA 57 58.912 45.544 38.385 1.00 0.00 H

ATOM 929 HB2 ALA 57 57.784 45.042 39.738 1.00 0.00 H

ATOM 930 HB3 ALA 57 58.874 43.920 39.028 1.00 0.00 H

ATOM 931 C ALA 57 57.672 43.756 36.505 1.00 0.00 C

ATOM 932 O ALA 57 57.681 44.294 35.434 1.00 0.00 O

ATOM 933 N LEU 58 57.963 42.409 36.643 1.00 0.00 N

ATOM 934 H LEU 58 57.725 42.082 37.569 1.00 0.00 H

ATOM 935 CA LEU 58 58.611 41.515 35.740 1.00 0.00 C

ATOM 936 HA LEU 58 59.530 41.945 35.341 1.00 0.00 H

ATOM 937 CB LEU 58 59.001 40.330 36.565 1.00 0.00 C

ATOM 938 HB2 LEU 58 59.335 40.628 37.559 1.00 0.00 H

ATOM 939 HB3 LEU 58 58.080 39.767 36.709 1.00 0.00 H

ATOM 940 CG LEU 58 60.076 39.384 35.888 1.00 0.00 C

ATOM 941 HG LEU 58 59.641 39.256 34.897 1.00 0.00 H

ATOM 942 CD1 LEU 58 61.505 39.880 35.785 1.00 0.00 C

ATOM 943 HD11 LEU 58 62.179 39.141 35.352 1.00 0.00 H

ATOM 944 HD12 LEU 58 61.552 40.797 35.197 1.00 0.00 H

ATOM 945 HD13 LEU 58 62.063 40.051 36.705 1.00 0.00 H

ATOM 946 CD2 LEU 58 59.967 38.050 36.610 1.00 0.00 C

ATOM 947 HD21 LEU 58 60.801 37.405 36.335 1.00 0.00 H

ATOM 948 HD22 LEU 58 60.033 38.237 37.682 1.00 0.00 H

ATOM 949 HD23 LEU 58 59.005 37.623 36.324 1.00 0.00 H

ATOM 950 C LEU 58 57.667 41.213 34.489 1.00 0.00 C

ATOM 951 O LEU 58 58.198 41.094 33.373 1.00 0.00 O

ATOM 952 N ALA 59 56.362 41.268 34.750 1.00 0.00 N

ATOM 953 H ALA 59 56.086 41.447 35.705 1.00 0.00 H

ATOM 954 CA ALA 59 55.317 41.164 33.727 1.00 0.00 C

ATOM 955 HA ALA 59 55.596 40.249 33.204 1.00 0.00 H

ATOM 956 CB ALA 59 53.938 40.985 34.439 1.00 0.00 C

ATOM 957 HB1 ALA 59 53.198 41.486 33.815 1.00 0.00 H

ATOM 958 HB2 ALA 59 53.706 39.922 34.498 1.00 0.00 H

ATOM 959 HB3 ALA 59 53.916 41.345 35.467 1.00 0.00 H

ATOM 960 C ALA 59 55.393 42.313 32.746 1.00 0.00 C

ATOM 961 O ALA 59 55.264 42.080 31.515 1.00 0.00 O

ATOM 962 N ASP 60 55.290 43.553 33.194 1.00 0.00 N

ATOM 963 H ASP 60 55.084 43.632 34.179 1.00 0.00 H

ATOM 964 CA ASP 60 55.456 44.769 32.355 1.00 0.00 C

ATOM 965 HA ASP 60 54.793 44.798 31.491 1.00 0.00 H

ATOM 966 CB ASP 60 55.159 46.004 33.276 1.00 0.00 C

ATOM 967 HB2 ASP 60 54.543 45.661 34.107 1.00 0.00 H

ATOM 968 HB3 ASP 60 56.081 46.357 33.739 1.00 0.00 H

ATOM 969 CG ASP 60 54.535 47.177 32.548 1.00 0.00 C

ATOM 970 OD1 ASP 60 55.125 48.333 32.592 1.00 0.00 O

ATOM 971 OD2 ASP 60 53.546 46.908 31.796 1.00 0.00 O

ATOM 972 C ASP 60 56.850 44.959 31.759 1.00 0.00 C

ATOM 973 O ASP 60 56.940 45.533 30.628 1.00 0.00 O

ATOM 974 N PHE 61 57.894 44.377 32.330 1.00 0.00 N

ATOM 975 H PHE 61 57.748 44.075 33.283 1.00 0.00 H

ATOM 976 CA PHE 61 59.167 44.115 31.720 1.00 0.00 C

ATOM 977 HA PHE 61 59.606 45.010 31.279 1.00 0.00 H

ATOM 978 CB PHE 61 60.240 43.665 32.800 1.00 0.00 C

ATOM 979 HB2 PHE 61 59.936 43.834 33.833 1.00 0.00 H

ATOM 980 HB3 PHE 61 60.313 42.579 32.734 1.00 0.00 H

ATOM 981 CG PHE 61 61.619 44.132 32.461 1.00 0.00 C

ATOM 982 CD1 PHE 61 61.894 45.493 32.727 1.00 0.00 C

ATOM 983 HD1 PHE 61 61.436 45.992 33.568 1.00 0.00 H

ATOM 984 CE1 PHE 61 62.997 46.103 32.089 1.00 0.00 C

ATOM 985 HE1 PHE 61 63.227 47.152 32.201 1.00 0.00 H

ATOM 986 CZ PHE 61 63.781 45.465 31.098 1.00 0.00 C

ATOM 987 HZ PHE 61 64.611 46.043 30.720 1.00 0.00 H

ATOM 988 CE2 PHE 61 63.340 44.198 30.664 1.00 0.00 C

ATOM 989 HE2 PHE 61 63.839 43.748 29.819 1.00 0.00 H

ATOM 990 CD2 PHE 61 62.391 43.529 31.373 1.00 0.00 C

ATOM 991 HD2 PHE 61 61.995 42.594 31.005 1.00 0.00 H

ATOM 992 C PHE 61 59.064 43.069 30.589 1.00 0.00 C

ATOM 993 O PHE 61 59.732 43.277 29.564 1.00 0.00 O

ATOM 994 N LEU 62 58.187 42.109 30.676 1.00 0.00 N

ATOM 995 H LEU 62 57.854 41.745 31.557 1.00 0.00 H

ATOM 996 CA LEU 62 57.949 41.185 29.511 1.00 0.00 C

ATOM 997 HA LEU 62 58.901 40.840 29.106 1.00 0.00 H

ATOM 998 CB LEU 62 57.202 39.919 29.797 1.00 0.00 C

ATOM 999 HB2 LEU 62 57.697 39.426 30.634 1.00 0.00 H

ATOM 1000 HB3 LEU 62 56.225 40.271 30.129 1.00 0.00 H

ATOM 1001 CG LEU 62 56.960 38.973 28.624 1.00 0.00 C

ATOM 1002 HG LEU 62 56.453 39.600 27.890 1.00 0.00 H

ATOM 1003 CD1 LEU 62 58.316 38.370 28.151 1.00 0.00 C

ATOM 1004 HD11 LEU 62 58.181 37.550 27.446 1.00 0.00 H

ATOM 1005 HD12 LEU 62 58.752 39.245 27.669 1.00 0.00 H

ATOM 1006 HD13 LEU 62 58.915 37.897 28.929 1.00 0.00 H

ATOM 1007 CD2 LEU 62 56.063 37.848 29.071 1.00 0.00 C

ATOM 1008 HD21 LEU 62 55.916 37.239 28.179 1.00 0.00 H

ATOM 1009 HD22 LEU 62 56.588 37.270 29.831 1.00 0.00 H

ATOM 1010 HD23 LEU 62 55.029 38.113 29.290 1.00 0.00 H

ATOM 1011 C LEU 62 57.438 42.022 28.322 1.00 0.00 C

ATOM 1012 O LEU 62 57.913 41.768 27.191 1.00 0.00 O

ATOM 1013 N TYR 63 56.463 42.969 28.525 1.00 0.00 N

ATOM 1014 H TYR 63 56.139 42.986 29.481 1.00 0.00 H

ATOM 1015 CA TYR 63 56.016 43.870 27.477 1.00 0.00 C

ATOM 1016 HA TYR 63 55.689 43.272 26.627 1.00 0.00 H

ATOM 1017 CB TYR 63 54.874 44.827 27.976 1.00 0.00 C

ATOM 1018 HB2 TYR 63 54.061 44.137 28.203 1.00 0.00 H

ATOM 1019 HB3 TYR 63 55.167 45.304 28.912 1.00 0.00 H

ATOM 1020 CG TYR 63 54.306 45.944 27.038 1.00 0.00 C

ATOM 1021 CD1 TYR 63 54.215 47.282 27.474 1.00 0.00 C

ATOM 1022 HD1 TYR 63 54.812 47.661 28.290 1.00 0.00 H

ATOM 1023 CE1 TYR 63 53.618 48.281 26.690 1.00 0.00 C

ATOM 1024 HE1 TYR 63 53.635 49.345 26.875 1.00 0.00 H

ATOM 1025 CZ TYR 63 53.030 47.936 25.391 1.00 0.00 C

ATOM 1026 OH TYR 63 52.370 48.904 24.720 1.00 0.00 O

ATOM 1027 HH TYR 63 51.884 48.582 23.957 1.00 0.00 H

ATOM 1028 CE2 TYR 63 53.021 46.558 24.990 1.00 0.00 C

ATOM 1029 HE2 TYR 63 52.659 46.249 24.021 1.00 0.00 H

ATOM 1030 CD2 TYR 63 53.704 45.591 25.794 1.00 0.00 C

ATOM 1031 HD2 TYR 63 53.803 44.572 25.451 1.00 0.00 H

ATOM 1032 C TYR 63 57.193 44.751 26.923 1.00 0.00 C

ATOM 1033 O TYR 63 57.287 45.084 25.682 1.00 0.00 O

ATOM 1034 N VAL 64 58.102 45.160 27.772 1.00 0.00 N

ATOM 1035 H VAL 64 58.284 44.660 28.631 1.00 0.00 H

ATOM 1036 CA VAL 64 59.263 46.037 27.309 1.00 0.00 C

ATOM 1037 HA VAL 64 58.858 46.730 26.572 1.00 0.00 H

ATOM 1038 CB VAL 64 59.815 46.945 28.510 1.00 0.00 C

ATOM 1039 HB VAL 64 59.316 46.570 29.404 1.00 0.00 H

ATOM 1040 CG1 VAL 64 61.285 47.114 28.738 1.00 0.00 C

ATOM 1041 HG11 VAL 64 61.845 47.295 27.820 1.00 0.00 H

ATOM 1042 HG12 VAL 64 61.448 47.849 29.526 1.00 0.00 H

ATOM 1043 HG13 VAL 64 61.658 46.140 29.054 1.00 0.00 H

ATOM 1044 CG2 VAL 64 59.284 48.411 28.289 1.00 0.00 C

ATOM 1045 HG21 VAL 64 58.198 48.330 28.243 1.00 0.00 H

ATOM 1046 HG22 VAL 64 59.614 49.000 29.144 1.00 0.00 H

ATOM 1047 HG23 VAL 64 59.638 48.778 27.326 1.00 0.00 H

ATOM 1048 C VAL 64 60.323 45.244 26.558 1.00 0.00 C

ATOM 1049 O VAL 64 60.997 45.799 25.762 1.00 0.00 O

ATOM 1050 N LEU 65 60.367 43.944 26.730 1.00 0.00 N

ATOM 1051 H LEU 65 60.150 43.662 27.675 1.00 0.00 H

ATOM 1052 CA LEU 65 61.206 43.113 25.855 1.00 0.00 C

ATOM 1053 HA LEU 65 62.141 43.635 25.651 1.00 0.00 H

ATOM 1054 CB LEU 65 61.518 41.740 26.559 1.00 0.00 C

ATOM 1055 HB2 LEU 65 60.652 41.320 27.072 1.00 0.00 H

ATOM 1056 HB3 LEU 65 61.803 40.983 25.828 1.00 0.00 H

ATOM 1057 CG LEU 65 62.581 42.034 27.650 1.00 0.00 C

ATOM 1058 HG LEU 65 62.291 42.837 28.328 1.00 0.00 H

ATOM 1059 CD1 LEU 65 62.668 40.748 28.554 1.00 0.00 C

ATOM 1060 HD11 LEU 65 63.571 40.764 29.164 1.00 0.00 H

ATOM 1061 HD12 LEU 65 61.748 40.799 29.136 1.00 0.00 H

ATOM 1062 HD13 LEU 65 62.552 39.812 28.008 1.00 0.00 H

ATOM 1063 CD2 LEU 65 63.938 42.313 27.095 1.00 0.00 C

ATOM 1064 HD21 LEU 65 64.373 41.480 26.543 1.00 0.00 H

ATOM 1065 HD22 LEU 65 63.910 43.117 26.359 1.00 0.00 H

ATOM 1066 HD23 LEU 65 64.608 42.512 27.932 1.00 0.00 H

ATOM 1067 C LEU 65 60.670 42.889 24.444 1.00 0.00 C

ATOM 1068 O LEU 65 61.409 42.467 23.546 1.00 0.00 O

ATOM 1069 N THR 66 59.321 42.917 24.278 1.00 0.00 N

ATOM 1070 H THR 66 58.755 42.963 25.113 1.00 0.00 H

ATOM 1071 CA THR 66 58.544 42.721 23.075 1.00 0.00 C

ATOM 1072 HA THR 66 59.041 41.935 22.507 1.00 0.00 H

ATOM 1073 CB THR 66 57.092 42.357 23.404 1.00 0.00 C

ATOM 1074 HB THR 66 56.564 43.286 23.619 1.00 0.00 H

ATOM 1075 CG2 THR 66 56.489 41.633 22.195 1.00 0.00 C

ATOM 1076 HG21 THR 66 56.564 42.216 21.277 1.00 0.00 H

ATOM 1077 HG22 THR 66 56.822 40.597 22.252 1.00 0.00 H

ATOM 1078 HG23 THR 66 55.409 41.620 22.341 1.00 0.00 H

ATOM 1079 OG1 THR 66 56.933 41.467 24.471 1.00 0.00 O

ATOM 1080 HG1 THR 66 57.413 41.656 25.280 1.00 0.00 H

ATOM 1081 C THR 66 58.456 44.048 22.245 1.00 0.00 C

ATOM 1082 O THR 66 58.617 44.055 21.020 1.00 0.00 O

ATOM 1083 N LEU 67 58.226 45.220 22.889 1.00 0.00 N

ATOM 1084 H LEU 67 58.215 45.064 23.887 1.00 0.00 H

ATOM 1085 CA LEU 67 58.057 46.523 22.278 1.00 0.00 C

ATOM 1086 HA LEU 67 57.098 46.580 21.762 1.00 0.00 H

ATOM 1087 CB LEU 67 58.124 47.628 23.392 1.00 0.00 C

ATOM 1088 HB2 LEU 67 58.742 47.291 24.224 1.00 0.00 H

ATOM 1089 HB3 LEU 67 58.562 48.546 23.000 1.00 0.00 H

ATOM 1090 CG LEU 67 56.771 48.155 23.954 1.00 0.00 C

ATOM 1091 HG LEU 67 56.328 47.294 24.454 1.00 0.00 H

ATOM 1092 CD1 LEU 67 56.979 49.281 24.956 1.00 0.00 C

ATOM 1093 HD11 LEU 67 57.970 49.115 25.381 1.00 0.00 H

ATOM 1094 HD12 LEU 67 57.007 50.227 24.417 1.00 0.00 H

ATOM 1095 HD13 LEU 67 56.130 49.368 25.634 1.00 0.00 H

ATOM 1096 CD2 LEU 67 55.690 48.563 22.971 1.00 0.00 C

ATOM 1097 HD21 LEU 67 55.025 47.732 22.734 1.00 0.00 H

ATOM 1098 HD22 LEU 67 55.104 49.322 23.488 1.00 0.00 H

ATOM 1099 HD23 LEU 67 56.199 49.061 22.145 1.00 0.00 H

ATOM 1100 C LEU 67 59.122 46.839 21.163 1.00 0.00 C

ATOM 1101 O LEU 67 58.793 47.376 20.114 1.00 0.00 O

ATOM 1102 N PRO 68 60.484 46.465 21.387 1.00 0.00 N

ATOM 1103 CD PRO 68 61.215 46.116 22.616 1.00 0.00 C

ATOM 1104 HD2 PRO 68 60.757 45.286 23.154 1.00 0.00 H

ATOM 1105 HD3 PRO 68 61.126 47.037 23.191 1.00 0.00 H

ATOM 1106 CG PRO 68 62.600 45.664 22.166 1.00 0.00 C

ATOM 1107 HG2 PRO 68 62.546 44.580 22.063 1.00 0.00 H

ATOM 1108 HG3 PRO 68 63.358 45.985 22.880 1.00 0.00 H

ATOM 1109 CB PRO 68 62.919 46.372 20.843 1.00 0.00 C

ATOM 1110 HB2 PRO 68 63.386 45.628 20.197 1.00 0.00 H

ATOM 1111 HB3 PRO 68 63.558 47.223 21.080 1.00 0.00 H

ATOM 1112 CA PRO 68 61.549 46.751 20.392 1.00 0.00 C

ATOM 1113 HA PRO 68 61.495 47.812 20.149 1.00 0.00 H

ATOM 1114 C PRO 68 61.156 46.073 19.076 1.00 0.00 C

ATOM 1115 O PRO 68 61.099 46.794 18.083 1.00 0.00 O

ATOM 1116 N ALA 69 60.755 44.813 19.109 1.00 0.00 N

ATOM 1117 H ALA 69 60.595 44.430 20.029 1.00 0.00 H

ATOM 1118 CA ALA 69 60.305 44.050 17.922 1.00 0.00 C

ATOM 1119 HA ALA 69 61.196 44.194 17.310 1.00 0.00 H

ATOM 1120 CB ALA 69 60.110 42.579 18.202 1.00 0.00 C

ATOM 1121 HB1 ALA 69 59.267 42.377 18.862 1.00 0.00 H

ATOM 1122 HB2 ALA 69 60.076 42.122 17.213 1.00 0.00 H

ATOM 1123 HB3 ALA 69 61.096 42.209 18.482 1.00 0.00 H

ATOM 1124 C ALA 69 59.092 44.630 17.244 1.00 0.00 C

ATOM 1125 O ALA 69 58.952 44.726 16.013 1.00 0.00 O

ATOM 1126 N LEU 70 58.124 44.944 18.103 1.00 0.00 N

ATOM 1127 H LEU 70 58.506 44.939 19.038 1.00 0.00 H

ATOM 1128 CA LEU 70 56.898 45.703 17.698 1.00 0.00 C

ATOM 1129 HA LEU 70 56.331 45.115 16.976 1.00 0.00 H

ATOM 1130 CB LEU 70 55.939 45.524 18.924 1.00 0.00 C

ATOM 1131 HB2 LEU 70 56.189 44.609 19.460 1.00 0.00 H

ATOM 1132 HB3 LEU 70 56.195 46.315 19.629 1.00 0.00 H

ATOM 1133 CG LEU 70 54.464 45.660 18.596 1.00 0.00 C

ATOM 1134 HG LEU 70 54.392 46.483 17.885 1.00 0.00 H

ATOM 1135 CD1 LEU 70 53.969 44.326 18.000 1.00 0.00 C

ATOM 1136 HD11 LEU 70 52.895 44.180 18.110 1.00 0.00 H

ATOM 1137 HD12 LEU 70 54.151 44.301 16.925 1.00 0.00 H

ATOM 1138 HD13 LEU 70 54.422 43.435 18.434 1.00 0.00 H

ATOM 1139 CD2 LEU 70 53.731 45.944 19.931 1.00 0.00 C

ATOM 1140 HD21 LEU 70 53.960 45.157 20.650 1.00 0.00 H

ATOM 1141 HD22 LEU 70 54.113 46.899 20.290 1.00 0.00 H

ATOM 1142 HD23 LEU 70 52.647 45.945 19.817 1.00 0.00 H

ATOM 1143 C LEU 70 57.090 47.068 17.008 1.00 0.00 C

ATOM 1144 O LEU 70 56.564 47.280 15.920 1.00 0.00 O

ATOM 1145 N ILE 71 58.054 47.819 17.509 1.00 0.00 N

ATOM 1146 H ILE 71 58.433 47.513 18.394 1.00 0.00 H

ATOM 1147 CA ILE 71 58.473 49.072 16.856 1.00 0.00 C

ATOM 1148 HA ILE 71 57.604 49.677 16.593 1.00 0.00 H

ATOM 1149 CB ILE 71 59.410 49.959 17.762 1.00 0.00 C

ATOM 1150 HB ILE 71 60.238 49.365 18.149 1.00 0.00 H

ATOM 1151 CG2 ILE 71 59.887 51.130 16.923 1.00 0.00 C

ATOM 1152 HG21 ILE 71 60.473 51.889 17.442 1.00 0.00 H

ATOM 1153 HG22 ILE 71 60.568 50.871 16.112 1.00 0.00 H

ATOM 1154 HG23 ILE 71 58.960 51.585 16.576 1.00 0.00 H

ATOM 1155 CG1 ILE 71 58.569 50.460 18.936 1.00 0.00 C

ATOM 1156 HG12 ILE 71 57.804 51.141 18.562 1.00 0.00 H

ATOM 1157 HG13 ILE 71 58.027 49.670 19.456 1.00 0.00 H

ATOM 1158 CD1 ILE 71 59.352 51.280 19.973 1.00 0.00 C

ATOM 1159 HD11 ILE 71 60.252 50.696 20.170 1.00 0.00 H

ATOM 1160 HD12 ILE 71 59.602 52.260 19.566 1.00 0.00 H

ATOM 1161 HD13 ILE 71 58.767 51.312 20.892 1.00 0.00 H

ATOM 1162 C ILE 71 59.160 48.744 15.502 1.00 0.00 C

ATOM 1163 O ILE 71 58.932 49.330 14.485 1.00 0.00 O

ATOM 1164 N PHE 72 60.163 47.868 15.543 1.00 0.00 N

ATOM 1165 H PHE 72 60.481 47.564 16.452 1.00 0.00 H

ATOM 1166 CA PHE 72 60.775 47.248 14.345 1.00 0.00 C

ATOM 1167 HA PHE 72 61.369 48.047 13.902 1.00 0.00 H

ATOM 1168 CB PHE 72 61.882 46.221 14.788 1.00 0.00 C

ATOM 1169 HB2 PHE 72 62.235 46.450 15.794 1.00 0.00 H

ATOM 1170 HB3 PHE 72 61.332 45.283 14.866 1.00 0.00 H

ATOM 1171 CG PHE 72 63.009 46.133 13.680 1.00 0.00 C

ATOM 1172 CD1 PHE 72 62.668 45.558 12.504 1.00 0.00 C

ATOM 1173 HD1 PHE 72 61.744 45.006 12.419 1.00 0.00 H

ATOM 1174 CE1 PHE 72 63.556 45.637 11.370 1.00 0.00 C

ATOM 1175 HE1 PHE 72 63.229 45.341 10.384 1.00 0.00 H

ATOM 1176 CZ PHE 72 64.814 46.050 11.561 1.00 0.00 C

ATOM 1177 HZ PHE 72 65.317 46.448 10.692 1.00 0.00 H

ATOM 1178 CE2 PHE 72 65.126 46.675 12.800 1.00 0.00 C

ATOM 1179 HE2 PHE 72 66.048 47.233 12.871 1.00 0.00 H

ATOM 1180 CD2 PHE 72 64.249 46.658 13.894 1.00 0.00 C

ATOM 1181 HD2 PHE 72 64.716 46.845 14.850 1.00 0.00 H

ATOM 1182 C PHE 72 59.824 46.711 13.351 1.00 0.00 C

ATOM 1183 O PHE 72 60.058 46.873 12.142 1.00 0.00 O

ATOM 1184 N TYR 73 58.711 46.036 13.735 1.00 0.00 N

ATOM 1185 H TYR 73 58.665 45.839 14.725 1.00 0.00 H

ATOM 1186 CA TYR 73 57.637 45.621 12.832 1.00 0.00 C

ATOM 1187 HA TYR 73 57.972 45.115 11.926 1.00 0.00 H

ATOM 1188 CB TYR 73 56.657 44.693 13.609 1.00 0.00 C

ATOM 1189 HB2 TYR 73 57.140 43.727 13.755 1.00 0.00 H

ATOM 1190 HB3 TYR 73 56.433 45.102 14.594 1.00 0.00 H

ATOM 1191 CG TYR 73 55.321 44.554 13.004 1.00 0.00 C

ATOM 1192 CD1 TYR 73 55.206 43.771 11.873 1.00 0.00 C

ATOM 1193 HD1 TYR 73 56.076 43.365 11.378 1.00 0.00 H

ATOM 1194 CE1 TYR 73 53.918 43.170 11.597 1.00 0.00 C

ATOM 1195 HE1 TYR 73 53.745 42.461 10.801 1.00 0.00 H

ATOM 1196 CZ TYR 73 52.792 43.830 12.078 1.00 0.00 C

ATOM 1197 OH TYR 73 51.520 43.368 11.872 1.00 0.00 O

ATOM 1198 HH TYR 73 51.503 42.512 11.436 1.00 0.00 H

ATOM 1199 CE2 TYR 73 52.946 44.916 13.011 1.00 0.00 C

ATOM 1200 HE2 TYR 73 52.062 45.482 13.266 1.00 0.00 H

ATOM 1201 CD2 TYR 73 54.198 45.180 13.563 1.00 0.00 C

ATOM 1202 HD2 TYR 73 54.323 45.864 14.389 1.00 0.00 H

ATOM 1203 C TYR 73 56.870 46.800 12.166 1.00 0.00 C

ATOM 1204 O TYR 73 56.758 46.877 10.952 1.00 0.00 O

ATOM 1205 N TYR 74 56.259 47.690 12.974 1.00 0.00 N

ATOM 1206 H TYR 74 56.373 47.622 13.975 1.00 0.00 H

ATOM 1207 CA TYR 74 55.462 48.842 12.592 1.00 0.00 C

ATOM 1208 HA TYR 74 54.696 48.483 11.904 1.00 0.00 H

ATOM 1209 CB TYR 74 54.643 49.434 13.759 1.00 0.00 C

ATOM 1210 HB2 TYR 74 55.238 49.531 14.667 1.00 0.00 H

ATOM 1211 HB3 TYR 74 54.251 50.411 13.478 1.00 0.00 H

ATOM 1212 CG TYR 74 53.390 48.688 14.193 1.00 0.00 C

ATOM 1213 CD1 TYR 74 53.260 48.082 15.439 1.00 0.00 C

ATOM 1214 HD1 TYR 74 53.970 48.354 16.205 1.00 0.00 H

ATOM 1215 CE1 TYR 74 52.080 47.347 15.746 1.00 0.00 C

ATOM 1216 HE1 TYR 74 51.916 46.855 16.693 1.00 0.00 H

ATOM 1217 CZ TYR 74 51.037 47.264 14.774 1.00 0.00 C

ATOM 1218 OH TYR 74 49.886 46.643 15.091 1.00 0.00 O

ATOM 1219 HH TYR 74 50.118 45.902 15.656 1.00 0.00 H

ATOM 1220 CE2 TYR 74 51.109 48.113 13.660 1.00 0.00 C

ATOM 1221 HE2 TYR 74 50.208 48.189 13.068 1.00 0.00 H

ATOM 1222 CD2 TYR 74 52.282 48.740 13.319 1.00 0.00 C

ATOM 1223 HD2 TYR 74 52.371 49.217 12.354 1.00 0.00 H

ATOM 1224 C TYR 74 56.226 49.859 11.731 1.00 0.00 C

ATOM 1225 O TYR 74 55.663 50.320 10.721 1.00 0.00 O

ATOM 1226 N PHE 75 57.540 49.964 11.994 1.00 0.00 N

ATOM 1227 H PHE 75 57.882 49.501 12.825 1.00 0.00 H

ATOM 1228 CA PHE 75 58.364 50.948 11.316 1.00 0.00 C

ATOM 1229 HA PHE 75 57.608 51.729 11.243 1.00 0.00 H

ATOM 1230 CB PHE 75 59.634 51.410 12.146 1.00 0.00 C

ATOM 1231 HB2 PHE 75 59.624 51.332 13.233 1.00 0.00 H

ATOM 1232 HB3 PHE 75 60.356 50.653 11.838 1.00 0.00 H

ATOM 1233 CG PHE 75 60.266 52.790 11.766 1.00 0.00 C

ATOM 1234 CD1 PHE 75 59.451 53.843 11.364 1.00 0.00 C

ATOM 1235 HD1 PHE 75 58.403 53.740 11.604 1.00 0.00 H

ATOM 1236 CE1 PHE 75 59.949 54.969 10.758 1.00 0.00 C

ATOM 1237 HE1 PHE 75 59.433 55.917 10.729 1.00 0.00 H

ATOM 1238 CZ PHE 75 61.380 54.934 10.516 1.00 0.00 C

ATOM 1239 HZ PHE 75 61.874 55.824 10.155 1.00 0.00 H

ATOM 1240 CE2 PHE 75 62.209 53.913 11.007 1.00 0.00 C

ATOM 1241 HE2 PHE 75 63.284 53.999 10.939 1.00 0.00 H

ATOM 1242 CD2 PHE 75 61.644 52.791 11.585 1.00 0.00 C

ATOM 1243 HD2 PHE 75 62.277 51.957 11.848 1.00 0.00 H

ATOM 1244 C PHE 75 58.758 50.598 9.907 1.00 0.00 C

ATOM 1245 O PHE 75 59.151 51.399 9.073 1.00 0.00 O

ATOM 1246 N ASN 76 58.822 49.276 9.586 1.00 0.00 N

ATOM 1247 H ASN 76 58.613 48.728 10.408 1.00 0.00 H

ATOM 1248 CA ASN 76 58.982 48.679 8.311 1.00 0.00 C

ATOM 1249 HA ASN 76 59.881 49.074 7.839 1.00 0.00 H

ATOM 1250 CB ASN 76 59.327 47.230 8.453 1.00 0.00 C

ATOM 1251 HB2 ASN 76 59.924 46.982 9.331 1.00 0.00 H

ATOM 1252 HB3 ASN 76 58.356 46.735 8.444 1.00 0.00 H

ATOM 1253 CG ASN 76 60.070 46.638 7.292 1.00 0.00 C

ATOM 1254 OD1 ASN 76 60.891 47.296 6.650 1.00 0.00 O

ATOM 1255 ND2 ASN 76 60.026 45.335 7.246 1.00 0.00 N

ATOM 1256 HD21 ASN 76 60.417 44.946 6.400 1.00 0.00 H

ATOM 1257 HD22 ASN 76 59.292 44.899 7.786 1.00 0.00 H

ATOM 1258 C ASN 76 57.769 48.840 7.406 1.00 0.00 C

ATOM 1259 O ASN 76 57.525 48.007 6.477 1.00 0.00 O

ATOM 1260 N LYS 77 56.831 49.763 7.614 1.00 0.00 N

ATOM 1261 H LYS 77 56.951 50.410 8.380 1.00 0.00 H

ATOM 1262 CA LYS 77 55.526 49.876 6.863 1.00 0.00 C

ATOM 1263 HA LYS 77 55.033 50.664 7.431 1.00 0.00 H

ATOM 1264 CB LYS 77 55.653 50.440 5.403 1.00 0.00 C

ATOM 1265 HB2 LYS 77 56.396 49.810 4.915 1.00 0.00 H

ATOM 1266 HB3 LYS 77 54.624 50.331 5.060 1.00 0.00 H

ATOM 1267 CG LYS 77 56.110 51.965 5.277 1.00 0.00 C

ATOM 1268 HG2 LYS 77 55.380 52.540 5.846 1.00 0.00 H

ATOM 1269 HG3 LYS 77 57.063 52.023 5.802 1.00 0.00 H

ATOM 1270 CD LYS 77 56.232 52.561 3.875 1.00 0.00 C

ATOM 1271 HD2 LYS 77 55.342 52.291 3.306 1.00 0.00 H

ATOM 1272 HD3 LYS 77 56.137 53.636 4.024 1.00 0.00 H

ATOM 1273 CE LYS 77 57.552 52.034 3.189 1.00 0.00 C

ATOM 1274 HE2 LYS 77 57.802 52.738 2.395 1.00 0.00 H

ATOM 1275 HE3 LYS 77 58.429 52.167 3.821 1.00 0.00 H

ATOM 1276 NZ LYS 77 57.587 50.649 2.676 1.00 0.00 N

ATOM 1277 HZ1 LYS 77 57.498 50.054 3.486 1.00 0.00 H

ATOM 1278 HZ2 LYS 77 56.873 50.440 1.993 1.00 0.00 H

ATOM 1279 HZ3 LYS 77 58.488 50.522 2.238 1.00 0.00 H

ATOM 1280 C LYS 77 54.711 48.629 7.152 1.00 0.00 C

ATOM 1281 O LYS 77 54.333 47.876 6.226 1.00 0.00 O

ATOM 1282 N THR 78 54.607 48.344 8.466 1.00 0.00 N

ATOM 1283 H THR 78 54.965 49.003 9.142 1.00 0.00 H

ATOM 1284 CA THR 78 53.751 47.288 9.043 1.00 0.00 C

ATOM 1285 HA THR 78 54.040 47.179 10.088 1.00 0.00 H

ATOM 1286 CB THR 78 52.279 47.726 8.950 1.00 0.00 C

ATOM 1287 HB THR 78 51.877 47.658 7.939 1.00 0.00 H

ATOM 1288 CG2 THR 78 51.457 46.955 9.953 1.00 0.00 C

ATOM 1289 HG21 THR 78 50.439 47.344 9.934 1.00 0.00 H

ATOM 1290 HG22 THR 78 51.475 45.874 9.813 1.00 0.00 H

ATOM 1291 HG23 THR 78 51.882 47.131 10.942 1.00 0.00 H

ATOM 1292 OG1 THR 78 52.131 49.101 9.332 1.00 0.00 O

ATOM 1293 HG1 THR 78 51.280 49.415 9.017 1.00 0.00 H

ATOM 1294 C THR 78 53.980 45.851 8.462 1.00 0.00 C

ATOM 1295 O THR 78 53.094 45.212 7.869 1.00 0.00 O

ATOM 1296 N ASP 79 55.215 45.353 8.589 1.00 0.00 N

ATOM 1297 H ASP 79 55.895 45.900 9.098 1.00 0.00 H

ATOM 1298 CA ASP 79 55.710 44.316 7.695 1.00 0.00 C

ATOM 1299 HA ASP 79 54.879 43.642 7.486 1.00 0.00 H

ATOM 1300 CB ASP 79 56.043 44.868 6.270 1.00 0.00 C

ATOM 1301 HB2 ASP 79 55.160 45.396 5.909 1.00 0.00 H

ATOM 1302 HB3 ASP 79 56.924 45.506 6.353 1.00 0.00 H

ATOM 1303 CG ASP 79 56.448 43.707 5.337 1.00 0.00 C

ATOM 1304 OD1 ASP 79 57.494 43.015 5.457 1.00 0.00 O

ATOM 1305 OD2 ASP 79 55.595 43.365 4.448 1.00 0.00 O

ATOM 1306 C ASP 79 56.947 43.545 8.311 1.00 0.00 C

ATOM 1307 O ASP 79 57.946 44.166 8.593 1.00 0.00 O

ATOM 1308 N TRP 80 56.797 42.242 8.691 1.00 0.00 N

ATOM 1309 H TRP 80 56.005 41.741 8.315 1.00 0.00 H

ATOM 1310 CA TRP 80 57.629 41.423 9.601 1.00 0.00 C

ATOM 1311 HA TRP 80 57.788 42.013 10.504 1.00 0.00 H

ATOM 1312 CB TRP 80 56.855 40.139 10.000 1.00 0.00 C

ATOM 1313 HB2 TRP 80 55.800 40.326 10.203 1.00 0.00 H

ATOM 1314 HB3 TRP 80 56.879 39.412 9.189 1.00 0.00 H

ATOM 1315 CG TRP 80 57.359 39.326 11.190 1.00 0.00 C

ATOM 1316 CD1 TRP 80 57.670 38.044 11.041 1.00 0.00 C

ATOM 1317 HD1 TRP 80 57.550 37.449 10.147 1.00 0.00 H

ATOM 1318 NE1 TRP 80 57.692 37.483 12.328 1.00 0.00 N

ATOM 1319 HE1 TRP 80 57.971 36.538 12.551 1.00 0.00 H

ATOM 1320 CE2 TRP 80 57.621 38.466 13.372 1.00 0.00 C

ATOM 1321 CZ2 TRP 80 57.636 38.495 14.758 1.00 0.00 C

ATOM 1322 HZ2 TRP 80 57.878 37.615 15.335 1.00 0.00 H

ATOM 1323 CH2 TRP 80 57.607 39.716 15.402 1.00 0.00 C

ATOM 1324 HH2 TRP 80 57.892 39.780 16.442 1.00 0.00 H

ATOM 1325 CZ3 TRP 80 57.402 40.893 14.620 1.00 0.00 C

ATOM 1326 HZ3 TRP 80 57.365 41.868 15.083 1.00 0.00 H

ATOM 1327 CE3 TRP 80 57.195 40.855 13.257 1.00 0.00 C

ATOM 1328 HE3 TRP 80 57.091 41.844 12.836 1.00 0.00 H

ATOM 1329 CD2 TRP 80 57.330 39.650 12.575 1.00 0.00 C

ATOM 1330 C TRP 80 58.956 41.065 8.958 1.00 0.00 C

ATOM 1331 O TRP 80 59.010 40.703 7.788 1.00 0.00 O

ATOM 1332 N ILE 81 59.958 41.135 9.765 1.00 0.00 N

ATOM 1333 H ILE 81 59.824 41.088 10.765 1.00 0.00 H

ATOM 1334 CA ILE 81 61.350 40.841 9.375 1.00 0.00 C

ATOM 1335 HA ILE 81 61.475 40.849 8.293 1.00 0.00 H

ATOM 1336 CB ILE 81 62.319 41.930 10.005 1.00 0.00 C

ATOM 1337 HB ILE 81 61.828 42.902 10.046 1.00 0.00 H

ATOM 1338 CG2 ILE 81 62.882 41.444 11.335 1.00 0.00 C

ATOM 1339 HG21 ILE 81 63.752 40.811 11.162 1.00 0.00 H

ATOM 1340 HG22 ILE 81 63.263 42.373 11.760 1.00 0.00 H

ATOM 1341 HG23 ILE 81 62.117 40.979 11.957 1.00 0.00 H

ATOM 1342 CG1 ILE 81 63.457 42.099 8.840 1.00 0.00 C

ATOM 1343 HG12 ILE 81 64.371 42.386 9.359 1.00 0.00 H

ATOM 1344 HG13 ILE 81 63.759 41.179 8.338 1.00 0.00 H

ATOM 1345 CD1 ILE 81 63.065 43.183 7.806 1.00 0.00 C

ATOM 1346 HD11 ILE 81 64.019 43.697 7.687 1.00 0.00 H

ATOM 1347 HD12 ILE 81 62.567 42.817 6.908 1.00 0.00 H

ATOM 1348 HD13 ILE 81 62.516 43.979 8.310 1.00 0.00 H

ATOM 1349 C ILE 81 61.761 39.377 9.636 1.00 0.00 C

ATOM 1350 O ILE 81 62.602 38.867 8.919 1.00 0.00 O

ATOM 1351 N PHE 82 61.225 38.737 10.641 1.00 0.00 N

ATOM 1352 H PHE 82 60.472 39.165 11.159 1.00 0.00 H

ATOM 1353 CA PHE 82 61.567 37.287 11.082 1.00 0.00 C

ATOM 1354 HA PHE 82 62.529 37.112 10.599 1.00 0.00 H

ATOM 1355 CB PHE 82 61.624 37.133 12.619 1.00 0.00 C

ATOM 1356 HB2 PHE 82 60.656 37.028 13.109 1.00 0.00 H

ATOM 1357 HB3 PHE 82 62.146 36.207 12.864 1.00 0.00 H

ATOM 1358 CG PHE 82 62.449 38.158 13.326 1.00 0.00 C

ATOM 1359 CD1 PHE 82 63.758 38.362 12.865 1.00 0.00 C

ATOM 1360 HD1 PHE 82 64.153 37.798 12.032 1.00 0.00 H

ATOM 1361 CE1 PHE 82 64.609 39.281 13.516 1.00 0.00 C

ATOM 1362 HE1 PHE 82 65.581 39.497 13.098 1.00 0.00 H

ATOM 1363 CZ PHE 82 63.976 40.184 14.429 1.00 0.00 C

ATOM 1364 HZ PHE 82 64.461 41.105 14.719 1.00 0.00 H

ATOM 1365 CE2 PHE 82 62.686 39.966 14.834 1.00 0.00 C

ATOM 1366 HE2 PHE 82 62.216 40.559 15.604 1.00 0.00 H

ATOM 1367 CD2 PHE 82 61.899 38.859 14.394 1.00 0.00 C

ATOM 1368 HD2 PHE 82 60.829 38.889 14.539 1.00 0.00 H

ATOM 1369 C PHE 82 60.602 36.299 10.516 1.00 0.00 C

ATOM 1370 O PHE 82 59.739 36.694 9.717 1.00 0.00 O

ATOM 1371 N GLY 83 60.724 34.977 10.885 1.00 0.00 N

ATOM 1372 H GLY 83 61.543 34.691 11.400 1.00 0.00 H

ATOM 1373 CA GLY 83 59.849 33.845 10.620 1.00 0.00 C

ATOM 1374 HA2 GLY 83 59.654 33.758 9.551 1.00 0.00 H

ATOM 1375 HA3 GLY 83 60.373 32.901 10.768 1.00 0.00 H

ATOM 1376 C GLY 83 58.476 33.723 11.379 1.00 0.00 C

ATOM 1377 O GLY 83 58.181 34.597 12.274 1.00 0.00 O

ATOM 1378 N ASP 84 57.645 32.669 11.097 1.00 0.00 N

ATOM 1379 H ASP 84 58.007 32.006 10.426 1.00 0.00 H

ATOM 1380 CA ASP 84 56.384 32.297 11.686 1.00 0.00 C

ATOM 1381 HA ASP 84 55.785 33.202 11.589 1.00 0.00 H

ATOM 1382 CB ASP 84 55.863 31.131 10.792 1.00 0.00 C

ATOM 1383 HB2 ASP 84 55.856 31.512 9.771 1.00 0.00 H

ATOM 1384 HB3 ASP 84 56.524 30.292 11.007 1.00 0.00 H

ATOM 1385 CG ASP 84 54.502 30.699 11.310 1.00 0.00 C

ATOM 1386 OD1 ASP 84 53.476 31.031 10.644 1.00 0.00 O

ATOM 1387 OD2 ASP 84 54.305 30.039 12.338 1.00 0.00 O

ATOM 1388 C ASP 84 56.499 31.989 13.156 1.00 0.00 C

ATOM 1389 O ASP 84 55.642 32.456 13.949 1.00 0.00 O

ATOM 1390 N ALA 85 57.365 31.046 13.529 1.00 0.00 N

ATOM 1391 H ALA 85 57.912 30.638 12.784 1.00 0.00 H

ATOM 1392 CA ALA 85 57.634 30.540 14.836 1.00 0.00 C

ATOM 1393 HA ALA 85 56.769 30.008 15.233 1.00 0.00 H

ATOM 1394 CB ALA 85 58.705 29.431 14.841 1.00 0.00 C

ATOM 1395 HB1 ALA 85 58.938 29.328 15.901 1.00 0.00 H

ATOM 1396 HB2 ALA 85 58.232 28.533 14.443 1.00 0.00 H

ATOM 1397 HB3 ALA 85 59.635 29.605 14.300 1.00 0.00 H

ATOM 1398 C ALA 85 57.842 31.750 15.865 1.00 0.00 C

ATOM 1399 O ALA 85 57.218 31.772 16.943 1.00 0.00 O

ATOM 1400 N MET 86 58.622 32.782 15.402 1.00 0.00 N

ATOM 1401 H MET 86 59.103 32.749 14.515 1.00 0.00 H

ATOM 1402 CA MET 86 58.811 34.034 15.939 1.00 0.00 C

ATOM 1403 HA MET 86 59.021 33.798 16.982 1.00 0.00 H

ATOM 1404 CB MET 86 59.954 34.969 15.225 1.00 0.00 C

ATOM 1405 HB2 MET 86 60.886 34.428 15.060 1.00 0.00 H

ATOM 1406 HB3 MET 86 59.735 35.132 14.169 1.00 0.00 H

ATOM 1407 CG MET 86 60.312 36.340 15.920 1.00 0.00 C

ATOM 1408 HG2 MET 86 60.987 36.914 15.284 1.00 0.00 H

ATOM 1409 HG3 MET 86 59.419 36.965 15.924 1.00 0.00 H

ATOM 1410 SD MET 86 60.797 36.261 17.696 1.00 0.00 S

ATOM 1411 CE MET 86 62.355 35.410 17.575 1.00 0.00 C

ATOM 1412 HE1 MET 86 62.167 34.401 17.208 1.00 0.00 H

ATOM 1413 HE2 MET 86 62.866 35.752 16.675 1.00 0.00 H

ATOM 1414 HE3 MET 86 63.108 35.617 18.336 1.00 0.00 H

ATOM 1415 C MET 86 57.499 34.895 16.078 1.00 0.00 C

ATOM 1416 O MET 86 57.334 35.671 16.977 1.00 0.00 O

ATOM 1417 N CYX 87 56.705 34.981 15.011 1.00 0.00 N

ATOM 1418 H CYX 87 56.952 34.365 14.249 1.00 0.00 H

ATOM 1419 CA CYX 87 55.362 35.623 14.981 1.00 0.00 C

ATOM 1420 HA CYX 87 55.637 36.651 15.216 1.00 0.00 H

ATOM 1421 CB CYX 87 54.699 35.757 13.647 1.00 0.00 C

ATOM 1422 HB2 CYX 87 55.293 36.442 13.043 1.00 0.00 H

ATOM 1423 HB3 CYX 87 54.653 34.786 13.153 1.00 0.00 H

ATOM 1424 SG CYX 87 53.008 36.565 13.843 1.00 0.00 S

ATOM 1425 C CYX 87 54.394 35.081 16.032 1.00 0.00 C

ATOM 1426 O CYX 87 54.002 35.862 16.859 1.00 0.00 O

ATOM 1427 N LYS 88 54.334 33.772 16.115 1.00 0.00 N

ATOM 1428 H LYS 88 54.958 33.281 15.491 1.00 0.00 H

ATOM 1429 CA LYS 88 53.859 33.019 17.229 1.00 0.00 C

ATOM 1430 HA LYS 88 52.799 33.262 17.298 1.00 0.00 H

ATOM 1431 CB LYS 88 53.870 31.428 16.995 1.00 0.00 C

ATOM 1432 HB2 LYS 88 54.773 31.097 16.482 1.00 0.00 H

ATOM 1433 HB3 LYS 88 53.959 30.939 17.966 1.00 0.00 H

ATOM 1434 CG LYS 88 52.638 30.808 16.208 1.00 0.00 C

ATOM 1435 HG2 LYS 88 52.840 29.741 16.116 1.00 0.00 H

ATOM 1436 HG3 LYS 88 51.760 31.016 16.820 1.00 0.00 H

ATOM 1437 CD LYS 88 52.557 31.425 14.810 1.00 0.00 C

ATOM 1438 HD2 LYS 88 52.466 32.511 14.850 1.00 0.00 H

ATOM 1439 HD3 LYS 88 53.504 31.166 14.338 1.00 0.00 H

ATOM 1440 CE LYS 88 51.327 30.790 14.000 1.00 0.00 C

ATOM 1441 HE2 LYS 88 50.516 30.548 14.687 1.00 0.00 H

ATOM 1442 HE3 LYS 88 51.033 31.515 13.242 1.00 0.00 H

ATOM 1443 NZ LYS 88 51.741 29.605 13.254 1.00 0.00 N

ATOM 1444 HZ1 LYS 88 50.937 29.162 12.832 1.00 0.00 H

ATOM 1445 HZ2 LYS 88 52.293 30.048 12.533 1.00 0.00 H

ATOM 1446 HZ3 LYS 88 52.297 28.989 13.830 1.00 0.00 H

ATOM 1447 C LYS 88 54.348 33.485 18.646 1.00 0.00 C

ATOM 1448 O LYS 88 53.614 33.799 19.506 1.00 0.00 O

ATOM 1449 N LEU 89 55.735 33.478 18.885 1.00 0.00 N

ATOM 1450 H LEU 89 56.396 33.038 18.262 1.00 0.00 H

ATOM 1451 CA LEU 89 56.362 33.853 20.137 1.00 0.00 C

ATOM 1452 HA LEU 89 56.022 33.158 20.905 1.00 0.00 H

ATOM 1453 CB LEU 89 57.877 33.770 20.109 1.00 0.00 C

ATOM 1454 HB2 LEU 89 58.109 32.824 19.620 1.00 0.00 H

ATOM 1455 HB3 LEU 89 58.329 34.558 19.506 1.00 0.00 H

ATOM 1456 CG LEU 89 58.545 33.968 21.540 1.00 0.00 C

ATOM 1457 HG LEU 89 58.356 34.987 21.876 1.00 0.00 H

ATOM 1458 CD1 LEU 89 58.065 33.036 22.687 1.00 0.00 C

ATOM 1459 HD11 LEU 89 58.466 33.423 23.624 1.00 0.00 H

ATOM 1460 HD12 LEU 89 56.996 33.238 22.749 1.00 0.00 H

ATOM 1461 HD13 LEU 89 58.321 31.981 22.588 1.00 0.00 H

ATOM 1462 CD2 LEU 89 60.093 33.665 21.452 1.00 0.00 C

ATOM 1463 HD21 LEU 89 60.470 33.880 20.452 1.00 0.00 H

ATOM 1464 HD22 LEU 89 60.629 34.367 22.091 1.00 0.00 H

ATOM 1465 HD23 LEU 89 60.373 32.707 21.889 1.00 0.00 H

ATOM 1466 C LEU 89 55.931 35.310 20.467 1.00 0.00 C

ATOM 1467 O LEU 89 55.451 35.511 21.590 1.00 0.00 O

ATOM 1468 N GLN 90 56.070 36.231 19.476 1.00 0.00 N

ATOM 1469 H GLN 90 56.560 35.920 18.650 1.00 0.00 H

ATOM 1470 CA GLN 90 55.529 37.555 19.577 1.00 0.00 C

ATOM 1471 HA GLN 90 56.232 38.109 20.198 1.00 0.00 H

ATOM 1472 CB GLN 90 55.545 38.386 18.246 1.00 0.00 C

ATOM 1473 HB2 GLN 90 56.577 38.418 17.896 1.00 0.00 H

ATOM 1474 HB3 GLN 90 54.929 37.874 17.507 1.00 0.00 H

ATOM 1475 CG GLN 90 55.011 39.856 18.326 1.00 0.00 C

ATOM 1476 HG2 GLN 90 55.192 40.167 19.354 1.00 0.00 H

ATOM 1477 HG3 GLN 90 55.585 40.472 17.634 1.00 0.00 H

ATOM 1478 CD GLN 90 53.482 40.032 18.065 1.00 0.00 C

ATOM 1479 OE1 GLN 90 53.007 39.970 16.961 1.00 0.00 O

ATOM 1480 NE2 GLN 90 52.686 40.229 19.089 1.00 0.00 N

ATOM 1481 HE21 GLN 90 51.838 40.745 18.901 1.00 0.00 H

ATOM 1482 HE22 GLN 90 52.920 39.868 20.002 1.00 0.00 H

ATOM 1483 C GLN 90 54.127 37.633 20.079 1.00 0.00 C

ATOM 1484 O GLN 90 53.831 38.387 20.968 1.00 0.00 O

ATOM 1485 N ARG 91 53.195 36.922 19.516 1.00 0.00 N

ATOM 1486 H ARG 91 53.539 36.375 18.740 1.00 0.00 H

ATOM 1487 CA ARG 91 51.829 36.815 19.923 1.00 0.00 C

ATOM 1488 HA ARG 91 51.482 37.817 20.175 1.00 0.00 H

ATOM 1489 CB ARG 91 50.899 36.360 18.737 1.00 0.00 C

ATOM 1490 HB2 ARG 91 50.975 35.286 18.570 1.00 0.00 H

ATOM 1491 HB3 ARG 91 49.857 36.471 19.037 1.00 0.00 H

ATOM 1492 CG ARG 91 50.968 37.206 17.499 1.00 0.00 C

ATOM 1493 HG2 ARG 91 50.747 38.226 17.814 1.00 0.00 H

ATOM 1494 HG3 ARG 91 51.999 37.235 17.147 1.00 0.00 H

ATOM 1495 CD ARG 91 49.975 36.937 16.370 1.00 0.00 C

ATOM 1496 HD2 ARG 91 50.015 35.900 16.034 1.00 0.00 H

ATOM 1497 HD3 ARG 91 48.981 37.322 16.595 1.00 0.00 H

ATOM 1498 NE ARG 91 50.409 37.606 15.103 1.00 0.00 N

ATOM 1499 HE ARG 91 50.248 37.024 14.293 1.00 0.00 H

ATOM 1500 CZ ARG 91 50.167 38.914 14.859 1.00 0.00 C

ATOM 1501 NH1 ARG 91 50.543 39.805 15.660 1.00 0.00 N

ATOM 1502 HH11 ARG 91 51.175 39.697 16.440 1.00 0.00 H

ATOM 1503 HH12 ARG 91 50.174 40.733 15.510 1.00 0.00 H

ATOM 1504 NH2 ARG 91 49.658 39.232 13.683 1.00 0.00 N

ATOM 1505 HH21 ARG 91 49.232 40.137 13.542 1.00 0.00 H

ATOM 1506 HH22 ARG 91 49.332 38.469 13.107 1.00 0.00 H

ATOM 1507 C ARG 91 51.580 35.964 21.174 1.00 0.00 C

ATOM 1508 O ARG 91 50.541 36.163 21.749 1.00 0.00 O

ATOM 1509 N PHE 92 52.506 35.158 21.693 1.00 0.00 N

ATOM 1510 H PHE 92 53.346 35.029 21.147 1.00 0.00 H

ATOM 1511 CA PHE 92 52.434 34.451 22.982 1.00 0.00 C

ATOM 1512 HA PHE 92 51.366 34.344 23.176 1.00 0.00 H

ATOM 1513 CB PHE 92 53.045 33.084 22.952 1.00 0.00 C

ATOM 1514 HB2 PHE 92 52.521 32.407 22.277 1.00 0.00 H

ATOM 1515 HB3 PHE 92 54.077 33.161 22.610 1.00 0.00 H

ATOM 1516 CG PHE 92 53.205 32.369 24.274 1.00 0.00 C

ATOM 1517 CD1 PHE 92 54.432 32.503 24.928 1.00 0.00 C

ATOM 1518 HD1 PHE 92 55.201 33.190 24.608 1.00 0.00 H

ATOM 1519 CE1 PHE 92 54.680 31.744 26.052 1.00 0.00 C

ATOM 1520 HE1 PHE 92 55.627 31.774 26.571 1.00 0.00 H

ATOM 1521 CZ PHE 92 53.729 30.771 26.451 1.00 0.00 C

ATOM 1522 HZ PHE 92 54.035 30.218 27.327 1.00 0.00 H

ATOM 1523 CE2 PHE 92 52.453 30.668 25.880 1.00 0.00 C

ATOM 1524 HE2 PHE 92 51.690 29.928 26.071 1.00 0.00 H

ATOM 1525 CD2 PHE 92 52.213 31.523 24.757 1.00 0.00 C

ATOM 1526 HD2 PHE 92 51.346 31.349 24.137 1.00 0.00 H

ATOM 1527 C PHE 92 52.988 35.344 24.130 1.00 0.00 C

ATOM 1528 O PHE 92 52.139 35.820 24.873 1.00 0.00 O

ATOM 1529 N ILE 93 54.155 35.997 23.972 1.00 0.00 N

ATOM 1530 H ILE 93 54.613 35.727 23.114 1.00 0.00 H

ATOM 1531 CA ILE 93 54.762 36.798 25.073 1.00 0.00 C

ATOM 1532 HA ILE 93 54.698 36.195 25.978 1.00 0.00 H

ATOM 1533 CB ILE 93 56.274 37.012 24.933 1.00 0.00 C

ATOM 1534 HB ILE 93 56.653 37.378 25.887 1.00 0.00 H

ATOM 1535 CG2 ILE 93 57.025 35.708 24.741 1.00 0.00 C

ATOM 1536 HG21 ILE 93 58.100 35.805 24.897 1.00 0.00 H

ATOM 1537 HG22 ILE 93 56.678 35.018 25.511 1.00 0.00 H

ATOM 1538 HG23 ILE 93 56.852 35.181 23.803 1.00 0.00 H

ATOM 1539 CG1 ILE 93 56.578 38.122 23.915 1.00 0.00 C

ATOM 1540 HG12 ILE 93 56.271 37.863 22.902 1.00 0.00 H

ATOM 1541 HG13 ILE 93 56.174 39.056 24.307 1.00 0.00 H

ATOM 1542 CD1 ILE 93 58.085 38.343 23.741 1.00 0.00 C

ATOM 1543 HD11 ILE 93 58.450 37.625 23.006 1.00 0.00 H

ATOM 1544 HD12 ILE 93 58.295 39.361 23.414 1.00 0.00 H

ATOM 1545 HD13 ILE 93 58.491 38.167 24.738 1.00 0.00 H

ATOM 1546 C ILE 93 54.011 38.135 25.236 1.00 0.00 C

ATOM 1547 O ILE 93 53.998 38.668 26.316 1.00 0.00 O

ATOM 1548 N PHE 94 53.137 38.477 24.331 1.00 0.00 N

ATOM 1549 H PHE 94 53.014 37.831 23.565 1.00 0.00 H

ATOM 1550 CA PHE 94 52.330 39.703 24.478 1.00 0.00 C

ATOM 1551 HA PHE 94 52.929 40.491 24.933 1.00 0.00 H

ATOM 1552 CB PHE 94 52.116 40.318 23.037 1.00 0.00 C

ATOM 1553 HB2 PHE 94 53.045 40.854 22.846 1.00 0.00 H

ATOM 1554 HB3 PHE 94 52.051 39.640 22.186 1.00 0.00 H

ATOM 1555 CG PHE 94 50.921 41.216 22.926 1.00 0.00 C

ATOM 1556 CD1 PHE 94 51.115 42.583 23.138 1.00 0.00 C

ATOM 1557 HD1 PHE 94 52.088 43.052 23.161 1.00 0.00 H

ATOM 1558 CE1 PHE 94 49.984 43.469 23.458 1.00 0.00 C

ATOM 1559 HE1 PHE 94 50.080 44.544 23.440 1.00 0.00 H

ATOM 1560 CZ PHE 94 48.668 42.896 23.421 1.00 0.00 C

ATOM 1561 HZ PHE 94 47.748 43.398 23.684 1.00 0.00 H

ATOM 1562 CE2 PHE 94 48.514 41.583 23.086 1.00 0.00 C

ATOM 1563 HE2 PHE 94 47.548 41.134 22.907 1.00 0.00 H

ATOM 1564 CD2 PHE 94 49.611 40.733 22.835 1.00 0.00 C

ATOM 1565 HD2 PHE 94 49.448 39.670 22.730 1.00 0.00 H

ATOM 1566 C PHE 94 51.101 39.387 25.258 1.00 0.00 C

ATOM 1567 O PHE 94 50.767 40.190 26.110 1.00 0.00 O

ATOM 1568 N HID 95 50.426 38.304 24.903 1.00 0.00 N

ATOM 1569 H HID 95 50.879 37.599 24.339 1.00 0.00 H

ATOM 1570 CA HID 95 49.218 37.853 25.594 1.00 0.00 C

ATOM 1571 HA HID 95 48.492 38.648 25.764 1.00 0.00 H

ATOM 1572 CB HID 95 48.549 36.767 24.640 1.00 0.00 C

ATOM 1573 HB2 HID 95 49.258 36.070 24.193 1.00 0.00 H

ATOM 1574 HB3 HID 95 47.770 36.272 25.218 1.00 0.00 H

ATOM 1575 CG HID 95 47.819 37.402 23.578 1.00 0.00 C

ATOM 1576 ND1 HID 95 48.306 37.577 22.364 1.00 0.00 N

ATOM 1577 HD1 HID 95 49.002 36.942 21.999 1.00 0.00 H

ATOM 1578 CE1 HID 95 47.417 38.339 21.653 1.00 0.00 C

ATOM 1579 HE1 HID 95 47.434 38.498 20.585 1.00 0.00 H

ATOM 1580 NE2 HID 95 46.386 38.689 22.459 1.00 0.00 N

ATOM 1581 CD2 HID 95 46.692 38.225 23.748 1.00 0.00 C

ATOM 1582 HD2 HID 95 46.131 38.406 24.652 1.00 0.00 H

ATOM 1583 C HID 95 49.555 37.243 26.944 1.00 0.00 C

ATOM 1584 O HID 95 48.761 37.480 27.808 1.00 0.00 O

ATOM 1585 N VAL 96 50.694 36.623 27.115 1.00 0.00 N

ATOM 1586 H VAL 96 51.323 36.411 26.354 1.00 0.00 H

ATOM 1587 CA VAL 96 51.229 36.212 28.482 1.00 0.00 C

ATOM 1588 HA VAL 96 50.472 35.667 29.046 1.00 0.00 H

ATOM 1589 CB VAL 96 52.465 35.350 28.369 1.00 0.00 C

ATOM 1590 HB VAL 96 53.167 35.967 27.808 1.00 0.00 H

ATOM 1591 CG1 VAL 96 53.160 34.924 29.703 1.00 0.00 C

ATOM 1592 HG11 VAL 96 52.622 34.219 30.337 1.00 0.00 H

ATOM 1593 HG12 VAL 96 54.142 34.588 29.370 1.00 0.00 H

ATOM 1594 HG13 VAL 96 53.302 35.782 30.359 1.00 0.00 H

ATOM 1595 CG2 VAL 96 52.118 34.086 27.608 1.00 0.00 C

ATOM 1596 HG21 VAL 96 52.982 33.509 27.276 1.00 0.00 H

ATOM 1597 HG22 VAL 96 51.408 33.520 28.212 1.00 0.00 H

ATOM 1598 HG23 VAL 96 51.697 34.338 26.635 1.00 0.00 H

ATOM 1599 C VAL 96 51.461 37.445 29.337 1.00 0.00 C

ATOM 1600 O VAL 96 51.011 37.384 30.483 1.00 0.00 O

ATOM 1601 N ASN 97 51.981 38.520 28.861 1.00 0.00 N

ATOM 1602 H ASN 97 52.266 38.476 27.893 1.00 0.00 H

ATOM 1603 CA ASN 97 52.058 39.812 29.664 1.00 0.00 C

ATOM 1604 HA ASN 97 52.450 39.481 30.626 1.00 0.00 H

ATOM 1605 CB ASN 97 53.017 40.900 29.001 1.00 0.00 C

ATOM 1606 HB2 ASN 97 54.020 40.856 29.425 1.00 0.00 H

ATOM 1607 HB3 ASN 97 52.997 40.811 27.915 1.00 0.00 H

ATOM 1608 CG ASN 97 52.470 42.285 29.303 1.00 0.00 C

ATOM 1609 OD1 ASN 97 51.625 42.885 28.620 1.00 0.00 O

ATOM 1610 ND2 ASN 97 52.706 42.709 30.535 1.00 0.00 N

ATOM 1611 HD21 ASN 97 52.364 43.650 30.669 1.00 0.00 H

ATOM 1612 HD22 ASN 97 53.481 42.256 30.998 1.00 0.00 H

ATOM 1613 C ASN 97 50.612 40.417 29.996 1.00 0.00 C

ATOM 1614 O ASN 97 50.409 40.800 31.148 1.00 0.00 O

ATOM 1615 N LEU 98 49.685 40.231 29.078 1.00 0.00 N

ATOM 1616 H LEU 98 49.969 39.947 28.152 1.00 0.00 H

ATOM 1617 CA LEU 98 48.355 40.758 29.200 1.00 0.00 C

ATOM 1618 HA LEU 98 48.458 41.838 29.299 1.00 0.00 H

ATOM 1619 CB LEU 98 47.694 40.614 27.810 1.00 0.00 C

ATOM 1620 HB2 LEU 98 48.493 40.664 27.071 1.00 0.00 H

ATOM 1621 HB3 LEU 98 47.293 39.615 27.641 1.00 0.00 H

ATOM 1622 CG LEU 98 46.591 41.613 27.522 1.00 0.00 C

ATOM 1623 HG LEU 98 46.063 41.876 28.438 1.00 0.00 H

ATOM 1624 CD1 LEU 98 47.113 42.957 27.105 1.00 0.00 C

ATOM 1625 HD11 LEU 98 46.353 43.623 26.699 1.00 0.00 H

ATOM 1626 HD12 LEU 98 47.440 43.411 28.041 1.00 0.00 H

ATOM 1627 HD13 LEU 98 47.848 42.777 26.321 1.00 0.00 H

ATOM 1628 CD2 LEU 98 45.664 41.224 26.361 1.00 0.00 C

ATOM 1629 HD21 LEU 98 45.338 40.187 26.448 1.00 0.00 H

ATOM 1630 HD22 LEU 98 44.732 41.788 26.389 1.00 0.00 H

ATOM 1631 HD23 LEU 98 46.284 41.255 25.465 1.00 0.00 H

ATOM 1632 C LEU 98 47.478 40.194 30.254 1.00 0.00 C

ATOM 1633 O LEU 98 46.677 40.968 30.865 1.00 0.00 O

ATOM 1634 N TYR 99 47.486 38.890 30.392 1.00 0.00 N

ATOM 1635 H TYR 99 48.178 38.359 29.882 1.00 0.00 H

ATOM 1636 CA TYR 99 46.731 38.219 31.367 1.00 0.00 C

ATOM 1637 HA TYR 99 45.845 38.761 31.699 1.00 0.00 H

ATOM 1638 CB TYR 99 46.211 36.911 30.831 1.00 0.00 C

ATOM 1639 HB2 TYR 99 47.026 36.238 30.566 1.00 0.00 H

ATOM 1640 HB3 TYR 99 45.472 36.437 31.476 1.00 0.00 H

ATOM 1641 CG TYR 99 45.412 37.024 29.456 1.00 0.00 C

ATOM 1642 CD1 TYR 99 44.231 37.779 29.445 1.00 0.00 C

ATOM 1643 HD1 TYR 99 43.949 38.263 30.368 1.00 0.00 H

ATOM 1644 CE1 TYR 99 43.496 37.970 28.270 1.00 0.00 C

ATOM 1645 HE1 TYR 99 42.709 38.707 28.216 1.00 0.00 H

ATOM 1646 CZ TYR 99 43.980 37.484 27.045 1.00 0.00 C

ATOM 1647 OH TYR 99 43.341 37.957 25.964 1.00 0.00 O

ATOM 1648 HH TYR 99 43.835 37.868 25.146 1.00 0.00 H

ATOM 1649 CE2 TYR 99 45.244 36.767 27.044 1.00 0.00 C

ATOM 1650 HE2 TYR 99 45.583 36.339 26.112 1.00 0.00 H

ATOM 1651 CD2 TYR 99 46.004 36.634 28.305 1.00 0.00 C

ATOM 1652 HD2 TYR 99 46.902 36.035 28.287 1.00 0.00 H

ATOM 1653 C TYR 99 47.562 37.995 32.580 1.00 0.00 C

ATOM 1654 O TYR 99 46.957 37.790 33.641 1.00 0.00 O

ATOM 1655 N GLY 100 48.879 38.002 32.502 1.00 0.00 N

ATOM 1656 H GLY 100 49.346 38.035 31.607 1.00 0.00 H

ATOM 1657 CA GLY 100 49.601 38.176 33.793 1.00 0.00 C

ATOM 1658 HA2 GLY 100 49.472 37.354 34.497 1.00 0.00 H

ATOM 1659 HA3 GLY 100 50.672 38.299 33.632 1.00 0.00 H

ATOM 1660 C GLY 100 49.310 39.507 34.508 1.00 0.00 C

ATOM 1661 O GLY 100 49.090 39.457 35.716 1.00 0.00 O

ATOM 1662 N SER 101 49.154 40.613 33.783 1.00 0.00 N

ATOM 1663 H SER 101 49.424 40.531 32.813 1.00 0.00 H

ATOM 1664 CA SER 101 49.079 41.952 34.405 1.00 0.00 C

ATOM 1665 HA SER 101 49.899 42.013 35.121 1.00 0.00 H

ATOM 1666 CB SER 101 49.389 43.045 33.371 1.00 0.00 C

ATOM 1667 HB2 SER 101 48.570 43.050 32.652 1.00 0.00 H

ATOM 1668 HB3 SER 101 49.490 44.022 33.843 1.00 0.00 H

ATOM 1669 OG SER 101 50.571 42.817 32.666 1.00 0.00 O

ATOM 1670 HG SER 101 50.379 42.153 32.000 1.00 0.00 H

ATOM 1671 C SER 101 47.819 42.048 35.254 1.00 0.00 C

ATOM 1672 O SER 101 47.863 42.476 36.378 1.00 0.00 O

ATOM 1673 N ILE 102 46.680 41.976 34.517 1.00 0.00 N

ATOM 1674 H ILE 102 46.623 41.885 33.513 1.00 0.00 H

ATOM 1675 CA ILE 102 45.447 41.750 35.272 1.00 0.00 C

ATOM 1676 HA ILE 102 45.166 42.754 35.589 1.00 0.00 H

ATOM 1677 CB ILE 102 44.271 41.461 34.296 1.00 0.00 C

ATOM 1678 HB ILE 102 44.186 42.311 33.619 1.00 0.00 H

ATOM 1679 CG2 ILE 102 44.495 40.335 33.279 1.00 0.00 C

ATOM 1680 HG21 ILE 102 43.680 40.276 32.558 1.00 0.00 H

ATOM 1681 HG22 ILE 102 45.426 40.667 32.821 1.00 0.00 H

ATOM 1682 HG23 ILE 102 44.661 39.408 33.830 1.00 0.00 H

ATOM 1683 CG1 ILE 102 42.895 41.377 34.888 1.00 0.00 C

ATOM 1684 HG12 ILE 102 42.325 40.603 34.374 1.00 0.00 H

ATOM 1685 HG13 ILE 102 43.017 41.189 35.955 1.00 0.00 H

ATOM 1686 CD1 ILE 102 42.106 42.647 34.909 1.00 0.00 C

ATOM 1687 HD11 ILE 102 41.267 42.489 35.587 1.00 0.00 H

ATOM 1688 HD12 ILE 102 42.740 43.414 35.352 1.00 0.00 H

ATOM 1689 HD13 ILE 102 41.836 42.850 33.873 1.00 0.00 H

ATOM 1690 C ILE 102 45.526 40.867 36.530 1.00 0.00 C

ATOM 1691 O ILE 102 45.118 41.314 37.622 1.00 0.00 O

ATOM 1692 N LEU 103 45.670 39.592 36.348 1.00 0.00 N

ATOM 1693 H LEU 103 45.788 39.260 35.402 1.00 0.00 H

ATOM 1694 CA LEU 103 45.788 38.538 37.395 1.00 0.00 C

ATOM 1695 HA LEU 103 44.829 38.364 37.883 1.00 0.00 H

ATOM 1696 CB LEU 103 46.191 37.198 36.861 1.00 0.00 C

ATOM 1697 HB2 LEU 103 47.059 37.375 36.225 1.00 0.00 H

ATOM 1698 HB3 LEU 103 46.406 36.642 37.773 1.00 0.00 H

ATOM 1699 CG LEU 103 45.025 36.387 36.186 1.00 0.00 C

ATOM 1700 HG LEU 103 44.523 37.061 35.492 1.00 0.00 H

ATOM 1701 CD1 LEU 103 45.577 35.227 35.469 1.00 0.00 C

ATOM 1702 HD11 LEU 103 46.169 34.653 36.182 1.00 0.00 H

ATOM 1703 HD12 LEU 103 44.795 34.573 35.083 1.00 0.00 H

ATOM 1704 HD13 LEU 103 46.268 35.541 34.687 1.00 0.00 H

ATOM 1705 CD2 LEU 103 43.829 36.043 37.169 1.00 0.00 C

ATOM 1706 HD21 LEU 103 43.372 35.091 36.896 1.00 0.00 H

ATOM 1707 HD22 LEU 103 44.289 35.975 38.155 1.00 0.00 H

ATOM 1708 HD23 LEU 103 43.171 36.910 37.213 1.00 0.00 H

ATOM 1709 C LEU 103 46.658 38.982 38.560 1.00 0.00 C

ATOM 1710 O LEU 103 46.316 38.828 39.729 1.00 0.00 O

ATOM 1711 N PHE 104 47.873 39.415 38.365 1.00 0.00 N

ATOM 1712 H PHE 104 48.265 39.253 37.449 1.00 0.00 H

ATOM 1713 CA PHE 104 48.690 39.959 39.400 1.00 0.00 C

ATOM 1714 HA PHE 104 48.629 39.200 40.180 1.00 0.00 H

ATOM 1715 CB PHE 104 50.168 40.172 39.164 1.00 0.00 C

ATOM 1716 HB2 PHE 104 50.312 40.822 38.301 1.00 0.00 H

ATOM 1717 HB3 PHE 104 50.575 40.735 40.005 1.00 0.00 H

ATOM 1718 CG PHE 104 50.934 38.858 38.886 1.00 0.00 C

ATOM 1719 CD1 PHE 104 51.032 37.957 39.948 1.00 0.00 C

ATOM 1720 HD1 PHE 104 50.774 38.369 40.912 1.00 0.00 H

ATOM 1721 CE1 PHE 104 51.568 36.688 39.695 1.00 0.00 C

ATOM 1722 HE1 PHE 104 51.664 35.932 40.460 1.00 0.00 H

ATOM 1723 CZ PHE 104 51.804 36.270 38.355 1.00 0.00 C

ATOM 1724 HZ PHE 104 52.018 35.227 38.171 1.00 0.00 H

ATOM 1725 CE2 PHE 104 51.709 37.191 37.285 1.00 0.00 C

ATOM 1726 HE2 PHE 104 51.976 36.983 36.260 1.00 0.00 H

ATOM 1727 CD2 PHE 104 51.384 38.502 37.591 1.00 0.00 C

ATOM 1728 HD2 PHE 104 51.310 39.157 36.736 1.00 0.00 H

ATOM 1729 C PHE 104 48.133 41.246 40.008 1.00 0.00 C

ATOM 1730 O PHE 104 48.117 41.240 41.270 1.00 0.00 O

ATOM 1731 N LEU 105 47.372 42.156 39.333 1.00 0.00 N

ATOM 1732 H LEU 105 47.383 42.104 38.325 1.00 0.00 H

ATOM 1733 CA LEU 105 46.527 43.138 40.005 1.00 0.00 C

ATOM 1734 HA LEU 105 47.175 43.644 40.721 1.00 0.00 H

ATOM 1735 CB LEU 105 46.188 44.266 38.993 1.00 0.00 C

ATOM 1736 HB2 LEU 105 47.094 44.442 38.413 1.00 0.00 H

ATOM 1737 HB3 LEU 105 45.405 43.792 38.402 1.00 0.00 H

ATOM 1738 CG LEU 105 45.753 45.572 39.638 1.00 0.00 C

ATOM 1739 HG LEU 105 44.937 45.392 40.338 1.00 0.00 H

ATOM 1740 CD1 LEU 105 46.823 46.353 40.376 1.00 0.00 C

ATOM 1741 HD11 LEU 105 47.058 45.769 41.267 1.00 0.00 H

ATOM 1742 HD12 LEU 105 47.703 46.328 39.732 1.00 0.00 H

ATOM 1743 HD13 LEU 105 46.589 47.381 40.654 1.00 0.00 H

ATOM 1744 CD2 LEU 105 45.151 46.472 38.579 1.00 0.00 C

ATOM 1745 HD21 LEU 105 44.223 46.961 38.874 1.00 0.00 H

ATOM 1746 HD22 LEU 105 45.933 47.162 38.263 1.00 0.00 H

ATOM 1747 HD23 LEU 105 44.764 45.953 37.703 1.00 0.00 H

ATOM 1748 C LEU 105 45.241 42.536 40.676 1.00 0.00 C

ATOM 1749 O LEU 105 44.768 43.213 41.533 1.00 0.00 O

ATOM 1750 N THR 106 44.833 41.322 40.379 1.00 0.00 N

ATOM 1751 H THR 106 45.440 40.809 39.756 1.00 0.00 H

ATOM 1752 CA THR 106 43.776 40.563 41.004 1.00 0.00 C

ATOM 1753 HA THR 106 42.994 41.276 41.266 1.00 0.00 H

ATOM 1754 CB THR 106 43.060 39.552 40.050 1.00 0.00 C

ATOM 1755 HB THR 106 43.546 38.581 39.958 1.00 0.00 H

ATOM 1756 CG2 THR 106 41.710 39.140 40.653 1.00 0.00 C

ATOM 1757 HG21 THR 106 40.932 39.902 40.671 1.00 0.00 H

ATOM 1758 HG22 THR 106 41.380 38.335 39.996 1.00 0.00 H

ATOM 1759 HG23 THR 106 41.837 38.585 41.583 1.00 0.00 H

ATOM 1760 OG1 THR 106 42.944 39.971 38.739 1.00 0.00 O

ATOM 1761 HG1 THR 106 43.695 40.530 38.530 1.00 0.00 H

ATOM 1762 C THR 106 44.221 39.872 42.309 1.00 0.00 C

ATOM 1763 O THR 106 43.426 39.932 43.277 1.00 0.00 O

ATOM 1764 N CYX 107 45.449 39.317 42.416 1.00 0.00 N

ATOM 1765 H CYX 107 46.035 39.358 41.594 1.00 0.00 H

ATOM 1766 CA CYX 107 46.138 38.864 43.619 1.00 0.00 C

ATOM 1767 HA CYX 107 45.550 38.119 44.156 1.00 0.00 H

ATOM 1768 CB CYX 107 47.467 38.149 43.275 1.00 0.00 C

ATOM 1769 HB2 CYX 107 48.021 38.669 42.493 1.00 0.00 H

ATOM 1770 HB3 CYX 107 48.164 38.243 44.108 1.00 0.00 H

ATOM 1771 SG CYX 107 47.248 36.338 42.811 1.00 0.00 S

ATOM 1772 C CYX 107 46.271 40.047 44.688 1.00 0.00 C

ATOM 1773 O CYX 107 45.726 39.953 45.802 1.00 0.00 O

ATOM 1774 N ILE 108 47.040 41.034 44.308 1.00 0.00 N

ATOM 1775 H ILE 108 47.658 40.744 43.564 1.00 0.00 H

ATOM 1776 CA ILE 108 47.129 42.320 45.070 1.00 0.00 C

ATOM 1777 HA ILE 108 47.468 42.191 46.098 1.00 0.00 H

ATOM 1778 CB ILE 108 47.941 43.360 44.335 1.00 0.00 C

ATOM 1779 HB ILE 108 47.554 43.467 43.321 1.00 0.00 H

ATOM 1780 CG2 ILE 108 47.869 44.682 45.043 1.00 0.00 C

ATOM 1781 HG21 ILE 108 46.884 45.118 44.882 1.00 0.00 H

ATOM 1782 HG22 ILE 108 47.998 44.515 46.113 1.00 0.00 H

ATOM 1783 HG23 ILE 108 48.531 45.447 44.638 1.00 0.00 H

ATOM 1784 CG1 ILE 108 49.406 42.926 44.190 1.00 0.00 C

ATOM 1785 HG12 ILE 108 49.962 42.849 45.124 1.00 0.00 H

ATOM 1786 HG13 ILE 108 49.374 41.895 43.836 1.00 0.00 H

ATOM 1787 CD1 ILE 108 50.281 43.626 43.147 1.00 0.00 C

ATOM 1788 HD11 ILE 108 51.198 43.059 42.985 1.00 0.00 H

ATOM 1789 HD12 ILE 108 49.880 43.727 42.139 1.00 0.00 H

ATOM 1790 HD13 ILE 108 50.650 44.558 43.576 1.00 0.00 H

ATOM 1791 C ILE 108 45.740 42.945 45.486 1.00 0.00 C

ATOM 1792 O ILE 108 45.575 43.120 46.703 1.00 0.00 O

ATOM 1793 N SER 109 44.733 42.976 44.597 1.00 0.00 N

ATOM 1794 H SER 109 44.960 42.922 43.615 1.00 0.00 H

ATOM 1795 CA SER 109 43.364 43.541 44.859 1.00 0.00 C

ATOM 1796 HA SER 109 43.510 44.480 45.393 1.00 0.00 H

ATOM 1797 CB SER 109 42.555 43.913 43.659 1.00 0.00 C

ATOM 1798 HB2 SER 109 42.214 43.005 43.162 1.00 0.00 H

ATOM 1799 HB3 SER 109 41.616 44.409 43.907 1.00 0.00 H

ATOM 1800 OG SER 109 43.105 44.882 42.819 1.00 0.00 O

ATOM 1801 HG SER 109 43.667 44.460 42.165 1.00 0.00 H

ATOM 1802 C SER 109 42.442 42.712 45.810 1.00 0.00 C

ATOM 1803 O SER 109 41.977 43.192 46.857 1.00 0.00 O

ATOM 1804 N ALA 110 42.350 41.359 45.573 1.00 0.00 N

ATOM 1805 H ALA 110 42.850 40.965 44.788 1.00 0.00 H

ATOM 1806 CA ALA 110 41.728 40.526 46.557 1.00 0.00 C

ATOM 1807 HA ALA 110 40.799 40.978 46.906 1.00 0.00 H

ATOM 1808 CB ALA 110 41.352 39.210 45.819 1.00 0.00 C

ATOM 1809 HB1 ALA 110 40.767 39.410 44.922 1.00 0.00 H

ATOM 1810 HB2 ALA 110 42.275 38.800 45.408 1.00 0.00 H

ATOM 1811 HB3 ALA 110 40.715 38.651 46.504 1.00 0.00 H

ATOM 1812 C ALA 110 42.573 40.367 47.828 1.00 0.00 C

ATOM 1813 O ALA 110 41.964 39.905 48.827 1.00 0.00 O

ATOM 1814 N HID 111 43.890 40.646 47.899 1.00 0.00 N

ATOM 1815 H HID 111 44.456 40.948 47.119 1.00 0.00 H

ATOM 1816 CA HID 111 44.633 40.824 49.194 1.00 0.00 C

ATOM 1817 HA HID 111 44.494 39.959 49.841 1.00 0.00 H

ATOM 1818 CB HID 111 46.123 40.674 49.060 1.00 0.00 C

ATOM 1819 HB2 HID 111 46.241 39.783 48.442 1.00 0.00 H

ATOM 1820 HB3 HID 111 46.630 41.464 48.507 1.00 0.00 H

ATOM 1821 CG HID 111 46.743 40.421 50.442 1.00 0.00 C

ATOM 1822 ND1 HID 111 47.169 41.405 51.302 1.00 0.00 N

ATOM 1823 HD1 HID 111 47.277 42.400 51.167 1.00 0.00 H

ATOM 1824 CE1 HID 111 48.036 40.758 52.188 1.00 0.00 C

ATOM 1825 HE1 HID 111 48.414 41.195 53.101 1.00 0.00 H

ATOM 1826 NE2 HID 111 47.891 39.467 52.038 1.00 0.00 N

ATOM 1827 CD2 HID 111 47.065 39.202 50.984 1.00 0.00 C

ATOM 1828 HD2 HID 111 47.079 38.247 50.481 1.00 0.00 H

ATOM 1829 C HID 111 44.179 42.088 49.854 1.00 0.00 C

ATOM 1830 O HID 111 43.751 42.101 51.001 1.00 0.00 O

ATOM 1831 N ARG 112 44.441 43.264 49.192 1.00 0.00 N

ATOM 1832 H ARG 112 44.829 43.110 48.272 1.00 0.00 H

ATOM 1833 CA ARG 112 43.958 44.587 49.602 1.00 0.00 C

ATOM 1834 HA ARG 112 44.441 44.917 50.522 1.00 0.00 H

ATOM 1835 CB ARG 112 44.310 45.695 48.549 1.00 0.00 C

ATOM 1836 HB2 ARG 112 44.144 45.247 47.569 1.00 0.00 H

ATOM 1837 HB3 ARG 112 43.713 46.576 48.785 1.00 0.00 H

ATOM 1838 CG ARG 112 45.857 46.028 48.618 1.00 0.00 C

ATOM 1839 HG2 ARG 112 46.089 46.303 49.647 1.00 0.00 H

ATOM 1840 HG3 ARG 112 46.524 45.197 48.388 1.00 0.00 H

ATOM 1841 CD ARG 112 46.322 47.077 47.654 1.00 0.00 C

ATOM 1842 HD2 ARG 112 46.412 46.627 46.665 1.00 0.00 H

ATOM 1843 HD3 ARG 112 45.676 47.952 47.723 1.00 0.00 H

ATOM 1844 NE ARG 112 47.651 47.525 48.005 1.00 0.00 N

ATOM 1845 HE ARG 112 48.096 47.042 48.772 1.00 0.00 H

ATOM 1846 CZ ARG 112 48.397 48.540 47.603 1.00 0.00 C

ATOM 1847 NH1 ARG 112 47.971 49.308 46.576 1.00 0.00 N

ATOM 1848 HH11 ARG 112 47.085 49.093 46.141 1.00 0.00 H

ATOM 1849 HH12 ARG 112 48.738 49.670 46.028 1.00 0.00 H

ATOM 1850 NH2 ARG 112 49.585 48.759 48.169 1.00 0.00 N

ATOM 1851 HH21 ARG 112 49.721 48.142 48.957 1.00 0.00 H

ATOM 1852 HH22 ARG 112 50.378 48.940 47.570 1.00 0.00 H

ATOM 1853 C ARG 112 42.488 44.528 50.082 1.00 0.00 C

ATOM 1854 O ARG 112 42.192 45.201 51.031 1.00 0.00 O

ATOM 1855 N TYR 113 41.520 43.786 49.553 1.00 0.00 N

ATOM 1856 H TYR 113 41.759 43.257 48.726 1.00 0.00 H

ATOM 1857 CA TYR 113 40.197 43.582 50.068 1.00 0.00 C

ATOM 1858 HA TYR 113 39.914 44.604 50.321 1.00 0.00 H

ATOM 1859 CB TYR 113 39.271 43.030 48.987 1.00 0.00 C

ATOM 1860 HB2 TYR 113 39.039 43.734 48.187 1.00 0.00 H

ATOM 1861 HB3 TYR 113 39.917 42.244 48.594 1.00 0.00 H

ATOM 1862 CG TYR 113 38.005 42.347 49.451 1.00 0.00 C

ATOM 1863 CD1 TYR 113 38.088 41.004 49.781 1.00 0.00 C

ATOM 1864 HD1 TYR 113 38.907 40.368 49.480 1.00 0.00 H

ATOM 1865 CE1 TYR 113 36.917 40.329 50.178 1.00 0.00 C

ATOM 1866 HE1 TYR 113 37.091 39.309 50.486 1.00 0.00 H

ATOM 1867 CZ TYR 113 35.688 40.940 50.307 1.00 0.00 C

ATOM 1868 OH TYR 113 34.557 40.244 50.593 1.00 0.00 O

ATOM 1869 HH TYR 113 33.819 40.813 50.825 1.00 0.00 H

ATOM 1870 CE2 TYR 113 35.592 42.257 49.788 1.00 0.00 C

ATOM 1871 HE2 TYR 113 34.637 42.756 49.709 1.00 0.00 H

ATOM 1872 CD2 TYR 113 36.774 43.017 49.496 1.00 0.00 C

ATOM 1873 HD2 TYR 113 36.799 43.985 49.018 1.00 0.00 H

ATOM 1874 C TYR 113 40.200 42.722 51.351 1.00 0.00 C

ATOM 1875 O TYR 113 39.561 43.010 52.329 1.00 0.00 O

ATOM 1876 N SER 114 40.841 41.548 51.210 1.00 0.00 N

ATOM 1877 H SER 114 41.210 41.309 50.300 1.00 0.00 H

ATOM 1878 CA SER 114 40.996 40.671 52.360 1.00 0.00 C

ATOM 1879 HA SER 114 40.111 40.128 52.693 1.00 0.00 H

ATOM 1880 CB SER 114 42.149 39.678 52.107 1.00 0.00 C

ATOM 1881 HB2 SER 114 42.955 40.140 51.538 1.00 0.00 H

ATOM 1882 HB3 SER 114 42.492 39.271 53.058 1.00 0.00 H

ATOM 1883 OG SER 114 41.397 38.761 51.270 1.00 0.00 O

ATOM 1884 HG SER 114 41.314 39.186 50.413 1.00 0.00 H

ATOM 1885 C SER 114 41.503 41.309 53.672 1.00 0.00 C

ATOM 1886 O SER 114 41.259 40.878 54.801 1.00 0.00 O

ATOM 1887 N GLY 115 42.459 42.271 53.532 1.00 0.00 N

ATOM 1888 H GLY 115 42.728 42.530 52.594 1.00 0.00 H

ATOM 1889 CA GLY 115 43.144 42.967 54.618 1.00 0.00 C

ATOM 1890 HA2 GLY 115 43.632 42.219 55.243 1.00 0.00 H

ATOM 1891 HA3 GLY 115 43.963 43.502 54.137 1.00 0.00 H

ATOM 1892 C GLY 115 42.230 43.825 55.500 1.00 0.00 C

ATOM 1893 O GLY 115 42.512 44.048 56.719 1.00 0.00 O

ATOM 1894 N VAL 116 41.155 44.383 54.997 1.00 0.00 N

ATOM 1895 H VAL 116 41.126 44.477 53.992 1.00 0.00 H

ATOM 1896 CA VAL 116 39.982 44.878 55.685 1.00 0.00 C

ATOM 1897 HA VAL 116 40.162 45.521 56.547 1.00 0.00 H

ATOM 1898 CB VAL 116 39.135 45.728 54.682 1.00 0.00 C

ATOM 1899 HB VAL 116 38.636 45.133 53.917 1.00 0.00 H

ATOM 1900 CG1 VAL 116 38.195 46.537 55.602 1.00 0.00 C

ATOM 1901 HG11 VAL 116 37.649 45.938 56.332 1.00 0.00 H

ATOM 1902 HG12 VAL 116 38.717 47.338 56.125 1.00 0.00 H

ATOM 1903 HG13 VAL 116 37.501 47.065 54.949 1.00 0.00 H

ATOM 1904 CG2 VAL 116 39.960 46.630 53.806 1.00 0.00 C

ATOM 1905 HG21 VAL 116 40.466 46.164 52.960 1.00 0.00 H

ATOM 1906 HG22 VAL 116 39.345 47.379 53.308 1.00 0.00 H

ATOM 1907 HG23 VAL 116 40.727 47.088 54.431 1.00 0.00 H

ATOM 1908 C VAL 116 39.096 43.779 56.271 1.00 0.00 C

ATOM 1909 O VAL 116 38.624 43.915 57.395 1.00 0.00 O

ATOM 1910 N VAL 117 38.940 42.636 55.549 1.00 0.00 N

ATOM 1911 H VAL 117 39.264 42.640 54.593 1.00 0.00 H

ATOM 1912 CA VAL 117 37.775 41.703 55.746 1.00 0.00 C

ATOM 1913 HA VAL 117 36.906 42.167 56.211 1.00 0.00 H

ATOM 1914 CB VAL 117 37.293 41.064 54.440 1.00 0.00 C

ATOM 1915 HB VAL 117 38.267 40.753 54.063 1.00 0.00 H

ATOM 1916 CG1 VAL 117 36.315 39.906 54.416 1.00 0.00 C

ATOM 1917 HG11 VAL 117 36.759 39.066 54.950 1.00 0.00 H

ATOM 1918 HG12 VAL 117 35.330 40.226 54.757 1.00 0.00 H

ATOM 1919 HG13 VAL 117 36.170 39.617 53.375 1.00 0.00 H

ATOM 1920 CG2 VAL 117 36.711 42.186 53.584 1.00 0.00 C

ATOM 1921 HG21 VAL 117 37.315 43.092 53.619 1.00 0.00 H

ATOM 1922 HG22 VAL 117 36.558 41.883 52.548 1.00 0.00 H

ATOM 1923 HG23 VAL 117 35.754 42.561 53.947 1.00 0.00 H

ATOM 1924 C VAL 117 38.155 40.629 56.852 1.00 0.00 C

ATOM 1925 O VAL 117 37.299 40.157 57.566 1.00 0.00 O

ATOM 1926 N TYR 118 39.449 40.162 56.910 1.00 0.00 N

ATOM 1927 H TYR 118 40.169 40.539 56.309 1.00 0.00 H

ATOM 1928 CA TYR 118 39.777 38.846 57.573 1.00 0.00 C

ATOM 1929 HA TYR 118 38.880 38.256 57.757 1.00 0.00 H

ATOM 1930 CB TYR 118 40.689 38.010 56.712 1.00 0.00 C

ATOM 1931 HB2 TYR 118 41.387 38.664 56.190 1.00 0.00 H

ATOM 1932 HB3 TYR 118 41.117 37.323 57.441 1.00 0.00 H

ATOM 1933 CG TYR 118 39.876 37.266 55.608 1.00 0.00 C

ATOM 1934 CD1 TYR 118 38.968 36.257 56.038 1.00 0.00 C

ATOM 1935 HD1 TYR 118 38.702 36.204 57.083 1.00 0.00 H

ATOM 1936 CE1 TYR 118 38.197 35.579 55.088 1.00 0.00 C

ATOM 1937 HE1 TYR 118 37.462 34.846 55.386 1.00 0.00 H

ATOM 1938 CZ TYR 118 38.383 35.827 53.729 1.00 0.00 C

ATOM 1939 OH TYR 118 37.695 35.196 52.741 1.00 0.00 O

ATOM 1940 HH TYR 118 37.188 34.467 53.107 1.00 0.00 H

ATOM 1941 CE2 TYR 118 39.151 36.931 53.294 1.00 0.00 C

ATOM 1942 HE2 TYR 118 39.238 37.199 52.251 1.00 0.00 H

ATOM 1943 CD2 TYR 118 39.830 37.720 54.271 1.00 0.00 C

ATOM 1944 HD2 TYR 118 40.392 38.620 54.073 1.00 0.00 H

ATOM 1945 C TYR 118 40.524 39.006 58.933 1.00 0.00 C

ATOM 1946 O TYR 118 41.132 40.055 59.185 1.00 0.00 O

ATOM 1947 N PRO 119 40.549 37.962 59.801 1.00 0.00 N

ATOM 1948 CD PRO 119 39.294 37.249 60.116 1.00 0.00 C

ATOM 1949 HD2 PRO 119 39.356 36.429 59.400 1.00 0.00 H

ATOM 1950 HD3 PRO 119 38.419 37.822 59.808 1.00 0.00 H

ATOM 1951 CG PRO 119 39.369 36.711 61.585 1.00 0.00 C

ATOM 1952 HG2 PRO 119 38.906 35.729 61.493 1.00 0.00 H

ATOM 1953 HG3 PRO 119 38.834 37.337 62.298 1.00 0.00 H

ATOM 1954 CB PRO 119 40.886 36.739 61.878 1.00 0.00 C

ATOM 1955 HB2 PRO 119 41.340 35.754 61.766 1.00 0.00 H

ATOM 1956 HB3 PRO 119 41.056 37.071 62.902 1.00 0.00 H

ATOM 1957 CA PRO 119 41.520 37.780 60.896 1.00 0.00 C

ATOM 1958 HA PRO 119 41.477 38.767 61.357 1.00 0.00 H

ATOM 1959 C PRO 119 42.903 37.341 60.370 1.00 0.00 C

ATOM 1960 O PRO 119 43.140 37.175 59.160 1.00 0.00 O

ATOM 1961 N LEU 120 43.967 37.154 61.248 1.00 0.00 N

ATOM 1962 H LEU 120 43.756 37.266 62.230 1.00 0.00 H

ATOM 1963 CA LEU 120 45.367 36.844 60.837 1.00 0.00 C

ATOM 1964 HA LEU 120 45.673 37.634 60.151 1.00 0.00 H

ATOM 1965 CB LEU 120 46.314 37.110 62.051 1.00 0.00 C

ATOM 1966 HB2 LEU 120 45.800 37.694 62.814 1.00 0.00 H

ATOM 1967 HB3 LEU 120 46.405 36.163 62.583 1.00 0.00 H

ATOM 1968 CG LEU 120 47.686 37.795 61.907 1.00 0.00 C

ATOM 1969 HG LEU 120 47.446 38.778 61.500 1.00 0.00 H

ATOM 1970 CD1 LEU 120 48.466 37.974 63.178 1.00 0.00 C

ATOM 1971 HD11 LEU 120 49.370 38.538 62.945 1.00 0.00 H

ATOM 1972 HD12 LEU 120 47.881 38.493 63.936 1.00 0.00 H

ATOM 1973 HD13 LEU 120 48.746 37.025 63.635 1.00 0.00 H

ATOM 1974 CD2 LEU 120 48.577 37.272 60.818 1.00 0.00 C

ATOM 1975 HD21 LEU 120 48.726 36.201 60.955 1.00 0.00 H

ATOM 1976 HD22 LEU 120 48.115 37.366 59.835 1.00 0.00 H

ATOM 1977 HD23 LEU 120 49.556 37.747 60.766 1.00 0.00 H

ATOM 1978 C LEU 120 45.539 35.495 60.149 1.00 0.00 C

ATOM 1979 O LEU 120 46.360 35.309 59.267 1.00 0.00 O

ATOM 1980 N LYS 121 44.769 34.466 60.620 1.00 0.00 N

ATOM 1981 H LYS 121 44.332 34.646 61.512 1.00 0.00 H

ATOM 1982 CA LYS 121 44.773 33.055 60.151 1.00 0.00 C

ATOM 1983 HA LYS 121 45.783 32.645 60.142 1.00 0.00 H

ATOM 1984 CB LYS 121 44.085 32.040 60.991 1.00 0.00 C

ATOM 1985 HB2 LYS 121 43.075 32.451 60.987 1.00 0.00 H

ATOM 1986 HB3 LYS 121 43.969 31.054 60.542 1.00 0.00 H

ATOM 1987 CG LYS 121 44.668 31.873 62.366 1.00 0.00 C

ATOM 1988 HG2 LYS 121 45.748 31.740 62.296 1.00 0.00 H

ATOM 1989 HG3 LYS 121 44.323 32.644 63.055 1.00 0.00 H

ATOM 1990 CD LYS 121 44.144 30.443 62.874 1.00 0.00 C

ATOM 1991 HD2 LYS 121 43.075 30.512 63.076 1.00 0.00 H

ATOM 1992 HD3 LYS 121 44.231 29.637 62.146 1.00 0.00 H

ATOM 1993 CE LYS 121 44.744 29.864 64.106 1.00 0.00 C

ATOM 1994 HE2 LYS 121 44.271 28.885 64.183 1.00 0.00 H

ATOM 1995 HE3 LYS 121 45.832 29.881 64.050 1.00 0.00 H

ATOM 1996 NZ LYS 121 44.370 30.706 65.294 1.00 0.00 N

ATOM 1997 HZ1 LYS 121 44.936 31.541 65.256 1.00 0.00 H

ATOM 1998 HZ2 LYS 121 43.376 30.857 65.199 1.00 0.00 H

ATOM 1999 HZ3 LYS 121 44.508 30.098 66.088 1.00 0.00 H

ATOM 2000 C LYS 121 44.428 32.882 58.595 1.00 0.00 C

ATOM 2001 O LYS 121 44.626 31.741 58.094 1.00 0.00 O

ATOM 2002 N SER 122 43.892 33.944 57.946 1.00 0.00 N

ATOM 2003 H SER 122 43.741 34.805 58.452 1.00 0.00 H

ATOM 2004 CA SER 122 43.819 34.105 56.484 1.00 0.00 C

ATOM 2005 HA SER 122 44.125 33.214 55.934 1.00 0.00 H

ATOM 2006 CB SER 122 42.453 34.567 56.004 1.00 0.00 C

ATOM 2007 HB2 SER 122 42.180 35.487 56.521 1.00 0.00 H

ATOM 2008 HB3 SER 122 42.421 34.802 54.941 1.00 0.00 H

ATOM 2009 OG SER 122 41.579 33.472 56.284 1.00 0.00 O

ATOM 2010 HG SER 122 41.104 33.629 57.103 1.00 0.00 H

ATOM 2011 C SER 122 44.836 35.123 56.004 1.00 0.00 C

ATOM 2012 O SER 122 45.646 34.843 55.158 1.00 0.00 O

ATOM 2013 N LEU 123 44.946 36.316 56.604 1.00 0.00 N

ATOM 2014 H LEU 123 44.238 36.676 57.228 1.00 0.00 H

ATOM 2015 CA LEU 123 45.882 37.396 55.976 1.00 0.00 C

ATOM 2016 HA LEU 123 45.649 37.439 54.912 1.00 0.00 H

ATOM 2017 CB LEU 123 45.546 38.745 56.555 1.00 0.00 C

ATOM 2018 HB2 LEU 123 44.470 38.883 56.665 1.00 0.00 H

ATOM 2019 HB3 LEU 123 45.926 38.620 57.569 1.00 0.00 H

ATOM 2020 CG LEU 123 46.172 39.947 55.763 1.00 0.00 C

ATOM 2021 HG LEU 123 47.247 39.869 55.596 1.00 0.00 H

ATOM 2022 CD1 LEU 123 45.396 40.243 54.465 1.00 0.00 C

ATOM 2023 HD11 LEU 123 45.582 41.260 54.121 1.00 0.00 H

ATOM 2024 HD12 LEU 123 45.701 39.577 53.657 1.00 0.00 H

ATOM 2025 HD13 LEU 123 44.327 40.142 54.656 1.00 0.00 H

ATOM 2026 CD2 LEU 123 46.007 41.108 56.631 1.00 0.00 C

ATOM 2027 HD21 LEU 123 46.387 41.974 56.088 1.00 0.00 H

ATOM 2028 HD22 LEU 123 44.952 41.355 56.752 1.00 0.00 H

ATOM 2029 HD23 LEU 123 46.489 41.066 57.607 1.00 0.00 H

ATOM 2030 C LEU 123 47.354 37.022 55.956 1.00 0.00 C

ATOM 2031 O LEU 123 48.043 36.963 54.898 1.00 0.00 O

ATOM 2032 N GLY 124 47.897 36.588 57.098 1.00 0.00 N

ATOM 2033 H GLY 124 47.269 36.473 57.880 1.00 0.00 H

ATOM 2034 CA GLY 124 49.263 36.078 57.294 1.00 0.00 C

ATOM 2035 HA2 GLY 124 49.983 36.598 56.662 1.00 0.00 H

ATOM 2036 HA3 GLY 124 49.631 36.511 58.224 1.00 0.00 H

ATOM 2037 C GLY 124 49.402 34.603 57.401 1.00 0.00 C

ATOM 2038 O GLY 124 50.196 34.090 58.167 1.00 0.00 O

ATOM 2039 N ARG 125 48.461 33.926 56.611 1.00 0.00 N

ATOM 2040 H ARG 125 47.663 34.438 56.264 1.00 0.00 H

ATOM 2041 CA ARG 125 48.534 32.525 56.099 1.00 0.00 C

ATOM 2042 HA ARG 125 49.217 31.981 56.751 1.00 0.00 H

ATOM 2043 CB ARG 125 47.139 31.821 56.045 1.00 0.00 C

ATOM 2044 HB2 ARG 125 46.621 32.087 56.966 1.00 0.00 H

ATOM 2045 HB3 ARG 125 46.526 32.165 55.212 1.00 0.00 H

ATOM 2046 CG ARG 125 47.107 30.266 56.024 1.00 0.00 C

ATOM 2047 HG2 ARG 125 46.120 29.890 55.755 1.00 0.00 H

ATOM 2048 HG3 ARG 125 47.721 29.943 55.184 1.00 0.00 H

ATOM 2049 CD ARG 125 47.606 29.543 57.315 1.00 0.00 C

ATOM 2050 HD2 ARG 125 48.679 29.734 57.361 1.00 0.00 H

ATOM 2051 HD3 ARG 125 47.269 29.986 58.252 1.00 0.00 H

ATOM 2052 NE ARG 125 47.114 28.193 57.310 1.00 0.00 N

ATOM 2053 HE ARG 125 46.753 27.787 56.459 1.00 0.00 H

ATOM 2054 CZ ARG 125 46.868 27.463 58.334 1.00 0.00 C

ATOM 2055 NH1 ARG 125 46.982 27.925 59.607 1.00 0.00 N

ATOM 2056 HH11 ARG 125 47.287 28.881 59.727 1.00 0.00 H

ATOM 2057 HH12 ARG 125 47.074 27.357 60.438 1.00 0.00 H

ATOM 2058 NH2 ARG 125 46.247 26.322 58.020 1.00 0.00 N

ATOM 2059 HH21 ARG 125 45.930 25.713 58.761 1.00 0.00 H

ATOM 2060 HH22 ARG 125 45.974 26.176 57.059 1.00 0.00 H

ATOM 2061 C ARG 125 49.250 32.602 54.760 1.00 0.00 C

ATOM 2062 O ARG 125 50.072 31.712 54.457 1.00 0.00 O

ATOM 2063 N LEU 126 49.113 33.663 53.918 1.00 0.00 N

ATOM 2064 H LEU 126 48.515 34.424 54.205 1.00 0.00 H

ATOM 2065 CA LEU 126 49.895 34.028 52.737 1.00 0.00 C

ATOM 2066 HA LEU 126 50.070 33.186 52.068 1.00 0.00 H

ATOM 2067 CB LEU 126 49.232 35.196 51.970 1.00 0.00 C

ATOM 2068 HB2 LEU 126 48.205 34.871 51.803 1.00 0.00 H

ATOM 2069 HB3 LEU 126 49.311 36.085 52.595 1.00 0.00 H

ATOM 2070 CG LEU 126 49.919 35.509 50.608 1.00 0.00 C

ATOM 2071 HG LEU 126 50.996 35.609 50.744 1.00 0.00 H

ATOM 2072 CD1 LEU 126 49.683 34.316 49.607 1.00 0.00 C

ATOM 2073 HD11 LEU 126 50.151 33.480 50.126 1.00 0.00 H

ATOM 2074 HD12 LEU 126 48.608 34.216 49.456 1.00 0.00 H

ATOM 2075 HD13 LEU 126 50.148 34.532 48.645 1.00 0.00 H

ATOM 2076 CD2 LEU 126 49.476 36.774 49.927 1.00 0.00 C

ATOM 2077 HD21 LEU 126 48.450 36.669 49.574 1.00 0.00 H

ATOM 2078 HD22 LEU 126 49.503 37.574 50.666 1.00 0.00 H

ATOM 2079 HD23 LEU 126 50.061 37.212 49.118 1.00 0.00 H

ATOM 2080 C LEU 126 51.370 34.376 53.138 1.00 0.00 C

ATOM 2081 O LEU 126 51.747 35.562 53.404 1.00 0.00 O

ATOM 2082 N LYS 127 52.203 33.373 53.370 1.00 0.00 N

ATOM 2083 H LYS 127 51.643 32.555 53.567 1.00 0.00 H

ATOM 2084 CA LYS 127 53.667 33.471 53.529 1.00 0.00 C

ATOM 2085 HA LYS 127 53.928 34.357 54.108 1.00 0.00 H

ATOM 2086 CB LYS 127 54.061 32.065 54.142 1.00 0.00 C

ATOM 2087 HB2 LYS 127 53.793 31.254 53.465 1.00 0.00 H

ATOM 2088 HB3 LYS 127 55.143 31.954 54.213 1.00 0.00 H

ATOM 2089 CG LYS 127 53.435 31.792 55.580 1.00 0.00 C

ATOM 2090 HG2 LYS 127 52.384 31.546 55.423 1.00 0.00 H

ATOM 2091 HG3 LYS 127 53.867 30.838 55.882 1.00 0.00 H

ATOM 2092 CD LYS 127 53.603 32.937 56.551 1.00 0.00 C

ATOM 2093 HD2 LYS 127 54.652 33.215 56.646 1.00 0.00 H

ATOM 2094 HD3 LYS 127 53.023 33.793 56.205 1.00 0.00 H

ATOM 2095 CE LYS 127 53.025 32.617 57.903 1.00 0.00 C

ATOM 2096 HE2 LYS 127 52.008 32.282 57.698 1.00 0.00 H

ATOM 2097 HE3 LYS 127 53.526 31.774 58.380 1.00 0.00 H

ATOM 2098 NZ LYS 127 53.159 33.781 58.808 1.00 0.00 N

ATOM 2099 HZ1 LYS 127 52.417 33.900 59.483 1.00 0.00 H

ATOM 2100 HZ2 LYS 127 54.004 33.727 59.359 1.00 0.00 H

ATOM 2101 HZ3 LYS 127 53.145 34.640 58.277 1.00 0.00 H

ATOM 2102 C LYS 127 54.381 33.621 52.215 1.00 0.00 C

ATOM 2103 O LYS 127 53.794 33.284 51.177 1.00 0.00 O

ATOM 2104 N LYS 128 55.689 33.939 52.284 1.00 0.00 N

ATOM 2105 H LYS 128 56.025 34.244 53.187 1.00 0.00 H

ATOM 2106 CA LYS 128 56.539 34.206 51.058 1.00 0.00 C

ATOM 2107 HA LYS 128 56.149 35.109 50.589 1.00 0.00 H

ATOM 2108 CB LYS 128 57.979 34.694 51.534 1.00 0.00 C

ATOM 2109 HB2 LYS 128 57.962 35.598 52.143 1.00 0.00 H

ATOM 2110 HB3 LYS 128 58.456 33.913 52.126 1.00 0.00 H

ATOM 2111 CG LYS 128 58.918 34.876 50.319 1.00 0.00 C

ATOM 2112 HG2 LYS 128 59.162 34.030 49.676 1.00 0.00 H

ATOM 2113 HG3 LYS 128 58.447 35.579 49.633 1.00 0.00 H

ATOM 2114 CD LYS 128 60.251 35.513 50.678 1.00 0.00 C

ATOM 2115 HD2 LYS 128 60.531 36.147 49.837 1.00 0.00 H

ATOM 2116 HD3 LYS 128 60.169 35.964 51.667 1.00 0.00 H

ATOM 2117 CE LYS 128 61.311 34.444 50.827 1.00 0.00 C

ATOM 2118 HE2 LYS 128 61.123 33.812 51.694 1.00 0.00 H

ATOM 2119 HE3 LYS 128 61.271 33.930 49.867 1.00 0.00 H

ATOM 2120 NZ LYS 128 62.695 35.016 50.881 1.00 0.00 N

ATOM 2121 HZ1 LYS 128 63.438 34.356 51.061 1.00 0.00 H

ATOM 2122 HZ2 LYS 128 62.933 35.379 49.969 1.00 0.00 H

ATOM 2123 HZ3 LYS 128 62.876 35.693 51.607 1.00 0.00 H

ATOM 2124 C LYS 128 56.636 33.106 49.944 1.00 0.00 C

ATOM 2125 O LYS 128 56.409 33.403 48.770 1.00 0.00 O

ATOM 2126 N LYS 129 56.844 31.818 50.403 1.00 0.00 N

ATOM 2127 H LYS 129 57.386 31.635 51.235 1.00 0.00 H

ATOM 2128 CA LYS 129 56.813 30.639 49.496 1.00 0.00 C

ATOM 2129 HA LYS 129 57.282 30.968 48.569 1.00 0.00 H

ATOM 2130 CB LYS 129 57.666 29.463 50.014 1.00 0.00 C

ATOM 2131 HB2 LYS 129 58.718 29.748 50.012 1.00 0.00 H

ATOM 2132 HB3 LYS 129 57.420 29.301 51.064 1.00 0.00 H

ATOM 2133 CG LYS 129 57.483 28.204 49.181 1.00 0.00 C

ATOM 2134 HG2 LYS 129 56.479 27.844 49.409 1.00 0.00 H

ATOM 2135 HG3 LYS 129 57.439 28.444 48.119 1.00 0.00 H

ATOM 2136 CD LYS 129 58.544 27.090 49.292 1.00 0.00 C

ATOM 2137 HD2 LYS 129 58.585 26.499 48.378 1.00 0.00 H

ATOM 2138 HD3 LYS 129 59.495 27.588 49.477 1.00 0.00 H

ATOM 2139 CE LYS 129 58.295 26.166 50.528 1.00 0.00 C

ATOM 2140 HE2 LYS 129 58.394 26.724 51.459 1.00 0.00 H

ATOM 2141 HE3 LYS 129 57.262 25.819 50.484 1.00 0.00 H

ATOM 2142 NZ LYS 129 59.326 25.157 50.569 1.00 0.00 N

ATOM 2143 HZ1 LYS 129 59.930 25.168 49.760 1.00 0.00 H

ATOM 2144 HZ2 LYS 129 59.854 25.146 51.430 1.00 0.00 H

ATOM 2145 HZ3 LYS 129 58.937 24.226 50.533 1.00 0.00 H

ATOM 2146 C LYS 129 55.404 30.298 49.200 1.00 0.00 C

ATOM 2147 O LYS 129 55.134 30.058 48.063 1.00 0.00 O

ATOM 2148 N ASN 130 54.440 30.230 50.127 1.00 0.00 N

ATOM 2149 H ASN 130 54.733 30.444 51.070 1.00 0.00 H

ATOM 2150 CA ASN 130 53.037 30.014 49.659 1.00 0.00 C

ATOM 2151 HA ASN 130 52.917 29.033 49.198 1.00 0.00 H

ATOM 2152 CB ASN 130 52.180 30.143 50.928 1.00 0.00 C

ATOM 2153 HB2 ASN 130 52.491 29.563 51.796 1.00 0.00 H

ATOM 2154 HB3 ASN 130 52.371 31.126 51.358 1.00 0.00 H

ATOM 2155 CG ASN 130 50.692 30.024 50.645 1.00 0.00 C

ATOM 2156 OD1 ASN 130 49.859 30.734 51.071 1.00 0.00 O

ATOM 2157 ND2 ASN 130 50.266 28.952 50.038 1.00 0.00 N

ATOM 2158 HD21 ASN 130 51.006 28.366 49.678 1.00 0.00 H

ATOM 2159 HD22 ASN 130 49.270 28.856 50.171 1.00 0.00 H

ATOM 2160 C ASN 130 52.619 30.962 48.490 1.00 0.00 C

ATOM 2161 O ASN 130 52.131 30.565 47.493 1.00 0.00 O

ATOM 2162 N ALA 131 52.995 32.260 48.513 1.00 0.00 N

ATOM 2163 H ALA 131 53.263 32.567 49.438 1.00 0.00 H

ATOM 2164 CA ALA 131 52.840 33.313 47.437 1.00 0.00 C

ATOM 2165 HA ALA 131 51.791 33.267 47.144 1.00 0.00 H

ATOM 2166 CB ALA 131 53.073 34.713 48.111 1.00 0.00 C

ATOM 2167 HB1 ALA 131 53.867 34.658 48.856 1.00 0.00 H

ATOM 2168 HB2 ALA 131 53.238 35.462 47.337 1.00 0.00 H

ATOM 2169 HB3 ALA 131 52.152 34.983 48.629 1.00 0.00 H

ATOM 2170 C ALA 131 53.672 33.112 46.170 1.00 0.00 C

ATOM 2171 O ALA 131 53.128 33.254 45.060 1.00 0.00 O

ATOM 2172 N ILE 132 54.888 32.683 46.296 1.00 0.00 N

ATOM 2173 H ILE 132 55.407 32.978 47.111 1.00 0.00 H

ATOM 2174 CA ILE 132 55.591 32.165 45.071 1.00 0.00 C

ATOM 2175 HA ILE 132 55.484 32.961 44.335 1.00 0.00 H

ATOM 2176 CB ILE 132 57.062 31.854 45.463 1.00 0.00 C

ATOM 2177 HB ILE 132 57.169 31.418 46.457 1.00 0.00 H

ATOM 2178 CG2 ILE 132 57.646 30.782 44.511 1.00 0.00 C

ATOM 2179 HG21 ILE 132 57.168 29.803 44.536 1.00 0.00 H

ATOM 2180 HG22 ILE 132 57.647 31.313 43.559 1.00 0.00 H

ATOM 2181 HG23 ILE 132 58.679 30.675 44.844 1.00 0.00 H

ATOM 2182 CG1 ILE 132 57.775 33.244 45.399 1.00 0.00 C

ATOM 2183 HG12 ILE 132 57.843 33.544 44.354 1.00 0.00 H

ATOM 2184 HG13 ILE 132 57.107 33.920 45.933 1.00 0.00 H

ATOM 2185 CD1 ILE 132 59.212 33.086 45.814 1.00 0.00 C

ATOM 2186 HD11 ILE 132 59.914 32.834 45.019 1.00 0.00 H

ATOM 2187 HD12 ILE 132 59.556 34.084 46.089 1.00 0.00 H

ATOM 2188 HD13 ILE 132 59.256 32.405 46.663 1.00 0.00 H

ATOM 2189 C ILE 132 54.845 30.961 44.508 1.00 0.00 C

ATOM 2190 O ILE 132 54.486 31.015 43.348 1.00 0.00 O

ATOM 2191 N CYX 133 54.413 29.993 45.271 1.00 0.00 N

ATOM 2192 H CYX 133 54.622 30.011 46.259 1.00 0.00 H

ATOM 2193 CA CYX 133 53.728 28.829 44.661 1.00 0.00 C

ATOM 2194 HA CYX 133 54.313 28.251 43.946 1.00 0.00 H

ATOM 2195 CB CYX 133 53.406 27.861 45.774 1.00 0.00 C

ATOM 2196 HB2 CYX 133 52.713 28.370 46.443 1.00 0.00 H

ATOM 2197 HB3 CYX 133 53.020 26.903 45.424 1.00 0.00 H

ATOM 2198 SG CYX 133 54.681 27.175 46.794 1.00 0.00 S

ATOM 2199 C CYX 133 52.339 29.206 44.024 1.00 0.00 C

ATOM 2200 O CYX 133 51.968 28.788 42.957 1.00 0.00 O

ATOM 2201 N ILE 134 51.631 30.125 44.697 1.00 0.00 N

ATOM 2202 H ILE 134 51.926 30.338 45.639 1.00 0.00 H

ATOM 2203 CA ILE 134 50.443 30.792 44.054 1.00 0.00 C

ATOM 2204 HA ILE 134 49.730 30.034 43.731 1.00 0.00 H

ATOM 2205 CB ILE 134 49.764 31.760 45.078 1.00 0.00 C

ATOM 2206 HB ILE 134 50.460 32.497 45.479 1.00 0.00 H

ATOM 2207 CG2 ILE 134 48.670 32.577 44.417 1.00 0.00 C

ATOM 2208 HG21 ILE 134 48.355 33.383 45.080 1.00 0.00 H

ATOM 2209 HG22 ILE 134 49.003 33.164 43.561 1.00 0.00 H

ATOM 2210 HG23 ILE 134 47.779 31.971 44.255 1.00 0.00 H

ATOM 2211 CG1 ILE 134 49.118 31.039 46.358 1.00 0.00 C

ATOM 2212 HG12 ILE 134 48.261 30.441 46.048 1.00 0.00 H

ATOM 2213 HG13 ILE 134 49.795 30.291 46.770 1.00 0.00 H

ATOM 2214 CD1 ILE 134 48.820 31.899 47.568 1.00 0.00 C

ATOM 2215 HD11 ILE 134 48.146 31.446 48.295 1.00 0.00 H

ATOM 2216 HD12 ILE 134 49.787 32.053 48.048 1.00 0.00 H

ATOM 2217 HD13 ILE 134 48.310 32.813 47.267 1.00 0.00 H

ATOM 2218 C ILE 134 50.860 31.471 42.668 1.00 0.00 C

ATOM 2219 O ILE 134 50.133 31.257 41.640 1.00 0.00 O

ATOM 2220 N SER 135 51.974 32.096 42.646 1.00 0.00 N

ATOM 2221 H SER 135 52.526 31.878 43.463 1.00 0.00 H

ATOM 2222 CA SER 135 52.467 32.935 41.484 1.00 0.00 C

ATOM 2223 HA SER 135 51.563 33.460 41.176 1.00 0.00 H

ATOM 2224 CB SER 135 53.559 34.021 41.830 1.00 0.00 C

ATOM 2225 HB2 SER 135 54.489 33.579 42.187 1.00 0.00 H

ATOM 2226 HB3 SER 135 53.693 34.695 40.984 1.00 0.00 H

ATOM 2227 OG SER 135 52.955 34.758 42.874 1.00 0.00 O

ATOM 2228 HG SER 135 53.089 34.253 43.679 1.00 0.00 H

ATOM 2229 C SER 135 52.926 31.948 40.410 1.00 0.00 C

ATOM 2230 O SER 135 52.433 32.127 39.312 1.00 0.00 O

ATOM 2231 N VAL 136 53.630 30.857 40.728 1.00 0.00 N

ATOM 2232 H VAL 136 54.188 30.855 41.569 1.00 0.00 H

ATOM 2233 CA VAL 136 53.963 29.792 39.769 1.00 0.00 C

ATOM 2234 HA VAL 136 54.305 30.289 38.861 1.00 0.00 H

ATOM 2235 CB VAL 136 55.069 28.823 40.311 1.00 0.00 C

ATOM 2236 HB VAL 136 54.895 28.680 41.377 1.00 0.00 H

ATOM 2237 CG1 VAL 136 55.221 27.459 39.655 1.00 0.00 C

ATOM 2238 HG11 VAL 136 55.696 26.693 40.267 1.00 0.00 H

ATOM 2239 HG12 VAL 136 54.228 27.144 39.332 1.00 0.00 H

ATOM 2240 HG13 VAL 136 55.733 27.678 38.718 1.00 0.00 H

ATOM 2241 CG2 VAL 136 56.404 29.545 40.181 1.00 0.00 C

ATOM 2242 HG21 VAL 136 56.308 30.549 40.593 1.00 0.00 H

ATOM 2243 HG22 VAL 136 57.157 28.942 40.690 1.00 0.00 H

ATOM 2244 HG23 VAL 136 56.757 29.681 39.158 1.00 0.00 H

ATOM 2245 C VAL 136 52.742 29.012 39.239 1.00 0.00 C

ATOM 2246 O VAL 136 52.708 28.854 38.018 1.00 0.00 O

ATOM 2247 N LEU 137 51.713 28.789 40.090 1.00 0.00 N

ATOM 2248 H LEU 137 51.816 29.093 41.047 1.00 0.00 H

ATOM 2249 CA LEU 137 50.389 28.429 39.691 1.00 0.00 C

ATOM 2250 HA LEU 137 50.530 27.436 39.266 1.00 0.00 H

ATOM 2251 CB LEU 137 49.459 28.050 40.830 1.00 0.00 C

ATOM 2252 HB2 LEU 137 49.965 27.209 41.304 1.00 0.00 H

ATOM 2253 HB3 LEU 137 49.283 28.908 41.478 1.00 0.00 H

ATOM 2254 CG LEU 137 47.981 27.550 40.462 1.00 0.00 C

ATOM 2255 HG LEU 137 47.399 28.311 39.942 1.00 0.00 H

ATOM 2256 CD1 LEU 137 48.079 26.267 39.560 1.00 0.00 C

ATOM 2257 HD11 LEU 137 47.114 25.830 39.302 1.00 0.00 H

ATOM 2258 HD12 LEU 137 48.656 26.427 38.649 1.00 0.00 H

ATOM 2259 HD13 LEU 137 48.660 25.455 39.997 1.00 0.00 H

ATOM 2260 CD2 LEU 137 47.316 27.133 41.853 1.00 0.00 C

ATOM 2261 HD21 LEU 137 47.893 26.376 42.385 1.00 0.00 H

ATOM 2262 HD22 LEU 137 47.183 28.036 42.449 1.00 0.00 H

ATOM 2263 HD23 LEU 137 46.355 26.667 41.638 1.00 0.00 H

ATOM 2264 C LEU 137 49.692 29.313 38.631 1.00 0.00 C

ATOM 2265 O LEU 137 49.516 28.880 37.552 1.00 0.00 O

ATOM 2266 N VAL 138 49.470 30.625 38.954 1.00 0.00 N

ATOM 2267 H VAL 138 49.577 30.839 39.936 1.00 0.00 H

ATOM 2268 CA VAL 138 48.646 31.455 38.022 1.00 0.00 C

ATOM 2269 HA VAL 138 47.723 30.938 37.759 1.00 0.00 H

ATOM 2270 CB VAL 138 48.216 32.754 38.723 1.00 0.00 C

ATOM 2271 HB VAL 138 47.603 33.284 37.994 1.00 0.00 H

ATOM 2272 CG1 VAL 138 47.342 32.470 39.935 1.00 0.00 C

ATOM 2273 HG11 VAL 138 48.007 31.955 40.629 1.00 0.00 H

ATOM 2274 HG12 VAL 138 46.866 33.370 40.324 1.00 0.00 H

ATOM 2275 HG13 VAL 138 46.525 31.781 39.720 1.00 0.00 H

ATOM 2276 CG2 VAL 138 49.449 33.609 39.060 1.00 0.00 C

ATOM 2277 HG21 VAL 138 49.795 33.902 38.069 1.00 0.00 H

ATOM 2278 HG22 VAL 138 49.028 34.505 39.516 1.00 0.00 H

ATOM 2279 HG23 VAL 138 50.191 33.096 39.671 1.00 0.00 H

ATOM 2280 C VAL 138 49.338 31.698 36.688 1.00 0.00 C

ATOM 2281 O VAL 138 48.773 31.584 35.623 1.00 0.00 O

ATOM 2282 N TRP 139 50.634 31.658 36.725 1.00 0.00 N

ATOM 2283 H TRP 139 51.057 31.764 37.635 1.00 0.00 H

ATOM 2284 CA TRP 139 51.510 31.450 35.552 1.00 0.00 C

ATOM 2285 HA TRP 139 51.192 32.186 34.813 1.00 0.00 H

ATOM 2286 CB TRP 139 52.950 31.690 35.966 1.00 0.00 C

ATOM 2287 HB2 TRP 139 53.234 31.157 36.873 1.00 0.00 H

ATOM 2288 HB3 TRP 139 53.534 31.322 35.122 1.00 0.00 H

ATOM 2289 CG TRP 139 53.532 33.067 36.177 1.00 0.00 C

ATOM 2290 CD1 TRP 139 54.162 33.518 37.299 1.00 0.00 C

ATOM 2291 HD1 TRP 139 54.318 33.039 38.253 1.00 0.00 H

ATOM 2292 NE1 TRP 139 54.732 34.753 37.058 1.00 0.00 N

ATOM 2293 HE1 TRP 139 55.311 35.248 37.721 1.00 0.00 H

ATOM 2294 CE2 TRP 139 54.408 35.203 35.773 1.00 0.00 C

ATOM 2295 CZ2 TRP 139 54.605 36.469 35.167 1.00 0.00 C

ATOM 2296 HZ2 TRP 139 55.215 37.257 35.583 1.00 0.00 H

ATOM 2297 CH2 TRP 139 53.844 36.824 34.002 1.00 0.00 C

ATOM 2298 HH2 TRP 139 54.237 37.652 33.429 1.00 0.00 H

ATOM 2299 CZ3 TRP 139 53.034 35.866 33.359 1.00 0.00 C

ATOM 2300 HZ3 TRP 139 52.399 36.096 32.516 1.00 0.00 H

ATOM 2301 CE3 TRP 139 53.051 34.532 33.904 1.00 0.00 C

ATOM 2302 HE3 TRP 139 52.389 33.811 33.446 1.00 0.00 H

ATOM 2303 CD2 TRP 139 53.606 34.199 35.172 1.00 0.00 C

ATOM 2304 C TRP 139 51.216 30.200 34.706 1.00 0.00 C

ATOM 2305 O TRP 139 50.737 30.398 33.566 1.00 0.00 O

ATOM 2306 N LEU 140 51.345 29.022 35.318 1.00 0.00 N

ATOM 2307 H LEU 140 51.679 29.016 36.271 1.00 0.00 H

ATOM 2308 CA LEU 140 50.885 27.803 34.608 1.00 0.00 C

ATOM 2309 HA LEU 140 51.616 27.663 33.812 1.00 0.00 H

ATOM 2310 CB LEU 140 50.906 26.598 35.575 1.00 0.00 C

ATOM 2311 HB2 LEU 140 51.876 26.400 36.029 1.00 0.00 H

ATOM 2312 HB3 LEU 140 50.231 26.687 36.426 1.00 0.00 H

ATOM 2313 CG LEU 140 50.513 25.308 34.903 1.00 0.00 C

ATOM 2314 HG LEU 140 49.599 25.440 34.323 1.00 0.00 H

ATOM 2315 CD1 LEU 140 51.509 24.693 33.950 1.00 0.00 C

ATOM 2316 HD11 LEU 140 51.622 25.276 33.036 1.00 0.00 H

ATOM 2317 HD12 LEU 140 52.536 24.894 34.254 1.00 0.00 H

ATOM 2318 HD13 LEU 140 51.212 23.672 33.714 1.00 0.00 H

ATOM 2319 CD2 LEU 140 49.973 24.267 35.865 1.00 0.00 C

ATOM 2320 HD21 LEU 140 49.691 23.351 35.346 1.00 0.00 H

ATOM 2321 HD22 LEU 140 50.719 24.000 36.613 1.00 0.00 H

ATOM 2322 HD23 LEU 140 49.048 24.706 36.240 1.00 0.00 H

ATOM 2323 C LEU 140 49.456 27.928 33.990 1.00 0.00 C

ATOM 2324 O LEU 140 49.308 27.595 32.870 1.00 0.00 O

ATOM 2325 N ILE 141 48.492 28.410 34.787 1.00 0.00 N

ATOM 2326 H ILE 141 48.677 28.766 35.714 1.00 0.00 H

ATOM 2327 CA ILE 141 47.170 28.568 34.282 1.00 0.00 C

ATOM 2328 HA ILE 141 46.855 27.581 33.945 1.00 0.00 H

ATOM 2329 CB ILE 141 46.202 28.968 35.412 1.00 0.00 C

ATOM 2330 HB ILE 141 46.428 29.993 35.705 1.00 0.00 H

ATOM 2331 CG2 ILE 141 44.787 28.882 34.907 1.00 0.00 C

ATOM 2332 HG21 ILE 141 44.052 29.065 35.690 1.00 0.00 H

ATOM 2333 HG22 ILE 141 44.606 29.611 34.117 1.00 0.00 H

ATOM 2334 HG23 ILE 141 44.634 27.845 34.608 1.00 0.00 H

ATOM 2335 CG1 ILE 141 46.319 28.016 36.654 1.00 0.00 C

ATOM 2336 HG12 ILE 141 46.476 26.966 36.404 1.00 0.00 H

ATOM 2337 HG13 ILE 141 47.307 28.245 37.055 1.00 0.00 H

ATOM 2338 CD1 ILE 141 45.376 28.095 37.852 1.00 0.00 C

ATOM 2339 HD11 ILE 141 45.387 29.040 38.396 1.00 0.00 H

ATOM 2340 HD12 ILE 141 44.369 28.010 37.443 1.00 0.00 H

ATOM 2341 HD13 ILE 141 45.596 27.310 38.575 1.00 0.00 H

ATOM 2342 C ILE 141 46.999 29.400 33.049 1.00 0.00 C

ATOM 2343 O ILE 141 46.185 29.066 32.189 1.00 0.00 O

ATOM 2344 N VAL 142 47.892 30.389 32.822 1.00 0.00 N

ATOM 2345 H VAL 142 48.664 30.469 33.468 1.00 0.00 H

ATOM 2346 CA VAL 142 47.864 31.174 31.561 1.00 0.00 C

ATOM 2347 HA VAL 142 46.818 31.289 31.279 1.00 0.00 H

ATOM 2348 CB VAL 142 48.528 32.518 31.855 1.00 0.00 C

ATOM 2349 HB VAL 142 49.319 32.455 32.603 1.00 0.00 H

ATOM 2350 CG1 VAL 142 49.242 33.181 30.612 1.00 0.00 C

ATOM 2351 HG11 VAL 142 50.192 32.684 30.417 1.00 0.00 H

ATOM 2352 HG12 VAL 142 48.512 33.140 29.804 1.00 0.00 H

ATOM 2353 HG13 VAL 142 49.498 34.237 30.699 1.00 0.00 H

ATOM 2354 CG2 VAL 142 47.487 33.506 32.354 1.00 0.00 C

ATOM 2355 HG21 VAL 142 46.648 33.666 31.677 1.00 0.00 H

ATOM 2356 HG22 VAL 142 47.111 33.122 33.302 1.00 0.00 H

ATOM 2357 HG23 VAL 142 47.839 34.497 32.641 1.00 0.00 H

ATOM 2358 C VAL 142 48.504 30.444 30.362 1.00 0.00 C

ATOM 2359 O VAL 142 47.838 30.158 29.368 1.00 0.00 O

ATOM 2360 N VAL 143 49.614 29.768 30.650 1.00 0.00 N

ATOM 2361 H VAL 143 49.918 29.806 31.612 1.00 0.00 H

ATOM 2362 CA VAL 143 50.458 29.175 29.608 1.00 0.00 C

ATOM 2363 HA VAL 143 50.349 29.840 28.751 1.00 0.00 H

ATOM 2364 CB VAL 143 51.959 29.100 29.960 1.00 0.00 C

ATOM 2365 HB VAL 143 52.532 28.748 29.102 1.00 0.00 H

ATOM 2366 CG1 VAL 143 52.412 30.574 30.233 1.00 0.00 C

ATOM 2367 HG11 VAL 143 52.239 31.154 29.326 1.00 0.00 H

ATOM 2368 HG12 VAL 143 51.927 31.000 31.111 1.00 0.00 H

ATOM 2369 HG13 VAL 143 53.452 30.687 30.540 1.00 0.00 H

ATOM 2370 CG2 VAL 143 52.356 28.238 31.128 1.00 0.00 C

ATOM 2371 HG21 VAL 143 51.720 27.361 31.248 1.00 0.00 H

ATOM 2372 HG22 VAL 143 53.436 28.137 31.018 1.00 0.00 H

ATOM 2373 HG23 VAL 143 52.271 28.823 32.044 1.00 0.00 H

ATOM 2374 C VAL 143 49.836 27.817 29.130 1.00 0.00 C

ATOM 2375 O VAL 143 50.252 27.400 28.050 1.00 0.00 O

ATOM 2376 N VAL 144 48.958 27.136 29.845 1.00 0.00 N

ATOM 2377 H VAL 144 48.935 27.496 30.789 1.00 0.00 H

ATOM 2378 CA VAL 144 48.086 26.077 29.340 1.00 0.00 C

ATOM 2379 HA VAL 144 48.604 25.652 28.481 1.00 0.00 H

ATOM 2380 CB VAL 144 47.875 24.939 30.357 1.00 0.00 C

ATOM 2381 HB VAL 144 47.549 24.117 29.719 1.00 0.00 H

ATOM 2382 CG1 VAL 144 49.178 24.528 31.050 1.00 0.00 C

ATOM 2383 HG11 VAL 144 49.263 23.446 31.151 1.00 0.00 H

ATOM 2384 HG12 VAL 144 50.084 24.797 30.508 1.00 0.00 H

ATOM 2385 HG13 VAL 144 49.156 25.025 32.020 1.00 0.00 H

ATOM 2386 CG2 VAL 144 46.850 25.202 31.441 1.00 0.00 C

ATOM 2387 HG21 VAL 144 46.901 26.152 31.973 1.00 0.00 H

ATOM 2388 HG22 VAL 144 45.890 25.235 30.926 1.00 0.00 H

ATOM 2389 HG23 VAL 144 46.731 24.331 32.086 1.00 0.00 H

ATOM 2390 C VAL 144 46.822 26.583 28.741 1.00 0.00 C

ATOM 2391 O VAL 144 46.550 26.025 27.674 1.00 0.00 O

ATOM 2392 N ALA 145 46.277 27.736 29.218 1.00 0.00 N

ATOM 2393 H ALA 145 46.733 28.174 30.005 1.00 0.00 H

ATOM 2394 CA ALA 145 44.975 28.160 28.699 1.00 0.00 C

ATOM 2395 HA ALA 145 44.171 27.424 28.696 1.00 0.00 H

ATOM 2396 CB ALA 145 44.417 29.077 29.722 1.00 0.00 C

ATOM 2397 HB1 ALA 145 45.133 29.883 29.883 1.00 0.00 H

ATOM 2398 HB2 ALA 145 43.426 29.345 29.355 1.00 0.00 H

ATOM 2399 HB3 ALA 145 44.292 28.598 30.693 1.00 0.00 H

ATOM 2400 C ALA 145 44.933 28.725 27.349 1.00 0.00 C

ATOM 2401 O ALA 145 44.062 28.370 26.561 1.00 0.00 O

ATOM 2402 N ILE 146 45.920 29.581 27.018 1.00 0.00 N

ATOM 2403 H ILE 146 46.583 29.768 27.756 1.00 0.00 H

ATOM 2404 CA ILE 146 45.896 30.367 25.780 1.00 0.00 C

ATOM 2405 HA ILE 146 44.860 30.670 25.628 1.00 0.00 H

ATOM 2406 CB ILE 146 46.660 31.661 25.947 1.00 0.00 C

ATOM 2407 HB ILE 146 46.605 32.271 25.046 1.00 0.00 H

ATOM 2408 CG2 ILE 146 46.004 32.530 27.012 1.00 0.00 C

ATOM 2409 HG21 ILE 146 44.984 32.211 27.225 1.00 0.00 H

ATOM 2410 HG22 ILE 146 46.539 32.366 27.947 1.00 0.00 H

ATOM 2411 HG23 ILE 146 45.980 33.563 26.663 1.00 0.00 H

ATOM 2412 CG1 ILE 146 48.177 31.457 26.321 1.00 0.00 C

ATOM 2413 HG12 ILE 146 48.424 30.741 27.105 1.00 0.00 H

ATOM 2414 HG13 ILE 146 48.492 30.776 25.530 1.00 0.00 H

ATOM 2415 CD1 ILE 146 49.079 32.707 26.342 1.00 0.00 C

ATOM 2416 HD11 ILE 146 50.037 32.188 26.389 1.00 0.00 H

ATOM 2417 HD12 ILE 146 48.837 33.266 25.438 1.00 0.00 H

ATOM 2418 HD13 ILE 146 48.871 33.298 27.234 1.00 0.00 H

ATOM 2419 C ILE 146 46.373 29.608 24.485 1.00 0.00 C

ATOM 2420 O ILE 146 46.199 30.074 23.382 1.00 0.00 O

ATOM 2421 N SER 147 46.900 28.386 24.628 1.00 0.00 N

ATOM 2422 H SER 147 46.556 27.777 25.357 1.00 0.00 H

ATOM 2423 CA SER 147 47.818 27.880 23.748 1.00 0.00 C

ATOM 2424 HA SER 147 48.438 28.699 23.382 1.00 0.00 H

ATOM 2425 CB SER 147 48.818 27.036 24.502 1.00 0.00 C

ATOM 2426 HB2 SER 147 48.398 26.205 25.069 1.00 0.00 H

ATOM 2427 HB3 SER 147 49.534 26.659 23.772 1.00 0.00 H

ATOM 2428 OG SER 147 49.561 27.854 25.348 1.00 0.00 O

ATOM 2429 HG SER 147 49.721 27.391 26.174 1.00 0.00 H

ATOM 2430 C SER 147 47.202 27.334 22.484 1.00 0.00 C

ATOM 2431 O SER 147 47.906 27.530 21.520 1.00 0.00 O

ATOM 2432 N PRO 148 46.276 26.382 22.378 1.00 0.00 N

ATOM 2433 CD PRO 148 45.495 25.923 23.529 1.00 0.00 C

ATOM 2434 HD2 PRO 148 45.155 26.772 24.123 1.00 0.00 H

ATOM 2435 HD3 PRO 148 46.041 25.182 24.112 1.00 0.00 H

ATOM 2436 CG PRO 148 44.221 25.331 22.934 1.00 0.00 C

ATOM 2437 HG2 PRO 148 43.480 26.081 22.660 1.00 0.00 H

ATOM 2438 HG3 PRO 148 43.803 24.454 23.430 1.00 0.00 H

ATOM 2439 CB PRO 148 44.789 24.738 21.610 1.00 0.00 C

ATOM 2440 HB2 PRO 148 43.989 24.847 20.877 1.00 0.00 H

ATOM 2441 HB3 PRO 148 45.143 23.711 21.702 1.00 0.00 H

ATOM 2442 CA PRO 148 45.979 25.559 21.213 1.00 0.00 C

ATOM 2443 HA PRO 148 46.818 24.875 21.087 1.00 0.00 H

ATOM 2444 C PRO 148 45.833 26.228 19.849 1.00 0.00 C

ATOM 2445 O PRO 148 46.275 25.670 18.844 1.00 0.00 O

ATOM 2446 N ILE 149 45.254 27.430 19.853 1.00 0.00 N

ATOM 2447 H ILE 149 44.811 27.775 20.692 1.00 0.00 H

ATOM 2448 CA ILE 149 44.804 28.059 18.617 1.00 0.00 C

ATOM 2449 HA ILE 149 44.993 27.348 17.813 1.00 0.00 H

ATOM 2450 CB ILE 149 43.280 28.266 18.597 1.00 0.00 C

ATOM 2451 HB ILE 149 42.889 27.993 19.578 1.00 0.00 H

ATOM 2452 CG2 ILE 149 42.901 29.734 18.382 1.00 0.00 C

ATOM 2453 HG21 ILE 149 43.143 30.186 17.420 1.00 0.00 H

ATOM 2454 HG22 ILE 149 41.828 29.703 18.571 1.00 0.00 H

ATOM 2455 HG23 ILE 149 43.249 30.428 19.147 1.00 0.00 H

ATOM 2456 CG1 ILE 149 42.461 27.518 17.550 1.00 0.00 C

ATOM 2457 HG12 ILE 149 41.553 28.080 17.331 1.00 0.00 H

ATOM 2458 HG13 ILE 149 42.893 27.574 16.551 1.00 0.00 H

ATOM 2459 CD1 ILE 149 42.033 26.089 17.907 1.00 0.00 C

ATOM 2460 HD11 ILE 149 41.223 25.768 17.252 1.00 0.00 H

ATOM 2461 HD12 ILE 149 42.851 25.410 17.668 1.00 0.00 H

ATOM 2462 HD13 ILE 149 41.739 26.037 18.956 1.00 0.00 H

ATOM 2463 C ILE 149 45.749 29.250 18.262 1.00 0.00 C

ATOM 2464 O ILE 149 46.142 29.420 17.097 1.00 0.00 O

ATOM 2465 N LEU 150 46.318 29.928 19.287 1.00 0.00 N

ATOM 2466 H LEU 150 46.027 29.668 20.219 1.00 0.00 H

ATOM 2467 CA LEU 150 47.458 30.872 19.169 1.00 0.00 C

ATOM 2468 HA LEU 150 47.063 31.647 18.512 1.00 0.00 H

ATOM 2469 CB LEU 150 47.611 31.293 20.629 1.00 0.00 C

ATOM 2470 HB2 LEU 150 46.608 31.641 20.876 1.00 0.00 H

ATOM 2471 HB3 LEU 150 47.783 30.500 21.357 1.00 0.00 H

ATOM 2472 CG LEU 150 48.597 32.541 20.884 1.00 0.00 C

ATOM 2473 HG LEU 150 48.834 33.083 19.968 1.00 0.00 H

ATOM 2474 CD1 LEU 150 47.905 33.548 21.882 1.00 0.00 C

ATOM 2475 HD11 LEU 150 47.391 33.092 22.729 1.00 0.00 H

ATOM 2476 HD12 LEU 150 48.762 34.089 22.285 1.00 0.00 H

ATOM 2477 HD13 LEU 150 47.190 34.202 21.383 1.00 0.00 H

ATOM 2478 CD2 LEU 150 49.946 31.938 21.341 1.00 0.00 C

ATOM 2479 HD21 LEU 150 50.568 32.821 21.491 1.00 0.00 H

ATOM 2480 HD22 LEU 150 50.005 31.292 22.217 1.00 0.00 H

ATOM 2481 HD23 LEU 150 50.348 31.419 20.470 1.00 0.00 H

ATOM 2482 C LEU 150 48.779 30.334 18.545 1.00 0.00 C

ATOM 2483 O LEU 150 49.240 30.832 17.498 1.00 0.00 O

ATOM 2484 N PHE 151 49.164 29.142 18.899 1.00 0.00 N

ATOM 2485 H PHE 151 48.618 28.683 19.614 1.00 0.00 H

ATOM 2486 CA PHE 151 50.130 28.273 18.209 1.00 0.00 C

ATOM 2487 HA PHE 151 50.909 28.891 17.763 1.00 0.00 H

ATOM 2488 CB PHE 151 50.725 27.417 19.363 1.00 0.00 C

ATOM 2489 HB2 PHE 151 49.874 26.999 19.902 1.00 0.00 H

ATOM 2490 HB3 PHE 151 51.274 26.605 18.886 1.00 0.00 H

ATOM 2491 CG PHE 151 51.592 28.183 20.359 1.00 0.00 C

ATOM 2492 CD1 PHE 151 51.315 28.332 21.703 1.00 0.00 C

ATOM 2493 HD1 PHE 151 50.311 28.231 22.087 1.00 0.00 H

ATOM 2494 CE1 PHE 151 52.201 28.996 22.449 1.00 0.00 C

ATOM 2495 HE1 PHE 151 51.908 29.185 23.471 1.00 0.00 H

ATOM 2496 CZ PHE 151 53.385 29.560 22.010 1.00 0.00 C

ATOM 2497 HZ PHE 151 54.029 30.014 22.749 1.00 0.00 H

ATOM 2498 CE2 PHE 151 53.652 29.467 20.591 1.00 0.00 C

ATOM 2499 HE2 PHE 151 54.669 29.631 20.266 1.00 0.00 H

ATOM 2500 CD2 PHE 151 52.746 28.696 19.818 1.00 0.00 C

ATOM 2501 HD2 PHE 151 53.071 28.535 18.801 1.00 0.00 H

ATOM 2502 C PHE 151 49.481 27.551 17.009 1.00 0.00 C

ATOM 2503 O PHE 151 49.865 26.349 16.750 1.00 0.00 O

ATOM 2504 N TYR 152 48.813 28.220 16.127 1.00 0.00 N

ATOM 2505 H TYR 152 48.612 29.186 16.344 1.00 0.00 H

ATOM 2506 CA TYR 152 48.663 27.872 14.704 1.00 0.00 C

ATOM 2507 HA TYR 152 49.559 27.455 14.243 1.00 0.00 H

ATOM 2508 CB TYR 152 47.591 26.804 14.475 1.00 0.00 C

ATOM 2509 HB2 TYR 152 48.016 25.878 14.864 1.00 0.00 H

ATOM 2510 HB3 TYR 152 46.720 27.066 15.075 1.00 0.00 H

ATOM 2511 CG TYR 152 47.068 26.720 13.048 1.00 0.00 C

ATOM 2512 CD1 TYR 152 45.701 27.053 12.779 1.00 0.00 C

ATOM 2513 HD1 TYR 152 44.982 27.168 13.577 1.00 0.00 H

ATOM 2514 CE1 TYR 152 45.208 27.166 11.473 1.00 0.00 C

ATOM 2515 HE1 TYR 152 44.171 27.322 11.213 1.00 0.00 H

ATOM 2516 CZ TYR 152 46.070 26.799 10.477 1.00 0.00 C

ATOM 2517 OH TYR 152 45.694 26.810 9.152 1.00 0.00 O

ATOM 2518 HH TYR 152 44.742 26.841 9.040 1.00 0.00 H

ATOM 2519 CE2 TYR 152 47.389 26.415 10.623 1.00 0.00 C

ATOM 2520 HE2 TYR 152 48.032 25.987 9.869 1.00 0.00 H

ATOM 2521 CD2 TYR 152 47.871 26.431 11.924 1.00 0.00 C

ATOM 2522 HD2 TYR 152 48.927 26.235 12.041 1.00 0.00 H

ATOM 2523 C TYR 152 48.303 29.175 13.931 1.00 0.00 C

ATOM 2524 O TYR 152 48.967 29.475 12.968 1.00 0.00 O

ATOM 2525 N SER 153 47.387 29.996 14.460 1.00 0.00 N

ATOM 2526 H SER 153 47.166 29.877 15.438 1.00 0.00 H

ATOM 2527 CA SER 153 46.833 31.236 13.975 1.00 0.00 C

ATOM 2528 HA SER 153 46.640 31.110 12.910 1.00 0.00 H

ATOM 2529 CB SER 153 45.479 31.494 14.528 1.00 0.00 C

ATOM 2530 HB2 SER 153 44.872 30.591 14.466 1.00 0.00 H

ATOM 2531 HB3 SER 153 45.687 31.606 15.592 1.00 0.00 H

ATOM 2532 OG SER 153 44.791 32.529 13.894 1.00 0.00 O

ATOM 2533 HG SER 153 44.533 33.260 14.459 1.00 0.00 H

ATOM 2534 C SER 153 47.784 32.507 14.106 1.00 0.00 C

ATOM 2535 O SER 153 48.867 32.377 14.469 1.00 0.00 O

ATOM 2536 N GLY 154 47.460 33.656 13.428 1.00 0.00 N

ATOM 2537 H GLY 154 46.523 33.705 13.055 1.00 0.00 H

ATOM 2538 CA GLY 154 48.220 34.944 13.566 1.00 0.00 C

ATOM 2539 HA2 GLY 154 47.468 35.732 13.550 1.00 0.00 H

ATOM 2540 HA3 GLY 154 48.734 35.146 14.505 1.00 0.00 H

ATOM 2541 C GLY 154 49.218 35.366 12.487 1.00 0.00 C

ATOM 2542 O GLY 154 49.813 36.414 12.517 1.00 0.00 O

ATOM 2543 N THR 155 49.309 34.534 11.425 1.00 0.00 N

ATOM 2544 H THR 155 48.584 33.833 11.372 1.00 0.00 H

ATOM 2545 CA THR 155 50.263 34.644 10.228 1.00 0.00 C

ATOM 2546 HA THR 155 50.684 35.643 10.345 1.00 0.00 H

ATOM 2547 CB THR 155 51.459 33.693 10.414 1.00 0.00 C

ATOM 2548 HB THR 155 52.095 33.641 9.530 1.00 0.00 H

ATOM 2549 CG2 THR 155 52.388 34.135 11.535 1.00 0.00 C

ATOM 2550 HG21 THR 155 53.404 33.759 11.410 1.00 0.00 H

ATOM 2551 HG22 THR 155 52.540 35.209 11.423 1.00 0.00 H

ATOM 2552 HG23 THR 155 51.984 33.806 12.492 1.00 0.00 H

ATOM 2553 OG1 THR 155 51.068 32.398 10.690 1.00 0.00 O

ATOM 2554 HG1 THR 155 51.926 31.966 10.665 1.00 0.00 H

ATOM 2555 C THR 155 49.495 34.501 8.901 1.00 0.00 C

ATOM 2556 O THR 155 48.379 33.971 8.803 1.00 0.00 O

ATOM 2557 N GLY 156 50.062 35.028 7.866 1.00 0.00 N

ATOM 2558 H GLY 156 50.947 35.494 8.007 1.00 0.00 H

ATOM 2559 CA GLY 156 49.583 34.845 6.511 1.00 0.00 C

ATOM 2560 HA2 GLY 156 48.954 33.968 6.360 1.00 0.00 H

ATOM 2561 HA3 GLY 156 48.888 35.660 6.309 1.00 0.00 H

ATOM 2562 C GLY 156 50.737 34.861 5.440 1.00 0.00 C

ATOM 2563 O GLY 156 51.914 35.021 5.761 1.00 0.00 O

ATOM 2564 N VAL 157 50.396 34.783 4.195 1.00 0.00 N

ATOM 2565 H VAL 157 49.402 34.777 4.017 1.00 0.00 H

ATOM 2566 CA VAL 157 51.356 34.683 3.020 1.00 0.00 C

ATOM 2567 HA VAL 157 52.335 34.540 3.478 1.00 0.00 H

ATOM 2568 CB VAL 157 50.789 33.492 2.132 1.00 0.00 C

ATOM 2569 HB VAL 157 49.773 33.745 1.831 1.00 0.00 H

ATOM 2570 CG1 VAL 157 51.565 33.392 0.862 1.00 0.00 C

ATOM 2571 HG11 VAL 157 51.492 32.394 0.429 1.00 0.00 H

ATOM 2572 HG12 VAL 157 51.138 34.085 0.137 1.00 0.00 H

ATOM 2573 HG13 VAL 157 52.613 33.643 1.024 1.00 0.00 H

ATOM 2574 CG2 VAL 157 50.694 32.154 2.939 1.00 0.00 C

ATOM 2575 HG21 VAL 157 50.535 31.380 2.188 1.00 0.00 H

ATOM 2576 HG22 VAL 157 51.693 31.979 3.338 1.00 0.00 H

ATOM 2577 HG23 VAL 157 49.899 32.056 3.678 1.00 0.00 H

ATOM 2578 C VAL 157 51.485 36.124 2.306 1.00 0.00 C

ATOM 2579 O VAL 157 50.558 36.898 2.062 1.00 0.00 O

ATOM 2580 N ARG 158 52.662 36.530 1.935 1.00 0.00 N

ATOM 2581 H ARG 158 53.341 35.798 2.087 1.00 0.00 H

ATOM 2582 CA ARG 158 53.039 37.543 0.952 1.00 0.00 C

ATOM 2583 HA ARG 158 52.306 38.347 0.891 1.00 0.00 H

ATOM 2584 CB ARG 158 54.362 38.118 1.331 1.00 0.00 C

ATOM 2585 HB2 ARG 158 55.126 37.397 1.039 1.00 0.00 H

ATOM 2586 HB3 ARG 158 54.468 39.000 0.698 1.00 0.00 H

ATOM 2587 CG ARG 158 54.655 38.776 2.670 1.00 0.00 C

ATOM 2588 HG2 ARG 158 53.795 39.440 2.761 1.00 0.00 H

ATOM 2589 HG3 ARG 158 54.949 38.039 3.417 1.00 0.00 H

ATOM 2590 CD ARG 158 55.875 39.613 2.550 1.00 0.00 C

ATOM 2591 HD2 ARG 158 56.742 39.150 2.079 1.00 0.00 H

ATOM 2592 HD3 ARG 158 55.666 40.478 1.921 1.00 0.00 H

ATOM 2593 NE ARG 158 56.215 40.099 3.942 1.00 0.00 N

ATOM 2594 HE ARG 158 55.967 41.032 4.240 1.00 0.00 H

ATOM 2595 CZ ARG 158 57.064 39.536 4.772 1.00 0.00 C

ATOM 2596 NH1 ARG 158 57.713 38.470 4.490 1.00 0.00 N

ATOM 2597 HH11 ARG 158 57.668 38.060 3.568 1.00 0.00 H

ATOM 2598 HH12 ARG 158 58.141 37.951 5.244 1.00 0.00 H

ATOM 2599 NH2 ARG 158 57.223 39.977 5.925 1.00 0.00 N

ATOM 2600 HH21 ARG 158 57.204 40.970 6.109 1.00 0.00 H

ATOM 2601 HH22 ARG 158 57.826 39.484 6.568 1.00 0.00 H

ATOM 2602 C ARG 158 53.024 36.994 -0.451 1.00 0.00 C

ATOM 2603 O ARG 158 53.422 35.852 -0.685 1.00 0.00 O

ATOM 2604 N LYS 159 52.779 37.854 -1.479 1.00 0.00 N

ATOM 2605 H LYS 159 52.320 38.698 -1.166 1.00 0.00 H

ATOM 2606 CA LYS 159 52.801 37.463 -2.945 1.00 0.00 C

ATOM 2607 HA LYS 159 52.000 36.729 -3.032 1.00 0.00 H

ATOM 2608 CB LYS 159 52.496 38.734 -3.827 1.00 0.00 C

ATOM 2609 HB2 LYS 159 52.180 38.417 -4.821 1.00 0.00 H

ATOM 2610 HB3 LYS 159 51.699 39.347 -3.406 1.00 0.00 H

ATOM 2611 CG LYS 159 53.694 39.701 -3.969 1.00 0.00 C

ATOM 2612 HG2 LYS 159 54.192 39.974 -3.038 1.00 0.00 H

ATOM 2613 HG3 LYS 159 54.378 39.123 -4.590 1.00 0.00 H

ATOM 2614 CD LYS 159 53.230 40.960 -4.754 1.00 0.00 C

ATOM 2615 HD2 LYS 159 54.044 41.625 -5.043 1.00 0.00 H

ATOM 2616 HD3 LYS 159 52.735 40.682 -5.684 1.00 0.00 H

ATOM 2617 CE LYS 159 52.189 41.845 -3.955 1.00 0.00 C

ATOM 2618 HE2 LYS 159 51.311 41.202 -3.899 1.00 0.00 H

ATOM 2619 HE3 LYS 159 52.583 42.028 -2.955 1.00 0.00 H

ATOM 2620 NZ LYS 159 51.833 43.090 -4.669 1.00 0.00 N

ATOM 2621 HZ1 LYS 159 52.681 43.620 -4.814 1.00 0.00 H

ATOM 2622 HZ2 LYS 159 51.371 42.925 -5.552 1.00 0.00 H

ATOM 2623 HZ3 LYS 159 51.317 43.773 -4.134 1.00 0.00 H

ATOM 2624 C LYS 159 54.048 36.752 -3.444 1.00 0.00 C

ATOM 2625 O LYS 159 53.815 35.957 -4.340 1.00 0.00 O

ATOM 2626 N ASN 160 55.254 36.866 -2.874 1.00 0.00 N

ATOM 2627 H ASN 160 55.463 37.710 -2.359 1.00 0.00 H

ATOM 2628 CA ASN 160 56.463 36.055 -3.264 1.00 0.00 C

ATOM 2629 HA ASN 160 56.397 35.920 -4.343 1.00 0.00 H

ATOM 2630 CB ASN 160 57.699 36.956 -3.072 1.00 0.00 C

ATOM 2631 HB2 ASN 160 58.561 36.490 -3.548 1.00 0.00 H

ATOM 2632 HB3 ASN 160 57.455 37.896 -3.566 1.00 0.00 H

ATOM 2633 CG ASN 160 57.927 37.422 -1.629 1.00 0.00 C

ATOM 2634 OD1 ASN 160 57.073 37.174 -0.777 1.00 0.00 O

ATOM 2635 ND2 ASN 160 59.060 38.058 -1.267 1.00 0.00 N

ATOM 2636 HD21 ASN 160 59.667 38.337 -2.025 1.00 0.00 H

ATOM 2637 HD22 ASN 160 59.110 38.400 -0.318 1.00 0.00 H

ATOM 2638 C ASN 160 56.474 34.617 -2.646 1.00 0.00 C

ATOM 2639 O ASN 160 57.497 33.977 -2.665 1.00 0.00 O

ATOM 2640 N LYS 161 55.345 34.247 -1.974 1.00 0.00 N

ATOM 2641 H LYS 161 54.518 34.824 -2.023 1.00 0.00 H

ATOM 2642 CA LYS 161 55.282 33.041 -1.046 1.00 0.00 C

ATOM 2643 HA LYS 161 54.334 33.054 -0.508 1.00 0.00 H

ATOM 2644 CB LYS 161 55.206 31.765 -1.923 1.00 0.00 C

ATOM 2645 HB2 LYS 161 56.009 31.737 -2.659 1.00 0.00 H

ATOM 2646 HB3 LYS 161 55.362 30.919 -1.253 1.00 0.00 H

ATOM 2647 CG LYS 161 53.864 31.447 -2.562 1.00 0.00 C

ATOM 2648 HG2 LYS 161 53.892 30.544 -3.172 1.00 0.00 H

ATOM 2649 HG3 LYS 161 53.147 31.155 -1.796 1.00 0.00 H

ATOM 2650 CD LYS 161 53.223 32.527 -3.521 1.00 0.00 C

ATOM 2651 HD2 LYS 161 52.813 33.367 -2.961 1.00 0.00 H

ATOM 2652 HD3 LYS 161 54.021 32.960 -4.124 1.00 0.00 H

ATOM 2653 CE LYS 161 52.149 32.125 -4.467 1.00 0.00 C

ATOM 2654 HE2 LYS 161 51.620 33.005 -4.834 1.00 0.00 H

ATOM 2655 HE3 LYS 161 52.506 31.671 -5.392 1.00 0.00 H

ATOM 2656 NZ LYS 161 51.051 31.269 -3.920 1.00 0.00 N

ATOM 2657 HZ1 LYS 161 51.515 30.448 -3.560 1.00 0.00 H

ATOM 2658 HZ2 LYS 161 50.697 31.718 -3.087 1.00 0.00 H

ATOM 2659 HZ3 LYS 161 50.328 30.875 -4.505 1.00 0.00 H

ATOM 2660 C LYS 161 56.412 33.033 0.004 1.00 0.00 C

ATOM 2661 O LYS 161 57.106 32.028 0.245 1.00 0.00 O

ATOM 2662 N THR 162 56.640 34.191 0.532 1.00 0.00 N

ATOM 2663 H THR 162 56.139 35.002 0.197 1.00 0.00 H

ATOM 2664 CA THR 162 57.111 34.330 1.919 1.00 0.00 C

ATOM 2665 HA THR 162 57.539 33.400 2.294 1.00 0.00 H

ATOM 2666 CB THR 162 58.307 35.338 1.964 1.00 0.00 C

ATOM 2667 HB THR 162 58.713 35.225 2.969 1.00 0.00 H

ATOM 2668 CG2 THR 162 59.414 35.011 0.983 1.00 0.00 C

ATOM 2669 HG21 THR 162 59.885 34.050 1.188 1.00 0.00 H

ATOM 2670 HG22 THR 162 59.146 34.860 -0.063 1.00 0.00 H

ATOM 2671 HG23 THR 162 60.211 35.755 0.990 1.00 0.00 H

ATOM 2672 OG1 THR 162 57.825 36.639 1.820 1.00 0.00 O

ATOM 2673 HG1 THR 162 57.271 36.684 1.037 1.00 0.00 H

ATOM 2674 C THR 162 56.009 34.674 2.967 1.00 0.00 C

ATOM 2675 O THR 162 54.884 35.080 2.616 1.00 0.00 O

ATOM 2676 N ILE 163 56.125 34.072 4.203 1.00 0.00 N

ATOM 2677 H ILE 163 56.980 33.553 4.342 1.00 0.00 H

ATOM 2678 CA ILE 163 55.188 34.067 5.364 1.00 0.00 C

ATOM 2679 HA ILE 163 54.203 34.294 4.957 1.00 0.00 H

ATOM 2680 CB ILE 163 55.235 32.708 6.215 1.00 0.00 C

ATOM 2681 HB ILE 163 56.091 32.635 6.887 1.00 0.00 H

ATOM 2682 CG2 ILE 163 53.960 32.709 6.968 1.00 0.00 C

ATOM 2683 HG21 ILE 163 53.992 31.780 7.538 1.00 0.00 H

ATOM 2684 HG22 ILE 163 54.026 33.528 7.684 1.00 0.00 H

ATOM 2685 HG23 ILE 163 53.060 32.805 6.361 1.00 0.00 H

ATOM 2686 CG1 ILE 163 55.142 31.332 5.420 1.00 0.00 C

ATOM 2687 HG12 ILE 163 56.094 31.216 4.904 1.00 0.00 H

ATOM 2688 HG13 ILE 163 55.153 30.482 6.102 1.00 0.00 H

ATOM 2689 CD1 ILE 163 54.180 31.221 4.254 1.00 0.00 C

ATOM 2690 HD11 ILE 163 54.269 32.033 3.533 1.00 0.00 H

ATOM 2691 HD12 ILE 163 54.328 30.354 3.611 1.00 0.00 H

ATOM 2692 HD13 ILE 163 53.184 31.237 4.696 1.00 0.00 H

ATOM 2693 C ILE 163 55.507 35.312 6.121 1.00 0.00 C

ATOM 2694 O ILE 163 56.661 35.685 6.341 1.00 0.00 O

ATOM 2695 N THR 164 54.469 35.993 6.637 1.00 0.00 N

ATOM 2696 H THR 164 53.539 35.664 6.416 1.00 0.00 H

ATOM 2697 CA THR 164 54.559 37.299 7.387 1.00 0.00 C

ATOM 2698 HA THR 164 55.556 37.540 7.755 1.00 0.00 H

ATOM 2699 CB THR 164 54.129 38.531 6.506 1.00 0.00 C

ATOM 2700 HB THR 164 54.924 38.628 5.767 1.00 0.00 H

ATOM 2701 CG2 THR 164 52.728 38.384 5.882 1.00 0.00 C

ATOM 2702 HG21 THR 164 52.819 37.739 5.008 1.00 0.00 H

ATOM 2703 HG22 THR 164 52.099 38.046 6.706 1.00 0.00 H

ATOM 2704 HG23 THR 164 52.408 39.289 5.366 1.00 0.00 H

ATOM 2705 OG1 THR 164 54.210 39.684 7.263 1.00 0.00 O

ATOM 2706 HG1 THR 164 53.967 40.414 6.690 1.00 0.00 H

ATOM 2707 C THR 164 53.565 37.294 8.576 1.00 0.00 C

ATOM 2708 O THR 164 52.591 36.519 8.574 1.00 0.00 O

ATOM 2709 N CYX 165 53.784 38.085 9.660 1.00 0.00 N

ATOM 2710 H CYX 165 54.651 38.602 9.659 1.00 0.00 H

ATOM 2711 CA CYX 165 52.986 38.369 10.892 1.00 0.00 C

ATOM 2712 HA CYX 165 52.438 37.446 11.078 1.00 0.00 H

ATOM 2713 CB CYX 165 53.841 38.814 12.078 1.00 0.00 C

ATOM 2714 HB2 CYX 165 54.799 38.339 11.862 1.00 0.00 H

ATOM 2715 HB3 CYX 165 53.958 39.893 11.978 1.00 0.00 H

ATOM 2716 SG CYX 165 53.471 38.566 13.875 1.00 0.00 S

ATOM 2717 C CYX 165 51.905 39.381 10.427 1.00 0.00 C

ATOM 2718 O CYX 165 51.993 40.603 10.676 1.00 0.00 O

ATOM 2719 N TYR 166 51.032 38.994 9.513 1.00 0.00 N

ATOM 2720 H TYR 166 51.239 38.096 9.100 1.00 0.00 H

ATOM 2721 CA TYR 166 49.978 39.781 8.905 1.00 0.00 C

ATOM 2722 HA TYR 166 49.540 40.519 9.578 1.00 0.00 H

ATOM 2723 CB TYR 166 50.432 40.394 7.551 1.00 0.00 C

ATOM 2724 HB2 TYR 166 51.513 40.523 7.498 1.00 0.00 H

ATOM 2725 HB3 TYR 166 50.169 39.717 6.738 1.00 0.00 H

ATOM 2726 CG TYR 166 49.741 41.684 7.188 1.00 0.00 C

ATOM 2727 CD1 TYR 166 48.574 41.726 6.362 1.00 0.00 C

ATOM 2728 HD1 TYR 166 48.318 40.859 5.772 1.00 0.00 H

ATOM 2729 CE1 TYR 166 47.920 42.924 6.165 1.00 0.00 C

ATOM 2730 HE1 TYR 166 47.332 42.969 5.260 1.00 0.00 H

ATOM 2731 CZ TYR 166 48.360 44.051 6.950 1.00 0.00 C

ATOM 2732 OH TYR 166 47.575 45.202 7.094 1.00 0.00 O

ATOM 2733 HH TYR 166 46.749 45.192 6.605 1.00 0.00 H

ATOM 2734 CE2 TYR 166 49.624 44.054 7.559 1.00 0.00 C

ATOM 2735 HE2 TYR 166 49.819 44.897 8.205 1.00 0.00 H

ATOM 2736 CD2 TYR 166 50.334 42.842 7.648 1.00 0.00 C

ATOM 2737 HD2 TYR 166 51.271 42.931 8.178 1.00 0.00 H

ATOM 2738 C TYR 166 48.854 38.785 8.663 1.00 0.00 C

ATOM 2739 O TYR 166 49.060 37.890 7.820 1.00 0.00 O

ATOM 2740 N ASP 167 47.713 38.790 9.386 1.00 0.00 N

ATOM 2741 H ASP 167 47.528 39.522 10.057 1.00 0.00 H

ATOM 2742 CA ASP 167 46.898 37.580 9.669 1.00 0.00 C

ATOM 2743 HA ASP 167 47.630 36.829 9.967 1.00 0.00 H

ATOM 2744 CB ASP 167 46.003 37.990 10.913 1.00 0.00 C

ATOM 2745 HB2 ASP 167 46.591 38.506 11.672 1.00 0.00 H

ATOM 2746 HB3 ASP 167 45.228 38.676 10.574 1.00 0.00 H

ATOM 2747 CG ASP 167 45.245 36.853 11.613 1.00 0.00 C

ATOM 2748 OD1 ASP 167 45.819 35.751 11.702 1.00 0.00 O

ATOM 2749 OD2 ASP 167 44.002 36.851 11.667 1.00 0.00 O

ATOM 2750 C ASP 167 46.051 37.049 8.522 1.00 0.00 C

ATOM 2751 O ASP 167 45.636 37.872 7.689 1.00 0.00 O

ATOM 2752 N THR 168 45.925 35.707 8.344 1.00 0.00 N

ATOM 2753 H THR 168 46.431 35.093 8.966 1.00 0.00 H

ATOM 2754 CA THR 168 45.196 35.147 7.259 1.00 0.00 C

ATOM 2755 HA THR 168 45.672 35.490 6.340 1.00 0.00 H

ATOM 2756 CB THR 168 45.107 33.534 7.230 1.00 0.00 C

ATOM 2757 HB THR 168 44.818 33.123 8.197 1.00 0.00 H

ATOM 2758 CG2 THR 168 44.186 32.800 6.219 1.00 0.00 C

ATOM 2759 HG21 THR 168 43.127 32.673 6.441 1.00 0.00 H

ATOM 2760 HG22 THR 168 44.062 33.400 5.317 1.00 0.00 H

ATOM 2761 HG23 THR 168 44.552 31.812 5.940 1.00 0.00 H

ATOM 2762 OG1 THR 168 46.467 33.187 6.869 1.00 0.00 O

ATOM 2763 HG1 THR 168 47.089 33.428 7.559 1.00 0.00 H

ATOM 2764 C THR 168 43.750 35.721 7.014 1.00 0.00 C

ATOM 2765 O THR 168 42.896 35.689 7.881 1.00 0.00 O

ATOM 2766 N THR 169 43.437 36.097 5.783 1.00 0.00 N

ATOM 2767 H THR 169 44.259 36.262 5.221 1.00 0.00 H

ATOM 2768 CA THR 169 42.096 36.391 5.312 1.00 0.00 C

ATOM 2769 HA THR 169 41.586 36.856 6.156 1.00 0.00 H

ATOM 2770 CB THR 169 42.066 37.487 4.209 1.00 0.00 C

ATOM 2771 HB THR 169 41.182 37.572 3.576 1.00 0.00 H

ATOM 2772 CG2 THR 169 42.131 38.804 4.948 1.00 0.00 C

ATOM 2773 HG21 THR 169 41.161 39.033 5.388 1.00 0.00 H

ATOM 2774 HG22 THR 169 42.901 38.935 5.708 1.00 0.00 H

ATOM 2775 HG23 THR 169 42.173 39.576 4.179 1.00 0.00 H

ATOM 2776 OG1 THR 169 43.220 37.450 3.409 1.00 0.00 O

ATOM 2777 HG1 THR 169 42.919 37.614 2.512 1.00 0.00 H

ATOM 2778 C THR 169 41.360 35.148 4.925 1.00 0.00 C

ATOM 2779 O THR 169 41.472 34.658 3.756 1.00 0.00 O

ATOM 2780 N SER 170 40.607 34.523 5.877 1.00 0.00 N

ATOM 2781 H SER 170 40.645 34.953 6.790 1.00 0.00 H

ATOM 2782 CA SER 170 39.634 33.470 5.678 1.00 0.00 C

ATOM 2783 HA SER 170 39.106 33.545 4.728 1.00 0.00 H

ATOM 2784 CB SER 170 40.251 32.035 5.718 1.00 0.00 C

ATOM 2785 HB2 SER 170 41.159 32.086 6.320 1.00 0.00 H

ATOM 2786 HB3 SER 170 39.575 31.253 6.063 1.00 0.00 H

ATOM 2787 OG SER 170 40.844 31.629 4.433 1.00 0.00 O

ATOM 2788 HG SER 170 40.074 31.648 3.860 1.00 0.00 H

ATOM 2789 C SER 170 38.564 33.536 6.880 1.00 0.00 C

ATOM 2790 O SER 170 38.694 34.240 7.828 1.00 0.00 O

ATOM 2791 N ASP 171 37.575 32.743 6.780 1.00 0.00 N

ATOM 2792 H ASP 171 37.571 32.010 6.086 1.00 0.00 H

ATOM 2793 CA ASP 171 36.292 32.886 7.402 1.00 0.00 C

ATOM 2794 HA ASP 171 36.198 33.933 7.689 1.00 0.00 H

ATOM 2795 CB ASP 171 35.221 32.455 6.372 1.00 0.00 C

ATOM 2796 HB2 ASP 171 35.587 33.072 5.551 1.00 0.00 H

ATOM 2797 HB3 ASP 171 35.230 31.403 6.089 1.00 0.00 H

ATOM 2798 CG ASP 171 33.815 32.876 6.728 1.00 0.00 C

ATOM 2799 OD1 ASP 171 33.147 33.628 6.011 1.00 0.00 O

ATOM 2800 OD2 ASP 171 33.339 32.355 7.774 1.00 0.00 O

ATOM 2801 C ASP 171 36.058 32.003 8.673 1.00 0.00 C

ATOM 2802 O ASP 171 36.235 32.543 9.796 1.00 0.00 O

ATOM 2803 N GLU 172 36.106 30.688 8.514 1.00 0.00 N

ATOM 2804 H GLU 172 36.299 30.342 7.585 1.00 0.00 H

ATOM 2805 CA GLU 172 36.190 29.743 9.628 1.00 0.00 C

ATOM 2806 HA GLU 172 35.433 30.024 10.360 1.00 0.00 H

ATOM 2807 CB GLU 172 35.960 28.287 9.106 1.00 0.00 C

ATOM 2808 HB2 GLU 172 35.002 28.208 8.593 1.00 0.00 H

ATOM 2809 HB3 GLU 172 36.739 27.953 8.420 1.00 0.00 H

ATOM 2810 CG GLU 172 35.868 27.291 10.293 1.00 0.00 C

ATOM 2811 HG2 GLU 172 36.748 26.659 10.412 1.00 0.00 H

ATOM 2812 HG3 GLU 172 35.596 27.765 11.236 1.00 0.00 H

ATOM 2813 CD GLU 172 34.772 26.299 10.169 1.00 0.00 C

ATOM 2814 OE1 GLU 172 33.571 26.660 10.356 1.00 0.00 O

ATOM 2815 OE2 GLU 172 34.957 25.168 9.647 1.00 0.00 O

ATOM 2816 C GLU 172 37.558 29.744 10.389 1.00 0.00 C

ATOM 2817 O GLU 172 37.354 29.757 11.645 1.00 0.00 O

ATOM 2818 N TYR 173 38.712 29.953 9.750 1.00 0.00 N

ATOM 2819 H TYR 173 38.734 29.591 8.807 1.00 0.00 H

ATOM 2820 CA TYR 173 39.945 30.492 10.369 1.00 0.00 C

ATOM 2821 HA TYR 173 40.467 29.676 10.869 1.00 0.00 H

ATOM 2822 CB TYR 173 40.748 31.027 9.154 1.00 0.00 C

ATOM 2823 HB2 TYR 173 41.006 30.217 8.472 1.00 0.00 H

ATOM 2824 HB3 TYR 173 40.135 31.797 8.687 1.00 0.00 H

ATOM 2825 CG TYR 173 42.098 31.627 9.532 1.00 0.00 C

ATOM 2826 CD1 TYR 173 43.070 30.760 9.881 1.00 0.00 C

ATOM 2827 HD1 TYR 173 42.959 29.714 9.637 1.00 0.00 H

ATOM 2828 CE1 TYR 173 44.295 31.252 10.354 1.00 0.00 C

ATOM 2829 HE1 TYR 173 45.038 30.546 10.694 1.00 0.00 H

ATOM 2830 CZ TYR 173 44.493 32.653 10.455 1.00 0.00 C

ATOM 2831 OH TYR 173 45.505 33.128 11.108 1.00 0.00 O

ATOM 2832 HH TYR 173 45.530 34.041 11.404 1.00 0.00 H

ATOM 2833 CE2 TYR 173 43.402 33.512 10.167 1.00 0.00 C

ATOM 2834 HE2 TYR 173 43.585 34.570 10.284 1.00 0.00 H

ATOM 2835 CD2 TYR 173 42.189 33.000 9.721 1.00 0.00 C

ATOM 2836 HD2 TYR 173 41.422 33.698 9.421 1.00 0.00 H

ATOM 2837 C TYR 173 39.747 31.530 11.396 1.00 0.00 C

ATOM 2838 O TYR 173 39.809 31.316 12.570 1.00 0.00 O

ATOM 2839 N LEU 174 39.101 32.603 10.940 1.00 0.00 N

ATOM 2840 H LEU 174 38.835 32.806 9.986 1.00 0.00 H

ATOM 2841 CA LEU 174 38.822 33.709 11.908 1.00 0.00 C

ATOM 2842 HA LEU 174 39.789 33.877 12.382 1.00 0.00 H

ATOM 2843 CB LEU 174 38.333 34.940 11.171 1.00 0.00 C

ATOM 2844 HB2 LEU 174 38.252 34.677 10.116 1.00 0.00 H

ATOM 2845 HB3 LEU 174 37.346 35.221 11.538 1.00 0.00 H

ATOM 2846 CG LEU 174 39.197 36.231 11.251 1.00 0.00 C

ATOM 2847 HG LEU 174 40.225 35.970 10.998 1.00 0.00 H

ATOM 2848 CD1 LEU 174 38.767 37.310 10.306 1.00 0.00 C

ATOM 2849 HD11 LEU 174 37.689 37.470 10.339 1.00 0.00 H

ATOM 2850 HD12 LEU 174 39.295 38.246 10.484 1.00 0.00 H

ATOM 2851 HD13 LEU 174 39.015 36.942 9.310 1.00 0.00 H

ATOM 2852 CD2 LEU 174 39.182 36.726 12.693 1.00 0.00 C

ATOM 2853 HD21 LEU 174 39.335 35.924 13.415 1.00 0.00 H

ATOM 2854 HD22 LEU 174 39.929 37.493 12.898 1.00 0.00 H

ATOM 2855 HD23 LEU 174 38.188 37.140 12.864 1.00 0.00 H

ATOM 2856 C LEU 174 37.831 33.348 13.053 1.00 0.00 C

ATOM 2857 O LEU 174 38.148 33.584 14.226 1.00 0.00 O

ATOM 2858 N ARG 175 36.623 32.783 12.711 1.00 0.00 N

ATOM 2859 H ARG 175 36.491 32.673 11.716 1.00 0.00 H

ATOM 2860 CA ARG 175 35.588 32.470 13.676 1.00 0.00 C

ATOM 2861 HA ARG 175 35.416 33.335 14.316 1.00 0.00 H

ATOM 2862 CB ARG 175 34.415 32.006 12.901 1.00 0.00 C

ATOM 2863 HB2 ARG 175 34.779 31.292 12.162 1.00 0.00 H

ATOM 2864 HB3 ARG 175 33.743 31.528 13.614 1.00 0.00 H

ATOM 2865 CG ARG 175 33.717 33.128 12.113 1.00 0.00 C

ATOM 2866 HG2 ARG 175 33.438 33.881 12.851 1.00 0.00 H

ATOM 2867 HG3 ARG 175 34.385 33.638 11.419 1.00 0.00 H

ATOM 2868 CD ARG 175 32.447 32.511 11.337 1.00 0.00 C

ATOM 2869 HD2 ARG 175 32.732 31.902 10.478 1.00 0.00 H

ATOM 2870 HD3 ARG 175 31.855 31.825 11.942 1.00 0.00 H

ATOM 2871 NE ARG 175 31.652 33.689 10.959 1.00 0.00 N

ATOM 2872 HE ARG 175 31.635 34.456 11.616 1.00 0.00 H

ATOM 2873 CZ ARG 175 30.913 33.908 9.909 1.00 0.00 C

ATOM 2874 NH1 ARG 175 30.103 34.956 9.952 1.00 0.00 N

ATOM 2875 HH11 ARG 175 29.846 35.345 9.056 1.00 0.00 H

ATOM 2876 HH12 ARG 175 29.464 35.167 10.705 1.00 0.00 H

ATOM 2877 NH2 ARG 175 31.074 33.232 8.755 1.00 0.00 N

ATOM 2878 HH21 ARG 175 30.494 33.373 7.940 1.00 0.00 H

ATOM 2879 HH22 ARG 175 31.958 32.768 8.600 1.00 0.00 H

ATOM 2880 C ARG 175 36.027 31.382 14.692 1.00 0.00 C

ATOM 2881 O ARG 175 35.550 31.451 15.816 1.00 0.00 O

ATOM 2882 N SER 176 36.926 30.454 14.319 1.00 0.00 N

ATOM 2883 H SER 176 37.361 30.549 13.412 1.00 0.00 H

ATOM 2884 CA SER 176 37.490 29.554 15.237 1.00 0.00 C

ATOM 2885 HA SER 176 36.692 28.948 15.668 1.00 0.00 H

ATOM 2886 CB SER 176 38.402 28.577 14.478 1.00 0.00 C

ATOM 2887 HB2 SER 176 39.103 29.116 13.842 1.00 0.00 H

ATOM 2888 HB3 SER 176 38.797 27.937 15.268 1.00 0.00 H

ATOM 2889 OG SER 176 37.669 27.783 13.551 1.00 0.00 O

ATOM 2890 HG SER 176 37.420 28.421 12.878 1.00 0.00 H

ATOM 2891 C SER 176 38.262 30.304 16.343 1.00 0.00 C

ATOM 2892 O SER 176 38.191 29.994 17.539 1.00 0.00 O

ATOM 2893 N TYR 177 39.134 31.225 15.997 1.00 0.00 N

ATOM 2894 H TYR 177 39.349 31.321 15.015 1.00 0.00 H

ATOM 2895 CA TYR 177 39.910 32.041 16.977 1.00 0.00 C

ATOM 2896 HA TYR 177 40.084 31.404 17.844 1.00 0.00 H

ATOM 2897 CB TYR 177 41.290 32.368 16.426 1.00 0.00 C

ATOM 2898 HB2 TYR 177 41.851 32.548 17.342 1.00 0.00 H

ATOM 2899 HB3 TYR 177 41.670 31.539 15.829 1.00 0.00 H

ATOM 2900 CG TYR 177 41.464 33.592 15.622 1.00 0.00 C

ATOM 2901 CD1 TYR 177 41.919 33.539 14.288 1.00 0.00 C

ATOM 2902 HD1 TYR 177 41.970 32.607 13.746 1.00 0.00 H

ATOM 2903 CE1 TYR 177 42.256 34.759 13.630 1.00 0.00 C

ATOM 2904 HE1 TYR 177 42.759 34.834 12.677 1.00 0.00 H

ATOM 2905 CZ TYR 177 42.119 36.022 14.304 1.00 0.00 C

ATOM 2906 OH TYR 177 42.599 37.094 13.716 1.00 0.00 O

ATOM 2907 HH TYR 177 43.063 36.914 12.895 1.00 0.00 H

ATOM 2908 CE2 TYR 177 41.791 36.030 15.707 1.00 0.00 C

ATOM 2909 HE2 TYR 177 41.838 36.961 16.252 1.00 0.00 H

ATOM 2910 CD2 TYR 177 41.553 34.808 16.389 1.00 0.00 C

ATOM 2911 HD2 TYR 177 41.425 34.821 17.462 1.00 0.00 H

ATOM 2912 C TYR 177 39.172 33.154 17.745 1.00 0.00 C

ATOM 2913 O TYR 177 39.545 33.582 18.836 1.00 0.00 O

ATOM 2914 N PHE 178 37.958 33.475 17.268 1.00 0.00 N

ATOM 2915 H PHE 178 37.872 33.331 16.272 1.00 0.00 H

ATOM 2916 CA PHE 178 37.076 34.523 17.837 1.00 0.00 C

ATOM 2917 HA PHE 178 37.597 35.467 17.999 1.00 0.00 H

ATOM 2918 CB PHE 178 35.883 34.832 16.845 1.00 0.00 C

ATOM 2919 HB2 PHE 178 36.221 35.045 15.831 1.00 0.00 H

ATOM 2920 HB3 PHE 178 35.285 33.927 16.741 1.00 0.00 H

ATOM 2921 CG PHE 178 34.959 35.918 17.396 1.00 0.00 C

ATOM 2922 CD1 PHE 178 33.610 35.579 17.707 1.00 0.00 C

ATOM 2923 HD1 PHE 178 33.159 34.676 17.321 1.00 0.00 H

ATOM 2924 CE1 PHE 178 32.777 36.611 18.184 1.00 0.00 C

ATOM 2925 HE1 PHE 178 31.764 36.440 18.517 1.00 0.00 H

ATOM 2926 CZ PHE 178 33.292 37.924 18.200 1.00 0.00 C

ATOM 2927 HZ PHE 178 32.546 38.674 18.420 1.00 0.00 H

ATOM 2928 CE2 PHE 178 34.537 38.251 17.671 1.00 0.00 C

ATOM 2929 HE2 PHE 178 34.719 39.290 17.441 1.00 0.00 H

ATOM 2930 CD2 PHE 178 35.435 37.241 17.325 1.00 0.00 C

ATOM 2931 HD2 PHE 178 36.308 37.498 16.742 1.00 0.00 H

ATOM 2932 C PHE 178 36.457 34.031 19.137 1.00 0.00 C

ATOM 2933 O PHE 178 36.689 34.801 20.071 1.00 0.00 O

ATOM 2934 N ILE 179 35.960 32.819 19.176 1.00 0.00 N

ATOM 2935 H ILE 179 35.776 32.375 18.288 1.00 0.00 H

ATOM 2936 CA ILE 179 35.431 32.263 20.388 1.00 0.00 C

ATOM 2937 HA ILE 179 34.822 33.000 20.911 1.00 0.00 H

ATOM 2938 CB ILE 179 34.532 30.973 20.168 1.00 0.00 C

ATOM 2939 HB ILE 179 33.705 31.267 19.522 1.00 0.00 H

ATOM 2940 CG2 ILE 179 35.273 29.872 19.357 1.00 0.00 C

ATOM 2941 HG21 ILE 179 34.805 28.903 19.536 1.00 0.00 H

ATOM 2942 HG22 ILE 179 35.191 30.188 18.317 1.00 0.00 H

ATOM 2943 HG23 ILE 179 36.290 29.752 19.731 1.00 0.00 H

ATOM 2944 CG1 ILE 179 33.920 30.395 21.477 1.00 0.00 C

ATOM 2945 HG12 ILE 179 33.474 29.419 21.284 1.00 0.00 H

ATOM 2946 HG13 ILE 179 34.812 30.236 22.083 1.00 0.00 H

ATOM 2947 CD1 ILE 179 32.952 31.270 22.235 1.00 0.00 C

ATOM 2948 HD11 ILE 179 31.961 31.359 21.788 1.00 0.00 H

ATOM 2949 HD12 ILE 179 32.786 30.906 23.249 1.00 0.00 H

ATOM 2950 HD13 ILE 179 33.395 32.258 22.359 1.00 0.00 H

ATOM 2951 C ILE 179 36.572 31.895 21.379 1.00 0.00 C

ATOM 2952 O ILE 179 36.371 32.033 22.582 1.00 0.00 O

ATOM 2953 N TYR 180 37.802 31.594 20.951 1.00 0.00 N

ATOM 2954 H TYR 180 38.006 31.603 19.962 1.00 0.00 H

ATOM 2955 CA TYR 180 38.914 31.401 21.872 1.00 0.00 C

ATOM 2956 HA TYR 180 38.535 30.805 22.702 1.00 0.00 H

ATOM 2957 CB TYR 180 40.037 30.600 21.184 1.00 0.00 C

ATOM 2958 HB2 TYR 180 39.547 30.203 20.295 1.00 0.00 H

ATOM 2959 HB3 TYR 180 40.803 31.293 20.838 1.00 0.00 H

ATOM 2960 CG TYR 180 40.666 29.527 22.116 1.00 0.00 C

ATOM 2961 CD1 TYR 180 41.437 30.035 23.180 1.00 0.00 C

ATOM 2962 HD1 TYR 180 41.422 31.077 23.463 1.00 0.00 H

ATOM 2963 CE1 TYR 180 42.023 29.123 24.061 1.00 0.00 C

ATOM 2964 HE1 TYR 180 42.559 29.477 24.929 1.00 0.00 H

ATOM 2965 CZ TYR 180 41.809 27.792 23.937 1.00 0.00 C

ATOM 2966 OH TYR 180 42.339 26.971 24.895 1.00 0.00 O

ATOM 2967 HH TYR 180 42.893 27.457 25.510 1.00 0.00 H

ATOM 2968 CE2 TYR 180 40.941 27.295 22.978 1.00 0.00 C

ATOM 2969 HE2 TYR 180 40.741 26.241 22.856 1.00 0.00 H

ATOM 2970 CD2 TYR 180 40.541 28.149 21.940 1.00 0.00 C

ATOM 2971 HD2 TYR 180 39.887 27.747 21.181 1.00 0.00 H

ATOM 2972 C TYR 180 39.446 32.724 22.501 1.00 0.00 C

ATOM 2973 O TYR 180 39.593 32.751 23.673 1.00 0.00 O

ATOM 2974 N SER 181 39.461 33.729 21.643 1.00 0.00 N

ATOM 2975 H SER 181 39.472 33.565 20.646 1.00 0.00 H

ATOM 2976 CA SER 181 39.656 35.169 21.996 1.00 0.00 C

ATOM 2977 HA SER 181 40.542 35.256 22.625 1.00 0.00 H

ATOM 2978 CB SER 181 39.956 36.174 20.924 1.00 0.00 C

ATOM 2979 HB2 SER 181 39.110 36.133 20.238 1.00 0.00 H

ATOM 2980 HB3 SER 181 40.045 37.199 21.283 1.00 0.00 H

ATOM 2981 OG SER 181 41.055 35.835 20.142 1.00 0.00 O

ATOM 2982 HG SER 181 41.602 36.621 20.069 1.00 0.00 H

ATOM 2983 C SER 181 38.520 35.749 22.863 1.00 0.00 C

ATOM 2984 O SER 181 38.774 36.669 23.587 1.00 0.00 O

ATOM 2985 N MET 182 37.297 35.114 22.871 1.00 0.00 N

ATOM 2986 H MET 182 37.147 34.324 22.260 1.00 0.00 H

ATOM 2987 CA MET 182 36.295 35.595 23.739 1.00 0.00 C

ATOM 2988 HA MET 182 36.515 36.612 24.064 1.00 0.00 H

ATOM 2989 CB MET 182 34.924 35.466 23.175 1.00 0.00 C

ATOM 2990 HB2 MET 182 34.684 34.604 22.552 1.00 0.00 H

ATOM 2991 HB3 MET 182 34.182 35.419 23.972 1.00 0.00 H

ATOM 2992 CG MET 182 34.829 36.763 22.257 1.00 0.00 C

ATOM 2993 HG2 MET 182 35.234 37.524 22.925 1.00 0.00 H

ATOM 2994 HG3 MET 182 35.420 36.805 21.342 1.00 0.00 H

ATOM 2995 SD MET 182 33.094 37.146 21.914 1.00 0.00 S

ATOM 2996 CE MET 182 32.732 37.977 23.533 1.00 0.00 C

ATOM 2997 HE1 MET 182 33.110 37.314 24.311 1.00 0.00 H

ATOM 2998 HE2 MET 182 33.256 38.916 23.709 1.00 0.00 H

ATOM 2999 HE3 MET 182 31.676 38.102 23.773 1.00 0.00 H

ATOM 3000 C MET 182 36.512 34.832 25.071 1.00 0.00 C

ATOM 3001 O MET 182 36.244 35.463 26.097 1.00 0.00 O

ATOM 3002 N CYX 183 37.059 33.604 25.110 1.00 0.00 N

ATOM 3003 H CYX 183 37.233 33.130 24.235 1.00 0.00 H

ATOM 3004 CA CYX 183 37.272 32.859 26.333 1.00 0.00 C

ATOM 3005 HA CYX 183 36.341 32.966 26.888 1.00 0.00 H

ATOM 3006 CB CYX 183 37.470 31.399 25.979 1.00 0.00 C

ATOM 3007 HB2 CYX 183 38.232 31.194 25.227 1.00 0.00 H

ATOM 3008 HB3 CYX 183 37.709 30.806 26.862 1.00 0.00 H

ATOM 3009 SG CYX 183 35.978 30.591 25.327 1.00 0.00 S

ATOM 3010 C CYX 183 38.330 33.471 27.250 1.00 0.00 C

ATOM 3011 O CYX 183 38.181 33.416 28.471 1.00 0.00 O

ATOM 3012 N THR 184 39.332 34.093 26.679 1.00 0.00 N

ATOM 3013 H THR 184 39.412 34.139 25.673 1.00 0.00 H

ATOM 3014 CA THR 184 40.337 34.823 27.366 1.00 0.00 C

ATOM 3015 HA THR 184 40.546 34.338 28.319 1.00 0.00 H

ATOM 3016 CB THR 184 41.647 35.021 26.572 1.00 0.00 C

ATOM 3017 HB THR 184 42.249 35.733 27.136 1.00 0.00 H

ATOM 3018 CG2 THR 184 42.390 33.767 26.656 1.00 0.00 C

ATOM 3019 HG21 THR 184 41.947 33.110 25.907 1.00 0.00 H

ATOM 3020 HG22 THR 184 43.409 33.965 26.323 1.00 0.00 H

ATOM 3021 HG23 THR 184 42.475 33.511 27.712 1.00 0.00 H

ATOM 3022 OG1 THR 184 41.356 35.376 25.242 1.00 0.00 O

ATOM 3023 HG1 THR 184 42.198 35.486 24.794 1.00 0.00 H

ATOM 3024 C THR 184 39.817 36.275 27.649 1.00 0.00 C

ATOM 3025 O THR 184 39.759 36.671 28.784 1.00 0.00 O

ATOM 3026 N THR 185 39.234 36.916 26.644 1.00 0.00 N

ATOM 3027 H THR 185 39.304 36.472 25.740 1.00 0.00 H

ATOM 3028 CA THR 185 38.786 38.288 26.838 1.00 0.00 C

ATOM 3029 HA THR 185 39.606 38.894 27.226 1.00 0.00 H

ATOM 3030 CB THR 185 38.162 38.988 25.531 1.00 0.00 C

ATOM 3031 HB THR 185 37.311 38.364 25.255 1.00 0.00 H

ATOM 3032 CG2 THR 185 37.664 40.456 25.788 1.00 0.00 C

ATOM 3033 HG21 THR 185 38.143 41.050 26.566 1.00 0.00 H

ATOM 3034 HG22 THR 185 37.600 41.033 24.865 1.00 0.00 H

ATOM 3035 HG23 THR 185 36.699 40.179 26.212 1.00 0.00 H

ATOM 3036 OG1 THR 185 39.077 39.117 24.523 1.00 0.00 O

ATOM 3037 HG1 THR 185 39.136 38.247 24.122 1.00 0.00 H

ATOM 3038 C THR 185 37.693 38.462 27.990 1.00 0.00 C

ATOM 3039 O THR 185 37.662 39.443 28.788 1.00 0.00 O

ATOM 3040 N VAL 186 36.916 37.359 28.204 1.00 0.00 N

ATOM 3041 H VAL 186 36.773 36.734 27.423 1.00 0.00 H

ATOM 3042 CA VAL 186 35.925 37.288 29.334 1.00 0.00 C

ATOM 3043 HA VAL 186 35.733 38.335 29.568 1.00 0.00 H

ATOM 3044 CB VAL 186 34.622 36.544 28.932 1.00 0.00 C

ATOM 3045 HB VAL 186 34.909 35.494 28.883 1.00 0.00 H

ATOM 3046 CG1 VAL 186 33.657 36.602 30.133 1.00 0.00 C

ATOM 3047 HG11 VAL 186 32.716 36.185 29.773 1.00 0.00 H

ATOM 3048 HG12 VAL 186 34.040 35.984 30.945 1.00 0.00 H

ATOM 3049 HG13 VAL 186 33.420 37.542 30.630 1.00 0.00 H

ATOM 3050 CG2 VAL 186 33.803 37.027 27.788 1.00 0.00 C

ATOM 3051 HG21 VAL 186 34.443 37.249 26.935 1.00 0.00 H

ATOM 3052 HG22 VAL 186 32.955 36.392 27.531 1.00 0.00 H

ATOM 3053 HG23 VAL 186 33.331 37.970 28.064 1.00 0.00 H

ATOM 3054 C VAL 186 36.554 36.647 30.603 1.00 0.00 C

ATOM 3055 O VAL 186 36.575 37.317 31.682 1.00 0.00 O

ATOM 3056 N ALA 187 36.990 35.383 30.477 1.00 0.00 N

ATOM 3057 H ALA 187 37.274 35.036 29.573 1.00 0.00 H

ATOM 3058 CA ALA 187 37.296 34.648 31.649 1.00 0.00 C

ATOM 3059 HA ALA 187 36.585 34.795 32.462 1.00 0.00 H

ATOM 3060 CB ALA 187 37.242 33.068 31.321 1.00 0.00 C

ATOM 3061 HB1 ALA 187 36.489 32.763 30.596 1.00 0.00 H

ATOM 3062 HB2 ALA 187 38.255 32.778 31.039 1.00 0.00 H

ATOM 3063 HB3 ALA 187 37.041 32.466 32.207 1.00 0.00 H

ATOM 3064 C ALA 187 38.681 35.042 32.291 1.00 0.00 C

ATOM 3065 O ALA 187 38.813 35.085 33.499 1.00 0.00 O

ATOM 3066 N MET 188 39.613 35.399 31.482 1.00 0.00 N

ATOM 3067 H MET 188 39.476 34.962 30.582 1.00 0.00 H

ATOM 3068 CA MET 188 40.974 35.706 31.836 1.00 0.00 C

ATOM 3069 HA MET 188 41.175 35.427 32.870 1.00 0.00 H

ATOM 3070 CB MET 188 42.024 35.004 30.998 1.00 0.00 C

ATOM 3071 HB2 MET 188 41.846 35.437 30.014 1.00 0.00 H

ATOM 3072 HB3 MET 188 43.009 35.157 31.438 1.00 0.00 H

ATOM 3073 CG MET 188 41.958 33.529 30.649 1.00 0.00 C

ATOM 3074 HG2 MET 188 41.045 33.220 30.140 1.00 0.00 H

ATOM 3075 HG3 MET 188 42.800 33.184 30.048 1.00 0.00 H

ATOM 3076 SD MET 188 41.956 32.546 32.146 1.00 0.00 S

ATOM 3077 CE MET 188 43.754 32.640 32.701 1.00 0.00 C

ATOM 3078 HE1 MET 188 44.412 32.281 31.909 1.00 0.00 H

ATOM 3079 HE2 MET 188 43.857 33.659 33.073 1.00 0.00 H

ATOM 3080 HE3 MET 188 43.921 31.935 33.516 1.00 0.00 H

ATOM 3081 C MET 188 41.274 37.189 31.812 1.00 0.00 C

ATOM 3082 O MET 188 42.380 37.606 32.078 1.00 0.00 O

ATOM 3083 N PHE 189 40.295 38.097 31.496 1.00 0.00 N

ATOM 3084 H PHE 189 39.475 37.632 31.135 1.00 0.00 H

ATOM 3085 CA PHE 189 40.309 39.550 31.755 1.00 0.00 C

ATOM 3086 HA PHE 189 41.191 39.741 32.367 1.00 0.00 H

ATOM 3087 CB PHE 189 40.454 40.199 30.417 1.00 0.00 C

ATOM 3088 HB2 PHE 189 41.423 39.831 30.081 1.00 0.00 H

ATOM 3089 HB3 PHE 189 39.850 39.793 29.606 1.00 0.00 H

ATOM 3090 CG PHE 189 40.485 41.723 30.560 1.00 0.00 C

ATOM 3091 CD1 PHE 189 41.624 42.478 30.942 1.00 0.00 C

ATOM 3092 HD1 PHE 189 42.519 41.945 31.230 1.00 0.00 H

ATOM 3093 CE1 PHE 189 41.588 43.842 30.903 1.00 0.00 C

ATOM 3094 HE1 PHE 189 42.484 44.366 31.202 1.00 0.00 H

ATOM 3095 CZ PHE 189 40.451 44.532 30.502 1.00 0.00 C

ATOM 3096 HZ PHE 189 40.348 45.607 30.485 1.00 0.00 H

ATOM 3097 CE2 PHE 189 39.284 43.776 30.383 1.00 0.00 C

ATOM 3098 HE2 PHE 189 38.354 44.326 30.388 1.00 0.00 H

ATOM 3099 CD2 PHE 189 39.352 42.372 30.298 1.00 0.00 C

ATOM 3100 HD2 PHE 189 38.455 41.835 30.029 1.00 0.00 H

ATOM 3101 C PHE 189 39.047 39.998 32.616 1.00 0.00 C

ATOM 3102 O PHE 189 39.220 40.458 33.730 1.00 0.00 O

ATOM 3103 N CYX 190 37.869 39.819 32.053 1.00 0.00 N

ATOM 3104 H CYX 190 37.889 39.838 31.044 1.00 0.00 H

ATOM 3105 CA CYX 190 36.657 40.617 32.460 1.00 0.00 C

ATOM 3106 HA CYX 190 37.011 41.644 32.555 1.00 0.00 H

ATOM 3107 CB CYX 190 35.617 40.432 31.300 1.00 0.00 C

ATOM 3108 HB2 CYX 190 36.009 40.848 30.371 1.00 0.00 H

ATOM 3109 HB3 CYX 190 35.447 39.356 31.263 1.00 0.00 H

ATOM 3110 SG CYX 190 33.917 41.110 31.337 1.00 0.00 S

ATOM 3111 C CYX 190 36.127 40.176 33.859 1.00 0.00 C

ATOM 3112 O CYX 190 35.913 40.990 34.784 1.00 0.00 O

ATOM 3113 N VAL 191 35.957 38.865 34.005 1.00 0.00 N

ATOM 3114 H VAL 191 36.212 38.319 33.194 1.00 0.00 H

ATOM 3115 CA VAL 191 35.615 38.098 35.273 1.00 0.00 C

ATOM 3116 HA VAL 191 34.626 38.479 35.526 1.00 0.00 H

ATOM 3117 CB VAL 191 35.411 36.569 35.035 1.00 0.00 C

ATOM 3118 HB VAL 191 36.336 36.248 34.557 1.00 0.00 H

ATOM 3119 CG1 VAL 191 35.210 35.678 36.275 1.00 0.00 C

ATOM 3120 HG11 VAL 191 35.922 34.854 36.230 1.00 0.00 H

ATOM 3121 HG12 VAL 191 35.433 36.061 37.271 1.00 0.00 H

ATOM 3122 HG13 VAL 191 34.216 35.232 36.314 1.00 0.00 H

ATOM 3123 CG2 VAL 191 34.197 36.339 34.078 1.00 0.00 C

ATOM 3124 HG21 VAL 191 34.495 36.854 33.165 1.00 0.00 H

ATOM 3125 HG22 VAL 191 34.074 35.260 33.980 1.00 0.00 H

ATOM 3126 HG23 VAL 191 33.238 36.732 34.416 1.00 0.00 H

ATOM 3127 C VAL 191 36.602 38.409 36.441 1.00 0.00 C

ATOM 3128 O VAL 191 36.128 38.530 37.588 1.00 0.00 O

ATOM 3129 N PRO 192 37.928 38.513 36.239 1.00 0.00 N

ATOM 3130 CD PRO 192 38.603 37.593 35.355 1.00 0.00 C

ATOM 3131 HD2 PRO 192 38.533 38.038 34.363 1.00 0.00 H

ATOM 3132 HD3 PRO 192 38.252 36.577 35.537 1.00 0.00 H

ATOM 3133 CG PRO 192 40.082 37.747 35.701 1.00 0.00 C

ATOM 3134 HG2 PRO 192 40.566 38.372 34.950 1.00 0.00 H

ATOM 3135 HG3 PRO 192 40.569 36.778 35.816 1.00 0.00 H

ATOM 3136 CB PRO 192 40.169 38.629 36.953 1.00 0.00 C

ATOM 3137 HB2 PRO 192 40.860 39.454 36.778 1.00 0.00 H

ATOM 3138 HB3 PRO 192 40.747 38.162 37.750 1.00 0.00 H

ATOM 3139 CA PRO 192 38.781 39.105 37.273 1.00 0.00 C

ATOM 3140 HA PRO 192 38.568 38.633 38.232 1.00 0.00 H

ATOM 3141 C PRO 192 38.522 40.635 37.424 1.00 0.00 C

ATOM 3142 O PRO 192 38.579 41.204 38.519 1.00 0.00 O

ATOM 3143 N LEU 193 38.363 41.319 36.232 1.00 0.00 N

ATOM 3144 H LEU 193 38.387 40.863 35.331 1.00 0.00 H

ATOM 3145 CA LEU 193 38.409 42.785 36.250 1.00 0.00 C

ATOM 3146 HA LEU 193 39.176 43.222 36.889 1.00 0.00 H

ATOM 3147 CB LEU 193 38.354 43.347 34.778 1.00 0.00 C

ATOM 3148 HB2 LEU 193 39.191 42.965 34.193 1.00 0.00 H

ATOM 3149 HB3 LEU 193 37.444 42.986 34.298 1.00 0.00 H

ATOM 3150 CG LEU 193 38.449 44.851 34.798 1.00 0.00 C

ATOM 3151 HG LEU 193 38.344 45.273 35.797 1.00 0.00 H

ATOM 3152 CD1 LEU 193 39.881 45.304 34.507 1.00 0.00 C

ATOM 3153 HD11 LEU 193 40.432 45.050 33.601 1.00 0.00 H

ATOM 3154 HD12 LEU 193 39.952 46.382 34.651 1.00 0.00 H

ATOM 3155 HD13 LEU 193 40.574 44.797 35.180 1.00 0.00 H

ATOM 3156 CD2 LEU 193 37.396 45.396 33.885 1.00 0.00 C

ATOM 3157 HD21 LEU 193 37.647 45.238 32.836 1.00 0.00 H

ATOM 3158 HD22 LEU 193 36.510 44.865 34.232 1.00 0.00 H

ATOM 3159 HD23 LEU 193 37.167 46.403 34.232 1.00 0.00 H

ATOM 3160 C LEU 193 37.157 43.354 37.012 1.00 0.00 C

ATOM 3161 O LEU 193 37.258 44.299 37.842 1.00 0.00 O

ATOM 3162 N VAL 194 35.981 42.738 36.920 1.00 0.00 N

ATOM 3163 H VAL 194 35.906 42.114 36.130 1.00 0.00 H

ATOM 3164 CA VAL 194 34.847 43.072 37.819 1.00 0.00 C

ATOM 3165 HA VAL 194 34.713 44.151 37.897 1.00 0.00 H

ATOM 3166 CB VAL 194 33.517 42.388 37.388 1.00 0.00 C

ATOM 3167 HB VAL 194 32.707 42.606 38.085 1.00 0.00 H

ATOM 3168 CG1 VAL 194 32.982 43.121 36.064 1.00 0.00 C

ATOM 3169 HG11 VAL 194 32.143 42.613 35.589 1.00 0.00 H

ATOM 3170 HG12 VAL 194 32.771 44.140 36.387 1.00 0.00 H

ATOM 3171 HG13 VAL 194 33.783 43.268 35.339 1.00 0.00 H

ATOM 3172 CG2 VAL 194 33.508 40.856 37.278 1.00 0.00 C

ATOM 3173 HG21 VAL 194 32.498 40.461 37.394 1.00 0.00 H

ATOM 3174 HG22 VAL 194 34.035 40.480 36.401 1.00 0.00 H

ATOM 3175 HG23 VAL 194 34.019 40.369 38.109 1.00 0.00 H

ATOM 3176 C VAL 194 35.132 42.636 39.301 1.00 0.00 C

ATOM 3177 O VAL 194 34.661 43.343 40.176 1.00 0.00 O

ATOM 3178 N LEU 195 35.904 41.525 39.623 1.00 0.00 N

ATOM 3179 H LEU 195 36.230 41.012 38.817 1.00 0.00 H

ATOM 3180 CA LEU 195 36.311 41.096 40.962 1.00 0.00 C

ATOM 3181 HA LEU 195 35.395 40.971 41.539 1.00 0.00 H

ATOM 3182 CB LEU 195 37.036 39.745 40.861 1.00 0.00 C

ATOM 3183 HB2 LEU 195 36.431 39.181 40.151 1.00 0.00 H

ATOM 3184 HB3 LEU 195 38.064 39.708 40.498 1.00 0.00 H

ATOM 3185 CG LEU 195 37.099 39.088 42.213 1.00 0.00 C

ATOM 3186 HG LEU 195 36.100 39.057 42.648 1.00 0.00 H

ATOM 3187 CD1 LEU 195 37.644 37.726 41.980 1.00 0.00 C

ATOM 3188 HD11 LEU 195 36.988 37.188 41.296 1.00 0.00 H

ATOM 3189 HD12 LEU 195 38.636 37.540 41.567 1.00 0.00 H

ATOM 3190 HD13 LEU 195 37.631 37.106 42.877 1.00 0.00 H

ATOM 3191 CD2 LEU 195 38.124 39.741 43.143 1.00 0.00 C

ATOM 3192 HD21 LEU 195 38.438 39.071 43.943 1.00 0.00 H

ATOM 3193 HD22 LEU 195 39.032 40.078 42.642 1.00 0.00 H

ATOM 3194 HD23 LEU 195 37.705 40.660 43.552 1.00 0.00 H

ATOM 3195 C LEU 195 37.172 42.265 41.567 1.00 0.00 C

ATOM 3196 O LEU 195 36.889 42.818 42.616 1.00 0.00 O

ATOM 3197 N ILE 196 38.208 42.606 40.780 1.00 0.00 N

ATOM 3198 H ILE 196 38.552 41.945 40.099 1.00 0.00 H

ATOM 3199 CA ILE 196 39.103 43.754 41.091 1.00 0.00 C

ATOM 3200 HA ILE 196 39.622 43.464 42.005 1.00 0.00 H

ATOM 3201 CB ILE 196 40.125 44.116 39.999 1.00 0.00 C

ATOM 3202 HB ILE 196 39.575 44.438 39.115 1.00 0.00 H

ATOM 3203 CG2 ILE 196 40.946 45.393 40.469 1.00 0.00 C

ATOM 3204 HG21 ILE 196 40.367 46.271 40.184 1.00 0.00 H

ATOM 3205 HG22 ILE 196 41.217 45.329 41.523 1.00 0.00 H

ATOM 3206 HG23 ILE 196 41.900 45.418 39.943 1.00 0.00 H

ATOM 3207 CG1 ILE 196 41.181 43.051 39.629 1.00 0.00 C

ATOM 3208 HG12 ILE 196 41.886 43.102 40.460 1.00 0.00 H

ATOM 3209 HG13 ILE 196 40.763 42.045 39.627 1.00 0.00 H

ATOM 3210 CD1 ILE 196 42.004 43.373 38.397 1.00 0.00 C

ATOM 3211 HD11 ILE 196 41.363 43.545 37.532 1.00 0.00 H

ATOM 3212 HD12 ILE 196 42.661 44.242 38.401 1.00 0.00 H

ATOM 3213 HD13 ILE 196 42.660 42.513 38.262 1.00 0.00 H

ATOM 3214 C ILE 196 38.310 45.044 41.389 1.00 0.00 C

ATOM 3215 O ILE 196 38.221 45.376 42.546 1.00 0.00 O

ATOM 3216 N LEU 197 37.446 45.429 40.484 1.00 0.00 N

ATOM 3217 H LEU 197 37.236 44.649 39.877 1.00 0.00 H

ATOM 3218 CA LEU 197 36.647 46.656 40.641 1.00 0.00 C

ATOM 3219 HA LEU 197 37.271 47.498 40.942 1.00 0.00 H

ATOM 3220 CB LEU 197 36.025 47.094 39.317 1.00 0.00 C

ATOM 3221 HB2 LEU 197 35.542 46.162 39.023 1.00 0.00 H

ATOM 3222 HB3 LEU 197 35.150 47.735 39.426 1.00 0.00 H

ATOM 3223 CG LEU 197 36.856 47.654 38.227 1.00 0.00 C

ATOM 3224 HG LEU 197 37.713 46.982 38.255 1.00 0.00 H

ATOM 3225 CD1 LEU 197 36.057 47.412 36.938 1.00 0.00 C

ATOM 3226 HD11 LEU 197 36.558 48.021 36.185 1.00 0.00 H

ATOM 3227 HD12 LEU 197 36.020 46.377 36.601 1.00 0.00 H

ATOM 3228 HD13 LEU 197 35.016 47.700 37.084 1.00 0.00 H

ATOM 3229 CD2 LEU 197 37.320 49.175 38.494 1.00 0.00 C

ATOM 3230 HD21 LEU 197 38.235 49.350 37.927 1.00 0.00 H

ATOM 3231 HD22 LEU 197 36.581 49.968 38.387 1.00 0.00 H

ATOM 3232 HD23 LEU 197 37.650 49.174 39.532 1.00 0.00 H

ATOM 3233 C LEU 197 35.575 46.614 41.843 1.00 0.00 C

ATOM 3234 O LEU 197 35.489 47.459 42.747 1.00 0.00 O

ATOM 3235 N GLY 198 34.807 45.546 41.865 1.00 0.00 N

ATOM 3236 H GLY 198 34.912 44.969 41.043 1.00 0.00 H

ATOM 3237 CA GLY 198 33.795 45.298 42.895 1.00 0.00 C

ATOM 3238 HA2 GLY 198 33.087 46.124 42.834 1.00 0.00 H

ATOM 3239 HA3 GLY 198 33.245 44.369 42.743 1.00 0.00 H

ATOM 3240 C GLY 198 34.424 45.171 44.248 1.00 0.00 C

ATOM 3241 O GLY 198 33.704 45.496 45.223 1.00 0.00 O

ATOM 3242 N CYX 199 35.677 44.662 44.368 1.00 0.00 N

ATOM 3243 H CYX 199 36.169 44.354 43.542 1.00 0.00 H

ATOM 3244 CA CYX 199 36.485 44.821 45.598 1.00 0.00 C

ATOM 3245 HA CYX 199 35.685 44.742 46.335 1.00 0.00 H

ATOM 3246 CB CYX 199 37.313 43.557 45.928 1.00 0.00 C

ATOM 3247 HB2 CYX 199 37.544 43.578 46.993 1.00 0.00 H

ATOM 3248 HB3 CYX 199 36.616 42.736 45.760 1.00 0.00 H

ATOM 3249 SG CYX 199 38.732 43.293 44.884 1.00 0.00 S

ATOM 3250 C CYX 199 37.299 46.167 45.861 1.00 0.00 C

ATOM 3251 O CYX 199 37.273 46.584 47.020 1.00 0.00 O

ATOM 3252 N TYR 200 37.801 46.884 44.892 1.00 0.00 N

ATOM 3253 H TYR 200 37.785 46.453 43.979 1.00 0.00 H

ATOM 3254 CA TYR 200 38.347 48.241 44.957 1.00 0.00 C

ATOM 3255 HA TYR 200 39.232 48.068 45.569 1.00 0.00 H

ATOM 3256 CB TYR 200 38.805 48.623 43.576 1.00 0.00 C

ATOM 3257 HB2 TYR 200 38.786 47.748 42.926 1.00 0.00 H

ATOM 3258 HB3 TYR 200 38.112 49.402 43.259 1.00 0.00 H

ATOM 3259 CG TYR 200 40.243 49.088 43.649 1.00 0.00 C

ATOM 3260 CD1 TYR 200 40.529 50.436 44.005 1.00 0.00 C

ATOM 3261 HD1 TYR 200 39.704 51.086 44.257 1.00 0.00 H

ATOM 3262 CE1 TYR 200 41.828 50.854 44.196 1.00 0.00 C

ATOM 3263 HE1 TYR 200 42.018 51.893 44.420 1.00 0.00 H

ATOM 3264 CZ TYR 200 42.856 49.878 44.022 1.00 0.00 C

ATOM 3265 OH TYR 200 44.157 50.304 44.007 1.00 0.00 O

ATOM 3266 HH TYR 200 44.127 51.259 44.095 1.00 0.00 H

ATOM 3267 CE2 TYR 200 42.578 48.533 43.738 1.00 0.00 C

ATOM 3268 HE2 TYR 200 43.364 47.796 43.821 1.00 0.00 H

ATOM 3269 CD2 TYR 200 41.248 48.140 43.526 1.00 0.00 C

ATOM 3270 HD2 TYR 200 41.057 47.127 43.206 1.00 0.00 H

ATOM 3271 C TYR 200 37.504 49.332 45.633 1.00 0.00 C

ATOM 3272 O TYR 200 38.055 50.088 46.426 1.00 0.00 O

ATOM 3273 N GLY 201 36.172 49.253 45.555 1.00 0.00 N

ATOM 3274 H GLY 201 35.824 48.403 45.135 1.00 0.00 H

ATOM 3275 CA GLY 201 35.215 50.016 46.421 1.00 0.00 C

ATOM 3276 HA2 GLY 201 35.051 50.973 45.927 1.00 0.00 H

ATOM 3277 HA3 GLY 201 34.239 49.532 46.454 1.00 0.00 H

ATOM 3278 C GLY 201 35.639 49.949 47.951 1.00 0.00 C

ATOM 3279 O GLY 201 35.963 50.919 48.586 1.00 0.00 O

ATOM 3280 N LEU 202 35.883 48.742 48.467 1.00 0.00 N

ATOM 3281 H LEU 202 35.622 47.932 47.922 1.00 0.00 H

ATOM 3282 CA LEU 202 36.254 48.502 49.847 1.00 0.00 C

ATOM 3283 HA LEU 202 35.474 48.978 50.440 1.00 0.00 H

ATOM 3284 CB LEU 202 36.169 47.018 50.252 1.00 0.00 C

ATOM 3285 HB2 LEU 202 35.245 46.478 50.043 1.00 0.00 H

ATOM 3286 HB3 LEU 202 36.961 46.557 49.663 1.00 0.00 H

ATOM 3287 CG LEU 202 36.393 46.719 51.751 1.00 0.00 C

ATOM 3288 HG LEU 202 37.438 46.838 52.038 1.00 0.00 H

ATOM 3289 CD1 LEU 202 35.500 47.574 52.633 1.00 0.00 C

ATOM 3290 HD11 LEU 202 34.488 47.671 52.241 1.00 0.00 H

ATOM 3291 HD12 LEU 202 35.400 47.156 53.635 1.00 0.00 H

ATOM 3292 HD13 LEU 202 36.018 48.532 52.581 1.00 0.00 H

ATOM 3293 CD2 LEU 202 36.139 45.226 52.019 1.00 0.00 C

ATOM 3294 HD21 LEU 202 35.342 44.693 51.499 1.00 0.00 H

ATOM 3295 HD22 LEU 202 36.981 44.550 51.869 1.00 0.00 H

ATOM 3296 HD23 LEU 202 35.813 44.954 53.023 1.00 0.00 H

ATOM 3297 C LEU 202 37.653 49.099 50.114 1.00 0.00 C

ATOM 3298 O LEU 202 37.756 49.979 51.002 1.00 0.00 O

ATOM 3299 N ILE 203 38.585 48.916 49.208 1.00 0.00 N

ATOM 3300 H ILE 203 38.309 48.375 48.401 1.00 0.00 H

ATOM 3301 CA ILE 203 39.915 49.514 49.334 1.00 0.00 C

ATOM 3302 HA ILE 203 40.451 49.165 50.217 1.00 0.00 H

ATOM 3303 CB ILE 203 40.712 48.837 48.167 1.00 0.00 C

ATOM 3304 HB ILE 203 40.279 49.085 47.198 1.00 0.00 H

ATOM 3305 CG2 ILE 203 42.163 49.348 48.005 1.00 0.00 C

ATOM 3306 HG21 ILE 203 42.779 48.824 48.735 1.00 0.00 H

ATOM 3307 HG22 ILE 203 42.597 49.046 47.052 1.00 0.00 H

ATOM 3308 HG23 ILE 203 42.272 50.407 48.242 1.00 0.00 H

ATOM 3309 CG1 ILE 203 40.830 47.284 48.197 1.00 0.00 C

ATOM 3310 HG12 ILE 203 41.524 46.901 48.946 1.00 0.00 H

ATOM 3311 HG13 ILE 203 39.852 46.909 48.498 1.00 0.00 H

ATOM 3312 CD1 ILE 203 41.215 46.542 46.896 1.00 0.00 C

ATOM 3313 HD11 ILE 203 42.067 47.064 46.461 1.00 0.00 H

ATOM 3314 HD12 ILE 203 41.471 45.511 47.140 1.00 0.00 H

ATOM 3315 HD13 ILE 203 40.334 46.595 46.256 1.00 0.00 H

ATOM 3316 C ILE 203 39.959 51.037 49.437 1.00 0.00 C

ATOM 3317 O ILE 203 40.810 51.619 50.178 1.00 0.00 O

ATOM 3318 N VAL 204 39.038 51.662 48.700 1.00 0.00 N

ATOM 3319 H VAL 204 38.725 51.007 47.997 1.00 0.00 H

ATOM 3320 CA VAL 204 38.713 53.111 48.658 1.00 0.00 C

ATOM 3321 HA VAL 204 39.680 53.595 48.520 1.00 0.00 H

ATOM 3322 CB VAL 204 37.993 53.432 47.328 1.00 0.00 C

ATOM 3323 HB VAL 204 37.094 52.820 47.254 1.00 0.00 H

ATOM 3324 CG1 VAL 204 37.506 54.861 47.195 1.00 0.00 C

ATOM 3325 HG11 VAL 204 36.836 55.022 46.351 1.00 0.00 H

ATOM 3326 HG12 VAL 204 36.873 55.043 48.064 1.00 0.00 H

ATOM 3327 HG13 VAL 204 38.312 55.592 47.133 1.00 0.00 H

ATOM 3328 CG2 VAL 204 38.939 53.119 46.165 1.00 0.00 C

ATOM 3329 HG21 VAL 204 39.500 53.981 45.804 1.00 0.00 H

ATOM 3330 HG22 VAL 204 39.606 52.262 46.257 1.00 0.00 H

ATOM 3331 HG23 VAL 204 38.349 52.946 45.265 1.00 0.00 H

ATOM 3332 C VAL 204 38.004 53.620 49.967 1.00 0.00 C

ATOM 3333 O VAL 204 38.096 54.857 50.316 1.00 0.00 O

ATOM 3334 N ARG 205 37.185 52.821 50.591 1.00 0.00 N

ATOM 3335 H ARG 205 36.913 51.972 50.116 1.00 0.00 H

ATOM 3336 CA ARG 205 36.637 52.936 51.931 1.00 0.00 C

ATOM 3337 HA ARG 205 36.210 53.916 52.147 1.00 0.00 H

ATOM 3338 CB ARG 205 35.390 52.061 52.128 1.00 0.00 C

ATOM 3339 HB2 ARG 205 35.567 50.988 52.056 1.00 0.00 H

ATOM 3340 HB3 ARG 205 35.096 52.038 53.177 1.00 0.00 H

ATOM 3341 CG ARG 205 34.066 52.406 51.354 1.00 0.00 C

ATOM 3342 HG2 ARG 205 33.839 53.455 51.542 1.00 0.00 H

ATOM 3343 HG3 ARG 205 34.192 52.192 50.293 1.00 0.00 H

ATOM 3344 CD ARG 205 32.926 51.501 51.988 1.00 0.00 C

ATOM 3345 HD2 ARG 205 33.177 50.462 52.205 1.00 0.00 H

ATOM 3346 HD3 ARG 205 32.565 51.951 52.913 1.00 0.00 H

ATOM 3347 NE ARG 205 31.758 51.447 50.957 1.00 0.00 N

ATOM 3348 HE ARG 205 31.696 52.263 50.365 1.00 0.00 H

ATOM 3349 CZ ARG 205 30.717 50.628 50.849 1.00 0.00 C

ATOM 3350 NH1 ARG 205 29.915 50.931 49.885 1.00 0.00 N

ATOM 3351 HH11 ARG 205 28.939 50.687 49.962 1.00 0.00 H

ATOM 3352 HH12 ARG 205 30.079 51.830 49.455 1.00 0.00 H

ATOM 3353 NH2 ARG 205 30.516 49.541 51.632 1.00 0.00 N

ATOM 3354 HH21 ARG 205 30.034 48.751 51.228 1.00 0.00 H

ATOM 3355 HH22 ARG 205 31.011 49.524 52.512 1.00 0.00 H

ATOM 3356 C ARG 205 37.635 52.746 53.050 1.00 0.00 C

ATOM 3357 O ARG 205 37.464 53.252 54.161 1.00 0.00 O

ATOM 3358 N ALA 206 38.672 51.948 52.913 1.00 0.00 N

ATOM 3359 H ALA 206 38.877 51.528 52.018 1.00 0.00 H

ATOM 3360 CA ALA 206 39.682 51.726 53.978 1.00 0.00 C

ATOM 3361 HA ALA 206 39.234 51.963 54.943 1.00 0.00 H

ATOM 3362 CB ALA 206 40.132 50.270 54.027 1.00 0.00 C

ATOM 3363 HB1 ALA 206 39.285 49.588 54.087 1.00 0.00 H

ATOM 3364 HB2 ALA 206 40.721 49.983 53.156 1.00 0.00 H

ATOM 3365 HB3 ALA 206 40.826 50.218 54.867 1.00 0.00 H

ATOM 3366 C ALA 206 40.845 52.743 53.849 1.00 0.00 C

ATOM 3367 O ALA 206 42.020 52.447 54.112 1.00 0.00 O

ATOM 3368 N LEU 207 40.579 53.939 53.364 1.00 0.00 N

ATOM 3369 H LEU 207 39.607 54.190 53.249 1.00 0.00 H

ATOM 3370 CA LEU 207 41.534 55.006 53.059 1.00 0.00 C

ATOM 3371 HA LEU 207 42.356 55.017 53.775 1.00 0.00 H

ATOM 3372 CB LEU 207 42.362 54.620 51.720 1.00 0.00 C

ATOM 3373 HB2 LEU 207 42.274 53.535 51.668 1.00 0.00 H

ATOM 3374 HB3 LEU 207 41.826 54.994 50.848 1.00 0.00 H

ATOM 3375 CG LEU 207 43.900 54.911 51.705 1.00 0.00 C

ATOM 3376 HG LEU 207 44.398 54.787 52.667 1.00 0.00 H

ATOM 3377 CD1 LEU 207 44.499 53.887 50.683 1.00 0.00 C

ATOM 3378 HD11 LEU 207 44.233 52.892 51.038 1.00 0.00 H

ATOM 3379 HD12 LEU 207 44.270 54.062 49.632 1.00 0.00 H

ATOM 3380 HD13 LEU 207 45.583 53.784 50.728 1.00 0.00 H

ATOM 3381 CD2 LEU 207 44.111 56.335 51.178 1.00 0.00 C

ATOM 3382 HD21 LEU 207 44.193 56.365 50.092 1.00 0.00 H

ATOM 3383 HD22 LEU 207 43.240 56.933 51.445 1.00 0.00 H

ATOM 3384 HD23 LEU 207 45.047 56.673 51.622 1.00 0.00 H

ATOM 3385 C LEU 207 41.127 56.488 52.818 1.00 0.00 C

ATOM 3386 O LEU 207 41.641 57.442 53.352 1.00 0.00 O

ATOM 3387 N ILE 208 40.108 56.624 51.980 1.00 0.00 N

ATOM 3388 H ILE 208 39.747 55.820 51.486 1.00 0.00 H

ATOM 3389 CA ILE 208 39.812 57.985 51.382 1.00 0.00 C

ATOM 3390 HA ILE 208 40.248 58.739 52.036 1.00 0.00 H

ATOM 3391 CB ILE 208 40.530 58.192 50.000 1.00 0.00 C

ATOM 3392 HB ILE 208 41.541 57.790 50.062 1.00 0.00 H

ATOM 3393 CG2 ILE 208 39.869 57.496 48.833 1.00 0.00 C

ATOM 3394 HG21 ILE 208 38.886 57.879 48.559 1.00 0.00 H

ATOM 3395 HG22 ILE 208 40.503 57.580 47.951 1.00 0.00 H

ATOM 3396 HG23 ILE 208 39.754 56.450 49.121 1.00 0.00 H

ATOM 3397 CG1 ILE 208 40.623 59.722 49.750 1.00 0.00 C

ATOM 3398 HG12 ILE 208 39.650 60.171 49.552 1.00 0.00 H

ATOM 3399 HG13 ILE 208 40.888 60.201 50.693 1.00 0.00 H

ATOM 3400 CD1 ILE 208 41.518 60.171 48.595 1.00 0.00 C

ATOM 3401 HD11 ILE 208 42.453 59.620 48.693 1.00 0.00 H

ATOM 3402 HD12 ILE 208 41.261 59.825 47.594 1.00 0.00 H

ATOM 3403 HD13 ILE 208 41.735 61.238 48.563 1.00 0.00 H

ATOM 3404 C ILE 208 38.381 58.502 51.383 1.00 0.00 C

ATOM 3405 O ILE 208 38.195 59.689 51.646 1.00 0.00 O

ATOM 3406 N TYR 209 37.367 57.688 51.202 1.00 0.00 N

ATOM 3407 H TYR 209 37.681 56.763 51.456 1.00 0.00 H

ATOM 3408 CA TYR 209 35.934 57.854 50.729 1.00 0.00 C

ATOM 3409 HA TYR 209 36.091 58.115 49.683 1.00 0.00 H

ATOM 3410 CB TYR 209 35.097 56.535 50.769 1.00 0.00 C

ATOM 3411 HB2 TYR 209 34.223 56.662 50.130 1.00 0.00 H

ATOM 3412 HB3 TYR 209 35.642 55.650 50.441 1.00 0.00 H

ATOM 3413 CG TYR 209 34.503 56.254 52.135 1.00 0.00 C

ATOM 3414 CD1 TYR 209 35.300 56.052 53.292 1.00 0.00 C

ATOM 3415 HD1 TYR 209 36.376 55.973 53.238 1.00 0.00 H

ATOM 3416 CE1 TYR 209 34.719 55.713 54.509 1.00 0.00 C

ATOM 3417 HE1 TYR 209 35.374 55.593 55.359 1.00 0.00 H

ATOM 3418 CZ TYR 209 33.337 55.615 54.596 1.00 0.00 C

ATOM 3419 OH TYR 209 32.711 55.345 55.749 1.00 0.00 O

ATOM 3420 HH TYR 209 31.754 55.356 55.676 1.00 0.00 H

ATOM 3421 CE2 TYR 209 32.557 55.874 53.427 1.00 0.00 C

ATOM 3422 HE2 TYR 209 31.479 55.896 53.487 1.00 0.00 H

ATOM 3423 CD2 TYR 209 33.206 55.929 52.164 1.00 0.00 C

ATOM 3424 HD2 TYR 209 32.556 56.041 51.308 1.00 0.00 H

ATOM 3425 C TYR 209 35.186 59.059 51.392 1.00 0.00 C

ATOM 3426 O TYR 209 34.574 59.881 50.663 1.00 0.00 O

ATOM 3427 N LYS 210 35.226 59.149 52.693 1.00 0.00 N

ATOM 3428 H LYS 210 35.805 58.463 53.156 1.00 0.00 H

ATOM 3429 CA LYS 210 34.372 60.003 53.538 1.00 0.00 C

ATOM 3430 HA LYS 210 33.426 59.974 52.998 1.00 0.00 H

ATOM 3431 CB LYS 210 33.937 59.374 54.898 1.00 0.00 C

ATOM 3432 HB2 LYS 210 33.455 58.405 54.774 1.00 0.00 H

ATOM 3433 HB3 LYS 210 34.760 59.228 55.598 1.00 0.00 H

ATOM 3434 CG LYS 210 32.933 60.317 55.652 1.00 0.00 C

ATOM 3435 HG2 LYS 210 33.348 61.299 55.884 1.00 0.00 H

ATOM 3436 HG3 LYS 210 32.004 60.424 55.092 1.00 0.00 H

ATOM 3437 CD LYS 210 32.387 59.678 56.938 1.00 0.00 C

ATOM 3438 HD2 LYS 210 31.350 60.011 56.948 1.00 0.00 H

ATOM 3439 HD3 LYS 210 32.283 58.594 56.884 1.00 0.00 H

ATOM 3440 CE LYS 210 33.134 60.053 58.216 1.00 0.00 C

ATOM 3441 HE2 LYS 210 34.202 59.852 58.292 1.00 0.00 H

ATOM 3442 HE3 LYS 210 33.011 61.132 58.113 1.00 0.00 H

ATOM 3443 NZ LYS 210 32.369 59.590 59.370 1.00 0.00 N

ATOM 3444 HZ1 LYS 210 32.813 59.784 60.256 1.00 0.00 H

ATOM 3445 HZ2 LYS 210 31.382 59.754 59.503 1.00 0.00 H

ATOM 3446 HZ3 LYS 210 32.471 58.586 59.393 1.00 0.00 H

ATOM 3447 C LYS 210 34.910 61.423 53.666 1.00 0.00 C

ATOM 3448 O LYS 210 34.217 62.397 53.373 1.00 0.00 O

ATOM 3449 N ASP 211 36.200 61.479 53.941 1.00 0.00 N

ATOM 3450 H ASP 211 36.685 60.644 54.237 1.00 0.00 H

ATOM 3451 CA ASP 211 36.698 62.716 54.592 1.00 0.00 C

ATOM 3452 HA ASP 211 35.825 63.366 54.650 1.00 0.00 H

ATOM 3453 CB ASP 211 37.229 62.452 56.025 1.00 0.00 C

ATOM 3454 HB2 ASP 211 37.486 63.441 56.405 1.00 0.00 H

ATOM 3455 HB3 ASP 211 36.437 62.049 56.656 1.00 0.00 H

ATOM 3456 CG ASP 211 38.334 61.450 56.144 1.00 0.00 C

ATOM 3457 OD1 ASP 211 39.435 61.879 56.618 1.00 0.00 O

ATOM 3458 OD2 ASP 211 38.149 60.254 55.938 1.00 0.00 O

ATOM 3459 C ASP 211 37.758 63.437 53.814 1.00 0.00 C

ATOM 3460 O ASP 211 38.011 64.636 54.020 1.00 0.00 O

ATOM 3461 N LEU 212 38.505 62.763 52.957 1.00 0.00 N

ATOM 3462 H LEU 212 38.451 61.754 52.982 1.00 0.00 H

ATOM 3463 CA LEU 212 39.425 63.291 51.992 1.00 0.00 C

ATOM 3464 HA LEU 212 39.798 62.584 51.251 1.00 0.00 H

ATOM 3465 CB LEU 212 38.679 64.259 51.119 1.00 0.00 C

ATOM 3466 HB2 LEU 212 37.668 63.904 50.922 1.00 0.00 H

ATOM 3467 HB3 LEU 212 38.531 65.209 51.632 1.00 0.00 H

ATOM 3468 CG LEU 212 39.201 64.441 49.679 1.00 0.00 C

ATOM 3469 HG LEU 212 40.178 64.923 49.639 1.00 0.00 H

ATOM 3470 CD1 LEU 212 39.238 63.161 48.924 1.00 0.00 C

ATOM 3471 HD11 LEU 212 39.503 63.368 47.887 1.00 0.00 H

ATOM 3472 HD12 LEU 212 39.903 62.406 49.344 1.00 0.00 H

ATOM 3473 HD13 LEU 212 38.235 62.741 48.847 1.00 0.00 H

ATOM 3474 CD2 LEU 212 38.322 65.431 48.939 1.00 0.00 C

ATOM 3475 HD21 LEU 212 38.530 65.526 47.874 1.00 0.00 H

ATOM 3476 HD22 LEU 212 37.281 65.161 49.117 1.00 0.00 H

ATOM 3477 HD23 LEU 212 38.431 66.412 49.403 1.00 0.00 H

ATOM 3478 C LEU 212 40.754 63.961 52.596 1.00 0.00 C

ATOM 3479 O LEU 212 41.531 64.365 51.747 1.00 0.00 O

ATOM 3480 N ASP 213 40.794 64.310 53.893 1.00 0.00 N

ATOM 3481 H ASP 213 39.810 64.324 54.121 1.00 0.00 H

ATOM 3482 CA ASP 213 41.841 65.049 54.625 1.00 0.00 C

ATOM 3483 HA ASP 213 41.792 66.128 54.474 1.00 0.00 H

ATOM 3484 CB ASP 213 41.639 64.663 56.082 1.00 0.00 C

ATOM 3485 HB2 ASP 213 40.643 64.289 56.319 1.00 0.00 H

ATOM 3486 HB3 ASP 213 42.191 63.816 56.490 1.00 0.00 H

ATOM 3487 CG ASP 213 41.901 65.942 56.917 1.00 0.00 C

ATOM 3488 OD1 ASP 213 40.968 66.791 56.987 1.00 0.00 O

ATOM 3489 OD2 ASP 213 42.869 66.096 57.671 1.00 0.00 O

ATOM 3490 C ASP 213 43.347 64.675 54.163 1.00 0.00 C

ATOM 3491 O ASP 213 43.720 63.537 54.094 1.00 0.00 O

ATOM 3492 N ASN 214 44.113 65.698 53.836 1.00 0.00 N

ATOM 3493 H ASN 214 43.588 66.534 53.623 1.00 0.00 H

ATOM 3494 CA ASN 214 45.546 65.566 53.657 1.00 0.00 C

ATOM 3495 HA ASN 214 45.791 64.860 52.864 1.00 0.00 H

ATOM 3496 CB ASN 214 46.076 66.850 53.102 1.00 0.00 C

ATOM 3497 HB2 ASN 214 45.510 67.079 52.199 1.00 0.00 H

ATOM 3498 HB3 ASN 214 46.010 67.706 53.774 1.00 0.00 H

ATOM 3499 CG ASN 214 47.516 66.768 52.624 1.00 0.00 C

ATOM 3500 OD1 ASN 214 48.330 67.514 53.170 1.00 0.00 O

ATOM 3501 ND2 ASN 214 47.800 66.032 51.575 1.00 0.00 N

ATOM 3502 HD21 ASN 214 47.201 65.264 51.306 1.00 0.00 H

ATOM 3503 HD22 ASN 214 48.604 66.269 51.012 1.00 0.00 H

ATOM 3504 C ASN 214 46.418 64.985 54.788 1.00 0.00 C

ATOM 3505 O ASN 214 46.947 65.714 55.520 1.00 0.00 O

ATOM 3506 N SER 215 46.691 63.656 54.722 1.00 0.00 N

ATOM 3507 H SER 215 46.071 63.050 54.202 1.00 0.00 H

ATOM 3508 CA SER 215 47.872 62.963 55.169 1.00 0.00 C

ATOM 3509 HA SER 215 48.305 63.603 55.937 1.00 0.00 H

ATOM 3510 CB SER 215 47.462 61.689 55.952 1.00 0.00 C

ATOM 3511 HB2 SER 215 47.136 61.947 56.960 1.00 0.00 H

ATOM 3512 HB3 SER 215 46.715 61.117 55.402 1.00 0.00 H

ATOM 3513 OG SER 215 48.580 60.882 56.170 1.00 0.00 O

ATOM 3514 HG SER 215 48.372 60.422 56.987 1.00 0.00 H

ATOM 3515 C SER 215 48.995 62.686 54.124 1.00 0.00 C

ATOM 3516 O SER 215 48.693 62.488 52.934 1.00 0.00 O

ATOM 3517 N PRO 216 50.273 62.736 54.542 1.00 0.00 N

ATOM 3518 CD PRO 216 50.739 63.012 55.918 1.00 0.00 C

ATOM 3519 HD2 PRO 216 50.019 62.623 56.639 1.00 0.00 H

ATOM 3520 HD3 PRO 216 50.793 64.091 56.060 1.00 0.00 H

ATOM 3521 CG PRO 216 52.079 62.408 56.128 1.00 0.00 C

ATOM 3522 HG2 PRO 216 51.925 61.434 56.591 1.00 0.00 H

ATOM 3523 HG3 PRO 216 52.724 63.098 56.674 1.00 0.00 H

ATOM 3524 CB PRO 216 52.540 62.197 54.708 1.00 0.00 C

ATOM 3525 HB2 PRO 216 53.090 61.256 54.746 1.00 0.00 H

ATOM 3526 HB3 PRO 216 53.184 63.010 54.374 1.00 0.00 H

ATOM 3527 CA PRO 216 51.333 62.150 53.771 1.00 0.00 C

ATOM 3528 HA PRO 216 51.643 62.817 52.966 1.00 0.00 H

ATOM 3529 C PRO 216 51.088 60.710 53.269 1.00 0.00 C

ATOM 3530 O PRO 216 51.547 60.361 52.176 1.00 0.00 O

ATOM 3531 N LEU 217 50.455 59.784 54.062 1.00 0.00 N

ATOM 3532 H LEU 217 50.081 60.129 54.934 1.00 0.00 H

ATOM 3533 CA LEU 217 50.158 58.444 53.602 1.00 0.00 C

ATOM 3534 HA LEU 217 51.024 57.900 53.226 1.00 0.00 H

ATOM 3535 CB LEU 217 49.643 57.638 54.767 1.00 0.00 C

ATOM 3536 HB2 LEU 217 49.256 58.321 55.524 1.00 0.00 H

ATOM 3537 HB3 LEU 217 48.795 57.010 54.495 1.00 0.00 H

ATOM 3538 CG LEU 217 50.677 56.700 55.566 1.00 0.00 C

ATOM 3539 HG LEU 217 50.153 56.292 56.430 1.00 0.00 H

ATOM 3540 CD1 LEU 217 50.891 55.506 54.621 1.00 0.00 C

ATOM 3541 HD11 LEU 217 51.542 55.938 53.861 1.00 0.00 H

ATOM 3542 HD12 LEU 217 51.526 54.765 55.106 1.00 0.00 H

ATOM 3543 HD13 LEU 217 49.956 55.046 54.299 1.00 0.00 H

ATOM 3544 CD2 LEU 217 52.012 57.364 55.968 1.00 0.00 C

ATOM 3545 HD21 LEU 217 52.487 56.811 56.779 1.00 0.00 H

ATOM 3546 HD22 LEU 217 52.623 57.552 55.085 1.00 0.00 H

ATOM 3547 HD23 LEU 217 51.700 58.297 56.437 1.00 0.00 H

ATOM 3548 C LEU 217 49.137 58.420 52.468 1.00 0.00 C

ATOM 3549 O LEU 217 49.357 57.826 51.428 1.00 0.00 O

ATOM 3550 N ARG 218 48.040 59.277 52.631 1.00 0.00 N

ATOM 3551 H ARG 218 47.990 59.874 53.443 1.00 0.00 H

ATOM 3552 CA ARG 218 47.089 59.549 51.545 1.00 0.00 C

ATOM 3553 HA ARG 218 46.629 58.602 51.264 1.00 0.00 H

ATOM 3554 CB ARG 218 45.842 60.406 52.024 1.00 0.00 C

ATOM 3555 HB2 ARG 218 46.290 61.248 52.552 1.00 0.00 H

ATOM 3556 HB3 ARG 218 45.310 60.974 51.260 1.00 0.00 H

ATOM 3557 CG ARG 218 44.923 59.538 52.893 1.00 0.00 C

ATOM 3558 HG2 ARG 218 44.370 58.903 52.200 1.00 0.00 H

ATOM 3559 HG3 ARG 218 45.495 58.981 53.635 1.00 0.00 H

ATOM 3560 CD ARG 218 43.907 60.526 53.495 1.00 0.00 C

ATOM 3561 HD2 ARG 218 44.423 61.100 54.265 1.00 0.00 H

ATOM 3562 HD3 ARG 218 43.405 61.127 52.737 1.00 0.00 H

ATOM 3563 NE ARG 218 42.884 59.810 54.169 1.00 0.00 N

ATOM 3564 HE ARG 218 42.968 58.806 54.253 1.00 0.00 H

ATOM 3565 CZ ARG 218 41.806 60.328 54.796 1.00 0.00 C

ATOM 3566 NH1 ARG 218 41.688 61.567 55.115 1.00 0.00 N

ATOM 3567 HH11 ARG 218 42.213 62.299 54.659 1.00 0.00 H

ATOM 3568 HH12 ARG 218 40.983 61.826 55.790 1.00 0.00 H

ATOM 3569 NH2 ARG 218 40.878 59.560 55.328 1.00 0.00 N

ATOM 3570 HH21 ARG 218 40.130 59.985 55.858 1.00 0.00 H

ATOM 3571 HH22 ARG 218 40.792 58.663 54.873 1.00 0.00 H

ATOM 3572 C ARG 218 47.698 60.017 50.227 1.00 0.00 C

ATOM 3573 O ARG 218 47.567 59.418 49.162 1.00 0.00 O

ATOM 3574 N ARG 219 48.550 60.997 50.232 1.00 0.00 N

ATOM 3575 H ARG 219 48.561 61.473 51.122 1.00 0.00 H

ATOM 3576 CA ARG 219 49.444 61.458 49.181 1.00 0.00 C

ATOM 3577 HA ARG 219 48.893 62.023 48.429 1.00 0.00 H

ATOM 3578 CB ARG 219 50.349 62.527 49.830 1.00 0.00 C

ATOM 3579 HB2 ARG 219 49.799 63.243 50.440 1.00 0.00 H

ATOM 3580 HB3 ARG 219 51.034 62.089 50.556 1.00 0.00 H

ATOM 3581 CG ARG 219 51.231 63.258 48.800 1.00 0.00 C

ATOM 3582 HG2 ARG 219 51.693 62.598 48.066 1.00 0.00 H

ATOM 3583 HG3 ARG 219 50.645 64.022 48.288 1.00 0.00 H

ATOM 3584 CD ARG 219 52.331 64.103 49.475 1.00 0.00 C

ATOM 3585 HD2 ARG 219 52.712 64.782 48.712 1.00 0.00 H

ATOM 3586 HD3 ARG 219 51.941 64.584 50.371 1.00 0.00 H

ATOM 3587 NE ARG 219 53.393 63.133 49.947 1.00 0.00 N

ATOM 3588 HE ARG 219 53.677 62.442 49.268 1.00 0.00 H

ATOM 3589 CZ ARG 219 54.161 63.263 51.016 1.00 0.00 C

ATOM 3590 NH1 ARG 219 53.878 64.127 51.917 1.00 0.00 N

ATOM 3591 HH11 ARG 219 54.479 64.337 52.701 1.00 0.00 H

ATOM 3592 HH12 ARG 219 53.315 64.908 51.614 1.00 0.00 H

ATOM 3593 NH2 ARG 219 54.971 62.323 51.252 1.00 0.00 N

ATOM 3594 HH21 ARG 219 55.357 62.174 52.174 1.00 0.00 H

ATOM 3595 HH22 ARG 219 55.349 61.818 50.463 1.00 0.00 H

ATOM 3596 C ARG 219 50.192 60.323 48.517 1.00 0.00 C

ATOM 3597 O ARG 219 50.099 60.229 47.294 1.00 0.00 O

ATOM 3598 N LYS 220 51.079 59.536 49.202 1.00 0.00 N

ATOM 3599 H LYS 220 51.132 59.599 50.209 1.00 0.00 H

ATOM 3600 CA LYS 220 51.697 58.239 48.692 1.00 0.00 C

ATOM 3601 HA LYS 220 52.389 58.521 47.898 1.00 0.00 H

ATOM 3602 CB LYS 220 52.475 57.493 49.843 1.00 0.00 C

ATOM 3603 HB2 LYS 220 51.876 57.154 50.687 1.00 0.00 H

ATOM 3604 HB3 LYS 220 52.904 56.599 49.389 1.00 0.00 H

ATOM 3605 CG LYS 220 53.668 58.326 50.311 1.00 0.00 C

ATOM 3606 HG2 LYS 220 54.347 58.585 49.498 1.00 0.00 H

ATOM 3607 HG3 LYS 220 53.287 59.311 50.581 1.00 0.00 H

ATOM 3608 CD LYS 220 54.384 57.656 51.533 1.00 0.00 C

ATOM 3609 HD2 LYS 220 53.677 57.783 52.352 1.00 0.00 H

ATOM 3610 HD3 LYS 220 54.458 56.576 51.402 1.00 0.00 H

ATOM 3611 CE LYS 220 55.791 58.179 51.926 1.00 0.00 C

ATOM 3612 HE2 LYS 220 55.585 59.242 52.059 1.00 0.00 H

ATOM 3613 HE3 LYS 220 56.141 57.704 52.842 1.00 0.00 H

ATOM 3614 NZ LYS 220 56.755 58.039 50.885 1.00 0.00 N

ATOM 3615 HZ1 LYS 220 57.359 58.832 51.045 1.00 0.00 H

ATOM 3616 HZ2 LYS 220 57.305 57.203 51.019 1.00 0.00 H

ATOM 3617 HZ3 LYS 220 56.379 58.064 49.948 1.00 0.00 H

ATOM 3618 C LYS 220 50.664 57.300 47.947 1.00 0.00 C

ATOM 3619 O LYS 220 50.945 56.766 46.900 1.00 0.00 O

ATOM 3620 N SER 221 49.536 57.074 48.642 1.00 0.00 N

ATOM 3621 H SER 221 49.449 57.490 49.559 1.00 0.00 H

ATOM 3622 CA SER 221 48.404 56.212 48.217 1.00 0.00 C

ATOM 3623 HA SER 221 48.755 55.207 47.980 1.00 0.00 H

ATOM 3624 CB SER 221 47.431 55.955 49.415 1.00 0.00 C

ATOM 3625 HB2 SER 221 47.375 56.889 49.973 1.00 0.00 H

ATOM 3626 HB3 SER 221 46.470 55.617 49.025 1.00 0.00 H

ATOM 3627 OG SER 221 47.926 54.908 50.185 1.00 0.00 O

ATOM 3628 HG SER 221 47.420 54.891 51.000 1.00 0.00 H

ATOM 3629 C SER 221 47.665 56.687 47.003 1.00 0.00 C

ATOM 3630 O SER 221 47.098 55.949 46.187 1.00 0.00 O

ATOM 3631 N ILE 222 47.765 57.995 46.698 1.00 0.00 N

ATOM 3632 H ILE 222 48.201 58.637 47.345 1.00 0.00 H

ATOM 3633 CA ILE 222 47.121 58.584 45.523 1.00 0.00 C

ATOM 3634 HA ILE 222 46.065 58.318 45.572 1.00 0.00 H

ATOM 3635 CB ILE 222 47.108 60.099 45.640 1.00 0.00 C

ATOM 3636 HB ILE 222 47.319 60.399 46.666 1.00 0.00 H

ATOM 3637 CG2 ILE 222 47.952 61.049 44.794 1.00 0.00 C

ATOM 3638 HG21 ILE 222 47.818 62.006 45.297 1.00 0.00 H

ATOM 3639 HG22 ILE 222 48.967 60.659 44.715 1.00 0.00 H

ATOM 3640 HG23 ILE 222 47.585 61.064 43.768 1.00 0.00 H

ATOM 3641 CG1 ILE 222 45.727 60.750 45.606 1.00 0.00 C

ATOM 3642 HG12 ILE 222 45.687 61.834 45.709 1.00 0.00 H

ATOM 3643 HG13 ILE 222 45.248 60.399 44.692 1.00 0.00 H

ATOM 3644 CD1 ILE 222 44.907 60.242 46.821 1.00 0.00 C

ATOM 3645 HD11 ILE 222 43.915 60.189 46.373 1.00 0.00 H

ATOM 3646 HD12 ILE 222 45.296 59.370 47.347 1.00 0.00 H

ATOM 3647 HD13 ILE 222 44.991 61.040 47.559 1.00 0.00 H

ATOM 3648 C ILE 222 47.767 58.213 44.183 1.00 0.00 C

ATOM 3649 O ILE 222 47.062 58.227 43.222 1.00 0.00 O

ATOM 3650 N TYR 223 49.042 57.788 44.251 1.00 0.00 N

ATOM 3651 H TYR 223 49.553 57.973 45.103 1.00 0.00 H

ATOM 3652 CA TYR 223 49.628 56.980 43.110 1.00 0.00 C

ATOM 3653 HA TYR 223 49.114 57.327 42.214 1.00 0.00 H

ATOM 3654 CB TYR 223 51.094 57.421 43.033 1.00 0.00 C

ATOM 3655 HB2 TYR 223 51.677 57.056 43.879 1.00 0.00 H

ATOM 3656 HB3 TYR 223 51.632 56.937 42.219 1.00 0.00 H

ATOM 3657 CG TYR 223 51.274 58.963 43.051 1.00 0.00 C

ATOM 3658 CD1 TYR 223 51.744 59.685 44.220 1.00 0.00 C

ATOM 3659 HD1 TYR 223 52.130 59.190 45.099 1.00 0.00 H

ATOM 3660 CE1 TYR 223 51.696 61.100 44.180 1.00 0.00 C

ATOM 3661 HE1 TYR 223 51.973 61.628 45.081 1.00 0.00 H

ATOM 3662 CZ TYR 223 51.285 61.794 43.017 1.00 0.00 C

ATOM 3663 OH TYR 223 51.501 63.102 42.895 1.00 0.00 O

ATOM 3664 HH TYR 223 52.003 63.400 43.657 1.00 0.00 H

ATOM 3665 CE2 TYR 223 50.839 61.008 41.895 1.00 0.00 C

ATOM 3666 HE2 TYR 223 50.562 61.529 40.991 1.00 0.00 H

ATOM 3667 CD2 TYR 223 50.843 59.657 41.912 1.00 0.00 C

ATOM 3668 HD2 TYR 223 50.459 59.038 41.115 1.00 0.00 H

ATOM 3669 C TYR 223 49.420 55.461 43.322 1.00 0.00 C

ATOM 3670 O TYR 223 48.947 54.694 42.498 1.00 0.00 O

ATOM 3671 N LEU 224 49.750 54.973 44.483 1.00 0.00 N

ATOM 3672 H LEU 224 50.069 55.632 45.179 1.00 0.00 H

ATOM 3673 CA LEU 224 49.978 53.525 44.829 1.00 0.00 C

ATOM 3674 HA LEU 224 50.618 53.038 44.093 1.00 0.00 H

ATOM 3675 CB LEU 224 50.508 53.447 46.224 1.00 0.00 C

ATOM 3676 HB2 LEU 224 50.114 54.254 46.842 1.00 0.00 H

ATOM 3677 HB3 LEU 224 50.244 52.473 46.636 1.00 0.00 H

ATOM 3678 CG LEU 224 52.055 53.578 46.241 1.00 0.00 C

ATOM 3679 HG LEU 224 52.266 54.456 45.632 1.00 0.00 H

ATOM 3680 CD1 LEU 224 52.536 53.657 47.689 1.00 0.00 C

ATOM 3681 HD11 LEU 224 52.308 54.652 48.071 1.00 0.00 H

ATOM 3682 HD12 LEU 224 51.926 52.907 48.194 1.00 0.00 H

ATOM 3683 HD13 LEU 224 53.623 53.613 47.741 1.00 0.00 H

ATOM 3684 CD2 LEU 224 52.753 52.348 45.597 1.00 0.00 C

ATOM 3685 HD21 LEU 224 53.798 52.305 45.903 1.00 0.00 H

ATOM 3686 HD22 LEU 224 52.214 51.455 45.912 1.00 0.00 H

ATOM 3687 HD23 LEU 224 52.792 52.499 44.519 1.00 0.00 H

ATOM 3688 C LEU 224 48.669 52.692 44.843 1.00 0.00 C

ATOM 3689 O LEU 224 48.704 51.482 44.756 1.00 0.00 O

ATOM 3690 N VAL 225 47.526 53.424 45.163 1.00 0.00 N

ATOM 3691 H VAL 225 47.713 54.417 45.183 1.00 0.00 H

ATOM 3692 CA VAL 225 46.160 52.903 45.276 1.00 0.00 C

ATOM 3693 HA VAL 225 46.027 51.828 45.153 1.00 0.00 H

ATOM 3694 CB VAL 225 45.637 53.062 46.770 1.00 0.00 C

ATOM 3695 HB VAL 225 45.415 54.117 46.933 1.00 0.00 H

ATOM 3696 CG1 VAL 225 44.392 52.236 46.975 1.00 0.00 C

ATOM 3697 HG11 VAL 225 44.647 51.197 46.771 1.00 0.00 H

ATOM 3698 HG12 VAL 225 44.057 52.503 47.978 1.00 0.00 H

ATOM 3699 HG13 VAL 225 43.613 52.602 46.307 1.00 0.00 H

ATOM 3700 CG2 VAL 225 46.625 52.612 47.789 1.00 0.00 C

ATOM 3701 HG21 VAL 225 46.783 51.551 47.597 1.00 0.00 H

ATOM 3702 HG22 VAL 225 47.638 53.009 47.860 1.00 0.00 H

ATOM 3703 HG23 VAL 225 46.218 52.570 48.799 1.00 0.00 H

ATOM 3704 C VAL 225 45.124 53.692 44.372 1.00 0.00 C

ATOM 3705 O VAL 225 44.194 53.004 44.049 1.00 0.00 O

ATOM 3706 N ILE 226 45.369 54.927 43.883 1.00 0.00 N

ATOM 3707 H ILE 226 46.173 55.403 44.266 1.00 0.00 H

ATOM 3708 CA ILE 226 44.295 55.681 43.201 1.00 0.00 C

ATOM 3709 HA ILE 226 43.368 55.109 43.152 1.00 0.00 H

ATOM 3710 CB ILE 226 43.920 56.997 43.919 1.00 0.00 C

ATOM 3711 HB ILE 226 44.846 57.538 44.119 1.00 0.00 H

ATOM 3712 CG2 ILE 226 43.103 57.946 42.948 1.00 0.00 C

ATOM 3713 HG21 ILE 226 42.137 57.558 42.624 1.00 0.00 H

ATOM 3714 HG22 ILE 226 42.880 58.852 43.512 1.00 0.00 H

ATOM 3715 HG23 ILE 226 43.623 58.266 42.045 1.00 0.00 H

ATOM 3716 CG1 ILE 226 43.345 56.811 45.334 1.00 0.00 C

ATOM 3717 HG12 ILE 226 44.087 56.416 46.027 1.00 0.00 H

ATOM 3718 HG13 ILE 226 43.287 57.786 45.819 1.00 0.00 H

ATOM 3719 CD1 ILE 226 42.032 56.094 45.399 1.00 0.00 C

ATOM 3720 HD11 ILE 226 42.116 55.014 45.283 1.00 0.00 H

ATOM 3721 HD12 ILE 226 41.433 56.342 46.276 1.00 0.00 H

ATOM 3722 HD13 ILE 226 41.465 56.599 44.617 1.00 0.00 H

ATOM 3723 C ILE 226 44.662 55.803 41.693 1.00 0.00 C

ATOM 3724 O ILE 226 43.965 55.214 40.856 1.00 0.00 O

ATOM 3725 N ILE 227 45.817 56.381 41.313 1.00 0.00 N

ATOM 3726 H ILE 227 46.456 56.611 42.061 1.00 0.00 H

ATOM 3727 CA ILE 227 46.138 56.723 39.934 1.00 0.00 C

ATOM 3728 HA ILE 227 45.259 57.230 39.535 1.00 0.00 H

ATOM 3729 CB ILE 227 47.290 57.765 39.801 1.00 0.00 C

ATOM 3730 HB ILE 227 47.992 57.722 40.634 1.00 0.00 H

ATOM 3731 CG2 ILE 227 48.179 57.690 38.527 1.00 0.00 C

ATOM 3732 HG21 ILE 227 47.585 57.638 37.614 1.00 0.00 H

ATOM 3733 HG22 ILE 227 48.764 58.601 38.401 1.00 0.00 H

ATOM 3734 HG23 ILE 227 48.719 56.743 38.533 1.00 0.00 H

ATOM 3735 CG1 ILE 227 46.767 59.246 39.851 1.00 0.00 C

ATOM 3736 HG12 ILE 227 46.272 59.411 38.894 1.00 0.00 H

ATOM 3737 HG13 ILE 227 45.936 59.217 40.554 1.00 0.00 H

ATOM 3738 CD1 ILE 227 47.703 60.290 40.337 1.00 0.00 C

ATOM 3739 HD11 ILE 227 48.632 60.156 39.784 1.00 0.00 H

ATOM 3740 HD12 ILE 227 47.330 61.313 40.274 1.00 0.00 H

ATOM 3741 HD13 ILE 227 47.964 60.113 41.380 1.00 0.00 H

ATOM 3742 C ILE 227 46.421 55.524 39.072 1.00 0.00 C

ATOM 3743 O ILE 227 45.947 55.510 37.946 1.00 0.00 O

ATOM 3744 N VAL 228 47.182 54.510 39.576 1.00 0.00 N

ATOM 3745 H VAL 228 47.828 54.757 40.312 1.00 0.00 H

ATOM 3746 CA VAL 228 47.216 53.121 39.068 1.00 0.00 C

ATOM 3747 HA VAL 228 47.980 53.099 38.290 1.00 0.00 H

ATOM 3748 CB VAL 228 47.779 52.065 40.028 1.00 0.00 C

ATOM 3749 HB VAL 228 48.832 52.297 40.189 1.00 0.00 H

ATOM 3750 CG1 VAL 228 47.122 52.140 41.421 1.00 0.00 C

ATOM 3751 HG11 VAL 228 47.899 51.794 42.103 1.00 0.00 H

ATOM 3752 HG12 VAL 228 46.953 53.168 41.742 1.00 0.00 H

ATOM 3753 HG13 VAL 228 46.363 51.363 41.515 1.00 0.00 H

ATOM 3754 CG2 VAL 228 47.867 50.690 39.419 1.00 0.00 C

ATOM 3755 HG21 VAL 228 48.180 50.480 38.396 1.00 0.00 H

ATOM 3756 HG22 VAL 228 48.509 50.033 40.006 1.00 0.00 H

ATOM 3757 HG23 VAL 228 46.829 50.360 39.383 1.00 0.00 H

ATOM 3758 C VAL 228 45.912 52.619 38.407 1.00 0.00 C

ATOM 3759 O VAL 228 45.805 52.266 37.196 1.00 0.00 O

ATOM 3760 N LEU 229 44.743 52.579 39.120 1.00 0.00 N

ATOM 3761 H LEU 229 44.648 52.786 40.104 1.00 0.00 H

ATOM 3762 CA LEU 229 43.602 51.760 38.674 1.00 0.00 C

ATOM 3763 HA LEU 229 44.078 50.841 38.334 1.00 0.00 H

ATOM 3764 CB LEU 229 42.618 51.358 39.869 1.00 0.00 C

ATOM 3765 HB2 LEU 229 42.669 52.096 40.669 1.00 0.00 H

ATOM 3766 HB3 LEU 229 41.620 51.503 39.456 1.00 0.00 H

ATOM 3767 CG LEU 229 42.631 49.895 40.339 1.00 0.00 C

ATOM 3768 HG LEU 229 42.122 49.970 41.300 1.00 0.00 H

ATOM 3769 CD1 LEU 229 41.730 48.961 39.529 1.00 0.00 C

ATOM 3770 HD11 LEU 229 41.840 47.924 39.844 1.00 0.00 H

ATOM 3771 HD12 LEU 229 40.717 49.338 39.668 1.00 0.00 H

ATOM 3772 HD13 LEU 229 42.080 48.890 38.500 1.00 0.00 H

ATOM 3773 CD2 LEU 229 44.050 49.378 40.685 1.00 0.00 C

ATOM 3774 HD21 LEU 229 44.747 49.384 39.847 1.00 0.00 H

ATOM 3775 HD22 LEU 229 44.488 49.927 41.518 1.00 0.00 H

ATOM 3776 HD23 LEU 229 43.922 48.369 41.077 1.00 0.00 H

ATOM 3777 C LEU 229 42.823 52.652 37.577 1.00 0.00 C

ATOM 3778 O LEU 229 42.289 52.071 36.652 1.00 0.00 O

ATOM 3779 N THR 230 42.838 53.968 37.647 1.00 0.00 N

ATOM 3780 H THR 230 43.416 54.176 38.449 1.00 0.00 H

ATOM 3781 CA THR 230 42.495 54.912 36.538 1.00 0.00 C

ATOM 3782 HA THR 230 41.427 54.739 36.406 1.00 0.00 H

ATOM 3783 CB THR 230 42.400 56.367 36.951 1.00 0.00 C

ATOM 3784 HB THR 230 41.848 56.849 36.144 1.00 0.00 H

ATOM 3785 CG2 THR 230 41.535 56.687 38.194 1.00 0.00 C

ATOM 3786 HG21 THR 230 42.161 56.382 39.032 1.00 0.00 H

ATOM 3787 HG22 THR 230 41.397 57.748 38.404 1.00 0.00 H

ATOM 3788 HG23 THR 230 40.502 56.339 38.170 1.00 0.00 H

ATOM 3789 OG1 THR 230 43.650 56.987 37.149 1.00 0.00 O

ATOM 3790 HG1 THR 230 44.173 56.446 37.745 1.00 0.00 H

ATOM 3791 C THR 230 43.252 54.623 35.232 1.00 0.00 C

ATOM 3792 O THR 230 42.715 54.290 34.156 1.00 0.00 O

ATOM 3793 N VAL 231 44.592 54.610 35.406 1.00 0.00 N

ATOM 3794 H VAL 231 45.052 54.908 36.255 1.00 0.00 H

ATOM 3795 CA VAL 231 45.420 54.410 34.196 1.00 0.00 C

ATOM 3796 HA VAL 231 45.023 55.004 33.373 1.00 0.00 H

ATOM 3797 CB VAL 231 46.864 54.876 34.388 1.00 0.00 C

ATOM 3798 HB VAL 231 47.306 54.773 33.397 1.00 0.00 H

ATOM 3799 CG1 VAL 231 46.963 56.417 34.585 1.00 0.00 C

ATOM 3800 HG11 VAL 231 47.988 56.787 34.614 1.00 0.00 H

ATOM 3801 HG12 VAL 231 46.394 56.909 33.796 1.00 0.00 H

ATOM 3802 HG13 VAL 231 46.579 56.640 35.580 1.00 0.00 H

ATOM 3803 CG2 VAL 231 47.771 54.363 35.512 1.00 0.00 C

ATOM 3804 HG21 VAL 231 47.762 53.280 35.636 1.00 0.00 H

ATOM 3805 HG22 VAL 231 48.803 54.569 35.227 1.00 0.00 H

ATOM 3806 HG23 VAL 231 47.645 54.857 36.475 1.00 0.00 H

ATOM 3807 C VAL 231 45.307 52.953 33.677 1.00 0.00 C

ATOM 3808 O VAL 231 45.263 52.817 32.448 1.00 0.00 O

ATOM 3809 N PHE 232 44.988 51.960 34.516 1.00 0.00 N

ATOM 3810 H PHE 232 44.954 52.156 35.506 1.00 0.00 H

ATOM 3811 CA PHE 232 44.748 50.595 34.053 1.00 0.00 C

ATOM 3812 HA PHE 232 45.574 50.422 33.362 1.00 0.00 H

ATOM 3813 CB PHE 232 44.865 49.566 35.171 1.00 0.00 C

ATOM 3814 HB2 PHE 232 45.642 49.831 35.887 1.00 0.00 H

ATOM 3815 HB3 PHE 232 44.108 49.697 35.945 1.00 0.00 H

ATOM 3816 CG PHE 232 44.850 48.141 34.869 1.00 0.00 C

ATOM 3817 CD1 PHE 232 46.019 47.350 34.927 1.00 0.00 C

ATOM 3818 HD1 PHE 232 47.001 47.781 35.057 1.00 0.00 H

ATOM 3819 CE1 PHE 232 45.993 46.116 34.269 1.00 0.00 C

ATOM 3820 HE1 PHE 232 46.855 45.478 34.400 1.00 0.00 H

ATOM 3821 CZ PHE 232 44.744 45.484 33.999 1.00 0.00 C

ATOM 3822 HZ PHE 232 44.720 44.544 33.468 1.00 0.00 H

ATOM 3823 CE2 PHE 232 43.581 46.275 34.093 1.00 0.00 C

ATOM 3824 HE2 PHE 232 42.647 45.804 33.826 1.00 0.00 H

ATOM 3825 CD2 PHE 232 43.619 47.606 34.500 1.00 0.00 C

ATOM 3826 HD2 PHE 232 42.697 48.168 34.518 1.00 0.00 H

ATOM 3827 C PHE 232 43.553 50.463 33.186 1.00 0.00 C

ATOM 3828 O PHE 232 43.433 49.825 32.152 1.00 0.00 O

ATOM 3829 N ALA 233 42.464 51.021 33.761 1.00 0.00 N

ATOM 3830 H ALA 233 42.605 51.508 34.634 1.00 0.00 H

ATOM 3831 CA ALA 233 41.198 51.200 33.185 1.00 0.00 C

ATOM 3832 HA ALA 233 40.782 50.195 33.117 1.00 0.00 H

ATOM 3833 CB ALA 233 40.291 51.822 34.254 1.00 0.00 C

ATOM 3834 HB1 ALA 233 40.363 51.291 35.204 1.00 0.00 H

ATOM 3835 HB2 ALA 233 40.476 52.896 34.243 1.00 0.00 H

ATOM 3836 HB3 ALA 233 39.278 51.562 33.950 1.00 0.00 H

ATOM 3837 C ALA 233 41.067 51.879 31.848 1.00 0.00 C

ATOM 3838 O ALA 233 40.701 51.279 30.830 1.00 0.00 O

ATOM 3839 N VAL 234 41.769 53.040 31.768 1.00 0.00 N

ATOM 3840 H VAL 234 42.083 53.457 32.632 1.00 0.00 H

ATOM 3841 CA VAL 234 41.847 53.796 30.484 1.00 0.00 C

ATOM 3842 HA VAL 234 40.816 53.869 30.138 1.00 0.00 H

ATOM 3843 CB VAL 234 42.318 55.202 30.767 1.00 0.00 C

ATOM 3844 HB VAL 234 43.246 55.178 31.338 1.00 0.00 H

ATOM 3845 CG1 VAL 234 42.719 56.029 29.455 1.00 0.00 C

ATOM 3846 HG11 VAL 234 43.444 55.485 28.850 1.00 0.00 H

ATOM 3847 HG12 VAL 234 41.894 56.237 28.774 1.00 0.00 H

ATOM 3848 HG13 VAL 234 43.260 56.952 29.664 1.00 0.00 H

ATOM 3849 CG2 VAL 234 41.259 56.116 31.395 1.00 0.00 C

ATOM 3850 HG21 VAL 234 40.271 55.840 31.025 1.00 0.00 H

ATOM 3851 HG22 VAL 234 41.382 55.909 32.458 1.00 0.00 H

ATOM 3852 HG23 VAL 234 41.578 57.145 31.229 1.00 0.00 H

ATOM 3853 C VAL 234 42.685 53.036 29.453 1.00 0.00 C

ATOM 3854 O VAL 234 42.386 53.090 28.268 1.00 0.00 O

ATOM 3855 N SER 235 43.847 52.399 29.797 1.00 0.00 N

ATOM 3856 H SER 235 44.028 52.402 30.791 1.00 0.00 H

ATOM 3857 CA SER 235 44.778 51.600 28.954 1.00 0.00 C

ATOM 3858 HA SER 235 44.992 52.364 28.207 1.00 0.00 H

ATOM 3859 CB SER 235 46.061 51.071 29.608 1.00 0.00 C

ATOM 3860 HB2 SER 235 45.753 50.408 30.417 1.00 0.00 H

ATOM 3861 HB3 SER 235 46.667 50.699 28.781 1.00 0.00 H

ATOM 3862 OG SER 235 46.804 52.100 30.098 1.00 0.00 O

ATOM 3863 HG SER 235 46.313 52.483 30.828 1.00 0.00 H

ATOM 3864 C SER 235 44.216 50.353 28.269 1.00 0.00 C

ATOM 3865 O SER 235 44.330 50.228 26.999 1.00 0.00 O

ATOM 3866 N TYR 236 43.344 49.538 28.933 1.00 0.00 N

ATOM 3867 H TYR 236 43.039 49.809 29.857 1.00 0.00 H

ATOM 3868 CA TYR 236 42.933 48.205 28.498 1.00 0.00 C

ATOM 3869 HA TYR 236 43.281 47.958 27.495 1.00 0.00 H

ATOM 3870 CB TYR 236 43.606 47.173 29.423 1.00 0.00 C

ATOM 3871 HB2 TYR 236 43.105 46.818 30.324 1.00 0.00 H

ATOM 3872 HB3 TYR 236 43.566 46.271 28.813 1.00 0.00 H

ATOM 3873 CG TYR 236 45.135 47.303 29.704 1.00 0.00 C

ATOM 3874 CD1 TYR 236 46.025 47.085 28.631 1.00 0.00 C

ATOM 3875 HD1 TYR 236 45.592 46.733 27.706 1.00 0.00 H

ATOM 3876 CE1 TYR 236 47.412 47.249 28.824 1.00 0.00 C

ATOM 3877 HE1 TYR 236 48.095 47.328 27.992 1.00 0.00 H

ATOM 3878 CZ TYR 236 47.865 47.489 30.096 1.00 0.00 C

ATOM 3879 OH TYR 236 49.237 47.211 30.283 1.00 0.00 O

ATOM 3880 HH TYR 236 49.726 47.488 29.505 1.00 0.00 H

ATOM 3881 CE2 TYR 236 46.988 47.639 31.186 1.00 0.00 C

ATOM 3882 HE2 TYR 236 47.393 47.971 32.130 1.00 0.00 H

ATOM 3883 CD2 TYR 236 45.584 47.631 30.997 1.00 0.00 C

ATOM 3884 HD2 TYR 236 44.951 47.797 31.856 1.00 0.00 H

ATOM 3885 C TYR 236 41.453 47.924 28.302 1.00 0.00 C

ATOM 3886 O TYR 236 41.111 46.991 27.522 1.00 0.00 O

ATOM 3887 N ILE 237 40.571 48.725 28.905 1.00 0.00 N

ATOM 3888 H ILE 237 40.921 49.199 29.725 1.00 0.00 H

ATOM 3889 CA ILE 237 39.072 48.502 28.761 1.00 0.00 C

ATOM 3890 HA ILE 237 38.959 47.421 28.839 1.00 0.00 H

ATOM 3891 CB ILE 237 38.268 49.053 29.963 1.00 0.00 C

ATOM 3892 HB ILE 237 38.715 50.034 30.124 1.00 0.00 H

ATOM 3893 CG2 ILE 237 36.792 49.282 29.754 1.00 0.00 C

ATOM 3894 HG21 ILE 237 36.407 49.744 30.663 1.00 0.00 H

ATOM 3895 HG22 ILE 237 36.627 49.984 28.936 1.00 0.00 H

ATOM 3896 HG23 ILE 237 36.325 48.324 29.530 1.00 0.00 H

ATOM 3897 CG1 ILE 237 38.594 48.317 31.298 1.00 0.00 C

ATOM 3898 HG12 ILE 237 38.166 47.316 31.358 1.00 0.00 H

ATOM 3899 HG13 ILE 237 39.666 48.122 31.309 1.00 0.00 H

ATOM 3900 CD1 ILE 237 38.273 49.160 32.557 1.00 0.00 C

ATOM 3901 HD11 ILE 237 38.966 48.892 33.355 1.00 0.00 H

ATOM 3902 HD12 ILE 237 38.305 50.245 32.456 1.00 0.00 H

ATOM 3903 HD13 ILE 237 37.316 48.804 32.938 1.00 0.00 H

ATOM 3904 C ILE 237 38.513 48.803 27.359 1.00 0.00 C

ATOM 3905 O ILE 237 37.903 47.909 26.793 1.00 0.00 O

ATOM 3906 N PRO 238 38.841 49.971 26.771 1.00 0.00 N

ATOM 3907 CD PRO 238 39.557 51.002 27.397 1.00 0.00 C

ATOM 3908 HD2 PRO 238 40.631 50.818 27.394 1.00 0.00 H

ATOM 3909 HD3 PRO 238 39.151 51.229 28.384 1.00 0.00 H

ATOM 3910 CG PRO 238 39.286 52.235 26.517 1.00 0.00 C

ATOM 3911 HG2 PRO 238 40.274 52.637 26.293 1.00 0.00 H

ATOM 3912 HG3 PRO 238 38.776 53.014 27.084 1.00 0.00 H

ATOM 3913 CB PRO 238 38.417 51.789 25.335 1.00 0.00 C

ATOM 3914 HB2 PRO 238 38.971 51.909 24.404 1.00 0.00 H

ATOM 3915 HB3 PRO 238 37.473 52.334 25.342 1.00 0.00 H

ATOM 3916 CA PRO 238 38.314 50.298 25.448 1.00 0.00 C

ATOM 3917 HA PRO 238 37.277 49.964 25.417 1.00 0.00 H

ATOM 3918 C PRO 238 39.039 49.522 24.321 1.00 0.00 C

ATOM 3919 O PRO 238 38.380 49.147 23.376 1.00 0.00 O

ATOM 3920 N PHE 239 40.256 49.121 24.591 1.00 0.00 N

ATOM 3921 H PHE 239 40.647 49.502 25.441 1.00 0.00 H

ATOM 3922 CA PHE 239 41.044 48.242 23.700 1.00 0.00 C

ATOM 3923 HA PHE 239 41.242 48.679 22.721 1.00 0.00 H

ATOM 3924 CB PHE 239 42.474 48.106 24.308 1.00 0.00 C

ATOM 3925 HB2 PHE 239 42.958 49.080 24.232 1.00 0.00 H

ATOM 3926 HB3 PHE 239 42.409 47.936 25.383 1.00 0.00 H

ATOM 3927 CG PHE 239 43.267 46.960 23.684 1.00 0.00 C

ATOM 3928 CD1 PHE 239 43.393 45.698 24.358 1.00 0.00 C

ATOM 3929 HD1 PHE 239 43.042 45.492 25.359 1.00 0.00 H

ATOM 3930 CE1 PHE 239 44.012 44.695 23.603 1.00 0.00 C

ATOM 3931 HE1 PHE 239 44.125 43.703 24.013 1.00 0.00 H

ATOM 3932 CZ PHE 239 44.633 44.867 22.366 1.00 0.00 C

ATOM 3933 HZ PHE 239 45.256 44.095 21.940 1.00 0.00 H

ATOM 3934 CE2 PHE 239 44.597 46.121 21.734 1.00 0.00 C

ATOM 3935 HE2 PHE 239 45.299 46.387 20.959 1.00 0.00 H

ATOM 3936 CD2 PHE 239 43.839 47.120 22.370 1.00 0.00 C

ATOM 3937 HD2 PHE 239 43.995 48.096 21.936 1.00 0.00 H

ATOM 3938 C PHE 239 40.397 46.951 23.468 1.00 0.00 C

ATOM 3939 O PHE 239 40.286 46.512 22.359 1.00 0.00 O

ATOM 3940 N HID 240 39.986 46.202 24.523 1.00 0.00 N

ATOM 3941 H HID 240 40.181 46.574 25.442 1.00 0.00 H

ATOM 3942 CA HID 240 39.313 44.984 24.433 1.00 0.00 C

ATOM 3943 HA HID 240 39.976 44.340 23.855 1.00 0.00 H

ATOM 3944 CB HID 240 39.267 44.345 25.844 1.00 0.00 C

ATOM 3945 HB2 HID 240 39.009 45.108 26.579 1.00 0.00 H

ATOM 3946 HB3 HID 240 38.448 43.626 25.858 1.00 0.00 H

ATOM 3947 CG HID 240 40.545 43.668 26.338 1.00 0.00 C

ATOM 3948 ND1 HID 240 41.301 44.147 27.394 1.00 0.00 N

ATOM 3949 HD1 HID 240 41.126 45.015 27.880 1.00 0.00 H

ATOM 3950 CE1 HID 240 42.303 43.238 27.631 1.00 0.00 C

ATOM 3951 HE1 HID 240 43.067 43.300 28.392 1.00 0.00 H

ATOM 3952 NE2 HID 240 42.111 42.205 26.744 1.00 0.00 N

ATOM 3953 CD2 HID 240 40.918 42.433 26.077 1.00 0.00 C

ATOM 3954 HD2 HID 240 40.553 41.747 25.327 1.00 0.00 H

ATOM 3955 C HID 240 37.984 45.029 23.740 1.00 0.00 C

ATOM 3956 O HID 240 37.667 44.133 22.960 1.00 0.00 O

ATOM 3957 N VAL 241 37.145 46.015 24.019 1.00 0.00 N

ATOM 3958 H VAL 241 37.383 46.724 24.697 1.00 0.00 H

ATOM 3959 CA VAL 241 35.980 46.432 23.182 1.00 0.00 C

ATOM 3960 HA VAL 241 35.237 45.650 23.339 1.00 0.00 H

ATOM 3961 CB VAL 241 35.397 47.670 23.787 1.00 0.00 C

ATOM 3962 HB VAL 241 36.225 48.304 24.104 1.00 0.00 H

ATOM 3963 CG1 VAL 241 34.487 48.494 22.804 1.00 0.00 C

ATOM 3964 HG11 VAL 241 33.691 47.785 22.573 1.00 0.00 H

ATOM 3965 HG12 VAL 241 34.054 49.373 23.281 1.00 0.00 H

ATOM 3966 HG13 VAL 241 35.033 48.735 21.892 1.00 0.00 H

ATOM 3967 CG2 VAL 241 34.795 47.351 25.162 1.00 0.00 C

ATOM 3968 HG21 VAL 241 33.870 46.787 25.037 1.00 0.00 H

ATOM 3969 HG22 VAL 241 35.518 46.825 25.785 1.00 0.00 H

ATOM 3970 HG23 VAL 241 34.564 48.318 25.609 1.00 0.00 H

ATOM 3971 C VAL 241 36.346 46.489 21.720 1.00 0.00 C

ATOM 3972 O VAL 241 35.680 45.763 20.987 1.00 0.00 O

ATOM 3973 N MET 242 37.441 47.243 21.339 1.00 0.00 N

ATOM 3974 H MET 242 38.037 47.633 22.056 1.00 0.00 H

ATOM 3975 CA MET 242 37.738 47.551 19.931 1.00 0.00 C

ATOM 3976 HA MET 242 36.777 47.828 19.498 1.00 0.00 H

ATOM 3977 CB MET 242 38.664 48.773 19.746 1.00 0.00 C

ATOM 3978 HB2 MET 242 38.705 49.314 20.692 1.00 0.00 H

ATOM 3979 HB3 MET 242 39.705 48.474 19.622 1.00 0.00 H

ATOM 3980 CG MET 242 38.248 49.737 18.644 1.00 0.00 C

ATOM 3981 HG2 MET 242 38.859 50.621 18.826 1.00 0.00 H

ATOM 3982 HG3 MET 242 38.419 49.273 17.673 1.00 0.00 H

ATOM 3983 SD MET 242 36.574 50.244 18.753 1.00 0.00 S

ATOM 3984 CE MET 242 36.409 51.235 20.309 1.00 0.00 C

ATOM 3985 HE1 MET 242 35.415 51.169 20.752 1.00 0.00 H

ATOM 3986 HE2 MET 242 37.126 50.726 20.952 1.00 0.00 H

ATOM 3987 HE3 MET 242 36.688 52.275 20.140 1.00 0.00 H

ATOM 3988 C MET 242 38.304 46.268 19.312 1.00 0.00 C

ATOM 3989 O MET 242 37.912 45.898 18.150 1.00 0.00 O

ATOM 3990 N LYS 243 39.115 45.481 20.008 1.00 0.00 N

ATOM 3991 H LYS 243 39.507 45.788 20.887 1.00 0.00 H

ATOM 3992 CA LYS 243 39.452 44.037 19.713 1.00 0.00 C

ATOM 3993 HA LYS 243 40.113 43.941 18.852 1.00 0.00 H

ATOM 3994 CB LYS 243 40.269 43.320 20.851 1.00 0.00 C

ATOM 3995 HB2 LYS 243 41.191 43.837 21.115 1.00 0.00 H

ATOM 3996 HB3 LYS 243 39.592 43.184 21.695 1.00 0.00 H

ATOM 3997 CG LYS 243 40.729 41.908 20.417 1.00 0.00 C

ATOM 3998 HG2 LYS 243 39.833 41.339 20.170 1.00 0.00 H

ATOM 3999 HG3 LYS 243 41.311 41.987 19.499 1.00 0.00 H

ATOM 4000 CD LYS 243 41.466 41.102 21.501 1.00 0.00 C

ATOM 4001 HD2 LYS 243 41.113 41.359 22.500 1.00 0.00 H

ATOM 4002 HD3 LYS 243 41.287 40.041 21.329 1.00 0.00 H

ATOM 4003 CE LYS 243 42.904 41.462 21.437 1.00 0.00 C

ATOM 4004 HE2 LYS 243 43.171 41.652 20.397 1.00 0.00 H

ATOM 4005 HE3 LYS 243 43.096 42.377 21.997 1.00 0.00 H

ATOM 4006 NZ LYS 243 43.819 40.358 22.031 1.00 0.00 N

ATOM 4007 HZ1 LYS 243 44.790 40.625 21.951 1.00 0.00 H

ATOM 4008 HZ2 LYS 243 43.604 40.163 22.998 1.00 0.00 H

ATOM 4009 HZ3 LYS 243 43.545 39.472 21.632 1.00 0.00 H

ATOM 4010 C LYS 243 38.259 43.228 19.330 1.00 0.00 C

ATOM 4011 O LYS 243 38.202 42.620 18.227 1.00 0.00 O

ATOM 4012 N THR 244 37.279 43.087 20.334 1.00 0.00 N

ATOM 4013 H THR 244 37.402 43.746 21.089 1.00 0.00 H

ATOM 4014 CA THR 244 36.003 42.389 20.136 1.00 0.00 C

ATOM 4015 HA THR 244 36.266 41.353 19.920 1.00 0.00 H

ATOM 4016 CB THR 244 35.071 42.542 21.345 1.00 0.00 C

ATOM 4017 HB THR 244 35.076 43.599 21.613 1.00 0.00 H

ATOM 4018 CG2 THR 244 33.661 42.082 21.228 1.00 0.00 C

ATOM 4019 HG21 THR 244 33.080 42.802 20.651 1.00 0.00 H

ATOM 4020 HG22 THR 244 33.439 41.097 20.819 1.00 0.00 H

ATOM 4021 HG23 THR 244 33.259 42.244 22.228 1.00 0.00 H

ATOM 4022 OG1 THR 244 35.485 41.831 22.413 1.00 0.00 O

ATOM 4023 HG1 THR 244 36.342 41.473 22.169 1.00 0.00 H

ATOM 4024 C THR 244 35.194 42.785 18.881 1.00 0.00 C

ATOM 4025 O THR 244 34.666 41.906 18.208 1.00 0.00 O

ATOM 4026 N MET 245 35.256 44.044 18.500 1.00 0.00 N

ATOM 4027 H MET 245 35.693 44.676 19.156 1.00 0.00 H

ATOM 4028 CA MET 245 34.707 44.536 17.248 1.00 0.00 C

ATOM 4029 HA MET 245 33.800 43.969 17.034 1.00 0.00 H

ATOM 4030 CB MET 245 34.173 46.000 17.345 1.00 0.00 C

ATOM 4031 HB2 MET 245 34.501 46.357 18.321 1.00 0.00 H

ATOM 4032 HB3 MET 245 34.753 46.557 16.609 1.00 0.00 H

ATOM 4033 CG MET 245 32.598 46.271 17.191 1.00 0.00 C

ATOM 4034 HG2 MET 245 31.996 45.363 17.196 1.00 0.00 H

ATOM 4035 HG3 MET 245 32.239 46.803 18.071 1.00 0.00 H

ATOM 4036 SD MET 245 32.316 47.381 15.752 1.00 0.00 S

ATOM 4037 CE MET 245 32.827 46.480 14.342 1.00 0.00 C

ATOM 4038 HE1 MET 245 32.101 45.677 14.216 1.00 0.00 H

ATOM 4039 HE2 MET 245 32.872 47.164 13.494 1.00 0.00 H

ATOM 4040 HE3 MET 245 33.756 45.952 14.560 1.00 0.00 H

ATOM 4041 C MET 245 35.525 44.314 15.942 1.00 0.00 C

ATOM 4042 O MET 245 35.001 43.821 14.934 1.00 0.00 O

ATOM 4043 N ASN 246 36.824 44.631 15.901 1.00 0.00 N

ATOM 4044 H ASN 246 37.295 45.049 16.690 1.00 0.00 H

ATOM 4045 CA ASN 246 37.703 44.272 14.760 1.00 0.00 C

ATOM 4046 HA ASN 246 37.418 44.852 13.882 1.00 0.00 H

ATOM 4047 CB ASN 246 39.173 44.742 15.071 1.00 0.00 C

ATOM 4048 HB2 ASN 246 39.215 45.830 15.108 1.00 0.00 H

ATOM 4049 HB3 ASN 246 39.364 44.546 16.126 1.00 0.00 H

ATOM 4050 CG ASN 246 40.414 44.225 14.329 1.00 0.00 C

ATOM 4051 OD1 ASN 246 41.026 43.202 14.557 1.00 0.00 O

ATOM 4052 ND2 ASN 246 40.690 44.900 13.189 1.00 0.00 N

ATOM 4053 HD21 ASN 246 41.663 44.957 12.924 1.00 0.00 H

ATOM 4054 HD22 ASN 246 40.099 45.572 12.721 1.00 0.00 H

ATOM 4055 C ASN 246 37.701 42.790 14.207 1.00 0.00 C

ATOM 4056 O ASN 246 37.434 42.566 13.018 1.00 0.00 O

ATOM 4057 N LEU 247 37.984 41.766 15.057 1.00 0.00 N

ATOM 4058 H LEU 247 37.924 42.002 16.037 1.00 0.00 H

ATOM 4059 CA LEU 247 37.773 40.333 14.595 1.00 0.00 C

ATOM 4060 HA LEU 247 38.616 40.118 13.938 1.00 0.00 H

ATOM 4061 CB LEU 247 38.002 39.348 15.766 1.00 0.00 C

ATOM 4062 HB2 LEU 247 37.221 39.478 16.515 1.00 0.00 H

ATOM 4063 HB3 LEU 247 38.041 38.360 15.308 1.00 0.00 H

ATOM 4064 CG LEU 247 39.308 39.530 16.556 1.00 0.00 C

ATOM 4065 HG LEU 247 39.290 40.459 17.126 1.00 0.00 H

ATOM 4066 CD1 LEU 247 39.477 38.470 17.724 1.00 0.00 C

ATOM 4067 HD11 LEU 247 38.631 38.599 18.399 1.00 0.00 H

ATOM 4068 HD12 LEU 247 39.639 37.443 17.397 1.00 0.00 H

ATOM 4069 HD13 LEU 247 40.308 38.638 18.409 1.00 0.00 H

ATOM 4070 CD2 LEU 247 40.424 39.392 15.470 1.00 0.00 C

ATOM 4071 HD21 LEU 247 40.495 40.325 14.910 1.00 0.00 H

ATOM 4072 HD22 LEU 247 41.419 39.228 15.883 1.00 0.00 H

ATOM 4073 HD23 LEU 247 40.208 38.478 14.917 1.00 0.00 H

ATOM 4074 C LEU 247 36.449 40.050 13.966 1.00 0.00 C

ATOM 4075 O LEU 247 36.474 39.235 13.024 1.00 0.00 O

ATOM 4076 N ARG 248 35.350 40.674 14.384 1.00 0.00 N

ATOM 4077 H ARG 248 35.368 41.177 15.259 1.00 0.00 H

ATOM 4078 CA ARG 248 34.042 40.543 13.656 1.00 0.00 C

ATOM 4079 HA ARG 248 33.856 39.514 13.351 1.00 0.00 H

ATOM 4080 CB ARG 248 32.819 41.038 14.400 1.00 0.00 C

ATOM 4081 HB2 ARG 248 32.687 40.322 15.211 1.00 0.00 H

ATOM 4082 HB3 ARG 248 32.981 42.049 14.776 1.00 0.00 H

ATOM 4083 CG ARG 248 31.438 41.077 13.738 1.00 0.00 C

ATOM 4084 HG2 ARG 248 30.770 41.316 14.566 1.00 0.00 H

ATOM 4085 HG3 ARG 248 31.510 41.815 12.939 1.00 0.00 H

ATOM 4086 CD ARG 248 31.098 39.695 13.207 1.00 0.00 C

ATOM 4087 HD2 ARG 248 31.542 39.588 12.217 1.00 0.00 H

ATOM 4088 HD3 ARG 248 31.347 38.832 13.825 1.00 0.00 H

ATOM 4089 NE ARG 248 29.628 39.664 13.060 1.00 0.00 N

ATOM 4090 HE ARG 248 29.077 40.494 12.895 1.00 0.00 H

ATOM 4091 CZ ARG 248 28.928 38.542 13.013 1.00 0.00 C

ATOM 4092 NH1 ARG 248 27.603 38.648 12.946 1.00 0.00 N

ATOM 4093 HH11 ARG 248 27.304 39.540 12.578 1.00 0.00 H

ATOM 4094 HH12 ARG 248 26.963 37.871 13.028 1.00 0.00 H

ATOM 4095 NH2 ARG 248 29.352 37.329 13.323 1.00 0.00 N

ATOM 4096 HH21 ARG 248 30.348 37.163 13.278 1.00 0.00 H

ATOM 4097 HH22 ARG 248 28.702 36.567 13.194 1.00 0.00 H

ATOM 4098 C ARG 248 34.029 41.311 12.358 1.00 0.00 C

ATOM 4099 O ARG 248 33.403 40.761 11.468 1.00 0.00 O

ATOM 4100 N ALA 249 34.461 42.515 12.199 1.00 0.00 N

ATOM 4101 H ALA 249 34.696 42.989 13.059 1.00 0.00 H

ATOM 4102 CA ALA 249 34.540 43.217 10.982 1.00 0.00 C

ATOM 4103 HA ALA 249 33.614 43.121 10.415 1.00 0.00 H

ATOM 4104 CB ALA 249 34.919 44.671 11.370 1.00 0.00 C

ATOM 4105 HB1 ALA 249 34.349 45.159 12.160 1.00 0.00 H

ATOM 4106 HB2 ALA 249 35.991 44.690 11.565 1.00 0.00 H

ATOM 4107 HB3 ALA 249 34.710 45.276 10.487 1.00 0.00 H

ATOM 4108 C ALA 249 35.590 42.644 9.943 1.00 0.00 C

ATOM 4109 O ALA 249 35.332 42.675 8.678 1.00 0.00 O

ATOM 4110 N ARG 250 36.680 42.125 10.414 1.00 0.00 N

ATOM 4111 H ARG 250 36.829 42.184 11.411 1.00 0.00 H

ATOM 4112 CA ARG 250 37.635 41.361 9.646 1.00 0.00 C

ATOM 4113 HA ARG 250 37.927 41.911 8.752 1.00 0.00 H

ATOM 4114 CB ARG 250 38.843 40.799 10.401 1.00 0.00 C

ATOM 4115 HB2 ARG 250 38.549 40.247 11.294 1.00 0.00 H

ATOM 4116 HB3 ARG 250 39.296 40.057 9.744 1.00 0.00 H

ATOM 4117 CG ARG 250 39.957 41.810 10.739 1.00 0.00 C

ATOM 4118 HG2 ARG 250 40.329 42.395 9.897 1.00 0.00 H

ATOM 4119 HG3 ARG 250 39.518 42.452 11.503 1.00 0.00 H

ATOM 4120 CD ARG 250 41.304 41.125 11.176 1.00 0.00 C

ATOM 4121 HD2 ARG 250 41.185 40.055 11.343 1.00 0.00 H

ATOM 4122 HD3 ARG 250 41.913 41.332 10.297 1.00 0.00 H

ATOM 4123 NE ARG 250 41.899 41.751 12.299 1.00 0.00 N

ATOM 4124 HE ARG 250 41.372 42.358 12.910 1.00 0.00 H

ATOM 4125 CZ ARG 250 43.110 41.505 12.703 1.00 0.00 C

ATOM 4126 NH1 ARG 250 43.490 42.130 13.776 1.00 0.00 N

ATOM 4127 HH11 ARG 250 44.267 41.724 14.276 1.00 0.00 H

ATOM 4128 HH12 ARG 250 42.771 42.604 14.304 1.00 0.00 H

ATOM 4129 NH2 ARG 250 43.873 40.695 12.083 1.00 0.00 N

ATOM 4130 HH21 ARG 250 43.672 40.561 11.103 1.00 0.00 H

ATOM 4131 HH22 ARG 250 44.815 40.585 12.431 1.00 0.00 H

ATOM 4132 C ARG 250 37.016 40.173 8.922 1.00 0.00 C

ATOM 4133 O ARG 250 37.394 39.771 7.799 1.00 0.00 O

ATOM 4134 N LEU 251 35.956 39.667 9.581 1.00 0.00 N

ATOM 4135 H LEU 251 35.629 40.170 10.394 1.00 0.00 H

ATOM 4136 CA LEU 251 34.956 38.800 8.917 1.00 0.00 C

ATOM 4137 HA LEU 251 35.566 38.121 8.321 1.00 0.00 H

ATOM 4138 CB LEU 251 34.248 37.931 9.985 1.00 0.00 C

ATOM 4139 HB2 LEU 251 34.865 37.685 10.849 1.00 0.00 H

ATOM 4140 HB3 LEU 251 33.446 38.575 10.348 1.00 0.00 H

ATOM 4141 CG LEU 251 33.762 36.462 9.655 1.00 0.00 C

ATOM 4142 HG LEU 251 33.133 35.962 10.392 1.00 0.00 H

ATOM 4143 CD1 LEU 251 32.900 36.336 8.437 1.00 0.00 C

ATOM 4144 HD11 LEU 251 33.169 37.006 7.620 1.00 0.00 H

ATOM 4145 HD12 LEU 251 32.868 35.298 8.107 1.00 0.00 H

ATOM 4146 HD13 LEU 251 31.928 36.705 8.766 1.00 0.00 H

ATOM 4147 CD2 LEU 251 34.961 35.527 9.394 1.00 0.00 C

ATOM 4148 HD21 LEU 251 35.588 35.746 10.259 1.00 0.00 H

ATOM 4149 HD22 LEU 251 34.563 34.515 9.330 1.00 0.00 H

ATOM 4150 HD23 LEU 251 35.622 35.610 8.531 1.00 0.00 H

ATOM 4151 C LEU 251 33.986 39.643 8.024 1.00 0.00 C

ATOM 4152 O LEU 251 33.930 39.562 6.804 1.00 0.00 O

ATOM 4153 N ASP 252 33.052 40.335 8.649 1.00 0.00 N

ATOM 4154 H ASP 252 33.151 40.445 9.649 1.00 0.00 H

ATOM 4155 CA ASP 252 31.768 40.536 8.075 1.00 0.00 C

ATOM 4156 HA ASP 252 31.401 39.735 7.433 1.00 0.00 H

ATOM 4157 CB ASP 252 30.842 40.572 9.203 1.00 0.00 C

ATOM 4158 HB2 ASP 252 31.025 39.721 9.859 1.00 0.00 H

ATOM 4159 HB3 ASP 252 30.882 41.560 9.663 1.00 0.00 H

ATOM 4160 CG ASP 252 29.445 40.323 8.691 1.00 0.00 C

ATOM 4161 OD1 ASP 252 28.661 41.342 8.627 1.00 0.00 O

ATOM 4162 OD2 ASP 252 28.993 39.181 8.564 1.00 0.00 O

ATOM 4163 C ASP 252 31.737 41.754 7.154 1.00 0.00 C

ATOM 4164 O ASP 252 31.011 41.801 6.133 1.00 0.00 O

ATOM 4165 N PHE 253 32.595 42.772 7.458 1.00 0.00 N

ATOM 4166 H PHE 253 33.287 42.647 8.183 1.00 0.00 H

ATOM 4167 CA PHE 253 32.515 44.092 6.853 1.00 0.00 C

ATOM 4168 HA PHE 253 31.468 44.349 6.689 1.00 0.00 H

ATOM 4169 CB PHE 253 32.894 45.133 7.915 1.00 0.00 C

ATOM 4170 HB2 PHE 253 33.892 44.836 8.239 1.00 0.00 H

ATOM 4171 HB3 PHE 253 33.129 46.087 7.443 1.00 0.00 H

ATOM 4172 CG PHE 253 31.955 45.534 9.112 1.00 0.00 C

ATOM 4173 CD1 PHE 253 30.883 44.739 9.497 1.00 0.00 C

ATOM 4174 HD1 PHE 253 30.586 43.902 8.882 1.00 0.00 H

ATOM 4175 CE1 PHE 253 30.084 45.168 10.616 1.00 0.00 C

ATOM 4176 HE1 PHE 253 29.121 44.711 10.791 1.00 0.00 H

ATOM 4177 CZ PHE 253 30.325 46.341 11.232 1.00 0.00 C

ATOM 4178 HZ PHE 253 29.528 46.693 11.870 1.00 0.00 H

ATOM 4179 CE2 PHE 253 31.462 47.149 10.846 1.00 0.00 C

ATOM 4180 HE2 PHE 253 31.700 48.124 11.244 1.00 0.00 H

ATOM 4181 CD2 PHE 253 32.227 46.749 9.759 1.00 0.00 C

ATOM 4182 HD2 PHE 253 32.957 47.462 9.404 1.00 0.00 H

ATOM 4183 C PHE 253 33.314 44.346 5.570 1.00 0.00 C

ATOM 4184 O PHE 253 34.489 44.565 5.568 1.00 0.00 O

ATOM 4185 N GLN 254 32.711 44.207 4.405 1.00 0.00 N

ATOM 4186 H GLN 254 31.737 43.940 4.406 1.00 0.00 H

ATOM 4187 CA GLN 254 33.440 44.002 3.086 1.00 0.00 C

ATOM 4188 HA GLN 254 34.465 44.365 3.162 1.00 0.00 H

ATOM 4189 CB GLN 254 33.340 42.531 2.720 1.00 0.00 C

ATOM 4190 HB2 GLN 254 32.288 42.396 2.466 1.00 0.00 H

ATOM 4191 HB3 GLN 254 34.059 42.357 1.919 1.00 0.00 H

ATOM 4192 CG GLN 254 33.876 41.556 3.734 1.00 0.00 C

ATOM 4193 HG2 GLN 254 33.435 41.825 4.694 1.00 0.00 H

ATOM 4194 HG3 GLN 254 33.580 40.562 3.398 1.00 0.00 H

ATOM 4195 CD GLN 254 35.392 41.565 3.878 1.00 0.00 C

ATOM 4196 OE1 GLN 254 36.074 41.218 2.921 1.00 0.00 O

ATOM 4197 NE2 GLN 254 36.000 41.607 5.053 1.00 0.00 N

ATOM 4198 HE21 GLN 254 36.992 41.435 5.129 1.00 0.00 H

ATOM 4199 HE22 GLN 254 35.427 41.669 5.883 1.00 0.00 H

ATOM 4200 C GLN 254 32.861 44.811 1.952 1.00 0.00 C

ATOM 4201 O GLN 254 33.604 45.184 1.033 1.00 0.00 O

ATOM 4202 N THR 255 31.561 45.235 1.999 1.00 0.00 N

ATOM 4203 H THR 255 31.008 45.063 2.827 1.00 0.00 H

ATOM 4204 CA THR 255 30.961 46.270 0.987 1.00 0.00 C

ATOM 4205 HA THR 255 31.022 45.897 -0.035 1.00 0.00 H

ATOM 4206 CB THR 255 29.498 46.397 1.190 1.00 0.00 C

ATOM 4207 HB THR 255 29.093 47.211 0.587 1.00 0.00 H

ATOM 4208 CG2 THR 255 28.724 45.109 0.901 1.00 0.00 C

ATOM 4209 HG21 THR 255 28.187 44.810 1.801 1.00 0.00 H

ATOM 4210 HG22 THR 255 27.936 45.214 0.156 1.00 0.00 H

ATOM 4211 HG23 THR 255 29.445 44.325 0.670 1.00 0.00 H

ATOM 4212 OG1 THR 255 29.256 46.830 2.508 1.00 0.00 O

ATOM 4213 HG1 THR 255 28.332 46.701 2.735 1.00 0.00 H

ATOM 4214 C THR 255 31.693 47.623 0.911 1.00 0.00 C

ATOM 4215 O THR 255 32.373 47.906 1.921 1.00 0.00 O

ATOM 4216 N PRO 256 31.715 48.449 -0.177 1.00 0.00 N

ATOM 4217 CD PRO 256 31.203 47.974 -1.433 1.00 0.00 C

ATOM 4218 HD2 PRO 256 30.118 47.876 -1.419 1.00 0.00 H

ATOM 4219 HD3 PRO 256 31.674 47.027 -1.694 1.00 0.00 H

ATOM 4220 CG PRO 256 31.478 49.029 -2.479 1.00 0.00 C

ATOM 4221 HG2 PRO 256 30.558 49.246 -3.022 1.00 0.00 H

ATOM 4222 HG3 PRO 256 32.215 48.752 -3.233 1.00 0.00 H

ATOM 4223 CB PRO 256 32.142 50.224 -1.803 1.00 0.00 C

ATOM 4224 HB2 PRO 256 31.353 50.975 -1.761 1.00 0.00 H

ATOM 4225 HB3 PRO 256 33.001 50.652 -2.320 1.00 0.00 H

ATOM 4226 CA PRO 256 32.525 49.671 -0.407 1.00 0.00 C

ATOM 4227 HA PRO 256 33.537 49.284 -0.288 1.00 0.00 H

ATOM 4228 C PRO 256 32.265 50.681 0.771 1.00 0.00 C

ATOM 4229 O PRO 256 33.261 51.326 1.246 1.00 0.00 O

ATOM 4230 N ALA 257 31.017 50.748 1.364 1.00 0.00 N

ATOM 4231 H ALA 257 30.175 50.418 0.913 1.00 0.00 H

ATOM 4232 CA ALA 257 30.737 51.439 2.630 1.00 0.00 C

ATOM 4233 HA ALA 257 31.057 52.479 2.555 1.00 0.00 H

ATOM 4234 CB ALA 257 29.163 51.475 2.822 1.00 0.00 C

ATOM 4235 HB1 ALA 257 28.538 50.641 2.501 1.00 0.00 H

ATOM 4236 HB2 ALA 257 28.934 51.702 3.863 1.00 0.00 H

ATOM 4237 HB3 ALA 257 28.768 52.232 2.144 1.00 0.00 H

ATOM 4238 C ALA 257 31.445 50.736 3.823 1.00 0.00 C

ATOM 4239 O ALA 257 32.153 51.347 4.560 1.00 0.00 O

ATOM 4240 N MET 258 31.063 49.458 4.085 1.00 0.00 N

ATOM 4241 H MET 258 30.484 48.990 3.403 1.00 0.00 H

ATOM 4242 CA MET 258 31.336 48.838 5.397 1.00 0.00 C

ATOM 4243 HA MET 258 31.060 49.540 6.184 1.00 0.00 H

ATOM 4244 CB MET 258 30.535 47.530 5.460 1.00 0.00 C

ATOM 4245 HB2 MET 258 30.835 46.823 4.687 1.00 0.00 H

ATOM 4246 HB3 MET 258 30.946 47.107 6.377 1.00 0.00 H

ATOM 4247 CG MET 258 29.035 47.740 5.564 1.00 0.00 C

ATOM 4248 HG2 MET 258 28.628 48.229 4.679 1.00 0.00 H

ATOM 4249 HG3 MET 258 28.566 46.760 5.653 1.00 0.00 H

ATOM 4250 SD MET 258 28.548 48.621 7.035 1.00 0.00 S

ATOM 4251 CE MET 258 28.563 47.289 8.327 1.00 0.00 C

ATOM 4252 HE1 MET 258 27.972 46.417 8.047 1.00 0.00 H

ATOM 4253 HE2 MET 258 29.627 47.075 8.426 1.00 0.00 H

ATOM 4254 HE3 MET 258 28.303 47.578 9.345 1.00 0.00 H

ATOM 4255 C MET 258 32.875 48.636 5.540 1.00 0.00 C

ATOM 4256 O MET 258 33.391 48.904 6.625 1.00 0.00 O

ATOM 4257 N CYX 259 33.657 48.623 4.505 1.00 0.00 N

ATOM 4258 H CYX 259 33.279 48.250 3.646 1.00 0.00 H

ATOM 4259 CA CYX 259 35.140 48.657 4.635 1.00 0.00 C

ATOM 4260 HA CYX 259 35.437 47.862 5.320 1.00 0.00 H

ATOM 4261 CB CYX 259 35.692 48.214 3.235 1.00 0.00 C

ATOM 4262 HB2 CYX 259 35.092 47.386 2.858 1.00 0.00 H

ATOM 4263 HB3 CYX 259 35.367 49.034 2.595 1.00 0.00 H

ATOM 4264 SG CYX 259 37.457 47.869 3.169 1.00 0.00 S

ATOM 4265 C CYX 259 35.625 49.994 5.178 1.00 0.00 C

ATOM 4266 O CYX 259 36.623 50.014 5.938 1.00 0.00 O

ATOM 4267 N ALA 260 35.076 51.124 4.858 1.00 0.00 N

ATOM 4268 H ALA 260 34.181 51.031 4.399 1.00 0.00 H

ATOM 4269 CA ALA 260 35.419 52.473 5.384 1.00 0.00 C

ATOM 4270 HA ALA 260 36.499 52.510 5.528 1.00 0.00 H

ATOM 4271 CB ALA 260 35.138 53.607 4.381 1.00 0.00 C

ATOM 4272 HB1 ALA 260 35.386 54.508 4.941 1.00 0.00 H

ATOM 4273 HB2 ALA 260 35.845 53.623 3.551 1.00 0.00 H

ATOM 4274 HB3 ALA 260 34.126 53.621 3.977 1.00 0.00 H

ATOM 4275 C ALA 260 34.756 52.727 6.837 1.00 0.00 C

ATOM 4276 O ALA 260 35.184 53.652 7.539 1.00 0.00 O

ATOM 4277 N PHE 261 33.934 51.810 7.358 1.00 0.00 N

ATOM 4278 H PHE 261 33.603 51.115 6.704 1.00 0.00 H

ATOM 4279 CA PHE 261 33.705 51.565 8.781 1.00 0.00 C

ATOM 4280 HA PHE 261 33.767 52.508 9.324 1.00 0.00 H

ATOM 4281 CB PHE 261 32.187 51.138 8.965 1.00 0.00 C

ATOM 4282 HB2 PHE 261 31.770 52.022 8.483 1.00 0.00 H

ATOM 4283 HB3 PHE 261 31.891 50.340 8.285 1.00 0.00 H

ATOM 4284 CG PHE 261 31.426 50.855 10.206 1.00 0.00 C

ATOM 4285 CD1 PHE 261 32.067 50.893 11.494 1.00 0.00 C

ATOM 4286 HD1 PHE 261 32.886 51.547 11.751 1.00 0.00 H

ATOM 4287 CE1 PHE 261 31.347 50.394 12.626 1.00 0.00 C

ATOM 4288 HE1 PHE 261 31.780 50.721 13.560 1.00 0.00 H

ATOM 4289 CZ PHE 261 30.024 49.928 12.541 1.00 0.00 C

ATOM 4290 HZ PHE 261 29.403 49.993 13.422 1.00 0.00 H

ATOM 4291 CE2 PHE 261 29.369 49.855 11.302 1.00 0.00 C

ATOM 4292 HE2 PHE 261 28.323 49.591 11.252 1.00 0.00 H

ATOM 4293 CD2 PHE 261 30.091 50.246 10.141 1.00 0.00 C

ATOM 4294 HD2 PHE 261 29.599 50.216 9.180 1.00 0.00 H

ATOM 4295 C PHE 261 34.714 50.630 9.450 1.00 0.00 C

ATOM 4296 O PHE 261 35.250 50.966 10.496 1.00 0.00 O

ATOM 4297 N ASN 262 35.094 49.479 8.839 1.00 0.00 N

ATOM 4298 H ASN 262 34.578 49.359 7.979 1.00 0.00 H

ATOM 4299 CA ASN 262 36.053 48.455 9.290 1.00 0.00 C

ATOM 4300 HA ASN 262 35.697 48.036 10.231 1.00 0.00 H

ATOM 4301 CB ASN 262 35.967 47.396 8.213 1.00 0.00 C

ATOM 4302 HB2 ASN 262 34.919 47.101 8.270 1.00 0.00 H

ATOM 4303 HB3 ASN 262 36.155 47.807 7.221 1.00 0.00 H

ATOM 4304 CG ASN 262 36.714 46.160 8.588 1.00 0.00 C

ATOM 4305 OD1 ASN 262 37.226 45.895 9.699 1.00 0.00 O

ATOM 4306 ND2 ASN 262 36.537 45.227 7.674 1.00 0.00 N

ATOM 4307 HD21 ASN 262 36.134 45.485 6.785 1.00 0.00 H

ATOM 4308 HD22 ASN 262 36.501 44.275 8.009 1.00 0.00 H

ATOM 4309 C ASN 262 37.506 49.083 9.421 1.00 0.00 C

ATOM 4310 O ASN 262 38.287 48.832 10.341 1.00 0.00 O

ATOM 4311 N ASP 263 37.890 49.944 8.506 1.00 0.00 N

ATOM 4312 H ASP 263 37.196 50.068 7.782 1.00 0.00 H

ATOM 4313 CA ASP 263 39.037 50.818 8.690 1.00 0.00 C

ATOM 4314 HA ASP 263 39.910 50.173 8.796 1.00 0.00 H

ATOM 4315 CB ASP 263 39.230 51.578 7.379 1.00 0.00 C

ATOM 4316 HB2 ASP 263 39.125 50.813 6.608 1.00 0.00 H

ATOM 4317 HB3 ASP 263 38.519 52.401 7.307 1.00 0.00 H

ATOM 4318 CG ASP 263 40.598 52.202 7.463 1.00 0.00 C

ATOM 4319 OD1 ASP 263 40.651 53.433 7.522 1.00 0.00 O

ATOM 4320 OD2 ASP 263 41.599 51.546 7.159 1.00 0.00 O

ATOM 4321 C ASP 263 39.136 51.849 9.842 1.00 0.00 C

ATOM 4322 O ASP 263 40.167 51.822 10.540 1.00 0.00 O

ATOM 4323 N ARG 264 38.089 52.688 10.084 1.00 0.00 N

ATOM 4324 H ARG 264 37.303 52.465 9.491 1.00 0.00 H

ATOM 4325 CA ARG 264 37.806 53.354 11.377 1.00 0.00 C

ATOM 4326 HA ARG 264 38.661 54.003 11.566 1.00 0.00 H

ATOM 4327 CB ARG 264 36.500 54.173 11.346 1.00 0.00 C

ATOM 4328 HB2 ARG 264 35.784 53.653 10.710 1.00 0.00 H

ATOM 4329 HB3 ARG 264 36.114 54.157 12.365 1.00 0.00 H

ATOM 4330 CG ARG 264 36.524 55.663 10.849 1.00 0.00 C

ATOM 4331 HG2 ARG 264 35.530 56.096 10.742 1.00 0.00 H

ATOM 4332 HG3 ARG 264 37.043 56.292 11.573 1.00 0.00 H

ATOM 4333 CD ARG 264 37.289 55.777 9.582 1.00 0.00 C

ATOM 4334 HD2 ARG 264 38.367 55.736 9.734 1.00 0.00 H

ATOM 4335 HD3 ARG 264 36.985 54.955 8.934 1.00 0.00 H

ATOM 4336 NE ARG 264 36.976 57.079 8.921 1.00 0.00 N

ATOM 4337 HE ARG 264 37.725 57.747 8.805 1.00 0.00 H

ATOM 4338 CZ ARG 264 35.928 57.277 8.110 1.00 0.00 C

ATOM 4339 NH1 ARG 264 35.623 58.527 7.986 1.00 0.00 N

ATOM 4340 HH11 ARG 264 34.752 58.834 7.577 1.00 0.00 H

ATOM 4341 HH12 ARG 264 36.336 59.204 8.216 1.00 0.00 H

ATOM 4342 NH2 ARG 264 35.074 56.454 7.659 1.00 0.00 N

ATOM 4343 HH21 ARG 264 34.318 56.733 7.049 1.00 0.00 H

ATOM 4344 HH22 ARG 264 35.205 55.468 7.834 1.00 0.00 H

ATOM 4345 C ARG 264 37.851 52.445 12.621 1.00 0.00 C

ATOM 4346 O ARG 264 38.286 52.958 13.612 1.00 0.00 O

ATOM 4347 N VAL 265 37.247 51.272 12.627 1.00 0.00 N

ATOM 4348 H VAL 265 36.722 50.959 11.824 1.00 0.00 H

ATOM 4349 CA VAL 265 37.408 50.220 13.673 1.00 0.00 C

ATOM 4350 HA VAL 265 37.093 50.684 14.608 1.00 0.00 H

ATOM 4351 CB VAL 265 36.518 49.079 13.325 1.00 0.00 C

ATOM 4352 HB VAL 265 36.945 48.606 12.440 1.00 0.00 H

ATOM 4353 CG1 VAL 265 36.572 48.058 14.467 1.00 0.00 C

ATOM 4354 HG11 VAL 265 36.074 47.132 14.178 1.00 0.00 H

ATOM 4355 HG12 VAL 265 37.593 47.731 14.664 1.00 0.00 H

ATOM 4356 HG13 VAL 265 36.149 48.295 15.443 1.00 0.00 H

ATOM 4357 CG2 VAL 265 35.043 49.420 13.208 1.00 0.00 C

ATOM 4358 HG21 VAL 265 34.888 50.489 13.057 1.00 0.00 H

ATOM 4359 HG22 VAL 265 34.791 48.934 12.265 1.00 0.00 H

ATOM 4360 HG23 VAL 265 34.519 49.181 14.133 1.00 0.00 H

ATOM 4361 C VAL 265 38.952 49.810 13.915 1.00 0.00 C

ATOM 4362 O VAL 265 39.450 50.256 14.912 1.00 0.00 O

ATOM 4363 N TYR 266 39.714 49.583 12.818 1.00 0.00 N

ATOM 4364 H TYR 266 39.158 49.227 12.053 1.00 0.00 H

ATOM 4365 CA TYR 266 41.151 49.290 12.899 1.00 0.00 C

ATOM 4366 HA TYR 266 41.428 48.496 13.592 1.00 0.00 H

ATOM 4367 CB TYR 266 41.636 48.907 11.564 1.00 0.00 C

ATOM 4368 HB2 TYR 266 40.997 48.149 11.111 1.00 0.00 H

ATOM 4369 HB3 TYR 266 41.539 49.775 10.912 1.00 0.00 H

ATOM 4370 CG TYR 266 43.139 48.504 11.393 1.00 0.00 C

ATOM 4371 CD1 TYR 266 43.888 48.841 10.251 1.00 0.00 C

ATOM 4372 HD1 TYR 266 43.510 49.494 9.478 1.00 0.00 H

ATOM 4373 CE1 TYR 266 45.129 48.238 10.051 1.00 0.00 C

ATOM 4374 HE1 TYR 266 45.574 48.272 9.068 1.00 0.00 H

ATOM 4375 CZ TYR 266 45.668 47.498 11.116 1.00 0.00 C

ATOM 4376 OH TYR 266 46.913 47.041 10.965 1.00 0.00 O

ATOM 4377 HH TYR 266 47.307 46.656 11.752 1.00 0.00 H

ATOM 4378 CE2 TYR 266 44.952 47.205 12.265 1.00 0.00 C

ATOM 4379 HE2 TYR 266 45.313 46.661 13.125 1.00 0.00 H

ATOM 4380 CD2 TYR 266 43.678 47.710 12.371 1.00 0.00 C

ATOM 4381 HD2 TYR 266 43.111 47.518 13.270 1.00 0.00 H

ATOM 4382 C TYR 266 41.972 50.569 13.296 1.00 0.00 C

ATOM 4383 O TYR 266 42.836 50.459 14.158 1.00 0.00 O

ATOM 4384 N ALA 267 41.696 51.782 12.761 1.00 0.00 N

ATOM 4385 H ALA 267 40.889 51.840 12.156 1.00 0.00 H

ATOM 4386 CA ALA 267 42.126 53.034 13.338 1.00 0.00 C

ATOM 4387 HA ALA 267 43.200 53.073 13.157 1.00 0.00 H

ATOM 4388 CB ALA 267 41.673 54.279 12.472 1.00 0.00 C

ATOM 4389 HB1 ALA 267 41.050 54.990 13.013 1.00 0.00 H

ATOM 4390 HB2 ALA 267 42.505 54.777 11.973 1.00 0.00 H

ATOM 4391 HB3 ALA 267 41.097 53.902 11.627 1.00 0.00 H

ATOM 4392 C ALA 267 41.909 53.282 14.863 1.00 0.00 C

ATOM 4393 O ALA 267 42.869 53.504 15.559 1.00 0.00 O

ATOM 4394 N THR 268 40.662 53.028 15.319 1.00 0.00 N

ATOM 4395 H THR 268 39.874 52.989 14.688 1.00 0.00 H

ATOM 4396 CA THR 268 40.388 53.076 16.704 1.00 0.00 C

ATOM 4397 HA THR 268 40.746 53.976 17.204 1.00 0.00 H

ATOM 4398 CB THR 268 38.830 53.114 16.817 1.00 0.00 C

ATOM 4399 HB THR 268 38.452 52.144 16.494 1.00 0.00 H

ATOM 4400 CG2 THR 268 38.362 53.461 18.255 1.00 0.00 C

ATOM 4401 HG21 THR 268 38.892 52.812 18.953 1.00 0.00 H

ATOM 4402 HG22 THR 268 38.435 54.500 18.578 1.00 0.00 H

ATOM 4403 HG23 THR 268 37.309 53.186 18.206 1.00 0.00 H

ATOM 4404 OG1 THR 268 38.264 54.146 15.998 1.00 0.00 O

ATOM 4405 HG1 THR 268 38.228 53.777 15.113 1.00 0.00 H

ATOM 4406 C THR 268 41.066 51.921 17.515 1.00 0.00 C

ATOM 4407 O THR 268 41.495 52.161 18.620 1.00 0.00 O

ATOM 4408 N TYR 269 41.027 50.687 17.012 1.00 0.00 N

ATOM 4409 H TYR 269 40.589 50.546 16.113 1.00 0.00 H

ATOM 4410 CA TYR 269 41.834 49.536 17.487 1.00 0.00 C

ATOM 4411 HA TYR 269 41.505 49.377 18.514 1.00 0.00 H

ATOM 4412 CB TYR 269 41.643 48.272 16.638 1.00 0.00 C

ATOM 4413 HB2 TYR 269 40.581 48.037 16.562 1.00 0.00 H

ATOM 4414 HB3 TYR 269 41.992 48.504 15.631 1.00 0.00 H

ATOM 4415 CG TYR 269 42.192 46.956 17.180 1.00 0.00 C

ATOM 4416 CD1 TYR 269 42.038 46.615 18.553 1.00 0.00 C

ATOM 4417 HD1 TYR 269 41.544 47.333 19.191 1.00 0.00 H

ATOM 4418 CE1 TYR 269 42.580 45.517 19.153 1.00 0.00 C

ATOM 4419 HE1 TYR 269 42.526 45.368 20.221 1.00 0.00 H

ATOM 4420 CZ TYR 269 43.148 44.545 18.360 1.00 0.00 C

ATOM 4421 OH TYR 269 43.701 43.471 18.960 1.00 0.00 O

ATOM 4422 HH TYR 269 44.269 43.041 18.317 1.00 0.00 H

ATOM 4423 CE2 TYR 269 43.241 44.744 16.962 1.00 0.00 C

ATOM 4424 HE2 TYR 269 43.748 44.024 16.338 1.00 0.00 H

ATOM 4425 CD2 TYR 269 42.750 45.980 16.372 1.00 0.00 C

ATOM 4426 HD2 TYR 269 43.063 46.289 15.386 1.00 0.00 H

ATOM 4427 C TYR 269 43.332 50.014 17.581 1.00 0.00 C

ATOM 4428 O TYR 269 43.975 49.841 18.628 1.00 0.00 O

ATOM 4429 N GLN 270 43.917 50.688 16.584 1.00 0.00 N

ATOM 4430 H GLN 270 43.320 50.975 15.822 1.00 0.00 H

ATOM 4431 CA GLN 270 45.343 51.069 16.700 1.00 0.00 C

ATOM 4432 HA GLN 270 45.806 50.206 17.178 1.00 0.00 H

ATOM 4433 CB GLN 270 46.009 51.282 15.282 1.00 0.00 C

ATOM 4434 HB2 GLN 270 45.337 51.899 14.684 1.00 0.00 H

ATOM 4435 HB3 GLN 270 46.948 51.824 15.395 1.00 0.00 H

ATOM 4436 CG GLN 270 46.054 50.010 14.491 1.00 0.00 C

ATOM 4437 HG2 GLN 270 45.063 49.557 14.473 1.00 0.00 H

ATOM 4438 HG3 GLN 270 46.215 50.267 13.443 1.00 0.00 H

ATOM 4439 CD GLN 270 46.927 48.958 15.028 1.00 0.00 C

ATOM 4440 OE1 GLN 270 46.549 48.063 15.780 1.00 0.00 O

ATOM 4441 NE2 GLN 270 48.207 49.063 14.748 1.00 0.00 N

ATOM 4442 HE21 GLN 270 48.832 48.390 15.168 1.00 0.00 H

ATOM 4443 HE22 GLN 270 48.586 49.811 14.185 1.00 0.00 H

ATOM 4444 C GLN 270 45.617 52.274 17.645 1.00 0.00 C

ATOM 4445 O GLN 270 46.657 52.217 18.348 1.00 0.00 O

ATOM 4446 N VAL 271 44.718 53.264 17.694 1.00 0.00 N

ATOM 4447 H VAL 271 43.986 53.273 16.998 1.00 0.00 H

ATOM 4448 CA VAL 271 44.755 54.288 18.728 1.00 0.00 C

ATOM 4449 HA VAL 271 45.643 54.908 18.607 1.00 0.00 H

ATOM 4450 CB VAL 271 43.545 55.221 18.592 1.00 0.00 C

ATOM 4451 HB VAL 271 42.646 54.660 18.337 1.00 0.00 H

ATOM 4452 CG1 VAL 271 43.302 56.207 19.740 1.00 0.00 C

ATOM 4453 HG11 VAL 271 42.904 55.773 20.657 1.00 0.00 H

ATOM 4454 HG12 VAL 271 44.260 56.666 19.982 1.00 0.00 H

ATOM 4455 HG13 VAL 271 42.557 56.945 19.441 1.00 0.00 H

ATOM 4456 CG2 VAL 271 43.838 56.281 17.471 1.00 0.00 C

ATOM 4457 HG21 VAL 271 44.546 57.020 17.846 1.00 0.00 H

ATOM 4458 HG22 VAL 271 44.187 55.742 16.589 1.00 0.00 H

ATOM 4459 HG23 VAL 271 42.972 56.862 17.154 1.00 0.00 H

ATOM 4460 C VAL 271 44.671 53.588 20.145 1.00 0.00 C

ATOM 4461 O VAL 271 45.505 53.859 21.018 1.00 0.00 O

ATOM 4462 N THR 272 43.906 52.558 20.276 1.00 0.00 N

ATOM 4463 H THR 272 43.205 52.337 19.583 1.00 0.00 H

ATOM 4464 CA THR 272 43.773 51.835 21.545 1.00 0.00 C

ATOM 4465 HA THR 272 43.770 52.629 22.291 1.00 0.00 H

ATOM 4466 CB THR 272 42.383 51.265 21.789 1.00 0.00 C

ATOM 4467 HB THR 272 42.370 50.642 22.683 1.00 0.00 H

ATOM 4468 CG2 THR 272 41.158 52.287 22.007 1.00 0.00 C

ATOM 4469 HG21 THR 272 41.213 53.086 21.267 1.00 0.00 H

ATOM 4470 HG22 THR 272 40.178 51.810 21.966 1.00 0.00 H

ATOM 4471 HG23 THR 272 41.218 52.624 23.042 1.00 0.00 H

ATOM 4472 OG1 THR 272 41.911 50.599 20.705 1.00 0.00 O

ATOM 4473 HG1 THR 272 41.651 51.174 19.982 1.00 0.00 H

ATOM 4474 C THR 272 45.014 50.881 21.883 1.00 0.00 C

ATOM 4475 O THR 272 45.381 50.811 23.067 1.00 0.00 O

ATOM 4476 N ARG 273 45.680 50.347 20.868 1.00 0.00 N

ATOM 4477 H ARG 273 45.141 50.281 20.016 1.00 0.00 H

ATOM 4478 CA ARG 273 46.975 49.693 21.062 1.00 0.00 C

ATOM 4479 HA ARG 273 46.964 49.101 21.977 1.00 0.00 H

ATOM 4480 CB ARG 273 47.119 48.741 19.891 1.00 0.00 C

ATOM 4481 HB2 ARG 273 46.164 48.292 19.619 1.00 0.00 H

ATOM 4482 HB3 ARG 273 47.392 49.385 19.055 1.00 0.00 H

ATOM 4483 CG ARG 273 48.126 47.565 20.219 1.00 0.00 C

ATOM 4484 HG2 ARG 273 49.040 47.967 20.656 1.00 0.00 H

ATOM 4485 HG3 ARG 273 47.670 46.789 20.835 1.00 0.00 H

ATOM 4486 CD ARG 273 48.607 46.793 18.972 1.00 0.00 C

ATOM 4487 HD2 ARG 273 48.920 47.603 18.312 1.00 0.00 H

ATOM 4488 HD3 ARG 273 49.403 46.117 19.282 1.00 0.00 H

ATOM 4489 NE ARG 273 47.481 46.243 18.251 1.00 0.00 N

ATOM 4490 HE ARG 273 46.688 46.860 18.156 1.00 0.00 H

ATOM 4491 CZ ARG 273 47.460 45.204 17.423 1.00 0.00 C

ATOM 4492 NH1 ARG 273 46.888 45.249 16.280 1.00 0.00 N

ATOM 4493 HH11 ARG 273 46.746 44.383 15.780 1.00 0.00 H

ATOM 4494 HH12 ARG 273 46.844 46.118 15.767 1.00 0.00 H

ATOM 4495 NH2 ARG 273 48.248 44.189 17.664 1.00 0.00 N

ATOM 4496 HH21 ARG 273 48.479 43.412 17.062 1.00 0.00 H

ATOM 4497 HH22 ARG 273 48.663 44.196 18.585 1.00 0.00 H

ATOM 4498 C ARG 273 48.084 50.689 21.267 1.00 0.00 C

ATOM 4499 O ARG 273 49.135 50.361 21.799 1.00 0.00 O

ATOM 4500 N GLY 274 47.866 51.924 20.854 1.00 0.00 N

ATOM 4501 H GLY 274 47.130 52.057 20.175 1.00 0.00 H

ATOM 4502 CA GLY 274 48.610 53.078 21.357 1.00 0.00 C

ATOM 4503 HA2 GLY 274 49.646 52.790 21.179 1.00 0.00 H

ATOM 4504 HA3 GLY 274 48.442 53.985 20.776 1.00 0.00 H

ATOM 4505 C GLY 274 48.370 53.525 22.864 1.00 0.00 C

ATOM 4506 O GLY 274 49.352 53.533 23.585 1.00 0.00 O

ATOM 4507 N LEU 275 47.116 53.685 23.252 1.00 0.00 N

ATOM 4508 H LEU 275 46.452 53.315 22.587 1.00 0.00 H

ATOM 4509 CA LEU 275 46.624 53.931 24.563 1.00 0.00 C

ATOM 4510 HA LEU 275 47.154 54.816 24.916 1.00 0.00 H

ATOM 4511 CB LEU 275 45.168 54.340 24.419 1.00 0.00 C

ATOM 4512 HB2 LEU 275 45.087 55.021 23.572 1.00 0.00 H

ATOM 4513 HB3 LEU 275 44.685 53.401 24.150 1.00 0.00 H

ATOM 4514 CG LEU 275 44.528 54.948 25.649 1.00 0.00 C

ATOM 4515 HG LEU 275 44.833 54.303 26.472 1.00 0.00 H

ATOM 4516 CD1 LEU 275 45.154 56.321 25.843 1.00 0.00 C

ATOM 4517 HD11 LEU 275 46.234 56.265 25.985 1.00 0.00 H

ATOM 4518 HD12 LEU 275 44.975 57.068 25.071 1.00 0.00 H

ATOM 4519 HD13 LEU 275 44.785 56.630 26.821 1.00 0.00 H

ATOM 4520 CD2 LEU 275 43.049 55.224 25.558 1.00 0.00 C

ATOM 4521 HD21 LEU 275 42.538 55.459 26.492 1.00 0.00 H

ATOM 4522 HD22 LEU 275 42.793 56.093 24.952 1.00 0.00 H

ATOM 4523 HD23 LEU 275 42.508 54.333 25.240 1.00 0.00 H

ATOM 4524 C LEU 275 46.931 52.865 25.678 1.00 0.00 C

ATOM 4525 O LEU 275 46.991 53.195 26.844 1.00 0.00 O

ATOM 4526 N ALA 276 47.181 51.571 25.299 1.00 0.00 N

ATOM 4527 H ALA 276 47.035 51.269 24.346 1.00 0.00 H

ATOM 4528 CA ALA 276 47.583 50.467 26.262 1.00 0.00 C

ATOM 4529 HA ALA 276 46.810 50.350 27.022 1.00 0.00 H

ATOM 4530 CB ALA 276 47.475 49.182 25.429 1.00 0.00 C

ATOM 4531 HB1 ALA 276 47.765 48.330 26.043 1.00 0.00 H

ATOM 4532 HB2 ALA 276 46.432 49.111 25.119 1.00 0.00 H

ATOM 4533 HB3 ALA 276 48.183 49.245 24.603 1.00 0.00 H

ATOM 4534 C ALA 276 48.890 50.745 26.983 1.00 0.00 C

ATOM 4535 O ALA 276 49.076 50.248 28.123 1.00 0.00 O

ATOM 4536 N SER 277 49.831 51.519 26.352 1.00 0.00 N

ATOM 4537 H SER 277 49.561 51.765 25.410 1.00 0.00 H

ATOM 4538 CA SER 277 51.153 51.925 26.907 1.00 0.00 C

ATOM 4539 HA SER 277 51.843 51.158 27.259 1.00 0.00 H

ATOM 4540 CB SER 277 52.024 52.665 25.774 1.00 0.00 C

ATOM 4541 HB2 SER 277 52.019 53.741 25.949 1.00 0.00 H

ATOM 4542 HB3 SER 277 52.991 52.266 26.080 1.00 0.00 H

ATOM 4543 OG SER 277 51.707 52.418 24.447 1.00 0.00 O

ATOM 4544 HG SER 277 51.068 53.050 24.111 1.00 0.00 H

ATOM 4545 C SER 277 51.098 52.933 28.065 1.00 0.00 C

ATOM 4546 O SER 277 52.128 53.188 28.765 1.00 0.00 O

ATOM 4547 N LEU 278 49.916 53.593 28.317 1.00 0.00 N

ATOM 4548 H LEU 278 49.166 53.359 27.682 1.00 0.00 H

ATOM 4549 CA LEU 278 49.626 54.474 29.487 1.00 0.00 C

ATOM 4550 HA LEU 278 50.199 55.402 29.492 1.00 0.00 H

ATOM 4551 CB LEU 278 48.209 54.943 29.302 1.00 0.00 C

ATOM 4552 HB2 LEU 278 48.151 55.651 28.475 1.00 0.00 H

ATOM 4553 HB3 LEU 278 47.527 54.134 29.042 1.00 0.00 H

ATOM 4554 CG LEU 278 47.470 55.782 30.433 1.00 0.00 C

ATOM 4555 HG LEU 278 47.490 55.206 31.359 1.00 0.00 H

ATOM 4556 CD1 LEU 278 48.010 57.147 30.829 1.00 0.00 C

ATOM 4557 HD11 LEU 278 47.417 57.404 31.707 1.00 0.00 H

ATOM 4558 HD12 LEU 278 49.090 57.048 30.938 1.00 0.00 H

ATOM 4559 HD13 LEU 278 47.848 57.812 29.980 1.00 0.00 H

ATOM 4560 CD2 LEU 278 45.998 56.212 30.034 1.00 0.00 C

ATOM 4561 HD21 LEU 278 45.443 55.332 29.710 1.00 0.00 H

ATOM 4562 HD22 LEU 278 45.413 56.655 30.840 1.00 0.00 H

ATOM 4563 HD23 LEU 278 46.005 56.792 29.111 1.00 0.00 H

ATOM 4564 C LEU 278 49.707 53.841 30.871 1.00 0.00 C

ATOM 4565 O LEU 278 49.879 54.548 31.877 1.00 0.00 O

ATOM 4566 N ASN 279 49.542 52.518 31.077 1.00 0.00 N

ATOM 4567 H ASN 279 49.219 52.016 30.262 1.00 0.00 H

ATOM 4568 CA ASN 279 49.918 51.763 32.219 1.00 0.00 C

ATOM 4569 HA ASN 279 49.551 52.280 33.106 1.00 0.00 H

ATOM 4570 CB ASN 279 49.136 50.452 32.146 1.00 0.00 C

ATOM 4571 HB2 ASN 279 48.072 50.662 32.036 1.00 0.00 H

ATOM 4572 HB3 ASN 279 49.493 49.924 31.263 1.00 0.00 H

ATOM 4573 CG ASN 279 49.344 49.674 33.468 1.00 0.00 C

ATOM 4574 OD1 ASN 279 48.722 50.011 34.428 1.00 0.00 O

ATOM 4575 ND2 ASN 279 50.252 48.729 33.418 1.00 0.00 N

ATOM 4576 HD21 ASN 279 50.550 48.344 32.534 1.00 0.00 H

ATOM 4577 HD22 ASN 279 50.445 48.328 34.325 1.00 0.00 H

ATOM 4578 C ASN 279 51.473 51.544 32.372 1.00 0.00 C

ATOM 4579 O ASN 279 52.154 51.950 33.398 1.00 0.00 O

ATOM 4580 N SER 280 52.027 51.037 31.265 1.00 0.00 N

ATOM 4581 H SER 280 51.416 50.811 30.493 1.00 0.00 H

ATOM 4582 CA SER 280 53.514 50.831 31.169 1.00 0.00 C

ATOM 4583 HA SER 280 53.868 50.343 32.078 1.00 0.00 H

ATOM 4584 CB SER 280 53.834 49.904 29.994 1.00 0.00 C

ATOM 4585 HB2 SER 280 53.757 50.548 29.117 1.00 0.00 H

ATOM 4586 HB3 SER 280 54.879 49.603 30.063 1.00 0.00 H

ATOM 4587 OG SER 280 52.971 48.766 29.911 1.00 0.00 O

ATOM 4588 HG SER 280 53.231 48.297 30.707 1.00 0.00 H

ATOM 4589 C SER 280 54.421 52.036 31.104 1.00 0.00 C

ATOM 4590 O SER 280 55.608 51.865 31.289 1.00 0.00 O

ATOM 4591 N CYX 281 53.909 53.242 31.001 1.00 0.00 N

ATOM 4592 H CYX 281 53.108 53.289 30.388 1.00 0.00 H

ATOM 4593 CA CYX 281 54.585 54.493 31.216 1.00 0.00 C

ATOM 4594 HA CYX 281 55.599 54.570 30.825 1.00 0.00 H

ATOM 4595 CB CYX 281 53.664 55.577 30.506 1.00 0.00 C

ATOM 4596 HB2 CYX 281 53.482 55.219 29.493 1.00 0.00 H

ATOM 4597 HB3 CYX 281 52.668 55.583 30.948 1.00 0.00 H

ATOM 4598 SG CYX 281 54.287 57.303 30.291 1.00 0.00 S

ATOM 4599 C CYX 281 54.737 54.886 32.697 1.00 0.00 C

ATOM 4600 O CYX 281 55.752 55.499 33.098 1.00 0.00 O

ATOM 4601 N VAL 282 53.783 54.374 33.456 1.00 0.00 N

ATOM 4602 H VAL 282 53.183 53.623 33.146 1.00 0.00 H

ATOM 4603 CA VAL 282 53.500 54.902 34.842 1.00 0.00 C

ATOM 4604 HA VAL 282 54.104 55.792 35.013 1.00 0.00 H

ATOM 4605 CB VAL 282 51.992 55.209 35.104 1.00 0.00 C

ATOM 4606 HB VAL 282 51.392 54.367 34.760 1.00 0.00 H

ATOM 4607 CG1 VAL 282 51.647 55.518 36.586 1.00 0.00 C

ATOM 4608 HG11 VAL 282 50.596 55.756 36.748 1.00 0.00 H

ATOM 4609 HG12 VAL 282 51.785 54.671 37.258 1.00 0.00 H

ATOM 4610 HG13 VAL 282 52.253 56.377 36.874 1.00 0.00 H

ATOM 4611 CG2 VAL 282 51.441 56.313 34.151 1.00 0.00 C

ATOM 4612 HG21 VAL 282 50.608 56.845 34.611 1.00 0.00 H

ATOM 4613 HG22 VAL 282 52.287 56.982 33.996 1.00 0.00 H

ATOM 4614 HG23 VAL 282 51.346 55.906 33.145 1.00 0.00 H

ATOM 4615 C VAL 282 53.921 53.846 35.855 1.00 0.00 C

ATOM 4616 O VAL 282 54.512 54.262 36.886 1.00 0.00 O

ATOM 4617 N ASN 283 53.801 52.564 35.538 1.00 0.00 N

ATOM 4618 H ASN 283 53.416 52.311 34.639 1.00 0.00 H

ATOM 4619 CA ASN 283 54.496 51.482 36.235 1.00 0.00 C

ATOM 4620 HA ASN 283 54.237 51.651 37.280 1.00 0.00 H

ATOM 4621 CB ASN 283 53.912 50.175 35.575 1.00 0.00 C

ATOM 4622 HB2 ASN 283 52.827 50.119 35.662 1.00 0.00 H

ATOM 4623 HB3 ASN 283 54.319 50.156 34.564 1.00 0.00 H

ATOM 4624 CG ASN 283 54.336 48.820 36.189 1.00 0.00 C

ATOM 4625 OD1 ASN 283 53.924 48.397 37.245 1.00 0.00 O

ATOM 4626 ND2 ASN 283 55.154 48.111 35.549 1.00 0.00 N

ATOM 4627 HD21 ASN 283 55.352 48.247 34.568 1.00 0.00 H

ATOM 4628 HD22 ASN 283 55.334 47.157 35.828 1.00 0.00 H

ATOM 4629 C ASN 283 55.992 51.476 36.408 1.00 0.00 C

ATOM 4630 O ASN 283 56.513 50.906 37.411 1.00 0.00 O

ATOM 4631 N PRO 284 56.844 52.000 35.488 1.00 0.00 N

ATOM 4632 CD PRO 284 56.593 51.932 34.083 1.00 0.00 C

ATOM 4633 HD2 PRO 284 55.975 52.778 33.785 1.00 0.00 H

ATOM 4634 HD3 PRO 284 56.136 50.964 33.875 1.00 0.00 H

ATOM 4635 CG PRO 284 57.905 52.106 33.421 1.00 0.00 C

ATOM 4636 HG2 PRO 284 57.854 52.679 32.495 1.00 0.00 H

ATOM 4637 HG3 PRO 284 58.215 51.088 33.184 1.00 0.00 H

ATOM 4638 CB PRO 284 58.810 52.786 34.418 1.00 0.00 C

ATOM 4639 HB2 PRO 284 58.671 53.866 34.372 1.00 0.00 H

ATOM 4640 HB3 PRO 284 59.845 52.457 34.334 1.00 0.00 H

ATOM 4641 CA PRO 284 58.245 52.256 35.753 1.00 0.00 C

ATOM 4642 HA PRO 284 58.661 51.339 36.171 1.00 0.00 H

ATOM 4643 C PRO 284 58.494 53.380 36.794 1.00 0.00 C

ATOM 4644 O PRO 284 59.625 53.673 37.310 1.00 0.00 O

ATOM 4645 N ILE 285 57.474 54.225 36.996 1.00 0.00 N

ATOM 4646 H ILE 285 56.559 53.854 36.782 1.00 0.00 H

ATOM 4647 CA ILE 285 57.665 55.516 37.706 1.00 0.00 C

ATOM 4648 HA ILE 285 58.662 55.441 38.140 1.00 0.00 H

ATOM 4649 CB ILE 285 57.725 56.760 36.815 1.00 0.00 C

ATOM 4650 HB ILE 285 58.184 57.487 37.485 1.00 0.00 H

ATOM 4651 CG2 ILE 285 58.588 56.602 35.575 1.00 0.00 C

ATOM 4652 HG21 ILE 285 58.693 57.611 35.177 1.00 0.00 H

ATOM 4653 HG22 ILE 285 59.488 55.993 35.653 1.00 0.00 H

ATOM 4654 HG23 ILE 285 58.008 56.162 34.763 1.00 0.00 H

ATOM 4655 CG1 ILE 285 56.251 57.138 36.538 1.00 0.00 C

ATOM 4656 HG12 ILE 285 55.839 56.322 35.945 1.00 0.00 H

ATOM 4657 HG13 ILE 285 55.657 57.324 37.433 1.00 0.00 H

ATOM 4658 CD1 ILE 285 55.972 58.396 35.633 1.00 0.00 C

ATOM 4659 HD11 ILE 285 56.137 59.294 36.229 1.00 0.00 H

ATOM 4660 HD12 ILE 285 56.655 58.566 34.801 1.00 0.00 H

ATOM 4661 HD13 ILE 285 54.919 58.407 35.349 1.00 0.00 H

ATOM 4662 C ILE 285 56.840 55.604 39.076 1.00 0.00 C

ATOM 4663 O ILE 285 56.882 56.710 39.736 1.00 0.00 O

ATOM 4664 N LEU 286 56.027 54.615 39.326 1.00 0.00 N

ATOM 4665 H LEU 286 55.926 53.951 38.571 1.00 0.00 H

ATOM 4666 CA LEU 286 55.150 54.544 40.506 1.00 0.00 C

ATOM 4667 HA LEU 286 54.349 55.282 40.466 1.00 0.00 H

ATOM 4668 CB LEU 286 54.534 53.100 40.395 1.00 0.00 C

ATOM 4669 HB2 LEU 286 54.090 52.882 39.424 1.00 0.00 H

ATOM 4670 HB3 LEU 286 55.355 52.408 40.586 1.00 0.00 H

ATOM 4671 CG LEU 286 53.406 52.787 41.369 1.00 0.00 C

ATOM 4672 HG LEU 286 53.992 52.767 42.288 1.00 0.00 H

ATOM 4673 CD1 LEU 286 52.409 53.867 41.578 1.00 0.00 C

ATOM 4674 HD11 LEU 286 51.798 53.556 42.426 1.00 0.00 H

ATOM 4675 HD12 LEU 286 52.909 54.804 41.821 1.00 0.00 H

ATOM 4676 HD13 LEU 286 51.809 54.017 40.681 1.00 0.00 H

ATOM 4677 CD2 LEU 286 52.702 51.484 40.990 1.00 0.00 C

ATOM 4678 HD21 LEU 286 51.940 51.131 41.686 1.00 0.00 H

ATOM 4679 HD22 LEU 286 52.149 51.635 40.063 1.00 0.00 H

ATOM 4680 HD23 LEU 286 53.360 50.626 40.852 1.00 0.00 H

ATOM 4681 C LEU 286 55.869 54.654 41.836 1.00 0.00 C

ATOM 4682 O LEU 286 55.389 55.431 42.643 1.00 0.00 O

ATOM 4683 N TYR 287 56.944 53.906 42.158 1.00 0.00 N

ATOM 4684 H TYR 287 57.522 53.417 41.490 1.00 0.00 H

ATOM 4685 CA TYR 287 57.668 53.966 43.467 1.00 0.00 C

ATOM 4686 HA TYR 287 56.929 53.767 44.244 1.00 0.00 H

ATOM 4687 CB TYR 287 58.598 52.725 43.545 1.00 0.00 C

ATOM 4688 HB2 TYR 287 59.265 52.829 42.689 1.00 0.00 H

ATOM 4689 HB3 TYR 287 59.174 52.978 44.435 1.00 0.00 H

ATOM 4690 CG TYR 287 57.932 51.357 43.603 1.00 0.00 C

ATOM 4691 CD1 TYR 287 57.846 50.708 42.392 1.00 0.00 C

ATOM 4692 HD1 TYR 287 58.553 50.869 41.591 1.00 0.00 H

ATOM 4693 CE1 TYR 287 57.107 49.510 42.370 1.00 0.00 C

ATOM 4694 HE1 TYR 287 57.334 48.783 41.604 1.00 0.00 H

ATOM 4695 CZ TYR 287 56.244 49.186 43.452 1.00 0.00 C

ATOM 4696 OH TYR 287 55.464 48.109 43.509 1.00 0.00 O

ATOM 4697 HH TYR 287 55.587 47.632 42.685 1.00 0.00 H

ATOM 4698 CE2 TYR 287 56.340 49.913 44.652 1.00 0.00 C

ATOM 4699 HE2 TYR 287 55.973 49.471 45.566 1.00 0.00 H

ATOM 4700 CD2 TYR 287 57.051 51.082 44.656 1.00 0.00 C

ATOM 4701 HD2 TYR 287 57.230 51.595 45.589 1.00 0.00 H

ATOM 4702 C TYR 287 58.423 55.282 43.580 1.00 0.00 C

ATOM 4703 O TYR 287 58.520 55.850 44.720 1.00 0.00 O

ATOM 4704 N PHE 288 58.735 55.968 42.504 1.00 0.00 N

ATOM 4705 H PHE 288 58.498 55.596 41.595 1.00 0.00 H

ATOM 4706 CA PHE 288 59.248 57.316 42.367 1.00 0.00 C

ATOM 4707 HA PHE 288 60.174 57.373 42.938 1.00 0.00 H

ATOM 4708 CB PHE 288 59.757 57.637 40.892 1.00 0.00 C

ATOM 4709 HB2 PHE 288 59.896 56.700 40.353 1.00 0.00 H

ATOM 4710 HB3 PHE 288 59.046 58.149 40.243 1.00 0.00 H

ATOM 4711 CG PHE 288 61.115 58.316 41.081 1.00 0.00 C

ATOM 4712 CD1 PHE 288 61.162 59.607 41.661 1.00 0.00 C

ATOM 4713 HD1 PHE 288 60.267 60.170 41.884 1.00 0.00 H

ATOM 4714 CE1 PHE 288 62.375 60.203 42.030 1.00 0.00 C

ATOM 4715 HE1 PHE 288 62.315 61.169 42.511 1.00 0.00 H

ATOM 4716 CZ PHE 288 63.616 59.502 41.682 1.00 0.00 C

ATOM 4717 HZ PHE 288 64.586 59.830 42.024 1.00 0.00 H

ATOM 4718 CE2 PHE 288 63.605 58.253 41.084 1.00 0.00 C

ATOM 4719 HE2 PHE 288 64.457 57.590 41.113 1.00 0.00 H

ATOM 4720 CD2 PHE 288 62.349 57.665 40.789 1.00 0.00 C

ATOM 4721 HD2 PHE 288 62.368 56.599 40.618 1.00 0.00 H

ATOM 4722 C PHE 288 58.219 58.399 42.752 1.00 0.00 C

ATOM 4723 O PHE 288 58.512 59.237 43.610 1.00 0.00 O

ATOM 4724 N LEU 289 56.985 58.329 42.188 1.00 0.00 N

ATOM 4725 H LEU 289 57.000 57.551 41.545 1.00 0.00 H

ATOM 4726 CA LEU 289 55.704 59.009 42.496 1.00 0.00 C

ATOM 4727 HA LEU 289 55.786 60.091 42.392 1.00 0.00 H

ATOM 4728 CB LEU 289 54.621 58.472 41.605 1.00 0.00 C

ATOM 4729 HB2 LEU 289 54.685 57.388 41.695 1.00 0.00 H

ATOM 4730 HB3 LEU 289 53.720 58.772 42.141 1.00 0.00 H

ATOM 4731 CG LEU 289 54.654 58.943 40.128 1.00 0.00 C

ATOM 4732 HG LEU 289 55.645 58.923 39.674 1.00 0.00 H

ATOM 4733 CD1 LEU 289 53.751 58.063 39.234 1.00 0.00 C

ATOM 4734 HD11 LEU 289 54.231 57.109 39.015 1.00 0.00 H

ATOM 4735 HD12 LEU 289 52.846 57.764 39.764 1.00 0.00 H

ATOM 4736 HD13 LEU 289 53.606 58.658 38.333 1.00 0.00 H

ATOM 4737 CD2 LEU 289 54.221 60.429 40.090 1.00 0.00 C

ATOM 4738 HD21 LEU 289 53.598 60.712 40.938 1.00 0.00 H

ATOM 4739 HD22 LEU 289 55.058 61.114 40.224 1.00 0.00 H

ATOM 4740 HD23 LEU 289 53.648 60.733 39.213 1.00 0.00 H

ATOM 4741 C LEU 289 55.410 58.840 44.007 1.00 0.00 C

ATOM 4742 O LEU 289 55.004 59.854 44.658 1.00 0.00 O

ATOM 4743 N ALA 290 55.639 57.632 44.529 1.00 0.00 N

ATOM 4744 H ALA 290 55.952 56.902 43.906 1.00 0.00 H

ATOM 4745 CA ALA 290 55.158 57.263 45.853 1.00 0.00 C

ATOM 4746 HA ALA 290 54.372 57.935 46.196 1.00 0.00 H

ATOM 4747 CB ALA 290 54.711 55.819 45.768 1.00 0.00 C

ATOM 4748 HB1 ALA 290 54.218 55.496 44.852 1.00 0.00 H

ATOM 4749 HB2 ALA 290 55.539 55.120 45.882 1.00 0.00 H

ATOM 4750 HB3 ALA 290 53.934 55.744 46.530 1.00 0.00 H

ATOM 4751 C ALA 290 56.277 57.474 46.964 1.00 0.00 C

ATOM 4752 O ALA 290 56.011 57.397 48.166 1.00 0.00 O

ATOM 4753 N GLY 291 57.507 57.643 46.611 1.00 0.00 N

ATOM 4754 H GLY 291 57.782 57.473 45.654 1.00 0.00 H

ATOM 4755 CA GLY 291 58.692 57.607 47.492 1.00 0.00 C

ATOM 4756 HA2 GLY 291 59.511 57.785 46.794 1.00 0.00 H

ATOM 4757 HA3 GLY 291 58.829 58.473 48.140 1.00 0.00 H

ATOM 4758 C GLY 291 59.004 56.318 48.310 1.00 0.00 C

ATOM 4759 O GLY 291 58.953 56.426 49.519 1.00 0.00 O

ATOM 4760 N ASP 292 59.198 55.149 47.734 1.00 0.00 N

ATOM 4761 H ASP 292 59.324 55.197 46.733 1.00 0.00 H

ATOM 4762 CA ASP 292 59.520 53.884 48.464 1.00 0.00 C

ATOM 4763 HA ASP 292 59.290 53.917 49.529 1.00 0.00 H

ATOM 4764 CB ASP 292 58.542 52.770 47.910 1.00 0.00 C

ATOM 4765 HB2 ASP 292 57.468 52.942 47.839 1.00 0.00 H

ATOM 4766 HB3 ASP 292 58.822 52.647 46.864 1.00 0.00 H

ATOM 4767 CG ASP 292 58.623 51.518 48.745 1.00 0.00 C

ATOM 4768 OD1 ASP 292 58.831 51.629 49.996 1.00 0.00 O

ATOM 4769 OD2 ASP 292 58.544 50.399 48.165 1.00 0.00 O

ATOM 4770 C ASP 292 61.031 53.615 48.431 1.00 0.00 C

ATOM 4771 O ASP 292 61.684 53.774 47.397 1.00 0.00 O

ATOM 4772 N THR 293 61.591 53.503 49.661 1.00 0.00 N

ATOM 4773 H THR 293 60.983 53.278 50.435 1.00 0.00 H

ATOM 4774 CA THR 293 63.028 53.390 49.723 1.00 0.00 C

ATOM 4775 HA THR 293 63.366 54.182 49.054 1.00 0.00 H

ATOM 4776 CB THR 293 63.425 53.844 51.176 1.00 0.00 C

ATOM 4777 HB THR 293 64.473 53.613 51.364 1.00 0.00 H

ATOM 4778 CG2 THR 293 63.273 55.437 51.360 1.00 0.00 C

ATOM 4779 HG21 THR 293 62.310 55.800 50.999 1.00 0.00 H

ATOM 4780 HG22 THR 293 63.394 55.651 52.422 1.00 0.00 H

ATOM 4781 HG23 THR 293 64.085 55.934 50.831 1.00 0.00 H

ATOM 4782 OG1 THR 293 62.683 53.331 52.313 1.00 0.00 O

ATOM 4783 HG1 THR 293 63.314 53.264 53.034 1.00 0.00 H

ATOM 4784 C THR 293 63.661 52.017 49.416 1.00 0.00 C

ATOM 4785 O THR 293 63.854 51.202 50.314 1.00 0.00 O

ATOM 4786 N PHE 294 63.725 51.583 48.179 1.00 0.00 N

ATOM 4787 H PHE 294 63.499 52.143 47.369 1.00 0.00 H

ATOM 4788 CA PHE 294 64.076 50.160 47.888 1.00 0.00 C

ATOM 4789 HA PHE 294 63.223 49.511 48.087 1.00 0.00 H

ATOM 4790 CB PHE 294 64.224 50.039 46.379 1.00 0.00 C

ATOM 4791 HB2 PHE 294 64.530 50.985 45.933 1.00 0.00 H

ATOM 4792 HB3 PHE 294 64.970 49.262 46.209 1.00 0.00 H

ATOM 4793 CG PHE 294 62.942 49.613 45.679 1.00 0.00 C

ATOM 4794 CD1 PHE 294 61.926 50.581 45.532 1.00 0.00 C

ATOM 4795 HD1 PHE 294 62.258 51.587 45.744 1.00 0.00 H

ATOM 4796 CE1 PHE 294 60.607 50.233 45.172 1.00 0.00 C

ATOM 4797 HE1 PHE 294 59.827 50.980 45.174 1.00 0.00 H

ATOM 4798 CZ PHE 294 60.305 48.903 44.930 1.00 0.00 C

ATOM 4799 HZ PHE 294 59.272 48.611 44.813 1.00 0.00 H

ATOM 4800 CE2 PHE 294 61.347 47.901 45.025 1.00 0.00 C

ATOM 4801 HE2 PHE 294 61.153 46.852 44.854 1.00 0.00 H

ATOM 4802 CD2 PHE 294 62.607 48.262 45.502 1.00 0.00 C

ATOM 4803 HD2 PHE 294 63.290 47.433 45.614 1.00 0.00 H

ATOM 4804 C PHE 294 65.384 49.666 48.508 1.00 0.00 C

ATOM 4805 O PHE 294 65.444 48.534 48.895 1.00 0.00 O

ATOM 4806 N ARG 295 66.465 50.522 48.440 1.00 0.00 N

ATOM 4807 H ARG 295 66.363 51.484 48.149 1.00 0.00 H

ATOM 4808 CA ARG 295 67.574 50.447 49.405 1.00 0.00 C

ATOM 4809 HA ARG 295 67.900 49.408 49.347 1.00 0.00 H

ATOM 4810 CB ARG 295 68.753 51.244 48.770 1.00 0.00 C

ATOM 4811 HB2 ARG 295 68.346 52.187 48.406 1.00 0.00 H

ATOM 4812 HB3 ARG 295 69.390 51.554 49.598 1.00 0.00 H

ATOM 4813 CG ARG 295 69.584 50.637 47.610 1.00 0.00 C

ATOM 4814 HG2 ARG 295 69.890 49.635 47.912 1.00 0.00 H

ATOM 4815 HG3 ARG 295 68.904 50.576 46.760 1.00 0.00 H

ATOM 4816 CD ARG 295 70.837 51.547 47.156 1.00 0.00 C

ATOM 4817 HD2 ARG 295 71.486 51.688 48.021 1.00 0.00 H

ATOM 4818 HD3 ARG 295 71.323 50.955 46.380 1.00 0.00 H

ATOM 4819 NE ARG 295 70.287 52.835 46.575 1.00 0.00 N

ATOM 4820 HE ARG 295 69.281 52.841 46.491 1.00 0.00 H

ATOM 4821 CZ ARG 295 70.912 53.946 46.414 1.00 0.00 C

ATOM 4822 NH1 ARG 295 72.148 54.245 46.563 1.00 0.00 N

ATOM 4823 HH11 ARG 295 72.782 53.514 46.274 1.00 0.00 H

ATOM 4824 HH12 ARG 295 72.482 55.194 46.647 1.00 0.00 H

ATOM 4825 NH2 ARG 295 70.205 54.856 45.764 1.00 0.00 N

ATOM 4826 HH21 ARG 295 69.197 54.837 45.823 1.00 0.00 H

ATOM 4827 HH22 ARG 295 70.769 55.688 45.668 1.00 0.00 H

ATOM 4828 C ARG 295 67.304 50.767 50.905 1.00 0.00 C

ATOM 4829 O ARG 295 67.441 51.882 51.324 1.00 0.00 O

ATOM 4830 N ARG 296 66.451 49.877 51.532 1.00 0.00 N

ATOM 4831 H ARG 296 66.096 49.120 50.965 1.00 0.00 H

ATOM 4832 CA ARG 296 66.310 49.631 52.982 1.00 0.00 C

ATOM 4833 HA ARG 296 65.968 50.523 53.506 1.00 0.00 H

ATOM 4834 CB ARG 296 65.343 48.444 53.105 1.00 0.00 C

ATOM 4835 HB2 ARG 296 65.698 47.518 52.652 1.00 0.00 H

ATOM 4836 HB3 ARG 296 65.375 48.169 54.159 1.00 0.00 H

ATOM 4837 CG ARG 296 63.859 48.651 52.690 1.00 0.00 C

ATOM 4838 HG2 ARG 296 63.845 48.797 51.610 1.00 0.00 H

ATOM 4839 HG3 ARG 296 63.237 47.828 53.043 1.00 0.00 H

ATOM 4840 CD ARG 296 63.281 50.001 53.186 1.00 0.00 C

ATOM 4841 HD2 ARG 296 63.718 50.164 54.171 1.00 0.00 H

ATOM 4842 HD3 ARG 296 63.511 50.876 52.577 1.00 0.00 H

ATOM 4843 NE ARG 296 61.817 49.788 53.208 1.00 0.00 N

ATOM 4844 HE ARG 296 61.453 49.422 54.076 1.00 0.00 H

ATOM 4845 CZ ARG 296 60.834 50.209 52.320 1.00 0.00 C

ATOM 4846 NH1 ARG 296 61.043 50.676 51.126 1.00 0.00 N

ATOM 4847 HH11 ARG 296 60.247 50.865 50.534 1.00 0.00 H

ATOM 4848 HH12 ARG 296 61.990 50.728 50.778 1.00 0.00 H

ATOM 4849 NH2 ARG 296 59.717 49.633 52.473 1.00 0.00 N

ATOM 4850 HH21 ARG 296 59.492 48.885 53.113 1.00 0.00 H

ATOM 4851 HH22 ARG 296 59.065 49.713 51.706 1.00 0.00 H

ATOM 4852 C ARG 296 67.648 49.245 53.642 1.00 0.00 C

ATOM 4853 O ARG 296 68.482 48.708 52.955 1.00 0.00 O

ATOM 4854 N ARG 297 67.938 49.374 54.946 1.00 0.00 N

ATOM 4855 H ARG 297 67.189 49.788 55.483 1.00 0.00 H

ATOM 4856 CA ARG 297 69.256 48.999 55.588 1.00 0.00 C

ATOM 4857 HA ARG 297 69.889 48.691 54.756 1.00 0.00 H

ATOM 4858 CB ARG 297 69.897 50.209 56.260 1.00 0.00 C

ATOM 4859 HB2 ARG 297 69.267 50.677 57.017 1.00 0.00 H

ATOM 4860 HB3 ARG 297 70.686 49.810 56.898 1.00 0.00 H

ATOM 4861 CG ARG 297 70.164 51.260 55.154 1.00 0.00 C

ATOM 4862 HG2 ARG 297 69.302 51.513 54.538 1.00 0.00 H

ATOM 4863 HG3 ARG 297 70.381 52.135 55.767 1.00 0.00 H

ATOM 4864 CD ARG 297 71.439 50.987 54.451 1.00 0.00 C

ATOM 4865 HD2 ARG 297 72.224 51.641 54.831 1.00 0.00 H

ATOM 4866 HD3 ARG 297 71.787 49.983 54.693 1.00 0.00 H

ATOM 4867 NE ARG 297 71.347 51.127 52.996 1.00 0.00 N

ATOM 4868 HE ARG 297 70.678 51.782 52.615 1.00 0.00 H

ATOM 4869 CZ ARG 297 71.673 50.323 51.974 1.00 0.00 C

ATOM 4870 NH1 ARG 297 71.569 50.720 50.733 1.00 0.00 N

ATOM 4871 HH11 ARG 297 71.864 51.676 50.591 1.00 0.00 H

ATOM 4872 HH12 ARG 297 71.672 49.990 50.043 1.00 0.00 H

ATOM 4873 NH2 ARG 297 72.272 49.165 52.151 1.00 0.00 N

ATOM 4874 HH21 ARG 297 72.574 48.635 51.346 1.00 0.00 H

ATOM 4875 HH22 ARG 297 72.602 48.945 53.079 1.00 0.00 H

ATOM 4876 C ARG 297 69.206 47.768 56.489 1.00 0.00 C

ATOM 4877 O ARG 297 69.446 46.638 56.028 1.00 0.00 O

ATOM 4878 OXT ARG 297 68.553 47.928 57.505 1.00 0.00 O

TER

ATOM 4879 O2B 6AD 298 46.731 42.665 15.381 1.00 0.00 O

ATOM 4880 PB 6AD 298 47.889 41.765 15.186 1.00 0.00 P

ATOM 4881 O1B 6AD 298 48.342 41.686 13.839 1.00 0.00 O

ATOM 4882 O3B 6AD 298 49.051 42.149 16.046 1.00 0.00 O

ATOM 4883 O3A 6AD 298 47.591 40.358 15.681 1.00 0.00 O

ATOM 4884 PA 6AD 298 46.248 39.678 15.318 1.00 0.00 P

ATOM 4885 O1A 6AD 298 45.484 40.092 14.148 1.00 0.00 O

ATOM 4886 O2A 6AD 298 45.510 39.814 16.580 1.00 0.00 O

ATOM 4887 O5' 6AD 298 46.798 38.171 15.061 1.00 0.00 O

ATOM 4888 C5' 6AD 298 45.902 37.089 15.505 1.00 0.00 C

ATOM 4889 C4' 6AD 298 46.484 36.230 16.738 1.00 0.00 C

ATOM 4890 O4' 6AD 298 46.373 36.997 17.933 1.00 0.00 O

ATOM 4891 C3' 6AD 298 45.632 34.840 16.826 1.00 0.00 C

ATOM 4892 O3' 6AD 298 46.619 33.818 16.604 1.00 0.00 O

ATOM 4893 C2' 6AD 298 45.241 34.745 18.285 1.00 0.00 C

ATOM 4894 O2' 6AD 298 45.703 33.608 18.995 1.00 0.00 O

ATOM 4895 C1' 6AD 298 45.703 36.108 18.948 1.00 0.00 C

ATOM 4896 N9 6AD 298 44.608 36.631 19.694 1.00 0.00 N

ATOM 4897 C8 6AD 298 43.794 37.711 19.398 1.00 0.00 C

ATOM 4898 N7 6AD 298 43.042 38.040 20.450 1.00 0.00 N

ATOM 4899 C5 6AD 298 43.421 37.160 21.396 1.00 0.00 C

ATOM 4900 C4 6AD 298 44.385 36.311 20.957 1.00 0.00 C

ATOM 4901 N3 6AD 298 44.864 35.310 21.651 1.00 0.00 N

ATOM 4902 C2 6AD 298 44.398 35.272 22.925 1.00 0.00 C

ATOM 4903 S1 6AD 298 44.848 33.955 24.058 1.00 0.00 S

ATOM 4904 C6 6AD 298 43.858 32.559 23.334 1.00 0.00 C

ATOM 4905 N1 6AD 298 43.509 36.061 23.531 1.00 0.00 N

ATOM 4906 C7 6AD 298 43.003 36.972 22.728 1.00 0.00 C

ATOM 4907 N6 6AD 298 41.999 37.608 23.344 1.00 0.00 N

ATOM 4908 H30 6AD 298 44.971 37.572 15.811 1.00 0.00 H

ATOM 4909 H31 6AD 298 45.684 36.460 14.638 1.00 0.00 H

ATOM 4910 H32 6AD 298 47.521 35.993 16.486 1.00 0.00 H

ATOM 4911 H33 6AD 298 44.808 34.768 16.110 1.00 0.00 H

ATOM 4912 H34 6AD 298 46.866 33.322 17.405 1.00 0.00 H

ATOM 4913 H35 6AD 298 44.150 34.805 18.281 1.00 0.00 H

ATOM 4914 H36 6AD 298 45.440 33.767 19.919 1.00 0.00 H

ATOM 4915 H37 6AD 298 46.476 35.876 19.695 1.00 0.00 H

ATOM 4916 H38 6AD 298 43.603 37.977 18.370 1.00 0.00 H

ATOM 4917 H39 6AD 298 44.294 32.437 22.340 1.00 0.00 H

ATOM 4918 H40 6AD 298 44.010 31.654 23.929 1.00 0.00 H

ATOM 4919 H41 6AD 298 42.786 32.771 23.304 1.00 0.00 H

ATOM 4920 H42 6AD 298 41.153 37.554 22.787 1.00 0.00 H

ATOM 4921 H43 6AD 298 41.765 37.265 24.269 1.00 0.00 H

END
